# Whole transcriptome targeted gene quantification provides new insights on pulmonary sarcomatoid carcinomas

**DOI:** 10.1038/s41598-019-40016-8

**Published:** 2019-03-05

**Authors:** Greta Alì, Rossella Bruno, Anello Marcello Poma, Ornella Affinito, Antonella Monticelli, Paolo Piaggi, Sara Ricciardi, Marco Lucchi, Franca Melfi, Antonio Chella, Sergio Cocozza, Gabriella Fontanini

**Affiliations:** 1 Unit of Pathological Anatomy, University Hospital of Pisa, Pisa, Italy; 2 Department of Surgical, Medical, Molecular Pathology and Critical Area, University of Pisa, Pisa, Italy; 3 Dipartimento di Medicina Molecolare e Biotecnologie Mediche, Università degli Studi di Napoli “Federico II”, Naples, Italy; 4 Istituto di Endocrinologia ed Oncologia Sperimentale (IEOS) “Gaetano Salvatore”, Consiglio Nazionale delle Ricerche (CNR), Naples, Italy; 5 National Institute of Diabetes and Digestive and Kidney Diseases, National Institutes of Health, Phoenix, Arizone USA; 6 Unit of Thoracic Surgery, University Hospital of Pisa, Pisa, Italy; 7 Unit of Pneumology, University Hospital of Pisa, Pisa, Italy

## Abstract

Pulmonary sarcomatoid carcinomas (PSC) are a rare group of lung cancer with a median overall survival of 9–12 months. PSC are divided into five histotypes, challenging to diagnose and treat. The identification of PSC biomarkers is warranted, but PSC molecular profile remains to be defined. Herein, a targeted whole transcriptome analysis was performed on 14 PSC samples, evaluated also for the presence of the main oncogene mutations and rearrangements. PSC expression data were compared with transcriptome data of lung adenocarcinomas (LUAD) and squamous cell carcinomas (LUSC) from The Cancer Genome Atlas. Deregulated genes were used for pathway enrichment analysis; the most representative genes were tested by immunohistochemistry (IHC) in an independent cohort (30 PSC, 31 LUAD, 31 LUSC). All PSC cases were investigated for PD-L1 expression. Thirty-eight genes deregulated in PSC were identified, among these *IGJ* and *SLMAP* were confirmed by IHC. Moreover, Forkhead box signaling and Fanconi anemia pathways were specifically enriched in PSC. Finally, some PSC harboured alterations in genes targetable by tyrosine kinase inhibitors, as *EGFR* and *MET*. We provide a deep molecular characterization of PSC; the identification of specific molecular profiles, besides increasing our knowledge on PSC biology, might suggest new strategies to improve patients management.

## Introduction

Pulmonary sarcomatoid carcinomas (PSC) are a rare group of lung cancer more frequently occurring in males with a median age of 60–70 years and a history of smoking. PSC tumors are generally characterized by an epithelial and a sarcoma-like component, and they are supposed to be associated with epithelial-mesenchymal transition^1^. According to 2015 World Health Organization (WHO) classification, PSC term refers to five different histological entities: pulmonary blastoma (PB), carcinosarcoma (CS), spindle cell carcinoma (SCC), giant cell carcinoma (GCC) and pleomorphic carcinoma (PLC)^1,2^.

PB is a biphasic tumor that consists of fetal adenocarcinoma and primitive mesenchymal stroma^1^. CS appears as a biphasic neoplasm, with a carcinoma component (typically squamous cell carcinoma or adenocarcinoma) intermingled with heterologous sarcomatous elements, such as osteosarcoma, chondrosarcoma, and rhabdomyosarcoma^1^. SCC and GCC consist almost entirely of spindle cells and epithelial giant tumor cells, respectively, without differentiated carcinomatous elements^1,2^. PLC is defined as a group of poorly differentiated non-small cell lung cancer (NSCLC), including adenocarcinoma, squamous cell carcinoma, and undifferentiated NSCLC, containing epithelial components and at least 10% of sarcomatoid components (giant and/or spindle cells)^1,3^.

The incidence of these tumors ranges from 0.8% to 2–3% of all lung cancer types, with PLC being the most frequent subtype^1,3,4^. The PSC morphological definition is often difficult because of the presence of heterogeneous components and poorly differentiated cells, so an accurate preoperative diagnosis may be extremely challenging. Moreover, specific signs, symptoms or PSC biomarkers have to be still identified^3^.

According to their rarity, PSC have been barely characterized from a molecular point of view and the mutational and transcriptome profiles remain to be clearly elucidated. However, different studies reported that about the 70% of PSC cases is altered in tumor protein p53 gene (TP53)^5,6^; and a significant percentage of PSC cases may present gene alterations similar to other NSCLC types^5,7^. In fact, among the most common driver oncogene mutations in PSC there are those ones involving *KRAS* (30–40% of patients)^5,8^ and *MET* genes (13–20%)^6,9,10^.

Although sharing some driver mutations, compared with other NSCLC types, patients with PSC have a more aggressive clinical course and a poorer prognosis, even at earlier tumor stages, with a median survival of 9–12 months in patients with complete tumor resection^11^. PSC patients usually show an unsatisfying response to systemic chemotherapeutical drugs and, currently, there are no efficient therapies^11^.

In this context, the identification of molecular profiles specific for PSC could provide new insights into the biological mechanisms underlying their growth and progression and favour the development of new specific diagnostic and therapeutic approaches.

In this study, we aimed to deeply characterize PSC tumors in order to identify peculiar molecular alterations. In detail, we analysed a selected series of PSC cases by performing a highly sensitive amplicon-based whole transcriptome quantification using semiconductor sequencing. PSC expression data were then compared with transcriptome data of lung adenocarcinoma (LUAD) and squamous cell carcinoma (LUSC) available on The Cancer Genome Atlas (TCGA) database.

## Results

### Clinical-pathological characteristics and genotyping

The first series of 14 PSC cases, which underwent transcriptomic analysis, was composed of 9 males and 5 females, with an average age of 71 years, ranging from 54 to 81 years. Genotyping results and clinical-pathological characteristics are reported in Table 1.

**Table 1:** Clinical-pathological characteristics of samples selected for transcriptome analysis.

Id	Age	Gender	T-stage	N-stage	Histotype*	Histotype components	Genotype	SLMAP score**	IGJ score**
SL20	67	MALE	T3	N2	GCC	Only giant cells	WT	180	10
SL14	67	FEMALE	T3	N0	PLC	Giant and spindle cells	*BRAF* p.D594V	120	3
SL7	77	FEMALE	T1c	N0	CS	SQC and sarcoma	WT	180	8
SL5	74	FEMALE	T1b	N0	CS	ADC and sarcoma	*PIK3CA* p.H1047R	10	2
SL4	53	MALE	T2a	N0	CS	SQC and sarcoma	*PIK3CA* p.H1047R	10	2
SL30	77	MALE	T2a	N0	CS	SQC and sarcoma	WT	140	12
SL11	74	MALE	T3	N0	PLC	ADC and spindle cells	*MET* p.H997Y	160	3
SL24	61	MALE	T2a	N0	SCC	Only spindle cells	WT	270	1
SL23	73	MALE	T1b	N0	PLC	ADC and spindle cells	*KRAS* p.G12V	60	4
SL2	74	FEMALE	T3	N0	PLC	ADC and spindle cells	*EGFR* p.E746_A750delELREA	15	Not perfomed^***^
SL16	69	FEMALE	T4	N2	PLC	ADC and spindle cells	*ALK* fusion positive****	140	2
SL9	68	MALE	T3	N0	PB	Fetal ADC and primitive stroma	WT	Not performed***	Not performed***
SL21	81	MALE	T4	N2	GCC	Only giant cells	*MET* c.3082 + 1 G > A	80	6
SL3	71	MALE	T2a	N0	SCC	Only spindle cells	*KRAS* p.G13C	160	8

^*^Histotype abbreviations: giant cell carcinoma (GCC), pleomorphic carcinoma (PLC), spindle cell carcinoma (SCC), carcinosarcoma (CS), pulmonary blastoma (PB), squamous cell carcinoma (SQC), and adenocarcinoma (ADC).

**SLMAP and IGJ scores were determined as reported in methods section.

***For these patients available archival material was not enough to perform also immunohistochemistry analyses.

****This patient harboured an *ALK* fusion variant whose specific probes were not included in the used panel. Indeed, NanoString analysis revealed only the presence of an imbalance between the 3′ kinase domain and 5′ exons of *ALK* gene^49^.

### Transcriptome analyses

The differential expression analyses between each tumor dataset and the respective normal control revealed 115, 118 and 275 differentially expressed genes in PSC, LUAD and LUSC respectively. Among these 38, 22 and 152 genes were deregulated only in PSC, LUAD and LUSC respectively, whereas 44 genes were deregulated in all datasets. Deregulated genes are reported in Table 2. FDR and fold changes are reported in Supplementary Table S1A–C.

**Table 2:** Differentially expressed genes determined by edgeR.

Dataset	Number of Deregulated Genes	Gene List
LUAD-LUSC-PSC	44	*SLC39A8, PLAU, CCNB1, ASPM, UBE2S, CRABP2, SPAG5, SUSD2, SPP1, DLC1, STX11, KPNA2, PLK1, AGER, UBE2C, VWF, CD93, PYCR1, CCNB2, EMP2, CDK1, EPAS1, ADRB2, RRM2, CSRNP1, CD52, TOP2A, CCNA2, RASIP1, TTK, LRRK2, ERO1L, PLOD2, MRC1, BUB1, GPR146, PDK4, MCM4, CFD, PTTG1, UBE2T, SLC2A1, HBB, DLGAP5*
LUAD-PSC	3	*PFKP, SULF1, KLF4*
LUSC-PSC	30	*LTBP2, GGH, ARHGAP11A, RGS5, ZFP36, PAICS, KLF2, NEDD9, ALOX5, NR2F1, ALOX5AP, GPX3, DUSP1, DYSF, GATA6, ARRB1, FOS, CYP27A1, SERPING1, CENPH, GAPDH, FANCI, UNC13B, AFAP1L1, CYR61, DSG2, C11orf9, GADD45B, MYADM, AQP1*
LUAD-LUSC	49	*KIAA1524, CKS1B, TK1, ARHGAP31, C15orf48, HMGB3, TGFBR3, LDLR, HIST1H4H, SERPINE2, GRK5, ADAMTS1, EGLN3, LTBP4, H19, NQO1, HIST1H2BD, KIAA1462, IGFBP3, TYMS, TNS1, CTHRC1, UHRF1, PTRF, ZWINT, MDK, KIAA0101, UBD, TBX2, HMGA1, NR4A3, HELLS, FBXO32, SPERCL1, PMAIP1, S100A2, EIF4EBP1, HEG1, MFAP2, MYBL2, CDCA5, GINS2, SMAD6, NUSAP1, AHNAK, KRT17, STEAP1, MMP11, ARNTL2*
PSC	38	*SLMAP, BTG2, BOD1, NDRG2, STOM, CAT, LDHA, ACP5, SPATA8, ENO2, ATF3, PALB2, RRN3P2, LMCD1, MRPS22, DCBLD2, HSPD1, PDLIM4, CTSH, RHOB, GNPNAT1, SLC40A1, CYB5A, CENPN, CD74, DKC1, C10orf54, RNPC3, ELMO1, ITGA1, SLC43A2, DCAF13, APOC1, KARS, IGJ, FHOD3, FANCD2, XRCC2*
LUAD	22	*COL3A1, ADAM8, MARCKSL1, GOLM1, FKBP10, OCIAD2, COL5A2, FHL2, FAP, ABCC3, LOXL2, COL1A1, PPP1R14B, PLAT, CAV2, NT5E, SRPX2, COL5A1, C10orf116, SYNM, AGR2, THBD*
LUSC	152	*FERMT2, PFN2, IL7R, C1orf112, TLR4, AK1, KRT19, FIBIN, WASF1, HSPB1, THOC3, NDUFA4L2, RNASEH2A, ENG, ADARB1, MTHFD2, KANK2, LMO7, SHROOM4, CLCN2, CCDC69, DTL, RAPGEF2, PPP1R15A, JUP, FAM162A, MFSD2A, ARHGEF6, PDK1, TMEM204, ENPP4, LAMB2, IGF2BP2, DSP, DOCK2, NR4A2, PTPRM, NCAPG2, NPM3, ITGA9, CD33, FAM105A, CELSR2, TGM2, DOK2, ACTL6A, S100A4, SMO, A2M, TIMP3, UTRN, SLCO2B1, SRXN1, FNIP2, MYH10, PHGDH, RPP40, NES, SNHG1, PER1, CLDN1, PABPC1L, RASL12, SGOL2, DRAM1, CGN, KNTC1, CD83, FSCN1, CABLES1, IGFBP2, CBR3, ATP11A, CHTF18, GYPC, ALDH2, SIGLEC11, C5orf34, MCM7, SLCO2A1, SRD5A1, KCTD12, TSPAN4, FLI1, DNA2, DDAH1, SFN, GCLM, PRELP, AKAP12, RACGAP1, NCF2, ENPP2, ARHGAP29, C10orf10, AHNAK2, GIMAP6, DOCK4, FEN1, ZNF331, SPTBN1, SNX30, RECK, LOC728554, ABCC5, CYBRD1, SOCS3, H2AFX, SNX25, RFC4, KLHDC7B, ME1, SLC9A3R2, SLC6A8, NEXN, CYBB, IL6ST, AHCYL2, ASNS, SYNE1, LPCAT1, C2, CCDC58, SORD, DAPK1, LOC440173, CCL2, SLC16A1, DSC2, KIF18A, COL13A1, HSPA4L, ITGAL, DOCK11, GPC1, COLEC12, DBF4, FKBP4, EGR1, ZEB2, CKS2, POLR2H, ITM2A, PTGIS, RPS6KA2, HK3, RAB11FIP1, UCK2, CBR1, KCTD1, KLF6, TFPI*

Dataset abbreviations: lung adenocarcinoma-TGCA data (LUAD), lung squamous cell carcinoma-TGCA data (LUSC), pulmonary sarcomatoid carcinoma (PSC).

### Immunohistochemistry

**Figure 1:**
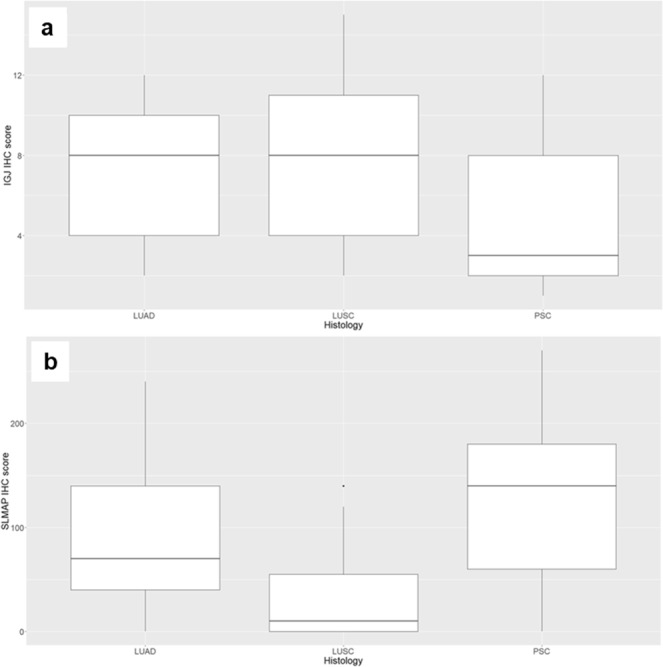
IGJ and SLMAP immunohistochemistry scores. Box-plots of (**a**) IGJ and (**b**) SLMAP immunohistochemistry scores in lung adenocarcinoma (LUAD), lung squamous cell carcinoma (LUSC) and pulmonary sarcomatoid carcinoma (PSC).

Among the 38 genes specifically deregulated in PSC group, 4 genes had a logFC ratio between PSC, LUAD and LUSC over the 90^th^ percentile: sarcolemma associated protein (*SLMAP*), joining chain of multimeric IgA and IgM (*IGJ*), RNA binding region (RNP1, RRM) containing 3 (*RNPC3*) and RRN3 homolog, RNA polymerase I transcription factor pseudogene 2 (*RRN3P2*). According to literature data, *IGJ* and *SLMAP* seemed to be the most relevant in human cancer, and they were selected to validate the differential gene expression by IHC on an independent cohort, including 30 PSC, 31 LUAD and 31 LUSC cases. The number of IGJ positive inflammatory cells infiltrating the tumor was lower in PSC than in LUSC samples (FDR = 0.0015) and in PSC than in LUAD samples (FDR = 0.0035) (Fig. 1a). The expression of SLMAP was higher in PSC (FDR < 0.0001) and LUAD (FDR = 0.0010) in comparison with LUSC samples (Fig. 1b).

Results related to the immunohistochemistry evaluation of PD-L1 expression on tumor and infiltrating immune cells of PSC cases are reported in Table 3. IGJ expression did not correlate with PD-L1, whereas PD-L1 expression levels were correlated in tumor and immune cells (ρ = 0.71, P < 0.0001).

**Table 3:** PD-L1 expression.

Histotype	NO PD-L1 (<1% positive cells)				PD-L1 LOW (1–49% positive cells)				PD-L1 HIGH (>50% positive cells)			
	IC 0	IC 1	IC 2	IC 3	IC 0	IC 1	IC 2	IC 3	IC 0	IC 1	IC 2	IC 3
CS (9)	1	2			2	4						
PLC (21)					3	4	1	1			9	3
GCC (7)	1									1	4	1
SCC (6)	2					1				2	1	
PB (1)		1										

IC, infiltrating immune cells; scored as reported in methods section.

Histotype abbreviations: giant cell carcinoma (GCC), pleomorphic carcinoma (PLC), spindle cell carcinoma (SCC), carcinosarcoma (CS) and pulmonary blastoma (PB).

### Pathway enrichment analysis

**Figure 2:**
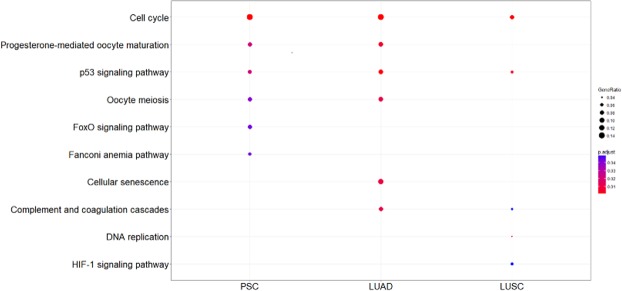
Pathway enrichment results. Enriched pathways using differentially expressed genes in LUAD, LUSC and PSC; Kyoto Encyclopedia of Genes and Genomes (KEGG) as reference database. Gene ratio refers to the number of differentially expressed genes belonging to a specific pathway. PSC, pulmonary sarcomatoid carcinoma; LUAD, lung adenocarcinoma; LUSC, lung squamous cell carcinoma.

Pathway analysis revealed 6, 6 and 5 enriched pathways in PSC, LUAD and LUSC respectively. Cell cycle and p53 signaling pathways were enriched in all groups, Forkhead box (FOXO) signaling and Fanconi anemia (FA) pathways were specifically enriched in PSC. Details are reported in Fig. 2.

## Discussion

PSC are a group of rare and aggressive tumors, which are extremely challenging both to diagnose and to treat^11^.

Preoperative pathological diagnosis, by bronchoscopy or CT-guided fine needle biopsy, may fail to identify these tumors, probably because of their high heterogeneity^1^. Currently, no specific signs, symptoms or biomarkers have been found for PSC in comparison with other NSCLC types. Concerning therapeutic options, the high relapse and low survival rates, even after surgical treatment, weaken the role of surgery itself, which could be considered only in a minority of patients mainly at early stage^11^. Furthermore, PSC are highly resistant to conventional chemotherapy^12^ and only few authors reported an increased overall survival associated with perioperative chemotherapy^11,13^.

PSC have been scarcely studied according to their rarity, and the unresolved diagnostic, prognostic and therapeutic issues underline the urgent need to better define the molecular profiles of these tumors to identify new potential markers.

In this study we characterized the gene expression profiles of a well selected series of 14 PSC cases, followed by a comparison of PSC expression data with transcriptome data of LUAD and LUSC available on TGCA database. The analysed 14 PSC cases included GCC, SCC, CS, PLC and one case of PB. As described in the introduction section, each PSC type is characterized by peculiar aspects, making this group a real heterogeneous one. Particularly, PB appears as the most histogenetically and morphologically different from all the other subtypes, it is a biphasic tumor where the epithelial component is fetal-type adenocarcinoma with a mesenchymal component that is heterologous and/or immature (blastema-like)^14^ and some evidences suggested that blastomas may have unique histologic, immunohistochemical and molecular characteristics^15^. Anyway, all PSC types represent poorly differentiated or dedifferentiated forms of conventional NSCLC. Even though our study cohort includes different PSC histotypes, we considered them as a unique group in the comparison with LUAD and LUSC, because all of them are generally referred to as sarcomatoid pulmonary carcinomas^2^ and, to date, no relevant genetic differences have been reported between them^5^. Moreover, we have performed a principal component analysis using transcriptome data and unsupervised clustering (Supplementary Fig. 1) using the expression levels of the 10.000 genes with the highest variance. In our series histotypes do not significantly influence gene expression profiles.

To analyse gene expression profiles of our samples, we used a targeted whole transcriptome sequencing approach reported as highly accurate and perfectly comparable with RNA-sequencing approaches and next generation sequencing platforms utilized to obtain TCGA data^16^. Although TCGA samples are fresh frozen and we analysed only formalin-fixed and paraffin-embedded (FFPE) tissues, the comparison of transcriptome data is supported by several evidences demonstrating a high correlation of gene expression profiles between these two types of specimens^17–19^. Anyway, in order to overcome this potential limitation, a validation of results was carried out on an independent cohort of PSC, LUAD and LUSC FFPE tissues from our archives.

In detail, the transcriptome analysis identified genes deregulated in PSC, two of which, *IGJ* and *SLMAP*, were confirmed by IHC on the second series on cases.

*IGJ* gene encodes for Immunoglobulin J chain, it is essential for cell development^20^, immunological defence and antibody secreting cells^21^. IGJ is a linker protein for immunoglobulin alpha and mu polypeptides, it links together IgM or IGA monomers to form pentameric IgM or dimeric IgA. IGJ participates in B cell differentiation and activation and it shows a high expression during the last stages of B cell activation^22^. However, it was reported that *IGJ* is transcriptionally active also during early stages of both B cell and intrathymic stages of T cell differentiation, but not in peripheral T cells, monocytes or natural killer cells^23^. In addition, an increased expression of *IGJ* was observed in early B cells in comparison with hemopoietic stem cells and pro-B cells^20^.

Our results demonstrated that it is down-regulated at RNA level and that the number of IGJ positive inflammatory cells infiltrating the tumor is lower in PSC in comparison to other NSCLC types. In some cancers, such as B-acute lymphoblastic leukaemia and Acute Myeloid Leukemia, *IGJ* expression was found to be correlated with prognosis^24,25^ and in hepatocellular carcinoma a high expression of *IGJ* together with *CD5 antigen-like* (CD5L) and galectin-3-binding protein (*LGALS3BP*) was reported to predict response to the chemotherapeutic agent Sorafenib^26^. As regards lung cancer, Martin and collaborators found that this gene could serve as a biomarker for the smoking exposure response^27^. In addition, Kuo and collaborators have recently identified a unique immune gene expression signature of bronchoalveolar lavage cells of tumor-bearing lung segments and tumor adiacent non-neoplastic lung tissues. This signature that included *IGJ* correlated to inhibitory checkpoints expression^28^. Moreover, *IGJ* transcription in the lungs was reported to decrease during tumorigenesis, probably due to the immunosuppressive effects of the tumor cells^26^.

The exact role of *IGJ* in lung cancer has not been definitively identified, but it is clear that the characterization of tumor-infiltrating immune cells has been acquiring an increasing importance, since it could provide crucial information for therapeutic assessment^28^. In the same context we evaluated the expression of PD-L1 on tumor and infiltrating immune cells of all PSC cases included in this study. Interestingly, as shown in Table 3 CS never had a high PD-L1 expression both in the tumor and immune cells. On the other hand, PLC never showed absence of PD-L1 expression in tumor cells. Notably, in our series IGJ and PD-L1 expression were not correlated, whereas PD-L1 levels were positively correlated in tumor and immune cells. Indeed, we cannot draw conclusions with the present data, but it is worth investigating PD-L1 expression across different PSC histotypes.

*SLMAP* gene was up-regulated in PSC only in comparison to LUSC samples. This gene encodes for an integral membrane protein containing C-terminal regions of coiled-coil structure, which is a component of a conserved striatin-interacting phosphatase and kinase complex^29^. The striatin complexes are involved in several cellular processes such as signaling, cell migration, cell cycle control and apoptosis. Moreover, it was demonstrated that depletion of *SLMAP* results in the constitutive activation of the Hippo Pathway, whose disruption was associated with tumorigenesis, cancer progression and tumor immunogenicity^29–31^.

Besides emerging single biomarkers, the pathway enrichment analysis revealed two pathways specifically enriched in PSC: the FOXO signaling pathway and the FA pathway, strongly involved in cancer processes.

FOXO pathway, essential for both cell growth and differentiation, comprises proteins from the Forkhead-box (FOX) family, which consist of a FOX domain and a transactivation domain and include DNA-binding proteins regulating transcription and DNA repair. In detail, FOXO transcription factors have a tumor suppression function, control apoptosis by regulating E2F transcription factor 1 (*E2F1*) transcriptional specificity, enhance the transcription of the pro-apoptotic mediator phorbol-12-myristate-13-acetate-induced protein 1 (*PMAIP1*) and are usually lost in cancer cells^32–34^. In addition, FOXO proteins were reported as key regulators of epithelial-mesenchymal transition^35^, which is involved in the sarcomatoid morphologic change^36^.

FA pathway is responsible for repairing DNA crosslinks and double strand breaks and maintaining chromosomal stability^37^. It was demonstrated that FA pathway activation occurs during DNA replication or upon DNA damage induced by carcinogens in cigarette smoke^38^ or by DNA cross linking agents, such as the chemotherapeutic agents gemcitabine and cisplatin^39^. Chemotherapy still plays an important role in the management of advanced NSCLC and platinum-based agents are the most effective in PSC^3,40^. In this context, it was proved that the up-regulation of FA pathway is linked to acquired resistance to cisplatin. Considering that this pathway is essential for the response to DNA damage, its enrichment in PSC might then explain why these tumors are less susceptible to DNA cross-link based chemotherapy and may suggest new therapeutic strategies based on the inhibition or downregulation of FA, which was already demonstrated to be able to reverse acquired resistance to cisplatin in NSCLC cell lines^41^.

In addition, it was reported that PSC may harbour molecular alterations similar to other NSCLC types^5,7^. Indeed, we found 9 out of 14 patients harbouring alterations in commonly mutated oncogenes, among which 5 had alterations in genes currently recommended for testing in NSCLC, as *MET, ALK*, *EGFR* and *BRAF*^5,6,8–10^. In this study, the analysis of targetable gene alterations seemed to further support a possible involvement of targeted therapy also in this subgroup of patients^6^.

**Figure 3:**
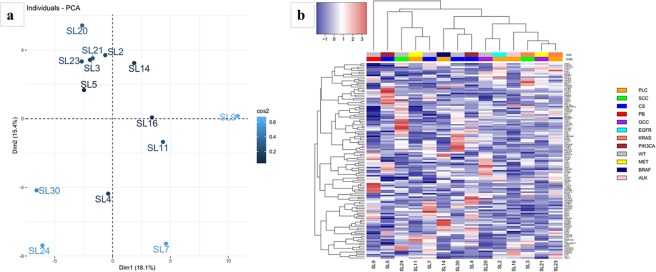
FOXO pathway analyses. (**a**) Principal component analysis and (**b**) hierarchical clustering using the expression levels of 130 genes belonging to FOXO signaling according to KEGG database.

Moreover, some mutated oncogenes may be directly involved in the deregulation of enriched pathways, for instance PIK3-AKT signaling activation leads to functional loss of FOXO transcription factors^32^. In order to investigate such a relationship, we analysed the expression levels of 130 genes belonging to FOXO signaling according to KEGG database. Principal component analysis and hierarchical clustering were performed, and the results are showed in Fig. 3. The two *PIK3CA* mutants (i.e. SL4 and SL5) did not have similar variation pattern nor expression profile, and they did not have altered levels of FOXO mRNA. Nevertheless, this could be explained by the post-translational effect of PI3K/AKT axis on FOXOs. In fact, activating mutations in *PIK3CA* result in increased AKT activity and the consequent phosphorylation of FOXO proteins. Phosphorylated FOXOs are retained in the cytoplasm and eventually degraded^32^. Interestingly, the pulmonary blastoma (PB) case had a peculiar FOXO-gene profile. This is in agreement with the theoretical lower differentiation of PB, since FOXO signaling plays a pivotal role in differentiation and epithelial-mesenchymal transition^35^.

Although this study suffers from some limitations, mainly due to the low number of samples on which transcriptome analysis was performed, the comparison with TCGA data and the IHC validation on an independent cohort let us be quite confident about the reliability of our analyses.

In conclusion, we identified 38 genes specifically deregulated in PSC samples, of which two were validated by IHC as deregulated in comparison with LUSC and LUAD samples, and two pathways with a crucial role in cancer specifically enriched in PSC.

Our findings could open new fields in the knowledge of PSC molecular landscape, and, certainly, they worth further investigation to better delineate their clinical implications in PSC patients’ management.

## Methods

### Study population

This study was retrospectively conducted in accordance to the principles of the Helsinki Declaration of 1975 and it was approved by the ethics committee “Comitato Etico di Area Vasta Nord Ovest”. Only archival and anonymous samples were analyzed, no protected health information was used and informed consents were obtained from patients.

A first series of 14 PSC tissues (surgical specimens) was selected for transcriptome analysis, comprising 1 PB, 4 CS, 2 SCC, 2 GCC and 5 PLC cases; 3 samples of normal lung parenchyma were also included in the transcriptome analysis. The validation of deregulated genes was conducted by IHC on a second series of lung tissues including 30 PSC, 31 LUAD, 31 LUSC surgical samples. Tumor samples were selected from the archives of the unit of pathological anatomy of the University Hospital of Pisa. All tissues were FFPE. The tumors were independently revised and classified by two expert pathologists (GA and GF) according to the current WHO histologic criteria^1^. In detail, the PSC validation cohort included: 4 CS cases with squamous cell carcinoma and sarcoma components, 1 CS case with adenocarcinoma and sarcoma component, 5 GCC cases, 4 SCC cases, 8 PLC cases with adenocarcinoma and spindle cell components, 2 PLC case with adenocarcinoma, spindle and giant cell components, 1 PLC case with adenocarcinoma and giant cell component, 2 PLC cases with giant and spindle cell components, 1 PLC case with squamous and spindle cell components, and 2 PLC cases with squamous, spindle and giant cell components.

Level 3 RNA-Seq data for 125 LUAD samples and 37 respective normal controls and 223 LUSC samples and 17 respective normal controls were downloaded from TCGA database (https://portal.gdc.cancer.gov/). More details about LUAD and LUSC patients are reported in Supplementary Table S2A,B.

### Nucleic acid purification

Only cases with adequate tumor material (>40% tumor cells) and minimal contamination from benign cells were selected for molecular analysis. For each FFPE tissue, three 10 µm thick sections and four 5 µm thick sections underwent standard deparaffinization for DNA and RNA purification, respectively.

All samples were enriched by manual macrodissection. Total DNA and total RNA were isolated using Qiagen Qiamp DNA mini kit and Qiagen RNeasy FFPE kit (Qiagen, Hilden, Germany) respectively, according to the manufacturer’s instructions.

The concentration of total DNA was assessed using a spectrophotometer (ND1000; NanoDrop Technologies, ThermoFisher Scientific, Waltham, MA, USA), whereas the concentration of RNA was determined using a Qubit fluorometer and the Qubit RNA BR assay kit (Life Technologies, Carlsbad, CA, USA). Moreover, each RNA sample was inspected for quality using the RNA 6000 Nano kit by Agilent 2100 Bioanalyzer (Agilent Technologies, Santa Clara, CA, USA).

DNA samples with a concentration greater or equal to 5 ng/µl were considered for molecular analyses. RNA samples with a concentration greater than 10 ng/µl and at least 50% of RNA fragments longer than 200 base pair (bp) were considered adequate for further analyses.

### Analysis of hotspot mutations and gene rearrangements

The mutational status of *KRAS, BRAF, NRAS, PIK3CA, ALK, ERBB2, DDR2, MAP2K1, EGFR* and *RET* was determined by Sequenom Mass-Array (MALDI-TOF MS) using the Myriapod Lung Status Kit (Diatech Pharmacogenetics, Jesi, AN, Italy) and analysis software Massarray Typer 4.0 (Diatech Pharmacogenetics, Jesi, AN, Italy) according to the manufacturer’s protocol. The limit of detection of this assay ranges from 2.5% to 5% for the most frequent mutation hotspots in NSCLC.

To analyse MET exon 14 and its splicing sites, we amplified a 407 bp gene region by polimerase chain reaction (PCR), using a forward (5′-TGTCGTCGATTCTTGTGTGC-3′) and a reverse (5′-TCAAATACTTACTTGGCAGAGGT-3′) primers located in intron 13 at 150 bp upstream and in intron 14 at 140 bp downstream exon 14, respectively. In case of PCR failure, due to a poor DNA quality and high fragmentation levels, we performed two separate PCR reactions, giving amplicons of 207 bp (Forward primer: 5′-TGTCGTCGATTCTTGTGTGC-3′; reverse primer: 5′-CACTTCGGGCACTTACAAGC-3′) and 260 bp lenght (Forward primer: GCTACGATGCAAGAGTACACA-3′; reverse primer 5′-TCAAATACTTACTTGGCAGAGGT-3′). PCR products were purified and used as template for direct sequencing by Sanger on an Abi Prism 3130 Genetic Analyzer (Applied Biosystem - ThermoFisher Scientific, San Francisco, CA, USA).

*ALK, ROS1* and *RET* rearrangements were evaluated using the RealQuant Lung Fusion Genes kit (Diatech Pharmacogenetics, Jesi, Italy) based on the NanoString technology (NanoString technologies, Seattle, WA, USA) and the iGENETICS RealQuant analysis software (Diatech Pharmacogenetics, Jesi, Italy), according to the manufacturer’s instructions. The ALK fusion positive case was confirmed by Fluorescent *In Situ* Hybridization (FISH) as previously reported^42^.

### Human transcriptome gene expression analysis

A targeted whole transcriptome analysis was performed using the Ion Ampliseq Transcriptome Human Gene Expression kit (Life Technologies, Carlsbad, CA, USA). This method relies on a highly sensitive amplicon-based transcript quantification by semiconductor sequencing, allowing the simultaneous amplification and evaluation of more than 20.000 human genes. Ten ng of total RNA were reverse transcribed to cDNA using the Invitrogen SuperScript VILO cDNA Synthesis kit (Applied Biosystem - ThermoFisher Scientific, San Francisco, CA, USA).

Transcriptome libraries were generated according to the manufacturer’s instructions. Briefly, after targets amplification and primers digestion, adapters and molecular barcodes were ligated to the amplicons followed by magnetic bead purification; then libraries were amplified and purified. Libraries were evaluated and quantified using an Agilent 2100 Bioanalyzer (Agilent Technologies, Santa Clara, CA, USA). Multiplexed barcoded libraries were pooled for emulsion PCR on Ion sphere particles using the Ion PI Template OT2 200 kit on the One Touch 2 instrument. An automated Ion OneTouch ES enrichment of template-positive Ion sphere particles was then performed. Finally, samples were loaded on Ion PI chips, and sequenced, using the Ion PI Sequencing 200 kit (ThermoFisher Scientific, Waltham, MA, USA) on the Ion Proton sequencing system (Life Technologies, Carlsbad, CA, USA).

### Immunohistochemistry

IHC analyses were performed on 3 µm thick tissue sections from the second series of samples. Immunoreaction was displayed using the avidin–biotin–peroxidase complex method and peroxidase activity was visualized with diaminobenzidine. Counterstaining was performed with hematoxylin and negative controls were carried out by omitting the primary antibodies. Immunostaining was executed as a fully automated assay using BenchMark XT automated slide stainers (Ventana Medical Systems, Tuscon, AZ, USA). All cases were independently evaluated by 2 pathologists (GA and GF), who were blinded to the clinical-pathological characteristics of the patients; discordant cases were revised until mutual agreement was reached.

**Figure 4:**
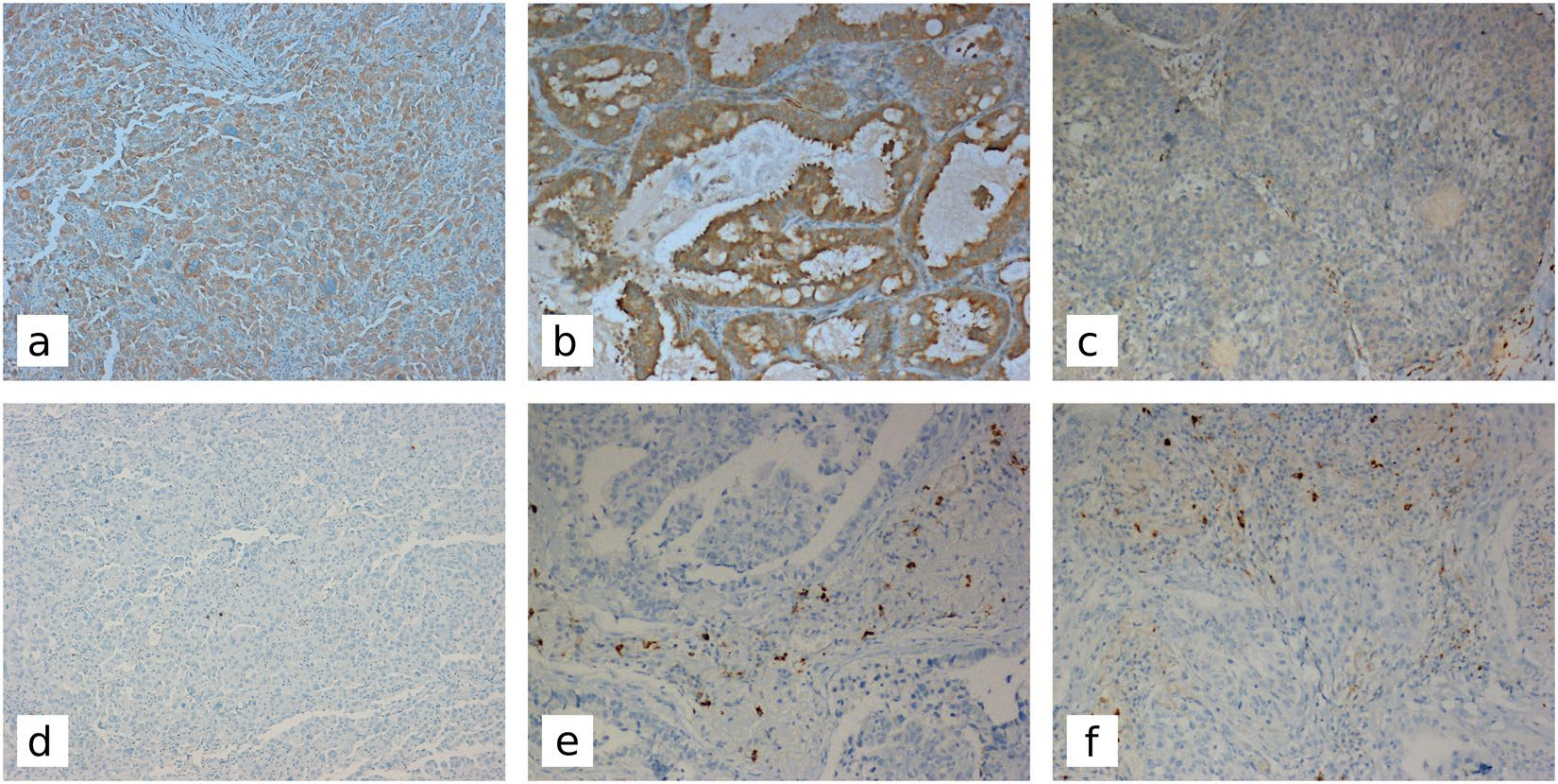
Immunohistochemical staining of SLMAP and IGJ in pulmonary sarcomatoid carcinomas, adenocarcinomas, and squamous cell carcinomas Original magnification x100; higher SLMAP expression levels in giant cell carcinoma (**a**) and adenocarcinoma (**b**) in comparison with squamous cell carcinoma (**c**); Giant cell carcinoma with rare IGJ positive cells (**d**) and two examples of adenocarcinoma (**e**) and squamous cell carcinoma (**f**) showing a higher number of IGJ positive cells.

For SLMAP immunohistochemical staining, the sections were incubated with a mouse antihuman SLMAP monoclonal antibody (clone SJ-09; Santa Cruz Biotechnology, Inc., Dallas, Texas) used at a 1:100 dilution. FFPE human testis tissue showing membrane and cytoplasmic localization was used as positive control. Immunostaining was heterogeneous so all cases were analyzed using a semiquantitative histologic scoring (H score), as previously reported^43^. Briefly, immunostaining intensity of each case was scored as follows: 0, none; 1, weak; 2, moderate; and 3, intense. In addition, the percentage of positive neoplastic cells was evaluated. For each case, a value designated H score was obtained by multiplying each intensity with the corresponding percentage of positive cells, thereby obtaining a final resulting score value (range 0–300) (Fig. 4a–c).

For IGJ immunohistochemistry, sections were incubated with a rabbit monoclonal antibody to IGJ (1:100 dilution; clone SP105; Abcam, Cambridge, UK) using FFPE human tonsil tissue as positive control. IGJ is a secreted protein expressed by inflammatory cells in the plasma cell stage. The relative number of cytoplasmic positive plasma cells infiltrating the tumors was assessed in five randomly chosen 200X microscopic fields and the average of their counts was calculated (Fig. 4d–f).

For PD-L1 immunohistochemical staining, the slides were incubated with a rabbit monoclonal antibody to PD-L1 (clone SP263, Ventana Medical Systems, Tuscon, AZ, USA) according to manufacturer’s instructions. Placental tissue was used as external positive control and inflammatory cells, such as dendritic cells, macrophages, mast cells, and T- and B-lymphocytes, were used as internal control. The PD-L1 expression was evaluated by tumor proportion score (TPS) as previously described^44^. Only viable tumor cells were included in the scoring. All other stained cells, such as tumor-associated immune cells, normal/non neoplastic cells and necrotic cells, were excluded from evaluation. The scoring was interpreted as no-PD-L1 expression (TPS < 1%), low PD-L1 expression (TPS 1–49%), and high PD-L1 expression (TPS ≥ 50%). We also evaluated the immunohistochemical expression of PD-L1 in the population of immune cells (IC) tipically found in the intratumoral and peritumoral regions. The immune cells were scored using the proportion of the tumor area that was occupied by PD-L1-positive immune cells of any intensity, including any PD-L1 staining regardless of the type of immune cell or its location, as previously described^45^. In detail, tumour-infiltrating immune cells were scored as IC3 to IC0 (IC3 ≥ 10%, IC2 ≥ 5% and < 10%, IC1 ≥ 1% and < 5%, and IC0 < 1%).

All evaluations were conducted using a LEICA DMLB microscope (Leica Microsystems Srl, Milan, Italy).

### Bioinformatics analyses

Raw sequencing reads were evaluated on the Torrent Server using a free ampliSeqRNA plug-in that provides quality control, visualization and normalized counts per million for each gene. RNA-seq reads for each library were mapped to the amplicon sequences of the panel (reference human genome hg19) using the same plug-in.

Raw counts for each gene in the UCSC coding gene annotation (hg19) were measured with HTSeq (http://www-huber.embl.de/users/anders/HTSeq/) version 0.5.3p3. Genes with very low counts across all libraries were filtered. In particular, we retained only those genes with at least a count of 10 in all samples. Differential gene expression was first determined between our PSC samples and normal controls; the same analysis was then performed between LUAD and LUSC datasets and their respective normal controls (TCGA data).

In detail, differential expression was detected by edgeR version 2.6.8^46^. Briefly, the raw read count data was first scaled to library size followed by normalizing with weighted trimmed mean of M values (TMM) to consider the compositional bias in sequenced libraries. Then, the dispersion of the reads counts was estimated and an exact test was performed to detect differential expressed mRNAs between the groups. Genes showing an absolute logFC value ≥ 2 and a FDR < 0.05 were defined as differentially expressed.

To select genes with the strongest deregulation in PSC, the ratio between the logFC in PSC with those ones obtained in LUAD and LUSC was calculated. IHC validation was then performed on genes with a ratio over than 90 percentile of this distribution. Differences of IHC scores between groups were estimated by Kruskal-Wallis and Dunn’s test with Benjamini-Hochberg correction for multiple comparison using dunn.test package version 1.3.5^47^. IHC scores of IGJ and PD-L1 (both in tumor and immune cells) were correlated by Spearman’s correlation test. Pathway enrichment analysis was performed on differentially expressed genes by clusterProfiler version 3.6.0^48^, using Kyoto Encyclopedia of Genes and Genomes database as reference.

## Supplementary information

Supplementary info

## Data Availability

Raw expression data of pulmonary sarcomatoid carcinomas generated in this study are available at GEO database with the accession code GSE110205.
