# Supplementary material for: Whole transcriptome targeted gene quantification provides new insights on pulmonary sarcomatoid carcinomas
Source: Sci Rep. 2019 Mar 5;9:3536. doi: 10.1038/s41598-019-40016-8 (PMC6401130; doi:10.1038/s41598-019-40016-8)
Supplement: Supplementary file 1 — Supplementary info [file 41598_2019_40016_MOESM1_ESM.pdf]

Manuscript title: Whole transcriptome targeted gene quantification provides new insights on pulmonary sarcomatoid carcinomas.

List of authors: Greta Alì, Rossella Bruno, Anello Marcello Poma, Ornella Affinito, Antonella Monticelli, Paolo Piaggi, Sara Ricciardi, Marco Lucchi, Franca Melfi, Sergio Cocozza, Gabriella Fontanini

Table S1_A: edgeR results

logFC, log fold change; logCPM, log counts per million; p value; FDR, Benjamini-Hochberg adjusted p value are reported for pulmonary sarcomatoid carcinoma (PSC) dataset

Gene	logFC	logCPM	PValue	FDR
FOS	-4,391524687	8,045383673	7,79761E-16	5,31719E-12
SUSD2	-4,127545784	5,747464472	1,42839E-14	4,8701E-11
PDK4	-3,449094984	6,624040802	2,69633E-14	6,12877E-11
HBB	-4,000959411	8,091884511	2,05019E-13	3,49506E-10
NEDD9	-2,871217174	5,596860504	1,05006E-12	1,43207E-09
CTSH	-2,715608361	7,331559743	1,96072E-11	2,22836E-08
AGER	-4,285545723	6,867738471	1,8499E-10	1,80206E-07
APOC1	-3,35513203	7,197806891	2,40273E-10	2,04803E-07
EMP2	-2,805607995	7,603382253	5,41029E-10	4,0992E-07
SPATA6	-2,617382393	4,127477021	7,87138E-10	5,36749E-07
ZFP36	-2,884071812	9,174200917	2,17542E-09	1,30746E-06
GPX3	-2,994025193	8,141118342	2,30085E-09	1,30746E-06
BTG2	-3,290054256	7,610240201	4,92853E-09	2,5852E-06
DUSP1	-2,736006837	8,751426621	8,25714E-09	3,80031E-06
CSRNP1	-2,341479304	5,3874307	8,35968E-09	3,80031E-06
GPR146	-2,311391013	5,499867416	1,4082E-08	6,00155E-06
CAT	-2,378898336	6,917650559	3,88206E-08	1,55716E-05
CYP27A1	-2,713467271	5,686629346	4,81395E-08	1,82369E-05
NDRG2	-2,523475475	5,531125924	6,00335E-08	2,15457E-05
DYSF	-2,389744178	4,517937154	8,40812E-08	2,86675E-05
ELMO1	-2,427718926	5,124116645	9,06177E-08	2,94249E-05
UNC13B	-2,408529453	5,728288659	1,04159E-07	3,22846E-05
ARRB1	-2,088010274	5,499353335	1,22157E-07	3,6217E-05
KLF2	-2,942019041	6,968589682	1,28714E-07	3,65708E-05
GADD45B	-2,612805863	6,376825297	1,76336E-07	4,80974E-05
LRRK2	-3,245531901	6,66374042	2,05472E-07	5,38889E-05
DLGAP5	3,997663389	7,049186818	2,62693E-07	6,3635E-05
AQP1	-3,159775986	8,303307702	2,66389E-07	6,3635E-05
STX11	-2,497678505	4,903315836	2,70628E-07	6,3635E-05
SLC43A2	-2,063294183	5,470827032	4,3578E-07	9,65824E-05
VWF	-2,561108735	7,567919633	4,39075E-07	9,65824E-05
NR2F1	-2,225456359	5,568963679	6,0945E-07	0,00012987
STOM	-2,506296536	7,294282291	8,7299E-07	0,000180392
IGJ	-3,89224826	8,959316905	9,90853E-07	0,000194547
ALOX5	-2,296402161	5,914557486	9,98555E-07	0,000194547
SLC39A8	-2,290511124	6,504750553	1,23302E-06	0,000233554
CFD	-2,738931404	5,583585933	1,61492E-06	0,000297625
LMCD1	-2,067895202	6,276733312	1,85069E-06	0,000332101
UBE2T	2,948268179	6,669936105	2,05243E-06	0,00035886
CCNB2	3,242707839	5,53469099	2,17128E-06	0,000370149
SLC2A1	3,606267485	7,812898272	3,14404E-06	0,000522907
ALDH2	-1,91687292	8,014956142	3,49603E-06	0,000567605
ATF3	-2,015824563	6,471671168	4,23065E-06	0,000670903

ITGA1	-2,145623168	6,604416731	4,79264E-06	0,00074275
ARHGAP31	-1,818569625	5,781784523	5,09394E-06	0,000761693
AFAP1L1	-2,479699337	5,354355252	5,13827E-06	0,000761693
PLK1	2,856930473	5,307467889	6,78582E-06	0,000984521
CCNB1	2,911810141	6,125608609	9,04827E-06	0,001285419
MYO6	-1,811862102	5,721703075	1,05719E-05	0,001471225
RAPGEF2	-1,731198885	3,465351772	1,1715E-05	0,00159769
C10orf54	-2,093500746	5,134933767	1,2163E-05	0,001626268
RASIP1	-2,217822277	6,300836319	1,2921E-05	0,001689669
RNPC3	-2,186001306	6,98543433	1,31328E-05	0,001689669
HLX	-1,983927014	4,690954795	1,36219E-05	0,001720139
CD93	-2,186838848	6,664975604	1,41273E-05	0,001751524
SLC40A1	-2,004693269	7,20736294	1,51955E-05	0,001843651
EPAS1	-2,02350967	7,346392739	1,54111E-05	0,001843651
PIK3C2B	-1,909009875	5,145804139	1,6757E-05	0,001970099
ENG	-1,995800814	6,769932597	1,85867E-05	0,002148186
CD52	-2,633306778	7,911422351	1,96221E-05	0,002230055
FAM82A2	-1,696816467	4,694275328	2,06979E-05	0,002313755
RALGAPA2	-1,870577783	5,953933761	2,21095E-05	0,002431693
SEPP1	-1,912232356	8,978322774	2,31965E-05	0,00247336
LIMD1	-1,696600163	5,373784683	2,32138E-05	0,00247336
SERPING1	-2,153824728	8,697498936	2,47071E-05	0,002591966
TTK	2,936746972	5,238217894	2,71115E-05	0,002801107
ACP5	-2,417644911	6,528001248	3,00302E-05	0,003056361
DLC1	-2,539849543	6,374775938	3,26306E-05	0,003272175
SPTBN1	-1,734307416	9,122996963	3,81461E-05	0,003769827
GATA6	-2,189649998	5,272339617	4,01323E-05	0,003909464
UBE2C	3,49705508	7,699064843	4,63607E-05	0,004452589
PYCR1	2,869070268	6,467020901	5,15707E-05	0,004884172
RRM2	2,973014126	7,483990249	5,51376E-05	0,005099645
LTBP2	-2,247625779	6,084412083	5,53415E-05	0,005099645
ZNF641	-1,526160158	3,464394377	6,35414E-05	0,005777185
ARHGAP11A	2,912288237	5,837477582	6,7478E-05	0,006054372
MLLT4	-1,426309879	6,608174847	6,92801E-05	0,006135334
EPB41L1	-1,607761459	5,14885004	7,04649E-05	0,006160259
CD74	-2,295229627	8,201516364	7,18341E-05	0,006200464
APLP2	-1,911260402	8,08987779	7,78785E-05	0,006638172
ADRB2	-2,210728856	5,132323464	8,298E-05	0,006985684
CYR61	-2,075032627	7,796100932	8,56555E-05	0,007122986
SOCS3	-1,948027854	6,815079233	0,00010136	0,008327361
CFLAR	-1,562471229	6,647367022	0,000103032	0,008363981
XRCC2	2,669232523	4,907749021	0,000106058	0,008508349
PFKP	2,060128684	6,103114272	0,000113538	0,008894181
DCAF13	2,253850546	6,45006168	0,000114662	0,008894181
DKC1	2,215829769	6,562710528	0,00011478	0,008894181
LILRA6	-1,889021876	5,179821479	0,000118194	0,009020536
HAGH	-1,587481853	4,451987337	0,000119057	0,009020536
CCNA2	2,293281747	6,431522066	0,000123103	0,00922461
KARS	2,03596428	5,802834356	0,000132207	0,009799091
TCP11L2	-1,42331118	4,564569781	0,000135708	0,009950434

MYADM	-2,012312461	7,021555665	0,00014112	0,010237186
UTRN	-1,635703202	7,069111106	0,000152917	0,010976234
TOP2A	2,602844452	6,740759562	0,000165594	0,011762356
JUNB	-1,909522544	9,49610397	0,000170657	0,011996993
MRC1	-2,098848382	6,906417967	0,000179443	0,012422491
ASAH1	-1,982563959	6,828055512	0,000180353	0,012422491
DOCK9	-1,799978582	6,025520287	0,000198956	0,013566824
SIK2	-1,55715178	4,698976274	0,000207785	0,014028552
BUB1	3,389734226	6,009651949	0,0002133	0,014259705
EFEMP1	-1,773306334	6,76281159	0,000223306	0,014783725
ALOX5AP	-2,157106927	6,679022385	0,000236721	0,01552114
CYB5A	-2,006590095	6,511205525	0,000240409	0,015612862
GRK5	-1,431961103	6,175793407	0,000245381	0,015785397
CENPN	2,645319543	5,7618525	0,000248387	0,015829478
RUFY3	-1,49484097	4,630902987	0,000253938	0,016033362
BDH2	-1,340809325	5,526308505	0,00026731	0,016481117
PTTG1	2,394287861	6,352129736	0,000267879	0,016481117
HLA-DMA	-1,693259871	6,788991261	0,00026828	0,016481117
SORBS3	-1,635652661	6,52959648	0,000275386	0,016750309
CRABP2	3,948748159	6,799560613	0,000278525	0,016750309
RRN3P2	-2,194198104	5,673095149	0,000280032	0,016750309
LAIR1	-1,545858818	6,195038698	0,000301878	0,017900045
KLF4	-2,006963257	6,553448136	0,000322126	0,018935995
ARRDC4	-1,656340794	4,836814109	0,000343187	0,019947529
BOD1	2,208016266	6,355453318	0,000345184	0,019947529
ATP6V0C	-1,581323409	7,275685302	0,000369592	0,02117853
SLC20A1	1,938769834	7,819363442	0,000407786	0,023094118
LRP1	-1,558762718	7,519925568	0,000409794	0,023094118
ID2	-1,45781113	6,186281633	0,000434389	0,024183022
GIMAP6	-1,700125471	5,31203855	0,000441371	0,024183022
ASPM	2,653583072	5,889523375	0,000443112	0,024183022
PDLIM4	2,40368338	5,595260611	0,000443302	0,024183022
HYAL2	-1,637175204	6,187130106	0,000449389	0,024218757
NDST1	-1,507882714	6,763963797	0,000451061	0,024218757
FANCD2	2,269971786	5,747268747	0,000461469	0,024571261
TXNDC5	-1,363504008	8,136925824	0,000466911	0,024571261
KLF10	-1,868450994	7,48308076	0,000468436	0,024571261
NR2F2	-1,680059749	6,232257367	0,000477384	0,024722134
HSPD1	2,373279356	9,324723675	0,000480403	0,024722134
CYGB	-1,996735663	4,358015206	0,000482189	0,024722134
HSD17B11	-1,574902558	5,918215973	0,000495015	0,025190363
UBE2S	2,015168666	7,680290909	0,000500415	0,025276494
EGR2	-1,877937925	5,69508922	0,00050882	0,025512062
GNPNAT1	2,112050729	7,524093401	0,000532826	0,02652073
C1orf198	-1,642124045	6,799300089	0,000543833	0,026872457
FHOD3	2,863887275	4,059716307	0,000548074	0,026887154
TJP1	-1,677413175	6,566333635	0,000555718	0,027067437
NBEAL1	-1,617830697	5,536020661	0,000578022	0,027954116
C11orf9	-2,07785671	4,546733254	0,000588812	0,028145503
KITLG	-1,766774248	5,908873427	0,000590234	0,028145503

OAZ1	-1,447008686	7,636708809	0,000629567	0,029812616
MSH6	1,840213109	7,129399071	0,000639043	0,030052643
ERO1L	2,336131211	7,640206522	0,000674985	0,031525486
SULF1	2,9220627	7,916235852	0,000687643	0,031898211
RHOB	-2,096940587	9,410806366	0,000696381	0,032085279
VASH1	-1,752131909	6,125189745	0,000733643	0,033193086
SPP1	3,694858892	8,752702194	0,000747276	0,033193086
GGH	2,508400015	6,317147643	0,000753915	0,033193086
PROS1	-1,480149602	5,889698078	0,000756589	0,033193086
CCT6A	1,826245103	8,596988318	0,000760699	0,033193086
HMOX1	-1,545845128	7,094074602	0,000761506	0,033193086
MFSD2A	-1,838636599	5,288411858	0,000762689	0,033193086
SH3D19	-1,55063992	8,116514929	0,000765012	0,033193086
PLAU	2,881126642	6,985169183	0,000768689	0,033193086
MED10	1,919443367	4,1198774	0,000771939	0,033193086
SPAG5	2,3674237	5,782532003	0,00077397	0,033193086
KPNA2	2,041333957	9,289835257	0,000796353	0,033939564
RECK	-1,418610047	3,855958754	0,000809052	0,033978703
MIDN	-1,827228996	4,675150954	0,000809405	0,033978703
AES	-1,450704795	8,498901794	0,00081222	0,033978703
F11R	-1,672217892	7,511855344	0,000825181	0,034310438
ACO1	-1,528572035	5,649561618	0,000842237	0,034807377
ENPP2	-1,667531343	6,811714381	0,000855486	0,035048073
MASTL	1,67712948	5,209689817	0,000862547	0,035048073
PIAS1	-1,21560856	6,183016639	0,000866702	0,035048073
LITAF	-1,5093139	6,766436145	0,000868621	0,035048073
NCAPG2	1,901550968	6,438376496	0,00087765	0,035071339
NAA20	1,784922649	7,689982851	0,000879484	0,035071339
SNRK	-1,317033411	5,372652347	0,000890947	0,035321905
ETS2	-1,50782664	7,753356775	0,000899095	0,035324556
RGS5	-2,030053248	6,814924918	0,000901459	0,035324556
EMP1	-1,822897708	7,460106006	0,000906555	0,035324556
PALB2	2,183267968	5,38660206	0,000928753	0,035983893
HERPUD1	-1,433264155	7,766393143	0,000937648	0,036081676
PSMD7	1,636200543	7,184200875	0,000941859	0,036081676
PNPLA2	-1,404322069	6,308838524	0,000948188	0,036121203
ERI2	1,942023516	4,691661748	0,000962505	0,036462896
RAB11FIP1	-1,612735499	7,172723452	0,000974248	0,036676574
FAM162A	1,689597327	7,155223764	0,000978903	0,036676574
GAPDH	2,130086352	13,04327338	0,00102332	0,038131253
TSPAN4	-1,361829108	5,031232799	0,001052994	0,0387519
SEMA3F	-1,886474284	4,779403598	0,001053086	0,0387519
RBMS3	-1,663698583	5,350599067	0,001057025	0,0387519
PLOD2	2,290990624	8,069412171	0,001064795	0,038828004
BAZ2B	-1,425465856	5,020917638	0,001079593	0,038868326
DNAJC9	1,677664387	5,837684846	0,001085499	0,038868326
FOXO1	-1,460653318	6,020890396	0,001086831	0,038868326
CENPH	2,303916102	4,423011723	0,001089306	0,038868326
RAD23B	1,625745638	7,967212072	0,001095678	0,038868326
FANCI	2,239959833	4,275438434	0,001105227	0,038868326

CCT4	1,897427071	9,317884464	0,001107145	0,038868326
KIAA0247	-1,311838314	6,69289926	0,001111501	0,038868326
TBL1XR1	1,691612175	8,542664924	0,0011484	0,039820407
SLMAP	2,23885055	6,407165732	0,001150406	0,039820407
TAOK2	-1,45016247	5,265809665	0,001161834	0,039893723
ARRB2	-1,185510237	7,063073519	0,001164225	0,039893723
CDK1	2,237886564	6,601449654	0,001195235	0,04075154
SNHG1	1,971086704	6,673796187	0,001245216	0,04206039
RACGAP1	1,911084	6,665107468	0,001247273	0,04206039
LDHA	2,064720579	10,42710335	0,001263066	0,04206039
PAICS	2,108608592	5,993675805	0,001263486	0,04206039
C2	-1,513516932	6,429946868	0,001270312	0,04206039
LPCAT1	-1,432053083	8,43788918	0,001275447	0,04206039
DNAJC7	1,763441088	7,599952881	0,0012768	0,04206039
CNN2	-1,450573272	7,830968789	0,001283148	0,042066276
GRN	-1,304249677	9,450403152	0,001303884	0,042541551
SNX30	-1,460367511	5,956755002	0,001320551	0,042880187
RNASEH1	1,782851454	5,956207548	0,001331566	0,043032918
DCBLD2	3,82795288	8,061624091	0,001354755	0,043575833
DTYMK	1,639093525	5,686838943	0,001393435	0,044609558
ENO2	2,136830916	7,61281086	0,001411307	0,044906893
DSG2	2,749704414	7,139012729	0,001415894	0,044906893
POLR2H	1,769661641	7,13302314	0,001488797	0,047000489
RPS6KA5	-1,599750605	3,606058354	0,001510749	0,047060825
MRPS22	2,250503199	6,414467425	0,001511122	0,047060825
ABLIM1	-1,508031915	5,030102288	0,001511412	0,047060825
HLA-DPB1	-1,684441441	8,34076537	0,00152354	0,047222815
C10orf11	-1,281725971	4,662174216	0,001541468	0,04756232
SNRPE	1,90384331	8,471979104	0,001579416	0,04831116
NFATC3	-1,343336161	7,002686279	0,001579907	0,04831116
HSD17B4	-1,309351478	6,292559701	0,001601108	0,048740866
MCM4	2,966665313	7,663777461	0,001609189	0,048769142
SNRPA1	1,571478656	7,733868234	0,001640219	0,049489626
MAPK3	-1,149347614	6,716725158	0,001655565	0,049591796
PARL	1,622688307	6,513554461	0,001658151	0,049591796
SLC25A38	-1,367209034	4,638828925	0,00166724	0,049645881
RIN3	-1,40782119	5,750618024	0,001729136	0,051157868
SLC16A3	1,660961103	7,602711804	0,001733021	0,051157868
KIAA0101	2,356465063	6,012064151	0,001755488	0,051592939
PAPD7	1,46571638	5,675345872	0,001762891	0,051592939
TBX2	-1,851351962	6,415553514	0,00179395	0,052277555
FLI1	-1,33188269	5,078178071	0,001814575	0,052653565
HN1	1,675353278	7,664483986	0,001860082	0,053527125
GPCPD1	-1,341936091	6,386770237	0,00186038	0,053527125
MPP6	1,699674302	6,012559387	0,00187649	0,053763799
GOLM1	1,904762781	8,035593297	0,001910109	0,054317511
MCAM	-2,215075552	7,082136927	0,00191914	0,054317511
ANAPC4	-1,323432475	4,759098755	0,001919713	0,054317511
MOAP1	-1,184659686	5,366151279	0,001960804	0,055250919
GLUL	-1,346371066	7,444002647	0,001974051	0,055360513

ARNTL2	4,371632377	6,834559445	0,001980931	0,055360513
LOC146880	-1,350235607	5,43153914	0,002012611	0,056007975
CDCA5	2,544030588	4,927093195	0,002026152	0,056007975
PARD6B	-1,605812694	4,479744823	0,002028739	0,056007975
EPB41L5	-1,54371685	4,064345773	0,002048218	0,056313554
QSOX1	-1,28195022	7,570952261	0,002056324	0,056313554
PRDX4	1,664796853	8,093306805	0,002095648	0,056967881
ZWINT	2,239431626	6,363547211	0,002104554	0,056967881
PLSCR4	-1,696191832	6,259964774	0,00210528	0,056967881
CKAP2	2,145051023	5,150953784	0,002155908	0,057814398
FEZ1	-1,491361152	4,988473689	0,002173124	0,057814398
PRKDC	1,947811162	8,43958429	0,002184455	0,057814398
TBC1D4	-1,789447089	5,213559225	0,002184554	0,057814398
CIRBP	-1,280761097	7,912923816	0,002186214	0,057814398
CDK4	1,704564081	7,528720941	0,002187434	0,057814398
NOP16	1,60426131	5,906838264	0,002246155	0,058850823
PDCD5	2,092546589	9,065565043	0,002250051	0,058850823
MRPL11	1,92091279	6,556749352	0,002260969	0,058850823
SLC15A3	-1,463218195	5,733538553	0,00226117	0,058850823
PTGER4	-1,704980693	5,051003977	0,002281653	0,059158141
RPS6KA2	-1,69262467	6,152924667	0,002347303	0,060629759
CCT5	1,672338641	8,874104398	0,002381434	0,061117051
AK1	-1,451441246	7,542912492	0,002384094	0,061117051
ELF3	-1,961249878	5,910792127	0,002395096	0,061169128
PMAIP1	2,34911584	5,88036297	0,002456903	0,06238962
ATXN1	-1,383341741	4,765517937	0,002461183	0,06238962
ATP11A	-1,672292048	6,032085742	0,002486736	0,062803896
LAGE3	1,757789633	6,849272906	0,00251775	0,063352534
PCYOX1	-1,301916758	5,810273077	0,0025313	0,06345931
XRCC3	1,929762554	4,990542892	0,002555673	0,063835663
SYNE1	-1,739867599	5,587220383	0,002601866	0,064601216
RPRD1A	1,519050476	6,671669442	0,00260527	0,064601216
NR4A2	-1,84463925	5,285186429	0,002622926	0,064803383
NUP155	1,49413737	7,378115545	0,002693421	0,066304835
PDCD10	1,785789621	8,058391337	0,002716192	0,066624867
OLA1	1,631033761	7,214518315	0,00273878	0,066714063
SAMHD1	-1,52557759	6,764791148	0,002754405	0,066714063
SGSM2	-1,27662592	5,117364757	0,002758912	0,066714063
SMC4	1,70658511	8,013273689	0,002766225	0,066714063
IFI30	-1,463668744	7,958873157	0,002777786	0,066714063
LRBA	-1,321943355	6,021812784	0,00277853	0,066714063
MYO5C	-1,788119792	5,74717517	0,002806942	0,067159777
RAN	1,632077171	9,811115559	0,002844103	0,067810976
CEP72	1,560972847	5,515009602	0,002882614	0,068489702
LIPA	-1,396725513	6,715584457	0,002911222	0,068841983
SCNM1	1,315978612	6,020190455	0,002917632	0,068841983
H1F0	2,617749873	9,504164588	0,002971873	0,069804973
RFX5	-1,259556026	4,506533778	0,002989419	0,069804973
TWF1	2,056467118	8,252494477	0,002997674	0,069804973
TBC1D9	-1,269781272	5,068543835	0,002999392	0,069804973

CREBZF	2,163175518	8,522297416	0,003095194	0,071789541
YWHAZ	1,950930667	6,675841946	0,00321071	0,074216386
FIGNL1	2,151986732	5,39833373	0,003258324	0,075062541
TNS1	-1,905949302	8,79205603	0,003281289	0,075337071
PLK2	-1,405801888	5,876341818	0,003340542	0,076178554
JUND	-1,500564954	7,123006657	0,003342249	0,076178554
ITM2A	-1,858721791	5,414557333	0,003351454	0,076178554
PFDN4	2,144409835	6,825888746	0,003425531	0,076947644
WDR76	1,984477416	5,626437378	0,003436257	0,076947644
PLXNA2	-1,403071727	5,791955844	0,003436592	0,076947644
ITGAX	-1,278668011	5,173676455	0,003437532	0,076947644
NCAPD2	2,189424421	7,189691891	0,003441712	0,076947644
PMEPA1	2,134900555	7,559835103	0,00346953	0,077316099
PPP1R14B	1,761947326	8,667456567	0,00350098	0,077627259
C5orf43	1,567070265	6,815518208	0,003513331	0,077627259
APP	-1,155798882	8,162452193	0,003517645	0,077627259
DSP	2,330432196	7,284783916	0,003554386	0,078185022
GAB1	-1,561935836	6,700621605	0,003617459	0,079316567
NAA38	2,170202559	7,639342475	0,003629445	0,079324318
MRPS23	1,603327382	6,061307971	0,00364538	0,079418033
RGL1	-1,188514198	5,496565848	0,003669983	0,079506661
A2M	-2,150199429	10,67432295	0,003672767	0,079506661
N4BP2L1	-1,502210439	4,004258085	0,003687591	0,079574948
DEK	2,350197846	9,115554796	0,003721052	0,079863675
RAB10	1,519569967	8,217301907	0,003727211	0,079863675
UBE2V2	1,573317563	7,307931349	0,003736107	0,079863675
C5orf34	1,962385203	3,887607609	0,003770129	0,080339094
LAPTM5	-1,520584506	8,590745251	0,003851476	0,081551819
IGFBP3	2,720935106	8,2356136	0,003851896	0,081551819
SMG1	1,416075671	7,183008503	0,003862918	0,081551819
NAA50	1,454544206	7,448417773	0,003897679	0,08203171
SFN	2,856399775	7,795560994	0,003979937	0,083505205
RASL12	-1,922066908	5,276251628	0,004031907	0,084336111
DOCK4	-1,371100381	5,689230626	0,004200509	0,087594111
PTK2B	-1,268772175	6,347841826	0,004216804	0,087665813
GABARAP	-1,313538893	8,072599575	0,004281114	0,088732278
DPY19L1	2,313349121	7,431086089	0,004325654	0,089383747
YKT6	1,382430849	7,526366131	0,004341543	0,089441024
RPAP3	1,376199027	6,322283109	0,004357652	0,089502493
PFN2	1,840759555	6,759109329	0,004383366	0,08963506
ZNF503	-1,297283981	5,082614382	0,00439203	0,08963506
FOXF2	-1,581819068	5,246583256	0,004403541	0,08963506
TMEM9B	-1,1496596	5,579871924	0,004422614	0,089755374
ITGAL	-1,400044712	5,7504971	0,004515714	0,091372859
OCIAD2	2,389911783	6,985258807	0,004530017	0,091391083
LYZ	-1,634715127	10,02192051	0,004578345	0,09209361
SPHK1	2,164266465	6,411744507	0,004611195	0,092481575
SRPK2	1,258181485	5,59319132	0,004627727	0,092540961
CYBASC3	-1,132793246	5,566368862	0,004681677	0,093050253
GPR180	1,526606173	5,422949189	0,004682651	0,093050253

SIGIRR	-1,384177555	5,033702466	0,004700201	0,093050253
SLC6A8	3,024948284	6,960149128	0,004721068	0,093050253
NXF1	-1,358593463	6,031491822	0,004721424	0,093050253
RPL36A	1,700683756	10,99980773	0,004830479	0,094925186
S100A2	4,604533293	8,773172682	0,004887875	0,095775914
BRMS1	1,594941092	6,608328045	0,00490707	0,095775914
SHROOM4	-1,660098867	5,145632965	0,004916427	0,095775914
ACP1	1,420463261	6,654608334	0,004929952	0,095775914
COMMD5	1,91223	3,305339204	0,005051023	0,097849221
TIMP3	-1,626194852	8,434414629	0,005088361	0,098135276
ZNF271	1,344739321	5,400118563	0,005094572	0,098135276
MYBL2	2,227118437	5,86451989	0,005257143	0,100725638
IFT74	-1,174815685	5,258282016	0,00525859	0,100725638
EXOC6	-1,069139936	5,399246045	0,005285827	0,100963737
ATP6V0D1	-1,187415917	6,117619441	0,005334588	0,101610491
FAM188B	-1,582851252	5,132092246	0,005379355	0,102177779
PREX1	-1,557076263	4,560660043	0,005402446	0,102331331
MYO1F	-1,197170693	5,642163081	0,005447041	0,102816877
ELOVL6	1,913685131	4,74548987	0,005466275	0,102816877
B4GALT2	1,761571761	7,299092666	0,005473314	0,102816877
CD81	-1,309552586	8,19945864	0,005521434	0,103161413
AHCYL2	-1,610590663	5,771774883	0,005521912	0,103161413
ITGB2	-1,356838806	5,976923523	0,005573953	0,103849142
CENPQ	2,594683558	4,859588359	0,005621239	0,104444763
C7orf23	-1,179507008	4,60244734	0,00573324	0,106006418
PRMT5	1,401841582	7,044744618	0,005736379	0,106006418
SKI	-1,411038852	5,915555181	0,005768669	0,106315017
NPL	-1,277289641	5,04751518	0,005801536	0,106456697
BOP1	2,248140026	7,067144379	0,005820939	0,106456697
MYO1B	-1,142061115	7,163225417	0,005823192	0,106456697
H2AFX	1,901163748	7,403414507	0,005853647	0,10672732
EHD2	-1,381155575	6,757121361	0,005875477	0,106761277
MAP3K5	-1,066058789	5,906148281	0,005886822	0,106761277
SCNN1A	-1,692647302	5,784130503	0,005924645	0,106762883
CISH	-1,394422394	5,155466695	0,005928039	0,106762883
HSPA4L	2,188686905	4,62482484	0,005935863	0,106762883
CYBRD1	-1,523064765	7,787468478	0,005953338	0,106762883
TAF13	1,354760032	6,218334147	0,005965194	0,106762883
CCL2	-1,428815054	8,576236627	0,00610073	0,108594622
EIF5B	1,474688104	7,731483082	0,006113966	0,108594622
MRPL47	1,736522552	7,021727526	0,006115315	0,108594622
SHFM1	1,525366453	9,709798167	0,00615067	0,108880432
NT5C3	1,452377548	5,562727267	0,006185985	0,108880432
IQGAP2	-1,197791986	4,27548927	0,006192582	0,108880432
USP5	2,129728638	9,020462579	0,006195279	0,108880432
SNHG4	1,839126374	6,132167327	0,006252365	0,109601219
ZZEF1	-1,090909867	4,362353186	0,006273286	0,109685985
KIF18A	1,988166683	4,520729012	0,006338477	0,110540835
INPP4B	-1,171150155	2,92911959	0,006354599	0,110540835
SPTAN1	-1,22438216	7,535094621	0,006389549	0,110615143

C11orf54	-1,342073688	4,471752269	0,006391313	0,110615143
TBC1D23	1,504986523	6,658352447	0,006411924	0,110690914
SORD	1,434735661	4,374132638	0,006492603	0,111800659
CCT2	1,494304455	7,461685847	0,006599719	0,113358904
PSMD12	1,215794289	6,209661603	0,006649501	0,113926995
CTHRC1	1,762038005	7,259218601	0,006684569	0,114240784
MRTO4	1,413121226	6,200875694	0,006740134	0,114902427
MARS2	1,69883451	4,06190116	0,006761055	0,11497165
MTDH	1,449247772	8,799791489	0,00678015	0,115009561
GYPC	-1,54220982	6,193526771	0,006890624	0,116593456
USP14	1,216549358	6,85435031	0,006947878	0,11721733
UCK2	1,425820161	6,07708075	0,006961874	0,11721733
COBLL1	-1,132627847	5,423912976	0,006995585	0,117303389
NPM3	1,676502061	5,648568551	0,007024503	0,117303389
C15orf23	1,586955923	4,811685226	0,007028779	0,117303389
MAGOHB	1,46699106	5,557589648	0,007035795	0,117303389
DCLRE1A	2,039006308	4,721203274	0,007089443	0,117909538
CHST3	1,460582416	6,032762517	0,007112723	0,118008892
LOC728554	2,046586388	7,380970171	0,007134364	0,118080655
PHTF1	1,887706949	5,144458176	0,007161674	0,118245655
CYBB	-1,3687273	7,405831922	0,00720767	0,118717633
MTRF1L	1,495443354	5,497321969	0,007270118	0,119337497
NFKBIA	-1,16816417	7,890230531	0,007280305	0,119337497
PDGFC	1,675148033	6,722552189	0,007367816	0,120409463
BTG1	-1,210304592	8,388319179	0,007381017	0,120409463
UQCRHL	1,592899594	5,334337919	0,007398894	0,120413019
FCGRT	-1,196090574	6,331113363	0,007448738	0,120935585
IPO7	1,333452021	7,752888071	0,007471334	0,121014312
UTP6	1,316928823	6,672925971	0,007512055	0,121385549
STAT5A	-1,171436485	4,796088272	0,007573631	0,12173422
CCDC58	1,694515578	5,078013745	0,007584991	0,12173422
ANKRD44	-1,371888415	4,528878262	0,007587189	0,12173422
PRKD2	-1,054531594	6,386587207	0,007635987	0,121989233
KAT2B	-1,260242666	4,869383014	0,007645568	0,121989233
RAB22A	1,425925251	7,913459612	0,007656752	0,121989233
RSPRY1	1,399064934	5,391982934	0,007728988	0,122846181
BMS1	1,373109739	6,429448178	0,007751257	0,122846181
PDSS1	1,345128554	4,149687501	0,007771268	0,122846181
FKBP4	1,351027363	7,294710369	0,0077826	0,122846181
LOC388796	1,259675691	4,918330874	0,007822361	0,123172319
SNX29	-1,195932443	6,117942186	0,007851743	0,123172319
NQO1	2,950101957	7,345705541	0,007857451	0,123172319
TOPBP1	1,88638019	6,935059989	0,007882281	0,123278147
SERINC5	-1,382695442	6,308531196	0,00795185	0,12382462
DOCK11	-1,251881571	4,775547339	0,007953539	0,12382462
ZC3H15	1,4243903	6,82474273	0,008026074	0,124378131
PDIA6	1,231238295	9,102253975	0,008037869	0,124378131
BNIP3	1,746887365	6,896971464	0,008045705	0,124378131
MDK	2,786181511	9,541074296	0,008106246	0,124378131
YPEL3	-1,173416767	7,615722811	0,00810862	0,124378131

ITPKC	-1,186045276	4,664385215	0,008141718	0,124378131
PABPC3	1,833003532	6,782107797	0,008170838	0,124378131
GARS	1,572117376	6,998557171	0,008176749	0,124378131
MRPL33	1,530735758	7,331717974	0,008181741	0,124378131
PFDN2	1,444701632	7,641718772	0,008191272	0,124378131
AHNAK2	2,402944337	6,360180688	0,008198498	0,124378131
TGFBI	1,789659458	8,647317004	0,008207972	0,124378131
EIF4EBP1	2,030025443	7,445321752	0,008251656	0,124762847
C10orf10	-1,880917435	8,056541137	0,008387534	0,126536711
RPL7	1,432579764	11,89077879	0,008434492	0,126623896
GPC1	2,49727765	7,679484558	0,008449386	0,126623896
RANBP1	1,668991448	6,985105467	0,008456212	0,126623896
ZNF259	1,689491844	4,420055978	0,00846759	0,126623896
KPNA4	1,398307446	7,751993617	0,008503677	0,126804203
TP53INP1	-1,224706722	4,370051077	0,008516839	0,126804203
CTNND1	-1,133181742	4,93339993	0,00857643	0,127413238
KIAA1524	1,654536762	3,995778766	0,008694656	0,128888823
E2F3	1,817080256	6,044635245	0,008992227	0,1327531
METTL5	1,468359692	6,695493811	0,008996203	0,1327531
DHRS7B	-1,273674535	4,696170251	0,009034064	0,1327531
APOE	-1,366524864	10,10140034	0,009034851	0,1327531
TSPYL1	-1,018967092	6,845845458	0,009064131	0,1327531
ANAPC2	-1,255795202	3,86731793	0,009072143	0,1327531
ARHGAP17	-0,992176442	7,287693826	0,009104999	0,132948579
PHLDA1	2,030537481	10,67461219	0,009145429	0,133253586
CACYBP	1,73142	7,1697077	0,009166319	0,133273197
FN1	1,89690615	9,035145094	0,009230041	0,133914155
ERGIC2	1,23957739	7,812347561	0,009324516	0,134997611
DVL1	1,982840878	7,296909242	0,009363161	0,13526991
MRPL39	1,497356696	6,802784707	0,009454062	0,136294393
IGFBP7	-1,705817509	8,160747298	0,009486937	0,136340113
PINK1	-1,254310444	4,865492724	0,009497222	0,136340113
ZNF639	1,419996425	7,533314427	0,009579995	0,137239469
CD82	-1,365539133	5,78620682	0,009602142	0,137268354
GCLM	2,368252286	7,420477308	0,009654756	0,137731759
PIP4K2C	1,175329436	5,844220025	0,009696731	0,138041772
LYPLA1	1,605474775	8,946774725	0,009725243	0,138159239
TNFRSF14	-1,179819792	5,759958266	0,00989335	0,140072298
BBS9	-1,050403619	5,29754647	0,00990099	0,140072298
ATIC	1,242723688	7,810164055	0,009978627	0,140878381
DDX5	-1,154932569	8,911652736	0,010032867	0,141351493
UBXN7	1,285840514	6,394263353	0,010221147	0,143707224
TSPAN13	-1,450589067	6,910807297	0,010273254	0,144142638
CBX3	1,653972229	8,059146931	0,010379282	0,145331257
WDR12	1,393944933	5,633251909	0,010458429	0,146139398
RAD1	1,158218553	6,558467202	0,010548803	0,146927489
CTGF	-1,418966672	8,508818745	0,010560773	0,146927489
EDEM1	-1,060112647	6,617253508	0,01059729	0,146927489
CTSD	-1,263505646	10,19776182	0,010601015	0,146927489
AASDHPPT	1,705728323	5,786616216	0,010648513	0,147286431

TUBA1C	1,896695019	11,65166431	0,010706912	0,147794395
CWC15	1,402494857	7,510732233	0,010797847	0,148715723
MEA1	1,371823666	7,7501312	0,010817275	0,148715723
ATP9B	-1,071562397	4,855599273	0,010935499	0,15003857
UGDH	2,057546727	7,23979311	0,011063215	0,151272374
SLC7A1	1,569525009	5,493424188	0,011069792	0,151272374
PSMC2	1,137189073	6,355456868	0,011124856	0,151420744
GMPS	1,528904234	7,387892649	0,011125061	0,151420744
THOC3	1,710903903	6,20906403	0,011191555	0,151947776
PBRM1	-1,10509621	6,18046177	0,011208349	0,151947776
ZEB2	-1,141170735	6,217843993	0,011241181	0,152090505
STXBP1	-1,182528047	5,492393964	0,01127396	0,152231954
DDIT4	1,929366417	9,640423588	0,011324123	0,15260711
NBEAL2	-0,977418513	7,353772971	0,011346802	0,152611125
FBXO5	1,347078265	4,751148824	0,011491095	0,154173084
BTBD10	1,437839561	5,714939465	0,011525425	0,154173084
PSMC6	1,379737851	8,20326046	0,011530763	0,154173084
MTFR1	1,567982658	5,166095792	0,01161539	0,155000676
LOC152217	1,356686187	6,848851152	0,011684193	0,155267359
ALDH9A1	-0,985189765	5,967961743	0,011706201	0,155267359
MED13L	-0,998959872	6,586981737	0,011723757	0,155267359
GPR183	-1,18435027	5,100463147	0,011726454	0,155267359
XBP1	-1,293500957	8,019834587	0,011798017	0,155912168
GNL3	1,325967069	6,139666465	0,011840204	0,156152702
ABCA1	-1,043249428	6,291195455	0,011862018	0,156152702
TSC22D1	-1,083162776	6,96430241	0,012005608	0,157700596
TOMM34	1,359091446	6,04635009	0,012025856	0,157700596
METTL2A	1,243667358	4,738813452	0,012226124	0,159939202
PPID	1,385743215	5,925140995	0,0122497	0,159939202
HMGB3	1,848297128	6,837132738	0,012266931	0,159939202
PPP3R1	1,064491488	7,698997242	0,012378824	0,161090078
IL4I1	1,908408815	4,954221405	0,012539856	0,162874817
TCEB1	1,196470345	7,894323717	0,012573352	0,1629994
CKS2	1,516572791	6,330435672	0,012730959	0,164729426
PION	-1,41048505	6,542435628	0,012840842	0,16554782
KCMF1	1,08500968	6,328204232	0,012842762	0,16554782
ZFP1	1,571782404	5,171053294	0,012947112	0,166578028
EIF3E	1,490153233	9,792187962	0,012987403	0,166723649
GTF2IRD2B	-1,061064754	5,556436728	0,01300733	0,166723649
TYROBP	-1,288992486	9,444969762	0,013048596	0,16693879
HIC2	2,493162202	5,445306548	0,01314189	0,167534727
OGFOD1	1,328178987	5,83632498	0,013144314	0,167534727
PPIF	1,37573966	7,076434007	0,013181596	0,167696458
RPGR	-0,922958025	3,730524326	0,013245722	0,168067364
FAM109B	1,430501511	5,582874831	0,013265215	0,168067364
MAPK6	1,21432088	5,797721577	0,013292258	0,168067364
PPP2R5E	1,304590691	6,914997865	0,013309338	0,168067364
STAT3	-1,063628328	7,420130924	0,013432237	0,16915029
ZNF789	1,677877121	5,575188146	0,013460577	0,16915029
ARHGAP15	-1,492356025	5,306899009	0,013470313	0,16915029

NFIX	-1,25763967	6,232668755	0,013494319	0,16915029
WDR43	1,43538875	6,430511935	0,013547775	0,169212988
C12orf75	1,50243694	7,255596247	0,01354895	0,169212988
TLK2	-0,996080829	7,189180857	0,013597044	0,169356566
CRIM1	-1,340852239	7,741593528	0,013610119	0,169356566
PFDN6	1,588606749	5,377978172	0,013644681	0,169477375
H1FX	-1,095819966	7,124820208	0,013748816	0,170460324
ANKHD1	-0,960670656	5,555172479	0,013933526	0,172349999
SDCBP	-1,242904286	6,374911456	0,013951782	0,172349999
PLEKHM1	-1,019092539	7,244191813	0,013999785	0,172630261
TAF1A	1,458054154	4,937755877	0,014171093	0,174427228
MSH2	1,482221273	6,814291578	0,014245656	0,174541834
MYO19	1,336493593	5,405653814	0,014273281	0,174541834
GOLGA5	1,108101905	5,903884996	0,014281972	0,174541834
IFITM2	-1,201360781	8,118121262	0,014303217	0,174541834
SOX9	1,971912231	4,25599891	0,014308386	0,174541834
HELLS	1,751403171	4,270751933	0,014378465	0,175083486
CD33	-1,266489695	4,280062588	0,014474444	0,175938028
DDX54	1,289210329	6,777735031	0,014586224	0,176902579
NT5E	2,978823446	6,547094946	0,014605683	0,176902579
LMO7	-1,26582414	6,697652745	0,014719616	0,17741049
ST6GAL1	-1,11884538	6,354928083	0,014745121	0,17741049
SLC44A2	-1,236892924	5,40494616	0,01475587	0,17741049
TRMT2A	1,572824983	6,161725275	0,014759477	0,17741049
SGOL2	1,704614427	5,294288352	0,014780865	0,17741049
SVIL	-1,339287353	5,976586525	0,01480372	0,17741049
FAM122A	-1,195475257	6,652483092	0,014891407	0,178011958
DNM1	1,96223901	5,923322154	0,014906119	0,178011958
NFKBIZ	-1,000143808	6,920196608	0,015036026	0,179249408
POLA2	1,395544788	5,492682502	0,015203684	0,180931799
DNAJC2	1,419336551	3,793500224	0,015310321	0,181709694
PTPN6	-1,019188091	6,348894245	0,015325015	0,181709694
ADARB1	-1,297065874	3,976271724	0,015348993	0,181709694
SET	1,263700826	9,671097893	0,015377602	0,181732872
RUNX2	1,684583332	6,246741042	0,015441737	0,181944679
PAK1IP1	1,661060994	6,610033418	0,015448888	0,181944679
HK3	-1,385753352	5,248210338	0,015541306	0,182414877
MRPL36	1,498162405	6,613890824	0,015542315	0,182414877
LDB1	-1,414969425	6,111580428	0,015629243	0,183119949
EVL	-1,161546003	6,76839194	0,015755688	0,184284794
CHUK	1,679523131	5,725937726	0,015994561	0,186643673
SLC33A1	1,104635396	5,725554062	0,016012106	0,186643673
UBA2	1,745431556	8,616058101	0,016059297	0,186874316
PARVG	-1,174699174	4,692762511	0,016276417	0,189078175
FAM3C	2,070319942	8,510204962	0,016308298	0,189126335
NUFIP1	1,264738411	3,556981822	0,016576401	0,191792405
PAWR	1,385553409	5,715905166	0,016617556	0,191792405
XPO5	1,400411217	5,582210221	0,016622571	0,191792405
RABGGTB	1,177705535	7,406036842	0,016662219	0,191925117
TDRD3	-1,107554994	5,482568932	0,01671656	0,19222635

MACF1	-1,071586355	9,10606679	0,016754296	0,19233593
C11orf24	1,150779357	6,792584247	0,016803181	0,192572926
DARS	1,106738091	7,127272619	0,016893269	0,192746672
PSMD14	1,154987199	5,898857438	0,016913616	0,192746672
VMA21	1,249351328	7,170821998	0,016915742	0,192746672
NT5DC3	1,427187959	5,348475051	0,016937898	0,192746672
RMI1	1,267832761	3,641252202	0,016959672	0,192746672
DAPK1	-1,463780313	6,820800993	0,017089005	0,193893388
TARS	1,351063137	6,926350575	0,017173306	0,194526198
NUSAP1	1,587446485	5,405417768	0,017268714	0,19489101
NPM1	1,355811915	10,22488598	0,01731162	0,19489101
ARHGEF6	-1,143714687	4,137112219	0,017316201	0,19489101
SRP72	1,260905587	7,035933424	0,017319835	0,19489101
PMP22	-1,201225063	8,484643585	0,017428004	0,195785108
LDLR	-1,223339023	6,338489163	0,017554373	0,196880377
ZNF107	1,518816291	4,94359848	0,017623817	0,197205162
PAK1	1,295925702	7,491951291	0,017641172	0,197205162
KNTC1	1,731919436	6,96506177	0,017702902	0,197571343
ZDHHC3	-1,013945074	3,961241699	0,017784824	0,19760125
DNAJC4	-1,000104108	5,391109186	0,017790082	0,19760125
PGK1	1,387615157	9,355061994	0,017792516	0,19760125
C3orf14	1,481403029	5,021523745	0,01790725	0,198552099
COL12A1	2,267265643	7,568052217	0,017985648	0,198967195
EGR1	-1,545676991	10,07714598	0,018003044	0,198967195
TK1	1,49365464	6,003269942	0,018090602	0,199436003
TMEM43	-1,010676156	5,707682718	0,018103957	0,199436003
DEDD	-0,883047121	6,360423457	0,018155307	0,199544022
SOX13	-1,107917543	4,789466116	0,018172289	0,199544022
RARS	1,379690342	6,096919338	0,01821077	0,199645079
TXNDC15	-1,033126544	4,191081869	0,018517338	0,202518001
MTBP	2,233130327	4,789472664	0,018532224	0,202518001
HMHA1	-0,997899512	4,966781573	0,018739959	0,204460447
WDR19	-0,961711689	4,565940391	0,018886859	0,205187867
FANCG	1,152784687	5,183465607	0,018888181	0,205187867
SNX25	-1,094780117	6,12703778	0,018908078	0,205187867
NR4A3	-1,472440932	5,073177655	0,018926993	0,205187867
CXCL16	-1,177091027	7,008096668	0,019175735	0,207554501
PWP1	1,188810641	5,922980793	0,019232172	0,207680813
KLF13	-1,018608247	7,466436951	0,019248317	0,207680813
ARAP3	-1,140588749	4,634725787	0,019342132	0,20836335
LRRC37A	-1,048085339	4,288319343	0,019448389	0,209177548
HSPG2	-1,444700612	8,184325604	0,019486435	0,20925669
UBFD1	1,588905497	3,901186051	0,019558951	0,209268074
SEMA4B	1,565009135	6,706284939	0,019560772	0,209268074
HSPA8	1,321953418	10,91462811	0,019587646	0,209268074
SHMT2	1,262708398	6,744186911	0,019610251	0,209268074
FLT3LG	-0,968224338	4,673391611	0,01969754	0,209871131
DOK2	-1,292479424	5,323132715	0,019995629	0,212325198
APBB1IP	-1,172310075	3,745254356	0,020006798	0,212325198
SRXN1	1,869447643	7,828598307	0,020021279	0,212325198

KIAA0430	-1,023711935	4,602040255	0,020100603	0,212789662
NOLC1	1,483111635	7,771657142	0,020128597	0,212789662
CCDC138	1,520507018	4,477910197	0,020158692	0,212789662
HCST	-1,256319989	5,79800641	0,020244635	0,213220892
DHX34	1,817202828	5,328372885	0,020262082	0,213220892
ISCU	-0,87083789	6,935516828	0,02035131	0,213829862
TRMT6	1,136950711	5,175906056	0,020382795	0,213831199
TFPI	-1,453355129	8,105270768	0,020452802	0,213995446
DHCR24	-1,168049227	7,24236271	0,020461216	0,213995446
GPX8	1,887196546	6,662506226	0,020499003	0,21406233
EMILIN2	-1,018737562	5,629063523	0,020622622	0,214579797
ZCCHC17	1,165044685	5,262893216	0,020632381	0,214579797
OSTC	1,194471125	8,054809395	0,020672505	0,214579797
NECAP2	-1,016913827	5,632199437	0,020703285	0,214579797
NUDT21	1,09351796	8,033670629	0,020731083	0,214579797
HDGF	1,067843242	8,619405233	0,020737364	0,214579797
DBN1	1,587672848	7,726867449	0,020825006	0,215160172
PUS1	1,300627518	4,990028197	0,020864242	0,215239438
ZNF394	-0,998303118	3,882359572	0,020905651	0,215340841
NSUN3	-1,117890185	4,378618468	0,021001133	0,215998078
CCDC109B	1,33088757	5,266333755	0,021375883	0,219225457
POT1	1,441957004	4,624658919	0,021379224	0,219225457
TMEM14A	1,274037186	6,746824867	0,021555014	0,220696154
NPAS2	2,017124288	5,441290605	0,021716231	0,221689054
RNF13	-0,873825366	6,129434268	0,02171701	0,221689054
SPRYD3	-1,051364414	5,607561722	0,021760884	0,221804885
MMP11	2,931877352	6,920852177	0,021860999	0,222492767
RUVBL2	1,374474763	5,591475134	0,021898409	0,222541359
MTHFD2	1,327175024	7,298973464	0,021983163	0,222898721
SETD6	1,31453653	4,583577012	0,02199895	0,222898721
RHOD	1,475566569	5,902959468	0,022176637	0,223790482
ARL4C	1,934208746	7,931650971	0,022184687	0,223790482
NDUFB5	0,965112918	8,335603402	0,022185418	0,223790482
TCEA1	1,112316764	7,333024957	0,022264424	0,224175728
BAZ1B	1,317834469	6,15086783	0,02228936	0,224175728
CDH24	1,840314058	5,336308725	0,022376987	0,224725583
DDX6	1,128006351	8,862839178	0,022448057	0,225107794
HSP90AB1	1,366104102	11,49095545	0,0225943	0,226145608
DNAJC21	1,208760869	6,49895226	0,022617877	0,226145608
APBB2	-0,897690064	6,043758951	0,022740206	0,227035819
PMS2CL	1,599450845	4,236564892	0,022857047	0,227868722
DNTTIP1	2,037241429	6,805025609	0,022896637	0,227930168
MTRR	-1,044487093	4,705788446	0,022977475	0,228401465
SLMO2	1,274276682	7,132386985	0,023107438	0,229358979
SNRNP48	1,795197432	6,633424132	0,023192073	0,229864459
ABCC10	-0,959052541	4,619352242	0,023253337	0,230137158
ZFP106	-1,10041621	6,370095752	0,023415611	0,231268292
HLA-DRB1	-1,39386897	10,36759201	0,023444438	0,231268292
SHB	1,271856104	7,188456948	0,023469374	0,231268292
ZNF438	-1,018761572	4,139074628	0,023548329	0,231513015

DPYD	-0,999912021	5,415263388	0,023604084	0,231513015
MBOAT2	1,597734851	6,153182214	0,023613387	0,231513015
LTA4H	-1,140524031	7,892377328	0,023630013	0,231513015
SLC30A9	1,132894515	7,367160831	0,023821802	0,232454887
SENP5	1,480074194	6,497742433	0,023829914	0,232454887
PTPN14	1,328878877	6,050106385	0,023861704	0,232454887
CHST15	-1,244845475	5,329959062	0,023878746	0,232454887
MDM2	1,410570499	7,292877454	0,023910323	0,232454887
ZFAND1	1,187601175	6,766538616	0,023930683	0,232454887
HNRNPAB	1,134178729	7,999666894	0,024030988	0,233097162
PAG1	-1,004275806	5,503247506	0,024228494	0,23427265
ECHDC2	-0,97555434	5,073152385	0,02426504	0,23427265
ZZZ3	1,204347297	6,827805572	0,024313442	0,23427265
NCF2	-1,284562602	5,949322309	0,024314278	0,23427265
DTL	1,460598627	4,578719329	0,024374446	0,23427265
CMAS	1,155205519	6,062782222	0,02438271	0,23427265
ZNF187	1,401052778	3,781227707	0,024392665	0,23427265
TAOK1	0,980353303	7,752785626	0,024463347	0,234621042
DCUN1D1	1,159975551	7,249587748	0,024579352	0,235315921
TSPAN6	1,420423406	6,030284666	0,024604818	0,235315921
HMGA1	1,878211657	4,286469296	0,024701758	0,235912165
HLA-E	-1,007464831	10,01118113	0,024800238	0,236521434
TRANK1	-1,041548331	4,924007252	0,024854562	0,236708457
TMEM48	1,380555928	5,217809547	0,024998346	0,237745777
SLC25A20	-1,006257533	4,072178482	0,025097548	0,238165314
VBP1	1,139932198	7,324296427	0,025112313	0,238165314
VARS	1,087554626	6,645143081	0,025231947	0,238472462
CUL4B	1,139046664	7,50614163	0,025239237	0,238472462
SACS	1,481103021	6,672953509	0,025263835	0,238472462
FRMD6	1,590193145	6,614877902	0,025284586	0,238472462
ATXN2	-1,064824323	5,296643438	0,02545185	0,239718462
SNX7	1,135474361	6,535986323	0,025605641	0,240793157
ZFP91	1,063487791	7,020890865	0,025636579	0,240793157
CD68	-1,285262191	7,336087414	0,025708195	0,241133672
DBF4	1,59175189	5,973381544	0,02577839	0,241459951
SFPQ	0,984914616	6,974581724	0,025842381	0,241727288
VPS54	1,253762655	6,660184616	0,025927778	0,242193864
CYCS	1,267923127	8,181754246	0,026125403	0,243706046
IER5L	1,742463615	7,337724186	0,026286398	0,243821938
MGAT4A	-1,007470111	5,555236473	0,026298685	0,243821938
HCK	-1,163358871	4,759144977	0,026395947	0,243821938
SNHG6	1,283425742	8,319274711	0,026401862	0,243821938
INPP5K	-0,850885604	5,700591717	0,026427683	0,243821938
ARHGAP18	-1,096331736	6,68308005	0,026483956	0,243821938
GLRX2	1,138971786	5,060634846	0,0265221	0,243821938
USP53	-1,25852463	5,450181264	0,026523031	0,243821938
TMEM179B	-0,915954277	5,81097571	0,026534927	0,243821938
LSM1	2,085360356	5,708823132	0,026547664	0,243821938
PTPLAD2	-1,269728252	4,254706525	0,026548264	0,243821938
PTRF	-1,093653703	8,919640617	0,026566901	0,243821938

CCDC112	1,404747638	4,458494256	0,026690162	0,244405381
ACOT13	1,228371701	5,69851097	0,026735065	0,244405381
IRF2BP2	-1,055688806	7,678261885	0,026737999	0,244405381
RAB11B	-0,898895852	7,944993277	0,026787468	0,244529779
CEBPZ	1,403339705	5,168481262	0,026826334	0,24455718
NIN	1,264352056	6,34464157	0,026920211	0,24508534
DNAL4	-0,982766362	4,423479481	0,027098625	0,245901098
RAI14	1,225476319	5,42126516	0,027171832	0,245901098
RABGGTA	-0,877352993	5,182083208	0,027238181	0,245901098
ITGAV	1,256856809	7,920939645	0,02727331	0,245901098
UNC93B1	-1,000835033	5,920801948	0,027287521	0,245901098
ELN	-1,644158387	9,024585411	0,027362766	0,245901098
NCOA1	-0,803473346	5,28765376	0,027526519	0,245901098
SEC23A	1,029974708	7,416597388	0,027552774	0,245901098
KTN1	1,315318149	8,463521255	0,027573001	0,245901098
TACC1	-0,837664461	7,579519959	0,027605126	0,245901098
RAD17	-1,105036213	5,096689782	0,027638151	0,245901098
STRN3	1,232644904	6,819654644	0,027656087	0,245901098
FBXL4	-1,042796236	4,582168801	0,027657387	0,245901098
TTC21B	-1,023410518	4,304474716	0,027750008	0,245901098
PIK3R1	-1,113620425	7,420382412	0,027763735	0,245901098
NOD1	-0,896987572	5,646324411	0,027811579	0,245901098
ABHD13	-1,004059208	3,312335326	0,02782071	0,245901098
TDG	1,064746685	5,429334852	0,027842478	0,245901098
C5orf28	1,244925036	5,146901381	0,027866192	0,245901098
KCTD5	1,359581024	8,054994511	0,027884186	0,245901098
FGD6	1,488018227	6,289043996	0,027919564	0,245901098
CSRP1	-1,272047566	6,940574409	0,027922108	0,245901098
PTS	1,26602552	6,815914617	0,02794363	0,245901098
THBS1	-1,434242733	7,422765139	0,027946403	0,245901098
SLC12A2	-0,877275615	5,217465636	0,027972422	0,245901098
SENP7	-0,926952446	4,751897395	0,027979224	0,245901098
LRRC37B	-0,959863663	3,242230097	0,028025052	0,245901098
TEX264	-0,979759765	4,779866142	0,028025566	0,245901098
RNF170	-0,952542756	3,724338804	0,028055588	0,245901098
WHSC1	1,844219304	6,848430092	0,028187167	0,24673722
ZC3H14	1,000988024	5,391431792	0,028455176	0,248763904
CXCL10	2,304250498	7,74733584	0,028534843	0,249080444
UBE2K	1,03848187	7,274054162	0,028600455	0,249080444
ACP2	-0,82485083	5,436240939	0,028600966	0,249080444
CCDC88A	1,152214818	5,728930358	0,028659381	0,249141681
CXCR4	-1,340222188	6,322976261	0,028681071	0,249141681
PYCARD	-1,076401611	5,719924298	0,028790453	0,249280466
SLC9A3R2	-1,05459824	8,649303331	0,02881069	0,249280466
WDR75	0,981752885	6,46445607	0,028835774	0,249280466
MPHOSPH10	1,040001087	5,735011384	0,028843274	0,249280466
LNX2	-1,021047151	4,388605462	0,0291589	0,25161828
CALU	1,420353345	8,461030183	0,029187573	0,25161828
HADH	-0,922515899	5,404789386	0,029332162	0,252545471
CAPRIN2	1,317885733	5,403022699	0,029387931	0,252706559

TXNDC11	-0,972052838	6,474233436	0,029516411	0,253491697
IRAK1BP1	-0,864412574	3,688003773	0,029692454	0,254594248
BAG2	1,501183782	6,463391356	0,029719463	0,254594248
RBBP8	1,129362999	5,861457345	0,029853332	0,255420161
CHPT1	-1,004275651	6,715386388	0,029968505	0,256084259
TAF15	1,026307579	6,845686374	0,030031857	0,256098341
GNAI3	1,140527731	7,256875769	0,030045267	0,256098341
ARHGEF2	-1,018076908	6,004896589	0,030099471	0,256176527
UHRF1	1,73310612	4,573125438	0,030142159	0,256176527
LONP1	1,586310509	6,116241159	0,030169398	0,256176527
POR	-0,958629189	6,4586378	0,030204711	0,256176527
CGN	-1,379298885	3,46693031	0,030257323	0,25630396
LEPREL2	1,837454254	5,093612889	0,030338568	0,256379462
USP54	-1,067941172	4,933313833	0,030341432	0,256379462
TBCEL	-0,954456274	4,939470587	0,030532649	0,257675904
ZDBF2	1,814147896	4,887897184	0,030634529	0,257775056
MCPH1	-0,828483677	5,714034141	0,030643909	0,257775056
FAM188A	-0,890749454	4,971748246	0,030657805	0,257775056
LRRC16A	-1,194676758	4,534079973	0,030769821	0,258398286
MPEG1	-1,04179798	5,155193471	0,030883009	0,259029813
NOP58	0,947820154	6,770879194	0,030958702	0,259033169
MBNL2	-0,958226392	7,87241066	0,030993387	0,259033169
NEDD8	1,310253615	4,315803112	0,03099737	0,259033169
TXNL4A	1,044581229	6,524775188	0,031047751	0,259136611
FAM160A2	-0,911036146	4,930797687	0,031177082	0,25989795
LRRC42	1,199215482	4,422183985	0,031398326	0,260566641
CDKN2C	1,547817459	5,291780543	0,03142284	0,260566641
OSGEP	-0,958715724	4,848700128	0,031429289	0,260566641
FLNC	1,801115619	6,626688844	0,031444761	0,260566641
RPL26L1	1,10455891	6,695673704	0,031461221	0,260566641
SERINC1	-0,979712126	8,107838337	0,031486569	0,260566641
LRFN4	1,286049316	5,345290802	0,031569621	0,260822993
HLA-DRA	-1,263502964	10,68538977	0,031594045	0,260822993
BTBD9	-1,16452524	6,817390118	0,031666185	0,261102438
KTI12	1,086826936	5,986356145	0,031818798	0,262009392
MRPL21	1,342508898	7,093831861	0,031853026	0,262009392
PTGES3	1,22459634	9,410627436	0,031938725	0,262397794
TRAK2	-0,908226769	5,997635083	0,032127985	0,262706255
DPY30	1,165523621	7,217906138	0,032139916	0,262706255
ATP8B1	-1,175530448	4,356121027	0,032141686	0,262706255
TLN1	-0,927994137	7,49745806	0,032149183	0,262706255
UCHL5	1,466255761	6,469962159	0,03217265	0,262706255
WDR18	1,11145032	5,57113216	0,032207425	0,262706255
CLU	-1,43907952	7,929219034	0,032512009	0,264873819
RGS10	1,853071665	7,387358923	0,03255809	0,264932714
PHB	1,032842456	7,306745141	0,0327056	0,265815839
CNOT7	1,068980936	6,851040787	0,032815271	0,266198074
CSE1L	1,111045247	6,775916481	0,03287215	0,266198074
RPL37	1,311031293	6,721478612	0,032878437	0,266198074
PACRGL	1,738540846	5,021574986	0,032908781	0,266198074

PSMA1	1,391945889	7,613468444	0,033115002	0,266897277
SPAG7	-0,965527754	4,590944651	0,033179072	0,266897277
HSPA4	0,977184489	7,307214925	0,033191996	0,266897277
KPNB1	1,305285515	8,18520209	0,033192665	0,266897277
ABCB6	1,536732804	5,618689262	0,033215795	0,266897277
C1QBP	1,10645635	7,50863898	0,033230061	0,266897277
KPNA5	-0,922001388	4,446801781	0,033303963	0,267110505
C12orf23	0,929094622	6,282760345	0,033342311	0,267110505
SIPA1	-0,98344187	6,13847323	0,033432098	0,267110505
PYGL	1,498129588	6,027308211	0,033449466	0,267110505
FER	-1,01530719	5,126190844	0,033452467	0,267110505
SF3B3	1,029518291	8,097301679	0,033761932	0,269266217
ZFP36L2	-0,968736744	9,371129441	0,033931934	0,269718464
ENTPD6	1,140279784	6,239584686	0,03398549	0,269718464
MFAP3	-0,894191507	4,936801161	0,034017579	0,269718464
ROCK1	-0,994774626	5,046852184	0,034030851	0,269718464
ZBTB4	-0,797577888	6,932611661	0,034056057	0,269718464
ENO1	1,151463548	10,32602794	0,034087787	0,269718464
RPL30	1,296131072	12,11227352	0,034095515	0,269718464
MRPS26	1,373542516	6,744704656	0,034208876	0,270072304
SETX	-0,819291522	6,098454708	0,03425639	0,270072304
KIF1C	-0,939280152	5,907080629	0,034259062	0,270072304
VPS53	-1,135068645	3,779480941	0,034336976	0,270373945
PDE3B	-1,063635767	4,485918754	0,034379153	0,270393823
EIF2AK1	1,139179685	7,734868512	0,034525761	0,271176123
XPO6	1,028740738	6,725698488	0,034561707	0,271176123
SSH2	-0,742989501	5,968155367	0,034597922	0,271176123
SREBF1	-0,996985478	5,424697336	0,034773772	0,272137584
RFC4	1,432873374	6,163021374	0,034829332	0,272137584
JAK1	-0,963522791	6,563549746	0,034840315	0,272137584
MTHFD1	1,096110333	7,211120382	0,034970313	0,272597854
ARHGEF3	-1,019912848	5,034916998	0,034979194	0,272597854
LOC285359	1,254063989	4,440746106	0,035108269	0,273010492
ATG3	1,129779052	6,907710129	0,035112216	0,273010492
NARG2	0,943404417	5,366938673	0,03517351	0,273175589
CPT1A	-0,987955065	5,700872638	0,035305553	0,273889151
COLEC12	-1,238486169	6,638660918	0,035445074	0,274380417
HIST1H2BD	1,191949081	8,957580374	0,035483701	0,274380417
HNRNPR	1,088074271	7,018404524	0,035489592	0,274380417
PARP2	1,856109489	6,028329751	0,035574612	0,274694771
GALNT1	1,042852334	7,379743776	0,035610819	0,274694771
PLEKHA1	-0,753084384	5,476804992	0,035700599	0,274839208
CKAP5	1,177686184	7,373497275	0,035738026	0,274839208
AATF	1,248824236	6,757241096	0,035750459	0,274839208
RHOU	-0,94642126	5,559758971	0,03580252	0,274929489
PIM3	-1,020361694	6,468149262	0,035865492	0,274961626
HK2	1,152955428	6,602948217	0,035887351	0,274961626
ATG5	1,098861513	6,527238438	0,035965312	0,275249677
NAT9	1,409242842	4,979127578	0,036073709	0,275583511
IMPDH1	1,071905511	6,503552193	0,036168902	0,275583511

EIF2S1	1,088425731	5,990943058	0,036172169	0,275583511
ITGB3	1,750314483	5,43057728	0,036227507	0,275583511
NPDC1	-0,971445435	6,48639998	0,03624695	0,275583511
MGST3	-0,860392817	7,533283998	0,036251416	0,275583511
PSMG1	1,330341918	5,717187223	0,036386084	0,276047516
KIAA1429	0,998729702	5,041067471	0,036393617	0,276047516
PLXDC2	-1,147757341	6,804061679	0,0364339	0,276047516
LRPPRC	1,261862988	6,819328471	0,036573448	0,276797269
COL5A2	1,369537451	8,400837949	0,036663802	0,276900318
CNPY2	0,982202971	7,606090748	0,036668278	0,276900318
PIK3CD	-0,903663197	4,354093497	0,036710644	0,276913586
PPT1	-0,864231584	7,02673592	0,036788259	0,277058404
CCNB1IP1	1,094317846	5,995123405	0,03684796	0,277058404
METAP1	0,994034481	5,772087232	0,036851734	0,277058404
PPM1G	1,065167579	6,263218986	0,03691942	0,277261589
TAF9	1,150496938	4,978410084	0,036984587	0,277445432
GCSH	1,339415383	5,455396181	0,037074943	0,277817625
PIK3R5	-0,93126775	5,068525649	0,037124063	0,277880336
BOLA3	1,621862061	6,932258737	0,037199562	0,277921009
MCM7	1,477635868	6,616608944	0,037211011	0,277921009
RNF6	1,160494857	6,381219741	0,037277659	0,278114174
ZNF431	-0,835762648	5,935331066	0,037465101	0,279207128
STX16	0,997362826	7,146513084	0,037555995	0,279457923
CNIH4	0,942005551	7,246657706	0,037580718	0,279457923
XPOT	1,041611995	6,390462892	0,037626251	0,279491727
BRIX1	1,038835013	6,235764118	0,037800047	0,280418091
KBTBD2	1,280883991	7,415077735	0,037833208	0,280418091
CPEB4	-0,928182556	5,723827093	0,037921522	0,280767491
HEG1	-0,942642553	6,580344379	0,038253991	0,282921873
SUZ12	1,302867993	6,921756165	0,038349597	0,283321672
RPL26	1,267644601	11,77964001	0,038420696	0,283365493
KHDRBS1	0,993492416	8,570490677	0,038438639	0,283365493
IGFBP2	2,336949589	8,026190333	0,038688628	0,284586638
C12orf45	1,198777836	5,243361263	0,038735807	0,284586638
USP48	-0,85110666	5,490565869	0,038742096	0,284586638
STEAP1	1,389050089	5,418816732	0,038800536	0,284586638
SMAD1	-1,203252834	5,20681046	0,038915794	0,284586638
FTSJ3	0,997231832	4,57711752	0,038922993	0,284586638
EIF4G1	1,209077567	9,320670394	0,038926287	0,284586638
GATSL3	-0,897522273	3,405644901	0,038938163	0,284586638
ILF2	0,959669447	8,110897308	0,03907846	0,285306227
DCN	-1,431116789	10,04571084	0,039288736	0,286534644
LPAR6	-1,113513264	4,927459577	0,039331957	0,286543392
OAF	1,857996552	5,791451133	0,039725327	0,289014483
PRIM1	1,193187123	4,570728675	0,039760012	0,289014483
HSDL2	-0,938046345	5,408763727	0,039798299	0,289014483
STRAP	1,306910386	7,574851313	0,039901276	0,289290417
TBCA	1,148889342	8,963302388	0,039921144	0,289290417
BUB3	1,058466542	7,058797691	0,040036501	0,289409949
OTUD6B	1,191283114	4,931602834	0,0400574	0,289409949

SBNO1	1,029209232	7,023689144	0,040064964	0,289409949
FBXO22	0,947300286	4,733178278	0,040206037	0,290121658
PLA2G16	-1,046974096	6,1136483	0,040364585	0,290695057
NHP2	1,009324749	5,446698769	0,040370761	0,290695057
C11orf75	-0,922131973	3,992916365	0,040494483	0,291015029
PDCD6	1,096463432	7,466581365	0,040500552	0,291015029
INPP5A	-0,995055168	5,884073901	0,040640575	0,291701838
PAN3	-0,845334445	4,737819496	0,040681691	0,291701838
PLXNA1	1,227458309	6,448973907	0,040819619	0,292383386
SH2D2A	1,578394178	4,578612618	0,040943923	0,292966014
NR2C2AP	1,120874221	4,373719918	0,041354889	0,29559642
NOP56	1,364483461	7,87068503	0,041424243	0,295782104
ZBTB1	-0,75177135	5,650069756	0,041630942	0,296947065
PLOD1	1,230210695	8,928724648	0,041687459	0,297019184
FAM43A	-1,310657706	6,810406035	0,041766808	0,297019184
NKTR	-1,047113467	4,473977998	0,041771726	0,297019184
GAS7	-1,135230985	6,785180821	0,041867248	0,297388299
HPS3	1,229452725	5,23804182	0,042124235	0,29890235
SLCO2B1	-1,01490358	8,16935929	0,042269112	0,299618583
C8orf4	-1,559039695	8,164034882	0,042364033	0,299979582
SORT1	-1,172765935	6,708663699	0,042439678	0,300203488
TMEM167A	0,930362798	7,965635563	0,042563615	0,300444501
DDX1	0,984236121	6,158750176	0,042601833	0,300444501
GGA2	-0,778308107	6,309559246	0,04266809	0,300444501
DCAF8	-0,757090963	5,604293976	0,042725359	0,300444501
TMEM66	-0,852333839	8,847495042	0,042763113	0,300444501
BET1L	-0,940776811	3,67519304	0,042797134	0,300444501
NIPAL3	-0,740031696	4,852542063	0,042822173	0,300444501
MTERFD1	1,230208768	4,286448386	0,042856539	0,300444501
TNFAIP2	-0,962391353	7,439839532	0,042870289	0,300444501
ARRDC2	-0,862353005	6,331725192	0,042982672	0,300922832
SERBP1	1,131147745	8,568486497	0,04310929	0,301499741
RGS2	-1,166975932	5,546540069	0,043207523	0,301877153
ZFAND6	-0,84355743	5,756963087	0,043344299	0,302380768
MINPP1	1,076142014	6,627257158	0,043388889	0,302380768
NOP14	1,14016367	6,66713307	0,043412637	0,302380768
METTL1	1,181805586	4,578308806	0,043526844	0,302796859
CCDC115	-0,870783317	5,250104257	0,043561185	0,302796859
KLHDC7B	1,859241874	4,881305788	0,043655359	0,302804783
RPF2	1,45766656	5,884532456	0,043658928	0,302804783
POC1A	1,085876491	3,831048521	0,043715309	0,302804783
C15orf38	-0,917099281	4,503275374	0,043739949	0,302804783
HSPA1B	1,316291677	8,849311813	0,043904264	0,303634056
ZFP112	1,591762961	4,614070094	0,043975692	0,303640956
DOCK6	-1,005386328	6,476230881	0,043994319	0,303640956
AAGAB	0,857891649	5,905783193	0,04429979	0,305440107
LGALS9	-1,036002901	4,456302603	0,044546836	0,306735161
SPATS2L	1,601752906	6,902277198	0,044626809	0,306735161
TMEM170A	1,13318606	5,209490213	0,044658498	0,306735161
TMBIM1	-0,991150585	7,460313371	0,044667549	0,306735161

CNP	0,922040855	6,567138315	0,044853367	0,307701317
NF2	1,179212113	7,262021981	0,045051801	0,308596373
ZNF461	1,312942087	3,438594834	0,045083651	0,308596373
NUDCD1	1,177769828	5,383748187	0,045119605	0,308596373
CCDC86	1,488800769	5,452475968	0,045232794	0,308815119
RNPEPL1	-0,960583547	6,848368338	0,045242162	0,308815119
TRPM2	1,379734716	4,177115368	0,045524459	0,310431283
RHBDD2	-1,013737267	6,215686733	0,045610528	0,31070748
RALGAPA1	-0,937783991	6,122118316	0,045776228	0,311525046
DYRK3	-0,953738694	4,399677222	0,045891936	0,31200111
PDCL3	1,182555308	4,212238362	0,046004063	0,312352391
DNA2	1,443575351	5,069661061	0,046035218	0,312352391
NONO	1,247410402	3,419500223	0,046225314	0,313330431
TUBA1A	-1,046067408	8,743758135	0,046429981	0,313867491
CRYL1	-0,815121071	5,08404569	0,046431278	0,313867491
RPL23	1,204920918	12,21476485	0,046442631	0,313867491
TTYH3	1,617973155	7,175225773	0,046679804	0,314812996
DAGLB	-0,872929478	4,098396958	0,046718707	0,314812996
CLIC6	-1,258810965	4,514938452	0,046721037	0,314812996
DOCK2	-0,941865123	4,375527812	0,046864487	0,31529636
EIF5A2	1,142969954	4,312085159	0,046905182	0,31529636
AP1G1	-0,739797339	6,498792634	0,046931486	0,31529636
CCDC22	-0,82753996	4,256511971	0,047189229	0,315865096
SAP30	1,05646179	5,718062064	0,04721715	0,315865096
CCDC97	-0,972884973	4,934708881	0,04722626	0,315865096
FAM118A	1,35368933	5,442024261	0,047231408	0,315865096
NELF	1,123304606	6,529890039	0,047247749	0,315865096
IRAK1	1,378577633	4,963201877	0,047309192	0,315966093
RAB23	1,221481285	6,584463288	0,047440806	0,316535086
INTS10	-0,990162875	5,678638002	0,047565381	0,317056046
BZW1	1,07768736	8,524982814	0,047801402	0,317547145
PCNA	1,061915318	6,874125921	0,04783521	0,317547145
MAP1B	1,571880114	7,917113426	0,047860204	0,317547145
ZNF710	-0,833333791	4,680239477	0,047884418	0,317547145
TCEB3	-0,830768386	5,263757408	0,048078029	0,317547145
CDK9	-0,829980488	4,8751267	0,048125525	0,317547145
RBL1	1,243776784	4,795055305	0,048134673	0,317547145
KRT8	1,304732026	7,671041098	0,048176834	0,317547145
TMCO3	1,346921597	7,117079169	0,048203206	0,317547145
GTF3A	0,934776647	8,124108886	0,048215163	0,317547145
GPR89C	1,093641068	3,740492479	0,048250272	0,317547145
UTP18	1,025987097	5,824824178	0,048270378	0,317547145
MRPL24	1,076743991	6,756783316	0,048274112	0,317547145
RPL23A	0,931240046	5,925443254	0,04830162	0,317547145
ZCCHC7	0,889891515	6,686258845	0,048344491	0,317547145
CMIP	-0,849419458	6,321676977	0,048384145	0,317547145
SEPT11	-0,716080161	6,433818954	0,048504626	0,318022655
DCTPP1	0,997858766	5,947048781	0,04858265	0,318022655
HDDC2	0,951895444	6,799055025	0,048596511	0,318022655
B3GALT4	-0,837259944	3,620631998	0,048751247	0,318729389

DFFA	1,229840749	4,460321576	0,04893374	0,319259132
ACADVL	-0,887884991	4,417827367	0,048954166	0,319259132
VWA5A	-1,239921135	6,081161583	0,048989495	0,319259132
SEC61G	2,19061082	9,66782468	0,04901955	0,319259132
ARHGEF10	-0,964978798	5,573740597	0,049077288	0,319330181
SPATA20	1,072186096	4,766320185	0,049247562	0,319978827
FAR1	1,11219175	5,828926581	0,049349783	0,319978827
GSTA4	-1,118631405	5,942675734	0,049448273	0,319978827
HERC2P2	-0,91851741	5,932579638	0,049483714	0,319978827
EPHX1	-1,12584586	6,771007807	0,049502698	0,319978827
SUB1	1,29398501	9,441266323	0,049546132	0,319978827
INTS1	1,684443976	7,371098393	0,049582289	0,319978827
FAM105A	-0,97697024	4,520558667	0,04958974	0,319978827
CHKB	-0,981707449	5,058148723	0,049599299	0,319978827
ADAM10	-0,686646997	6,791571974	0,049787903	0,320836228
FCHSD2	-0,82110519	2,412089858	0,049826304	0,320836228
GNB5	0,964166835	4,904249679	0,049937876	0,320968137
C1QB	-1,122139669	9,349266583	0,049954942	0,320968137
SYNGR2	-0,908542754	7,318169288	0,05001965	0,320968137
RASSF5	-0,996142645	4,745089762	0,050035068	0,320968137
C3orf38	-0,767291515	4,778495923	0,050148034	0,321390457
ARL17A	1,435423086	4,759087203	0,050219437	0,321545862
GOLPH3	0,984930707	7,718465041	0,050271179	0,321575208
MAP3K3	-1,06204859	4,395871186	0,050472998	0,322141341
FKBP3	1,220094039	6,033561436	0,050491012	0,322141341
PLXND1	-0,974395384	6,952535152	0,05050256	0,322141341
MEIS3P1	1,640462306	6,921553589	0,050548649	0,322141341
HRSP12	1,531456832	5,484893761	0,050661262	0,32255756
ARAP1	-0,77845049	7,25507785	0,050713297	0,322587658
KDM5C	-0,834632029	6,485317808	0,050874747	0,32331305
TMEM175	-0,966187685	4,773786677	0,051040927	0,324067117
FADD	1,141295342	5,63928835	0,051180866	0,324653327
SBF2	-0,778422052	6,468314241	0,051340104	0,325360754
CCNL1	-0,958961074	4,936553776	0,051416206	0,325540488
FBL	1,264982042	8,269404495	0,051552862	0,325574843
SRPK1	1,225366219	6,228253851	0,051617597	0,325574843
AGXT2L2	-0,90434018	5,647847896	0,051652951	0,325574843
FBXO11	-0,781172965	6,538065042	0,051704043	0,325574843
KIAA0922	-0,845527703	5,649802196	0,051738101	0,325574843
PTAR1	-0,943138959	4,463676159	0,051774606	0,325574843
CEP78	1,107601873	4,745709296	0,051816906	0,325574843
NOB1	0,928446359	6,001283633	0,05185966	0,325574843
CST3	-0,866138012	10,13115775	0,051881387	0,325574843
TUBB2A	1,387191282	6,064272664	0,051899084	0,325574843
CCNC	1,150804934	6,983955264	0,052011164	0,325685536
ARHGAP5	1,089244647	7,017904349	0,052012252	0,325685536
PTPLAD1	1,083619971	5,849906934	0,052131556	0,326133103
ETFDH	-0,832832972	3,990354337	0,052209775	0,326323057
TTC27	0,961276803	3,224112617	0,052299677	0,326450143
TMEM204	-1,101860621	6,53159276	0,052325855	0,326450143

NIPSNAP1	1,360665878	5,621790679	0,052460083	0,3269884
NDUFB9	1,1055826	8,452977989	0,052570831	0,3272823
SPATS2	1,139584314	4,451795009	0,052603226	0,3272823
CABLES1	-0,992636496	4,626303374	0,052655847	0,32731105
TOP1	1,152223589	7,021089824	0,052718799	0,32740391
ZHX2	-0,937739015	5,917742689	0,052934583	0,328444876
INSR	-0,861903369	5,847057732	0,053034279	0,328552569
TYMS	1,344520343	7,004839345	0,053048303	0,328552569
MSL3	1,040072752	5,742186609	0,053130564	0,328763444
PLCB2	-0,95360342	5,886890001	0,053240498	0,329145021
NDRG1	1,416389026	9,619786655	0,053433885	0,329534887
MED17	1,178618034	5,683466584	0,053505607	0,329534887
PRELP	-1,577395468	7,790128384	0,053517494	0,329534887
ZNF669	1,331912715	4,525421298	0,053537841	0,329534887
RCC1	1,190364196	4,512239173	0,053545191	0,329534887
FANCE	1,532068663	5,873418753	0,053645899	0,329856976
DDAH1	-1,087681669	6,070195038	0,053778897	0,330318686
STON2	-1,046350748	4,692197431	0,053817871	0,330318686
RUSC1	0,957438246	6,452097002	0,054118052	0,331801454
ANKRD50	-0,882211929	6,320844141	0,05415677	0,331801454
SNRPG	0,941616215	7,521687585	0,054358068	0,332461098
ABCE1	1,814709953	7,91598525	0,054361948	0,332461098
N4BP1	-0,827912783	4,101045314	0,054611244	0,333426857
CCNDBP1	-0,942138177	5,488968424	0,054617656	0,333426857
SEC11C	-0,892432823	6,773863273	0,054689121	0,333564502
FAM100A	-0,759864837	6,042832671	0,054784755	0,333725414
GOLIM4	0,956264854	6,974222613	0,054818874	0,333725414
KIAA1919	-0,875458579	4,187767573	0,054862324	0,333725414
TM9SF3	-0,848369491	6,603524888	0,054933738	0,333861997
RSRC1	1,358977734	5,384482005	0,055008364	0,334017842
NET1	1,084261457	6,170324347	0,055086677	0,334195772
SYNE2	-0,930036813	5,463288741	0,055148994	0,334221146
DCUN1D3	-0,771339619	5,642315613	0,055188885	0,334221146
UAP1	1,137835176	6,700774415	0,055277282	0,334459439
EFNB2	1,125006516	5,938490764	0,055349915	0,334602011
GFPT1	0,882508564	6,189991413	0,055435885	0,33482489
ZC3H8	1,075318773	3,818135737	0,055551328	0,335191953
EIF4A3	0,875932387	6,537725208	0,05559497	0,335191953
COPS3	0,978182874	6,599160318	0,056220371	0,338663173
ENY2	1,427246744	7,760759421	0,056594477	0,340433914
RUVBL1	1,024303818	5,478743818	0,056642365	0,340433914
NIF3L1	0,956392273	4,541486924	0,056664099	0,340433914
PSMB7	0,955233168	7,532811351	0,056820299	0,341071846
CHCHD2	1,145524646	10,03414754	0,056954211	0,341376947
MPHOSPH6	1,148023431	5,211737136	0,056971252	0,341376947
IARS	1,066186291	7,944228843	0,057169228	0,34216277
NFE2L3	1,679762822	4,648285569	0,057288057	0,34216277
NUCB1	-0,820535737	8,42404272	0,057323781	0,34216277
RNF169	-0,765053418	5,064246635	0,057335304	0,34216277
CCT3	0,982852102	7,474759008	0,057353284	0,34216277

B4GALT4	1,086666663	5,065391187	0,05750139	0,34262747
HIST4H4	1,292229945	3,414462078	0,057531669	0,34262747
ACOT7	1,287325057	5,063686464	0,057601486	0,342707446
MLH3	-0,851910103	4,52884305	0,057650435	0,342707446
PSMD2	1,07974846	7,536336814	0,057712449	0,342707446
SLC23A2	-0,991974948	5,094052915	0,057748255	0,342707446
NUCKS1	0,954248119	9,443789052	0,057796387	0,342707446
LRRC59	1,089606977	7,874651889	0,057859734	0,342784993
B2M	-1,061340283	11,37062321	0,058325085	0,344569203
BCL6	-0,943532341	6,801975248	0,058358589	0,344569203
CDK6	1,195327771	7,267733862	0,05836431	0,344569203
KPTN	0,967875919	3,076913162	0,058373313	0,344569203
CITED4	-1,118860624	4,60137489	0,05841355	0,344569203
PTPRM	-1,053240034	5,407678094	0,058550596	0,345079096
HIF1A	1,234071037	8,005287671	0,058634308	0,345274045
SRRM2	-0,783880066	9,110538434	0,058693163	0,345322413
DCAF11	-0,697892715	4,931353272	0,05909584	0,347391838
HOXA5	-1,374270419	5,375585156	0,059227522	0,347866043
KIAA0907	1,525842152	6,263521259	0,059369263	0,348209734
TMED10	-0,795856694	7,683492145	0,059388168	0,348209734
CD200	-0,893046522	5,025460547	0,059484043	0,348472243
C5orf51	1,093446231	5,714178905	0,059589997	0,348543745
PSME3	0,942279786	6,522340447	0,059598476	0,348543745
MARCH8	-0,816065951	4,002045345	0,059767365	0,349061682
DAP3	1,077927542	7,076162768	0,059789419	0,349061682
BCAT1	1,057310051	6,785473965	0,059976432	0,349752143
DDX17	-0,691894436	8,602119993	0,06003848	0,349752143
PHTF2	1,101921552	5,190715023	0,060061557	0,349752143
CRISPLD2	-1,148285818	6,60926426	0,06040511	0,351452597
TROVE2	-0,89036668	6,119099984	0,060495444	0,351678121
LAMB2	-0,920375354	6,83730251	0,060594729	0,351955242
C14orf166	1,09307081	9,00380405	0,060714767	0,352352338
ADRM1	0,963304742	7,59678118	0,060826819	0,352407328
IRS2	-0,918926169	4,881747951	0,060827603	0,352407328
DNAJB11	1,025355126	8,137959677	0,060932373	0,352714643
FAM8A1	-0,865165118	3,984370989	0,061052812	0,353112066
VPS13D	-0,806285226	5,195126809	0,061122727	0,353139363
UNG	0,968549168	4,717219233	0,061161107	0,353139363
TEX10	0,965486213	4,962643299	0,061326918	0,353505269
NCOA7	-0,751960323	4,664780865	0,061328162	0,353505269
MCM6	0,939781487	5,534463897	0,061524692	0,354338575
SYNJ2	1,06982406	5,06320381	0,061642471	0,354717306
LLGL1	1,330085072	5,501497611	0,061835567	0,355528444
ITGB1	1,099162557	10,3390347	0,061982484	0,35607292
ABI3	-1,008625211	6,174851573	0,062071403	0,356078745
TXNIP	-1,038733044	9,241306648	0,062087935	0,356078745
TRIB3	1,682948511	6,219061337	0,062195458	0,356395651
RPS9	-0,804975629	8,476653247	0,062298987	0,356689164
JMJD1C	-0,843173044	4,788370108	0,062370728	0,35680033
TGFB2	1,428190934	6,163028855	0,062578799	0,357361782

VAMP8	-0,910919381	7,042647977	0,062591221	0,357361782
PSAP	-0,850638126	10,70575692	0,062626093	0,357361782
ODC1	1,224636923	8,148607604	0,062690151	0,35742821
MMP14	1,171845721	9,475808758	0,062847407	0,358025454
KLF11	-0,884822831	4,75742809	0,06290294	0,358042695
CYP7B1	-0,950009173	4,082948104	0,063061672	0,358148566
PSPC1	1,235121719	4,736316998	0,063062977	0,358148566
ZNF551	1,19036569	2,537928338	0,0631428	0,358148566
SLBP	1,080975353	6,897849696	0,063146314	0,358148566
ZNF772	1,401515332	4,036853376	0,063234712	0,358148566
CLNS1A	1,313177447	7,753810846	0,063236673	0,358148566
GCA	-0,943853892	4,360634818	0,063369706	0,358570276
ALDH18A1	1,172092617	6,280077547	0,0634325	0,358570276
CLK1	-0,757641605	6,791088392	0,063468884	0,358570276
VDAC1	0,932223821	7,879447105	0,063617851	0,358945978
IGF2BP2	1,508768685	5,450802034	0,063640664	0,358945978
GUF1	1,213531964	4,836699023	0,063726319	0,35913204
PPA1	1,176527427	8,202848876	0,063890236	0,359758479
CUL7	1,353085572	5,903174653	0,064348563	0,361940862
POLA1	1,025763284	5,535562488	0,064408981	0,361940862
TNC	1,737707896	9,372408348	0,064449221	0,361940862
RPS28	-0,952419722	10,70075889	0,064490123	0,361940862
MRPS16	1,005237647	7,039913173	0,064856635	0,363527584
POSTN	2,113144292	8,538642494	0,064879465	0,363527584
ERH	0,837699777	8,586149401	0,065078212	0,364137982
SLCO2A1	-1,142203733	6,849108493	0,065140356	0,364137982
CCDC90A	1,079630744	7,055875347	0,065187644	0,364137982
CCDC51	1,086134063	3,166057835	0,065202006	0,364137982
CNOT1	0,919756043	6,957231378	0,065293175	0,364348739
MRPL12	1,057504977	5,736488618	0,065440041	0,364768297
GPI	1,140725018	9,903279866	0,065497039	0,364768297
UBE2M	0,941279685	7,254436234	0,06552884	0,364768297
THOC2	0,922618592	6,181053233	0,065604282	0,364786324
ZC3HC1	0,990647556	4,826507651	0,06563907	0,364786324
AIDA	1,240105014	8,472606505	0,065811035	0,365444177
MGC72080	0,872856756	4,537195205	0,065885099	0,365557763
DHX33	1,129735544	4,726476572	0,066058087	0,366219589
SMN1	0,872872445	6,421131173	0,066199569	0,366416879
RNF7	1,02533325	7,815904453	0,066201143	0,366416879
ANKH	1,431712425	5,696938002	0,06652324	0,36790103
LMBRD2	1,04295885	4,247601022	0,066672662	0,367915307
HSPA9	0,845579572	7,915691076	0,066684303	0,367915307
RPN1	0,839037115	9,195572522	0,066693597	0,367915307
RPL13	1,46972925	10,63033205	0,066741639	0,367915307
C10orf76	-0,708738902	5,301320057	0,066833906	0,368126337
CPOX	0,926760298	3,992517333	0,066984412	0,368657553
SNHG5	1,268276515	8,438963234	0,067178329	0,369208045
PITRM1	1,358489536	6,105435768	0,067192724	0,369208045
E2F5	1,603465669	5,138525315	0,067436089	0,370203754
ARHGEF35	1,325347851	3,618727708	0,067482514	0,370203754

C7orf29	1,807510551	4,579174008	0,067750128	0,370897915
DAG1	-0,726695629	6,534587899	0,067761221	0,370897915
SASS6	1,709899279	4,535666755	0,067800729	0,370897915
FAM115A	-0,799335944	4,863074596	0,067826617	0,370897915
NASP	1,164479974	7,338529618	0,067953101	0,371151747
LTBP4	-1,5028795	8,465789233	0,067981893	0,371151747
TMEM50B	-0,764567923	4,315716232	0,068637787	0,374135497
MICAL1	-0,850075285	7,181814042	0,068638144	0,374135497
NUP54	0,826483089	6,533765957	0,068796819	0,374628629
PA2G4P4	-0,835676268	2,261376469	0,068838491	0,374628629
RBM12	1,374877	3,230703968	0,068995875	0,375155548
PTP4A2	1,051536387	7,638505901	0,069053775	0,375155548
VPS33A	1,005808528	4,898252023	0,069100362	0,375155548
PDCD4	-0,809130151	6,365606027	0,069181684	0,375298253
RAP2C	1,037202408	5,134525695	0,069318139	0,375739578
NDUFAF2	0,962692414	5,899352991	0,069955138	0,378891254
LGALS3	-0,997791177	8,012393485	0,070065955	0,378970741
TBC1D13	-0,800885473	5,096491981	0,070080966	0,378970741
DRAM1	-0,958219905	6,582738968	0,070458791	0,380557768
AHR	-0,94443929	7,437551962	0,070486063	0,380557768
EXOSC2	1,00451775	6,296104411	0,07060537	0,380900331
RBM34	0,947296368	5,800951883	0,070783009	0,38155679
GTF2E2	0,840919123	5,125531681	0,070961756	0,382218179
UQCRB	1,019049015	8,078175882	0,071206497	0,382801682
RNF10	-0,759283545	5,456471371	0,071218739	0,382801682
TSPAN3	-0,74501989	7,88087961	0,0712385	0,382801682
CYC1	1,031036282	7,95140957	0,071305634	0,382860726
KLHL21	1,095058803	5,104586285	0,071547732	0,383192199
TMEM106C	1,034267287	6,682718302	0,071577672	0,383192199
QARS	-0,780373268	6,799344612	0,07161642	0,383192199
NEAT1	-0,986017161	11,30966782	0,071644347	0,383192199
TSC22D3	-0,93971237	7,87044281	0,071648343	0,383192199
PLXNB1	-1,16168353	5,98596588	0,071837096	0,383806927
CASP3	1,116703693	6,073558059	0,071892248	0,383806927
EID3	1,556358879	5,157509312	0,071932139	0,383806927
SSFA2	1,061904986	7,966331575	0,072005921	0,383900214
PRKCI	0,739007199	6,570251173	0,072151645	0,384226597
CDKN2AIPNL	1,16254901	6,009120218	0,072228916	0,384226597
SNW1	0,940633848	7,210564696	0,072236178	0,384226597
RBL2	-0,874336286	6,468431293	0,072398406	0,384382988
EIF2S3	1,001976779	9,099022501	0,072429224	0,384382988
SDAD1	1,017690473	5,923841671	0,072458793	0,384382988
RHBDD3	1,417677618	4,968753705	0,072491058	0,384382988
MDC1	1,196029164	6,265200768	0,072567905	0,384491488
ATG2B	-0,744644167	5,491164263	0,072749793	0,385053734
HAUS5	1,731686589	5,621225449	0,072842872	0,385053734
RRAGC	0,920468257	6,429423839	0,072843425	0,385053734
NIP7	0,948290195	5,045895292	0,073003993	0,385603583
SCAMP2	-0,805763943	6,653667481	0,073088431	0,385750782
SIRPA	-0,754515663	6,136996169	0,073256262	0,386108842

RAD23A	1,202041561	10,07064514	0,073269518	0,386108842
HMGXB4	1,022325756	6,169941101	0,073464086	0,386835214
TMEM109	-0,775297126	6,295629129	0,073949724	0,388657863
TWISTNB	0,912872934	4,962709317	0,073949988	0,388657863
PPP2R5A	-0,883108752	4,867644817	0,073981215	0,388657863
PCIF1	-0,720038299	6,383296135	0,074106918	0,389018535
GTF3C6	1,089269666	6,388803263	0,074324575	0,389379233
CHTF18	1,661417356	4,521778436	0,074431124	0,389379233
DKK3	-0,881923501	6,716571383	0,074440249	0,389379233
CDC5L	0,908579333	6,43306267	0,074493817	0,389379233
ARL1	0,764253976	8,141853191	0,074536348	0,389379233
LATS2	-0,967622555	4,80431773	0,074577245	0,389379233
MKL2	-0,777586602	5,878743164	0,074616681	0,389379233
LDHB	1,132070486	8,737829249	0,074632447	0,389379233
GMPR	-0,869372727	3,761777547	0,074829255	0,389770141
SMARCC1	1,005143056	6,888583774	0,074874601	0,389770141
DHX37	1,060884788	5,160955726	0,074878851	0,389770141
SPIN4	1,142192756	5,920782936	0,075075686	0,390496644
C9orf116	-1,02964321	4,029387063	0,075216823	0,390932559
TCF4	-0,921802527	5,297904047	0,075302003	0,391077198
CCL5	-1,078120448	7,068537777	0,075370158	0,391133263
RALGDS	-0,899075233	5,796665014	0,075428966	0,391140775
OXCT1	1,305566656	5,870350061	0,075627545	0,391700122
CARD8	-0,913538643	5,451272735	0,075676241	0,391700122
RNF115	0,87275071	6,388996108	0,07570916	0,391700122
PUS7L	1,025980606	5,173183717	0,07578754	0,391734156
HN1L	1,069124016	7,034237763	0,075886915	0,391734156
UBTD1	-0,764447832	5,271168324	0,075900236	0,391734156
SSR1	1,103400108	9,018665004	0,076017852	0,391734156
INTS4	0,913289991	5,876650695	0,07606318	0,391734156
IMP4	1,058839026	6,193464205	0,076140151	0,391734156
ZC3HAV1L	1,128511284	4,798303722	0,076182576	0,391734156
PFKFB2	-0,782410239	4,775993728	0,076217651	0,391734156
PEA15	-0,86442664	6,763732162	0,076232765	0,391734156
MTMR10	-0,743366764	6,236889928	0,076316577	0,391851103
ZNF302	1,186921674	5,229386518	0,076420991	0,391851103
PRKAG1	1,125757675	5,132189098	0,076478549	0,391851103
EEF1E1	1,267737376	6,247383814	0,076507005	0,391851103
FAT1	1,286534722	6,521521735	0,076560387	0,391851103
PRKAR1A	-0,733486264	8,653135374	0,076600311	0,391851103
TMED5	-0,904276416	6,693712828	0,076792581	0,392357541
STYXL1	0,917723411	6,145841029	0,076814389	0,392357541
GLTSCR2	-0,673554301	8,855694517	0,076882869	0,392413385
SAT2	-0,837815134	5,404814915	0,077015869	0,392798214
STIP1	0,959566087	7,705081165	0,077086171	0,392855998
ST13	0,914942903	8,783090026	0,077148318	0,392855998
CKS1B	0,91566179	6,329801333	0,077200035	0,392855998
AIMP2	0,935666784	5,583264486	0,077279312	0,392966165
ERCC6	-0,829016715	3,576960831	0,077413023	0,393352758
SCARB2	-0,703906766	7,858719315	0,077660673	0,394317299

TIGD1	1,001562089	4,301241492	0,077811254	0,394609676
FMR1	0,964361938	5,32889585	0,077931736	0,394609676
CDKN1B	-0,71696435	5,685893064	0,077962887	0,394609676
FAM60A	1,003902169	6,46711947	0,077969233	0,394609676
TMCO7	-0,791513547	4,508651001	0,078022549	0,394609676
BAIAP2L1	1,253612335	5,831284635	0,078065472	0,394609676
FYCO1	-0,758573364	6,079708732	0,078161934	0,394804614
SNAP47	1,025565838	4,6122401	0,078237293	0,394892749
MTX2	0,926675257	6,25664325	0,078370691	0,394989135
TINF2	-0,698479673	3,570006912	0,078375729	0,394989135
VEGFC	1,621095587	6,392281173	0,078506128	0,394989135
GTF2H3	0,888810977	4,736124987	0,078541308	0,394989135
SERPINE2	1,86868217	6,971002161	0,078584302	0,394989135
ACIN1	-0,700390871	6,303465262	0,078643685	0,394989135
AHNAK	-0,73212519	7,327295716	0,078701336	0,394989135
FKBP14	1,154625535	5,097536774	0,078775461	0,394989135
CLPTM1L	0,894578619	7,474419407	0,078777713	0,394989135
FXR1	0,971493313	7,814532063	0,078884249	0,39523269
GSPT1	0,981152719	8,072254816	0,079271117	0,396879401
RPL7L1	0,909692846	6,55545964	0,079378589	0,397125897
AP2M1	1,031400684	9,159789611	0,079632176	0,397249244
LMNB1	1,298702354	4,780657174	0,079761551	0,397249244
LAMA5	-0,8472922	6,878872187	0,079780704	0,397249244
AZIN1	0,864099052	7,434739274	0,079936763	0,397249244
TMEM63B	-0,922950126	5,977049148	0,07994652	0,397249244
FBXO8	-0,856591723	4,463740476	0,080053379	0,397249244
POFUT1	1,151184173	6,622695844	0,080065918	0,397249244
S100A16	1,338091221	7,571560196	0,080080511	0,397249244
RSF1	1,010444122	6,012288738	0,080200376	0,397249244
SLC9A1	-0,688117335	5,810905535	0,080280595	0,397249244
ZNF592	-0,716321892	5,344316466	0,080350059	0,397249244
DYNC2LI1	-0,722038788	5,164825379	0,080359155	0,397249244
FAM114A1	0,997546081	6,318158172	0,080359813	0,397249244
RNF8	1,063108506	4,613352709	0,080371752	0,397249244
MMP2	-1,087545124	9,451516389	0,080377593	0,397249244
CHST11	0,976746624	6,89549149	0,080381863	0,397249244
CDKN1C	-0,900636382	4,378265269	0,0803936	0,397249244
SMN2	1,225030834	5,018503316	0,080544485	0,397249431
DNAJC3	-0,764670123	5,885562924	0,080596863	0,397249431
FAM57A	1,140760557	5,811602049	0,080678481	0,397249431
TTBK2	-0,71107233	4,299594185	0,080688147	0,397249431
SLC11A2	-0,833237577	4,962905284	0,080761358	0,397249431
PRPF4	1,019549733	4,612903163	0,080774382	0,397249431
TUBG1	0,937483034	6,696627553	0,080801431	0,397249431
PAR-SN	-0,951794194	4,972777988	0,080953079	0,397598371
DCUN1D5	1,046146724	5,406131905	0,080992187	0,397598371
CHD1	0,815312461	6,615460694	0,081047329	0,397598371
OXSM	1,103896105	3,770156131	0,081259151	0,398145425
ST6GALNAC4	-0,90698244	4,723984594	0,08131722	0,398145425
FAM98A	0,824473116	5,957556395	0,081417381	0,398145425

C15orf48	1,714381905	6,873953022	0,081438374	0,398145425
GLG1	1,006925723	5,622604785	0,08145078	0,398145425
RALGPS2	1,10110262	4,366583536	0,08162952	0,398459529
ADCK2	0,73487068	5,700165777	0,081631905	0,398459529
CHCHD3	0,981164982	6,753261426	0,081903001	0,398792203
ERCC5	-0,854255965	5,737923342	0,081945564	0,398792203
SLC25A43	1,057400378	4,983802805	0,081976941	0,398792203
MFN1	1,057490177	5,154163209	0,081989728	0,398792203
YIF1A	0,898740603	6,655160227	0,082017888	0,398792203
ARHGDIB	-0,842987907	8,204798858	0,082050955	0,398792203
FAM76A	-0,747366187	4,927585891	0,082305193	0,398960319
EPHA4	-0,874747759	4,174638708	0,082311971	0,398960319
LSM14B	0,77308564	6,109296985	0,082333223	0,398960319
PRKAB1	-0,798046073	6,948285203	0,082341131	0,398960319
TOMM40	1,065323906	6,567338545	0,08237808	0,398960319
PTPN13	-1,019293439	5,021378076	0,082580063	0,399041575
POLD2	1,086941897	7,57789588	0,082641416	0,399041575
KIAA1462	-0,940666006	7,526617169	0,08266872	0,399041575
MAPKAPK5	1,108218869	6,994725359	0,082710406	0,399041575
CHAF1A	1,272402319	4,739574971	0,082726232	0,399041575
LSM2	0,916617631	6,964387696	0,082764077	0,399041575
AKR1B1	0,953354333	6,759549401	0,082851194	0,399041575
EIF4A1	0,852132198	10,27072606	0,082878492	0,399041575
NICN1	-0,832500519	3,836920721	0,082973868	0,399041575
KDM3A	0,831204942	5,862527735	0,082980049	0,399041575
TRRAP	0,964538281	7,681084442	0,083278154	0,400159485
DNAJC11	0,844991222	5,683130606	0,083338737	0,400159485
LETMD1	-0,804475031	4,375395148	0,083388566	0,400159485
ANAPC1	0,941610658	6,067088048	0,083497205	0,400399047
UQCRH	0,85418491	7,642262663	0,083703379	0,40110565
DYNC1LI1	0,987896959	6,279188777	0,083946163	0,401614949
GABPB1	0,998524966	5,038607903	0,083971235	0,401614949
FAM122B	1,003943678	6,889834602	0,08398635	0,401614949
POLD1	0,996584149	5,128611832	0,084364363	0,403139868
EDC3	0,856117941	5,367142922	0,084512686	0,403393709
OBFC1	-0,806060131	4,985351697	0,084535798	0,403393709
TRIM37	0,76199915	5,397649909	0,084630131	0,403533984
SLC3A2	0,878146517	6,775035318	0,084683551	0,403533984
KLF3	-0,613348838	5,960741156	0,084800492	0,403654515
NID2	-0,895200804	6,054525539	0,084858376	0,403654515
NRAS	0,934068935	6,170379353	0,084886431	0,403654515
OSBPL10	1,188277682	6,108095988	0,085038357	0,404095159
FAM168A	-0,710320054	5,633961261	0,085137894	0,404286422
ATP6V0B	-0,822755877	6,038530235	0,085251791	0,404545553
NCSTN	-0,763705867	6,12567786	0,085594297	0,405888393
RBM23	-0,793467153	4,978615813	0,085669104	0,405960821
TMEM231	-0,912444056	3,668673725	0,085783339	0,405997325
ZNF317	1,059634095	4,69361341	0,085795886	0,405997325
SPA17	1,117424417	4,358692256	0,085963586	0,4065088
POLM	0,850604083	3,611309213	0,086125511	0,406898804

C16orf88	0,885955167	4,843316687	0,086196705	0,406898804
POLR3F	0,816909784	4,208630613	0,086293095	0,406898804
RNF166	-0,793401215	4,484353987	0,086362543	0,406898804
WDR5	0,701623236	5,393039101	0,086482088	0,406898804
ITGA9	-0,996509835	6,758092097	0,086566934	0,406898804
NFU1	1,003831413	6,359906422	0,086638569	0,406898804
FDFT1	-0,855023338	5,514761714	0,086655471	0,406898804
SCFD1	1,046463189	7,841918855	0,086684982	0,406898804
ME1	1,354853125	6,035708219	0,086694034	0,406898804
PSMD11	0,802718556	7,192253668	0,086718165	0,406898804
MLL5	-0,65493957	7,171996461	0,086762115	0,406898804
FBF1	1,511857623	5,576186527	0,086839446	0,406981567
LEO1	1,114986505	5,63636687	0,086915227	0,407056958
CD276	1,196070842	4,793663637	0,087328461	0,408534055
H19	2,462244592	11,08939098	0,08735044	0,408534055
NOL11	1,000438269	6,624871307	0,087635209	0,409341186
BSCL2	-0,752030787	5,801241338	0,087681819	0,409341186
UCP2	-0,750264302	6,94670369	0,08771657	0,409341186
DDB2	-0,743672176	5,563178568	0,087763134	0,409341186
PRPF31	0,802651946	6,660098309	0,087850155	0,409368931
NINJ2	-0,986910825	5,211115091	0,08788915	0,409368931
AZI2	-0,649134784	3,659622441	0,087959942	0,409419006
ZNF544	0,942506243	5,318830558	0,088055905	0,40953402
CCT8	0,94255702	7,768665958	0,088104767	0,40953402
STX3	-0,871458435	3,375573087	0,088190458	0,409653089
SCD	-0,936688072	9,098593381	0,088411366	0,410194105
COPS2	0,73195011	7,516985678	0,088427238	0,410194105
MPHOSPH8	-0,962926567	5,207317746	0,088642529	0,41091326
CTNNA1	-0,822690772	8,189838841	0,088816065	0,411438009
HSF1	1,294665732	7,856827733	0,089001533	0,412017279
EMG1	0,889023947	7,06038398	0,089162022	0,41210718
PRKAR2B	-0,911952692	4,248191482	0,089250291	0,41210718
RBPMS	-1,120522598	6,75026219	0,089324227	0,41210718
L2HGDH	1,315601813	4,07475443	0,089338293	0,41210718
ARHGEF17	-0,947692243	6,688696837	0,08942468	0,41210718
COQ2	-0,75834061	5,270955901	0,089469293	0,41210718
SMPD4	0,895830003	6,544387723	0,089488028	0,41210718
CD83	-0,979248073	5,353743122	0,089504434	0,41210718
ABCF2	0,899582794	6,600734838	0,089760808	0,413008737
C4orf46	0,977882198	3,704959733	0,090097245	0,413816572
DHX57	0,757824416	4,535478164	0,090155025	0,413816572
ISG20L2	0,886559702	6,889393718	0,09018786	0,413816572
ATP5O	0,792559425	7,973819871	0,090203106	0,413816572
ATP5L	1,0493241	10,24485024	0,090239807	0,413816572
SENP3	-0,63101542	6,223472201	0,090511027	0,414781378
CDC42EP4	-0,819258213	5,350420947	0,09062037	0,41500356
TPCN1	-0,789253807	5,700071469	0,090696904	0,415075293
ZNF282	0,901614256	5,688551201	0,090955033	0,415977443
CRBN	-0,836065462	5,21777887	0,09108318	0,416284317
MUS81	1,071093122	6,60762534	0,0912529	0,416780661

NAPG	0,814424421	6,11708615	0,091448898	0,417396277
FJX1	1,025810896	5,494136608	0,091728347	0,418391706
ESF1	0,905264474	5,981485448	0,091868605	0,418596847
PLEKHO1	0,959658095	4,632046611	0,091939258	0,418596847
TRIM26	-0,704420817	5,255782306	0,091957483	0,418596847
CRIP1	-1,04319251	8,629581581	0,092081269	0,418880704
EIF5A	0,867839075	9,795689705	0,092230272	0,418986259
SF3B14	0,861639752	6,808789947	0,092397763	0,418986259
FUT10	-0,886142923	4,725198691	0,092399618	0,418986259
EZR	-0,778403353	8,203712692	0,092404532	0,418986259
FCGR3A	-0,852316234	6,521089192	0,092487371	0,418986259
MRPL3	0,95468656	6,783176663	0,092523535	0,418986259
CEP170	0,915599199	5,657655578	0,092534581	0,418986259
TH1L	0,97747566	7,067678981	0,092667414	0,419309289
CDK7	1,025382738	6,179005599	0,092917642	0,420162731
COL1A2	1,054718208	12,61268265	0,093022505	0,420291554
MICB	1,158381252	3,972399723	0,093069401	0,420291554
ZNF780B	1,706685782	5,423677725	0,093152851	0,420381649
RHEB	0,87571399	8,79772622	0,093212649	0,420381649
EXOSC3	0,830278857	5,939105394	0,093277522	0,420396181
CEBPD	-0,974166138	9,170074985	0,093389157	0,420621309
RAB18	0,778373193	7,160296379	0,093625656	0,421133006
PLP2	1,031662153	9,454250171	0,093626285	0,421133006
PRPF39	0,888316428	4,910624551	0,0936971	0,421173716
PNPLA6	-0,853535501	5,908840243	0,093844317	0,421281655
LSM12	0,876334842	7,932711504	0,09387908	0,421281655
PABPC1	0,921659459	10,47151485	0,093906455	0,421281655
BCL2L12	1,387919155	5,422490949	0,094263835	0,422586351
POLR2J2	1,127798674	5,598826987	0,094321224	0,422586351
B4GALT1	0,942894628	7,828185166	0,094410534	0,422708755
TMEM183A	0,774975745	5,872699455	0,094692054	0,42369102
SLC29A1	-0,800021969	6,704009515	0,09477878	0,423800986
ATP6V1B2	-0,730679364	6,075967463	0,094996044	0,424494119
SLC16A1	1,227749817	5,619445204	0,095234812	0,425201003
ZNF273	1,292415518	3,938460998	0,095340094	0,425201003
EIF3M	1,057139556	7,580578401	0,095341301	0,425201003
CSF2RB	-0,892100342	5,747591138	0,095535124	0,425605756
NAMPT	1,318022916	10,0339708	0,095556887	0,425605756
SSH1	-0,661386542	6,160362401	0,095786498	0,426349952
LYAR	1,313677863	6,100863061	0,096068262	0,426474674
MAP4K4	1,070147795	6,312496455	0,096103349	0,426474674
B3GNT2	-0,871706159	5,039319977	0,096118889	0,426474674
PLEKHM3	-0,86372824	5,287338361	0,09627731	0,426474674
BRCA2	1,09135106	3,870902762	0,096327535	0,426474674
GIMAP2	-0,925862854	4,603962927	0,09634059	0,426474674
VTI1A	-0,801375652	4,305782866	0,096358405	0,426474674
DDX31	1,019097508	4,317685856	0,096436124	0,426474674
FBXO32	1,290599056	5,170368392	0,096459675	0,426474674
ALDOC	1,481297937	5,564412884	0,096505145	0,426474674
ADSS	0,755385202	6,065943372	0,096569702	0,426474674

PGAM5	0,803510686	6,078697176	0,096597685	0,426474674
YRDC	0,7731334	4,47790663	0,096627566	0,426474674
SNAI2	1,18270578	6,901759467	0,096872985	0,427281298
VAMP2	-0,86303668	8,935338037	0,097048281	0,427777781
ERP29	-0,849158327	7,360028975	0,097175816	0,428049648
ANAPC7	0,925401686	6,411560198	0,097235504	0,428049648
IMPAD1	0,766906045	7,587789373	0,097701963	0,429825604
RREB1	-0,659870691	6,298002489	0,098234859	0,431891361
RPS4X	0,872605809	12,23096897	0,098300693	0,431902336
H2AFZ	0,918059644	8,53955809	0,098420603	0,432150734
SKAP1	-0,894427923	3,439544415	0,098559022	0,432480033
SMC3	0,913999092	6,181812685	0,098708932	0,432698496
ANKFY1	-0,639194233	5,274344105	0,098795097	0,432698496
EIF3I	1,295331542	6,08321576	0,09881222	0,432698496
TJP2	-0,79957332	5,864202749	0,098891823	0,432698496
ZNHIT3	0,89060196	4,83211507	0,098926082	0,432698496
DSC2	1,209408975	6,241161063	0,099175279	0,433510404
KRT17	3,996069106	10,07370214	0,099329017	0,433759813
NCOR1	0,871771011	5,742615921	0,099359558	0,433759813
FHOD1	-0,807991717	4,338922525	0,099574737	0,434421067
KDM6B	-0,991365774	6,118761803	0,099837419	0,435170688
XIAP	0,989271748	6,010625386	0,099874194	0,435170688
TAF7	0,805747328	7,680334015	0,099953993	0,43524028
SNX4	0,810542962	5,871406254	0,100397888	0,436894193
SLC25A16	-0,63579744	5,293289315	0,100494934	0,437037597
KDELR2	0,802942941	9,406804341	0,10104914	0,439167679
FUCA2	1,031082574	7,300812218	0,101116103	0,439178794
TMEM87A	-0,708520126	5,69463407	0,101275067	0,43958923
RBM8A	1,041581977	7,523061096	0,101360514	0,439680243
EIF3A	0,802634043	7,898998447	0,101572368	0,44031912
GTF2F2	0,835528156	5,863570378	0,101637717	0,440322484
SSX2IP	1,164489449	4,344183627	0,10174368	0,440501686
SLC7A7	-0,834018591	5,766970529	0,10192979	0,441027434
ERMP1	-0,84040526	4,063217872	0,102164491	0,441225959
FAM53B	-0,6542019	4,642983267	0,102171296	0,441225959
UBA6	1,248403025	6,427765167	0,102211997	0,441225959
IL10RA	-0,806613926	6,067968271	0,102234494	0,441225959
RPIA	0,917366711	6,313019962	0,102482794	0,442017819
ENAH	1,267082423	8,815717292	0,102560396	0,442072909
TTLL4	0,946152129	4,944412099	0,102864675	0,44254002
KRAS	0,905936454	4,532334843	0,102916259	0,44254002
AFF1	-0,656329972	6,547980746	0,102923368	0,44254002
LARGE	-0,803814259	4,612786544	0,102928358	0,44254002
PRKCA	0,903874978	6,634638881	0,103132075	0,442944211
TCF7L2	-0,79922539	5,480653753	0,103152281	0,442944211
APOL4	-1,037963201	4,889908399	0,10334562	0,443495143
THUMPD3	0,97043041	4,833029309	0,103455545	0,443687648
ARMC10	0,918996412	6,649228512	0,103784	0,444555635
ELMOD3	0,948328916	5,046505937	0,103861387	0,444555635
ATF1	0,992221783	5,748175856	0,103903123	0,444555635

AMZ2	0,947977111	4,585423376	0,103927343	0,444555635
GINS2	1,2601712	5,143042947	0,104143756	0,444555635
ITFG2	-0,776680565	6,210238306	0,10418381	0,444555635
PDCD11	0,862972724	5,569534427	0,104185608	0,444555635
POLE	1,005192813	6,02008996	0,104305505	0,444555635
NOL6	0,936915408	4,580558697	0,10432138	0,444555635
TXN	0,958016791	7,783008738	0,104366311	0,444555635
RPP21	0,842928479	5,931530662	0,104567304	0,444555635
DRG1	0,7635565	6,354936922	0,104623748	0,444555635
RPRD2	-0,705216878	5,67655629	0,104626612	0,444555635
RPL22	0,887138394	5,364258577	0,104672713	0,444555635
SEPHS2	0,890903676	6,548580482	0,104763669	0,444555635
SH3BGRL	-0,677051835	7,580067891	0,104927986	0,444555635
NUP107	0,953217758	5,860074917	0,105127182	0,444555635
RAD21	1,001379399	8,028439647	0,105228912	0,444555635
DGCR6L	1,05373067	6,439679174	0,105257248	0,444555635
PER1	-1,135899258	6,990089087	0,105274588	0,444555635
HSP90B1	0,887605525	10,09533739	0,10531772	0,444555635
CDC34	1,017025219	6,485110151	0,105345383	0,444555635
BCL2L11	1,006251053	5,386993048	0,10535974	0,444555635
VCPIP1	0,856245352	6,278638408	0,10540402	0,444555635
KIAA1147	-0,651501853	5,502060333	0,105417279	0,444555635
MGAT2	0,914737482	7,338629963	0,10541736	0,444555635
FADS3	-0,895059294	4,764906492	0,105418164	0,444555635
MGST2	-0,661830441	6,515164751	0,105550718	0,444839521
USP47	0,797664518	7,168163084	0,10590526	0,446058042
WSB2	0,822096839	7,526406572	0,106002258	0,446190986
DAP	0,777657023	9,287596305	0,106091942	0,446292998
CECR5	1,269673338	5,226667963	0,106340351	0,44646282
PLEKHF2	-0,687056219	5,61821131	0,106345134	0,44646282
PFAS	1,167614834	5,150273246	0,106389038	0,44646282
RNF168	0,879126184	6,007516747	0,106394205	0,44646282
IER3	1,470957044	8,122151464	0,106512237	0,446622758
F2R	-0,946548425	5,131710999	0,10660613	0,446622758
UBE2J2	0,769542987	6,909356351	0,106644466	0,446622758
ARFGAP3	-0,724533648	5,403959345	0,106694306	0,446622758
C10orf116	-1,190059397	7,365498376	0,106895543	0,447190618
CTSZ	-0,749213825	8,541779026	0,107048319	0,447301835
CDK12	0,855118974	7,291733617	0,107091508	0,447301835
TMEFF1	1,181991324	4,193861765	0,107118917	0,447301835
FARP1	-0,710437189	6,771910307	0,107258586	0,447442718
FLYWCH1	-0,730517924	5,383888216	0,107369738	0,447442718
DCTN1	-0,660624671	6,051727014	0,107379145	0,447442718
SPRED1	1,092350322	7,009538087	0,107469388	0,447442718
GDI2	0,799571915	9,074638876	0,107480741	0,447442718
PABPN1	0,708669074	8,792739975	0,107555597	0,447481158
STYX	0,804144883	6,443887974	0,10767997	0,447725435
MAST4	-0,898238465	6,975298968	0,107783457	0,44788263
HMGB2	0,937124582	7,284201569	0,108008458	0,448544261
RAB34	1,192113535	5,100223186	0,108148297	0,448851634

STIM2	-0,64319141	4,275388337	0,108282463	0,449104121
STAT6	-0,656038412	7,044664728	0,108377255	0,449104121
SGK196	1,100685149	5,224781363	0,108406714	0,449104121
ACTR8	-0,696106603	5,450226712	0,108538837	0,449378466
DHX36	0,883183	6,27525212	0,108898615	0,45059445
BRD7	0,799059997	6,951376393	0,109154642	0,45122141
LGALS3BP	-0,731011427	7,805086943	0,109209824	0,45122141
MRPL51	0,9364458	6,842878644	0,109248651	0,45122141
RNF113A	1,109085245	5,396000112	0,109420427	0,451418662
RPS18	0,818226006	10,05770849	0,1094506	0,451418662
RNF2	0,934312321	6,037016246	0,109495009	0,451418662
CDC42BPA	0,862142491	6,941062142	0,109618159	0,451536039
CPNE8	0,782625154	4,059271659	0,109655914	0,451536039
PSMC1	0,70373968	8,012451498	0,109867178	0,452132945
DCK	1,057209314	6,281622519	0,110057326	0,452642283
SMC2	1,001000015	4,906465373	0,110304351	0,452975364
EFTUD2	0,665796515	6,249899246	0,110323376	0,452975364
SRRD	0,819623916	5,406948391	0,110337598	0,452975364
PMF1	-0,685732377	5,192820817	0,110568281	0,453594609
MLH1	1,146131649	4,424783947	0,110659572	0,453594609
CHMP2B	-0,68556615	7,328356558	0,110687994	0,453594609
MDM4	-0,653475922	5,529907014	0,111091492	0,454974704
C7orf13	1,348065275	6,408909884	0,111504105	0,456390451
TSC2	-0,57808829	5,765208334	0,111813952	0,457384127
COL16A1	-1,050729035	5,981140926	0,112006696	0,457897879
TFDP1	0,861952659	6,220337916	0,11216899	0,458286606
TPMT	1,1295153	5,655439226	0,112309814	0,45851151
BMP1	-0,671880799	5,404894793	0,112358518	0,45851151
IL17RA	-0,598606433	5,84439382	0,11258036	0,45914203
RRS1	1,121773519	5,28834035	0,112942659	0,460108572
SEPN1	-0,791089352	7,257653574	0,112962361	0,460108572
FTSJD2	-0,720183884	4,880152797	0,113019777	0,460108572
SIPA1L3	1,363996304	6,008963584	0,113215042	0,460434016
PIGM	-0,855500992	4,590864035	0,113234762	0,460434016
MAGEF1	1,247020542	5,833660241	0,113400959	0,460835006
RHOT2	0,855533451	6,183075387	0,113942318	0,462693955
ALKBH1	0,92485798	4,067685715	0,113994111	0,462693955
VAV3	-0,893511439	4,393147013	0,1140927	0,462818632
BCCIP	0,750775664	7,043909397	0,114342335	0,46355552
INCENP	1,127407886	5,530318174	0,114802641	0,464497311
NPC2	-0,824855308	9,136620098	0,114829057	0,464497311
CCAR1	0,762069805	5,971328771	0,114840614	0,464497311
LOXL1	1,482870681	5,091783994	0,114978966	0,464497311
SIL1	-0,685226021	4,978501241	0,11498081	0,464497311
CKAP4	1,065422203	7,167969861	0,115047223	0,464497311
PTPN18	-0,769357981	5,297998224	0,115064683	0,464497311
RPS3	0,965946793	11,11797898	0,115119586	0,464497311
KLF16	0,8853701	5,846994628	0,115229308	0,464665081
MBIP	-0,75224541	5,327650321	0,115379516	0,464995817
PLEKHA8	1,133058939	5,786039139	0,115489148	0,465162728

DCUN1D2	1,624909974	5,321837616	0,115725662	0,465840196
ESYT1	-0,626098206	7,220533027	0,115797258	0,465853396
CBX4	1,123793537	6,649826004	0,115899765	0,465990858
CENPV	1,606402491	5,57276731	0,116135146	0,466035103
POFUT2	1,048936893	5,11173492	0,116149225	0,466035103
TOMM20	0,739101277	7,864960229	0,116153922	0,466035103
UBE2G1	0,762121003	6,364060943	0,116208441	0,466035103
F3	1,344421705	7,043969188	0,116252487	0,466035103
SLC25A13	1,084721523	4,83842167	0,116755236	0,467775533
RNF26	0,882128133	6,232800839	0,116881822	0,468007717
UPF3A	0,848661757	5,673988436	0,11698382	0,468141238
MCTS1	0,929409925	5,725431546	0,117483569	0,469865372
GPATCH8	-0,633899156	5,143818437	0,117608737	0,470090257
WDR59	-0,679402579	5,338028592	0,118004427	0,471296046
BAP1	0,793885092	6,488803082	0,118060816	0,471296046
PRPF40A	0,81680776	6,746358299	0,118178162	0,471296046
ANP32E	1,210796444	7,289815875	0,11821776	0,471296046
PSMD4	0,732774703	6,201098835	0,118255981	0,471296046
FAM134C	-0,555421367	7,01173883	0,118525476	0,471758421
GBE1	0,909523794	5,306697712	0,118551997	0,471758421
SLCO3A1	-0,767688047	6,014310178	0,118579547	0,471758421
ADIPOR2	0,976000835	5,4477101	0,118808755	0,472166409
UBE2Z	0,736062598	8,012460181	0,118820583	0,472166409
MRPL9	0,690673801	6,678825965	0,118925573	0,472186537
LIN7C	0,841114363	7,467456766	0,11896414	0,472186537
AVL9	0,744013598	6,64167329	0,119097358	0,472440306
DHFR	0,867650645	5,856394084	0,119269692	0,47252141
AKTIP	-0,688377826	4,958014521	0,119311141	0,47252141
P4HA1	1,111729219	6,010037692	0,119325688	0,47252141
PLEKHM1P	-0,643325991	6,035631828	0,119484589	0,47277655
TMEM14B	0,901658704	5,76080471	0,119528783	0,47277655
LRWD1	0,955903741	4,581225712	0,119729344	0,473158223
PRKAB2	0,939891688	3,966567472	0,119764055	0,473158223
PRR14	-0,622829259	5,947573105	0,119847461	0,47321357
ISY1	0,943509388	5,683915558	0,120131851	0,474061974
INO80E	-0,563719174	5,833519117	0,120312256	0,47449929
ARSB	-0,624361039	5,255928448	0,120451993	0,474775804
SLC25A25	-0,941282427	4,682506772	0,12058245	0,474949697
MAP3K6	-0,816402408	6,274818644	0,120675728	0,474949697
TBC1D12	-0,659948278	4,674665457	0,120705063	0,474949697
PXN	1,057132892	7,659015192	0,120909297	0,475478949
ZMYM5	-0,743000003	3,604256027	0,121008871	0,475596249
KCNAB2	-0,737919275	5,21373808	0,121094162	0,475657311
MEAF6	-0,571636549	6,661643597	0,121291766	0,475696816
ZNF16	1,057727715	4,075249083	0,121299812	0,475696816
IQCB1	0,998481872	6,066535682	0,121313501	0,475696816
EIF2C2	1,180469481	7,660301412	0,121394378	0,475700589
TBRG4	0,916310223	5,325846733	0,121453985	0,475700589
FXYD6	-1,098620762	8,264085126	0,121762617	0,476223704
TUBGCP6	-0,789923228	6,104029912	0,121941041	0,476223704

C16orf62	-0,642325884	4,727264977	0,121947264	0,476223704
SV2A	1,140273794	3,670301418	0,121954923	0,476223704
TPP1	-0,663307017	7,573250429	0,122011785	0,476223704
TDP2	0,788620198	6,898309309	0,122138983	0,476223704
TGM2	-0,913565913	8,955616527	0,12218047	0,476223704
TPRG1L	-0,730979418	5,631375165	0,122196397	0,476223704
GCC2	-0,675732032	6,053346749	0,122216085	0,476223704
DLG5	0,939597813	6,258073927	0,122384418	0,476607279
SLC7A5P2	1,036970529	6,408083246	0,122506168	0,47680911
ZNF543	-0,665541406	4,424651214	0,122632683	0,476829129
FAM20B	0,84513615	6,123639501	0,122703513	0,476829129
CHD9	-0,657247891	6,140587702	0,122721091	0,476829129
LCMT1	-0,586817614	4,166147971	0,122993645	0,477615982
EZH1	-0,850210668	5,970255027	0,123100298	0,477698768
CALCOCO1	-0,812295269	6,202971057	0,123155072	0,477698768
STK38L	0,890783289	4,4256892	0,12335742	0,477796557
ENTPD5	0,79345149	5,019154605	0,123374465	0,477796557
HNRNPF	0,729157698	6,938284292	0,123390488	0,477796557
EED	0,929123494	5,668949612	0,123698096	0,478715843
NDUFA8	0,785394317	5,739389757	0,12380053	0,47880492
RRP15	1,144756149	4,144919195	0,123861545	0,47880492
DHRSX	0,849369791	6,060882144	0,124097564	0,479287039
SEPT6	-0,690277153	4,717650425	0,124126838	0,479287039
ATP5G1	0,801619016	6,582123658	0,124343762	0,479400083
ATXN10	-0,710561754	5,742090841	0,124386962	0,479400083
SCYL1	0,801123668	5,170269417	0,124439917	0,479400083
UTP23	0,803304597	4,682865338	0,124463628	0,479400083
NME4	1,214927408	6,326353775	0,124571811	0,479400083
ACO2	-0,638135588	5,850518526	0,124577936	0,479400083
EIF6	0,781641962	7,214603594	0,124772257	0,479877055
BIRC3	1,384013093	6,193311954	0,125188126	0,481205092
COX5A	0,753058482	7,150257076	0,125480165	0,482055911
KIAA0391	0,74050554	5,585193182	0,125637397	0,482264526
UACA	-0,692308306	6,81704815	0,125675915	0,482264526
MFSD3	0,921430644	4,342336658	0,1260041	0,482443044
NCAPD3	0,784975559	5,03283583	0,126058856	0,482443044
LPGAT1	0,795937692	6,035465198	0,126115954	0,482443044
SLC25A6	-0,968909049	8,143999376	0,126223431	0,482443044
PLEKHO2	-0,646167348	6,127451944	0,126285067	0,482443044
MAD2L2	0,74325634	5,586052444	0,1263399	0,482443044
PARVA	-0,632538623	5,601270338	0,126429113	0,482443044
ARID1B	-0,528781801	6,374351028	0,126451395	0,482443044
DUS1L	1,000528348	4,357986422	0,12646118	0,482443044
NFE2L1	0,79120086	7,497962955	0,12646894	0,482443044
RNF215	1,171805761	4,391120186	0,126500684	0,482443044
C10orf35	1,517453063	4,336539176	0,126699378	0,482816292
LIMK1	0,760866827	6,378335014	0,126785473	0,482816292
LOC642852	0,772285135	4,670151414	0,12687274	0,482816292
GPN1	0,820813957	5,571670628	0,126932021	0,482816292
ZNF740	-0,646614252	3,861308371	0,126973373	0,482816292

KCTD20	1,004932419	6,010297532	0,127063748	0,482816292
S100PBP	0,798683139	5,331374518	0,127094184	0,482816292
SHISA4	-0,753609845	4,760177968	0,127216797	0,483012995
RSU1	-0,643169561	7,225240186	0,127559074	0,484043031
ZNF638	-0,709753514	6,002207849	0,127764103	0,484551402
SAAL1	1,246271136	6,40529003	0,127945783	0,484970702
AKR7A2	-0,667803995	4,913540625	0,128018604	0,484977145
DOT1L	-0,698641833	5,185394854	0,128235563	0,485281038
FUBP3	0,753913435	6,401545021	0,128250823	0,485281038
AP3D1	0,874513057	5,843748402	0,12831232	0,485281038
IKBKB	-0,729956121	6,41955151	0,128513629	0,485772969
TFRC	-0,839496001	7,960599267	0,128680021	0,486025988
SMG5	0,82197272	7,820647001	0,128723117	0,486025988
FANCM	1,15925164	3,320369168	0,128838427	0,486172104
TOB2	-0,740752684	3,266802698	0,128904409	0,486172104
USP7	0,669487631	7,397925289	0,129163337	0,486643922
CLIP1	0,958131141	5,974038148	0,12919525	0,486643922
SLC25A19	0,760438632	3,965931626	0,129243605	0,486643922
MANBA	-0,621708647	5,847434295	0,129467855	0,487219261
CAMK1D	0,799943737	4,762589731	0,129648547	0,487603784
MTF2	0,889042886	5,388822615	0,129713046	0,487603784
RAD51C	0,708075143	4,146714799	0,129999415	0,487965998
IL1R1	-0,836981743	6,713363968	0,130019932	0,487965998
KLF6	-0,696997614	7,571733646	0,130024082	0,487965998
POLH	1,052836677	4,77580333	0,130514225	0,488958552
MPST	-0,645604367	6,409076018	0,130676037	0,488958552
DONSON	1,119245077	4,998367154	0,130808091	0,488958552
DEXI	-0,7424357	3,6954144	0,130881342	0,488958552
TMEM56	-0,714057306	3,598787403	0,130894734	0,488958552
USP30	-0,698678213	3,269310053	0,130968211	0,488958552
NCF4	-0,752620056	4,801342048	0,131025926	0,488958552
DECR1	-0,743426658	4,59270726	0,131031567	0,488958552
AFTPH	-0,577108591	6,81542873	0,131085931	0,488958552
ZNF672	-0,660935619	5,529986527	0,131199482	0,488958552
CRYAB	-0,99554319	5,751710031	0,131221774	0,488958552
VAT1	-0,604994329	8,496699867	0,131282914	0,488958552
IFI16	0,864665651	8,618176664	0,131328918	0,488958552
RSBN1	-0,701150335	3,383718591	0,131344938	0,488958552
MED12	-0,571966232	5,768717274	0,131364139	0,488958552
ATP5B	0,73450349	8,963237706	0,131626079	0,489226598
SNAP29	0,742468952	5,750397341	0,13163536	0,489226598
SMAD7	-0,592985138	7,424212677	0,131765966	0,489226598
RIPK2	1,111696208	6,043499081	0,131784957	0,489226598
TUBA1B	0,978915297	10,73503202	0,131794876	0,489226598
SOD2	-0,749365201	8,777141607	0,131993439	0,489697096
TNFRSF21	0,795570344	6,555446238	0,132223414	0,490283555
NEXN	-0,843755621	4,800423981	0,132371481	0,490565832
ARNT	-0,581711933	7,179056176	0,132650736	0,491000904
GALNT7	0,970304358	5,955181544	0,132668665	0,491000904
SLFN13	1,134933152	3,794550769	0,132709746	0,491000904

RNF125	-0,708863431	3,856936556	0,132856161	0,491000904
HUWE1	1,128210164	7,070705283	0,132905098	0,491000904
RYK	1,110837686	7,852625375	0,13294573	0,491000904
NPC1	-0,59606942	6,579783838	0,132992913	0,491000904
DGCR14	1,056974124	5,311187791	0,13309832	0,491124159
MTERFD2	-0,693522366	4,996130487	0,133289648	0,491564149
HSPA7	1,194772071	7,114502476	0,133409068	0,491738615
NMRAL1	0,754072878	5,660126744	0,133740685	0,492694615
FOSL2	-0,820244299	7,807836688	0,134034574	0,493476164
WARS2	0,79040332	4,019312945	0,134134117	0,493476164
SLC5A3	1,019339763	5,588169637	0,134169938	0,493476164
POMGNT1	0,787045978	6,520599069	0,134269058	0,493549734
FLNB	0,846755328	7,90699635	0,134385395	0,493549734
PHF20L1	0,721940432	6,454894263	0,134407077	0,493549734
FAM136A	0,750934896	6,241415528	0,134534491	0,49365039
EEF1B2	0,834703388	9,268348395	0,134695961	0,49365039
COG1	-0,675520868	5,693836332	0,134698379	0,49365039
ANO10	-0,596423485	4,727207113	0,134724062	0,49365039
EIF4G2	0,784189677	10,71893075	0,134920373	0,494104202
IGSF8	-0,796429499	5,699372168	0,13523053	0,494717777
ZNF680	1,073530149	4,979512702	0,135233016	0,494717777
CAPN2	-0,685246975	7,358660247	0,135426658	0,495053128
WRNIP1	0,834510515	7,200035228	0,135603327	0,495053128
KCTD6	0,809130247	5,226129495	0,13564252	0,495053128
TESK1	0,82452097	5,382562962	0,135667838	0,495053128
HCCS	0,717169313	4,62080503	0,135723411	0,495053128
FASN	-0,806212796	6,697056126	0,13578901	0,495053128
EHD4	-0,865892097	5,941434003	0,135920881	0,495053128
HSPB11	1,096001338	5,880866308	0,135954393	0,495053128
PCM1	-0,652224529	6,040692046	0,135978077	0,495053128
TEP1	-0,73407517	5,578337457	0,136256704	0,495802808
RAC1	0,760875719	8,354342542	0,136440138	0,495999459
METTL2B	0,838961064	5,399754227	0,136456223	0,495999459
PCGF6	0,904533703	4,501015611	0,136664612	0,496236017
SRP54	-0,689605983	6,207365329	0,136670898	0,496236017
CCDC111	-0,659077467	5,371844303	0,136739621	0,496236017
NME7	1,115545965	5,129341523	0,136945007	0,496391548
KRR1	0,675841768	6,525609891	0,136976889	0,496391548
RLF	0,802742472	5,063245311	0,137045871	0,496391548
THOP1	0,898113499	5,607387311	0,13707366	0,496391548
NSUN5	0,780717106	4,826090703	0,137314234	0,496623531
TOM1	-0,631399581	6,322226364	0,137318006	0,496623531
GNPTG	-0,678380061	6,043860376	0,137356207	0,496623531
RPF1	0,759080655	6,279372382	0,137749944	0,497773816
TRPS1	1,414467022	6,529559244	0,137825552	0,497773816
AGAP1	-0,838818794	5,141350656	0,137924625	0,497773816
SNAP23	-0,587049557	6,585113538	0,138016487	0,497773816
PHAX	0,759973891	5,417465661	0,138039344	0,497773816
DMTF1	-0,791474415	5,468997831	0,138359266	0,498288234
NSUN4	1,131339757	5,770881392	0,138388143	0,498288234

FXYD5	1,222490691	9,807296877	0,138569891	0,498288234
SSBP1	1,026478687	7,000286975	0,138580771	0,498288234
ITCH	0,758192389	6,536458949	0,138597558	0,498288234
UBE2Q2	-0,704727115	5,539073814	0,13862044	0,498288234
CPNE1	1,356999414	5,177760671	0,13873594	0,498382555
ARHGAP10	-0,81355564	4,232346545	0,138792854	0,498382555
MCM5	0,963568616	5,867162663	0,138969703	0,498635298
ARPC1B	-0,618710022	6,630069844	0,139069803	0,498635298
ERCC8	0,892366815	3,835628266	0,139155462	0,498635298
AMIGO2	1,341248877	4,786382045	0,139155737	0,498635298
TGFBR3	-0,978142382	4,918832073	0,139617097	0,500025728
C6orf47	0,720957454	5,675761614	0,139771798	0,500317002
SENP2	0,9661838	6,135090323	0,13990527	0,500532024
SSSCA1	0,955162898	5,638355988	0,140131422	0,500855371
PEX10	0,925795759	5,56342318	0,14014255	0,500855371
ZDHHC2	-0,75984482	4,373943624	0,140655387	0,502360858
EXOSC7	0,779038741	5,709489788	0,140711136	0,502360858
SNRPB	0,831914645	8,275052766	0,14098722	0,502917423
PKD2	-0,756960087	4,464084893	0,141048243	0,502917423
FOXO3	-0,784018069	6,31480211	0,141194859	0,502917423
COPG2	0,908979486	3,744296431	0,141233605	0,502917423
ARL6IP6	0,835370836	6,00404715	0,141284989	0,502917423
C22orf28	0,699348384	6,487998393	0,141358811	0,502917423
ALAS1	-0,54604651	5,942821929	0,141383297	0,502917423
GPSM1	-0,918053863	4,454172372	0,141711144	0,503820799
ARHGEF11	-0,762851324	4,465431911	0,141911458	0,504270055
TOMM70A	0,854736185	6,72156503	0,142078402	0,504579812
NUPL2	0,761492898	6,89725426	0,14217275	0,504579812
ASPH	1,185343951	6,718614249	0,142220619	0,504579812
ERCC3	-0,649065484	5,249446263	0,14255766	0,505259607
ZNF841	0,779502202	6,175318907	0,142560417	0,505259607
ELF1	-0,680261451	6,704692947	0,1429486	0,506238245
GRSF1	0,73836919	5,359100918	0,142985021	0,506238245
FAM35A	0,755277545	5,384543152	0,143179052	0,506662144
GALNT2	0,735584929	7,171527973	0,143256022	0,506671585
GPR125	1,160965946	5,233723083	0,143938794	0,508572093
CWF19L2	-0,628361164	5,463756534	0,144004691	0,508572093
INO80C	-0,707686938	4,331273384	0,144071503	0,508572093
CLCN2	1,531790865	5,356510049	0,144280469	0,508572093
RANBP9	0,752055717	6,434311106	0,144322358	0,508572093
ZNF25	-0,667988872	4,373997712	0,144392544	0,508572093
PLEC	0,917729719	6,075011019	0,144416382	0,508572093
UQCRC1	0,702116443	6,0891283	0,144435582	0,508572093
C1orf112	0,886870551	4,972712351	0,144464605	0,508572093
CIRH1A	0,759086018	6,31153985	0,144555326	0,508628878
RPL10	-0,61740271	10,56702719	0,144694023	0,508854329
SLC4A1AP	0,731983335	4,877430534	0,144788765	0,508925044
PYGB	0,880185837	7,82648319	0,144950085	0,509229588
VIM	-0,725089095	11,68648292	0,145104417	0,509509279
DNAJA2	0,730844515	7,21319431	0,145292157	0,50990593

TSPAN15	-0,743165305	5,809600322	0,14539085	0,509921119
NUP37	0,935841916	4,476922749	0,145465209	0,509921119
EIF2B5	1,015150153	6,40378606	0,145588498	0,509921119
BCAT2	-0,75828955	5,70723072	0,145613922	0,509921119
CEP97	0,90430391	5,226651308	0,145696445	0,509921119
POLR2C	-0,641692873	5,128487703	0,145745162	0,509921119
FAM49B	0,808868487	5,785943223	0,145828855	0,509952288
CELF1	0,745063653	4,893846689	0,14629723	0,510927259
SMARCE1	0,610393987	6,835798696	0,146354154	0,510927259
TOM1L2	-0,823846056	5,996916423	0,146376342	0,510927259
CPNE3	-0,621094109	7,187525009	0,146407371	0,510927259
RCN2	0,723415807	5,862791893	0,146741657	0,511831896
CCDC43	0,738185081	5,363755321	0,146856429	0,511970341
GMPR2	-0,692886219	4,575210314	0,146961734	0,512075659
NUP88	0,808800917	5,308529292	0,147104915	0,512312778
NPEPPS	0,640999447	7,70917787	0,147267643	0,512617692
FAM48A	0,996308901	3,193813394	0,147665243	0,513259248
PDXP	0,917860885	5,326654905	0,147708648	0,513259248
FAM76B	0,846985404	3,88661627	0,147740107	0,513259248
LGALS8	-0,626389822	6,360751352	0,147803319	0,513259248
SLC25A46	0,808545119	5,93375441	0,147828298	0,513259248
COG5	-0,591002529	5,920101272	0,148017535	0,513294569
LAPTM4B	0,982617633	8,3646744	0,148158782	0,513294569
PITPNB	0,641915927	7,143064639	0,148531101	0,513294569
CYB5R1	-0,614603429	5,894371955	0,148574727	0,513294569
GTF2H1	0,901301102	6,76733225	0,148617214	0,513294569
WDR48	0,931524088	6,202624341	0,148619629	0,513294569
CDC42	-0,63386549	8,378637916	0,148642433	0,513294569
GPNMB	-0,862380356	9,063352431	0,14864269	0,513294569
ANKRD16	1,082399008	4,342781063	0,148760239	0,513294569
ELOVL5	0,751532992	8,352024097	0,148787276	0,513294569
HSPA1A	1,544395353	11,55917213	0,148790462	0,513294569
SLC25A40	0,810384711	5,320350814	0,148798896	0,513294569
PROSC	-0,587885459	4,854243372	0,148817035	0,513294569
AP4E1	0,830265533	3,553054381	0,149004268	0,513680539
FAM102B	0,644407725	5,700481376	0,149255929	0,514138218
ATP6V1D	0,718913123	6,630771447	0,149320529	0,514138218
PLSCR1	0,811297775	7,448117646	0,149547389	0,514138218
FBXL6	0,916908217	3,995320153	0,149592592	0,514138218
FDX1L	0,773305941	5,487431185	0,149613925	0,514138218
ATP2B1	0,818137559	5,737297555	0,149621538	0,514138218
TSG101	-0,578483574	5,525980327	0,149706343	0,514138218
GSN	-0,700819003	8,605876933	0,149885679	0,514138218
NARF	0,789780754	5,191977924	0,149889406	0,514138218
C14orf169	0,882982829	4,18430117	0,149891007	0,514138218
FAP	1,213039669	5,483422455	0,150006322	0,514275067
CMPK1	0,747273688	7,628606595	0,150152857	0,514313277
PHGDH	1,486497863	5,463762421	0,150226354	0,514313277
DDX27	0,784268899	6,107254506	0,150289102	0,514313277
XRCC4	0,798181165	4,432619763	0,150346679	0,514313277

MYL9	-0,975577242	8,049739531	0,150394585	0,514313277
RDBP	0,759129341	5,834270904	0,150839423	0,515467018
ATRIP	0,78906846	3,414865827	0,150895202	0,515467018
PGM2	-0,623704877	3,484142108	0,150958738	0,515467018
CAB39	0,868688281	7,613190042	0,151107465	0,515716618
FCER1G	-0,807324946	8,860820423	0,151530871	0,516902956
FKBP10	1,197315136	7,392204891	0,151764072	0,517439603
ING2	1,245443283	5,425957048	0,1518416	0,517445212
PCCA	-0,623130014	5,623033276	0,151940202	0,517522596
DUSP16	-0,648682298	6,035669503	0,152077118	0,517627494
INTS3	-0,834071401	4,805071755	0,152183868	0,517627494
DPP3	-0,660536876	4,576180389	0,152198728	0,517627494
UBA7	-0,690917826	7,196097045	0,152344026	0,517644678
ARHGAP27	-0,604620506	5,225214884	0,152358933	0,517644678
PHF3	0,868772592	7,423434249	0,152431517	0,517644678
NOTCH2	-0,568359249	6,349697894	0,152530211	0,517722005
HPRT1	1,196568398	6,186995147	0,152733422	0,51777673
ITSN2	-0,733769747	5,342737415	0,152777108	0,51777673
AGPAT6	1,122121927	6,688774492	0,152799982	0,51777673
NARS	0,691426428	7,208675931	0,152920769	0,51777673
MCM3	0,765649749	7,129776875	0,152934384	0,51777673
ALAD	-0,631539263	5,222446661	0,153001923	0,51777673
VRK1	1,113496208	5,541419453	0,153221634	0,518067258
WBP11	0,649448072	7,289820917	0,153299392	0,518067258
SERP1	-0,548916494	7,507998908	0,153436113	0,518067258
UBP1	0,724683203	6,701746674	0,153457655	0,518067258
WFDC2	-1,125803332	8,499515568	0,153467644	0,518067258
PNPT1	0,719456358	5,623657729	0,153599279	0,518255063
ADAMTS1	-0,985998372	6,440776178	0,153804916	0,518692246
SEPW1	-0,841451711	4,21174528	0,153894928	0,518739256
CAD	0,741807639	5,789378779	0,154293204	0,519665187
RCN1	0,686519078	8,623326421	0,154322042	0,519665187
HSF4	0,812635692	5,28140044	0,154543116	0,52015277
PGAM1	0,606282558	7,468192088	0,154864011	0,520975673
MSTO1	0,818159568	4,922576402	0,155126566	0,521601604
RBM33	0,716702593	6,398491588	0,155276301	0,521847755
SMO	1,46296322	5,202130358	0,155824365	0,523431698
PHKG2	1,16788688	5,863741067	0,156048565	0,523926717
ELP2	-0,646384416	4,62851998	0,156312607	0,524554956
UBN2	-0,588571024	4,705945907	0,156421712	0,524631895
ADM	-0,909182615	6,337659972	0,156583686	0,524631895
TASP1	0,866500173	4,484661209	0,15664848	0,524631895
C12orf52	0,734813748	4,635207392	0,156676153	0,524631895
TMEM159	-0,704267788	4,832470663	0,156720219	0,524631895
C4orf52	0,780068066	5,622960628	0,156889542	0,524941015
DENND4A	-0,587144643	5,190825278	0,157442859	0,525726682
LSM14A	0,68229008	7,596861232	0,157487294	0,525726682
COL4A2	-0,853648175	7,867129481	0,157584278	0,525726682
C16orf5	-0,527303961	5,223185183	0,15760358	0,525726682
GADD45GIP1	0,707663489	7,481702572	0,157709115	0,525726682

CORO7	-0,625586143	3,849225926	0,157721673	0,525726682
NDUFB11	0,697815574	7,879025799	0,1577883	0,525726682
UBE2L6	-0,716576712	5,843135732	0,157990209	0,525726682
DENR	0,887856159	7,38317734	0,158027858	0,525726682
DTX3L	0,838516486	6,856635994	0,158069695	0,525726682
MTIF2	0,747388504	5,820255852	0,158075706	0,525726682
RSL24D1	-0,7185828	6,80016275	0,158268857	0,525726682
CXCL9	1,579173595	7,202191109	0,158385201	0,525726682
C14orf2	0,660016245	8,371765847	0,158414757	0,525726682
MRS2	0,80063213	6,180204466	0,158455737	0,525726682
RAD52	0,888865475	3,89165331	0,158472651	0,525726682
TFCP2	-0,678292196	4,634452321	0,158505302	0,525726682
NUS1	0,890020685	6,966645399	0,158615663	0,525726682
RNF219	0,915212527	4,399327516	0,158692401	0,525726682
STXBP3	-0,602167772	5,387369833	0,15873099	0,525726682
AMPD2	0,890486164	6,678722169	0,158743399	0,525726682
ALG1	-0,632385898	4,618801685	0,158859494	0,525855772
RAP2B	0,852165435	6,023527599	0,159251129	0,526896385
NDUFB8	-0,548771405	8,095432981	0,159462968	0,527341407
IGBP1	0,966128483	5,805344517	0,159574498	0,527421319
ATP11B	0,73734492	6,99472332	0,159652527	0,527421319
METTL9	0,690806761	6,600244784	0,15971917	0,527421319
TMEM14C	0,778494841	7,424958493	0,159947621	0,527804394
MAPK7	0,915033436	5,575284008	0,16003711	0,527804394
RGS19	-0,607360913	4,173201572	0,160067383	0,527804394
FNDC3B	0,607921118	8,57105353	0,160299765	0,528315175
CD320	0,918615514	5,38697372	0,160645216	0,529191547
TSC1	-0,573800328	5,204365001	0,160787108	0,529191547
ZNF160	-0,546727797	5,019933465	0,160832625	0,529191547
EBNA1BP2	0,629558639	6,589389227	0,160876093	0,529191547
ZNF3	0,892620703	5,369018916	0,161094679	0,52965507
IRF5	-0,619131995	5,253799292	0,161311221	0,530111429
C4A	-1,014660397	4,709214525	0,16168623	0,531087863
TUBA4A	0,869211539	5,967402284	0,162085001	0,53214137
SEC24B	0,70446147	6,374198044	0,162336023	0,532709019
RPP40	1,17182727	5,470818971	0,162497246	0,532981587
SNX2	-0,617208863	3,982693923	0,162743414	0,532992862
ZNF260	1,239532398	6,078675397	0,162797612	0,532992862
TMEM41B	-0,613151123	3,576561528	0,162820005	0,532992862
MTA2	0,821846886	6,533735096	0,162842287	0,532992862
ARHGEF9	-0,581240211	4,62414687	0,162915768	0,532992862
ARRDC1	-0,605490438	4,583382939	0,163053434	0,532992862
RNASEH2A	1,029714575	4,783513413	0,163111584	0,532992862
AP3B1	-0,617332694	5,132969716	0,163185757	0,532992862
SLC30A7	-0,622005664	5,20370335	0,163231939	0,532992862
ZFAND2A	0,714304504	5,379879903	0,163379067	0,532992862
RPS16	1,372974007	12,39664649	0,163389531	0,532992862
PICALM	0,93165802	6,620316194	0,163456513	0,532992862
KLHDC5	1,181726024	6,11301471	0,163516801	0,532992862
AKIRIN1	0,715347873	7,244976777	0,163779	0,53351778

GMCL1	0,7016215	4,734730866	0,16383432	0,53351778
RHOBTB3	0,987651506	6,776958808	0,163917849	0,533534994
GOLGA4	0,809680591	6,514835089	0,164010087	0,533580526
ZDHHC18	-0,535764327	5,868555387	0,164234259	0,534055036
MC1R	1,096102808	5,103696179	0,164315051	0,534056544
PSMA6	0,825436047	8,141651484	0,16439136	0,534056544
MAP2K4	0,725008506	5,77441785	0,164598731	0,534475594
MRPS6	0,905126316	6,56933361	0,165014388	0,535570259
LOXL2	0,896539602	6,987307704	0,165113229	0,535636113
GGA3	0,888146799	5,300971566	0,165209516	0,535693625
FEN1	0,886081642	4,507665767	0,165316711	0,535786433
SLC25A3	0,778822871	8,287074213	0,1656451	0,535944849
ADAT1	0,681338619	5,699854011	0,165729906	0,535944849
ABCA5	-0,817726827	5,565560664	0,165891401	0,535944849
ZNF511	0,88824787	4,080455641	0,165892502	0,535944849
PTP4A3	-0,913576029	5,047353018	0,165994606	0,535944849
EFNB1	-0,702780656	6,699450599	0,165998943	0,535944849
UFD1L	0,674888889	6,289349045	0,166110282	0,535944849
GNPAT	0,833432271	5,925950934	0,166125301	0,535944849
SUN2	-0,615703257	7,185407504	0,166127776	0,535944849
MINA	0,775382773	5,475188463	0,166151549	0,535944849
KIAA0020	0,837720059	6,700536909	0,166768298	0,537679918
GTF2H4	0,789780415	5,962352123	0,16693304	0,537862699
APOOL	0,800745779	5,670052098	0,166982744	0,537862699
EEF2K	-0,60216601	4,831514044	0,167423526	0,538678579
UBXN2A	0,770796095	5,389924564	0,167466134	0,538678579
POLR1C	0,885003594	5,258512487	0,167476613	0,538678579
TANC2	0,746058298	6,322840233	0,167552026	0,538678579
PA2G4	0,738952602	7,718190768	0,167642648	0,538715936
BHLHE40	-0,639702021	8,103453799	0,167760181	0,538839696
FAM134A	-0,589908073	5,88247548	0,167867303	0,538929915
SYNCRIP	0,686172256	7,802782623	0,168491078	0,540464061
MAP3K2	0,585542746	6,606721104	0,16852856	0,540464061
TMEM141	0,571946837	6,121925448	0,168582938	0,540464061
MYEOV2	0,631591457	9,06306121	0,168701963	0,540591489
SUCLG1	-0,546870053	4,96402904	0,168979101	0,540604717
SNX14	-0,702810982	5,511196155	0,169017168	0,540604717
SYK	-0,639503178	4,659177681	0,169023373	0,540604717
CUL2	0,943908659	5,730980476	0,169072607	0,540604717
CLCC1	-0,685738243	5,066248968	0,169102487	0,540604717
PMS2L2	0,692616356	5,187320857	0,169424891	0,5413816
NOL7	0,777032405	7,001710863	0,169790625	0,542090453
ARL3	0,70693272	6,940043754	0,16980572	0,542090453
TPM2	1,924765382	8,818910955	0,169913162	0,54217962
MRPS7	0,774805003	6,123956376	0,170031075	0,542302105
EGLN3	1,12306724	6,003925418	0,170123415	0,542342948
RPS5	0,813067062	10,54599917	0,170491906	0,542880442
HDAC3	0,69299163	6,728011734	0,170506047	0,542880442
LRP6	-0,753388881	6,361330372	0,17054104	0,542880442
PPP3CA	-0,654233287	5,922541309	0,170610469	0,542880442

ACTN1	0,802176011	8,753779293	0,170760296	0,543103758
RFC3	0,858789706	4,578438324	0,171451244	0,544729519
PPP1R10	-0,648699489	5,394981363	0,171497021	0,544729519
DAB2IP	-0,655121454	6,001248895	0,171511113	0,544729519
C1R	-0,900341798	7,48812146	0,171853108	0,545067706
SLC25A1	1,072479204	6,930792499	0,171886978	0,545067706
MIIP	0,649548028	6,245168358	0,171899705	0,545067706
IL13RA1	0,991994098	6,715462423	0,171985956	0,545067706
ANGPTL2	-0,802207526	5,892421764	0,172027244	0,545067706
RPA2	0,687135896	5,808490689	0,172097195	0,545067706
ANP32A	0,696970176	7,562366824	0,172408539	0,54559745
LANCL1	-0,649038464	5,387636828	0,172424476	0,54559745
TAF5L	0,738684081	5,397980865	0,172705517	0,545979304
MYBBP1A	0,737744682	7,14745425	0,172729714	0,545979304
COX7B	0,790518209	8,407891692	0,172911443	0,545979304
SH2B1	-0,775737391	6,149555248	0,172917239	0,545979304
NACC1	0,786763923	6,122130024	0,172997571	0,545979304
NSMCE2	0,803316276	5,444474902	0,173025557	0,545979304
SLC31A1	-0,686746333	5,49028731	0,173318674	0,546445192
F13A1	1,127655873	7,129663657	0,173333473	0,546445192
SGCB	-0,665282635	5,645893304	0,173563849	0,546918616
SMNDC1	0,76091913	5,60216017	0,173689465	0,547061645
MRPS33	0,768116501	4,154415748	0,173991926	0,547761284
SMYD4	-0,679934514	2,860900194	0,174276318	0,548212774
GTF3C1	-0,58582884	6,506057701	0,174296128	0,548212774
PPP1R15A	-0,682713184	7,441356557	0,174627724	0,54880892
NGFRAP1	0,979695947	6,85725259	0,174646628	0,54880892
BCL9L	0,892563086	7,770284075	0,1748146	0,549074836
C11orf73	0,69452797	5,693872899	0,174916884	0,549074836
PPT2	0,837502682	6,164598048	0,174972814	0,549074836
PGP	0,857153406	4,794922536	0,175251573	0,549696632
BCLAF1	-0,558279302	6,591860566	0,175339638	0,549719995
TRIM25	-0,521484194	5,59358595	0,175528993	0,549769411
REL	-0,580639588	5,639175419	0,175555393	0,549769411
PRKCD	-0,582553934	4,506263196	0,175618403	0,549769411
POLR2D	0,69398402	5,679598185	0,175714061	0,549769411
PACSIN2	-0,528124484	6,285951379	0,175876676	0,549769411
UBL4A	0,785885726	4,834860851	0,175946222	0,549769411
DOCK7	0,813572318	4,292579089	0,176049459	0,549769411
KRT18	1,200130645	8,691351006	0,17606526	0,549769411
MICAL3	1,038536766	6,219139205	0,176212971	0,549769411
ZNF22	1,117824941	5,776343287	0,176231812	0,549769411
ZNF264	-0,526541505	5,687265912	0,176242254	0,549769411
PAK4	1,229829078	7,855560678	0,176678173	0,55041349
CRKL	1,081925127	6,436164592	0,17676235	0,55041349
CYP51A1	0,69993359	6,92059832	0,176830998	0,55041349
ZNF426	0,806583804	4,965868386	0,177002246	0,55041349
AGL	0,744525368	5,564189253	0,177192821	0,55041349
NES	-0,736806902	8,309536552	0,177210144	0,55041349
RNGTT	-0,630631043	3,518823896	0,177259668	0,55041349

GNA11	-0,638143644	5,317592671	0,177304312	0,55041349
SPEN	-0,660496357	6,342432361	0,177455997	0,55041349
ZNF7	0,761894312	4,834777765	0,177514471	0,55041349
RPLP0	0,732828577	12,2073059	0,177519155	0,55041349
MYNN	0,909631046	5,782814111	0,177755239	0,55041349
FBXO46	0,749276193	4,163666398	0,177797084	0,55041349
ADAM8	1,14039138	4,552732898	0,177837046	0,55041349
IFITM1	-0,691303853	9,642746282	0,177858118	0,55041349
CCDC117	0,640099835	6,128041431	0,178088387	0,55041349
RAD50	0,780587617	5,54922451	0,178183373	0,55041349
CRY1	0,751836546	5,752740719	0,17822258	0,55041349
MAML2	-0,628191219	5,836473221	0,178365286	0,55041349
YIPF5	0,662207045	7,565368136	0,17858666	0,55041349
SAMD4B	1,139524969	7,767050641	0,178601789	0,55041349
RPS2	1,032354024	11,02131154	0,178631266	0,55041349
GLT8D1	0,617437715	6,786427354	0,178728433	0,55041349
VEZT	0,5798665	7,367229237	0,178742316	0,55041349
LRCH4	-0,549486402	6,098907087	0,178754102	0,55041349
ATG2A	-0,599656234	5,106301476	0,178783461	0,55041349
ARL5A	0,697198558	6,864870076	0,17879423	0,55041349
HECA	-0,629605651	5,839593408	0,178921692	0,55041349
TMSB10	0,814510851	12,58738837	0,179073938	0,55041349
PSMB2	0,609577677	8,981615605	0,179084806	0,55041349
SIKE1	0,697032169	6,272952141	0,179151615	0,55041349
ZNF33B	0,93086225	5,103657892	0,179265787	0,55041349
MRPS18C	0,67558354	6,044450509	0,17929042	0,55041349
CAND1	0,722635924	7,272554996	0,179382895	0,55041349
PWP2	0,776458317	5,249469885	0,179530423	0,55041349
GOSR2	0,793307044	5,314268674	0,179593994	0,55041349
RNF34	0,687039193	5,849521921	0,179609445	0,55041349
ACBD3	0,680525335	6,466080389	0,179671239	0,55041349
ANKMY1	-0,656999489	4,60549514	0,179674382	0,55041349
CFL1	0,623390267	11,17641617	0,179707732	0,55041349
HK1	0,63075812	5,280472185	0,179758153	0,55041349
GSPT2	1,010814763	4,974531027	0,179989772	0,55087534
EID2	0,782843627	5,312642975	0,180160609	0,550947779
NDFIP1	-0,543440271	6,394224519	0,180243013	0,550947779
UXS1	-0,529208388	5,58414956	0,180255829	0,550947779
CIITA	-0,740381859	6,661688375	0,18048376	0,551342561
GOLGA7	0,982619807	7,664000328	0,180579441	0,551342561
MRPL23	-0,667052111	6,065696135	0,180627553	0,551342561
SLC19A2	-0,560398329	3,474521432	0,180785161	0,55157674
CD151	-0,612899363	7,923884741	0,181059384	0,551876103
SNRPF	0,752322792	7,649160215	0,181119501	0,551876103
CTTN	0,752957198	8,171219198	0,181126077	0,551876103
SDHAF2	-0,54158593	5,635316558	0,181391751	0,552438745
SEC61A2	0,740041417	4,425179769	0,181592947	0,552804601
PSMC4	1,040556754	7,57034306	0,181760033	0,553066338
SLC7A6OS	0,652275134	4,885065125	0,181990086	0,553371874
FAM120B	-0,555241209	4,811006523	0,18208491	0,553371874

WWP2	-0,53973344	5,964975859	0,182103899	0,553371874
TRNP1	0,870820185	5,403110984	0,182640563	0,554755455
EIF2AK2	0,707776383	7,658544456	0,182726181	0,554768402
LUM	-0,929802362	9,949191844	0,182808096	0,554770097
PELP1	0,908171762	7,035863121	0,18308169	0,555171275
MALL	-0,750944185	7,67878603	0,183103123	0,555171275
C17orf70	0,726442182	3,949978933	0,18321888	0,555275353
HAPLN3	0,914846562	4,348212065	0,183425438	0,555654405
BAK1	0,734715723	5,457486963	0,183539661	0,55575353
SEC61B	-0,518882989	7,80900037	0,184049511	0,557049983
ZNF800	-0,589202995	5,98354693	0,184434129	0,55773546
ALKBH8	0,838030432	4,565323593	0,184590223	0,55773546
PLEKHA2	-0,776781374	5,366395733	0,184625557	0,55773546
TMED10P1	1,093394423	5,027552979	0,184648102	0,55773546
ZFYVE21	-0,549344274	4,458269091	0,184684949	0,55773546
EIF2B2	-0,602863396	4,920878063	0,185238678	0,559056008
PHB2	0,630194914	8,191666053	0,185286197	0,559056008
ZBED5	-0,572526888	4,283394315	0,185453428	0,559313103
ABCF1	0,626019986	6,579269976	0,185780771	0,559879914
WDR60	-0,637622947	4,03628766	0,185805579	0,559879914
RPL32	0,816151821	11,2840226	0,185968022	0,560057635
OST4	0,591853543	7,98026193	0,186028823	0,560057635
ERAL1	0,738767144	6,519894748	0,186382804	0,560757214
NDUFB7	0,855395359	7,347789912	0,186497665	0,560757214
PSEN1	-0,551403231	5,606645001	0,186507899	0,560757214
SLC35B1	0,667968614	6,709098285	0,187021989	0,562055064
CHPF	0,883739334	6,29082128	0,187232929	0,562312742
IP6K1	-0,610253716	4,962349942	0,187272655	0,562312742
PSD3	0,912626317	4,903002021	0,187630099	0,563093584
TMEM69	0,771756321	5,979820787	0,187733387	0,563093584
ACTL6A	0,807351312	4,986239413	0,187841072	0,563093584
GALT	0,690574002	3,737228717	0,188064657	0,563093584
FDPS	0,736068693	5,879172577	0,188257138	0,563093584
MYH10	-0,886333433	7,310594553	0,188283626	0,563093584
MCM3AP	0,67172757	5,686892077	0,188456336	0,563093584
SNHG12	0,649740156	5,498023679	0,188539898	0,563093584
CYTH1	-0,676914126	5,865624016	0,188560344	0,563093584
NAA15	0,878102907	5,66892499	0,188635839	0,563093584
DDX51	0,847024298	5,510345635	0,188639347	0,563093584
CFL2	1,042528934	7,049786847	0,188659237	0,563093584
SPARCL1	-1,090417154	9,078972256	0,188738721	0,563093584
C2orf49	0,627454542	6,159255326	0,188796866	0,563093584
GLRX	-0,698325774	5,545901374	0,188798746	0,563093584
RPL7A	0,711372893	11,3694869	0,188853941	0,563093584
HSBP1	0,650068241	7,161543069	0,18917551	0,563625844
ITPRIPL2	0,760510126	7,753893339	0,189385593	0,563625844
LY75	-0,674680497	3,791157451	0,189450887	0,563625844
TRIM28	0,634489571	7,697234208	0,189575631	0,563625844
UBE2L3	0,65091278	8,050738333	0,189705069	0,563625844
NDUFA3	-0,54499188	6,492627034	0,189765055	0,563625844

AP3S1	0,881319805	6,264157797	0,189781229	0,563625844
TIPIN	0,726498438	4,901984649	0,189847692	0,563625844
NUMA1	-0,473752488	7,323781703	0,189858398	0,563625844
MXD4	-0,609657762	6,605716856	0,189913226	0,563625844
GRWD1	0,647017139	6,09431577	0,190023671	0,563625844
ACLY	0,629289823	7,453746116	0,190024317	0,563625844
ZNF585A	1,008521215	6,052226347	0,190196515	0,563834554
IPO4	0,669874174	5,643463725	0,190307656	0,563834554
GLCE	-0,593192012	3,602512612	0,19034274	0,563834554
CCDC82	-0,629301257	4,514469331	0,190577051	0,564283505
ZRSR2	-0,579940243	3,48216168	0,190942384	0,564875581
ZDHHC9	0,706802667	6,438905189	0,191163569	0,564875581
MTSS1	-0,696411183	5,532173022	0,191178801	0,564875581
HNRNPA1	0,765773333	6,664885136	0,19119034	0,564875581
PPM1D	-0,586129653	4,484607468	0,191217082	0,564875581
IFITM3	-0,668720573	9,640143107	0,191274046	0,564875581
DTX3	-0,583180541	6,175772247	0,191446762	0,565140896
NDUFS8	0,59059889	7,350554146	0,191588681	0,565315109
HAT1	0,885995151	6,979361997	0,191804718	0,565535851
UNC119B	-0,53689709	4,505597672	0,191956675	0,565535851
GRB2	-0,621110053	6,287838624	0,191959206	0,565535851
PPIB	0,662642431	9,943904733	0,19220036	0,565535851
CDKN1A	-0,733065852	7,623021078	0,192402014	0,565535851
YTHDF1	0,599838803	6,674855699	0,192448249	0,565535851
PUM1	-0,587500323	5,676455385	0,192449358	0,565535851
TRAP1	0,604800839	4,798207908	0,192490359	0,565535851
SON	0,607203384	7,889431706	0,192536995	0,565535851
RNF135	-0,576745882	5,013866646	0,192693909	0,565535851
SMCR7L	0,676470375	4,992834561	0,1927544	0,565535851
SLC25A22	0,789573243	5,46167617	0,192766646	0,565535851
POLDIP2	0,788954722	5,998874145	0,192766714	0,565535851
DLST	-0,526943416	6,544684682	0,192824586	0,565535851
HSPE1	0,678973197	7,052018391	0,19308859	0,565941009
MAD1L1	-0,570598995	4,847144089	0,193145973	0,565941009
CAP2	0,863512563	4,278959507	0,193211713	0,565941009
NDUFA2	-0,655727424	6,478652818	0,193529893	0,566629601
SFMBT2	0,751006909	5,450752904	0,193782341	0,567125229
SEPT7P2	-0,547093777	5,506763862	0,194049704	0,567465884
MED23	-0,551798579	4,498431651	0,194065177	0,567465884
HSD3B7	0,734893733	4,418005454	0,194180127	0,567502361
MRPL20	0,565984434	7,766456323	0,194244099	0,567502361
TNIP2	0,827432237	5,697536054	0,194566613	0,56820117
SAC3D1	0,740857949	3,411619018	0,194738566	0,56845988
GHITM	0,669313044	6,962773314	0,194993859	0,568961542
FAM91A1	0,699732249	6,896371876	0,195232904	0,56922794
NVL	0,677476874	4,695553869	0,195369176	0,56922794
GFM2	0,715401746	5,149050185	0,19541115	0,56922794
PREB	0,642770344	5,849337706	0,195419066	0,56922794
GPX4	-0,518137233	7,875500524	0,195528091	0,569230196
YEATS2	0,976978041	7,335736242	0,195685668	0,569230196

LHFPL2	0,826714465	5,79687554	0,195749426	0,569230196
LSG1	0,766395817	4,549498311	0,195757782	0,569230196
DYNC1H1	0,575292757	7,404459834	0,195906477	0,569230196
TMEM160	0,715339969	5,063379639	0,195920703	0,569230196
CHD1L	0,90248208	3,912984089	0,196314293	0,570092049
EP300	-0,701059149	4,813426726	0,196384547	0,570092049
RLIM	0,65959013	6,980670055	0,196802965	0,570822087
ATP2B4	-0,615171436	7,691707678	0,196880682	0,570822087
TIA1	0,868080673	6,153237564	0,196920739	0,570822087
STEAP3	0,850204275	6,649038599	0,196970871	0,570822087
SIRT1	-0,516366144	3,997214967	0,197187618	0,571135639
ABCA11P	0,737959627	6,225864869	0,19724658	0,571135639
FAM192A	0,560746729	6,407582164	0,197436738	0,571443598
UBA5	0,612193904	4,755800613	0,197574704	0,571591606
COPB2	0,663875796	7,180951582	0,197699845	0,571591606
PLEKHG2	1,413766471	7,379886891	0,197739346	0,571591606
ADCY3	-0,591428678	5,275190373	0,19811042	0,572421591
PMM2	0,752323884	5,358656195	0,198236394	0,572484632
COX7A2L	0,777867336	5,594752784	0,19838106	0,572484632
ZNF670	-0,59888674	3,210588994	0,198384101	0,572484632
ITGA2	1,284884834	5,968161291	0,198555002	0,572735431
RPL11	0,652219158	12,43612599	0,199003763	0,573787171
PPM1B	-0,566933637	6,57249778	0,199097701	0,573815394
GDAP2	-0,492568096	5,279729079	0,199321365	0,574217316
RFWD2	0,594688759	6,367915373	0,199703547	0,575075375
RAB7L1	0,742859493	4,468075488	0,199859257	0,575146048
ZDHHC17	-0,604313104	4,342089252	0,199896779	0,575146048
CINP	0,699937611	5,830631263	0,200220497	0,575292096
NECAB3	-0,729854767	4,315113023	0,200389564	0,575292096
NOL10	0,718220086	5,777320782	0,200417917	0,575292096
SLC44A3	-0,675381132	3,108278515	0,200451983	0,575292096
PLEKHA3	-0,656905292	5,303368012	0,200492604	0,575292096
CLDN1	1,118547107	7,724909347	0,20061577	0,575292096
HUS1	-0,469116689	4,51483801	0,200677962	0,575292096
FAM46C	-0,80773831	6,548235633	0,200687316	0,575292096
EPB41L3	-0,600751463	5,277742474	0,200706833	0,575292096
BTRC	0,801455645	4,99769619	0,200809806	0,575345405
RAD9A	0,800223199	4,362635198	0,200911064	0,575393762
ZNF397	-0,628774142	3,679947568	0,201088966	0,575510567
EIF3B	0,62984334	7,097758704	0,201217942	0,575510567
TMEM133	-0,581503849	5,246946523	0,201243927	0,575510567
IKZF5	-0,661608972	4,593450833	0,201364838	0,575510567
TRIP12	0,574202556	6,817223259	0,20137384	0,575510567
INO80	-0,568375083	6,145508879	0,201736206	0,5760249
ARF1	-0,503943126	8,909697421	0,201777058	0,5760249
PTTG1IP	-0,519903673	6,515193296	0,201807228	0,5760249
NDUFA11	-0,693141078	6,35008337	0,202029716	0,576378568
MKKS	0,561972384	4,613370226	0,202100184	0,576378568
SNRPA	1,164028072	7,17653818	0,202384725	0,576670363
EXOC5	0,831497206	6,647282765	0,202418464	0,576670363

FHL2	0,95815846	6,465959018	0,202456203	0,576670363
RFX3	-0,631669828	4,670150442	0,202765977	0,577311564
C11orf84	0,857716357	4,264378669	0,202959819	0,577622289
PRKCSH	-0,665818895	7,518984287	0,203076419	0,577713018
MRPL42	0,632240576	5,442995344	0,203725314	0,57902928
KIAA1279	0,714743743	5,464566511	0,203774497	0,57902928
GPX1	-0,529657563	9,324750748	0,203861407	0,57902928
CBY1	-0,572486844	4,022383349	0,203878765	0,57902928
ATP6V1E1	-0,513236862	7,087621154	0,204070513	0,57910801
PDDC1	0,599134773	4,57031147	0,204076338	0,57910801
RPP14	0,800339848	4,219144388	0,20430022	0,5792126
MMADHC	0,784070904	7,0801869	0,204347913	0,5792126
BLOC1S1	-0,520291144	5,862577448	0,204368018	0,5792126
ZNF652	-0,492917257	5,409325206	0,204479477	0,579223615
DDOST	0,611059611	8,787903385	0,204633567	0,579223615
SMCR8	-0,657250997	5,984576512	0,204697806	0,579223615
BTN3A2	-0,648265092	5,55354054	0,204788562	0,579223615
GBP5	1,111352286	6,053700815	0,204796618	0,579223615
VPS26A	0,838762894	7,041546915	0,204942481	0,579395844
PIH1D1	0,85919707	4,960967601	0,205666644	0,58099036
CDIPT	-0,449024072	7,365202676	0,205682607	0,58099036
POLR2L	-0,516473655	9,275819507	0,205767791	0,58099036
PDHX	0,67402161	6,122898363	0,205847296	0,58099036
CD7	-0,589781091	4,553586052	0,205992776	0,581124712
AEBP1	-0,730949899	7,385343822	0,20606534	0,581124712
NDUFS6	0,85912122	6,921689357	0,206161373	0,58115519
FOXC1	1,149683525	4,741574264	0,206460938	0,581343873
MTA1	0,767974764	7,327818292	0,206524127	0,581343873
SNX21	0,84495907	6,02007594	0,206525545	0,581343873
MXRA5	1,077452898	7,300401111	0,206569322	0,581343873
ELAVL1	0,614955955	5,577877926	0,20674922	0,581540527
SBNO2	-0,564898154	5,942828493	0,206809764	0,581540527
MBNL1	0,690771124	6,336836595	0,206944345	0,581679096
AP2B1	-0,519223041	7,370065461	0,207296866	0,582190556
CLDN12	0,911313241	6,468053723	0,20736227	0,582190556
STK25	-0,638984666	6,183361807	0,207441113	0,582190556
FNIP2	-0,571603765	5,923043357	0,207467818	0,582190556
STAU1	0,733341486	4,985304005	0,208587277	0,585091172
IRS1	0,640414977	6,681932081	0,208715743	0,585210795
FKBP11	-0,709310609	5,090379719	0,209300661	0,586609622
UGCG	0,779847541	6,563444408	0,209486377	0,586888909
CYFIP1	-0,499526494	7,088306333	0,209618553	0,586944156
NAT10	0,54417326	6,253048207	0,209678247	0,586944156
LMNA	-0,536014876	8,449608648	0,20993656	0,587426099
CARKD	1,313325181	5,272269125	0,210215507	0,587582216
ANXA7	0,692841225	8,427542137	0,210236222	0,587582216
LOC728190	0,79206189	3,861707734	0,210328815	0,587582216
PKN2	-0,491482956	6,095691066	0,210337027	0,587582216
MAP1S	-0,480845833	5,85021783	0,210463432	0,587584148
AEBP2	0,617899927	7,197942311	0,210517057	0,587584148

NCBP2	0,588996622	6,305147361	0,210596225	0,587584148
NLN	0,827741193	4,465452126	0,21094418	0,588171241
MVD	-0,523307347	5,65041656	0,210979154	0,588171241
M6PR	-0,584570256	6,8671978	0,211142459	0,588208618
NSUN2	0,653877288	6,328396901	0,211165082	0,588208618
MRPS28	0,711104184	5,267666497	0,211322046	0,588405486
CYTIP	-0,723337752	6,300169929	0,211553504	0,588627133
XPNPEP3	-0,690402589	4,811451186	0,211597586	0,588627133
HGSNAT	-0,501820562	6,4553063	0,211660614	0,588627133
LAMA4	-0,717436211	6,473270182	0,211941152	0,58868243
DHX32	0,761091769	5,167732149	0,212260702	0,58868243
GNA15	0,977765965	5,68483004	0,212317004	0,58868243
NOTCH1	-0,603913048	7,631024714	0,212382007	0,58868243
GSTO1	-0,578832821	4,589328372	0,21248753	0,58868243
IKBIP	0,837829889	5,620559807	0,212493395	0,58868243
GPX7	0,987378278	4,316925495	0,212562233	0,58868243
PCNP	0,676682935	8,556431816	0,212772358	0,58868243
TRNT1	0,700105926	5,263199764	0,212773044	0,58868243
NLK	0,813578214	5,716453538	0,212899635	0,58868243
ZCRB1	0,979904451	6,095142924	0,212906347	0,58868243
PLXNA3	0,794373864	6,668101613	0,212910162	0,58868243
BLVRB	-0,545590609	6,359270492	0,212928077	0,58868243
IQGAP1	-0,502101362	7,416379409	0,212961018	0,58868243
DUSP14	0,833102162	5,123833494	0,212975444	0,58868243
NUDCD2	-0,454113349	6,151875538	0,213229502	0,588911729
COMMD3	0,657325608	6,469756326	0,213268433	0,588911729
C9orf3	-0,625293304	4,357472475	0,213435204	0,588911729
TMEM65	0,688157926	5,808092604	0,213475763	0,588911729
POLR1B	0,738201394	5,11265667	0,213490218	0,588911729
EBAG9	0,604661621	5,129853629	0,213885779	0,589460862
SETD5	0,676070058	6,478405488	0,213891331	0,589460862
NCDN	0,868435932	4,727984498	0,21401794	0,589460862
CIR1	0,577617389	6,655998437	0,214035063	0,589460862
TBCK	-0,505223856	5,523211111	0,214231748	0,589596864
C9orf78	0,516817608	7,369454592	0,214459118	0,589596864
TRIB1	-0,658794481	6,587140549	0,214585205	0,589596864
SF3B2	0,653459521	7,541023194	0,214617026	0,589596864
ABCF3	0,986070809	4,879204369	0,214659046	0,589596864
TAF4	0,541961321	4,657483532	0,214738818	0,589596864
ITGA6	1,260397848	7,981899173	0,214748834	0,589596864
UTP20	0,73821585	4,613581748	0,214776156	0,589596864
METTL3	-0,530029752	5,578776994	0,214990794	0,589948582
KIAA0284	1,010764252	4,684996608	0,215224292	0,590311767
ARID4B	-0,580751014	5,06925233	0,215342326	0,590311767
KIAA1704	-0,561198386	4,811902253	0,215382853	0,590311767
SMARCA2	-0,543893302	6,30008822	0,216029047	0,591844947
EXOSC8	0,656266375	6,702082411	0,216275887	0,591882854
UBL3	-0,499117991	6,994986376	0,216342878	0,591882854
RRP1	0,772481274	5,335613429	0,216430455	0,591882854
TCF7L1	-0,673759821	5,358428633	0,216430566	0,591882854

MEN1	0,711139333	4,995212685	0,216476879	0,591882854
KIAA1109	-0,536734274	7,029674002	0,216727229	0,592329851
RHOA	-0,527602704	9,013401159	0,217075338	0,593043561
MEMO1	0,540925246	6,801880163	0,21757737	0,594111467
CNNM3	-0,663458511	5,773202623	0,217892057	0,594111467
METAP2	0,768281773	7,429856899	0,217903648	0,594111467
GFPT2	0,900760014	4,58082299	0,217940738	0,594111467
DCAF16	0,868193779	6,26457415	0,218019845	0,594111467
BCR	1,06640786	6,858895107	0,218074767	0,594111467
MFAP1	0,622832212	5,761416201	0,218076111	0,594111467
KIAA0947	0,68144181	4,766191809	0,218394467	0,59471011
TSPAN5	0,9739838	5,626124354	0,218584014	0,59471011
MPHOSPH9	0,909124214	4,737612782	0,218769488	0,59471011
AKT2	1,057182471	6,20825976	0,218838304	0,59471011
PRCC	0,605592741	6,179985669	0,218845781	0,59471011
NR2F6	0,65561202	5,712104206	0,218920674	0,59471011
CREB3L2	-0,533596734	5,232613824	0,218947036	0,59471011
CARM1	0,618594835	6,30247942	0,21899356	0,59471011
SLC35D1	0,801616495	4,516122013	0,219258067	0,595191384
MLF2	0,535571286	6,849238687	0,219494895	0,595525403
SSR4	-0,567244146	7,416470536	0,219556457	0,595525403
CAPZB	-0,43938334	7,379986004	0,219800836	0,595525403
RBM5	-0,69563788	6,703210535	0,219819722	0,595525403
CCDC66	-0,474914251	4,100654075	0,219840954	0,595525403
RBBP7	0,636340703	6,340169819	0,219945933	0,595525403
PTPRG	-0,580626922	5,709031971	0,22001222	0,595525403
APC	-0,549642369	4,39168995	0,220387971	0,595525403
DPH5	0,663224863	4,80828656	0,220443447	0,595525403
TWSG1	-0,527300836	6,028128442	0,220454421	0,595525403
MIPEP	0,680919439	4,973174717	0,220536593	0,595525403
TACSTD2	1,447318797	9,337308816	0,220576654	0,595525403
HDLBP	0,60830354	8,72043631	0,22059717	0,595525403
TMEM30A	-0,505847994	6,931842144	0,220682325	0,595525403
MRPL14	0,705846942	7,056317167	0,220691112	0,595525403
ARRDC3	-0,552291069	7,268749985	0,221134151	0,596267953
NT5C	0,610155828	6,607520366	0,221141172	0,596267953
VAPB	0,659998976	5,764490757	0,221667201	0,596963703
MTMR2	0,563694531	5,931338107	0,221719102	0,596963703
MAN1A2	0,658831386	7,001058223	0,221860695	0,596963703
CDV3	0,57877757	8,261828416	0,222052624	0,596963703
MAD2L1BP	0,618343785	5,426873568	0,222129706	0,596963703
BCAR3	0,770612088	5,85133855	0,222184301	0,596963703
TAF2	0,704348579	5,523424878	0,222224969	0,596963703
MITD1	0,730761891	6,432030202	0,222254439	0,596963703
ANKRD17	0,881972077	7,163516271	0,22226462	0,596963703
BAX	-0,530305709	5,944684596	0,22227465	0,596963703
POLD4	0,676827947	8,599046347	0,222709864	0,597722349
FBXW9	0,834427725	4,223741659	0,222732437	0,597722349
IL7	-0,585818877	3,367962227	0,222901135	0,597939749
EIF2S2	0,723406397	6,188674185	0,223229222	0,598548044

P4HA2	0,688509678	6,81531202	0,22330345	0,598548044
SUCLG2	-0,520185647	5,881624508	0,223392776	0,598552197
PXK	-0,518904169	5,969109189	0,223528731	0,598681231
TFE3	0,789605701	8,43215276	0,223733657	0,598784808
CTNNB1	0,610498989	9,407970469	0,223743025	0,598784808
TNS3	-0,535261685	8,009032333	0,223859386	0,598811606
ITPK1	-0,708249181	5,660900896	0,223928669	0,598811606
STAMBP	0,602300414	5,964497988	0,224050009	0,598901219
PRDX6	0,539055447	8,900581365	0,22477707	0,600609263
THOC7	0,601302518	7,066197432	0,225014937	0,601009343
SOLH	0,653060781	4,939023631	0,225485797	0,601997695
LIN9	0,704501338	3,546703586	0,225561536	0,601997695
CLK4	0,621391855	4,116178915	0,225797529	0,602391765
TMEM134	0,698667904	5,628155061	0,226091417	0,602939919
POLR2J	0,653333125	6,419400588	0,226214717	0,603032899
TPCN2	0,714208336	4,688835484	0,226681958	0,603474101
PASK	1,073238631	4,354677538	0,226688291	0,603474101
POLR2B	0,617717677	7,103455915	0,226720086	0,603474101
KDM5B	0,583094784	6,585314655	0,226783262	0,603474101
NAP1L1	0,883710701	9,50273106	0,226822719	0,603474101
TMEM106A	-0,686436675	4,553312787	0,227088551	0,60394572
VOPP1	-0,534585668	7,27093384	0,227300192	0,604272907
RP9P	0,740194156	3,974805557	0,227407341	0,604322159
LONRF1	-0,625501516	5,658437583	0,22763598	0,604323628
DPY19L4	-0,582760722	3,914918224	0,227660131	0,604323628
LOC349196	-0,863955446	6,860282195	0,227775346	0,604323628
RBM10	-0,568222536	5,073363228	0,227826768	0,604323628
C1orf109	0,658775622	4,555258353	0,227954827	0,604323628
HIST2H2BE	0,627326399	9,269644785	0,227959656	0,604323628
TMEM59	-0,504618102	7,384220818	0,228028259	0,604323628
POP4	0,600607283	5,51556361	0,228330801	0,604605314
CLIP3	-0,610081735	5,401996753	0,228346474	0,604605314
C4orf27	-0,49860745	4,30828733	0,228400541	0,604605314
HDGFRP3	0,838633694	6,654574867	0,228915575	0,604681466
CCDC88C	-0,58042081	5,575920546	0,228947668	0,604681466
HIGD2A	-0,521119907	6,992031628	0,228950084	0,604681466
USP42	-0,48857549	4,698738193	0,228984786	0,604681466
SEP15	-0,58880386	6,063458157	0,229021887	0,604681466
RBM39	-0,47261321	8,152561039	0,229035862	0,604681466
LSM3	0,588678892	6,659399645	0,229050041	0,604681466
ZNF542	1,176442628	6,467904013	0,22924823	0,604803022
MMAB	-0,593490699	3,869657857	0,229273473	0,604803022
PLBD1	-0,70190569	5,487921802	0,229510868	0,60488074
PELI1	-0,626624829	5,608139467	0,229736729	0,60488074
SGPP1	-0,569159669	5,94966969	0,229769445	0,60488074
ACBD6	0,567788675	5,30802395	0,229794701	0,60488074
NFIC	-0,578489187	6,876102306	0,22982937	0,60488074
EFTUD1	0,65054529	5,122244239	0,229835166	0,60488074
DUSP6	-0,775948037	7,398345901	0,230010082	0,604977446
EDEM3	0,571285819	6,487451889	0,23004935	0,604977446

CNPY4	0,806708562	4,373377777	0,230317503	0,605232692
ANKRD11	0,789529643	6,668689858	0,230323924	0,605232692
IPO5	0,588314463	5,681153328	0,230567793	0,60564013
MSI2	0,699708023	5,729127486	0,230787054	0,605702091
SELK	-0,494639813	5,710407093	0,230972604	0,605702091
PPP4R2	0,571098145	7,04011519	0,230998039	0,605702091
CCDC47	0,593059546	6,583183474	0,231070076	0,605702091
RBMX	0,636718447	7,33219856	0,23110627	0,605702091
JAK3	0,745356222	5,494597194	0,231124335	0,605702091
PDS5A	0,609552544	7,798942591	0,231718985	0,606815344
DNAJA4	0,78643215	5,87847867	0,231756678	0,606815344
LSP1	-0,558821533	7,02929824	0,231816098	0,606815344
DHRS4	-0,574775301	5,003731057	0,232067576	0,607240522
SH3YL1	-0,645273706	6,060838463	0,232299592	0,607614468
C1S	-0,582752752	8,289526717	0,232467981	0,607821764
CCM2	0,676115706	6,64766591	0,232649546	0,60806334
ZC3H4	-0,500058576	5,03541714	0,232749188	0,608090695
LMNB2	0,925211326	5,458950018	0,232855258	0,608134815
WDR45L	0,586707132	6,041247293	0,232999265	0,608277943
TSN	0,629128279	6,872417304	0,233426594	0,609016751
ILK	-0,497542415	7,69270171	0,233528383	0,609016751
OLFML2A	-0,609037931	5,088559849	0,233550199	0,609016751
MICALL1	0,598337159	6,832877767	0,233754073	0,6091303
HNRNPA1L2	0,73817454	4,281784763	0,2337724	0,6091303
SLC4A7	0,769843465	5,526622577	0,23395604	0,609375949
BCL10	1,065152946	6,097989965	0,234395434	0,610275273
PIGK	0,604099305	5,653923687	0,234573342	0,610275273
GAL3ST4	-0,645463076	4,340838489	0,234580172	0,610275273
DSN1	0,57795794	5,754415639	0,2346593	0,610275273
TMEM98	-0,690235443	7,005145831	0,234766578	0,610321499
USP25	-0,486438903	6,729711215	0,235369385	0,610829768
BLMH	0,710891946	5,045576349	0,235419517	0,610829768
GALNT6	1,091410957	4,340703424	0,235431002	0,610829768
TRIM44	0,698074945	7,976406788	0,23554253	0,610829768
VDAC2	0,687487157	7,746011949	0,235659555	0,610829768
PGAP3	-0,558199882	6,081185901	0,235722788	0,610829768
HIPK3	-0,495375807	6,942497189	0,235743629	0,610829768
TMC6	-0,535045392	4,668205824	0,235840694	0,610829768
DICER1	-0,659273473	5,088616592	0,23589805	0,610829768
TLR4	-0,642425065	5,864607569	0,2359419	0,610829768
RGPD8	0,512754251	4,613147553	0,235947442	0,610829768
XRN2	-0,612459607	4,353871762	0,236307811	0,611159838
C17orf89	0,688127512	6,261508202	0,236353013	0,611159838
ZMYM6	-0,502051157	5,280526569	0,23638124	0,611159838
NUDT19	0,603405431	4,947498598	0,236509947	0,611159838
PTGIS	-0,65637725	5,177032927	0,23652307	0,611159838
ECSIT	0,687914951	5,002231626	0,236742276	0,611494539
HIP1R	-0,557386402	5,36714384	0,237042054	0,611637128
NUP188	0,541040276	6,316090185	0,2371202	0,611637128
RTTN	0,679277227	4,273182888	0,237122896	0,611637128

CD9	-0,702587995	8,934374774	0,237156264	0,611637128
HAUS2	0,676159747	5,337728558	0,237616575	0,612196084
ZDHHC24	-0,531611114	5,500801806	0,237764472	0,612196084
PPP2R2D	-0,58400319	3,687592252	0,237834311	0,612196084
FAS	-0,538453088	4,920466349	0,237938098	0,612196084
MAMDC4	0,807779733	3,368833042	0,237956769	0,612196084
SMAP2	-0,485723224	5,535682639	0,237984186	0,612196084
ARHGEF1	-0,573724999	4,045289673	0,238073756	0,612196084
EXOC4	0,581744419	5,166624041	0,238125785	0,612196084
ZNF337	0,826582005	5,533483089	0,238180996	0,612196084
JUP	-0,676542984	7,622271117	0,238297594	0,612264995
BBS5	-0,589124396	3,079911077	0,238531446	0,612558124
SAP30L	-0,567493461	5,43463114	0,23861442	0,612558124
ENPP4	-0,732120316	5,652418929	0,238681176	0,612558124
MATR3	0,56051997	8,494054484	0,238837476	0,612728649
ZFYVE16	-0,5710303	5,819470542	0,238927674	0,612729525
MRPL15	0,677046855	5,62270569	0,23940565	0,613724485
R3HDM2	-0,469514209	4,889850813	0,240205262	0,615125258
UBE2E3	0,697290564	5,66718052	0,240219568	0,615125258
WDFY3	-0,486106147	5,104120184	0,240222696	0,615125258
RPE	0,68574489	5,710095104	0,240403137	0,615181021
HDAC1	0,574410308	7,692738816	0,240435945	0,615181021
SNAPC1	0,53645233	4,620867233	0,240558853	0,615181021
THAP6	0,733659043	4,562790334	0,240605336	0,615181021
FMOD	-0,747970984	5,802884228	0,241040619	0,615829583
SHISA5	-0,429372259	8,922634208	0,241171413	0,615829583
ASNSD1	0,713688054	5,718164237	0,241211847	0,615829583
RABGAP1L	-0,569112727	5,774493852	0,24122024	0,615829583
RPL28	-0,539154642	10,7053141	0,24153877	0,616118553
IPO13	0,769324433	7,445583508	0,241550605	0,616118553
KBTBD7	0,693895924	4,115114264	0,241688421	0,616118553
HIST1H1C	-0,588270994	5,561565389	0,241694842	0,616118553
GLYR1	-0,504808945	5,744914429	0,241796483	0,616147315
ANXA2P2	0,79844246	5,043006261	0,242029634	0,616511048
PCBP1	-0,478472637	8,587236747	0,242141969	0,616566873
SMARCAL1	-0,489827669	4,158410718	0,242335831	0,616625684
PEBP1	-0,542651489	6,689668845	0,242400283	0,616625684
SKIL	0,601340934	7,331931178	0,242436348	0,616625684
TBCE	0,698907123	5,07048878	0,242781106	0,616962296
SEL1L3	-0,608124345	5,469496775	0,242793866	0,616962296
LYSMD1	0,982339845	5,147836766	0,2428626	0,616962296
PREP	-0,479208125	5,758708086	0,242930601	0,616962296
CPSF1	1,018019511	5,872831233	0,243111083	0,617091226
ANKRD36	-0,61590296	6,879448897	0,243238256	0,617091226
EPRS	0,570085373	6,922232453	0,243252855	0,617091226
TMEM127	-0,460528357	7,685199114	0,243461301	0,617193606
SSB	0,888147483	7,060247544	0,243474234	0,617193606
HDAC10	-0,581985408	4,865483641	0,243658478	0,617431126
PI4KB	0,571196747	6,183844683	0,24406481	0,618075777
ZNF280C	0,824363341	4,814610108	0,244174456	0,618075777

BSG	-0,474741655	8,295368234	0,244184799	0,618075777
IGFBP5	1,42185289	10,50333057	0,244618971	0,618875784
AGPS	-0,586050569	5,437900357	0,244729743	0,618875784
CDC42EP3	-0,591159401	6,944560016	0,244773132	0,618875784
BPGM	0,553459508	4,983330512	0,245001593	0,619223818
CNOT6L	-0,525927865	6,440914003	0,245444506	0,620068525
FKBP8	-0,762362318	6,105762344	0,245517674	0,620068525
CD63	-0,490490598	9,198423038	0,24601738	0,621100523
TUG1	0,68907684	7,919513096	0,246155544	0,62121934
PRPF19	0,678454575	7,849258747	0,246366675	0,621522145
DIDO1	-0,495256174	4,818025648	0,246543012	0,621588351
LIMA1	-0,452925895	6,809719299	0,24657523	0,621588351
SEC16A	0,57209169	7,661948623	0,246725224	0,621722696
USP4	-0,490451841	5,21092556	0,246810872	0,621722696
POLR1E	0,581971866	5,683845829	0,247067191	0,622138545
SAFB	0,513321906	5,841600319	0,247225981	0,622308587
C11orf83	0,837768295	4,455351528	0,247952273	0,623906477
PSMD13	0,553505835	4,963814405	0,24806373	0,623956687
AMMECR1L	-0,469887312	5,583122869	0,24820128	0,624072466
SHC1	0,580171237	8,779032228	0,248305127	0,62410345
USP28	0,570296501	5,539190753	0,248615453	0,624184453
LRRC58	0,534699432	6,117782554	0,24872994	0,624184453
THOC5	0,631419912	6,224685911	0,24888614	0,624184453
SEH1L	0,574649689	5,217808712	0,249142285	0,624184453
INTS8	0,683216458	6,752420402	0,249196461	0,624184453
HADHA	-0,408590238	7,036534728	0,249412815	0,624184453
TRIM22	-0,643710229	6,647812902	0,249538313	0,624184453
SMEK2	0,545561772	7,016201848	0,249566428	0,624184453
NPLOC4	0,603413022	7,691327365	0,249584278	0,624184453
PPA2	-0,537155581	5,202697725	0,249606905	0,624184453
S100A13	-0,491339589	5,027512004	0,24977235	0,624184453
FTL	-0,516400719	11,87165802	0,249876776	0,624184453
PPOX	0,628644203	4,117476239	0,249901603	0,624184453
ERF	-0,465629643	5,593988311	0,249944059	0,624184453
FXR2	-0,545613178	6,052540445	0,250000656	0,624184453
BRD9	0,644257498	6,35128352	0,250162211	0,624184453
HNRNPH3	0,624913357	6,661879124	0,250174363	0,624184453
SNX9	-0,538241128	3,897046731	0,250177208	0,624184453
ZUFSP	0,825568366	4,165275937	0,250228084	0,624184453
LRIG3	0,827875748	5,750276238	0,250230381	0,624184453
PSMA4	0,690313952	6,903769151	0,250259612	0,624184453
LRP10	-0,506226494	6,989942008	0,250588328	0,624511014
MRPL10	0,681541077	5,553334291	0,250606602	0,624511014
SERINC3	-0,461967519	6,241845736	0,250665295	0,624511014
MDFI	1,170848299	6,250045805	0,250911166	0,624895268
XRCC5	0,508727499	9,430665576	0,251418076	0,625929121
RHOBTB1	-0,578829715	4,457078409	0,251766109	0,626468524
PDF	0,812495178	3,94494283	0,251925024	0,626468524
HNRNPD	0,552792032	6,83262314	0,252063551	0,626468524
MTX3	-0,557049616	5,191920866	0,252117125	0,626468524

HIST1H2BK	0,637327247	6,925892756	0,252255787	0,626468524
TOR1A	-0,530852797	3,977810658	0,252310473	0,626468524
MLXIP	-0,474683017	5,345473529	0,25239989	0,626468524
SEC14L1	-0,48560102	8,326792112	0,252513428	0,626468524
CISD2	0,603411369	7,259677908	0,252542803	0,626468524
GCN1L1	0,578766256	6,078232261	0,25255345	0,626468524
SERPINF1	-0,552385744	7,665046228	0,252774548	0,62678896
SPIRE1	-0,482286927	4,77050603	0,252936962	0,626963702
PRDX2	0,67795313	7,640435429	0,253283282	0,627594005
ATF7	-0,464681704	7,616364888	0,253458866	0,627753757
MSC	1,438751128	5,688865455	0,253568806	0,627753757
CDC16	0,571571697	5,793644016	0,253767497	0,627753757
LRMP	0,664739422	4,527530939	0,253809134	0,627753757
HABP4	0,793808332	4,442094106	0,253854358	0,627753757
SMAD3	0,618472614	7,73336717	0,253900112	0,627753757
SUPT5H	0,916304139	7,972024072	0,254138016	0,628114219
SMUG1	0,592484628	5,147902719	0,254698329	0,629270979
WIPI1	-0,522393944	6,045562614	0,254847208	0,62941076
ORAOV1	0,721747506	4,46903616	0,25502682	0,629482699
AHCY	0,706686447	6,740792877	0,25507417	0,629482699
LOC100129034	-0,52422621	6,962426045	0,25517032	0,629482699
H3F3B	-0,436659469	10,94363232	0,255263869	0,629482699
SLC26A6	0,704790701	4,55362809	0,255337901	0,629482699
TRMT5	0,550674156	4,243140054	0,255601134	0,62970463
DNAJC14	0,54076566	5,378673654	0,255612614	0,62970463
TRERF1	0,700326923	5,599636698	0,255968168	0,630213468
CCL4	-0,66589233	6,573721167	0,256229983	0,630213468
AQR	-0,45591035	4,844574233	0,256466895	0,630213468
RCAN3	0,59998061	5,602401552	0,256472898	0,630213468
DNM1L	0,61472023	7,333128582	0,256574226	0,630213468
TOMM22	0,50952342	7,128259652	0,256654985	0,630213468
PRKRIP1	0,700032859	4,520774264	0,256909472	0,630213468
HIST1H4H	0,696089962	9,241496124	0,256947646	0,630213468
GPSM2	0,659237934	4,942834774	0,257046621	0,630213468
SF1	-0,714280427	6,068096481	0,257129119	0,630213468
B3GALNT1	-0,622332175	5,094577139	0,257274114	0,630213468
MAGEH1	-0,502920786	5,800040005	0,257472597	0,630213468
IFNGR2	0,678030616	7,4190404	0,257637871	0,630213468
DLD	0,661446409	6,631572136	0,257650543	0,630213468
PLCD1	-0,60781828	5,356277886	0,257698496	0,630213468
TNPO1	0,533234106	5,647865128	0,257849348	0,630213468
KCTD1	0,799832412	4,938628486	0,257858279	0,630213468
SLC30A1	0,54612025	6,073357676	0,257859631	0,630213468
MRPS2	0,593919013	6,647113457	0,257989994	0,630213468
OXSR1	0,614131439	5,195411938	0,257993853	0,630213468
PLEKHB2	-0,498140542	6,002341057	0,258032747	0,630213468
TDP1	0,641053575	4,180121381	0,258146082	0,630213468
BZW2	0,634925761	6,113685042	0,258265339	0,630213468
FAM86A	0,640517116	3,939375016	0,258430011	0,630213468
ZNF776	-0,48226273	3,471714375	0,258712182	0,630213468

PI4KAP2	1,46203468	8,839885594	0,258760085	0,630213468
PRPF6	-0,475156121	4,805481637	0,258781749	0,630213468
LOC282997	0,708088659	4,487446873	0,25879935	0,630213468
ATP6V1G1	-0,546277559	7,224445083	0,258894046	0,630213468
HSP90AA1	0,679682174	9,141688526	0,258997046	0,630213468
CMTM7	-0,550593256	6,346204072	0,259028401	0,630213468
CSDA	0,64340723	8,280695239	0,259055856	0,630213468
RNF41	-0,49397496	5,126340326	0,259173164	0,630213468
WIPI2	0,585098014	6,888866435	0,259199124	0,630213468
GTF2H2	-0,495096256	5,013552968	0,259303339	0,630213468
AHSA1	0,7086705	6,122034591	0,259385168	0,630213468
NFXL1	0,55791301	4,440575293	0,259531554	0,630213468
WDR34	0,548302012	5,557615232	0,25956187	0,630213468
SYMPK	0,623050691	7,183703133	0,259622152	0,630213468
RANBP10	-0,59696573	4,51177387	0,259754189	0,630213468
EPC2	0,694868298	6,056606947	0,259812441	0,630213468
RPL21	0,653702562	11,92145745	0,259831177	0,630213468
ITGA5	0,728360671	8,229628115	0,259832659	0,630213468
MFAP2	1,060401515	6,704491048	0,259885654	0,630213468
SRPRB	0,590209126	5,375103487	0,260334238	0,630784581
CNOT4	-0,457007986	5,510675453	0,260368478	0,630784581
RPRD1B	-0,41368731	5,537316334	0,26039868	0,630784581
UBE2N	0,528259634	7,097607238	0,26052755	0,630872643
FKBP5	0,8355317	6,932259807	0,260666082	0,630984031
AAAS	-0,525874211	4,716274638	0,260808593	0,630988
CLCN3	0,840906812	4,135656763	0,26085279	0,630988
SLC25A32	0,543287335	6,077590211	0,261107625	0,631020981
TBCC	-0,468191626	5,059933143	0,261220294	0,631020981
TIMP2	-0,517620885	8,968894747	0,261378774	0,631020981
SLC39A14	0,763986133	5,205051136	0,261500322	0,631020981
TJAP1	0,688888523	5,682093623	0,261539282	0,631020981
AMMECR1	0,627017854	4,534183232	0,261598486	0,631020981
QTRT1	0,731418849	5,942161469	0,261653873	0,631020981
DAB2	-0,558566102	7,733109191	0,261670304	0,631020981
BANF1	0,525308897	4,41047718	0,261699272	0,631020981
ILF3	0,799634806	7,593342772	0,262009135	0,631544818
ADRBK1	-0,439981799	6,627088205	0,262278214	0,631970014
MEIS1	-0,727832362	5,632020509	0,26274464	0,632784956
MLLT3	-0,420991867	4,50048326	0,262802023	0,632784956
COMMD2	-0,538623317	3,659735934	0,26294805	0,63291308
ALG13	-0,568764005	6,514737217	0,263408097	0,633501312
ENTPD4	-0,466113813	5,386800247	0,263417762	0,633501312
ST3GAL1	-0,542717971	4,758428305	0,263498693	0,633501312
CRTC1	0,842006319	5,943583716	0,263580223	0,633501312
DENND5A	-0,499910713	5,05625449	0,26377528	0,633501312
SNRPD2	0,63313223	7,22125973	0,263930478	0,633501312
GORAB	0,744936187	5,647570276	0,263949344	0,633501312
TATDN1	-0,478474214	6,558456356	0,263988639	0,633501312
UBE2J1	-0,437830597	7,110882794	0,264107921	0,633501312
HIST1H2AC	-0,682234951	4,66397962	0,264121459	0,633501312

NLRP1	-0,717529242	6,102347595	0,264299527	0,633705511
PDCD2	0,709207956	5,68715735	0,264634268	0,633904341
KIAA0513	-0,613676306	4,557729604	0,264667884	0,633904341
PFDN5	0,586884257	9,145703899	0,26471353	0,633904341
PSMD10	0,840873827	5,789481682	0,264754299	0,633904341
YARS	0,620054676	6,132461683	0,265272954	0,634923227
TTC30B	-0,489677161	3,728894365	0,26571626	0,635559792
ANKRD9	0,610283303	4,306927138	0,265725322	0,635559792
GPSM3	-0,498952781	5,730525153	0,265907886	0,635773448
TRAF3	0,562675566	6,804697731	0,266141532	0,636109044
COPB1	0,708027808	7,706409065	0,266247676	0,636139769
SUMO1P3	0,680870219	8,366623592	0,266468307	0,636224137
ZFP64	0,654934666	5,718088316	0,26646959	0,636224137
TAF1B	0,612548122	5,735113194	0,266608516	0,636333032
TTF2	0,635666362	4,773254166	0,266742543	0,636430161
EMD	-0,544635382	5,889915444	0,266927507	0,636648713
DDX11	0,818263757	4,232742274	0,267058238	0,636737806
CXorf56	0,503300743	4,922780366	0,267216002	0,636891267
TLE4	-0,552221762	5,570948027	0,267574153	0,637522065
DDX3X	-0,45635139	7,546326615	0,268054844	0,637767869
DPM1	0,563712641	7,439169449	0,268278012	0,637767869
RANBP6	0,547916739	5,299492327	0,26829381	0,637767869
CAPZA2	1,065254717	7,50751827	0,268296945	0,637767869
POLR3D	0,616388379	6,085493859	0,26831998	0,637767869
C20orf194	-0,608555695	5,314850735	0,268325631	0,637767869
MCL1	-0,475317516	8,231248041	0,268332016	0,637767869
TADA2B	0,597541923	6,035786728	0,268539106	0,637816708
MAPRE1	0,516052335	7,448530944	0,268539635	0,637816708
SNTA1	0,753094213	5,354341198	0,269468641	0,639723942
PIGG	-0,429953444	5,568904472	0,269615402	0,639723942
SQLE	0,870547952	4,681138335	0,269641491	0,639723942
QSER1	0,535035652	6,160698836	0,269798005	0,639723942
MYO1D	-0,562166212	6,996099379	0,26985907	0,639723942
USP3	0,503191336	5,929952633	0,269973123	0,639723942
YIPF6	0,538458645	6,405718788	0,270003096	0,639723942
GNAI1	-0,626491525	5,672585088	0,270093156	0,639723942
ACTR3	0,713603267	7,448590588	0,270359819	0,639835355
PELO	-0,518085099	6,127705879	0,270374666	0,639835355
ARL2BP	0,552328655	7,249437512	0,270700286	0,639835355
REXO4	0,661943365	4,499716957	0,270826902	0,639835355
ATG9A	-0,501305725	5,283325783	0,270843029	0,639835355
CHRAC1	0,56131949	6,346323211	0,270899292	0,639835355
DNAJC25	0,665136121	4,227895691	0,2709769	0,639835355
TMEM205	-0,551971092	6,224476394	0,271002513	0,639835355
PTPN11	0,589178624	6,87820589	0,271027634	0,639835355
RTN4	0,648552135	9,017261839	0,271078507	0,639835355
MIOS	0,541619658	5,360569345	0,271327705	0,640201944
EFHA1	0,678301145	5,696629443	0,271474801	0,640302451
MRPS10	0,717598547	5,930963149	0,271615631	0,640302451
TMEM209	0,564157355	5,64628623	0,271793963	0,640302451

NPRL3	-0,41855292	5,639803368	0,271833064	0,640302451
ZNF595	0,885526754	5,212178225	0,271931663	0,640302451
FUBP1	0,743709938	6,782038528	0,2719337	0,640302451
SLC39A9	-0,441538039	4,600823775	0,272400279	0,641179669
MSL1	0,493948249	6,520990633	0,272524374	0,641244628
TXNDC17	0,769775493	6,179185326	0,272615952	0,641244628
CCDC159	-0,626000134	5,445853244	0,273076409	0,642106218
LENG9	-0,485648601	8,295713468	0,273420652	0,642597206
ABTB1	-0,556062084	6,120208733	0,27347369	0,642597206
PHF6	0,649626283	6,675872972	0,273765508	0,643061315
KIAA0664	0,653788305	5,513505985	0,273862434	0,643067472
C22orf13	-0,50712768	6,648432192	0,274467745	0,644129618
ZNF197	0,682589005	4,586629828	0,274503691	0,644129618
MIB1	0,595506786	6,676365625	0,274786051	0,644309773
CIZ1	0,50859547	6,515031144	0,274843545	0,644309773
UBE2R2	0,5041992	7,11432057	0,27494358	0,644309773
DOCK1	-0,495514647	5,754013761	0,275077457	0,644309773
LOC100288778	0,958971674	5,057090787	0,275208208	0,644309773
VAMP7	0,659875555	5,367724904	0,27521367	0,644309773
DAZAP1	0,527294811	8,17283242	0,275241878	0,644309773
ADAR	0,537961103	8,380129029	0,275961595	0,64577286
LOC100216545	-0,557110215	5,661392252	0,276293092	0,646326792
CUL4A	1,092144242	7,192204311	0,276615346	0,646697537
JMJD8	-0,494475044	6,820509809	0,276641255	0,646697537
KLHDC3	0,783818102	8,570765702	0,277116675	0,647424134
SESTD1	-0,549428165	4,551899277	0,277141963	0,647424134
RNH1	-0,448345637	7,214800486	0,277530976	0,648110864
VPS41	0,452102177	5,942612955	0,277705886	0,648129197
LOC727896	-0,634785024	4,285365254	0,277728921	0,648129197
PKP4	0,640309262	5,392565941	0,277884006	0,648269258
NDUFAF3	-0,442867806	6,699464004	0,278029017	0,648385727
GSR	0,714113635	7,581894984	0,278386239	0,648996842
ZNF207	-0,457965221	7,550821239	0,27864892	0,649362989
ZMYND8	-0,479979162	4,811746702	0,278763759	0,649362989
CXorf40B	0,515959144	4,728773231	0,278828983	0,649362989
C9orf114	0,601976642	5,37436102	0,279016717	0,649578352
PUF60	0,636639292	5,804515884	0,279283047	0,649731238
PTPN1	-0,424565277	6,377477298	0,279368009	0,649731238
CSAD	1,080747526	5,252443355	0,279589966	0,649731238
CBR1	0,616640007	5,344528645	0,279596633	0,649731238
ATG10	0,592702941	4,0463476	0,279644505	0,649731238
PIGC	0,590975439	5,618169921	0,279654082	0,649731238
VPS13A	-0,45527435	4,857538971	0,280070876	0,65032842
ATP13A3	0,551770473	6,161617465	0,280120596	0,65032842
NFYA	0,817030568	6,387299765	0,280321301	0,65032842
ZC3H7A	-0,500202119	5,948255014	0,280452917	0,65032842
ACOT2	-0,53309626	4,792181314	0,28048338	0,65032842
RSL1D1	0,620503762	7,188518242	0,280606598	0,65032842
DSTN	-0,457613637	10,2766689	0,280648614	0,65032842
REEP5	-0,470927381	7,791811366	0,28069176	0,65032842

TNFAIP1	-0,537801728	6,808718966	0,280864818	0,65032842
RPS3A	0,60525584	11,16571241	0,280956328	0,65032842
GCFC1	0,660823544	5,120607385	0,280960189	0,65032842
SLC39A7	0,606600976	8,094751337	0,281411963	0,651153096
GPATCH2	-0,575751736	5,577296263	0,281592775	0,651350451
POMP	0,672114663	4,799445688	0,281876126	0,651470215
YES1	0,570905717	6,623674949	0,281891068	0,651470215
TMEM208	0,57203288	6,2434363	0,281931164	0,651470215
ZNF526	0,697682246	5,494407819	0,282160859	0,65159629
KCTD12	-0,56642792	8,617316011	0,282182535	0,65159629
COTL1	-0,501098147	7,620367151	0,282357187	0,65159629
RNF11	-0,492172935	7,505025116	0,282425321	0,65159629
CD14	-0,520659412	7,912029894	0,282463504	0,65159629
STARD3NL	0,52892321	5,9133419	0,282852444	0,652156046
ISOC2	0,570125409	6,227499646	0,282973242	0,652156046
SHROOM1	-0,52548109	5,455613613	0,28299307	0,652156046
CASK	0,63485075	7,954678092	0,283477461	0,652941047
COX10	0,554882108	6,146815211	0,283525215	0,652941047
AIM1	1,124179601	6,328141736	0,283760207	0,653261598
SMS	0,542762531	6,694426142	0,284160116	0,653592247
ATP5E	0,633232611	9,422459431	0,284189024	0,653592247
ATXN1L	-0,452100424	5,985882838	0,284191379	0,653592247
THYN1	-0,472870657	5,008495579	0,284621423	0,654296314
IMPA2	-0,583982746	5,784517132	0,284689421	0,654296314
FERMT2	-0,612746821	6,256537984	0,285007192	0,654805945
FYN	-0,583537149	4,920488813	0,285116148	0,65483564
MTF1	-0,484033993	4,050173548	0,28548898	0,655303436
GZF1	0,627783314	4,653203825	0,285512026	0,655303436
ASNA1	0,633741961	7,223721998	0,285770807	0,655368606
GUSBP3	-0,525762348	5,331285004	0,285786535	0,655368606
ERBB2	-0,533907776	4,787582374	0,285828748	0,655368606
PLCB3	0,553343305	4,842086869	0,285974065	0,655481396
LEF1	0,695412161	4,896540792	0,286320189	0,655878278
NUP214	-0,430276337	6,041015164	0,286339585	0,655878278
LOC440173	1,206940895	5,80768321	0,286712266	0,656511398
MTFMT	0,480112741	4,740749294	0,2870189	0,656992909
PARP8	-0,578322766	5,755019888	0,287313564	0,65744671
PLVAP	-0,586995885	5,294968631	0,287486479	0,657501444
CDC123	0,588161752	7,155542843	0,287530328	0,657501444
POLR2F	0,465435389	7,058762907	0,287728618	0,65773431
PRDM4	-0,436327612	5,317917915	0,288113388	0,658284051
RNPS1	0,575856318	7,197450595	0,288162178	0,658284051
USP9X	0,543803092	6,1412599	0,288628525	0,659091196
TERF2IP	-0,424748917	5,042422779	0,288708814	0,659091196
TFAM	0,739676064	5,819763157	0,288808582	0,6590983
SRD5A1	0,616359519	4,781708031	0,289027045	0,659376187
GNB2	-0,472132167	8,3624723	0,289407127	0,659386629
VPS72	0,533073844	5,743220352	0,28942003	0,659386629
RAP1A	0,722598905	8,3477005	0,289422261	0,659386629
UBD	1,314877088	6,372062167	0,289439053	0,659386629

TIGD2	-0,522090336	4,102990648	0,289531728	0,659386629
ELOVL1	-0,507333614	5,977372868	0,289761442	0,659386629
SUGT1	0,603888639	5,879305985	0,289776029	0,659386629
STON1	-0,594772771	5,053336966	0,28980521	0,659386629
CDK17	-0,518901213	6,348063472	0,289959769	0,659401971
ZNF331	-0,477298485	5,149044743	0,290059111	0,659401971
HSPA14	0,707316893	5,309463847	0,290260622	0,659401971
C9orf142	0,547592675	5,942599665	0,29037471	0,659401971
PHF8	0,651332292	5,112641416	0,29041178	0,659401971
NR1H2	-0,486476036	4,498438542	0,290453581	0,659401971
PPP1CC	0,737787809	8,433515638	0,290582362	0,659401971
NLRC5	-0,523821446	6,62782041	0,290629615	0,659401971
MAEA	-0,438673102	6,518062909	0,290838124	0,659401971
DR1	0,801246464	6,078855114	0,29094298	0,659401971
ECD	-0,469313727	6,230021978	0,290948529	0,659401971
PDE8A	-0,453356376	5,851940003	0,290972361	0,659401971
ANKRD13A	-0,424134672	6,034941271	0,291129273	0,659538376
WBP2	-0,451747276	5,722614607	0,29132341	0,659758995
TMEM126A	0,656041191	5,820149338	0,292278685	0,661488757
ZMYND19	0,664557484	4,621059023	0,292285143	0,661488757
SPTLC2	-0,426961218	6,309370781	0,292378225	0,661488757
LTV1	0,560731677	4,948300021	0,292475314	0,661488944
NDUFS5	0,541716632	8,019572304	0,292741115	0,661870579
HSPA6	0,755808389	5,936220899	0,293022063	0,662086254
ASF1A	0,562604271	5,495349113	0,293030696	0,662086254
VEGFA	0,622514908	8,053118707	0,293216484	0,662277636
CEP76	0,618898806	4,461201903	0,293309644	0,662277636
TSEN15	0,608122873	3,870801776	0,293570729	0,662583177
SORL1	-0,5892001	6,03155439	0,293639296	0,662583177
SNRPD1	0,740939786	7,727727185	0,293975809	0,66312307
INTS7	0,550082909	5,000152049	0,294200357	0,663355794
ANKRD27	0,681894636	4,410208783	0,294510982	0,663355794
ZFR	0,644843477	6,216128129	0,294616657	0,663355794
C16orf72	-0,551253712	4,679243116	0,294723507	0,663355794
PPP2R2A	0,541111787	6,374868725	0,294837731	0,663355794
SNRNP25	0,599974191	3,642442669	0,29484346	0,663355794
NOC4L	-0,515995582	3,855649717	0,294981291	0,663355794
THOC1	0,721628335	5,978180214	0,295058955	0,663355794
PDIK1L	1,0001905	3,907208337	0,295139475	0,663355794
ITGB5	-0,495285727	6,633233147	0,295235531	0,663355794
RGL2	-0,459826531	6,10186514	0,295374235	0,663355794
NINJ1	-0,518409269	5,607721717	0,295387346	0,663355794
NPR3	-0,575004726	5,039889868	0,295485613	0,663355794
PSMB4	0,462297182	8,325495663	0,295537656	0,663355794
RNASEK	-0,442955085	7,964932357	0,295538188	0,663355794
MRPL19	0,57290162	6,527612252	0,29596274	0,664088246
GPS1	0,466724324	7,808011897	0,296059285	0,664088246
MLL4	0,580649357	5,577715965	0,296307774	0,664427067
CIB1	-0,455285617	7,23411234	0,29671789	0,664606103
BMPR1A	-0,504372863	5,080177147	0,296747424	0,664606103

MORC2	0,591040379	6,523331004	0,29679656	0,664606103
EMB	0,883916142	5,675581303	0,296871007	0,664606103
UBQLN4	0,793514925	5,549947602	0,296959028	0,664606103
MGC57346	0,789323157	4,707949438	0,297040037	0,664606103
COL13A1	-0,611904886	5,324116318	0,297134595	0,664606103
ASAP2	0,565947812	6,584312632	0,297226002	0,664606103
PTPN2	0,655350257	6,298656863	0,297397949	0,664606103
DCLRE1B	0,704384858	5,547297495	0,29742391	0,664606103
SMARCB1	0,515827848	4,698358854	0,297459719	0,664606103
ZBTB2	0,651966317	4,442255716	0,297695121	0,664914192
PAFAH1B1	-0,379046596	5,612983749	0,297817267	0,664969202
UNC119	0,49799685	5,958259487	0,298030588	0,665009013
LGMN	-0,398393019	7,97593018	0,298051241	0,665009013
ANXA4	-0,501667386	7,371516417	0,298134065	0,665009013
STK16	-0,376882409	5,254333331	0,298225189	0,665009013
FASTKD5	0,540115398	5,692624918	0,29833103	0,665027556
C18orf8	-0,488262673	4,830101183	0,298462409	0,665102996
GTF3C4	0,5160351	6,05577193	0,298787853	0,665337687
POGZ	-0,439049933	7,349839249	0,298844601	0,665337687
ABHD2	-0,576338534	7,014307923	0,298860439	0,665337687
TMBIM4	-0,496113721	7,43718904	0,299014716	0,665463886
GTF2H5	-0,479153718	4,714113472	0,299548708	0,666163558
DCTN4	-0,423813929	6,065668743	0,299589018	0,666163558
ASB3	-0,442776079	4,76149162	0,299778571	0,666163558
GRINA	-0,449843055	7,640521851	0,29987458	0,666163558
MOSPD3	-0,433414654	5,768669943	0,299909133	0,666163558
KDM3B	-0,382182046	6,234735317	0,299915255	0,666163558
NAGA	-0,496963632	5,275591041	0,300167616	0,666506993
PDE12	0,563431909	5,520194797	0,300629834	0,667316029
ALKBH4	0,649891925	3,12568413	0,301026346	0,667456769
GAK	-0,379010654	6,071322333	0,301029368	0,667456769
TCEB2	0,562719753	9,669515877	0,301078408	0,667456769
MRPL32	0,502108432	6,78268756	0,301084766	0,667456769
WDR54	0,55300832	5,617863214	0,301250953	0,667608141
ATRX	0,519983336	6,522540693	0,301506652	0,667957719
SLC2A4RG	0,556527914	7,45141916	0,301986601	0,668803713
POP5	0,515553383	3,974070525	0,302231406	0,668985967
PLA2G12A	-0,511074492	5,86318824	0,302306657	0,668985967
CHD4	0,558103628	7,317255879	0,302363213	0,668985967
NKG7	-0,591272351	6,188722668	0,302601374	0,66929574
PMM1	-0,505676764	5,81701869	0,302840475	0,669607392
TMEM67	-0,482707291	3,350353212	0,303057657	0,669820729
CCDC85B	-0,434370586	6,527532273	0,303137583	0,669820729
C19orf43	-0,422901833	7,283898774	0,303231646	0,669820729
ABCC3	0,953469788	5,297265472	0,30336655	0,669901717
EIF2C3	0,701356578	6,882113379	0,30358999	0,670178097
GOLGB1	-0,495033128	6,25630708	0,303749693	0,670313642
IFT57	-0,414594647	6,053022826	0,304165415	0,671013901
TAF9B	0,601683078	6,410449305	0,304451031	0,671273086
ITM2B	-0,5291747	9,897746401	0,304479785	0,671273086

SGTA	0,507286021	6,439631367	0,304598419	0,671317589
CBWD2	0,4404884	5,678683722	0,304994369	0,671973055
SPRED2	-0,472940735	5,472895877	0,305493386	0,672855104
CCDC23	-0,472478292	4,171126999	0,305651075	0,672985044
ATAD3A	0,538541254	5,310949098	0,30623372	0,673824787
WDR82	0,437588272	7,112629497	0,306323761	0,673824787
SLC38A10	-0,4289726	5,937346819	0,30634096	0,673824787
C10orf47	0,856457655	5,628282001	0,306516161	0,673824787
ATL2	0,452682009	5,801210054	0,306526542	0,673824787
PKN1	-0,486318675	7,021711206	0,306692012	0,673971262
R3HDM1	0,423282943	6,410039891	0,306864533	0,674133134
HINFP	0,554512037	4,70813668	0,307435325	0,674844021
MRPL44	0,697212805	5,092931641	0,307481942	0,674844021
CCDC91	-0,42525549	5,213074103	0,307553326	0,674844021
ACSL4	0,79341541	7,678892265	0,307583988	0,674844021
PRKD3	0,517261967	6,576658749	0,307838163	0,675184442
CHFR	0,627336285	5,840902379	0,308276866	0,675929244
SLC1A4	0,800536282	5,838939511	0,308471489	0,676138567
TM4SF1	-0,532618108	8,051787254	0,308942163	0,676952638
LLPH	0,617767314	6,892208156	0,309173529	0,677177128
PRPS1	0,552288813	5,962917755	0,309243229	0,677177128
ATP9A	0,712039286	4,553179054	0,309485219	0,677313535
ZNF571	1,211147439	6,628682556	0,309504176	0,677313535
SPG7	0,745296278	5,882649868	0,309636598	0,677385936
NUP210	0,797860963	7,295349463	0,31002648	0,677644252
TOMM7	-0,512892072	3,432730453	0,310102769	0,677644252
C10orf88	0,54715898	3,432942273	0,310150525	0,677644252
ADNP	0,591389216	6,340235719	0,310252946	0,677644252
PTPN3	-0,472486924	4,803764214	0,310307369	0,677644252
FBXL3	-0,408644781	5,351198026	0,310350931	0,677644252
SMARCD1	-0,437941985	4,702697367	0,31049637	0,677744797
SAT1	-0,517222925	8,114129687	0,310640596	0,677842632
CNPY3	0,532985776	7,324862137	0,310949273	0,678299134
KCTD11	0,688806327	6,952374038	0,311098115	0,678406795
MRPS31	0,480589402	5,095268118	0,311373299	0,678789811
RNF167	-0,442902515	5,583662473	0,311603843	0,679075297
TPR	-0,431854809	6,675297052	0,312062875	0,679537137
LIG1	0,643617911	5,780848585	0,312101697	0,679537137
POLG	0,576010561	5,71109242	0,31213176	0,679537137
TRIP10	-0,473733161	6,178344227	0,312261907	0,679537137
RNF216	0,715032108	6,790907329	0,312314032	0,679537137
FADS1	-0,628193225	5,646111476	0,31251009	0,679695923
ARHGEF12	-0,413365518	8,390951162	0,312586364	0,679695923
ILKAP	0,432930655	5,244671809	0,312800294	0,67971463
TRAPPC2L	-0,392291156	6,65473904	0,312810003	0,67971463
LOC220729	0,605031108	3,514600507	0,312894005	0,67971463
FAU	0,50314857	10,31785891	0,313013831	0,679758381
GNPTAB	-0,426145493	5,757482775	0,313357645	0,680170399
CBR3	0,635141651	3,55392578	0,313403049	0,680170399
S100A10	0,616371076	8,230806829	0,313588647	0,68035666

ACTR2	0,456280651	9,185457816	0,313847245	0,680611473
COASY	0,473276972	5,872445274	0,313905717	0,680611473
SLC25A37	0,732358031	5,465330635	0,314014118	0,680630093
NCBP1	0,420352069	6,239220068	0,314290955	0,68063221
VPS25	-0,470312363	6,01493309	0,314417681	0,68063221
SDHAP2	-0,609717747	6,098047534	0,31446281	0,68063221
PRELID1	0,501236873	6,428266526	0,314480566	0,68063221
ZNF131	0,711592362	4,56413872	0,314514166	0,68063221
FOXK1	0,531195183	6,767741662	0,314754981	0,680761165
NIPBL	-0,393997358	7,42011801	0,314773421	0,680761165
KIF3B	0,543304772	5,700010264	0,315306281	0,680777909
ZBTB37	-0,504238657	4,222972367	0,315455758	0,680777909
ZNF142	0,673833118	5,023022515	0,315581537	0,680777909
PDE4B	-0,525608056	6,156686209	0,315709482	0,680777909
LMF2	0,533048004	7,340047071	0,315766298	0,680777909
TAGLN	-0,658478592	9,066744637	0,315827522	0,680777909
TMEM181	-0,439806123	5,614285617	0,315861249	0,680777909
ETHE1	-0,525808646	5,039857184	0,315896129	0,680777909
C17orf85	0,564421959	6,450269173	0,315920982	0,680777909
UBQLN1	0,458507655	7,343415853	0,315965642	0,680777909
ZNF823	-0,422534821	3,635072026	0,316029358	0,680777909
USP8	0,480174443	5,808364603	0,316051959	0,680777909
BATF	0,75785583	5,243285959	0,316119012	0,680777909
PRPF8	-0,353599948	7,372036391	0,316178859	0,680777909
SAP30BP	0,442947424	6,900400971	0,316595001	0,681458748
ATP6AP2	-0,437304836	7,050727107	0,316720602	0,681513974
SIPA1L1	0,654250381	5,758800327	0,316855003	0,681570559
KDM4B	-0,476390847	4,446492774	0,317007951	0,681570559
MRPL1	-0,538121189	3,869947095	0,317046754	0,681570559
SMARCD3	-0,55792637	5,08045442	0,317521734	0,681988184
REXO2	0,584113961	5,99935237	0,317529983	0,681988184
CTTNBP2NL	-0,527782992	5,640151867	0,317541059	0,681988184
SARNP	0,444723061	5,899683645	0,317797711	0,682301354
CD86	-0,51602364	4,59262147	0,317886993	0,682301354
SLC38A7	0,611992521	3,841440965	0,318210658	0,682781144
MRPL30	0,643786122	5,007584432	0,318456288	0,683093245
NANP	0,545525974	4,944309165	0,319084659	0,684225877
CRLF3	0,484657522	5,961285755	0,319286233	0,684442887
CDK3	0,750096931	4,591623259	0,319512457	0,684570795
MAT2A	-0,529897994	6,201208984	0,31960696	0,684570795
BCL2L1	0,573714109	7,452843481	0,319647076	0,684570795
MTA3	0,521787053	6,505323325	0,320141048	0,68541344
CAMLG	0,528973904	6,329887407	0,320283951	0,685504163
KANK2	-0,649321269	6,910382715	0,320401313	0,685540181
NANS	-0,464964195	5,293315621	0,320843354	0,686254506
RIT1	0,566265941	5,160819717	0,320936445	0,686254506
GRPEL1	0,557954313	5,302584831	0,321701289	0,687674322
PUM2	0,412403281	7,681564287	0,322063204	0,688232211
TAF10	0,551099449	6,859803338	0,322312668	0,688549524
AP1S2	0,534872429	7,51684285	0,322569568	0,68888252

GNAQ	-0,39353934	7,877664627	0,323033893	0,689658146
EIF4EBP3	-0,468510002	4,9355106	0,323340043	0,690015667
NCOA2	0,803823274	6,396637151	0,323509922	0,690015667
DNAJB12	-0,450645256	3,824637267	0,323527057	0,690015667
RBM15B	-0,403720939	5,956744673	0,323606116	0,690015667
PTPRF	-0,440571959	6,99722523	0,323978859	0,69059451
CAPN1	0,595781392	7,94367744	0,32411968	0,690678781
IFNGR1	-0,440189285	7,263041528	0,324526141	0,691137302
IPO8	-0,364055797	6,08616179	0,324634053	0,691137302
RPS6KB1	0,470092885	6,409628917	0,324638918	0,691137302
PTDSS1	0,544973907	6,486348043	0,325201997	0,69211998
PSMA2	0,49507889	6,631348295	0,325452321	0,692434034
BPHL	0,690561251	3,907060102	0,325779521	0,692434034
DCP1A	-0,364441602	6,021963187	0,325799911	0,692434034
LRRFIP2	0,45017079	6,520811577	0,325910824	0,692434034
SPSB1	-0,55149844	6,482875455	0,325920647	0,692434034
DNASE2	-0,558570256	6,423200198	0,325958828	0,692434034
DHX15	0,465976593	7,394986479	0,326096667	0,69251111
PSMD6	0,600698278	5,630361375	0,326390509	0,692919328
ARIH1	-0,384950873	5,651466904	0,326733011	0,69320824
RER1	-0,561372141	5,740152015	0,326865138	0,69320824
THOC6	0,474687761	4,563069563	0,326913346	0,69320824
TBC1D20	0,640460204	6,60965259	0,327012794	0,69320824
TMEM176B	-0,503760837	6,961205035	0,327034889	0,69320824
ATPAF1	0,503448656	5,59429042	0,327189723	0,693320921
ATG4A	0,577586844	3,732619186	0,327508409	0,693780628
TIMM9	0,587967554	6,428973356	0,327972205	0,694353181
PAPOLG	0,483255702	5,13371063	0,328078992	0,694353181
HTT	-0,401683625	6,375975766	0,328084169	0,694353181
SURF1	-0,388894439	4,681850025	0,328413577	0,694834683
RNF114	0,498589134	7,262462109	0,328585694	0,694983203
ATP5H	0,488147838	7,721665832	0,328745029	0,695066061
XPR1	0,494645543	5,593631512	0,328854718	0,695066061
JRKL	0,673300752	4,807302383	0,328930661	0,695066061
DNAJA3	0,483283559	4,823952185	0,329211963	0,695214684
BET1	0,547965153	6,771474951	0,329263334	0,695214684
ZADH2	0,878934883	6,622727841	0,329341946	0,695214684
COX16	-0,476102117	6,564657775	0,329408805	0,695214684
SACM1L	-0,382261132	6,589598872	0,329973358	0,696174992
MRPL48	0,675923226	4,634324427	0,330079079	0,696174992
HNMT	-0,493353138	6,923773357	0,330261232	0,696174992
C7orf26	-0,42642399	5,716515792	0,330336908	0,696174992
NCKIPSD	0,6710554	5,342467799	0,330401754	0,696174992
VPS4B	-0,472324364	4,814724056	0,330476382	0,696174992
HLTF	0,602152955	5,385508147	0,330690701	0,69641133
GAPVD1	-0,37584979	5,640245936	0,330907564	0,696652879
POLD3	0,458268242	5,306619821	0,331202699	0,696812512
KDELC2	0,760746203	6,115275096	0,331277339	0,696812512
PFN1	0,531369904	12,14398297	0,331289949	0,696812512
COL1A1	0,922691635	12,85970958	0,331833105	0,697634181

TDRD7	-0,471576174	3,717384812	0,331885215	0,697634181
MLLT10	-0,416458022	4,745479339	0,332053963	0,697773798
MARK4	0,610254525	4,979980293	0,332193228	0,697776898
CPSF3L	0,716006921	6,454275798	0,332260095	0,697776898
MTPAP	0,614370228	5,785976927	0,332977313	0,698854221
FAM168B	0,512385898	7,858747961	0,333166331	0,698854221
NID1	-0,621387429	6,482163552	0,333173464	0,698854221
ELP4	-0,386774406	3,72025246	0,333213232	0,698854221
COX18	0,487243606	5,046426263	0,333285515	0,698854221
P4HB	0,688359767	8,837397726	0,334114313	0,700376728
CLEC16A	-0,447138499	5,034117043	0,334367375	0,700467142
TPM1	0,716607124	8,997027418	0,334461893	0,700467142
BDP1	0,529313824	6,371208701	0,334465613	0,700467142
SF3A3	0,513233928	6,549021409	0,335169132	0,70153315
STK10	-0,446306741	4,978050463	0,335290563	0,70153315
TTYH2	-0,560081912	3,918781882	0,335478877	0,70153315
CHST14	-0,457771376	5,146079616	0,335482971	0,70153315
EWSR1	-0,375845022	8,250746367	0,335489016	0,70153315
PIGF	-0,418795406	5,318383116	0,335823092	0,701823501
CCNI	-0,385272172	8,69224294	0,335909599	0,701823501
PELI2	-0,538768185	5,706582987	0,335936634	0,701823501
TMEM185B	-0,48448067	5,012815141	0,336219825	0,701992768
TMEM19	0,534924978	5,515729784	0,336321621	0,701992768
TMEM110	0,498752594	5,470876118	0,33638525	0,701992768
COMMD10	-0,384993718	4,843433781	0,336467622	0,701992768
TSNAX	-0,44962548	4,467827581	0,336583019	0,701992768
RAB2B	0,474138839	5,904352202	0,336718734	0,701992768
CLN6	0,475599666	7,01297921	0,336738282	0,701992768
DDX42	0,489079794	6,37944971	0,337047358	0,702275907
SIAH2	0,864630855	6,476997067	0,337080323	0,702275907
TRAPPC9	-0,493212784	6,510556657	0,337364244	0,702275907
ZFYVE27	0,631096684	4,541429805	0,337559379	0,702275907
EIF4A2	0,518072095	9,203638119	0,337609421	0,702275907
RNF213	0,445797153	7,720140341	0,337620533	0,702275907
SH3BGRL3	0,678261361	10,80551644	0,33768138	0,702275907
KLRK1	-0,585606553	5,146304781	0,337698006	0,702275907
TSPYL2	-0,554447363	4,976568132	0,33786142	0,702401532
ZC3H7B	-0,463347064	6,083576307	0,33863907	0,703532331
ARF4	0,45103028	8,458034921	0,338712508	0,703532331
PTCD2	-0,449114953	4,701934722	0,338714862	0,703532331
ZNF414	-0,405888874	5,062341356	0,338849098	0,703550917
MYL12B	-0,474259613	8,237049502	0,339039745	0,703550917
CLIC4	-0,456023465	9,234326253	0,339107455	0,703550917
PARD3	-0,478425536	4,644709769	0,33913651	0,703550917
SLC25A30	-0,420952653	5,291647534	0,339493073	0,703777553
EIF4H	0,514250162	10,03753459	0,339634285	0,703777553
FIBIN	-0,622706502	5,334937958	0,33965938	0,703777553
C15orf61	-0,426934677	4,706741532	0,339760605	0,703777553
PAIP1	0,525161765	6,960569967	0,339761799	0,703777553
GGCX	-0,396937019	5,795538851	0,340146941	0,703906411

ANP32B	0,582765537	7,849126461	0,340201022	0,703906411
RAB8B	-0,468693026	6,169397201	0,340333078	0,703906411
HSD17B12	-0,37162301	5,737399254	0,340558492	0,703906411
TPT1	-0,437004897	11,56319267	0,340680009	0,703906411
UBB	-0,356617073	11,08713033	0,340770971	0,703906411
NSMCE1	-0,391652478	5,595831891	0,340783709	0,703906411
SRP68	0,399605843	5,671115452	0,341150996	0,703906411
RPS21	0,514488925	10,45975883	0,341392284	0,703906411
HELZ	-0,451548387	6,802369416	0,341507616	0,703906411
TBK1	0,46659646	6,230639991	0,341525698	0,703906411
SNX16	0,568649366	4,051204909	0,341615276	0,703906411
CCDC106	-0,39316672	4,991287892	0,341825367	0,703906411
VPRBP	-0,366826229	3,707310925	0,341966057	0,703906411
YWHAH	-0,361911797	8,552336665	0,341972438	0,703906411
CLSTN1	0,594847001	8,133632399	0,341983155	0,703906411
PARP1	0,474064503	7,524682309	0,342061246	0,703906411
SLC10A7	-0,387974462	5,268449446	0,342096897	0,703906411
SP140L	-0,457257287	5,500891182	0,342343992	0,703906411
RC3H1	-0,373104498	6,211089914	0,342354444	0,703906411
IMPDH2	0,505396679	7,758998464	0,342389309	0,703906411
PPP3CB	-0,49977899	4,656029194	0,342510888	0,703906411
RC3H2	0,443971952	5,983149039	0,342648501	0,703906411
ZNRF2	0,491209435	6,215784413	0,34281281	0,703906411
CDK10	-0,483700032	5,979755844	0,342833625	0,703906411
SF3A2	0,581795594	6,101426087	0,342896719	0,703906411
RRP1B	0,509100447	5,623167315	0,342908487	0,703906411
EIF4E2	0,570486517	5,736682923	0,342915135	0,703906411
OPTN	0,955365624	6,446792697	0,342948473	0,703906411
VTA1	-0,458611576	4,162594498	0,343034631	0,703906411
CCDC69	-0,50761266	5,428727918	0,343225284	0,703906411
STAG1	0,668828329	5,445742656	0,343239855	0,703906411
RB1	-0,419376523	6,07937747	0,343248761	0,703906411
MXI1	0,602254052	6,566290556	0,343333733	0,703906411
DCLRE1C	0,525550688	5,05808402	0,343784811	0,704619365
PTCD3	0,445262323	6,001868629	0,344227005	0,705313687
UCK1	-0,381898871	4,600357936	0,344744077	0,706160968
FLOT2	-0,431414704	6,602005396	0,345000001	0,706376123
SCO1	0,576119353	5,127795493	0,345086221	0,706376123
SLC38A2	0,482519543	8,624559047	0,345159883	0,706376123
IRF3	0,443048787	6,854388589	0,345427363	0,706711427
RPL4	0,518102991	11,32845186	0,345952772	0,7074453
PATL1	0,513463207	6,13961893	0,345993558	0,7074453
AGAP5	-0,461972425	3,596049629	0,34613692	0,707526276
ECHS1	-0,333229449	6,071571525	0,346523243	0,707537149
STARD10	-0,513667325	5,504353933	0,346624423	0,707537149
RPS23	0,484736832	11,40697039	0,346801515	0,707537149
NUP153	0,539950433	6,860720536	0,346873545	0,707537149
C19orf66	-0,473816255	5,317193629	0,346958646	0,707537149
FBXO21	-0,416704881	4,901166757	0,346996137	0,707537149
IVNS1ABP	0,525135239	7,331034378	0,347085252	0,707537149

C10orf118	0,542441908	6,25996217	0,347172293	0,707537149
NEK11	0,62600289	3,919009168	0,347188535	0,707537149
NFIB	-0,501482275	5,165775969	0,347467529	0,707537149
DNAJA1	0,416676103	8,672823161	0,347562595	0,707537149
VPS29	0,432807903	7,056211915	0,347665695	0,707537149
ST8SIA4	0,599833885	4,595053629	0,347738748	0,707537149
ACAP3	0,732557394	4,951731593	0,347867576	0,707537149
EIF2C1	-0,432876016	5,026401269	0,347881153	0,707537149
MAP7D1	0,382704358	6,726231685	0,347882205	0,707537149
MAGOH	0,528385806	5,688299393	0,347906153	0,707537149
ORMDL2	0,536063419	6,101677492	0,348240354	0,707648345
MTCH2	0,41016691	6,332111638	0,348243066	0,707648345
CSK	0,461161299	6,247577997	0,348272158	0,707648345
PIGX	0,587665691	5,405736356	0,348708624	0,708324131
TTC31	-0,418566735	4,491293697	0,348828327	0,708356273
COPS5	0,533702269	5,033207031	0,349039201	0,708573477
PI4KAP1	0,843732548	7,01642153	0,349324238	0,708625668
TARDBP	0,416981564	7,749963955	0,34946187	0,708625668
FCF1	0,513723371	7,300425568	0,349622171	0,708625668
IWS1	0,439225506	6,331031551	0,349628469	0,708625668
PDHA1	0,419597842	6,670510859	0,349683334	0,708625668
CCDC9	-0,465143948	5,622849216	0,349823479	0,708625668
CDK8	0,618124132	5,596641799	0,349853295	0,708625668
PIK3CB	0,625916499	6,307646359	0,349896264	0,708625668
DDX47	0,488072857	5,450209611	0,350463831	0,70896537
NDUFA1	0,450324552	8,492348254	0,350499763	0,70896537
NR1D2	0,56787434	5,336936777	0,350593307	0,70896537
GATAD1	0,485640291	6,122137666	0,350680526	0,70896537
MIER3	-0,385090854	4,381076754	0,350698674	0,70896537
SSR3	0,538211646	7,890149397	0,350786358	0,70896537
SSPN	-0,460139923	4,705226806	0,350791781	0,70896537
SRRT	0,5778705	4,912129005	0,351076833	0,70914973
MRPS24	0,528817651	6,073427537	0,351090994	0,70914973
NBEA	-0,645751025	4,887936516	0,351298454	0,709183436
LDOC1	0,794691235	8,595904607	0,351315683	0,709183436
FBRS	-0,44211923	5,889294795	0,351515323	0,709289018
ZNF805	-0,377667	4,865404979	0,35157602	0,709289018
CXADR	-0,458670094	5,484831131	0,352143946	0,710208729
ANKRD52	0,457522603	5,835675409	0,352279405	0,710208729
PEX12	0,622553374	3,614376524	0,35243395	0,710208729
SRGAP1	-0,463829334	4,378414825	0,352505112	0,710208729
ETV1	-0,541639566	5,174352275	0,352552654	0,710208729
C16orf80	-0,345523562	3,504046554	0,352736888	0,710370006
NUDT9	-0,357189881	5,213665055	0,352904351	0,710497422
MFSD7	-0,45207861	4,037843169	0,353084526	0,710650348
DDT	0,451613235	6,777691878	0,3534771	0,7111142
GLRX3	0,51872758	6,176786935	0,353523557	0,7111142
PAPOLA	0,455269734	6,124166215	0,353935558	0,711732991
PNMA1	0,559119796	4,720641036	0,354200267	0,711893261
LYN	-0,436453304	5,438841765	0,354387086	0,711893261

DOCK10	-0,418572769	4,715188989	0,354411538	0,711893261
UBR3	-0,40836715	4,711643773	0,354432853	0,711893261
MYSM1	-0,454155859	5,170024247	0,354861972	0,712448242
PSME4	0,401870608	6,518888238	0,35503674	0,712448242
SMEK1	0,493852871	6,012924739	0,355563329	0,712448242
PRPF4B	0,639282232	7,114833472	0,355606061	0,712448242
TMEM99	0,624305682	3,927809872	0,355617322	0,712448242
SLTM	-0,342864022	6,764292073	0,355651556	0,712448242
RPL36AL	-0,474900285	7,160195781	0,355668639	0,712448242
DHRS7	-0,428670242	4,145865233	0,35593247	0,712448242
SPNS1	-0,544150749	4,842099041	0,356086657	0,712448242
OSBPL5	-0,482878268	5,693601994	0,356096519	0,712448242
MET	1,157500145	5,904160842	0,356266035	0,712448242
RCN3	0,848781308	6,015295659	0,356292849	0,712448242
SOS1	-0,396680011	5,445135889	0,356380135	0,712448242
AACS	0,458769838	4,975265086	0,356389195	0,712448242
SNTB2	0,481181759	6,789642651	0,35651733	0,712448242
GBAS	-0,392837779	5,703017619	0,356588318	0,712448242
CWC27	0,436234154	6,589041577	0,356632024	0,712448242
C1orf43	0,454940627	8,047687091	0,356743521	0,712448242
U2AF1	0,390274004	6,940121375	0,35678554	0,712448242
ISCA1	0,551757419	4,474888407	0,357023185	0,712448242
COIL	-0,411556945	4,535617435	0,357052621	0,712448242
ANXA2	0,591106311	10,71094495	0,357080824	0,712448242
SETMAR	0,764397741	4,5520587	0,357134632	0,712448242
WBP5	0,580487723	5,10698043	0,35721668	0,712448242
SERPINH1	0,61311504	9,206394275	0,357328222	0,712462322
SUPT4H1	0,443035756	7,507721803	0,358079269	0,713601916
C12orf57	-0,427603799	7,328933047	0,358109071	0,713601916
HARBI1	0,535061529	3,804663449	0,358393947	0,713842184
MSRB2	-0,370098586	5,412169722	0,358439014	0,713842184
HMG20A	-0,37164813	5,474232974	0,359008943	0,714768463
DGKD	0,631108368	4,986664622	0,359159292	0,714859081
ZFX	-0,345020833	6,52332663	0,359594089	0,715515639
JOSD1	0,558688713	4,696814948	0,359733679	0,715584585
PPP2R5C	0,510665437	6,426991552	0,360235434	0,7163568
SEC31A	0,468662945	9,058855846	0,360331987	0,7163568
TIMP1	0,793788285	10,81514515	0,360531315	0,716491834
RFC2	0,538034432	5,501119656	0,360610056	0,716491834
IFRD2	0,523626974	5,711283627	0,36093282	0,716924234
SMURF2	0,684767207	5,003260872	0,361233519	0,71695826
ABHD4	-0,371824915	4,725777453	0,361247653	0,71695826
RUNX3	0,628842003	5,395389773	0,361282902	0,71695826
HECTD2	-0,426021705	4,544107215	0,361570331	0,71695826
CAPNS1	-0,396730145	7,393280438	0,361571405	0,71695826
NOL9	0,526153875	7,235067176	0,361648307	0,71695826
AP1M1	0,48816544	7,071320312	0,361815633	0,71695826
MSH3	0,565637842	3,648354106	0,362022308	0,71695826
HDAC4	0,484943023	5,470885402	0,362041016	0,71695826
CPD	0,558379905	7,022004436	0,362114631	0,71695826

NDE1	0,464128185	5,893428441	0,362209716	0,71695826
CCDC124	0,562108735	6,682317215	0,362211645	0,71695826
RBAK	0,566570144	5,122215005	0,362550758	0,717421247
DENND1A	-0,418355041	6,057265052	0,362710594	0,717529313
TXLNG	0,495087125	4,872779359	0,362965894	0,717619084
GSS	0,455120523	5,677196313	0,36296645	0,717619084
CD58	-0,373929626	5,656498496	0,363292048	0,71805463
RECQL	0,802332694	5,476919222	0,363804417	0,718858974
FBXL12	0,388144061	6,332541179	0,364378295	0,719784354
MFSD1	-0,477547396	6,039093415	0,364750889	0,720311703
PPP1R11	-0,357100231	6,668668334	0,3651552	0,72072385
H2AFY2	0,78509395	4,532925037	0,365170978	0,72072385
C2orf68	0,537598511	4,69117063	0,365358458	0,720773449
MBD1	0,46414007	5,755998082	0,36540751	0,720773449
ULK2	-0,473326476	5,313369072	0,36553048	0,720807503
FAM120A	0,435226145	7,940542726	0,365725052	0,720829564
FZD1	0,646681893	7,449399917	0,365753086	0,720829564
SOAT1	0,540413157	7,274542433	0,366070942	0,721247544
IRF1	-0,441135409	6,640823185	0,366290389	0,721282204
EGFR	0,679121435	7,23790269	0,366300084	0,721282204
SYPL1	0,55121624	7,703754948	0,366442752	0,721354828
PCNX	0,442378634	6,920089927	0,366686156	0,721625655
ZNF614	0,518707285	3,525577645	0,366906166	0,721679392
GAS2L1	0,685012017	5,716669416	0,367076387	0,721679392
PSMC5	0,427459257	7,117264324	0,367125357	0,721679392
AKIRIN2	-0,363434606	5,993522555	0,367136796	0,721679392
TIMM10	0,471559572	6,280946902	0,367454372	0,721756764
RAB5A	0,500843498	6,963356941	0,3674874	0,721756764
LIG3	0,536568531	4,979474171	0,367493692	0,721756764
SPPL2B	0,611170338	6,249511674	0,367671806	0,721811598
TPI1	0,654249322	8,333259497	0,367733318	0,721811598
OXR1	-0,385687535	4,340892087	0,368050501	0,721831961
RAB12	0,459232529	7,516920032	0,368058575	0,721831961
KIAA1430	0,427943827	6,369597328	0,368237761	0,721831961
RUNDC1	0,495021307	5,735598529	0,368256278	0,721831961
ZNF224	0,790431422	6,998144087	0,368397596	0,721831961
ING5	-0,515804475	4,421246666	0,36847284	0,721831961
RALB	0,695795993	6,745476719	0,368484683	0,721831961
CHMP5	0,529952972	7,179848619	0,368691233	0,721880485
ATP5F1	0,501202788	6,823765746	0,36872118	0,721880485
TMEM164	-0,42977013	5,82652078	0,368872531	0,721969515
SNX1	-0,350097372	6,39357227	0,369178061	0,722360172
TREX1	0,652968214	4,212982484	0,369467695	0,722399497
RRBP1	0,695611279	8,047786948	0,369483448	0,722399497
FAM40A	-0,356331816	5,256846759	0,369742478	0,722399497
SFXN3	-0,399714445	5,935599309	0,369750882	0,722399497
ITGB3BP	0,652021485	4,49062288	0,369830334	0,722399497
ABHD5	-0,34065099	5,413806395	0,369833794	0,722399497
ZNF485	0,815754209	4,693242009	0,369971655	0,722461831
SLC20A2	-0,431391571	4,384261441	0,370155838	0,722614561

RHOQ	0,47358349	6,841788095	0,370265268	0,722621312
STARD4	-0,398356397	4,404830519	0,370390962	0,722659791
GRIPAP1	0,616844359	4,073381423	0,370571357	0,722668389
NAPEPLD	0,451840967	4,483612781	0,370709645	0,722668389
ADAM17	-0,365284484	5,829451263	0,37084143	0,722668389
ZNF462	-0,479604835	4,961353087	0,370873817	0,722668389
STAG2	-0,420390822	4,206786211	0,370925262	0,722668389
TXLNA	0,427534886	6,957533109	0,371260168	0,723114277
KCTD3	0,459601726	6,471633193	0,371509189	0,723392677
COX11	0,691063575	4,719819946	0,371669088	0,723497435
SPPL3	-0,429531661	6,475286166	0,371838129	0,723619921
RARS2	-0,362633127	5,512629057	0,372146173	0,724012769
SNX6	0,644896537	6,762152303	0,37249545	0,724485588
VPS45	0,576795182	5,535872232	0,372637757	0,724555707
DYNC2H1	-0,470224238	5,316403887	0,372824216	0,72471161
ATP6V0E1	-0,434688961	8,343276519	0,373051207	0,724821162
PAF1	0,671180559	7,956534546	0,373093975	0,724821162
HEATR2	0,410536877	6,422445857	0,373363473	0,724821162
REPS1	-0,362714361	5,602357146	0,373385956	0,724821162
RFX1	-0,409522467	5,427488899	0,373412046	0,724821162
GIGYF2	-0,377328525	6,118997162	0,373629072	0,72503604
ARL5B	0,549034467	5,895847198	0,373992039	0,725533915
RARRES1	-0,428676574	5,804454249	0,374596272	0,726212137
MAGT1	-0,379774102	5,651278098	0,374652013	0,726212137
ATP5I	0,43478698	7,925239546	0,374745387	0,726212137
TMEM219	-0,362120142	8,119097299	0,374784071	0,726212137
STAT5B	-0,352415343	6,191944886	0,375107048	0,726212137
ACAT1	-0,383674621	6,023883333	0,375158757	0,726212137
C1GALT1	0,614137399	5,466453608	0,375301055	0,726212137
PRMT1	0,445812294	8,074826293	0,375426305	0,726212137
PPFIBP1	-0,398322328	5,672760757	0,375554751	0,726212137
C1orf54	-0,473266795	5,481118856	0,375813259	0,726212137
TRAK1	-0,503434741	6,368082107	0,375832468	0,726212137
MRPL37	0,485216935	6,53143798	0,375836768	0,726212137
RANBP2	0,405195344	6,899882309	0,375900194	0,726212137
ZBTB25	-0,368934049	4,165126229	0,375951227	0,726212137
SBDS	0,440070196	7,971101307	0,376099085	0,726212137
KLHL5	0,463288668	6,049755231	0,37620215	0,726212137
NAA16	0,534138735	4,293349606	0,376238117	0,726212137
CEP57	0,765993768	5,114308081	0,376258613	0,726212137
PSMB1	0,413277958	7,631039674	0,376720425	0,726695721
TMEM45A	0,60240735	5,130082042	0,376722302	0,726695721
ACOT11	0,630972481	3,765540662	0,37725028	0,727001133
SLC7A6	-0,418783261	3,554661856	0,377274174	0,727001133
CCNG2	0,629118081	6,208165993	0,377285903	0,727001133
PIAS2	-0,404868326	6,033822242	0,377307084	0,727001133
ZCCHC24	-0,491991318	6,788669679	0,377433722	0,727039703
LARP4	0,442870356	5,540205616	0,377843297	0,727490796
NPHP3	-0,471320075	5,253596142	0,37796192	0,727490796
HAX1	0,445643601	7,05467288	0,377987959	0,727490796

MGA	-0,413221195	5,3539066	0,378145235	0,727588138
TMEM2	-0,459904662	5,154561646	0,37845261	0,727974145
ARHGEF10L	0,571757539	5,518068468	0,37880332	0,728431755
XYLT2	0,601948311	6,170120493	0,37900304	0,728431755
RAB7A	0,416278498	8,380862495	0,379010979	0,728431755
LPIN3	-0,438603744	4,781107549	0,37922891	0,728645234
NISCH	-0,423956837	5,560361197	0,379517285	0,728993906
C10orf12	0,548335286	4,500183966	0,379658188	0,72905919
WRN	-0,358108876	5,358999625	0,37998678	0,729484755
TBC1D9B	-0,39920914	6,763848825	0,380980274	0,730709453
NPEPL1	-0,422372001	6,231630185	0,381008385	0,730709453
ZCCHC11	0,495033405	5,4838687	0,381046925	0,730709453
AGR2	-0,632883381	7,714400253	0,381297197	0,730709453
TRMT1	0,659659552	5,905672009	0,381418133	0,730709453
DDX10	0,611473682	5,537045568	0,381491267	0,730709453
OTUB1	-0,311202652	6,14087943	0,38151597	0,730709453
LEMD3	0,519968722	5,587520695	0,381524836	0,730709453
SIRT6	-0,387889503	4,29262194	0,3816159	0,730709453
LIN37	0,443688977	5,653639704	0,381770114	0,730709453
TOP2B	0,507397313	6,703021239	0,38181278	0,730709453
PYGO2	0,543854579	5,156524614	0,382005083	0,730709453
OSMR	0,649026836	7,382574966	0,382078256	0,730709453
CLPX	-0,338473761	5,574178893	0,382124932	0,730709453
TECPR2	0,471031655	4,886003476	0,382947644	0,732077372
CDK2AP1	0,48901292	8,181405605	0,383121824	0,732162858
CDC42SE2	-0,410417097	6,014626674	0,383287835	0,732162858
DDX60L	-0,401957834	4,586921665	0,383314475	0,732162858
DUSP18	-0,386724405	5,013029902	0,383510614	0,732332365
MYL12A	-0,449162271	8,326258387	0,383786596	0,732654199
C6orf226	0,493820827	3,984745986	0,384242272	0,732828201
SMURF1	0,499168097	7,061335012	0,384271587	0,732828201
C22orf32	-0,356853741	5,329427375	0,384333732	0,732828201
GTF2I	0,441925081	6,251850307	0,384389659	0,732828201
C11orf74	0,578192457	4,663150271	0,384415086	0,732828201
GMIP	-0,45690375	4,324589328	0,384740262	0,73324311
B3GALT6	0,544626142	7,184787673	0,384997835	0,733516761
C2orf29	0,407200442	6,845460289	0,385159833	0,733516761
ARHGEF7	-0,418688094	4,631862247	0,385349187	0,733516761
ICK	0,440941691	5,035252814	0,385377031	0,733516761
MED15	-0,349942712	8,07664882	0,385421697	0,733516761
NBN	0,464452744	6,150927108	0,385540308	0,733537767
NAF1	-0,370003098	4,126200605	0,385847303	0,733917087
THAP11	-0,39285359	5,083503762	0,386411081	0,734784485
RAB32	0,533061736	5,619700109	0,386747431	0,734958088
YWHAQ	0,481401581	9,391035745	0,386799989	0,734958088
OSBPL2	0,486426869	6,75756871	0,386977988	0,734958088
LOC253039	0,629323922	3,690887398	0,386981518	0,734958088
TOR1AIP1	-0,330062235	6,588727976	0,387120809	0,734958088
ATG4B	-0,394633433	6,453576004	0,387149062	0,734958088
MYOF	0,573543466	8,297349621	0,387296169	0,735032723

UQCRQ	-0,431700644	7,62444131	0,387887702	0,735857209
SLC26A2	-0,487068552	5,011773358	0,387946424	0,735857209
ARHGAP33	0,826593426	5,636654327	0,388123052	0,735987511
ARMC8	0,672508219	7,404328318	0,388479213	0,736276126
XPO1	0,603073049	6,266488243	0,388491201	0,736276126
GOLT1B	0,488036361	6,50789121	0,388684772	0,736319889
PSMD8	0,493580697	9,275765954	0,388779032	0,736319889
MRPL54	-0,402520356	5,267111298	0,388907025	0,736319889
MAN2A2	-0,437393016	6,91203859	0,389124083	0,736319889
LRPAP1	-0,370186455	6,646069512	0,389159401	0,736319889
ELK4	-0,325162215	6,794296471	0,389162176	0,736319889
CDH1	-0,521949038	5,947506625	0,389434344	0,736630455
PRKRIR	0,586734259	6,586248791	0,390000072	0,737495977
ALG6	0,530381627	4,9474434	0,390168386	0,73760971
AP4B1	0,581831004	4,481989947	0,390455757	0,737948395
C3orf52	-0,453653758	3,748679806	0,390807317	0,738408173
FTSJ2	0,456400831	4,386200016	0,391510202	0,739531321
RPL5	0,436870364	10,50042619	0,391823199	0,73984149
YEATS4	0,594535862	4,647315024	0,392011424	0,73984149
FLII	0,445052504	7,18738878	0,392046063	0,73984149
AGGF1	0,437993599	5,646487165	0,392214932	0,73984149
NUDT4	-0,314334853	6,673084213	0,392253546	0,73984149
SLC25A24	-0,36581282	4,835586422	0,392325389	0,73984149
PTOV1	0,496384152	6,526067314	0,392712114	0,740366023
MTOR	0,431262185	5,570105872	0,393093291	0,74087981
NAT14	0,444837857	5,473181049	0,393367122	0,740890498
ZYX	-0,349932522	9,882768516	0,393427111	0,740890498
TAB3	0,542333124	6,306137121	0,393604431	0,740890498
PDK1	0,587525317	3,496551196	0,393873016	0,740890498
OSBPL8	0,440109848	7,833403718	0,393905385	0,740890498
TMEM41A	0,545448067	6,215903648	0,393907004	0,740890498
DLG1	0,553479865	5,493322916	0,394016505	0,740890498
HCLS1	-0,398817407	5,911261507	0,394085314	0,740890498
LOC650623	-0,536796016	4,645617153	0,394164537	0,740890498
C18orf21	0,502894251	5,743091079	0,394185471	0,740890498
SAP18	-0,358083996	7,755751504	0,394361892	0,741017839
ZC3H6	0,584030731	4,38407273	0,39471833	0,741191792
LOC90834	-0,484613637	4,497149569	0,394865	0,741191792
TRPT1	0,445268952	5,258018868	0,394925672	0,741191792
PDS5B	-0,371556946	4,841684229	0,394965946	0,741191792
WDR1	0,407068412	6,105738831	0,395371293	0,741191792
TTC5	-0,45818619	4,685704913	0,395483009	0,741191792
ISG15	0,754527839	7,022620736	0,395615012	0,741191792
HP1BP3	0,444426514	7,191947798	0,395772712	0,741191792
MDN1	0,523881556	6,062249846	0,39583914	0,741191792
LRRCC1	0,522307399	4,211855361	0,395923772	0,741191792
DEF6	-0,37809244	4,276014019	0,395929366	0,741191792
TEAD4	0,526048187	5,516784327	0,39595461	0,741191792
PNRC2	0,448581425	8,351501489	0,395956969	0,741191792
PHF23	0,442014005	4,69203438	0,396162	0,741191792

PITPNA	-0,351227085	6,382499048	0,396178225	0,741191792
CRCP	0,408760375	6,408036696	0,396193589	0,741191792
CTDSP1	-0,345914342	8,081304409	0,396489098	0,741541184
MUL1	-0,356158261	4,310561294	0,396811665	0,741940977
KLC2	0,642740312	5,083653047	0,396940842	0,741979057
SUPT16H	0,442257708	5,186884878	0,39710221	0,742002594
H3F3A	-0,335549973	9,253333441	0,397256433	0,742002594
ZNF484	-0,389805347	2,957131377	0,397328354	0,742002594
EIF3H	0,450087539	7,780765113	0,39738869	0,742002594
SFRP4	0,968710435	6,63029723	0,39796861	0,74283991
AKT1S1	0,461603137	4,775088181	0,39817634	0,74283991
NRP1	-0,425272321	7,718740612	0,398269994	0,74283991
MYO9A	-0,337887391	5,325682401	0,398382641	0,74283991
SHKBP1	0,600092249	6,588187457	0,39845641	0,74283991
SPCS1	0,449431268	8,362001539	0,398512417	0,74283991
GOLGA1	0,453620111	4,042015509	0,398599682	0,74283991
C21orf59	-0,463930449	5,002810502	0,398832502	0,743070719
RRAGA	-0,330883637	6,486827612	0,399330258	0,743794872
INVS	-0,411554108	4,87681721	0,399725543	0,744163058
SIVA1	-0,405944256	6,009896184	0,399746192	0,744163058
EIF4ENIF1	-0,381067752	5,204101889	0,39991865	0,744280915
RNF4	0,42967693	6,198084551	0,400278006	0,744693839
C4orf34	-0,456315936	6,488300746	0,400460133	0,744693839
ATXN2L	0,371991602	7,309847502	0,400468149	0,744693839
RPS27	-0,349086476	9,768528708	0,400659262	0,744784166
AARS	0,455240899	7,078315002	0,400915353	0,744784166
C5orf42	-0,424490256	5,831958311	0,400946458	0,744784166
CIAO1	0,510204356	5,919054916	0,40105672	0,744784166
HSPB1	0,538341897	11,68968098	0,401240969	0,744784166
COX4I1	0,432093248	8,907215933	0,401256144	0,744784166
GTF2IRD1	0,54846488	4,732960483	0,401281276	0,744784166
SUMO2	0,408356375	9,50587131	0,401431218	0,744859722
C9orf123	0,451681297	6,085144243	0,401637972	0,744880455
CABIN1	-0,419236409	6,057419193	0,401660864	0,744880455
WEE1	0,504852724	6,438667771	0,401907856	0,744936025
MRPS36	-0,401490577	4,86210732	0,401909317	0,744936025
ZNF574	0,586271241	4,418515849	0,402086894	0,744982798
AARS2	0,609571048	4,526482626	0,402278277	0,744982798
CARD16	-0,502086076	5,564072016	0,402305023	0,744982798
DVL3	0,58600174	7,065200598	0,402378833	0,744982798
NDRG3	-0,354001379	4,466012931	0,402480808	0,744982798
CLP1	0,524312001	3,327224713	0,40284537	0,745455245
HMGCS1	0,673422993	7,060930667	0,403001586	0,74545571
SRM	0,425439724	6,599519795	0,403373524	0,74545571
C1QA	-0,518426336	8,473171532	0,40338562	0,74545571
CDK5	0,562032531	4,448698824	0,403462145	0,74545571
PRR3	0,648662022	5,427432423	0,403591842	0,74545571
CD2BP2	-0,388195638	5,466850272	0,403671791	0,74545571
B4GALT3	0,382975929	5,959794259	0,403691515	0,74545571
MBD6	-0,372794968	5,868871868	0,403720184	0,74545571

ACAA1	-0,337739054	6,078843914	0,40427461	0,746277359
ZNF673	0,629699999	4,738838873	0,405246216	0,747868457
TBC1D17	-0,380987807	6,913940817	0,405550822	0,748228099
COPS6	-0,3317344	7,335232687	0,405776565	0,748297824
GOLPH3L	0,473603666	4,770948474	0,405808088	0,748297824
KHSRP	0,49193755	7,457079827	0,405980851	0,748299463
CASP9	-0,312553524	5,57868744	0,406028452	0,748299463
RTEL1	0,458941102	5,706107021	0,406210789	0,748428859
PLD3	0,801229359	11,05094778	0,406318175	0,748428859
KIAA1217	0,525847812	6,107978955	0,406491864	0,748546589
HPS4	0,614485723	4,916192225	0,406838303	0,748982287
SEMA4A	-0,416581584	5,523439664	0,407089396	0,749242264
TAP1	0,490412982	6,862138397	0,40725604	0,749346718
SBF1	0,48521757	7,06026212	0,407793832	0,749352856
TPD52	0,651832611	4,910948216	0,40798439	0,749352856
ICMT	0,425078507	5,806876246	0,40799855	0,749352856
SF3B5	-0,328281291	7,327582157	0,408069892	0,749352856
FAM46A	-0,438674648	5,511837821	0,408162814	0,749352856
PHF14	0,51168104	5,030518227	0,408176355	0,749352856
KIAA0753	0,420643593	3,43284424	0,408257086	0,749352856
PLA2R1	-0,457163723	4,64936046	0,408431611	0,749352856
ARHGAP21	0,440124919	5,4501384	0,408433946	0,749352856
RUFY1	-0,361942879	5,773706434	0,408486932	0,749352856
FKBP9	0,489037528	8,067125484	0,408516678	0,749352856
RAB11A	-0,309090158	6,629899724	0,408799782	0,749352856
ZYG11B	-0,33280129	4,446345013	0,408819916	0,749352856
GPR56	0,768155088	6,803758445	0,408911528	0,749352856
HYMAI	-0,445512777	3,404434662	0,408992309	0,749352856
HNRNPA0	-0,344435217	6,457207035	0,40920336	0,749352856
PRDX5	-0,395501003	7,750039574	0,40925953	0,749352856
RPL37A	0,414254824	11,56818181	0,409548943	0,749352856
ARID4A	-0,33203849	5,238728876	0,409600781	0,749352856
MED8	0,366662357	5,604067571	0,409608287	0,749352856
STK24	0,401086645	8,285673012	0,409651846	0,749352856
DUS3L	0,45388599	4,779957017	0,409685409	0,749352856
ARMC5	0,548487592	3,412255297	0,409786889	0,749352856
PKNOX1	-0,310573751	4,828030941	0,410089806	0,749664575
CHMP1B	-0,294419504	7,855496777	0,410177229	0,749664575
TIAL1	0,366406737	5,295009053	0,410387526	0,74976889
USP31	0,446656468	6,3380408	0,410536812	0,74976889
FTH1	-0,348438	12,3223263	0,410645274	0,74976889
HERPUD2	-0,319773737	6,367088254	0,410674117	0,74976889
SDCCAG8	-0,326507019	5,265665902	0,410832694	0,749857638
QTRTD1	0,490002065	4,734421516	0,411117184	0,750018178
SEC11A	-0,445510283	5,354287603	0,411235041	0,750018178
RPLP1	0,396892739	12,94077139	0,411520731	0,750018178
DARS2	0,519688834	4,154726896	0,41155049	0,750018178
ATF2	-0,329675956	6,925640002	0,411637024	0,750018178
DUT	-0,383275964	5,910384961	0,411688833	0,750018178
DNAJC13	0,457581975	7,577534089	0,411783259	0,750018178

ARHGAP4	-0,429073274	4,286640854	0,411886978	0,750018178
PPAP2A	-0,438415288	6,921328459	0,411910555	0,750018178
RPS15A	0,390469022	10,19830972	0,412144404	0,750243644
ZXDB	0,467582158	4,854324599	0,412406821	0,75052098
MAK16	0,555396953	5,85297051	0,412654251	0,750770901
MRPS17	0,41845554	4,7645006	0,412995169	0,751040881
ZDHHC7	-0,323085816	5,254479697	0,413022922	0,751040881
RPL38	0,41928455	10,41158692	0,41343343	0,751382156
MYO1E	0,487025972	5,766442541	0,413530756	0,751382156
ITM2C	-0,511122954	7,395071441	0,413541169	0,751382156
C14orf119	-0,371545286	5,298328107	0,414012869	0,751756398
NIPA2	0,485894747	4,526129868	0,414043432	0,751756398
TPM3	0,536127754	8,842678268	0,414077875	0,751756398
RBM17	0,429149324	6,836221432	0,414467879	0,751998159
PSMA7	0,364545282	8,558931756	0,414504925	0,751998159
RCC2	0,539295377	7,220968567	0,41454188	0,751998159
VPS13B	0,498492586	6,157929176	0,415342507	0,753117653
CLTC	0,370836714	9,693579296	0,41539126	0,753117653
HTRA2	0,430659106	5,776488628	0,41559271	0,753117653
ZNF765	0,528523032	3,390891724	0,415600782	0,753117653
NIPSNAP3A	0,384588587	5,643437958	0,415827143	0,753195017
GPKOW	-0,323908149	4,492472249	0,415933997	0,753195017
HNRNPK	0,380887635	8,585136018	0,41597484	0,753195017
ASCC2	0,511969224	6,867351076	0,41613888	0,753273583
IMMT	0,405311338	6,186124586	0,416239164	0,753273583
ZNF649	-0,429641365	3,958148108	0,416574091	0,753592782
RXRA	-0,402923808	6,506610243	0,41674005	0,753592782
AIFM1	-0,35516713	4,962561801	0,416808743	0,753592782
MTR	-0,325725013	5,600501157	0,4168576	0,753592782
FHL3	0,51732172	6,379427151	0,417020609	0,753620132
LRRC8C	-0,378287357	6,443542417	0,417093764	0,753620132
CDC26	0,424372518	6,611150828	0,417418349	0,75387132
WAPAL	0,443903447	5,881051732	0,417471522	0,75387132
MARCKS	-0,400177991	5,555360669	0,417593047	0,75387132
RBPJ	0,457583195	6,787272826	0,417675003	0,75387132
BRD1	-0,447986502	4,882343261	0,418236156	0,7546844
BPTF	0,485773182	7,480935254	0,418550314	0,754728654
VPS8	0,400073042	5,138309948	0,418645837	0,754728654
FAM149B1	-0,387333159	4,7420251	0,418672095	0,754728654
HTRA1	-0,412841769	7,186337965	0,418703402	0,754728654
DSCR3	-0,29389653	6,670082932	0,419168634	0,755367579
LCLAT1	0,422498517	5,64705984	0,419525751	0,755777413
CASC4	-0,299391406	6,651552813	0,419617728	0,755777413
ACTR10	-0,354449627	5,649711124	0,419856046	0,755938558
TBCCD1	-0,414537607	4,855521025	0,419928913	0,755938558
TRAF2	0,407624123	3,650244914	0,420148531	0,756068846
LSM4	0,475986064	5,15805917	0,420223042	0,756068846
PPP2R1A	0,439668471	6,81349439	0,420628639	0,75655253
LYRM1	-0,375646769	3,583029657	0,420807935	0,75655253
SERPINB6	-0,346334374	7,189199592	0,420875152	0,75655253

IFT52	-0,323684361	4,519445722	0,420935665	0,75655253
ARFRP1	-0,393799644	4,099592141	0,421204546	0,75683631
GART	0,496002995	5,330793173	0,421411878	0,756862577
TOMM6	0,495050905	6,897011139	0,421441151	0,756862577
CHD8	0,527679201	4,108115838	0,421606825	0,756921638
RPAP2	0,425516609	4,358361763	0,42176921	0,756921638
LILRB4	-0,421458817	5,170005265	0,421807042	0,756921638
ZNF274	0,474283116	4,346183544	0,422674941	0,758132058
FBXO31	-0,3516013	5,262427012	0,422703928	0,758132058
SNRNP200	0,478308637	7,206041402	0,423645498	0,759215898
ZNF148	-0,349124578	3,89125321	0,423656608	0,759215898
PPIL3	0,460404321	4,182621914	0,423819575	0,759215898
PSMB5	0,443707975	6,073178144	0,423857819	0,759215898
CNOT3	0,47510366	6,50422369	0,423864925	0,759215898
NCK1	0,397458761	6,407035438	0,424162755	0,759380026
SNORA8	-0,375173311	14,97867908	0,42418701	0,759380026
ZCCHC8	0,498507202	6,920627965	0,424290643	0,759380026
YBX1	0,594115916	9,885286396	0,424509097	0,759560115
POLR3C	0,486473326	5,925768086	0,424634407	0,759560115
HNRNPUL2	0,468483611	8,16466616	0,424780699	0,759560115
GTPBP2	0,403957198	5,347976065	0,424928344	0,759560115
HIST1H2BC	0,554790727	8,370111047	0,424948209	0,759560115
FAM190B	0,391763975	5,873817236	0,425236506	0,759876241
EFNA1	-0,561001207	5,606695598	0,425601446	0,760137258
CSRP2BP	-0,369807514	4,143165247	0,425782031	0,760137258
ALKBH5	0,530342383	7,562783336	0,425813874	0,760137258
NCKAP1	0,473652554	6,5380089	0,425842378	0,760137258
IDS	0,446771543	8,696336871	0,426056898	0,760137258
FLJ42627	0,607131271	6,340811551	0,426086594	0,760137258
SLC12A9	-0,341568745	6,546932354	0,426186876	0,760137258
WARS	0,550228184	8,822659442	0,426420225	0,760137258
IRF2BP1	-0,425923463	5,025447896	0,426456712	0,760137258
FN3K	-0,453127885	4,483908376	0,426497309	0,760137258
OLFML2B	0,809386279	6,066712033	0,426646126	0,760203798
UBAC2	-0,36340394	5,552016798	0,426929299	0,760509637
COL6A2	0,444459587	11,13669906	0,427331204	0,760857102
NDUFV2	0,376889274	6,248397946	0,427347514	0,760857102
EGLN1	0,363763991	6,314091883	0,427534222	0,760990828
ADD1	-0,336533807	6,834599275	0,427766574	0,761161176
SIGLEC11	-0,441408012	4,583508851	0,427853173	0,761161176
COPS7A	0,396248933	6,823328839	0,427968657	0,761168043
VPS13C	-0,324453806	7,339466103	0,428225727	0,761426657
FAM175B	0,447461304	4,217116569	0,428497876	0,761711944
FAM104B	0,387011496	5,602319049	0,428695169	0,761864049
THAP4	-0,363298817	4,340074014	0,429192877	0,762483789
HERC4	0,433080232	6,272744461	0,429267527	0,762483789
IBTK	-0,320784591	5,182409209	0,429382339	0,762489107
ASCC3	0,562254165	5,938060254	0,429812996	0,763055147
FGFR1OP2	0,455858913	5,200201631	0,429951574	0,763102495
TRIM4	-0,343474727	4,214739558	0,430231423	0,763400487

TNFRSF12A	0,605230148	7,650823723	0,430539715	0,763748782
GATC	0,525358688	5,354233839	0,430806263	0,763770534
SEPT9	0,393363469	8,857514199	0,430865165	0,763770534
CHSY1	-0,414810185	5,537420222	0,430887996	0,763770534
ZNF880	0,462591567	4,766211895	0,431083418	0,763895728
IPPK	0,42714002	4,917359844	0,431182675	0,763895728
GLI2	0,636782047	5,570518406	0,431516701	0,764227424
SP2	-0,31413983	4,842293583	0,431594047	0,764227424
NDUFAF4	0,392923678	5,677197875	0,43207009	0,764692135
LYST	-0,413650534	5,780737215	0,432080774	0,764692135
TMEM70	0,39474417	5,664953603	0,43239575	0,765051017
CSF1	-0,369187706	6,624931892	0,432511446	0,765057212
INPP4A	-0,328301331	4,859203363	0,432673876	0,765146048
TALDO1	0,496120851	9,077816733	0,432931544	0,765403215
ERCC2	0,43986524	4,003988934	0,433107145	0,765515195
NFIA	-0,465218766	7,667596274	0,433304133	0,76555512
INSIG1	-0,347378715	6,052680183	0,433411943	0,76555512
CCDC93	-0,394973233	3,793684852	0,433466537	0,76555512
JTB	0,34412993	8,136082986	0,433615305	0,765612861
RBM25	-0,3732226	8,421715405	0,433836652	0,765612861
CSF1R	-0,362682345	5,196200575	0,433926576	0,765612861
MESDC1	0,482332143	3,574240365	0,433948337	0,765612861
DHX16	0,546288594	3,762290329	0,434257663	0,765960425
MREG	0,518486017	4,654064684	0,434505455	0,766041161
RPL29	0,423953313	10,48519055	0,434604272	0,766041161
OAS1	0,632991695	4,939335164	0,434744105	0,766041161
YWHAG	0,455953961	7,358399895	0,434798457	0,766041161
DLAT	0,513899605	5,901697083	0,435079693	0,766041161
PRPF38A	-0,287672852	5,421216689	0,435080957	0,766041161
MKLN1	-0,331239077	5,566724007	0,435170625	0,766041161
ZNF436	-0,366168837	3,867007332	0,435279316	0,766041161
UBE3C	0,435516723	6,729380047	0,435314489	0,766041161
ARHGEF5	0,65110396	5,107128102	0,435788656	0,766394675
DGCR2	-0,390897323	6,487781789	0,435837456	0,766394675
PLEKHA6	0,580919959	3,485322459	0,435852552	0,766394675
SDHD	-0,307963611	6,289855429	0,436014844	0,766482398
APTX	0,519053341	4,31886323	0,436319941	0,766687403
KIAA1522	0,530049545	7,012741777	0,436442102	0,766687403
NSMCE4A	-0,309623361	4,97342715	0,436468764	0,766687403
ATP5G3	0,438732002	6,573382898	0,436661792	0,766828936
DNAJC8	-0,328847599	6,162957658	0,43677613	0,766832243
C7orf49	-0,383301117	3,512010391	0,437060931	0,767002752
MTMR3	-0,375871336	6,329027962	0,43709821	0,767002752
CTNNBIP1	-0,384907963	6,141654206	0,437319133	0,767192994
EPM2AIP1	-0,395273922	5,006553653	0,437574115	0,767442874
ELL2	0,539594577	8,122778959	0,437722281	0,767505332
COPE	0,40512199	8,184198579	0,437934321	0,767679726
URM1	-0,341296009	4,229325809	0,439190134	0,7695622
CRLS1	0,361135336	5,980949709	0,439391851	0,7695622
C20orf112	0,66785998	5,639380586	0,439411052	0,7695622

DCP2	0,412015355	5,964211662	0,439459629	0,7695622
OFD1	-0,378529913	5,659796174	0,439652246	0,76970184
RAPGEF6	0,420453442	5,016598048	0,440202158	0,770466764
DGCR11	0,629456812	5,635800907	0,440401782	0,770618361
GGCT	0,57409466	5,699430259	0,440534467	0,770652778
MS4A6A	-0,351573015	7,004413809	0,440811114	0,770890586
ELMOD2	0,348508801	5,274320434	0,440896507	0,770890586
LAPTM4A	-0,346054164	9,734512091	0,441180646	0,77103215
TRIM21	0,469526109	4,741336111	0,44121473	0,77103215
TP53	0,509429395	7,111246222	0,441316686	0,77103215
CAPN7	-0,316255079	5,228683246	0,441679444	0,77119654
SLC41A2	0,593374184	4,581014516	0,441734184	0,77119654
METTL6	0,448201604	4,878975631	0,441750064	0,77119654
TBC1D22A	-0,303019154	5,754511313	0,442422142	0,772172148
SNX13	-0,357916883	6,032583446	0,442631499	0,772339864
FBXL19	-0,375407718	3,607403089	0,442854815	0,772531846
LRIG1	-0,432581144	6,126941342	0,443083272	0,772732693
ATF5	0,529988915	6,653507066	0,443337201	0,772858836
DDX24	-0,302279986	7,221924708	0,443469906	0,772858836
ZBTB44	0,437713021	5,541923749	0,443495619	0,772858836
SCRIB	0,559082117	4,745121641	0,443868487	0,773170022
MECP2	-0,290158109	6,110089347	0,443929836	0,773170022
ERGIC1	-0,300337339	6,289708759	0,444185412	0,773170022
APBA3	0,439573587	5,469906978	0,444255165	0,773170022
INPP1	-0,359160864	5,337792252	0,444277667	0,773170022
ASH2L	-0,427609304	5,269523496	0,444354497	0,773170022
HACE1	0,418467736	4,53189113	0,444602647	0,773404452
L3MBTL4	-0,449532445	4,644018262	0,444758921	0,773469278
RDX	0,485378546	7,899516312	0,444983121	0,773469278
ZNF514	-0,332643953	4,869407608	0,445091241	0,773469278
UBE2E1	0,35559225	7,481090058	0,445093628	0,773469278
TAF1D	0,440557214	7,114427924	0,445471966	0,773833168
CCDC90B	0,466818496	4,838355863	0,445659746	0,773833168
RNF149	-0,304143875	6,70267524	0,44585282	0,773833168
CYP20A1	-0,273675965	6,208568848	0,445952652	0,773833168
ARL8A	-0,30606137	6,474098059	0,445972957	0,773833168
ACBD5	-0,347404774	5,060217499	0,44603662	0,773833168
MORF4L2	0,582593585	5,577136638	0,446097402	0,773833168
IL18R1	0,521825675	3,385842765	0,446347523	0,773917265
HES4	0,737470795	5,089984123	0,44637287	0,773917265
HCFC1	0,439755154	7,663654381	0,446629457	0,774101843
USP16	0,400222371	6,186295067	0,446706372	0,774101843
RICTOR	0,375116329	5,687811857	0,446932415	0,774296782
CDK19	-0,350501689	5,361266334	0,447180769	0,774373321
CREB1	-0,308411371	5,74723742	0,447203716	0,774373321
ZNF507	0,426244752	3,880277678	0,447532479	0,774745869
SPTLC1	0,326222468	7,124162731	0,447682242	0,774757723
COL3A1	0,545164586	12,51324811	0,447766562	0,774757723
KIDINS220	-0,3149818	6,584156437	0,448165824	0,775195529
BRPF3	0,493712037	5,934630843	0,448246953	0,775195529

SIK3	0,575919096	6,510730244	0,448717884	0,775767251
GPD2	0,350663872	5,239531066	0,448805075	0,775767251
MRPL49	-0,331323264	4,051026943	0,449104732	0,776056809
CEPT1	-0,294613955	5,399976609	0,449248744	0,776056809
CDK13	-0,286438296	5,324239454	0,449349827	0,776056809
PDCL	-0,333326391	4,49255569	0,449427825	0,776056809
C12orf29	0,389813917	4,515521372	0,449814095	0,776137794
SIGLEC10	-0,425325475	4,49422996	0,449822122	0,776137794
POLR1A	0,377859845	5,23356303	0,449920029	0,776137794
HSDL1	0,444567207	4,651380917	0,450022686	0,776137794
NCKAP5L	-0,336473278	5,559918317	0,450043824	0,776137794
TNK2	0,636976783	6,024506495	0,450598504	0,776547239
NUMB	-0,309650907	7,303726754	0,450686441	0,776547239
RGMB	0,53480983	5,365393667	0,450811858	0,776547239
PTPRA	-0,371465638	6,309512887	0,450866546	0,776547239
SGPL1	0,419213967	6,28436871	0,450998764	0,776547239
UGP2	0,449193665	6,776740393	0,45106941	0,776547239
DDX19B	-0,279009722	6,30848458	0,451135586	0,776547239
RPL18A	0,398133975	10,10888067	0,451253722	0,776547239
ZNF192	0,590993362	5,489964412	0,45130616	0,776547239
PARP3	-0,319442307	5,139139266	0,451659344	0,776958897
RAB5C	-0,284643075	7,628696446	0,452144193	0,777596785
MTMR14	-0,287619558	5,950071451	0,452395209	0,777720999
KIAA0494	-0,297043915	6,671256693	0,452444523	0,777720999
TPP2	0,35762884	6,643319758	0,452560428	0,777724184
ATP5A1	0,433095126	7,987319809	0,453386923	0,778948205
USP32	0,337781804	6,860470556	0,453667221	0,779015067
KIAA1468	-0,292179317	5,291868221	0,453899394	0,779015067
EIF3C	-0,279622994	7,984909215	0,453907491	0,779015067
CMTM3	-0,305779042	7,358180734	0,453907573	0,779015067
ATP5D	-0,324179099	6,046259623	0,453997049	0,779015067
UBR5	0,414298968	6,479225941	0,454185093	0,779141672
FAM195B	0,413625481	6,132344308	0,454701307	0,779630039
RFFL	0,393515898	6,201728931	0,454824241	0,779630039
ISOC1	0,460235713	5,463291188	0,454872539	0,779630039
SRPX	0,586029241	5,675130582	0,454927105	0,779630039
ROGDI	-0,314200853	5,777019853	0,455303483	0,780079008
ALG8	0,500411062	5,421724038	0,45563202	0,780445804
EIF3D	0,350974997	6,871969572	0,456244073	0,780869092
PPP4C	0,321856764	6,903316802	0,456510025	0,780869092
PCOLCE	0,73487166	8,260124371	0,456906086	0,780869092
DUSP12	0,434741115	5,404915807	0,456977699	0,780869092
INPPL1	0,425222868	6,647297874	0,457078401	0,780869092
TSTA3	0,331816488	6,318127901	0,457087229	0,780869092
NUP35	0,466688371	3,112658275	0,457258887	0,780869092
TMEM126B	0,538398871	5,878235355	0,457302031	0,780869092
ZNF33A	0,395017899	5,838954915	0,457360768	0,780869092
MRPL16	0,614737833	6,315225492	0,457416492	0,780869092
ZNF548	0,644340779	3,620466619	0,457472808	0,780869092
STK3	0,386774394	5,360431959	0,457478156	0,780869092

SCARB1	0,684204434	6,17211525	0,457569691	0,780869092
DDX21	0,580382507	5,106883768	0,457601689	0,780869092
NSD1	-0,390541902	3,099701403	0,457629545	0,780869092
RBMS2	-0,357607678	6,252707639	0,457863425	0,780869092
FZD5	-0,357527869	6,5193777	0,458078509	0,780869092
TMLHE	-0,388603407	4,004309045	0,458320077	0,780869092
BRAP	0,383809451	5,853602911	0,458333074	0,780869092
SCP2	-0,324051292	5,422425876	0,458364569	0,780869092
ITGB1BP1	0,348727418	6,956483159	0,458389483	0,780869092
DPAGT1	0,453076801	4,879155053	0,458398442	0,780869092
DDX60	-0,345973622	5,480084936	0,458671643	0,781017042
BAZ2A	0,366853997	6,324141534	0,458714365	0,781017042
KDELR1	0,414420233	8,42384647	0,459360927	0,781834206
SDC2	0,659834684	8,514814502	0,459521813	0,781834206
EPB41L2	-0,317117791	7,239059444	0,459538275	0,781834206
FAM160B2	-0,325086187	5,619155423	0,459988948	0,78229335
SLK	-0,304071822	5,062220629	0,460385026	0,78229335
GPBP1L1	0,419990484	8,313972858	0,460444583	0,78229335
RBMS1	-0,291976416	7,746015195	0,460535228	0,78229335
ZGPAT	0,401079802	4,685390304	0,460559523	0,78229335
EDIL3	0,882154825	7,098720403	0,460630257	0,78229335
ACTA2	-0,546123006	8,951777628	0,460825009	0,78229335
CUL5	0,4051905	5,702829679	0,460839296	0,78229335
TSR1	0,517776644	4,902818639	0,460891301	0,78229335
TIMM22	0,362576178	4,994676843	0,46099587	0,78229335
FRG1	0,363396487	6,518415847	0,461130507	0,78229335
COL5A1	0,565308272	8,135494601	0,461282171	0,78229335
ANKRD36B	-0,391095748	6,202914388	0,46129954	0,78229335
NEK1	0,390391278	4,813842942	0,461503785	0,782380527
TSEN34	-0,324088954	5,194889959	0,461580416	0,782380527
WIBG	0,42173143	5,245589804	0,462066586	0,782978479
IMPACT	0,395117235	5,818999689	0,462162836	0,782978479
FAM50A	0,41033268	5,039787197	0,462468344	0,783301449
WRB	-0,343815663	6,25321682	0,46266811	0,783372759
ZNF44	0,566423649	5,824515464	0,463005188	0,783372759
GABARAPL2	-0,279042886	6,704360436	0,463009246	0,783372759
CSTF2	0,356820537	4,492458465	0,463059528	0,783372759
RAI1	0,455858208	6,714440188	0,46308485	0,783372759
ZNF780A	0,634152294	5,650306993	0,463251982	0,783399219
ZNF558	0,355164949	4,19248236	0,463330261	0,783399219
SPAST	0,476528678	6,076804492	0,463633701	0,783717949
DUSP3	-0,298353827	5,796759378	0,464155005	0,784287219
TRA2A	-0,260501665	6,126210872	0,464200501	0,784287219
CSNK2A2	0,402774397	7,053288235	0,464401544	0,784432531
VPS18	-0,293544423	4,778846923	0,464771788	0,784862196
ZNF217	-0,274089658	4,967300184	0,464886114	0,784862196
DOPEY2	-0,328159443	4,546951381	0,465329376	0,784919789
ANKLE2	0,410292772	7,770283915	0,465351285	0,784919789
AKAP10	0,444201065	5,899085493	0,465381908	0,784919789
GSDMD	-0,334623333	6,606244494	0,465405647	0,784919789

ZNF621	0,446457609	4,611549998	0,465571839	0,784919789
SPPL2A	-0,279233281	6,636094066	0,465721612	0,784919789
FLOT1	0,380433433	7,760863721	0,465752972	0,784919789
PRR24	-0,379596666	5,626132314	0,466172244	0,784919789
ACAD11	0,428514544	4,833193801	0,46621994	0,784919789
VAV2	0,393161029	6,44848238	0,466229788	0,784919789
JARID2	0,472638747	5,927967445	0,466274511	0,784919789
MINK1	-0,301771796	6,707472425	0,46630152	0,784919789
PARG	-0,353092156	3,753136137	0,466548173	0,785141162
MRPL53	0,359263714	6,977124322	0,466688875	0,785184169
TERF1	0,441682864	3,190064005	0,467179389	0,785785004
CREBL2	-0,337817014	5,760725025	0,467384762	0,785785004
DCXR	-0,3269501	6,120740035	0,467412476	0,785785004
BRI3BP	-0,301269441	4,732891935	0,467506931	0,785785004
CGGBP1	0,342680044	6,28874522	0,467655353	0,785840771
SEMA3C	0,675183107	6,936822314	0,467787065	0,785868439
FEM1C	-0,335547629	4,689541398	0,468106854	0,786211979
TBC1D25	0,508253616	5,209612902	0,468246774	0,786253325
QPCTL	0,770374209	4,940595567	0,468531524	0,786376314
IREB2	0,376093525	6,129594839	0,468550662	0,786376314
DDX46	-0,349829253	4,652186392	0,468914967	0,786706153
RPS13	0,425295005	10,00321388	0,468977931	0,786706153
FBXW4	-0,327493208	6,754509443	0,469548165	0,787403359
ACOT8	0,417447575	4,622194958	0,469624499	0,787403359
HSPH1	0,505685063	6,574045075	0,470114533	0,787477823
PIK3AP1	-0,366443778	6,30367051	0,470305708	0,787477823
FAM175A	-0,363488629	4,95745709	0,470349763	0,787477823
DDX55	0,406527964	5,83035907	0,470371048	0,787477823
TMBIM6	-0,337659498	9,790593116	0,47043897	0,787477823
PDZD11	0,412395534	5,202414808	0,470466346	0,787477823
WDR70	0,4032287	5,432982825	0,470477292	0,787477823
ITPR3	-0,319833964	6,472429762	0,47102679	0,787894629
SUPV3L1	0,413437177	5,2034374	0,471159918	0,787894629
SLC25A5	0,382046346	8,497538714	0,471212967	0,787894629
SRPR	-0,318729443	7,551239407	0,471226157	0,787894629
CAPZA1	0,459310231	7,352223175	0,471539556	0,787894629
LIMD2	-0,299952504	5,81406345	0,471558886	0,787894629
TMX1	0,386941655	7,158714599	0,471572309	0,787894629
STUB1	-0,374259013	4,053432248	0,471650664	0,787894629
SF3A1	-0,298448336	7,446388589	0,472330817	0,788431192
CD2AP	0,47957747	7,938957852	0,472360821	0,788431192
CTNNAL1	0,57531428	6,243273019	0,472396657	0,788431192
PGAP1	-0,317755136	6,030440384	0,472500515	0,788431192
CTSA	-0,32840172	7,158592022	0,472647991	0,788431192
ZNF212	0,481887378	3,41830155	0,472665598	0,788431192
TRAM1	-0,301385883	8,151095693	0,472810524	0,788480059
BAZ1A	0,461850673	5,162294146	0,473207336	0,788837297
FSCN1	0,511790754	7,670185137	0,473256105	0,788837297
WIPF1	-0,357897395	6,932038511	0,473418627	0,788915352
PCMT1	0,476168944	4,168604605	0,473656422	0,789118774

GDI1	0,477984719	6,357918607	0,474238797	0,78953584
EHD1	-0,346466896	6,198125311	0,474370409	0,78953584
FOXN2	0,384873814	4,249081595	0,474393156	0,78953584
FAM178A	-0,299114906	5,221590677	0,474684646	0,78953584
AP1B1	0,383507148	7,363265831	0,474694977	0,78953584
SRRM1	-0,325627245	7,473703879	0,474713839	0,78953584
LAMB1	-0,291577863	7,383683419	0,474872023	0,78953584
PDIA3P	0,318445361	8,475297786	0,474996096	0,78953584
SAR1A	-0,257198041	6,952352692	0,475109361	0,78953584
PPP1CA	0,35341091	7,744483634	0,475278215	0,78953584
ADCY9	-0,339481121	5,271899533	0,475389996	0,78953584
CNIH	0,312415426	7,687409595	0,475517477	0,78953584
RABEP1	0,553615703	5,417678431	0,475591328	0,78953584
MAP3K1	-0,331658054	6,322956775	0,475647847	0,78953584
SKA2	0,522076045	5,179861299	0,475734245	0,78953584
JAK2	-0,321640454	5,134202717	0,475759315	0,78953584
TIPRL	0,38739374	4,759560892	0,476029252	0,789791598
ZNF320	0,574268189	4,905547024	0,476618831	0,790577428
DNTTIP2	0,462545624	6,760809761	0,476886415	0,790828906
PPP2R4	0,405225986	5,836167798	0,477813457	0,792173588
ZNF117	0,669622307	5,447801676	0,478213713	0,79253059
WDR44	0,429556551	5,365191564	0,478261238	0,79253059
TIMM17B	0,384410955	4,198048388	0,478427174	0,79261295
AHI1	-0,357059216	4,859294984	0,478939785	0,793269466
RAB3IP	-0,311695362	4,712937509	0,479250121	0,793590717
IDH1	0,456308679	6,470713399	0,479791172	0,794293761
DDX58	0,580513972	6,649253815	0,480229129	0,794477864
SAMD8	-0,301957704	3,767830873	0,480320873	0,794477864
ZFHX3	-0,348744172	7,465337	0,480403019	0,794477864
PPP1R15B	0,330551286	7,674817025	0,480498014	0,794477864
ARHGAP1	-0,288661185	7,59992885	0,480542195	0,794477864
NRBF2	0,500523227	5,514321385	0,480661037	0,794477864
EPB41	-0,360641533	5,202562716	0,480717945	0,794477864
PGGT1B	-0,270502677	5,014640372	0,480917646	0,794508666
LARS	0,313231144	6,257058947	0,480995467	0,794508666
ING3	0,355009741	3,750018431	0,481086124	0,794508666
APEX1	0,432798532	6,617061267	0,481203558	0,794510185
CDK11B	0,398001791	5,44289176	0,48156941	0,794882143
APPL2	0,389591792	5,095694416	0,481661976	0,794882143
ZCCHC10	0,356502271	4,077869052	0,482197179	0,795572843
ZNF827	0,414135965	4,983746953	0,482553389	0,795681018
TFB2M	0,475277878	4,168156541	0,482637923	0,795681018
CANX	-0,426696175	7,819794006	0,482736265	0,795681018
SGSM3	-0,32935302	6,863006461	0,482925122	0,795681018
TWF2	-0,301679844	7,070630872	0,483034659	0,795681018
VDAC3	0,585983597	6,534012451	0,483122654	0,795681018
ERGIC3	0,405528865	7,093682481	0,483163526	0,795681018
TECR	-0,335179933	7,317630167	0,483246956	0,795681018
IL7R	-0,460332441	6,632615194	0,483338712	0,795681018
CALR	-0,315673444	10,00756553	0,483502601	0,795681018

ANXA6	-0,332047742	8,403229866	0,483546288	0,795681018
PHC3	-0,285080288	6,805407517	0,483996015	0,796106473
TBC1D22B	0,393509448	4,226299741	0,484038339	0,796106473
MSN	-0,283114316	7,744714655	0,484271268	0,796290145
KRT19	0,633299823	7,715291653	0,484383564	0,796290145
TAB1	-0,30952887	4,373455885	0,484679604	0,796584773
KIAA1191	0,414740531	7,142712948	0,48490725	0,796654568
SNX33	-0,306311364	5,813141456	0,484984553	0,796654568
GLT8D2	0,45521265	5,064620038	0,485072557	0,796654568
UBTD2	0,386653093	6,084841962	0,485736728	0,797553274
CCDC134	0,471716162	4,983607366	0,486020614	0,797610313
RNF181	0,374748551	7,066187	0,486048127	0,797610313
SYDE1	0,420510718	6,414122887	0,486161765	0,797610313
PDE4D	-0,386304558	5,862579859	0,486435351	0,797610313
STT3A	0,343395548	7,084878331	0,486453032	0,797610313
ZNF23	0,396566159	4,409348187	0,48655109	0,797610313
EI24	0,374119689	7,434240278	0,486590248	0,797610313
MYO5A	0,399321349	6,546402743	0,487048153	0,797781811
EEF1G	0,410200912	10,90598924	0,487253504	0,797781811
HIPK1	0,616549248	4,26697555	0,487309421	0,797781811
C7orf43	-0,2720333	5,377117773	0,48731598	0,797781811
PDGFRA	-0,42645525	6,794564886	0,487416353	0,797781811
STX6	-0,303182882	5,232359616	0,487575891	0,797781811
NSL1	0,469962382	4,947295078	0,487619325	0,797781811
NUPL1	0,304303253	5,885766543	0,487630824	0,797781811
RAB14	-0,348592041	7,45254176	0,487821025	0,797901552
EXOC6B	-0,324474961	4,21915964	0,488647828	0,799062239
NUBP1	-0,249051132	5,006361791	0,488846092	0,799067823
NFATC2IP	0,320183793	6,442172213	0,488885608	0,799067823
FNIP1	-0,29739756	6,608351839	0,489249835	0,79935998
UBL5	-0,307048139	5,59765064	0,489355984	0,79935998
ZFAND5	-0,301442416	8,79727178	0,489416031	0,79935998
CDC37	-0,282281137	6,541122179	0,489956952	0,799816959
HMGN2	-0,250205627	7,042488695	0,489990582	0,799816959
STRBP	0,398082819	3,991537423	0,490047698	0,799816959
AHCTF1	0,382891456	6,04612992	0,490210513	0,799891238
TRIM27	0,389176049	6,02582521	0,490805089	0,800577163
MOV10	0,436688997	6,127634654	0,490865687	0,800577163
PTPRS	-0,437441309	6,624641619	0,490994732	0,800596144
ARFGEF1	0,322333948	5,579579494	0,491134314	0,800632295
EIF5	0,335263471	5,944473789	0,491563331	0,801100014
UNC50	-0,28373234	6,069230122	0,49165619	0,801100014
CCDC130	0,523863193	4,008140751	0,491920579	0,801319753
SAMD4A	-0,346799736	5,206188245	0,492041596	0,801319753
NFE2L2	-0,303126719	7,119069093	0,492143588	0,801319753
GGA1	-0,302755863	4,628749965	0,492776688	0,802007891
TUBB6	-0,384150592	6,551919743	0,492801446	0,802007891
MFSD6	-0,353019968	6,281635848	0,493022304	0,802175875
POLR1D	-0,327891755	5,635459008	0,493188688	0,802255167
ASPN	0,711430553	6,858813595	0,493407925	0,802420377

TUSC3	-0,387569181	6,47728178	0,494163396	0,803427573
SPSB2	0,435519908	4,059480464	0,494262893	0,803427573
RAVER1	0,466636456	6,504310685	0,494434983	0,803515766
GLA	0,385351082	4,855447043	0,495212818	0,80419882
PNN	0,391335745	6,426706265	0,495247086	0,80419882
STX18	0,435613805	5,902829138	0,495298909	0,80419882
C1QTNF3	0,776960021	5,228114615	0,495327034	0,80419882
PMS1	0,442364007	3,874195878	0,495817712	0,804384144
SMARCA1	-0,289815809	6,190692772	0,495837673	0,804384144
NUP50	0,338745037	6,313704231	0,495849945	0,804384144
ZNHIT1	-0,293415484	5,540402086	0,495913028	0,804384144
FNTA	0,380676581	6,435672063	0,496271447	0,804774077
MRFAP1L1	0,35919713	6,402908983	0,496809122	0,805277067
MUT	-0,288025332	5,346853244	0,496817806	0,805277067
AMD1	-0,270840801	6,165015873	0,497361433	0,805456861
MRPL43	-0,249423038	6,650873199	0,497370916	0,805456861
VPS11	-0,265024184	5,749774418	0,497379601	0,805456861
CDK2AP2	-0,28797781	6,511944033	0,497401209	0,805456861
ATP6V1A	0,350389249	5,510289554	0,497603106	0,805592493
KIAA1731	0,417433265	4,913231751	0,497825966	0,805761989
CDC42SE1	0,313098052	7,045687438	0,498275553	0,806002996
ACAA2	-0,261566998	6,173344224	0,49840943	0,806002996
TMEM60	-0,364632236	5,78926422	0,498474524	0,806002996
PANK3	0,339336967	6,554472089	0,49861647	0,806002996
GPAM	0,454643034	5,203051225	0,498793964	0,806002996
RPL27A	0,379880092	11,18319969	0,498837348	0,806002996
SYNRG	0,346197756	6,819170034	0,498985791	0,806002996
FLJ45340	-0,307820408	6,443811612	0,499053229	0,806002996
SSRP1	0,311659877	7,410799054	0,499175173	0,806002996
TRIO	0,390452842	7,295326039	0,49939767	0,806002996
C1orf85	0,376783905	6,893250125	0,499398632	0,806002996
FBXW7	0,374851686	5,515445352	0,499485691	0,806002996
LOC440944	0,414301739	3,913484663	0,499702399	0,806002996
NDUFS4	-0,310031769	6,090487051	0,499821872	0,806002996
FBXO3	-0,231431477	5,448956606	0,499846888	0,806002996
OGFRL1	-0,353311326	5,471051692	0,499887511	0,806002996
LENG8	-0,454608125	7,32712363	0,500047487	0,806002996
KIAA0319L	-0,29069748	5,86887883	0,50010246	0,806002996
TSPO	-0,323793247	6,267314278	0,500454396	0,806379614
BCL2L13	0,405933	5,00713457	0,500808502	0,80675955
KIF3A	0,432147555	3,327626963	0,501156503	0,807018091
HNRNPA3	-0,468683133	5,636508978	0,50127771	0,807018091
EXOSC9	0,357867148	5,399983587	0,501324041	0,807018091
DUSP7	0,347935018	7,273243757	0,501629964	0,807319973
WBP1	-0,321950226	6,698699318	0,501769238	0,807353571
PCGF3	-0,304775895	5,148885326	0,502041922	0,807601761
ITGA4	-0,288524817	6,381948628	0,502296931	0,807821408
PGD	0,479455762	8,00387431	0,502417254	0,807824393
SRGN	-0,358092465	8,869787459	0,502549598	0,807846702
ZRANB1	0,617845288	6,684978426	0,502914219	0,807907627

FAM127C	0,426284458	6,143411868	0,503004316	0,807907627
HNRNPC	0,351951684	7,863043186	0,503027496	0,807907627
TLE1	-0,26471115	5,640979911	0,503061414	0,807907627
BNIP2	-0,261379366	6,116939155	0,503498354	0,808418949
TNFRSF10B	0,379839342	5,678534943	0,503853675	0,808518651
PAK2	0,338767311	6,329327921	0,503955938	0,808518651
ECH1	-0,30703848	7,232996101	0,504310468	0,808518651
HCFC1R1	0,388836495	6,254543578	0,504355757	0,808518651
UGGT1	0,387002943	4,931519449	0,50446036	0,808518651
EXT1	-0,288292708	6,774049608	0,504592506	0,808518651
SAR1B	-0,28011474	5,222700315	0,504604299	0,808518651
ATN1	0,437097232	8,392241138	0,504766131	0,808518651
TAF8	-0,281906106	4,992661669	0,504800222	0,808518651
PPP2R1B	0,383397984	5,133653452	0,504849744	0,808518651
CBLL1	0,353422994	5,03126397	0,504910635	0,808518651
NOC3L	0,383995952	3,904892909	0,505211956	0,808518651
THAP1	0,394643008	6,004924922	0,505338758	0,808518651
ZNF654	-0,267329733	4,142729655	0,505500312	0,808518651
APRT	0,372973159	7,046099897	0,505526518	0,808518651
ZBTB40	-0,284873281	4,408881849	0,505554982	0,808518651
FUNDC2	0,290746277	5,574120294	0,505576115	0,808518651
MRPS15	0,385042498	5,873262812	0,505846682	0,808761671
TNPO3	0,323273816	6,453162155	0,50614888	0,809055136
GLUD1	-0,275368778	6,101145109	0,506326195	0,809148893
QRICH1	0,414056567	6,119587504	0,506942576	0,809762193
CLOCK	0,287484618	5,669662949	0,507138725	0,809762193
FARSA	0,368864038	7,314385571	0,507178616	0,809762193
BAG4	0,465826339	6,460269365	0,507185705	0,809762193
CLDND1	-0,343229931	5,686143216	0,507360486	0,809762193
HEXB	-0,278985775	7,017950451	0,507422474	0,809762193
ATP6AP1	-0,282720492	7,30632613	0,508035357	0,810119901
PDLIM7	0,435306128	7,782475719	0,508314885	0,810119901
DPH2	0,419460911	4,782759415	0,508324283	0,810119901
RANGAP1	0,418160686	4,969185422	0,508394445	0,810119901
RPS6KA4	0,341206596	5,550613443	0,508521553	0,810119901
SEPHS1	0,374575153	6,651973417	0,508693242	0,810119901
TRIP6	0,34975039	7,253931934	0,509069671	0,810119901
TEX2	-0,282418986	5,583176441	0,5091308	0,810119901
UQCRFS1	0,357048565	7,915659027	0,509149141	0,810119901
MRPS35	0,507436696	5,939044758	0,509164204	0,810119901
TIPARP	-0,358961297	6,426624191	0,509527008	0,810119901
ZNF395	-0,29462126	5,7192646	0,509542781	0,810119901
CAMK1	-0,323261624	4,459550142	0,509559278	0,810119901
CKB	-0,413854109	7,317411242	0,509603306	0,810119901
PARP10	0,399300322	6,533488922	0,509653808	0,810119901
CWC22	-0,262451418	4,536077411	0,509671554	0,810119901
UBE2W	0,327548284	5,306989923	0,509757673	0,810119901
SYNJ1	-0,258389489	3,823812807	0,509785085	0,810119901
RAP2A	-0,294950411	5,503341482	0,510131234	0,810295759
MARCH5	0,30913054	5,738237276	0,510193353	0,810295759

ATR	0,36782569	5,271913047	0,510328661	0,810295759
TAF6L	-0,270606654	4,537457022	0,510371064	0,810295759
PIP5K1C	-0,317109483	6,637506024	0,510930483	0,810543783
CUL1	0,342605945	6,390636703	0,510937219	0,810543783
YWHAE	0,355471888	8,928257971	0,510950648	0,810543783
ABL1	-0,245471734	6,567703472	0,511002746	0,810543783
RANBP3	0,316044015	6,6397017	0,511141896	0,81057595
MED1	0,366789653	5,509111834	0,511351322	0,810678273
ZNF28	-0,376950778	3,736188157	0,51144419	0,810678273
TCFL5	0,407469743	3,103516328	0,511732739	0,81094714
PEF1	-0,263507345	6,076495659	0,512101504	0,811080486
RELB	0,331393896	6,237592379	0,512119229	0,811080486
PRSS23	0,498718405	6,403727916	0,51221005	0,811080486
SPARC	-0,357097638	10,99727163	0,512332399	0,811080486
SLC35A4	-0,254992834	6,300076622	0,512411605	0,811080486
NEDD4L	-0,310623948	5,870270399	0,513701759	0,81292737
KATNAL1	-0,303287594	4,291937713	0,51381683	0,81292737
DCTN2	0,280570191	7,667145654	0,514061836	0,813126342
DYNLL2	-0,241561219	6,711837103	0,514397892	0,813292581
PSMB3	0,326480539	7,730455894	0,51440547	0,813292581
RPL9	0,346282142	6,022413861	0,514534004	0,813307227
TNFRSF10D	-0,288891573	4,220002608	0,514674966	0,813341505
RPL10A	-0,247370753	9,708635102	0,514914746	0,813531893
NRF1	-0,237132612	5,078723607	0,515239993	0,813853464
SEMA4C	0,379060341	6,855418201	0,515371416	0,813853464
WDR74	0,501108669	4,876713344	0,515524546	0,813853464
OSBPL1A	-0,272202537	6,261770845	0,515595683	0,813853464
KLHL20	-0,291672533	5,458304934	0,515940529	0,814209319
C6orf89	0,329965357	7,198857274	0,516359982	0,81468272
DIAPH2	-0,322509415	3,469446132	0,516918957	0,815134829
TTL	0,396004274	5,618637829	0,51693691	0,815134829
ZNF37A	0,369119231	5,118165574	0,517005152	0,815134829
TRAPPC10	0,364811353	6,000963589	0,517844938	0,816258321
PGRMC2	-0,307543609	5,89353892	0,517957142	0,816258321
PNP	-0,302612368	4,775862448	0,518144204	0,816364447
CAMTA2	-0,245005384	5,372533666	0,518670066	0,816866785
EXTL3	-0,310301746	3,716426744	0,518702622	0,816866785
CDC42EP1	-0,288067518	8,661063633	0,518916829	0,817015436
PCGF5	-0,28563304	5,853466881	0,519044577	0,817027925
FKBP1A	-0,270626524	10,53710667	0,519525098	0,817595579
RAB28	0,390765332	4,843344284	0,519978736	0,818087774
TYW3	0,457406856	5,563989922	0,520077798	0,818087774
OBSL1	-0,322710793	5,989984305	0,52027202	0,818204544
CLN3	-0,244940564	6,14976082	0,520894134	0,818829546
SNTB1	-0,319868189	5,505814453	0,520940877	0,818829546
GNL1	0,371632857	5,889549107	0,521029682	0,818829546
MALT1	0,357388348	4,865264353	0,521276063	0,818851658
PPP2R5D	0,35841446	4,737311587	0,52128392	0,818851658
RNASET2	-0,248338029	7,078440344	0,521441257	0,818910164
C6orf62	0,432529027	6,713534916	0,521814392	0,819222691

RPN2	0,32393541	8,2265502	0,521880535	0,819222691
ANAPC13	0,416561708	6,99439842	0,522061056	0,819317454
CCDC142	0,402509321	4,541090642	0,522868783	0,820303084
TNPO2	-0,288108491	5,613254718	0,523040683	0,820303084
RAP1B	0,342820879	6,849305803	0,523049979	0,820303084
HSD17B10	-0,265540013	5,160537683	0,523367613	0,820525885
ITPA	0,455878073	4,669087007	0,523432703	0,820525885
CTCF	0,306873624	6,226962589	0,523698873	0,820754451
S100A11	0,473930148	10,21670716	0,523907736	0,820794817
WHSC2	0,40635074	4,878686537	0,524074892	0,820794817
CELSR2	0,579920964	3,684250681	0,52419195	0,820794817
EIF4EBP2	-0,250256452	8,372916826	0,524206105	0,820794817
AP3S2	-0,245676714	5,069313808	0,524463075	0,820910583
ATOX1	0,324116292	7,061696388	0,524520811	0,820910583
WBP4	0,413073228	4,189227432	0,524999016	0,821182066
EXOSC10	-0,247158185	6,800318023	0,525153399	0,821182066
CPE	-0,342828589	7,188378644	0,525330692	0,821182066
NUP85	0,37949317	4,588701195	0,525459033	0,821182066
PARP6	0,44819059	6,27527907	0,52549167	0,821182066
SKAP2	-0,283066759	5,625400479	0,525528084	0,821182066
ARL6IP5	-0,312161242	7,13689658	0,525606039	0,821182066
IFNAR1	-0,276003291	5,372071714	0,52565768	0,821182066
ADPGK	-0,252603342	5,456400828	0,526042037	0,821420211
NRARP	-0,352115312	7,139043905	0,526140845	0,821420211
TMEM33	-0,236525715	6,890302044	0,526171503	0,821420211
ZNF623	-0,292881121	4,445071686	0,526669795	0,821911372
EGLN2	0,508457885	6,80328838	0,526730503	0,821911372
SLC38A6	0,354788013	6,12227009	0,526847721	0,821911372
GMFB	-0,281377318	6,473348145	0,527221836	0,822306884
CLPTM1	-0,269926799	8,083937402	0,527649402	0,822638365
EIF3L	-0,236681419	8,91641486	0,52792883	0,822638365
FIS1	-0,262544738	6,786182296	0,528013097	0,822638365
KIAA1671	-0,322407947	4,58114317	0,528048042	0,822638365
DAXX	-0,312933736	4,820618895	0,528188741	0,822638365
LZTS2	0,436228093	6,829898029	0,528195706	0,822638365
EPC1	-0,258927737	4,653920126	0,528291714	0,822638365
RAB5B	-0,238603716	7,93875284	0,528447204	0,822638365
FAM98B	0,40985266	4,403935942	0,528529502	0,822638365
TCP1	0,305205376	7,111215139	0,528640756	0,822638365
RAB3GAP2	0,291118896	5,34179762	0,528836663	0,822755465
ABHD12	-0,273622245	6,965141005	0,529034508	0,822875527
FRRS1	-0,292508983	3,716564829	0,529293971	0,823091354
SUMO1	-0,271908878	3,961123754	0,529640509	0,823174124
CHMP2A	-0,271899339	7,53116905	0,529695835	0,823174124
POLR3K	0,35346759	5,244474411	0,529709349	0,823174124
SLC41A3	-0,311931811	4,768266053	0,530069649	0,823546352
ZNF177	-0,355625944	4,530820787	0,530341892	0,823710827
FBXW2	-0,237938432	6,206816516	0,530417106	0,823710827
NUCB2	-0,289696188	4,979406412	0,530676905	0,823801139
TMEM64	0,347457292	5,747292334	0,530821748	0,823801139

MFN2	0,305019179	7,267245542	0,530944361	0,823801139
MYO18A	-0,268967032	4,665700117	0,531038041	0,823801139
NBPF9	-0,31571557	5,975237363	0,531132495	0,823801139
CASC3	-0,31388304	7,69641356	0,531216753	0,823801139
SYNM	-0,372828312	5,490912624	0,53152348	0,823801139
CASP6	0,351767131	3,841459515	0,531575785	0,823801139
CSNK1A1	0,358146304	8,699675426	0,531755471	0,823801139
BRWD3	-0,25205682	4,032232741	0,531792653	0,823801139
PGLS	-0,314398603	5,910214375	0,531804167	0,823801139
SLC2A3	-0,254231959	7,900051468	0,532582694	0,824670299
GORASP1	0,409888401	5,25307754	0,532607127	0,824670299
ZKSCAN1	0,386337732	7,360194772	0,532738535	0,824673287
INPP5F	0,428481707	3,452973497	0,532850931	0,824673287
LUC7L2	0,301892769	6,228397467	0,533552245	0,825571309
PLIN2	-0,306152985	7,187983579	0,534125493	0,826270811
RPS6KA3	-0,222086685	6,548306015	0,534311423	0,826370967
DKFZP586I1420	0,447095758	6,813059177	0,534472701	0,826428435
IL6ST	-0,309222102	8,0106936	0,53459097	0,826428435
FBLIM1	-0,32926438	6,663594506	0,535139098	0,826976932
DNAJC16	0,345262797	4,831735107	0,535196783	0,826976932
TMED4	0,395933754	6,985785138	0,535400444	0,826976932
HTATSF1	0,341564417	4,5663582	0,535552011	0,826976932
TRO	0,724349294	5,129000708	0,535667773	0,826976932
OAS3	0,435092235	5,769432071	0,535738556	0,826976932
COG8	-0,250639215	5,891029147	0,535820033	0,826976932
RSAD2	0,453375277	4,128589663	0,535928803	0,826976932
GOLGA3	0,360464915	5,751637147	0,536052066	0,826976932
HDAC5	-0,289595535	5,93618771	0,536200647	0,826976932
OSGEPL1	-0,287059425	3,925406084	0,536279805	0,826976932
GNL2	0,317477682	6,170942412	0,536409274	0,826986437
C16orf52	-0,263742805	5,144329796	0,536528523	0,826986437
JAG2	0,488643925	5,721636074	0,537080316	0,827216702
CNN3	0,412902639	8,47869634	0,537095693	0,827216702
CD248	-0,361516706	7,191806529	0,537143746	0,827216702
RARRES3	-0,374901468	7,155937435	0,537283219	0,827216702
ADIPOR1	-0,226890765	6,404455604	0,537284466	0,827216702
YAP1	0,302903473	8,089477727	0,537720168	0,827655617
FIP1L1	0,298100335	5,166598393	0,537880618	0,827655617
PLEKHM2	-0,293998785	6,709794224	0,53793367	0,827655617
FAM120C	0,363465451	4,625945585	0,538085993	0,827703222
FAM108A1	-0,288184328	8,697116935	0,538335773	0,827786597
DCAF5	-0,259620173	5,405834015	0,538382982	0,827786597
NEDD1	0,367574641	4,938620626	0,539044203	0,827855516
RBX1	-0,229576014	7,174884933	0,539114898	0,827855516
TRMT12	0,425115049	5,036549389	0,539172179	0,827855516
RASA1	-0,303954695	6,888006976	0,539208466	0,827855516
RAF1	-0,294104948	4,578259913	0,539238728	0,827855516
UBE2D4	-0,256917833	5,208152511	0,539271565	0,827855516
DNAJC5	0,303857033	6,637289726	0,539277636	0,827855516
NINL	0,426542577	3,863701064	0,540144798	0,829000086

SFMBT1	-0,265117103	3,950725551	0,540416645	0,829134327
LOC387647	0,361723243	5,235811912	0,540475449	0,829134327
HEATR5A	-0,289623785	4,699396601	0,540909386	0,829332721
MAPK9	0,327131281	6,113339685	0,540957352	0,829332721
PSMD3	0,360672094	7,480541019	0,541066071	0,829332721
KDM2A	0,318755642	6,955206295	0,541136985	0,829332721
SND1	0,44254678	6,075698656	0,541212877	0,829332721
CHPF2	0,470644864	5,568259832	0,541358312	0,829369204
PDZD8	0,382158464	5,568859382	0,541579037	0,829520991
OCIAD1	0,349240966	6,155447142	0,541952376	0,82986904
MCFD2	0,338305397	6,697312177	0,542049671	0,82986904
VCAN	0,545506474	6,598308856	0,542327249	0,830107634
TRIM56	-0,27123287	4,728226219	0,542647762	0,830411824
RASSF4	-0,274259807	6,671309821	0,542947393	0,83068393
USF2	0,322973979	7,651411251	0,543500092	0,831177144
MRPL38	-0,216058778	6,927084248	0,543513548	0,831177144
TMEM123	0,31832088	8,987638181	0,543711647	0,831293659
ZNF180	-0,256987793	3,022740455	0,544411786	0,832168143
RPL24	-0,288912505	8,13439531	0,544527681	0,832168143
ASS1	-0,277158049	5,013632622	0,544805136	0,832405607
NKAP	0,345709651	4,796427791	0,54523761	0,832879763
ARPC5	-0,225025295	8,140213009	0,545466645	0,833043012
OSGIN2	-0,278951195	4,641249603	0,545785634	0,833175623
KHNYN	-0,258059535	5,25784033	0,545797846	0,833175623
HADHB	-0,238843191	7,537660674	0,546344406	0,8338233
CDYL	0,365940799	4,703421069	0,546953346	0,834356357
NDUFB10	-0,253373163	5,144803352	0,546994243	0,834356357
VASN	-0,300382213	6,118083132	0,547176349	0,834356357
CLASP1	-0,278917106	6,586530377	0,547195361	0,834356357
ESYT2	0,334873626	8,876262091	0,547342595	0,834356357
EHHADH	0,352495049	4,104105645	0,547427826	0,834356357
FAM45A	0,340668557	5,582480463	0,547817105	0,834492367
FBXW5	-0,251840103	6,198099632	0,548023071	0,834492367
GEMIN6	0,387612125	4,247327932	0,548036944	0,834492367
RAB4B	-0,26217261	4,880038934	0,548075902	0,834492367
HIPK2	0,411224191	7,318306765	0,54825324	0,834492367
ACPL2	0,513077504	5,681384712	0,548344174	0,834492367
FBXL5	-0,232282331	7,762353728	0,548373706	0,834492367
ACACA	-0,248469058	5,534266066	0,548602854	0,83465481
OPN3	-0,344785869	6,476412651	0,548732559	0,83466592
WDR35	0,408457527	5,72379808	0,549580985	0,835665338
NGDN	0,314794372	5,325596504	0,549634703	0,835665338
SSBP3	-0,249427039	6,837392392	0,550045083	0,836042145
UBAP2	0,289226458	6,280996466	0,550342949	0,836042145
CREB3L1	-0,322750468	4,586512048	0,550621912	0,836042145
C17orf49	-0,245291693	5,430341792	0,550827677	0,836042145
KDM4A	-0,241338131	4,675626629	0,550877516	0,836042145
CNTROB	0,59176101	5,572601919	0,550933675	0,836042145
C11orf10	0,376689086	8,492961912	0,550994796	0,836042145
TRAF3IP1	0,321212108	5,544976149	0,551073994	0,836042145

LAS1L	0,349293611	5,367598892	0,551159048	0,836042145
RPS15	-0,258470924	10,48581374	0,55119872	0,836042145
TIMM13	0,41960557	5,186768524	0,551252417	0,836042145
RNF141	0,31574291	5,477825911	0,551353794	0,836042145
ETFB	-0,304928342	4,727805191	0,551570779	0,836099999
PRMT7	-0,23992246	5,417890317	0,551672641	0,836099999
STK17B	-0,263179438	6,246256889	0,551759788	0,836099999
ZC3H18	0,277803842	5,541217458	0,552178523	0,836284312
TRIM8	0,361181227	7,964509473	0,552194812	0,836284312
UBA1	-0,245447152	7,780065259	0,552249341	0,836284312
ODF2	0,302580073	4,013519318	0,552816229	0,836956897
MAPK14	0,275907381	6,781199516	0,553264722	0,837449975
FAM198B	0,314716806	7,022868052	0,553393174	0,837458512
MED18	-0,261348835	3,998682807	0,55383959	0,837689491
BCAP29	0,344544909	7,055549606	0,553922276	0,837689491
CRELD2	-0,248727794	6,765440902	0,553981476	0,837689491
CD3G	0,589497129	5,765245258	0,554143069	0,837689491
PBXIP1	-0,277584481	6,747584382	0,554197135	0,837689491
S100A6	-0,283755286	8,174877764	0,554451308	0,837689491
PMPCA	-0,274813814	4,903396541	0,554769995	0,837689491
ZNF706	-0,250343628	5,733105943	0,554861437	0,837689491
SNX8	0,396756874	5,747081413	0,554950674	0,837689491
RFT1	0,377574736	5,573276533	0,555066195	0,837689491
SDC4	-0,28943305	8,524992567	0,555121201	0,837689491
MYH9	-0,250422682	9,218479988	0,555185929	0,837689491
SKIV2L	0,400895166	3,392372791	0,555189425	0,837689491
ZNF573	0,41484244	4,413271556	0,555265655	0,837689491
LMAN2	-0,245540758	7,169795123	0,556684384	0,839644064
CD164	-0,255275074	8,357326467	0,556988801	0,839873855
TRIM41	0,395652872	4,297142325	0,557083069	0,839873855
RPL35	0,308558511	11,3225254	0,557250693	0,839940866
ORMDL3	-0,285282576	6,434511679	0,55758767	0,840226066
GAS5	0,32593226	7,238192615	0,557686343	0,840226066
ARID5B	0,3435683	5,846522053	0,55788568	0,840336143
C1orf174	0,289597722	4,951554842	0,558005874	0,840336143
NRBP2	0,49337819	5,434024656	0,558204151	0,840449129
SFXN4	-0,259920961	4,463862293	0,558356814	0,840493403
INO80D	-0,231790316	6,262704999	0,55878772	0,840694869
LOC93622	0,530725803	5,209117289	0,558794773	0,840694869
ECE1	-0,266417917	7,050269888	0,558860513	0,840694869
NOC2L	0,281898284	5,876824918	0,559079448	0,840838719
YPEL5	-0,259330201	7,987607921	0,55961867	0,841464104
NDUFA4L2	-0,415290276	8,755354331	0,560021181	0,841883693
ZNF17	0,448711918	4,868795011	0,560257907	0,842053927
MAPKAPK2	-0,234297383	6,535319821	0,560525174	0,842136701
ATXN3	0,320963763	5,373659641	0,560846038	0,842136701
PARP11	-0,272700478	4,755065924	0,560888617	0,842136701
MTHFR	-0,263569674	5,402689589	0,56089406	0,842136701
TMEM39A	0,266505309	5,580582565	0,560930473	0,842136701
FASTKD3	0,397270803	3,954850523	0,561188239	0,842338234

SLC35E1	-0,246125794	7,030598603	0,561517106	0,842646378
HDHD2	-0,235155802	4,960372621	0,561829583	0,842831103
OTUD4	0,399806224	6,108857313	0,561945158	0,842831103
SPATA7	-0,271124689	4,443324611	0,562214864	0,842831103
ERI1	0,382071613	5,243988247	0,562280828	0,842831103
HNRNPM	0,293329275	7,275923738	0,562312307	0,842831103
LIX1L	-0,259197755	6,274729544	0,562381804	0,842831103
SGMS1	-0,247407683	5,718608106	0,563015751	0,843541809
AMIGO3	0,461722584	5,776136987	0,563103434	0,843541809
COX8A	0,316316806	7,679187823	0,563619302	0,84412915
PTK2	-0,249009503	6,093975914	0,563785949	0,844193322
SEC63	0,310244224	7,175750427	0,564082446	0,844290685
GFM1	0,315335354	6,25741541	0,564098601	0,844290685
ASL	0,38769567	6,248997428	0,564533871	0,84461882
MED16	0,358818431	6,770803891	0,564651408	0,84461882
KPNA3	0,314909246	6,463746353	0,564724937	0,84461882
FYTTD1	-0,319745404	5,600147368	0,56487773	0,84461882
DCAF10	0,285750515	5,482482336	0,565123935	0,84461882
C1orf21	-0,25211859	5,792864922	0,565143375	0,84461882
SRBD1	-0,216057152	5,444314545	0,565252421	0,84461882
WDR20	0,323146406	5,168160214	0,56530874	0,84461882
PPP1R16A	0,406628058	5,134620181	0,565685721	0,844863898
MAP2K2	0,317956185	5,681603825	0,56578556	0,844863898
RBCK1	0,441540381	6,335485197	0,566040164	0,844863898
DBT	-0,233548804	4,017557888	0,566058373	0,844863898
C9orf91	-0,278510843	5,376358359	0,566092265	0,844863898
NCOA3	-0,204516453	6,276846276	0,566526747	0,845327328
TMEM120B	-0,244357345	4,535691931	0,566905658	0,845601237
RASA4	0,553551061	7,388330017	0,566958331	0,845601237
TYK2	0,298771516	7,292514482	0,567198454	0,845774384
EPS15	-0,207213305	6,682697441	0,567690254	0,846252767
TRIP4	-0,206277693	4,782378082	0,567767475	0,846252767
GDE1	-0,216068371	6,06987317	0,568059043	0,846397024
ANO6	-0,251504777	7,2327921	0,568253342	0,846397024
EYA3	0,370950107	6,201046079	0,568395162	0,846397024
PDLIM5	-0,258702387	6,999533933	0,568474884	0,846397024
USP21	0,29992662	4,416448644	0,568558416	0,846397024
HPS1	-0,271081647	4,837615079	0,568608999	0,846397024
CUX1	-0,295742853	5,435835564	0,568748156	0,846419396
PLXNB2	-0,26263958	7,771653039	0,569174586	0,846869191
TET3	-0,240574292	4,442525015	0,569465099	0,847000596
NAA25	0,305111508	5,57156225	0,569511326	0,847000596
MRPL46	-0,247005201	5,234683813	0,570560614	0,848211377
RIF1	0,331954092	6,507046899	0,570574217	0,848211377
PANX1	0,33802529	4,604780971	0,570700095	0,84821359
MGEA5	-0,210230045	7,476993031	0,570906399	0,848261652
SMG6	-0,2417073	5,076363979	0,570981226	0,848261652
KLHL2	0,302429846	5,825043967	0,571106339	0,848262715
CORO1B	-0,202383826	6,864972182	0,571284887	0,848343129
LACTB	0,333572197	5,237094542	0,571565477	0,848434032

CXorf26	0,289736679	5,812152912	0,571594947	0,848434032
SRCAP	0,282845205	6,860831359	0,571792964	0,848543247
BRD2	0,28456884	8,021941127	0,572051419	0,848742086
SPCS3	-0,255624665	7,012225806	0,572432729	0,849017876
NCL	0,44927508	7,538160418	0,57260072	0,849017876
IER5	0,329907351	7,262301361	0,572610825	0,849017876
LGALS1	0,406719659	9,626098584	0,573064523	0,849372745
MGC2752	-0,229656243	4,623947786	0,573099281	0,849372745
GOLGA8B	-0,350131147	6,038439027	0,573272554	0,849444925
PPHLN1	0,360937703	4,280159565	0,57362841	0,849621117
ALKBH3	-0,240080801	3,920288229	0,573640655	0,849621117
HS2ST1	0,316141871	6,005143303	0,573966916	0,849742433
IGF2R	-0,193004543	7,606829216	0,573971791	0,849742433
POP7	0,287531074	5,128511871	0,574233	0,849944612
MRPL40	0,314090474	5,116766732	0,574358757	0,84994626
NR2C1	-0,309096317	4,133305022	0,574704952	0,850122396
EIF2B1	0,291290753	6,517002809	0,574837117	0,850122396
NAB1	0,331002009	6,153092444	0,574970109	0,850122396
E2F6	0,336189425	5,210556496	0,575020945	0,850122396
KLF5	0,443514362	5,980330952	0,575229502	0,850122396
TRAFD1	-0,240445049	5,669824346	0,575310558	0,850122396
SLC16A4	-0,300559965	5,336822561	0,575350471	0,850122396
ZDHHC8	0,347111851	5,621386746	0,575830595	0,850183957
RIC8A	-0,230884442	5,615378318	0,575890128	0,850183957
EPHA2	0,411917894	6,524351548	0,575905754	0,850183957
PFKFB3	0,352286884	6,894421076	0,575941085	0,850183957
SLC25A39	0,361843066	6,620723666	0,576479828	0,850183957
THADA	-0,225753122	4,574923391	0,57658268	0,850183957
RASA2	-0,243773373	3,70528688	0,576781416	0,850183957
EARS2	0,364163929	4,970319098	0,576808067	0,850183957
ATP6V1H	-0,252271415	5,035182396	0,576883792	0,850183957
EPN2	0,327352417	7,101510192	0,576940113	0,850183957
ZNF101	0,322326929	3,855079031	0,576965306	0,850183957
RNASEH2C	0,319428034	5,666691299	0,577161274	0,850183957
ARHGAP29	-0,321183398	6,529933108	0,577424892	0,850183957
COX6C	-0,267947055	6,587442484	0,577515768	0,850183957
STEAP2	0,36614412	6,957917393	0,577518749	0,850183957
SEC22C	-0,23964063	4,339894883	0,577773413	0,850183957
YTHDC1	0,284086214	6,840610275	0,577789139	0,850183957
PTGR2	0,361286419	4,986099956	0,577824005	0,850183957
WDR77	0,29272508	6,245045078	0,577840236	0,850183957
FSTL1	0,45320826	9,417595031	0,57798425	0,850183957
KIAA1586	0,451972002	5,313615467	0,578010386	0,850183957
PKD1	0,463068062	7,937443472	0,578148383	0,850203542
MAP4K2	-0,285676075	4,553806826	0,578340615	0,850302858
MAVS	-0,214637124	6,474614681	0,578466122	0,85030405
SSR2	-0,254464073	7,116520041	0,578841494	0,850517361
CIC	-0,231860169	7,181874706	0,578860694	0,850517361
PIGT	-0,270957714	7,112738928	0,579244425	0,85071635
IMPA1	0,282193745	5,053724622	0,579421856	0,85071635

TRUB1	0,293609398	4,904964557	0,57944588	0,85071635
POLI	-0,226622339	4,103128871	0,579495153	0,85071635
DHPS	0,340480029	5,242671539	0,579911875	0,851113404
NUDT16L1	0,439923201	4,290182138	0,58001525	0,851113404
BLOC1S2	-0,240133016	5,348484427	0,580512723	0,851660124
SMAD6	-0,344773101	5,806456759	0,580809737	0,851682879
F8A1	-0,273019446	5,259224815	0,580917262	0,851682879
C14orf93	-0,240560696	4,321617921	0,581103898	0,851682879
AGAP6	-0,26206651	5,212960894	0,581118341	0,851682879
MLLT6	0,429108627	6,359133063	0,581152726	0,851682879
ZNF428	-0,285532917	4,633950157	0,58132753	0,851756
TIMM17A	0,343145487	5,805153529	0,581715656	0,85214158
AP4S1	-0,267209576	4,950223166	0,581877003	0,852158377
CMTM8	0,458239895	6,173832377	0,581977058	0,852158377
KIF21A	0,385119474	3,998838749	0,582304967	0,852455468
PDPK1	0,357434222	4,528763262	0,582574038	0,852547106
MYO10	-0,246263327	6,828017749	0,58281935	0,852547106
SPINT2	0,467621703	9,018275692	0,58297025	0,852547106
TMEM115	-0,271815059	5,739558797	0,583013543	0,852547106
HERC2	0,387920059	7,825219037	0,58304229	0,852547106
ATAD2B	-0,231818784	3,527638618	0,583299696	0,852547106
TMED1	0,370107881	5,106445742	0,583365101	0,852547106
EDF1	0,290686407	7,506003495	0,583521226	0,852547106
DBI	-0,238916336	8,340183858	0,583596903	0,852547106
PRMT2	-0,24282546	4,606740528	0,58370894	0,852547106
HYI	-0,225523927	6,3097767	0,583955703	0,852547106
MTHFD1L	0,385617412	4,584772416	0,584044061	0,852547106
ANKRD40	0,312179902	7,147838704	0,584095642	0,852547106
ULK3	0,372874331	5,962003046	0,584117917	0,852547106
LCORL	0,348284505	5,929660151	0,584282288	0,85260452
DCAF17	0,391983728	4,050237392	0,584497377	0,852637684
NKIRAS1	0,394604668	3,215725442	0,584555092	0,852637684
MRPS18A	0,333344747	5,168955898	0,584808722	0,852801679
UQCR10	0,34795339	5,763514469	0,584917649	0,852801679
SUMF1	-0,248747777	4,988405734	0,585145107	0,852950938
ZNF318	0,299378806	4,25412323	0,585291722	0,852982314
AP2A1	-0,21550915	6,776974541	0,585605971	0,853257931
IGF1R	-0,22505048	6,680288948	0,586009197	0,853563187
EIF2B4	-0,216351739	5,139364616	0,586089193	0,853563187
KIAA0240	-0,221332788	4,83971319	0,58631617	0,853563187
HS1BP3	-0,234571503	4,463679304	0,586380907	0,853563187
ZNF280D	-0,232537264	5,336892392	0,586441345	0,853563187
APBB1	-0,247222124	6,26798429	0,586707995	0,853631193
TTC8	0,328599491	3,648909481	0,586866695	0,853631193
ACAT2	-0,246002806	4,323542962	0,587139954	0,853631193
C6orf1	0,389586295	6,575317655	0,587144233	0,853631193
PIP4K2A	-0,195865823	5,845573789	0,587233943	0,853631193
RBM3	0,308542206	8,829712801	0,587239174	0,853631193
CCDC104	0,322067883	6,083039579	0,587670102	0,853888426
COPA	-0,187217022	7,415081994	0,587743971	0,853888426

PRPSAP1	0,307221451	5,355643988	0,587791798	0,853888426
ARCN1	-0,197911145	8,139090617	0,58798677	0,853951378
CCT7	0,264716828	6,363993633	0,588085595	0,853951378
STX8	0,340983373	6,291034619	0,588335353	0,853991955
HIP1	-0,270541035	5,636617889	0,588769303	0,853991955
HINT2	-0,271449365	5,7654149	0,588859486	0,853991955
SETD1A	0,323202524	6,16231425	0,589208585	0,853991955
PIBF1	0,308656241	3,494729917	0,589300424	0,853991955
CALD1	-0,255897533	8,793683104	0,589374862	0,853991955
COG4	0,286404152	5,023471695	0,589476823	0,853991955
GPC4	-0,332998185	5,773361042	0,589590432	0,853991955
MYO9B	-0,250922618	5,727870032	0,589613801	0,853991955
GALNT4	0,363692225	4,698019426	0,589637832	0,853991955
MRP63	-0,258620277	3,922006674	0,589890453	0,853991955
PSMD9	0,294466307	4,844083257	0,589911637	0,853991955
SNX5	0,268738846	6,764762952	0,590002131	0,853991955
LEPROT	-0,231688144	7,846733271	0,590011631	0,853991955
MAF1	-0,211617404	6,725145035	0,59010637	0,853991955
ADAMTS12	0,434789337	5,270231881	0,590117333	0,853991955
SMC5	-0,213023564	6,218821388	0,590336556	0,85400123
LOC401397	0,351223837	6,818103538	0,590417682	0,85400123
SNRPD3	0,270031501	7,492869581	0,590653539	0,85400123
PIGH	-0,218187606	4,957904388	0,590683839	0,85400123
TRIM24	-0,229412738	5,012328127	0,590749934	0,85400123
DPH1	-0,218927496	4,745415745	0,591121239	0,854356873
NDUFS2	0,288779028	6,682048826	0,591414498	0,854599589
TMCO1	0,236039218	7,240230432	0,591760462	0,85467564
EHBP1L1	0,261783686	7,86139042	0,591829389	0,85467564
PLEKHG3	0,310148717	6,089021665	0,59184314	0,85467564
RB1CC1	0,301494233	5,723565883	0,592293045	0,854974172
NDST2	-0,216844709	6,261083304	0,592349256	0,854974172
MRPL4	0,340712635	3,807193676	0,59242601	0,854974172
ZMYM2	0,33685981	7,438971073	0,592844733	0,855397426
INPP5B	-0,262639487	5,144425185	0,593592024	0,856294481
KIRREL	0,31057574	6,91599063	0,594015166	0,85672365
YTHDF2	-0,205380192	6,235174384	0,594423006	0,857130573
DSEL	-0,304752444	5,360188473	0,594594897	0,85715278
RBM4	0,335326934	6,152655801	0,594689808	0,85715278
NR3C1	-0,220653885	7,21531421	0,594848849	0,857200824
CRYZL1	-0,226404555	4,835327279	0,595295802	0,8576295
VPS35	0,309046031	6,738708026	0,595399909	0,8576295
DNAJB9	-0,24815595	5,272790834	0,595523637	0,8576295
GRB10	-0,258556154	7,783520179	0,596116906	0,858001995
GALNS	0,354738967	5,633590849	0,596117758	0,858001995
GABPB2	0,292315788	4,169035096	0,596238102	0,858001995
PDIA3	0,251207786	8,701477934	0,596285592	0,858001995
C16orf58	-0,210966513	4,227860014	0,597097282	0,858841422
WDYHV1	0,374470936	4,093479224	0,597250255	0,858841422
SWAP70	0,281690616	6,782879116	0,597338072	0,858841422
COMMD1	-0,186324598	5,945352193	0,597415775	0,858841422

DPH3	0,38146552	5,202369495	0,597514377	0,858841422
CSNK1G2	-0,20835143	6,243246858	0,597624659	0,858841422
NEURL4	0,337603485	4,386544425	0,598107763	0,859283483
MAPK8IP3	0,414814328	5,628003509	0,598184293	0,859283483
ARF3	-0,19011961	7,698324052	0,598691206	0,859776444
TTC14	-0,28243232	6,602821231	0,598779635	0,859776444
IFT122	-0,2589529	5,150970022	0,599197323	0,860195063
ZNF697	-0,220423369	4,397846519	0,599546738	0,860515514
FBXO6	-0,239278167	4,604442178	0,6005523	0,861777384
C8orf59	-0,239080026	5,649850078	0,601133355	0,862429696
UBE2D1	0,348616953	6,359273355	0,601263416	0,862434532
BUD31	0,280556402	6,069545876	0,601389676	0,862434532
ERLEC1	0,327853499	6,340794022	0,601594667	0,862547105
ANAPC10	0,372828302	4,98194968	0,601908142	0,86281514
FOXO3B	-0,226482388	5,115580302	0,602331922	0,863209453
APOA1BP	0,285726281	5,942547578	0,602674284	0,863209453
VPS36	-0,228739307	6,130329031	0,602675552	0,863209453
TRNAU1AP	0,27036934	4,38215078	0,60270633	0,863209453
UTP14C	-0,223372224	4,231369173	0,602816163	0,863209453
SMU1	-0,181590076	6,607901645	0,603172832	0,863432109
KDM5A	0,252339588	6,197747703	0,603243764	0,863432109
PDE4DIP	-0,272144006	5,61085781	0,603459914	0,863432109
KCTD10	-0,219040943	5,452920802	0,603603606	0,863432109
NT5C3L	-0,25543054	5,103371032	0,603622631	0,863432109
CLIP4	0,31070805	5,993647558	0,603731382	0,863432109
PIK3CA	0,293482176	5,484303494	0,604346253	0,863535209
ZDHHC4	0,321617259	5,57514174	0,604387508	0,863535209
NUP93	0,29265765	6,004105896	0,604423991	0,863535209
IGFBP4	-0,271190925	7,978795006	0,604439941	0,863535209
PPARD	0,257370216	6,010336419	0,60446885	0,863535209
ARF5	0,339075338	6,928600303	0,604667326	0,863535209
AAMP	-0,174545058	5,458847621	0,604689929	0,863535209
GTF2F1	0,29234753	5,77933868	0,604820485	0,863540805
PHPT1	0,362006471	7,291624203	0,60497706	0,86358354
ATP1B1	-0,245507688	7,787145105	0,60537868	0,863643359
MYCBP2	-0,199404715	6,59636167	0,605392431	0,863643359
LOC550643	0,269311165	5,896160373	0,605624714	0,863643359
ADD3	0,473727948	5,45791519	0,605736095	0,863643359
MLL	-0,237551428	6,667209273	0,605974632	0,863643359
SEPT2	-0,182013574	8,206518985	0,606074769	0,863643359
AXIN1	0,336982609	5,53335189	0,606238578	0,863643359
PLAGL2	0,393371274	6,000389595	0,606267078	0,863643359
ATP8B2	0,478614467	5,795426346	0,606306146	0,863643359
KCNQ1OT1	-0,233120591	8,762496892	0,606536513	0,863643359
PCID2	0,278810351	5,0245091	0,606626941	0,863643359
GTF3C3	0,282703332	4,629718582	0,606729589	0,863643359
HIF1AN	-0,204193409	6,322953619	0,606857035	0,863643359
CCNG1	-0,227783405	5,78474882	0,606866715	0,863643359
PRPF18	0,362385487	4,575309595	0,607049296	0,863643359
C16orf13	-0,23293456	4,757212222	0,607132018	0,863643359

SURF4	-0,202360532	7,158605447	0,607172058	0,863643359
ATXN7L3B	0,294839508	7,526060119	0,607300015	0,863645214
C7orf60	0,348862619	4,334401123	0,60826805	0,864791901
MIA3	0,355536637	6,072412407	0,608578417	0,864791901
RPL8	-0,260680078	7,800321247	0,608581819	0,864791901
BTAF1	-0,198592241	7,076058149	0,608613628	0,864791901
ARPC4	-0,177608484	7,238795144	0,608752431	0,864808922
ZNF625	0,401973734	3,41079821	0,609030636	0,865023934
GAN	0,387645061	5,010743722	0,609421741	0,865399177
NOP2	0,375667388	4,99499411	0,609847619	0,865723438
PITPNC1	-0,204624073	5,298741665	0,609994496	0,865723438
RABGAP1	0,341525296	5,354348652	0,610070136	0,865723438
ZNF581	0,299049469	5,658299488	0,610157918	0,865723438
TK2	-0,214518259	5,270854234	0,61062621	0,86620764
HARS2	0,318166572	4,166954368	0,610761426	0,866219252
WASL	-0,196555496	5,486461067	0,610995754	0,866371396
ZNF410	0,269905325	5,881918435	0,611683002	0,86716557
CEP192	-0,230429887	5,281114747	0,612084906	0,867292218
SLC10A3	0,301348359	5,74806256	0,612108683	0,867292218
GATAD2A	0,283674027	7,711230669	0,612268193	0,867292218
MARS	0,225158311	7,568261253	0,612295226	0,867292218
ZNF81	0,363355143	4,231705657	0,612408276	0,867292218
ABCD3	0,280404562	6,270470007	0,612623375	0,867416694
HOOK2	-0,222189202	4,944493628	0,612782124	0,867461346
BRWD1	-0,21035397	6,079935773	0,613576727	0,868381969
TATDN3	-0,235717362	4,759689262	0,613718582	0,868381969
ARMC7	-0,258154749	4,577582695	0,613932183	0,868381969
PPM1K	-0,243591523	5,149026955	0,613941849	0,868381969
UCKL1	-0,199771189	5,93821256	0,6141224	0,868402888
MAT2B	-0,184814432	7,304630332	0,61421134	0,868402888
AMFR	-0,187572474	6,381533645	0,61440393	0,868495107
PEX26	-0,209097892	5,435561142	0,614985268	0,869080982
WASF1	0,332343907	4,629929202	0,615073298	0,869080982
RWDD2B	-0,220769083	3,001256374	0,615285858	0,869095769
ZSCAN29	0,332914312	4,026353984	0,615503682	0,869095769
YTHDF3	-0,190658637	4,831149426	0,615512052	0,869095769
TRIM47	-0,190797558	3,790391591	0,615593571	0,869095769
RPS8	0,250338708	11,09305345	0,615837836	0,869260651
PIN1	-0,211310798	5,235157074	0,616268608	0,869369994
MRPL50	0,258055901	6,369486496	0,616273217	0,869369994
AUP1	0,226907024	7,803817494	0,616628104	0,869369994
DCAF12	-0,229829869	4,801197529	0,616739159	0,869369994
YY1	0,239170775	7,866492782	0,61674713	0,869369994
NUDT15	0,253598156	4,561557901	0,616787349	0,869369994
SETD2	-0,22739381	6,647825702	0,616853128	0,869369994
GIT2	-0,191924124	4,763371909	0,61693524	0,869369994
GIPC1	0,306818927	6,049007179	0,617208482	0,869460857
NAA10	0,320189793	5,476098141	0,617321935	0,869460857
RAB3D	-0,235102254	5,406607143	0,617475505	0,869460857
FPGS	-0,186649046	6,217102369	0,617612769	0,869460857

SGK3	-0,212285816	4,2341357	0,617883806	0,869460857
LRRC8D	0,320191081	4,882630095	0,618199275	0,869460857
ZNF154	0,387372028	4,092350419	0,618242862	0,869460857
TMEM87B	-0,2551786	5,586711693	0,618277906	0,869460857
PRMT3	0,264130238	4,780289398	0,618301735	0,869460857
LEMD2	0,250014347	5,460622578	0,618392536	0,869460857
PSMB6	0,296286919	6,950703236	0,618761478	0,869460857
SEPT8	-0,21289865	6,224447441	0,618789795	0,869460857
UROD	-0,190374642	5,123782771	0,618859114	0,869460857
MLX	-0,183786906	5,896294016	0,61907669	0,869460857
THY1	-0,331092463	8,014824655	0,619107491	0,869460857
EVI5L	-0,23607591	4,843226906	0,619141258	0,869460857
WAC	-0,212834815	8,207196866	0,619167315	0,869460857
BCL7B	0,26103758	6,078603691	0,619457322	0,869641815
CCNT1	-0,21405578	5,002739651	0,619551245	0,869641815
DRAM2	-0,226485562	4,427362227	0,619822415	0,869843393
ARPC2	0,239530942	10,15924256	0,620227781	0,870066752
NDUFA5	0,292266038	6,191501596	0,620236762	0,870066752
LRRC47	-0,195073074	5,724501428	0,620683363	0,87037557
BOK	0,34842818	4,136408378	0,620712186	0,87037557
DYNLT3	0,380182377	6,40280717	0,62092666	0,870497306
C17orf62	0,337997206	5,687037114	0,621112545	0,870578919
WDR11	-0,193599661	6,062106942	0,621538573	0,870692485
GBA	0,233272019	6,324000893	0,621565717	0,870692485
DPY19L3	-0,23313029	6,108538261	0,621576626	0,870692485
COPZ1	-0,181723407	6,61172137	0,621963078	0,870749963
EFHD2	0,332247719	8,195865346	0,622072715	0,870749963
ITFG3	-0,184155596	6,881421637	0,622076416	0,870749963
NEO1	0,388697117	7,312918436	0,622128438	0,870749963
PATZ1	-0,248059782	4,083629361	0,622448263	0,870777116
RGS1	-0,265261097	7,113804444	0,622463814	0,870777116
C1orf131	0,400786154	4,161810771	0,62255091	0,870777116
YIPF3	0,273963989	8,120944645	0,622658633	0,870777116
RASA3	0,295117969	5,394748274	0,623087423	0,8711981
ACTB	-0,2086504	13,33320356	0,623354131	0,87124911
TSC22D4	-0,185608887	6,281520044	0,623379441	0,87124911
NUTF2	0,24137814	7,151370323	0,623707109	0,871528438
SRF	-0,195011862	6,733733393	0,624046371	0,871556253
MSH5	0,308211043	4,927480755	0,624079332	0,871556253
PAIP2	-0,192671408	5,956866259	0,624203186	0,871556253
VAC14	0,253026995	6,822735446	0,62434361	0,871556253
PIK3C3	-0,176012018	4,409129225	0,624373795	0,871556253
FAM129B	0,289469305	7,857144162	0,624581891	0,871556253
GNB2L1	0,284692812	11,01028335	0,624621705	0,871556253
DNMT1	-0,244632329	5,245522327	0,625073048	0,871749046
DCPS	0,29596345	5,979848232	0,625128529	0,871749046
TSEN54	0,309939903	5,09337124	0,62526832	0,871749046
FLAD1	0,324408476	3,667109841	0,62527124	0,871749046
TSPYL4	-0,20611665	5,191365739	0,625634486	0,871957605
NT5DC2	0,321120253	7,581295605	0,625676575	0,871957605

SDHB	-0,21494361	3,663028975	0,626192649	0,872498503
GFER	0,247005571	4,998924306	0,626433197	0,872655357
PCYT1A	0,333752502	5,974750715	0,627156273	0,873258257
FLCN	-0,264264493	4,792446382	0,62729838	0,873258257
LRRFIP1	0,33805601	7,887191915	0,627353012	0,873258257
SAMD1	0,280052337	5,259589012	0,627378237	0,873258257
CMTM6	0,272545734	6,592754709	0,627551484	0,873321136
TEX261	0,293500626	7,089343705	0,627838613	0,873483893
HNRNPA2B1	0,269218919	9,753947716	0,6281792	0,873483893
WDR5B	0,364488802	6,133016512	0,628230917	0,873483893
PSMG4	-0,194684338	5,664769761	0,628328794	0,873483893
RCOR1	0,231563932	6,331966422	0,628464452	0,873483893
TSFM	0,27689351	3,468084773	0,628544244	0,873483893
ABCC1	0,34322036	6,810267589	0,628641995	0,873483893
NDOR1	0,405994304	4,335777902	0,628723202	0,873483893
KANK1	-0,268800628	5,491778877	0,628821298	0,873483893
AKAP8L	-0,195002276	5,467289394	0,629106719	0,873702387
EXOG	0,333723855	4,200062241	0,629483239	0,873941017
KLHL24	-0,246530839	5,234842237	0,629563058	0,873941017
NRD1	0,251213678	5,687567788	0,629663033	0,873941017
TYW1	-0,191949605	4,191646377	0,629819097	0,873979736
KCTD2	-0,174961924	5,218059843	0,630076351	0,874079419
RBBP4	0,25858883	7,550230037	0,630147298	0,874079419
FMNL1	-0,199090844	5,469323462	0,63135754	0,875580042
NHLRC2	-0,197674354	4,36173493	0,6321797	0,876528283
IDE	0,302322212	5,62843577	0,632298376	0,876528283
MEGF9	-0,24526331	6,351209946	0,632430192	0,876532821
ADH5	-0,178769505	7,621676665	0,632828071	0,876906038
OGDH	-0,224015354	5,83695048	0,633136725	0,877155491
PARK7	0,206399006	8,158021054	0,633895607	0,87789243
FBXL18	0,336430349	5,404397224	0,634150853	0,87789243
NAP1L4	0,294934665	6,375077025	0,634183329	0,87789243
MCCC2	-0,162675871	6,041608095	0,634183621	0,87789243
TUT1	0,274899113	6,492921111	0,634597771	0,878287437
HERC1	-0,201828522	5,779708228	0,635034922	0,878629387
BNIP3L	-0,214110315	8,263126931	0,635102544	0,878629387
C2orf69	0,338979507	4,760472007	0,635270549	0,878668838
ZMYM4	-0,197054923	7,101908942	0,635388773	0,878668838
TATDN2	0,289873982	6,104865277	0,636053032	0,879409088
SELT	-0,227826709	5,830541828	0,636534491	0,879820996
JAG1	0,47655393	6,441963319	0,636634033	0,879820996
TRAF4	0,289674452	5,609765092	0,63676184	0,879820996
WSB1	-0,196898251	5,588462054	0,636876125	0,879820996
CCDC25	0,292833687	4,990797668	0,636996078	0,879820996
FNBP1	0,289832221	6,267512208	0,637206563	0,879933486
XPNPEP1	-0,192226626	4,758220257	0,637575366	0,880208926
HEATR1	0,284765358	5,472830513	0,637664188	0,880208926
UBE2A	0,289204585	6,334716392	0,638083937	0,880610072
SPOPL	0,327471168	5,061240256	0,638300419	0,880612105
MKNK2	-0,217945258	7,713594955	0,63836277	0,880612105

UQCRC2	0,211598382	6,21022309	0,638472833	0,880612105
ZNF430	0,346506861	5,193239016	0,639011001	0,881176141
STK19	0,33287776	4,688852088	0,63925246	0,881312083
RPS6KB2	0,264328568	4,551726045	0,639477216	0,881312083
TTC28	-0,225519706	4,580240984	0,639553632	0,881312083
OR2A9P	-0,254588176	4,393631349	0,639626558	0,881312083
FANCF	0,379145404	4,383577085	0,639837253	0,881424289
FBXL20	-0,196773274	4,601481509	0,64027503	0,88171923
RBM42	-0,214447612	6,007509646	0,640340202	0,88171923
RAB1B	0,254352396	7,692083683	0,640463098	0,88171923
ANXA1	-0,20939638	9,05259062	0,64060706	0,88171923
SNX18	-0,206364114	5,808198868	0,640697872	0,88171923
KPNA6	0,223201753	6,430778194	0,640877958	0,881789104
IDH3A	-0,17424102	5,442843944	0,641363147	0,882208428
TRIM14	-0,208455998	5,814741259	0,64144147	0,882208428
NUFIP2	-0,159286877	6,703646495	0,642045126	0,882724879
TGFBR1	-0,199421609	5,947235633	0,642075876	0,882724879
TAF1C	0,342436115	3,88328864	0,642298743	0,882840322
CCNT2	0,233164635	4,637340881	0,642418782	0,882840322
KIAA1143	0,289791256	6,904310702	0,642944028	0,882876303
TRIAP1	0,296123215	4,392979686	0,643030522	0,882876303
STARD3	-0,179845362	6,701204127	0,643173988	0,882876303
FAM82B	0,43236713	6,05213489	0,643432421	0,882876303
CTBP2	-0,209935986	3,960404969	0,64343425	0,882876303
RRP8	0,300963939	3,580141317	0,643473181	0,882876303
SAP130	0,206402504	6,082660224	0,643523254	0,882876303
NT5C2	0,296952531	7,111671068	0,643888701	0,882876303
NDUFS1	0,227328073	5,842922498	0,643916633	0,882876303
CCDC12	-0,177344069	5,338706188	0,643942385	0,882876303
DNASE1L1	0,277678416	5,029522493	0,643943121	0,882876303
DOLK	0,251788422	4,663052618	0,644038948	0,882876303
KIFAP3	-0,227850915	4,325109247	0,644327835	0,882876303
SLC35A5	-0,196330107	5,256176645	0,644479498	0,882876303
USP37	0,235656069	5,484161745	0,644529076	0,882876303
ADSL	-0,191888434	5,524367079	0,644585054	0,882876303
MTX1	0,300606041	4,459770981	0,644646005	0,882876303
VAMP3	-0,177955243	6,131505694	0,64486434	0,882997978
GALNT10	0,272919828	6,302209113	0,645068182	0,883099765
C7orf31	0,267432049	4,255412312	0,645455994	0,883284107
DDX50	-0,186746609	4,080555333	0,645471625	0,883284107
ZNF445	-0,188193539	4,31132054	0,645591434	0,883284107
ATG7	0,251295103	4,602874073	0,64591783	0,883510094
PHKA2	-0,217366263	4,780418194	0,64601574	0,883510094
ERBB2IP	0,262116233	6,79675532	0,646547626	0,883947461
RAB35	-0,185223537	6,854818061	0,646594799	0,883947461
DDX59	0,210173928	5,299075341	0,646751025	0,883983812
RCAN1	0,265343296	5,021767943	0,647253235	0,884382045
PEPD	-0,193414739	6,495459932	0,647404082	0,884382045
USP34	0,22556422	5,033977216	0,647431467	0,884382045
SIN3B	-0,247899729	5,93710552	0,647754434	0,88454351

GLIS2	-0,207100178	5,644325654	0,647824177	0,88454351
RNFT1	0,320394809	4,518371872	0,648010904	0,88454351
MDFIC	-0,174755225	6,311510352	0,648100126	0,88454351
CCS	0,290363042	5,290576419	0,648397266	0,88454351
MKRN1	0,262106538	6,620518483	0,648642153	0,88454351
MARCKSL1	-0,213968528	5,905475134	0,648724762	0,88454351
FBXO28	0,31550709	5,277510095	0,648886684	0,88454351
ADAM15	0,259998111	6,409006119	0,648894677	0,88454351
DDX49	0,344296354	5,191013714	0,648971767	0,88454351
OTUD5	0,313934772	6,339533617	0,649063554	0,88454351
ASTE1	0,313061541	3,378306682	0,64910628	0,88454351
CREBBP	0,295427347	5,428068884	0,6494603	0,884801682
ARID2	0,248072848	5,133330153	0,649555246	0,884801682
SKIV2L2	0,233993453	5,99864624	0,649745982	0,884884731
HNRNPU	0,229325552	9,279674191	0,649972567	0,885016561
RNF138	0,25356887	5,509592318	0,650117571	0,885037276
FNDC3A	0,279064293	6,620411421	0,651172594	0,88613085
C21orf91	0,246052756	5,515655661	0,651180773	0,88613085
TGFB1	0,313550472	7,824346342	0,651672955	0,88662368
TNIK	-0,209129746	4,905707481	0,651809975	0,886633197
NDUFV3	0,258459188	6,139060693	0,652121789	0,886880431
NHP2L1	-0,186309492	6,828175835	0,652835824	0,887674474
APIP	0,259682076	6,198929015	0,653471862	0,888362167
KLHL23	0,409779069	5,390101863	0,653972024	0,888864906
PCBP4	0,266841225	4,659374295	0,654406856	0,889278667
NOTCH2NL	0,308568417	6,495769553	0,654713791	0,889437294
MRPS27	0,22845024	5,896386507	0,654784458	0,889437294
LPCAT2	-0,199443803	5,105290842	0,655126528	0,889724715
RGPD6	-0,167224887	3,917377521	0,65545666	0,889995812
ARMCX3	0,239812489	5,409321731	0,656411082	0,891114308
NSFL1C	0,270500831	6,283261693	0,656657686	0,891271648
ERI3	-0,173706848	4,498804394	0,657347262	0,891927777
XAB2	-0,162961442	6,069282981	0,657402699	0,891927777
DSTYK	0,273536726	4,001677021	0,65766477	0,8920843
MAPKBP1	-0,22152484	4,376904836	0,657779713	0,8920843
SHMT1	0,279745543	4,679035826	0,657944962	0,89213098
CHORDC1	0,299497299	4,915695144	0,658118773	0,892170382
DOCK5	0,324548885	4,767764256	0,658235693	0,892170382
NRP2	0,351799986	7,312809289	0,658627122	0,892523518
POLR2A	-0,169203483	8,055371366	0,658786181	0,892561687
NDUFAB1	0,233216286	7,690823625	0,659062488	0,892758662
RNF5	0,249574131	6,764401523	0,659345343	0,892850844
AXL	-0,202322268	4,66418994	0,65943996	0,892850844
FAM129A	-0,216345545	6,611358736	0,659635502	0,892850844
TCEAL3	0,236120574	5,493352799	0,659891236	0,892850844
UBE2Q1	0,218723839	6,529429023	0,660092691	0,892850844
C17orf59	0,270139076	4,851982502	0,660118318	0,892850844
RNF130	-0,15847911	7,972814537	0,660227217	0,892850844
GPR108	-0,18006454	5,89049479	0,660389288	0,892850844
OSTF1	-0,195990523	6,196317987	0,660551983	0,892850844

BRD4	0,226712439	8,029298471	0,660669071	0,892850844
SHOC2	0,28710357	6,41148211	0,660808203	0,892850844
FNBP1L	-0,180443844	3,775658239	0,660825081	0,892850844
VASP	0,324766501	4,792477551	0,660892004	0,892850844
LOC100132832	-0,196240177	6,305101071	0,661058064	0,892850844
DYNLL1	0,219052039	8,570175391	0,661357936	0,892850844
EIF3K	0,334267492	8,066098091	0,661655826	0,892850844
ARFIP2	-0,19228425	4,587642189	0,66172007	0,892850844
PCBD1	-0,212612085	6,359330829	0,661738838	0,892850844
TMEM147	0,381292185	6,360998825	0,6619904	0,892850844
RPS19BP1	0,258644968	6,300584652	0,662061213	0,892850844
AGPAT1	-0,192651431	6,993188546	0,662102876	0,892850844
DUSP10	-0,180189538	5,185243421	0,662173042	0,892850844
AIMP1	0,190252318	5,38125269	0,662219282	0,892850844
CSNK2A1	0,210731865	7,277863366	0,662272998	0,892850844
HSPA5	0,229526515	8,989884661	0,662672625	0,893100989
MYO7A	0,3148686	4,182271378	0,662720487	0,893100989
NAE1	0,257174606	4,692294535	0,663021874	0,893192875
CLPP	0,263992841	4,394568276	0,663145523	0,893192875
DAAM1	-0,172646765	6,637155525	0,663181629	0,893192875
IL32	-0,233219625	8,60527887	0,663518921	0,893378597
LMBR1L	0,292720847	5,504252895	0,663581551	0,893378597
ATP1A1	-0,173833217	9,625629666	0,664236813	0,894072555
UTP15	0,27600931	4,571978993	0,664359237	0,894072555
LMTK2	0,203574489	4,994980395	0,66504489	0,894667249
DHX38	-0,186907415	5,469376312	0,665211281	0,894667249
CRIPT	-0,172651044	4,502129652	0,665228126	0,894667249
DDX20	0,247860049	5,240055172	0,665325945	0,894667249
DIP2B	-0,195779089	4,155026955	0,665536328	0,894773702
MEIS2	0,38161647	6,140120067	0,665975634	0,894959229
ZNF768	0,247593912	4,372160895	0,666086072	0,894959229
CDKN2D	-0,185672336	5,350289276	0,666458024	0,894959229
C12orf49	-0,190881435	5,695028987	0,666513526	0,894959229
RBM14	-0,186689093	5,320177095	0,666673959	0,894959229
ANAPC16	-0,185142858	6,56104701	0,666725068	0,894959229
ARFGEF2	0,227439357	5,6782718	0,666785486	0,894959229
VHL	-0,179092211	6,259416183	0,666854049	0,894959229
NTAN1	0,246197906	5,554039111	0,667237191	0,894959229
PIM2	-0,246800494	7,164893602	0,667285287	0,894959229
STK39	0,300295565	5,607351913	0,667313208	0,894959229
BST2	-0,199849689	8,193536342	0,667348183	0,894959229
PARP4	0,233836106	6,763551007	0,667382641	0,894959229
DYNLT1	-0,17639983	6,870057759	0,667511753	0,894959229
PPIG	0,237102114	5,935404909	0,667755677	0,895110273
USE1	-0,17212219	5,978875431	0,668657398	0,896142845
ALG12	-0,205843761	4,838248021	0,668877862	0,896262161
CLDN4	-0,208447089	5,927877372	0,669107977	0,89639436
SLC25A44	-0,184968203	4,508527243	0,669946903	0,897341963
PHF1	0,235308467	6,678749258	0,670720426	0,898159512
SLC39A3	-0,172991788	5,871774171	0,670832936	0,898159512

SUMF2	-0,171614808	6,437440038	0,671065346	0,898159512
C1orf122	0,244521257	5,754225135	0,671084135	0,898159512
ZNF143	-0,19895344	3,920609552	0,67190392	0,899080226
ANXA11	-0,165032891	7,9852251	0,672169656	0,899191476
ZHX1	0,443754189	5,048234941	0,67226807	0,899191476
RFX7	0,233537751	5,721245152	0,672671022	0,899191476
CCDC8	-0,228343334	4,3722995	0,672683775	0,899191476
KDELC1	-0,193626814	3,704303735	0,672939355	0,899191476
RNF14	0,219976398	5,560456323	0,67315173	0,899191476
FBXO42	-0,189911897	5,941210956	0,673187039	0,899191476
NGLY1	-0,176208441	5,35655094	0,673348091	0,899191476
MANSC1	-0,210234846	6,314177271	0,673379777	0,899191476
CYTH4	-0,191976153	4,142300755	0,673567206	0,899191476
ABCA2	0,326737713	5,137134184	0,67367912	0,899191476
DDRGK1	-0,170963572	5,51580312	0,673679361	0,899191476
ANKS1A	-0,170832264	5,419838638	0,673910528	0,899191476
MARCH7	-0,194856279	4,903389309	0,673983925	0,899191476
CHID1	0,201395326	5,619499878	0,674038078	0,899191476
DPM2	-0,16653077	5,762223756	0,67424319	0,899191476
NFX1	0,225646165	4,19798137	0,674322488	0,899191476
WDFY1	-0,150704115	6,017873608	0,674360641	0,899191476
ARFGAP1	0,29716656	5,79597604	0,674696405	0,899463301
PABPC4	-0,148238651	7,543800474	0,67490343	0,899493929
NSMAF	0,263667263	5,315377064	0,674983199	0,899493929
COQ6	0,305777212	3,481005046	0,675146673	0,899535983
RAVER2	0,271720214	4,301666982	0,675634766	0,900010445
BTBD3	0,280044302	5,46558726	0,675887716	0,900171549
NUP43	0,294002319	4,277907936	0,676304982	0,900282524
GTF2A1	0,202187299	6,41583088	0,676320434	0,900282524
XPO7	-0,159364348	5,48485058	0,676367117	0,900282524
PPP1R12A	0,2452976	5,769593332	0,676645395	0,900357089
RRAS2	0,310493236	6,737129152	0,67668721	0,900357089
MANEA	-0,171922	5,242254913	0,677211246	0,900838355
SPAG9	-0,153363388	6,633428189	0,677313132	0,900838355
STX5	-0,13810978	5,522999927	0,677493523	0,900902562
GBA2	0,210157005	5,313372925	0,677779416	0,900902868
PFKL	0,276907367	7,554193381	0,678025858	0,900902868
CYB5R4	0,252616867	3,208876257	0,678161692	0,900902868
GYS1	0,250186619	6,598443313	0,678243916	0,900902868
BIVM	-0,16000366	5,130008165	0,678256403	0,900902868
SLC35C1	-0,169138921	4,389976645	0,678286453	0,900902868
FAM96B	-0,150781995	6,225231623	0,678561153	0,900970655
CSGALNACT2	-0,152292444	5,549730578	0,678601743	0,900970655
CNBP	0,191856587	8,082233399	0,67891149	0,901028419
UQCC	0,242295238	5,17908952	0,678914198	0,901028419
DCP1B	-0,166146553	5,23804682	0,679041655	0,901028419
LYRM4	0,351119729	4,90313253	0,679253957	0,901134773
ERO1LB	-0,166184737	3,542155933	0,67974331	0,901608565
EDEM2	-0,171104476	6,206860073	0,679995167	0,90176722
IER3IP1	-0,164675063	7,503502713	0,680339762	0,901883208

SUMO3	0,183754999	7,051971233	0,680347151	0,901883208
ZNF326	0,253409715	4,617739694	0,680527537	0,901946992
CSTF2T	0,253336101	6,552939706	0,680699917	0,902000143
TRMT112	-0,158079587	7,971835142	0,681073232	0,90231948
SEC62	0,218149443	7,063362453	0,681221282	0,902340311
CUTC	-0,173370798	3,402667959	0,681663916	0,902751262
ZFP36L1	-0,167401779	10,20273118	0,682068634	0,90300619
SMG7	0,230268302	6,906261436	0,682121262	0,90300619
PTMA	-0,147835967	10,63198872	0,682413131	0,903217225
AHCYL1	-0,146477111	7,375945665	0,682862911	0,903263651
PPP1R7	-0,162467807	5,623435086	0,682982113	0,903263651
GALK2	-0,2010085	4,409020093	0,683048067	0,903263651
NDUFB2	0,239168317	6,90594866	0,683057378	0,903263651
CIAPIN1	0,28122676	3,846637135	0,683341951	0,903263651
APAF1	-0,163753585	3,929009772	0,683482806	0,903263651
NF1	-0,148513014	6,341704126	0,683495321	0,903263651
SLC39A1	0,235318851	7,472109671	0,683571067	0,903263651
TCERG1	-0,191253742	5,164176086	0,683640373	0,903263651
STAM	-0,149893544	4,525203923	0,683787062	0,903282444
ACTR1A	-0,14343994	5,516327681	0,684187254	0,90360144
AFG3L2	-0,169064058	4,575457362	0,684293568	0,90360144
ZNF267	0,240215339	5,761407917	0,684962711	0,904309918
C10orf137	-0,186725163	5,390751198	0,685213681	0,904357201
EEF1A1	-0,167658768	12,91030445	0,685263772	0,904357201
LARP1	0,200440203	7,058786877	0,68553928	0,904383153
VCP	0,243632356	8,654303025	0,68558725	0,904383153
SS18	-0,153143436	5,938272336	0,685681317	0,904383153
MED25	-0,157162148	5,034658429	0,686042431	0,904684459
IFI27	0,407548339	9,053446986	0,686384561	0,904960619
RNF123	0,22907515	4,182314224	0,687245435	0,905843907
ATP5J2	0,21954337	8,311453558	0,687648444	0,905843907
C19orf53	0,249685709	6,409434615	0,687758277	0,905843907
CDC25B	-0,179012719	7,330722356	0,687887264	0,905843907
HNRNPH1	0,296876956	7,025754856	0,687996539	0,905843907
UBXN2B	0,289789774	5,448659752	0,688301209	0,905843907
IL8	0,416321617	6,74218035	0,688352018	0,905843907
DPF2	-0,152628085	6,146720665	0,68847486	0,905843907
CLEC2B	0,30068027	6,854223817	0,688822639	0,905843907
ZFPL1	0,226035085	4,957451957	0,688830849	0,905843907
ESRRA	0,25880455	5,621459389	0,689021154	0,905843907
ANKRD36BP1	-0,144582802	7,214338875	0,689096233	0,905843907
YIF1B	0,221641828	6,121744739	0,689172042	0,905843907
CDK2	0,190070626	5,836931967	0,689328336	0,905843907
ZMAT2	-0,163168737	6,498957813	0,689334285	0,905843907
UBE2E2	-0,169788952	6,31904149	0,689528095	0,905843907
CYP1B1	0,400116096	6,686099153	0,689546592	0,905843907
PRR13	0,233001227	8,174849099	0,689635589	0,905843907
REXO1	-0,160774332	5,159462522	0,68971207	0,905843907
SH3BP1	0,248270728	5,180455604	0,689908891	0,905843907
CDCA7L	-0,175783811	4,253760591	0,689970405	0,905843907

TAB2	0,202201772	7,750919492	0,690062562	0,905843907
SKP1	-0,147324238	7,378584276	0,690198758	0,905843907
TARS2	-0,159370718	4,581637952	0,690242695	0,905843907
CSNK1E	-0,151335612	7,563367702	0,690606829	0,906147386
CCDC88B	0,309009723	4,573254321	0,690990289	0,906158308
PPP1R9B	0,266511726	6,586757957	0,691080575	0,906158308
OSBPL9	0,238942371	7,535323174	0,691122542	0,906158308
SMAD5	-0,149052114	5,523969044	0,691146702	0,906158308
DTNBP1	0,20411436	4,836374874	0,691691688	0,906698505
UXT	0,199872815	6,474797516	0,692560387	0,907602726
GPR153	0,297374692	5,079493499	0,692647688	0,907602726
ATMIN	0,187310472	6,553638947	0,692833131	0,90763538
SENP6	-0,164323894	5,944427749	0,692938816	0,90763538
CETN2	-0,146502866	5,616098585	0,693243796	0,907730233
PRDX3	0,217812454	6,797796899	0,693277468	0,907730233
VGLL4	0,264623596	6,971708006	0,693544856	0,907906003
SEC23IP	-0,130176137	7,200231543	0,693847118	0,908127351
TXN2	-0,15873352	6,252761574	0,694188118	0,908399305
POLR2J3	0,325953373	5,576138802	0,694398882	0,908500762
DDX26B	0,314100213	4,451265017	0,694844542	0,908827194
SMC1A	0,234770735	6,246531894	0,694914942	0,908827194
VEZF1	-0,15407394	5,291884946	0,695160933	0,908900484
RPS19	0,229247498	11,00979793	0,69523756	0,908900484
ARMC1	-0,142668089	6,048989929	0,695616425	0,909221469
DUSP23	0,226295256	6,624997461	0,696054858	0,909351204
AKAP12	0,277175478	6,367445994	0,696099694	0,909351204
PITPNM1	-0,153709633	3,82075087	0,696115748	0,909351204
CLIC1	0,241200919	9,247953582	0,696392416	0,90944533
UPF2	0,226275052	4,972344445	0,696454541	0,90944533
SMARCC2	-0,14455916	7,541513595	0,696621903	0,90948971
PRR12	-0,150868029	6,669795938	0,697022818	0,909665471
PQLC1	0,283634387	4,75705791	0,69702333	0,909665471
PDIA5	0,392346088	6,423737932	0,697327829	0,909836043
DIS3	0,210465685	6,479460309	0,697532579	0,909836043
TP53RK	0,225970967	5,073282449	0,697554309	0,909836043
AIF1	-0,180509358	6,95675655	0,697702542	0,909855351
MGAT5	-0,169708098	5,211284336	0,698030365	0,909952396
SIX5	0,295694687	5,372428607	0,698043846	0,909952396
CUL3	0,176469863	7,695323901	0,698404618	0,910088681
MRPS9	0,257095051	5,008580231	0,699149894	0,910088681
ACVR1	-0,146944442	5,782396243	0,699230741	0,910088681
DHX29	0,291104091	6,801442896	0,699325335	0,910088681
DNPEP	-0,152451502	5,187441833	0,699475891	0,910088681
PAPD5	0,285071738	3,623357521	0,699578844	0,910088681
PPM1A	0,201085829	5,862801959	0,69958603	0,910088681
CTBS	0,198549042	5,78573794	0,699601258	0,910088681
HOOK3	0,234165741	6,478629705	0,699608249	0,910088681
RHOF	0,362343452	5,981460852	0,699648581	0,910088681
ELAC2	0,248720151	5,269051173	0,69967025	0,910088681
SQSTM1	0,265558059	10,56035171	0,699749956	0,910088681

USP49	-0,181837422	4,067001697	0,700114076	0,910388613
HDAC7	-0,163945025	7,518183991	0,700626113	0,910880737
TNIP1	0,204911594	7,553422723	0,701320132	0,911240733
PILRB	-0,178634187	7,310940919	0,70133276	0,911240733
ZNF662	0,464749705	5,428766811	0,701579276	0,911240733
DGKA	0,25413816	5,570668078	0,701629159	0,911240733
PLBD2	0,193793151	5,722784397	0,701802769	0,911240733
RPS26	0,250909017	9,83982878	0,701829187	0,911240733
INF2	-0,142768009	6,322513459	0,701838441	0,911240733
THBD	-0,19188057	7,436310452	0,702028861	0,91131445
LPP	-0,159344065	6,497184532	0,702400394	0,911623199
UBE2B	-0,159756642	6,62849726	0,702597404	0,911705366
REST	0,23259993	6,348595855	0,702751993	0,911732466
FUT4	-0,18427835	5,49093674	0,703117339	0,912032934
ZNF532	0,207566257	7,796124968	0,70341511	0,912245652
POLR3GL	0,171666104	6,184074674	0,703585371	0,912292954
RNF214	0,271435076	4,642815061	0,704045055	0,912435787
RAB13	-0,128522917	7,665110912	0,704188027	0,912435787
FTO	-0,140196022	6,234962026	0,704549557	0,912435787
PPCDC	0,233702629	3,874317665	0,704614414	0,912435787
SYAP1	0,197929968	5,803897044	0,70483949	0,912435787
CDR2	0,204793104	5,121720228	0,704925563	0,912435787
UQCR11	-0,136630036	8,825221711	0,704925845	0,912435787
HIVEP1	0,25417975	4,56495563	0,70494143	0,912435787
CHMP4B	0,208827831	7,149119409	0,704941476	0,912435787
FRMD4A	0,287731198	5,855201044	0,705033606	0,912435787
ZEB1	-0,152794731	6,078121753	0,705600839	0,912824566
MED24	-0,172586023	5,717853093	0,705790696	0,912824566
RAB31	0,225943793	7,684931578	0,70599079	0,912824566
ANKRD10	0,286720798	6,754535831	0,706010284	0,912824566
ZNF227	0,221800793	4,179700289	0,706016485	0,912824566
BCAS3	-0,168778952	6,256346257	0,706162402	0,912824566
DCAF4	0,242068376	5,023455734	0,706357177	0,912824566
HIAT1	0,187334577	6,297080244	0,706404933	0,912824566
IARS2	0,229179972	6,298192038	0,706607471	0,91291329
ZNF330	-0,120595003	5,602301108	0,707455266	0,913683465
UBE4B	-0,156478723	5,43012713	0,707625539	0,913683465
NPAT	-0,159842952	4,726893126	0,707685662	0,913683465
CALM1	-0,157776437	9,305070061	0,707739561	0,913683465
TLE3	-0,132254673	5,510894742	0,707917842	0,913740632
NBAS	-0,145472787	4,956187718	0,708059213	0,913750147
ZNF335	0,306915022	4,995363018	0,708318614	0,913911945
MANBAL	-0,127764068	5,614790672	0,70900687	0,914444157
RECQL5	0,242348561	5,257302393	0,709199834	0,914444157
NUDC	0,173552489	5,270535339	0,709215671	0,914444157
KLHDC10	0,241674491	5,377959739	0,70926751	0,914444157
CBLB	-0,174161783	5,420465503	0,709628096	0,914736104
NUMBL	0,284984084	5,580994348	0,709806578	0,914793244
GPBP1	0,227152935	7,102310404	0,710046025	0,914924866
EAPP	-0,146517202	5,606538537	0,710342273	0,914924866

GTPBP4	-0,155327193	4,78821596	0,710513334	0,914924866
MED4	0,193498067	5,86551922	0,710576289	0,914924866
LIMS1	-0,156923196	6,553147116	0,710579571	0,914924866
S100A4	-0,194341453	8,934120545	0,71105811	0,91502272
PDIA4	0,298178197	5,254891151	0,711284383	0,91502272
GPAA1	0,250659254	7,145290165	0,711359022	0,91502272
ACSL3	0,216401129	6,73558404	0,711625678	0,91502272
AMBRA1	0,301634372	4,457959097	0,711836014	0,91502272
ATP2A2	-0,142885773	6,932964537	0,712312396	0,91502272
RASSF8	-0,142585822	5,505030641	0,712344841	0,91502272
ARFGAP2	0,24756859	5,429248118	0,712444115	0,91502272
SFT2D2	0,169862675	6,074308919	0,712486861	0,91502272
ASAP1	0,238271427	6,668638179	0,712670234	0,91502272
GTPBP1	-0,150394886	6,143216768	0,71282529	0,91502272
AGTPBP1	-0,1642067	4,620995131	0,713124878	0,91502272
PIM1	-0,153024558	7,209640876	0,713172064	0,91502272
HNRPDL	0,184031913	8,46197968	0,713203927	0,91502272
LRP5	0,378097101	6,234956425	0,713379376	0,91502272
CDK14	0,319130615	5,763630308	0,713583729	0,91502272
RFXANK	-0,152705605	6,334417786	0,71379349	0,91502272
BRI3	-0,165233142	8,740252923	0,713838677	0,91502272
FGL2	-0,169054445	5,918314352	0,713967394	0,91502272
SFI1	-0,183942333	5,663458884	0,713997814	0,91502272
GGNBP2	0,213063704	6,097499968	0,714146208	0,91502272
CDKAL1	-0,138067409	5,702110169	0,714174325	0,91502272
FAM18B2	0,282495767	4,493547306	0,714177314	0,91502272
APPL1	-0,146992567	5,514126382	0,714259333	0,91502272
EPS15L1	-0,144264741	6,874004495	0,714292865	0,91502272
UMPS	-0,126987624	5,502459312	0,714390954	0,91502272
RPAIN	0,202484085	4,452077697	0,714454383	0,91502272
ZMYND11	0,18363792	5,89802913	0,71447677	0,91502272
QRSL1	-0,145971452	3,566278181	0,714669594	0,91502272
EXOC8	-0,150031269	5,693575046	0,714790286	0,91502272
AKAP9	-0,159578955	5,782200453	0,714815373	0,91502272
UBIAD1	-0,151420103	5,149193808	0,715070223	0,915177149
PNPLA4	0,293840142	4,549493584	0,715233209	0,915213971
PTPN12	0,230222731	6,938128174	0,715374199	0,915222639
EIF4G3	-0,137452143	6,95707816	0,7155708	0,915283189
GOPC	0,19416859	5,238261954	0,715689979	0,915283189
DEAF1	0,197166167	4,951537824	0,716074774	0,915603578
USP36	0,213986514	7,364281241	0,71673325	0,916173381
MMS19	0,206664228	6,539965441	0,716789117	0,916173381
ZBTB6	0,206211091	4,336286167	0,717071063	0,916221457
HSPBP1	-0,119094828	6,503534527	0,717095456	0,916221457
OPA1	-0,158214552	4,54128832	0,71755906	0,916403192
INTS12	0,190730944	4,610553405	0,717562531	0,916403192
DHX8	-0,148522084	4,425242935	0,717760013	0,916403192
LRCH3	0,213739128	5,771951585	0,717775253	0,916403192
HOMER1	0,219468627	2,984596739	0,718041912	0,916572032
ZNF808	0,273945475	4,69557382	0,718422359	0,916783338

FAM20A	-0,150724794	5,634201876	0,718476339	0,916783338
ASXL2	-0,112928941	6,39223524	0,719048809	0,917244781
ZNF646	-0,156541562	4,779859374	0,719106995	0,917244781
LCP2	0,296451523	7,22202965	0,719418556	0,91747057
HNRNPL	-0,121513263	8,036534894	0,719674048	0,91761496
ZXDC	-0,148559907	5,900181035	0,719907362	0,91761496
C9orf64	0,191963587	5,20211897	0,720059698	0,91761496
ZBED4	-0,145245851	3,897280885	0,720070047	0,91761496
SLC25A36	-0,134857176	6,243478287	0,720232029	0,917649888
HELB	0,239388542	5,005948261	0,720399664	0,917692006
PHF16	0,220896266	4,926250431	0,720726183	0,917936466
NUDT3	-0,115697061	6,47469801	0,721066884	0,918172954
AMN1	-0,154797252	3,291557398	0,721404002	0,918172954
MAP4K3	0,221417058	6,502029093	0,721440633	0,918172954
UBE2O	-0,149122151	6,148366769	0,72145046	0,918172954
SLC25A17	-0,159928231	3,049456692	0,721587671	0,918176214
DDX28	0,211483065	5,147263587	0,72278595	0,919529364
RSBN1L	0,177270391	5,597129256	0,723636929	0,920440258
GLB1	-0,128678523	6,170254885	0,723896527	0,920574563
CC2D1A	0,255138259	5,852474295	0,724012521	0,920574563
USO1	0,203885194	5,636177689	0,724952051	0,921457408
TMX2	-0,107227393	7,302568694	0,724977122	0,921457408
MIER2	0,244856247	5,309850285	0,725347104	0,921522966
PCDHB9	-0,15461987	6,140931871	0,725545326	0,921522966
LOC100170939	-0,153166714	4,997521378	0,725674693	0,921522966
PEX13	0,19149432	6,432796813	0,725678293	0,921522966
HAUS1	0,198229842	4,707501435	0,725704403	0,921522966
MUM1	0,187966073	5,657606897	0,725912005	0,921614963
BTBD2	-0,142206074	6,251948316	0,726090161	0,921669547
FRS2	-0,13525078	5,517099329	0,726466331	0,921829197
SREBF2	-0,145435813	6,768894688	0,726773502	0,921829197
SAV1	0,167160161	5,823692137	0,72692631	0,921829197
LBR	0,217334149	6,536953585	0,726940266	0,921829197
ACADM	-0,153895642	4,850314228	0,727090168	0,921829197
ZW10	0,220916556	3,57534587	0,727143707	0,921829197
ZBTB38	-0,137172274	7,31879079	0,72716223	0,921829197
TPST2	-0,153616482	6,294673476	0,727581278	0,921907962
PPIL4	0,183488793	5,864759906	0,727645518	0,921907962
IFFO2	0,219957024	5,012010405	0,727746329	0,921907962
BTN3A1	-0,144081803	4,839512873	0,727822521	0,921907962
PVR	0,233217084	6,655964682	0,727960807	0,921907962
CETN3	0,231740601	3,991985524	0,72804853	0,921907962
BCL7C	0,17933462	5,087154425	0,728222071	0,921907962
STAT1	0,27875628	7,429208665	0,728305938	0,921907962
ATF7IP	0,198773674	7,294566826	0,728868123	0,921923178
MTIF3	0,208442474	5,895152428	0,728914041	0,921923178
KIAA0754	0,248626351	6,732406512	0,729029136	0,921923178
ZNF629	-0,145983746	3,551157411	0,729206277	0,921923178
SUSD1	0,219295383	5,58548514	0,729641958	0,921923178
MEX3C	0,185921136	5,799790084	0,729763384	0,921923178

FIZ1	-0,153859972	4,318149355	0,729790729	0,921923178
RPLP2	-0,12366933	11,32117873	0,729825213	0,921923178
ARID1A	-0,125937013	7,173143657	0,72983716	0,921923178
ZMIZ1	0,333758236	6,271171016	0,729845907	0,921923178
SLFN5	-0,139056306	6,969960262	0,729874581	0,921923178
POLR3H	-0,125973786	5,390443413	0,729940349	0,921923178
PIAS3	0,213265148	6,437558869	0,730384403	0,922226607
MKNK1	0,273503201	3,997789033	0,730484106	0,922226607
LSMD1	0,398376392	7,106920388	0,730586322	0,922226607
C1orf52	0,205007421	4,684061773	0,73098634	0,922397042
DYRK1B	-0,167180137	4,27800282	0,730991877	0,922397042
SUN1	0,166603176	7,102297169	0,73130167	0,922576915
BMI1	-0,142890009	7,283669807	0,731405016	0,922576915
KIAA0913	0,2554052	6,972886746	0,731637154	0,922699048
ATP6V1C1	0,181205338	6,413533221	0,732149106	0,922934095
ENDOG	0,273002793	3,986704587	0,732202034	0,922934095
MBTPS1	0,242951924	7,450481851	0,732237862	0,922934095
TSSC1	0,216632726	4,687828262	0,732423519	0,922934095
MARCH6	0,159515507	7,913328038	0,732500267	0,922934095
PLSCR3	-0,149267047	6,249507111	0,73323551	0,923532828
TP53I3	0,254735123	4,273911257	0,733246331	0,923532828
HECTD1	-0,111939991	6,454190175	0,733493977	0,923604388
BTBD1	0,187196108	7,389371617	0,733574038	0,923604388
NR2C2	-0,130637338	5,649246586	0,733792641	0,923709068
SUDS3	-0,122509065	5,070047517	0,734394504	0,924296072
LSM6	0,223914157	5,564708769	0,734567861	0,92434365
SLC17A5	-0,174296573	6,302532321	0,735072525	0,924672411
KRTCAP2	-0,157989701	7,882348751	0,735100328	0,924672411
PCDHB19P	0,338754511	4,388619801	0,735338902	0,924801913
UPK3BL	-0,144469285	6,103640169	0,735807411	0,925144279
ATP1B3	-0,141850522	7,356819232	0,736090212	0,925144279
UBE2D2	0,194959383	7,987329476	0,736235846	0,925144279
ZBTB10	-0,149718172	4,39902496	0,736243472	0,925144279
LUC7L3	0,203854188	6,871864155	0,736365192	0,925144279
UBE2H	0,202136694	7,222643873	0,736481255	0,925144279
SPG21	-0,104087421	6,27312727	0,736560828	0,925144279
ROBO1	0,204184756	5,891788468	0,736972313	0,925474647
WDR33	0,158838622	7,085411517	0,737095294	0,925474647
MED21	-0,114321417	5,914573704	0,737316847	0,925582396
UBQLN2	-0,11478098	7,140534316	0,737582179	0,92571366
ZFAND2B	-0,140979396	5,251746079	0,737692921	0,92571366
VPS4A	0,170933039	6,521994527	0,737850565	0,925741123
PIGN	-0,132293556	5,378436438	0,738038067	0,92580603
MTSS1L	0,318516651	6,650165279	0,73832931	0,925977176
C1orf56	0,278879812	4,043522694	0,73850553	0,925977176
C19orf24	-0,127720483	4,99054468	0,738693778	0,925977176
EIF4E	0,214658258	6,986209034	0,738717677	0,925977176
SLC30A5	0,203303453	4,577551969	0,738881616	0,92601245
FRMD8	-0,128822952	5,771557128	0,739294257	0,92625553
SNX27	0,206005868	5,735525741	0,739347243	0,92625553

ARPP19	-0,1138668	6,52070982	0,739856181	0,926560931
RDH11	0,19789243	5,656869984	0,739862776	0,926560931
IAH1	0,18871771	6,016054859	0,740377631	0,926633319
EHMT2	0,22122589	5,808601099	0,740459008	0,926633319
DDR1	-0,142882916	6,123065941	0,740671495	0,926633319
LOC100190986	0,25619769	9,860753485	0,740809446	0,926633319
STAM2	0,16951382	6,647699963	0,740914837	0,926633319
VRK2	0,266982337	6,212621961	0,740970543	0,926633319
SETDB1	0,210513374	4,762659068	0,741219154	0,926633319
APOBEC3G	0,252400942	5,004448335	0,741222579	0,926633319
CBWD1	0,2377774	6,029207197	0,74136651	0,926633319
CRTAP	-0,121784717	8,185409033	0,741401738	0,926633319
SH3RF1	-0,137955274	4,506768102	0,741455827	0,926633319
TUFM	0,157501149	6,53337858	0,741551257	0,926633319
NDUFA9	0,190736139	5,90815884	0,741806086	0,926747459
DBR1	-0,130017117	4,360069439	0,741914412	0,926747459
MON2	-0,131004724	7,190750539	0,742093769	0,926771667
NDUFB4	-0,138990407	7,581072967	0,742334615	0,926771667
ARAF	-0,126102304	6,114812964	0,742435117	0,926771667
LUZP1	-0,121643215	4,774428469	0,742477433	0,926771667
TMEM97	0,226279871	5,218953599	0,742870212	0,927092235
SLC27A1	-0,137915867	4,087224017	0,743318244	0,927358499
DNMT3A	0,243354268	3,749647164	0,743425247	0,927358499
AGAP3	0,278637417	6,41455362	0,743491555	0,927358499
POLR3A	0,199704635	4,253543825	0,744037081	0,927869212
TIAM1	-0,144646155	4,3297564	0,744308898	0,927990395
ATG16L1	-0,143618185	4,438046604	0,744441586	0,927990395
FAM100B	-0,115311327	8,481026977	0,744695675	0,927990395
USP24	0,186620906	7,274817252	0,74483421	0,927990395
AKT3	-0,144189218	7,501174811	0,74493045	0,927990395
LZTR1	-0,146080926	6,032233137	0,745229193	0,927990395
DCTN6	-0,120553125	6,884823837	0,7452317	0,927990395
CASP4	0,23794528	6,99048114	0,74525394	0,927990395
TBRG1	-0,129394344	7,326252462	0,745602651	0,927990395
IFIH1	0,319317807	4,741616623	0,745629376	0,927990395
ALDH7A1	-0,136092071	5,398446366	0,745697909	0,927990395
FAM13B	-0,13834834	5,484232826	0,745769429	0,927990395
EIF2A	0,207633988	7,056382695	0,74590341	0,927990395
LOC100271836	-0,124983587	9,421203184	0,746101741	0,928067817
NUP133	0,192003272	4,998288022	0,746441388	0,928273328
TRIM5	-0,146529394	4,936581868	0,746539218	0,928273328
C12orf44	0,183539802	5,223309931	0,746760699	0,928379436
UBE2F	0,18109884	5,097006195	0,747037084	0,92855375
MYC	0,281849503	7,027207016	0,747224746	0,928617741
NFKBID	-0,124079414	4,409210335	0,747476835	0,92876176
SNX3	-0,138462258	7,301202962	0,747841714	0,928819562
HSF2	0,243577874	4,339340668	0,747907545	0,928819562
ATP11C	0,198595357	5,433690761	0,747953707	0,928819562
COMT	-0,121937814	7,194443297	0,748086783	0,928819562
LY6E	-0,127786109	7,086161579	0,748334809	0,928819562

EEA1	0,27186692	4,424684324	0,748340618	0,928819562
EIF4B	-0,155056884	6,045604763	0,748616687	0,928993119
CTSL1	-0,140160905	8,29525673	0,74888583	0,929072604
SRD5A3	0,280452348	4,171777332	0,748953234	0,929072604
SNHG11	0,193865679	4,05204606	0,749685532	0,929485125
TMED2	0,16125657	8,405419467	0,749704899	0,929485125
THRAP3	0,184139397	6,819581867	0,749744308	0,929485125
INO80B	0,214269315	5,939718814	0,750127554	0,929485125
C2orf43	-0,110155537	5,197203029	0,750131084	0,929485125
BTN2A1	-0,120949596	6,128903732	0,750143985	0,929485125
TRAM2	-0,113131517	6,648706352	0,750430698	0,929485125
STAU2	0,210523518	5,10497631	0,750483606	0,929485125
GTF3C2	0,16463845	6,526836286	0,750512553	0,929485125
KIAA1009	-0,117383835	4,548092666	0,750979364	0,929894368
ABT1	0,179572211	4,969939945	0,751501334	0,930163674
NFAT5	-0,114855002	6,695584481	0,751587718	0,930163674
CPSF6	0,159666495	7,635894051	0,75169728	0,930163674
DUSP8	0,295095146	4,755623868	0,751838105	0,930163674
ARL8B	0,16824931	8,384791333	0,752002754	0,930163674
TTC19	-0,128346934	4,476621596	0,7520153	0,930163674
BCS1L	0,230826101	5,000753576	0,75222824	0,930218782
VCAM1	0,331335202	5,568489021	0,752383191	0,930218782
KIAA0355	0,270342344	4,377526334	0,752633777	0,930218782
CTDSP2	0,244584763	6,668636021	0,752668156	0,930218782
GAS6	-0,160302243	7,497672597	0,752741933	0,930218782
UFC1	-0,098940494	6,611410159	0,753118674	0,930515716
NUPR1	0,348995869	10,36686288	0,753316483	0,930591503
SUV420H1	0,201508595	6,332625109	0,753576161	0,930743677
METTL10	0,188021467	4,689067707	0,754071034	0,931186233
HNRNPUL1	0,154377487	7,628076814	0,754301983	0,931194268
PDPR	0,193241053	5,036865821	0,75445754	0,931194268
SBDSP1	0,198318777	5,768480192	0,754551026	0,931194268
COPS8	0,175765658	7,090347102	0,754681276	0,931194268
CAPRIN1	0,199119041	8,71156649	0,754760335	0,931194268
HBS1L	0,186006094	5,025702343	0,755661347	0,93178553
SATB1	-0,157794607	5,004017262	0,755673093	0,93178553
SLC1A5	-0,136545966	6,750967274	0,755777511	0,93178553
ZNF586	0,228808031	6,792280804	0,755873381	0,93178553
FKBP2	-0,112735369	5,999500555	0,755922797	0,93178553
TUBB	0,205038055	8,702924687	0,756083615	0,93181532
ULK1	-0,120568111	6,37745548	0,756439801	0,931890003
ANXA5	-0,124435809	7,668186181	0,756534433	0,931890003
RPL15	-0,117203672	10,79032033	0,756554195	0,931890003
YME1L1	0,162825475	6,8556331	0,757078299	0,932367152
LTBR	0,190293535	8,355318969	0,757370948	0,932559135
MAST2	0,210754547	5,761134762	0,757661837	0,932748884
ZNF124	0,263637714	5,895368211	0,757934701	0,932916376
TBCD	-0,115376994	7,310581103	0,758189465	0,932946081
SNAPC3	0,186629191	5,810450502	0,758232465	0,932946081
MRFAP1	-0,101458775	8,643924417	0,758644656	0,933284848

ALMS1	0,186840072	3,777773437	0,758969268	0,933515772
PMPCB	0,166304437	6,504721683	0,759387613	0,933847877
PDXK	-0,110414836	6,918114579	0,759602663	0,933847877
PCDHB14	0,211365049	3,832278189	0,759840118	0,933847877
TPM4	-0,122894842	9,751936297	0,759912232	0,933847877
SOCS4	-0,113393141	4,4760551	0,759954929	0,933847877
FAM174A	-0,104811936	5,186478199	0,760170852	0,933847877
ABI1	0,190731356	7,616752778	0,760355762	0,933847877
ETV5	-0,112442498	7,22004042	0,760430568	0,933847877
RRM1	0,244387063	4,385030767	0,760592961	0,933847877
TM9SF4	0,166170543	6,260398638	0,760608756	0,933847877
CDC42BPB	-0,113251285	6,171590639	0,761462473	0,934525372
MAP2K7	-0,123368621	5,330134397	0,761508829	0,934525372
LASP1	0,188327925	8,34787745	0,76157171	0,934525372
EXOSC5	0,191265896	5,028201475	0,761863078	0,934569993
MLL2	0,262119034	7,433526829	0,761936896	0,934569993
CHD2	-0,105393019	6,909006152	0,762102558	0,934569993
ELL	0,272964105	4,908775429	0,762277044	0,934569993
APEH	0,176774796	5,537767347	0,762309593	0,934569993
CRAMP1L	0,19109554	4,957114682	0,762592377	0,934569993
LAT	-0,110830035	4,974865656	0,762768853	0,934569993
PES1	0,239193499	5,854532551	0,7629618	0,934569993
PBX3	-0,123631741	4,597298205	0,763037076	0,934569993
ATF4	0,180072354	8,464106989	0,763115798	0,934569993
TMED9	0,157502954	7,885024188	0,76322307	0,934569993
CCNJ	0,174146708	4,706385338	0,76334173	0,934569993
TMED3	0,214248695	6,25228085	0,763389773	0,934569993
DOLPP1	-0,114789949	4,080951131	0,763810016	0,934916621
MRE11A	0,176056703	6,130184333	0,764206081	0,935098854
NDUFA4	0,169573791	8,899445309	0,764465321	0,935098854
KIN	0,280529142	4,645016889	0,764476394	0,935098854
ERP44	-0,11815408	6,486441576	0,764752835	0,935098854
RCHY1	0,16218183	5,175126687	0,764944582	0,935098854
IRF9	-0,097597443	7,146826767	0,764968414	0,935098854
TAF3	-0,110498167	4,551942565	0,765147584	0,935098854
FUS	-0,128592665	7,148738377	0,765164549	0,935098854
RLTPR	0,329043031	3,78882888	0,765193079	0,935098854
BTF3	-0,120232713	6,714530199	0,765472668	0,935272912
FAM117B	0,251281398	4,086217592	0,76610599	0,93561301
BLNK	-0,127403459	4,623596374	0,766148406	0,93561301
RYBP	0,156825366	5,09603524	0,766162641	0,93561301
ZNF564	-0,118080287	5,253717054	0,766324505	0,935643116
MGST1	0,372769922	8,582538769	0,766687786	0,935919085
RNF217	0,231635084	5,556459195	0,766902874	0,936014086
MED29	0,253869838	6,99715163	0,767468228	0,936536479
PSMD1	0,197033277	6,203332411	0,767658516	0,936601077
TEAD1	0,259460902	5,377423534	0,768117911	0,936645772
CS	-0,108613169	5,606144259	0,768125927	0,936645772
CBL	0,178165948	5,252485183	0,768159971	0,936645772
NOSIP	-0,096806297	6,260537869	0,768244581	0,936645772

CHD3	0,24178746	7,985530646	0,768850583	0,937217041
G3BP1	0,133422746	8,42996415	0,76923829	0,937470225
PPP2R3C	0,171832461	4,338916517	0,769385091	0,937470225
EXOC3	-0,109621642	5,705225326	0,769786871	0,937470225
C11orf31	0,171072979	7,697165848	0,770175273	0,937470225
MMAA	-0,114386285	4,039430197	0,770244078	0,937470225
NNT	0,169002095	6,89526949	0,770317158	0,937470225
FAM193A	0,20897602	4,124557757	0,770493741	0,937470225
SLC39A4	0,178715385	6,660384511	0,77053496	0,937470225
VKORC1L1	0,160761644	6,495879342	0,770565476	0,937470225
RSC1A1	0,191411283	6,351290787	0,770623951	0,937470225
NUDT16	0,206067418	4,925321561	0,770663587	0,937470225
PTPRJ	-0,109484255	5,18799469	0,770797814	0,937470225
RAPH1	-0,113201673	5,930255631	0,770845513	0,937470225
MEPCE	-0,117137089	6,130503336	0,771106672	0,937620612
ZNF77	-0,12088615	4,423852238	0,771955688	0,938485618
TOR3A	0,150628715	6,853063886	0,772579194	0,938728799
RPS20	0,168198996	10,80192033	0,772587473	0,938728799
RIPK1	-0,101164338	4,887493672	0,772623163	0,938728799
ZCCHC14	0,168626166	6,952341298	0,772736415	0,938728799
MPDU1	-0,101355009	5,194675271	0,772900116	0,938728799
DHX40	0,154967624	4,168490904	0,77299163	0,938728799
SLC4A2	0,25022369	5,925651761	0,773119363	0,938728799
CBX5	0,189938457	6,57775694	0,773487588	0,938978279
BFAR	0,151305644	5,785463085	0,773601469	0,938978279
PPIC	-0,114432633	6,698800551	0,77394038	0,938978279
GOSR1	0,193018361	6,172913241	0,774027615	0,938978279
TBC1D1	0,206405775	5,881267447	0,774075003	0,938978279
ZNF678	0,227890061	5,151046476	0,774200491	0,938978279
TRAF3IP2	-0,106180444	5,438714786	0,774292132	0,938978279
STT3B	0,154671824	6,591550532	0,774461108	0,938978279
CLK3	0,262262583	6,05713112	0,774690028	0,938978279
ACOT9	-0,101251427	5,697392436	0,774701833	0,938978279
FGFR1OP	0,216745044	4,793718083	0,774980788	0,939149457
SLC43A3	-0,118230151	7,711529786	0,775299548	0,939368802
SEC13	-0,10805377	6,554923609	0,775814919	0,939513022
NBR1	0,140902198	7,977974695	0,77588241	0,939513022
TMEM189	0,271265325	7,539813507	0,776025917	0,939513022
PLEKHA5	0,184779325	3,263501521	0,776032067	0,939513022
COMMD4	-0,103212783	5,731453321	0,776204157	0,939513022
TNRC6C	0,287381546	5,041631049	0,776245251	0,939513022
BAG3	0,172921476	7,997547555	0,776821425	0,939755894
RNF139	-0,095297482	6,258330244	0,777156247	0,939755894
HERC5	-0,112057156	3,943960178	0,777431423	0,939755894
JKAMP	0,148534624	6,019869177	0,777465958	0,939755894
MRPS11	-0,09766304	5,350207864	0,777490147	0,939755894
ZNF688	0,187295666	4,193327615	0,777568544	0,939755894
MRPS14	0,172886738	4,656583454	0,777596821	0,939755894
FEM1B	0,189137923	7,600428738	0,777637874	0,939755894
SNHG8	0,161729101	6,453059047	0,777815799	0,939755894

PAPSS1	-0,105094719	5,603840404	0,778030848	0,939755894
TMEM57	-0,103734486	5,127093416	0,778245117	0,939755894
C11orf49	-0,100566037	5,315473214	0,778333514	0,939755894
EBPL	-0,114701757	6,109162625	0,778367616	0,939755894
TPD52L2	-0,094931693	6,146827605	0,778375317	0,939755894
RBM6	-0,120609609	6,660085887	0,778713765	0,939842431
SEPT7	0,157650963	9,175918709	0,778722648	0,939842431
TULP3	0,20350894	5,693579073	0,778864881	0,939847749
ORAI2	0,194875026	5,678403721	0,779138031	0,940011011
EIF3G	-0,094345225	7,95369441	0,779871856	0,940353136
TMUB2	0,161413041	5,700472944	0,779893668	0,940353136
TUBG2	0,217520323	5,700895928	0,779900969	0,940353136
G3BP2	-0,089595757	7,300102523	0,780092657	0,940353136
ZNF713	0,276056784	4,129637102	0,780294824	0,940353136
SLC35C2	-0,106920908	6,01496345	0,780318553	0,940353136
COPS4	-0,100437387	4,889122817	0,780386919	0,940353136
WBSCR22	0,19925416	5,987023411	0,780601173	0,940398767
HBP1	-0,100079452	6,194605059	0,780700604	0,940398767
PAAF1	0,193182509	4,621361571	0,781129036	0,94047916
HYOU1	0,193938318	6,588756482	0,781212726	0,94047916
RPS12	0,156244506	11,06051481	0,781472844	0,94047916
GJA1	-0,124106728	8,247047573	0,781583988	0,94047916
CLSTN3	0,176547474	6,049911057	0,781792934	0,94047916
PPRC1	-0,097590916	5,072370274	0,78192199	0,94047916
RBBP9	0,188088383	4,324988526	0,78216852	0,94047916
BAHD1	0,201962527	6,558908143	0,782255351	0,94047916
BHLHE41	-0,116303313	5,846246682	0,782289327	0,94047916
ZNRD1	0,152537935	4,700145123	0,782298693	0,94047916
TUBGCP4	-0,101706936	4,815408184	0,782419176	0,94047916
CTNNBL1	0,161062356	6,230714518	0,78242239	0,94047916
PQBP1	0,190289507	5,175024094	0,782902036	0,940803727
IK	-0,092945096	6,82239911	0,783220296	0,940803727
ISCA2	0,181895915	6,077789609	0,783265621	0,940803727
LYSMD3	-0,09432838	4,603511882	0,783650677	0,940803727
GPR137B	-0,102744122	6,104660016	0,783695611	0,940803727
DERL1	0,150913795	6,251434113	0,783841604	0,940803727
FOXK2	0,191378175	3,532803903	0,783860335	0,940803727
DBNDD2	0,172055534	7,175535172	0,78386998	0,940803727
DNM2	-0,098991299	4,699790715	0,784059942	0,940803727
CYTH2	-0,094845068	6,676552461	0,78407209	0,940803727
FAM133B	0,147956162	5,385178906	0,784371808	0,940967161
DNAJB14	-0,094383589	6,101009145	0,784484281	0,940967161
TTLL5	0,18998764	6,134873256	0,784641737	0,940990504
MALAT1	-0,110105441	12,49002656	0,785547836	0,941911498
PJA2	-0,090231435	7,581578389	0,785967976	0,942001771
AMOTL2	-0,120126453	5,47504521	0,785970691	0,942001771
PIP4K2B	0,219281972	6,832991586	0,786037553	0,942001771
GNG12	0,214638847	7,257227301	0,786490136	0,942120435
PPP1R2	-0,099335722	6,248869236	0,78661451	0,942120435
PICK1	-0,107278178	5,469867614	0,786711772	0,942120435

SLC6A6	-0,110393608	6,454601887	0,786796695	0,942120435
CYBA	0,211701009	7,409679452	0,786827376	0,942120435
ZNF664	0,153948721	6,895123175	0,787096556	0,942250671
FCHO2	-0,095897576	6,332787138	0,787212505	0,942250671
SDF2	0,176460306	5,610755128	0,787671002	0,942497071
CDK5RAP3	-0,109432387	6,784995495	0,787774257	0,942497071
TCEAL4	-0,095823086	6,490198204	0,787833011	0,942497071
TIMM44	-0,099369143	5,890803604	0,788222329	0,942797415
RAC2	0,273195802	6,681314456	0,788581103	0,943061127
FAM111A	-0,098978927	6,21774478	0,788735424	0,943080283
WDR36	0,193811666	4,188218472	0,788927734	0,943144849
WTAP	-0,106855986	4,475957055	0,789091627	0,943175426
RPS6	0,170717052	10,6811058	0,789872583	0,94394196
PGRMC1	0,156990636	8,000108003	0,790009792	0,94394196
ABL2	0,221276118	5,48533168	0,790404279	0,944161542
TBPL1	-0,094740098	5,03067704	0,790514045	0,944161542
FASTKD2	-0,090915575	4,932343308	0,790608947	0,944161542
C6orf106	0,126219103	7,086010279	0,790979568	0,944322681
AIP	0,214946077	5,159816392	0,79110403	0,944322681
PVRIG	0,197098265	5,840852048	0,791159331	0,944322681
WDR6	0,158491231	6,676661751	0,791423198	0,944472312
CSTF3	0,166048157	4,534280987	0,79170293	0,944571727
TP53BP2	0,173370175	6,271822564	0,79179703	0,944571727
GCC1	0,20167455	5,716369254	0,792305662	0,944571727
RARRES2	-0,11157923	8,981308503	0,792378664	0,944571727
POGK	-0,092652367	4,994271179	0,792407136	0,944571727
SMCHD1	-0,089853654	6,455547533	0,79248443	0,944571727
ANKRD12	-0,095459064	7,668507138	0,792581314	0,944571727
GSK3B	0,184026586	7,159876185	0,792614668	0,944571727
SERPINB1	0,291364035	6,825885218	0,793206357	0,945111681
RPA1	-0,088637814	5,142167346	0,793378247	0,945151339
PRCP	-0,11068609	5,995040526	0,793914517	0,945624994
SERPINE1	-0,113325152	8,689877057	0,794291151	0,945654
RPL27	-0,090774854	10,30934526	0,794406617	0,945654
RP2	0,134466728	5,500252325	0,794709432	0,945654
COL4A1	-0,111536252	7,78870619	0,794742003	0,945654
SCRN3	0,159684921	4,558525916	0,794797664	0,945654
SCPEP1	-0,094397768	7,000111256	0,794826207	0,945654
C3orf18	0,198198099	3,853090522	0,794959891	0,945654
PXMP2	0,183290856	4,691191162	0,795048303	0,945654
TGS1	0,184241711	3,892398242	0,795271061	0,945753987
LILRB2	-0,095449464	4,489237981	0,795543479	0,945912988
LOC283922	-0,091631313	7,492382883	0,795781211	0,946030697
WDR13	0,150679647	7,106771051	0,796044329	0,94609342
FAM89B	-0,086726936	6,053398332	0,796170071	0,94609342
UBA3	0,129747479	6,135122774	0,79647238	0,94609342
SART1	-0,105846241	5,058137914	0,79649466	0,94609342
PCSK7	-0,092646525	6,105521975	0,796648764	0,94609342
TAGLN2	-0,093327695	9,181837685	0,796666435	0,94609342
MTG1	-0,101018546	5,820386753	0,796874556	0,946175796

PGM1	-0,09129734	6,226293943	0,797453472	0,946470445
CHIC2	0,17907605	5,156169307	0,797506931	0,946470445
PABPC1L	-0,097150043	4,987964174	0,797746165	0,946470445
RBM22	-0,089870727	6,164053262	0,797855838	0,946470445
PHKB	-0,100560321	6,365057783	0,797948069	0,946470445
TYSND1	-0,087623762	3,950340326	0,79801122	0,946470445
MT1F	0,232002813	5,239359806	0,798094304	0,946470445
TKT	-0,098759155	7,194324986	0,798320952	0,946546658
CC2D1B	-0,08278578	5,525943588	0,798535363	0,946546658
DYNC1I2	0,130140389	7,152117498	0,798577134	0,946546658
HAS2	-0,111193265	4,218356013	0,79871381	0,946546658
ZNF92	0,260864334	4,10051087	0,798978356	0,946637738
UBASH3B	-0,105304726	5,428385917	0,799111656	0,946637738
CLINT1	0,126853014	6,841516035	0,799207135	0,946637738
MPPE1	-0,078007095	5,492959681	0,800124367	0,947396543
HERC6	-0,102311209	4,364174338	0,800341775	0,947396543
SPIN1	-0,077898827	6,81444138	0,800353927	0,947396543
ZNF251	0,232786602	4,807397778	0,800403502	0,947396543
SNRNP70	0,214858439	4,992876796	0,8006824	0,94756218
TSR2	0,149704723	6,10710183	0,801138508	0,947937444
HPCAL1	-0,095857867	7,216723946	0,801317303	0,947984505
DDX23	0,189304656	4,757607211	0,801629519	0,948047251
POLRMT	0,175308994	5,412019473	0,801661432	0,948047251
PHACTR4	0,179073297	5,027489743	0,801787432	0,948047251
KCTD17	0,195616116	5,53677737	0,802255763	0,94830046
DPYSL3	-0,102149263	6,40825236	0,802390561	0,94830046
HEATR7A	0,213617698	6,237974933	0,802423985	0,94830046
STARD7	-0,086778775	6,525203008	0,802557847	0,94830046
CXorf38	-0,107612698	2,856764351	0,802892738	0,94842953
MAP4	-0,083114738	7,809566667	0,802945252	0,94842953
KCNK5	-0,109526772	4,372062627	0,803143718	0,948499656
DDHD2	0,197148228	6,23577403	0,804396887	0,949815129
TMEM167B	-0,088352192	5,56116906	0,804908443	0,950254618
UBAP2L	-0,088459503	7,143113297	0,805108679	0,950326481
DZIP1	-0,096946608	4,992118834	0,805581812	0,950622303
CCRN4L	0,167995811	4,582439414	0,805638113	0,950622303
PTBP1	0,184641922	8,346098967	0,805784219	0,950630205
COL18A1	-0,091899514	7,34561397	0,806016168	0,950739362
CHMP4A	-0,07834311	5,081417783	0,806816786	0,951519139
RING1	0,191398963	7,020988186	0,807168118	0,9516831
EPS8	-0,08264802	6,573405628	0,807354396	0,9516831
CPSF7	-0,087513106	5,358331143	0,807374503	0,9516831
RND3	0,255123162	5,911514175	0,807798805	0,951698677
PHF21A	-0,077976432	6,095696013	0,807977729	0,951698677
SCAMP3	-0,080125776	6,440340911	0,808011913	0,951698677
GIGYF1	0,175437761	6,91405814	0,808124705	0,951698677
EML4	-0,088620606	6,506592296	0,808322608	0,951698677
RASSF3	-0,08436983	6,649848003	0,808344697	0,951698677
TCF25	-0,088728058	6,194289279	0,808371151	0,951698677
MANF	0,147268179	7,050511556	0,808565256	0,951698677

ASB7	0,137519106	3,950228217	0,808935265	0,951698677
SRP14	-0,071216623	8,70898456	0,808960757	0,951698677
CDKN2AIP	0,206583561	3,633438645	0,80899181	0,951698677
ADAM19	0,261759492	5,645586156	0,809063541	0,951698677
CRTC2	0,169063029	6,109736532	0,809202072	0,951698677
TRIM23	-0,0883063	4,058334342	0,809382229	0,951746408
MAGED2	0,141786777	8,664834842	0,809599888	0,951838213
CWF19L1	0,156136123	5,975968485	0,810054665	0,952127138
EIF1AX	-0,082715442	7,746786106	0,810476412	0,952127138
SSBP4	-0,081723807	6,020434926	0,810494006	0,952127138
SETD7	-0,084203206	6,165235398	0,810526884	0,952127138
MED13	-0,072739556	6,142947546	0,81054378	0,952127138
OCEL1	-0,095456357	3,496772143	0,811039923	0,952545855
PTK7	-0,085080465	6,178386932	0,811276712	0,952602677
RPL32P3	0,217685152	5,127122927	0,811393189	0,952602677
PSME1	-0,082080019	6,821472072	0,811518052	0,952602677
STAT2	-0,079343411	6,313475349	0,811647097	0,952602677
RPL6	0,183720729	9,947031993	0,812262905	0,952814528
PDCD7	0,165366401	4,838154231	0,81233997	0,952814528
MIER1	0,128139178	5,862127043	0,812778533	0,952814528
OGG1	0,163665923	4,541089195	0,812901938	0,952814528
NCOR2	-0,071228155	8,061272964	0,813055861	0,952814528
BTBD7	0,134494631	6,277180664	0,813073492	0,952814528
ZBTB7A	-0,075166653	7,670711565	0,813082981	0,952814528
CAST	0,13824056	7,788265047	0,813121358	0,952814528
SPTY2D1	0,134892563	5,78146342	0,813216058	0,952814528
RP9	-0,089311038	4,410588574	0,813611752	0,952814528
SLC38A1	-0,081592609	4,784720205	0,813878658	0,952814528
CENPB	0,147687088	7,468899956	0,813909117	0,952814528
CCDC7	0,153820679	4,882471545	0,814154258	0,952814528
SLC44A1	0,196568758	6,527366557	0,814273537	0,952814528
UFM1	0,169620278	3,039325922	0,814663166	0,952814528
DUSP22	0,216233902	7,021586584	0,81467124	0,952814528
DIP2A	-0,083045468	4,541629938	0,814688794	0,952814528
PRKRA	-0,076153097	6,460248788	0,814693047	0,952814528
HIBADH	0,17506109	5,506064987	0,814868738	0,952814528
PPP6C	0,120637459	6,107450274	0,814895407	0,952814528
VEGFB	-0,076894279	6,671353115	0,814919489	0,952814528
LAMP2	0,201195049	8,228386313	0,814938504	0,952814528
ABI2	0,132625266	6,736220454	0,815085767	0,952814528
BAG1	-0,081382452	5,757798438	0,815230331	0,952814528
RAB11FIP3	-0,087206533	5,843431811	0,815320835	0,952814528
TGFBRAP1	0,137944713	4,30894332	0,815659608	0,953047098
UBE2G2	-0,08353027	6,102915437	0,815985313	0,953137848
PTGS1	-0,087706895	5,359592174	0,816144513	0,953137848
URB1	0,191864603	4,651327255	0,816401301	0,953137848
PHRF1	-0,078745946	5,377438259	0,816476151	0,953137848
KLHL8	0,151882109	2,802929416	0,816500163	0,953137848
PLTP	-0,077675438	7,434109265	0,816575936	0,953137848
STX4	0,116818954	6,772385817	0,81676221	0,953192112

ZDHHC20	0,153233239	6,841922874	0,81705036	0,95336523
CLCN7	0,144540649	6,071173987	0,817293762	0,953486085
AKT1	0,194946753	6,140238985	0,817499213	0,95356263
WDR26	0,113406097	6,214753471	0,817770209	0,95371559
ETNK1	-0,06842327	5,69602511	0,818133309	0,953975895
WTIP	0,326532871	5,927028419	0,818503178	0,954041983
GTF3C5	0,122239385	5,385662399	0,818554858	0,954041983
FAM126B	0,153869671	5,70922733	0,818719283	0,954041983
DBNL	-0,06941902	6,772174061	0,818872367	0,954041983
LOC441454	-0,085943626	4,839662447	0,818918934	0,954041983
PAM	0,161764978	5,649577111	0,819217431	0,954041983
HPS5	0,174814097	5,343234525	0,819247442	0,954041983
GPN3	-0,082333036	3,884326173	0,819309261	0,954041983
FBXW11	-0,074343022	4,242069605	0,819553967	0,954163991
RPL35A	0,179856312	3,573601755	0,820122669	0,954663106
CYB5B	-0,069349899	6,909521704	0,820303227	0,95471031
ZFC3H1	-0,072784308	5,864841921	0,820472038	0,954743827
KIAA1033	0,139640324	3,068585825	0,820631152	0,954766051
ASAP3	0,190051979	5,040352798	0,821069116	0,954965337
ASXL1	0,178422702	6,662907948	0,82108253	0,954965337
CDC40	-0,066045809	5,6421222	0,821396229	0,955067835
SYF2	0,137861715	7,051896121	0,82155776	0,955067835
RBM19	-0,079776685	4,838882362	0,821590837	0,955067835
GSTP1	0,157572092	9,944685561	0,822140889	0,955360317
RFK	-0,083760673	4,269257755	0,822222682	0,955360317
ORMDL1	-0,073498688	5,058299123	0,822359471	0,955360317
BCKDK	-0,067553843	6,159324497	0,822523895	0,955360317
CFB	0,267790335	6,292319584	0,822542957	0,955360317
RNF40	-0,069414684	6,353240313	0,822708128	0,955389428
TRIP11	-0,072962002	5,30565691	0,823439823	0,955882473
CERK	0,171202618	6,695338403	0,82351448	0,955882473
FOXP1	-0,072650718	7,577060752	0,823553238	0,955882473
SNAPIN	0,133329533	6,365492142	0,82386574	0,956082451
CROT	0,14639311	3,992670181	0,824262524	0,956352339
MPRIP	0,296910168	8,141009648	0,824508835	0,956352339
CDK5RAP2	0,1361539	6,041169479	0,82451905	0,956352339
DYNLRB1	0,151782491	6,39188012	0,825353943	0,957009007
LSM10	0,149846264	4,393130464	0,825503937	0,957009007
ACVR1B	0,133258577	5,114407772	0,82550623	0,957009007
IFNAR2	-0,071734463	5,975631027	0,826021359	0,957341212
CAMK2G	-0,070907197	5,016551987	0,826133935	0,957341212
C6orf48	0,132605665	8,192090272	0,826213965	0,957341212
RNF31	-0,071450551	5,61750306	0,826917112	0,957877909
RALY	0,125944371	7,25133114	0,826958095	0,957877909
RPL23AP82	0,205417928	4,380346148	0,827145566	0,957932339
ZNF782	0,190353574	5,735166048	0,828014827	0,957994551
TAOK3	0,147239204	7,404394398	0,828325988	0,957994551
SDF4	0,121783734	8,076865829	0,828336836	0,957994551
BICD2	0,165624538	5,539185242	0,828397872	0,957994551
USP6NL	0,173378641	4,543248632	0,828519354	0,957994551

HINT1	0,154758837	8,451180371	0,828637317	0,957994551
DNAJB6	0,137071212	8,317423058	0,828764138	0,957994551
SH3BP4	-0,076293569	6,205349456	0,828905565	0,957994551
ST20	0,219386321	4,973318539	0,82894246	0,957994551
ARPC1A	-0,07424207	7,279461912	0,829071339	0,957994551
BIRC2	0,131293926	6,553165601	0,829254812	0,957994551
INTS2	-0,072177939	4,18323422	0,829474203	0,957994551
SMARCA4	0,147666552	6,381031789	0,829569287	0,957994551
MKL1	-0,080589514	4,333428248	0,829673875	0,957994551
MRC2	0,197054437	7,498808701	0,829711862	0,957994551
PRKAA1	0,12999433	5,592485667	0,829719608	0,957994551
PARN	-0,07065532	5,709385943	0,829981676	0,957994551
YARS2	-0,067397208	4,858716999	0,830084171	0,957994551
SLC35B3	-0,075151765	4,634973143	0,830302421	0,957994551
PCBP2	0,13224838	8,804893586	0,830330773	0,957994551
UBA52	0,143774985	10,22431725	0,830430652	0,957994551
IDH3G	0,123289979	7,230477734	0,830517607	0,957994551
FAM102A	0,152808063	5,843859228	0,830830952	0,957994551
CCDC57	-0,071998564	5,252557965	0,83094102	0,957994551
ZNF141	-0,072677917	5,20005544	0,831113058	0,957994551
EFR3A	0,125942723	6,779391899	0,831182613	0,957994551
PCNXL2	-0,07063824	5,516057845	0,831358553	0,957994551
MARK2	-0,06993755	6,589288959	0,831529114	0,957994551
LZTFL1	-0,06603606	5,660882886	0,831722725	0,957994551
USP38	-0,077602858	3,954576566	0,831794042	0,957994551
ZNF460	0,161908696	6,495420852	0,831812422	0,957994551
RFNG	-0,07557449	5,149317449	0,831849827	0,957994551
SLC9A6	-0,073663624	5,872887745	0,832157141	0,957994551
SMARCD2	0,108741686	6,705906325	0,832218907	0,957994551
KDSR	0,142585395	6,067628453	0,832300255	0,957994551
CBFB	-0,069591082	5,383629039	0,83240696	0,957994551
NBPF14	-0,074268702	5,614887047	0,832606304	0,957994551
RALGAPB	0,116373185	7,074039421	0,832646135	0,957994551
FBXO33	-0,069416161	4,089612811	0,832726469	0,957994551
ATXN7L3	0,137273011	6,217392919	0,832862649	0,957994551
LPCAT4	0,163310432	4,52601725	0,832964444	0,957994551
LRIG2	0,171404108	4,626243463	0,833099822	0,957994551
PCF11	-0,071261517	5,441149469	0,833522157	0,958016216
SPDYE7P	0,187546504	7,776283659	0,833742389	0,958016216
C2orf18	-0,06715251	5,826106845	0,833831747	0,958016216
EXOSC1	-0,062623096	6,797614203	0,833927524	0,958016216
RCOR3	0,220159145	6,476132727	0,833950108	0,958016216
RNF187	-0,07355376	5,914383008	0,833961616	0,958016216
ZNF607	0,162695523	3,152918298	0,834292039	0,958182386
BBS7	-0,068821835	3,745381691	0,834654077	0,958182386
E2F4	-0,062453986	6,080776355	0,834801524	0,958182386
KIAA0556	-0,071491657	4,82329225	0,834864188	0,958182386
ANKZF1	0,173531171	4,63954214	0,835118051	0,958182386
C1orf123	-0,067098817	6,208636121	0,835546158	0,958182386
GLOD4	0,193446001	4,661093242	0,83556365	0,958182386

SCYL2	-0,069656963	5,269559797	0,835745383	0,958182386
COBRA1	-0,069696153	5,169672501	0,83591155	0,958182386
CDK18	-0,071965203	6,295890502	0,835943066	0,958182386
PRPF38B	0,106772963	6,491577178	0,836028349	0,958182386
CCNK	0,112326343	7,827938949	0,83602995	0,958182386
WWP1	-0,070660728	3,830360293	0,836044797	0,958182386
TMEM106B	0,150955248	6,359947191	0,8360735	0,958182386
FLJ45445	0,208746967	2,928366373	0,836308677	0,958290854
POM121C	0,116849884	5,712333655	0,836690784	0,958315733
KRTCAP3	-0,076525065	4,47151739	0,836840744	0,958315733
MBOAT7	0,161944595	6,770852415	0,83688243	0,958315733
HMG20B	-0,070604861	5,392312946	0,836892534	0,958315733
SNRPC	0,134894923	6,033092404	0,837631164	0,958634548
MGAT4B	0,184258592	8,0268572	0,837726936	0,958634548
QKI	-0,05968003	7,58362548	0,837730083	0,958634548
SNRPB2	-0,065909904	6,47955086	0,837733285	0,958634548
USP39	-0,058992802	5,91797101	0,8379656	0,958739501
CAMKK2	-0,067081215	4,267553771	0,838816104	0,959551587
RPL39	0,177011427	9,66548276	0,839034343	0,959640253
C12orf10	0,147884881	4,78641464	0,839469858	0,959832963
LEPRE1	0,198229761	6,136410819	0,839585291	0,959832963
NFYB	-0,066886435	5,47536875	0,839788707	0,959832963
ZNFX1	-0,066355577	5,850683121	0,839900267	0,959832963
TRA2B	-0,060390925	6,320730723	0,839906627	0,959832963
HS6ST1	0,213959215	5,731966675	0,840250173	0,960050692
UNC5B	-0,067623273	7,451832338	0,840567183	0,960050692
HDAC6	0,145464958	7,510256843	0,840626931	0,960050692
PPL	0,213057835	6,129857725	0,840660314	0,960050692
MTMR1	0,134378646	5,232288827	0,841214286	0,960522474
CAPG	-0,066503528	8,423189204	0,841389825	0,960562066
GPR89A	0,143174095	5,036409542	0,841777883	0,960728963
G0S2	-0,06312343	7,616885202	0,841975987	0,960728963
SLC1A3	0,238761577	5,106017709	0,842083213	0,960728963
UBXN1	-0,056325348	7,206315433	0,842396619	0,960728963
ADAM9	0,161696395	7,03551859	0,842497464	0,960728963
ANKIB1	0,166035044	5,556326128	0,842552727	0,960728963
GSTK1	0,14020481	7,384412075	0,842605672	0,960728963
ELMO2	-0,061934835	5,354148499	0,842663136	0,960728963
EMP3	0,130922738	8,732358823	0,842974177	0,96083375
ASH1L	0,172636349	7,414450919	0,843036857	0,96083375
CD99L2	0,106936446	6,22453751	0,84323246	0,96089608
C19orf10	-0,057892898	6,803933883	0,843554068	0,961013805
SH3PXD2B	-0,064780179	6,330642471	0,843617632	0,961013805
RNPEP	0,116778206	6,580547387	0,844023687	0,961315771
TBL2	0,165310083	4,646222067	0,844355308	0,961532873
SP1	0,112814847	6,325786081	0,844604433	0,961655974
DDX52	-0,064737961	4,024280185	0,844911589	0,961721414
SAFB2	-0,057354338	4,139143513	0,845010846	0,961721414
MED14	-0,053317211	7,778068046	0,845219559	0,961721414
PLEKHJ1	-0,065748613	4,441978223	0,845519425	0,961721414

PTPN23	-0,057852917	6,316385886	0,845527167	0,961721414
YWHAB	0,132683575	8,03572197	0,84557724	0,961721414
SARS	0,123999395	6,848665492	0,845649156	0,961721414
SLC46A1	-0,066153179	3,476575029	0,845982515	0,961794261
ISG20	0,158230091	5,099721046	0,845995304	0,961794261
ATP5C1	0,121039808	7,534795189	0,84650128	0,961983759
GNB4	-0,064710978	3,922154398	0,846751593	0,961983759
CCND1	0,141585941	7,83137551	0,846956249	0,961983759
TMX3	-0,059508738	5,614775212	0,846988935	0,961983759
ZNF14	-0,062481443	3,671121197	0,84706816	0,961983759
PANK4	-0,06149716	5,33462989	0,847251935	0,961983759
MOCS3	0,12082074	4,269793085	0,847638401	0,961983759
STK4	0,120980645	6,355791401	0,847639186	0,961983759
USMG5	0,163771913	7,909080092	0,847757729	0,961983759
CHI3L1	0,361118697	7,048546531	0,84779584	0,961983759
ADNP2	-0,056784213	5,14190719	0,847909896	0,961983759
ZFYVE9	-0,058991363	5,564080736	0,84795928	0,961983759
MESDC2	0,102322835	6,58955619	0,848029161	0,961983759
PLCG1	0,146473146	5,900009367	0,848137023	0,961983759
DHDDS	-0,055183555	5,536015232	0,848531685	0,962271339
PDE7A	0,163551816	6,249117776	0,848744533	0,962352672
ARIH2	-0,057176569	5,950130968	0,849426082	0,962936959
HACL1	0,206645586	4,07957945	0,849623304	0,962936959
SRC	-0,057712589	4,647088837	0,849953971	0,962936959
TMTC3	-0,056043228	5,920634225	0,84996241	0,962936959
EEFSEC	-0,059585474	3,225762102	0,850301893	0,962936959
DDA1	-0,058088166	6,788785709	0,850385893	0,962936959
KLHL7	0,112264769	4,159467689	0,850432895	0,962936959
SYT11	-0,060278709	5,368353027	0,850443186	0,962936959
MARVELD1	0,146731412	5,641984304	0,850600018	0,962936959
CEP70	0,156038487	3,486867503	0,850671981	0,962936959
CSNK1D	0,1426722	7,363662262	0,850963045	0,962949965
GLE1	0,110986245	6,076125337	0,850994503	0,962949965
MPZL3	-0,061350359	5,209801714	0,851249489	0,962949965
SQRDL	-0,059626427	6,109813349	0,851303736	0,962949965
FAM135A	0,138717725	6,494871324	0,851465881	0,962949965
PACS1	0,102717761	6,055573001	0,851655325	0,962949965
NPIP	0,298910979	4,704851011	0,851671981	0,962949965
NEK7	-0,057069464	6,951832755	0,852062129	0,963211909
TEAD3	0,154506774	5,685528132	0,852265261	0,963211909
NEK4	0,107703272	5,304460133	0,852519822	0,963211909
MMD	0,151866243	5,516627418	0,852650014	0,963211909
STRADA	-0,05846295	4,638166702	0,852651594	0,963211909
CD99	0,10523183	8,54786749	0,852752081	0,963211909
NENF	-0,052667397	7,424442759	0,852892434	0,963211909
RFXAP	-0,060874734	3,358363909	0,853120115	0,963309499
TOR1AIP2	0,102029185	5,120738605	0,8535952	0,963317347
GANAB	0,159053073	5,484893104	0,8536078	0,963317347
KIF13B	-0,054862257	6,254917775	0,853680182	0,963317347
GTF2A2	-0,052425192	6,890569585	0,853804776	0,963317347

ENSA	0,105412561	8,026725083	0,853833413	0,963317347
NMT1	0,102654956	6,629351666	0,853996971	0,963342488
NUP98	-0,048983184	6,160687684	0,854466379	0,963582577
LMBR1	0,124609159	6,111253126	0,854518344	0,963582577
RAB11FIP2	0,113811269	6,002173348	0,854633733	0,963582577
ARL6IP4	0,115414066	7,419965553	0,855434886	0,964226793
AP2S1	0,125972682	7,15396725	0,855594666	0,964226793
H2AFY	0,10559692	7,083700794	0,855731061	0,964226793
UBXN4	0,096380087	6,915647063	0,855770722	0,964226793
DPCD	0,11196252	4,941619482	0,85599957	0,964325305
UPF1	-0,054012112	5,282867742	0,856340364	0,964549875
ACTN4	-0,054659748	9,354894335	0,856606291	0,964605107
ZNF12	-0,054452923	5,204153927	0,856672316	0,964605107
ZBTB8OS	0,102392607	5,829058771	0,857221181	0,964879845
ZNF616	0,13544382	3,009330901	0,857330371	0,964879845
TOLLIP	-0,054506009	5,156802101	0,857618022	0,964879845
TRPM7	0,118127197	5,747454724	0,857697613	0,964879845
KIF5B	0,106078974	8,275551292	0,857849484	0,964879845
MAPK12	0,162373997	5,957239019	0,857889913	0,964879845
MFHAS1	-0,055083623	5,247762212	0,858170113	0,964879845
ARPC5L	-0,055235172	4,539077378	0,85840299	0,964879845
ZDHHC6	0,108349008	5,402092163	0,858460773	0,964879845
CDC27	0,150615633	8,304867919	0,858484529	0,964879845
DUSP11	-0,049016107	5,956018066	0,858663156	0,964879845
MRPS30	-0,053182639	5,504978186	0,858750129	0,964879845
UBR4	-0,051948245	7,595052042	0,858917266	0,964879845
ZNF468	0,146013953	4,557762875	0,859186258	0,964879845
PACS2	0,12589447	6,677210729	0,859276734	0,964879845
NGRN	-0,049737174	7,7355975	0,859529139	0,964879845
PCK2	0,122257447	5,59817836	0,859560605	0,964879845
ANAPC5	-0,051336646	5,534608347	0,859747379	0,964879845
ZMPSTE24	-0,051760923	5,04930947	0,859960015	0,964879845
TTC3	0,135279813	5,543298859	0,860071655	0,964879845
GLO1	0,115553143	7,277079146	0,860351949	0,964879845
FXN	-0,053701994	3,538583471	0,860397043	0,964879845
PSME2	0,118861543	7,312667863	0,860497686	0,964879845
RABAC1	-0,051492949	7,378037992	0,860660757	0,964879845
MFSD10	0,132174382	5,657772141	0,860813953	0,964879845
PPWD1	0,10947008	5,682175124	0,86095261	0,964879845
SHROOM2	-0,055780261	3,843821357	0,860966155	0,964879845
SRI	-0,049821307	6,97290373	0,860989364	0,964879845
MLLT1	-0,051500977	6,686370724	0,861019776	0,964879845
GNPDA1	0,125756306	4,907589496	0,862150438	0,965457565
ZNF304	0,14887527	4,517839994	0,862213005	0,965457565
CNOT2	-0,048452841	5,653392968	0,862234407	0,965457565
TRAPPC6B	-0,05330749	4,949996245	0,862335137	0,965457565
APH1A	-0,049933592	6,700987555	0,862350609	0,965457565
SOX12	0,203501646	4,804003743	0,86238507	0,965457565
CLTB	-0,047623872	6,393540697	0,862526394	0,965457565
CUTA	-0,0474575	7,233553125	0,862896363	0,965677102

ITGA7	0,350645229	5,593356534	0,863005757	0,965677102
PSMB10	0,10914975	6,821224646	0,863869104	0,966065093
SCAF1	-0,048931504	5,240763552	0,864025228	0,966065093
HMOX2	-0,046159647	6,871094762	0,864039429	0,966065093
PSENEN	0,097806325	6,60352752	0,86404379	0,966065093
KIAA0528	-0,047006001	5,647638013	0,86406086	0,966065093
EML3	-0,04992339	5,270791147	0,864544295	0,966345373
NMD3	-0,044822094	6,466412554	0,864594973	0,966345373
IFI6	-0,044741634	7,517502202	0,864966743	0,966404893
CISD3	0,15153383	5,089254456	0,865069968	0,966404893
ZNF417	-0,050893182	4,341194943	0,865073393	0,966404893
FYB	-0,048005308	7,074367118	0,86530275	0,966502777
TMEM138	-0,046773948	5,825521562	0,865523497	0,966539866
DGCR8	0,142389354	5,349299451	0,86561944	0,966539866
MAPKAP1	-0,049115174	5,151507129	0,86600944	0,966817022
ETF1	-0,048340367	5,73820103	0,86634698	0,966822368
YLPM1	-0,04833083	5,131488791	0,866489636	0,966822368
ZNF408	0,140081882	3,357527814	0,866560852	0,966822368
FAM103A1	0,106255961	5,812142551	0,867087608	0,966822368
FEZ2	0,185096138	6,099476459	0,867223416	0,966822368
CFDP1	-0,048824263	4,645941453	0,86724358	0,966822368
SLC35F5	-0,046784984	5,937863022	0,867574755	0,966822368
GALK1	0,134563004	4,735810873	0,867785451	0,966822368
RABIF	-0,045016748	5,672394255	0,867906405	0,966822368
NSF	-0,048010168	5,129710651	0,867957709	0,966822368
ECM1	0,236814965	6,249369182	0,867980454	0,966822368
SOD1	-0,045758628	7,327541686	0,86833985	0,966822368
CEP135	0,115607331	4,385001334	0,868374218	0,966822368
PTP4A1	-0,042775773	7,148410316	0,868480603	0,966822368
DYRK1A	0,112258913	6,19713209	0,868691342	0,966822368
MED22	0,124167613	4,900433688	0,868741171	0,966822368
CRAT	0,13913254	5,246294231	0,86874227	0,966822368
PPP1CB	-0,044336044	8,168290058	0,868744485	0,966822368
PIKFYVE	0,119646468	6,73425662	0,86899583	0,966822368
TCF3	-0,048026064	4,456734878	0,869007642	0,966822368
SMC6	0,138547332	6,029093181	0,869102718	0,966822368
TNKS1BP1	-0,045726744	6,023563624	0,869133468	0,966822368
USP40	-0,048513205	5,192821377	0,869396205	0,966956895
RNF20	0,103657089	5,53022955	0,869875905	0,967332648
PRKACA	-0,044310233	5,663534074	0,870093439	0,967416788
FAM21A	-0,044445526	5,757809595	0,870555318	0,967615031
TBC1D10B	-0,044936503	5,450083725	0,870631637	0,967615031
TP53BP1	-0,045608217	5,417413216	0,870718222	0,967615031
FBN1	-0,041935153	6,707371071	0,870839338	0,967615031
MRPS18B	0,108800407	6,881489361	0,871066062	0,967669185
MAPK1IP1L	0,10585372	8,6161381	0,871171891	0,967669185
KIAA2013	0,108153141	7,323190463	0,871330787	0,967688052
CDC23	0,10846879	4,831923289	0,87163539	0,967868706
RINT1	0,15686073	4,83266745	0,871979042	0,968092655
ABCC5	0,19305997	5,775296618	0,872196627	0,968176591

DNMBP	-0,044269168	4,463589239	0,872558464	0,9684206
SART3	-0,043117049	5,63352074	0,872817936	0,968528676
VPS28	0,125598474	6,984479432	0,872939909	0,968528676
MRAS	0,153099014	4,74890259	0,873133932	0,968586349
WLS	0,171269096	7,360981994	0,873543614	0,968867364
NFKB2	-0,042469415	5,661896539	0,873671421	0,968867364
CAP1	0,113251263	7,565190907	0,874194664	0,969289986
HSPA13	-0,043201152	5,230384665	0,874505492	0,969429899
MAP1LC3B2	0,092778724	6,280198204	0,874605182	0,969429899
RABGEF1	0,138399023	5,838694394	0,874991726	0,969588469
FDX1	0,101701043	7,409500472	0,875539627	0,969588469
WIPF2	0,099028864	4,20865435	0,875767313	0,969588469
NDUFS3	0,084340842	6,802782858	0,875856867	0,969588469
ATF6B	-0,040718386	8,644075105	0,875900092	0,969588469
NDUFV1	0,106543459	7,194251238	0,876254585	0,969588469
METTL4	0,132955662	4,063582947	0,876375852	0,969588469
PPP2CB	-0,040618857	7,821743027	0,876603544	0,969588469
DNAJC1	-0,04160385	5,661624167	0,876684458	0,969588469
NDUFA13	0,10455929	8,619712311	0,876689672	0,969588469
DERA	0,117277549	5,023942292	0,876715249	0,969588469
PLS3	0,110871342	8,40781382	0,87684079	0,969588469
ASB8	-0,045184708	3,737896252	0,876873569	0,969588469
FMNL3	0,134662401	7,125903191	0,876914411	0,969588469
NAV1	0,131896332	5,813938844	0,877018739	0,969588469
PQLC2	0,10154971	6,048625385	0,87702327	0,969588469
DHX9	0,083753729	6,51019235	0,877357734	0,969801003
BMPR2	0,090014148	6,681937701	0,877759434	0,970087777
NCLN	-0,039743701	6,350926586	0,878100449	0,970307399
ELF4	0,111920737	5,909446719	0,878675709	0,970722274
CES2	0,103437146	5,197120902	0,878848977	0,970722274
MLST8	-0,040472295	5,640028291	0,878952122	0,970722274
CCDC6	0,097871781	6,08823936	0,879096584	0,970722274
PTDSS2	0,10972798	4,386078655	0,879187676	0,970722274
TTC1	-0,039159082	5,627352776	0,879336507	0,970729422
ADAP2	-0,039180223	5,263157489	0,879722754	0,970969759
CSNK2B	-0,036906584	7,470776998	0,879839	0,970969759
RASAL2	-0,040195954	4,83329453	0,88013512	0,971139382
FAM185A	-0,034543518	4,536670286	0,880287424	0,97115029
CXXC5	-0,03822653	5,071160062	0,880895718	0,971625542
P2RX4	-0,03921342	4,917831555	0,881178784	0,971625542
TMED7	0,110450278	7,348554774	0,881242494	0,971625542
TRAPPC1	-0,028597062	7,30095372	0,88169781	0,971625542
STAB1	0,158647386	6,975352174	0,881727012	0,971625542
DCHS1	0,216979205	6,054805133	0,881891569	0,971625542
NANOG	-0,04106459	4,877599527	0,882022174	0,971625542
CHCHD10	-0,036667454	6,451741121	0,882523246	0,971625542
POLDIP3	-0,036951434	6,013035272	0,882721781	0,971625542
KAT5	-0,036982941	5,103007728	0,88299782	0,971625542
HEATR3	0,162235057	3,545690897	0,883053117	0,971625542
IL15RA	-0,034736753	6,231667284	0,883210896	0,971625542

ANPEP	-0,023553967	5,61143817	0,883224122	0,971625542
RFC1	0,093384494	5,851078719	0,883397557	0,971625542
FH	0,124096604	4,256309959	0,883448431	0,971625542
CPT2	-0,039360615	4,367400394	0,883534261	0,971625542
PTPRK	0,097912509	5,568436159	0,883796166	0,971625542
C9orf89	0,111932311	5,251074495	0,8839373	0,971625542
RIOK2	-0,035956162	4,59003767	0,883968961	0,971625542
GBP2	0,122297685	8,238955937	0,884045233	0,971625542
STX7	-0,035756525	6,786074357	0,884086698	0,971625542
FAM126A	-0,032136224	6,355519831	0,884103214	0,971625542
PSMB9	0,126197012	6,302750261	0,884143101	0,971625542
RAB2A	-0,035643257	6,286941137	0,884214526	0,971625542
CLK2	-0,031516499	5,918565767	0,884280409	0,971625542
PYROXD1	-0,036006224	5,578205905	0,884605756	0,971826429
CHIC1	-0,041554523	3,639002836	0,885593928	0,972662969
GNAI2	-0,034516712	10,41351278	0,885652497	0,972662969
MEST	0,183869617	7,42619197	0,886570083	0,97312468
COX6A1	0,094847858	10,15076929	0,88662302	0,97312468
AGPAT3	0,085910453	6,055252141	0,886626498	0,97312468
PTPMT1	0,097774788	5,953342305	0,886643736	0,97312468
BBX	0,092735644	6,76028538	0,886959101	0,973314147
TOX4	0,077084987	7,538645876	0,887158065	0,973335702
AMOTL1	-0,030914139	5,429774334	0,887264221	0,973335702
EPCAM	0,183690556	6,626203958	0,887822705	0,973791705
WDR47	-0,033894628	4,455255222	0,888043678	0,973877427
SCMH1	-0,032918606	6,130570377	0,888258381	0,973956247
RRP7B	-0,029437863	5,759557151	0,888534232	0,974102078
BRD3	-0,033569484	4,882655038	0,888694017	0,97412064
DDX41	-0,034478375	5,249708283	0,889062094	0,97429243
ITFG1	0,154447387	8,004323445	0,88919817	0,97429243
CORO1A	-0,029515699	5,738557091	0,88931244	0,97429243
THG1L	-0,033950773	4,828460537	0,889422257	0,97429243
SF3B1	0,099185736	9,207564275	0,889960548	0,974320338
MEF2A	0,096386004	5,104333808	0,890311471	0,974320338
ALG14	0,108844371	3,827387899	0,890520679	0,974320338
NDUFB1	0,101937951	7,402651695	0,890556228	0,974320338
MAPK13	0,133955384	5,924116559	0,890661744	0,974320338
STRN	0,118280358	5,028462444	0,890746393	0,974320338
USP35	0,116420987	5,167451579	0,890992464	0,974320338
RNF103	-0,036024217	4,207298302	0,891048155	0,974320338
TMEM203	0,104500594	5,785963961	0,891071318	0,974320338
NDUFA12	0,085595536	6,710553217	0,891162427	0,974320338
HOMER3	0,172643877	7,229880344	0,891210992	0,974320338
TCIRG1	0,115169514	7,661204922	0,891269393	0,974320338
CASD1	0,100882526	5,588208949	0,891443601	0,974320338
PHF5A	0,089113299	5,236506071	0,89174141	0,974320338
FAM104A	-0,03298981	4,643533607	0,891902066	0,974320338
CTBP1	-0,030929965	8,245344017	0,891945501	0,974320338
CTSC	-0,029584628	8,287293942	0,892041289	0,974320338
SYS1	0,088379045	5,989885038	0,89228638	0,974320338

DDB1	-0,031216572	6,97443307	0,892386757	0,974320338
SSBP2	-0,03120728	6,36291753	0,892541135	0,974320338
PTGES2	-0,028810731	4,657048179	0,89269117	0,974320338
CISD1	0,101188054	5,87031615	0,892731304	0,974320338
SRPX2	0,130769183	5,692655454	0,892734048	0,974320338
SHPRH	0,08844358	4,407134943	0,893043745	0,974502368
DMXL2	-0,030531879	5,508607903	0,893344904	0,974675025
BMP2K	0,092346675	5,946845145	0,893595832	0,974717408
FLJ10038	-0,030389897	5,561129368	0,89373555	0,974717408
ACTG1	0,114122424	12,41994688	0,893891956	0,974717408
FAM58A	0,096081978	4,24043444	0,893955517	0,974717408
PRPSAP2	0,104057622	6,671289239	0,894261729	0,97488501
GSK3A	0,088096776	7,578742759	0,894599166	0,97488501
SUPT7L	0,105823033	4,872100395	0,894698117	0,97488501
RRAGB	-0,03144805	5,07271553	0,894731733	0,97488501
FAM172A	-0,030385208	5,496236213	0,894824061	0,97488501
RAB21	-0,030126424	5,798876125	0,89497057	0,974888869
ROCK2	0,091782898	6,856320262	0,8952406	0,974930044
DECR2	0,114723327	5,961000281	0,895294315	0,974930044
SDC1	0,251382609	5,492005675	0,895951638	0,975340618
ZC3H11A	0,078733994	7,125988438	0,896206194	0,975340618
DCUN1D4	0,105873109	4,766577538	0,896298931	0,975340618
C1orf50	0,102568155	4,465211106	0,896403446	0,975340618
NEK9	-0,029097077	5,237087674	0,896628762	0,975340618
RHOC	0,10822062	8,331219168	0,896803909	0,975340618
CTPS2	-0,025354247	6,676999942	0,896846851	0,975340618
POLR3E	0,107225323	4,083561864	0,896877675	0,975340618
IFT80	0,127176724	5,661795548	0,897045572	0,975340618
RASAL3	-0,026678967	5,100333445	0,89710168	0,975340618
MTMR6	0,067029984	5,339363298	0,897356627	0,975420194
DHODH	-0,029442973	4,383106742	0,897754691	0,975420194
ZNF692	0,121646994	5,906696943	0,89778582	0,975420194
GUSB	-0,028160042	6,111048592	0,897922378	0,975420194
HPS6	-0,029545535	5,275559026	0,898021203	0,975420194
ACD	-0,028477912	4,738657585	0,89803314	0,975420194
MAFB	0,134482034	6,568027223	0,898217838	0,975455326
MT1X	0,154698428	6,561103843	0,898351583	0,975455326
ZSWIM6	0,109046422	6,351497744	0,898602769	0,975572725
EEF1D	-0,024972713	7,833788927	0,899129381	0,975989056
CTSB	-0,021469033	9,095001178	0,899470491	0,976203928
FABP5	-0,004615633	8,468683734	0,899834612	0,976443701
SEC23B	-0,027142761	4,215296737	0,900290675	0,976783152
GNB1	0,101118973	7,492705311	0,900680022	0,977007775
UBR2	-0,027000181	5,270505866	0,900784262	0,977007775
SLU7	-0,026699084	5,755405791	0,901462218	0,977491992
GLS	0,07366718	7,961716085	0,901517398	0,977491992
TYMP	0,110845908	8,615264613	0,902428299	0,977564845
PI4KA	0,090494029	6,115178484	0,902697804	0,977564845
KIF16B	-0,01901168	4,450019926	0,902787099	0,977564845
RBPMS2	0,001012764	6,176866909	0,902906132	0,977564845

USP33	-0,024427633	6,686677624	0,902916542	0,977564845
C11orf57	0,092653668	5,375243019	0,902955653	0,977564845
TMEM9	-0,024469801	5,789272414	0,903018022	0,977564845
ASNS	-0,022323277	5,651419098	0,903042842	0,977564845
PDGFA	-0,014224621	7,597894211	0,903318198	0,977564845
ATP5G2	-0,023713776	8,379402278	0,90349098	0,977564845
RELT	0,102368734	3,206742949	0,903577714	0,977564845
RNF144A	-0,016665821	4,505282985	0,903806897	0,977564845
NOMO1	0,088162926	6,679297013	0,904187939	0,977564845
KIAA0100	-0,02008049	5,980640177	0,904231635	0,977564845
ACAP2	0,08201157	6,139608524	0,904299794	0,977564845
RPS27A	0,084341099	10,29513019	0,904355524	0,977564845
PTPN4	0,090973704	6,273811082	0,904381277	0,977564845
PPIL2	-0,021169308	4,631062622	0,904463523	0,977564845
LUC7L	-0,023655217	6,936902613	0,90446879	0,977564845
MYD88	0,073043361	5,488854579	0,904595683	0,977564845
RPL13AP20	0,07314221	8,843364101	0,904655538	0,977564845
HIGD1A	0,107465829	3,831137311	0,905286637	0,977564845
SRA1	-0,016056603	4,64729289	0,905325475	0,977564845
GALNT11	0,091851622	5,341323353	0,905442818	0,977564845
C5orf24	0,114627422	6,797441497	0,905447278	0,977564845
B3GALNT2	0,146770904	4,962943303	0,905522323	0,977564845
PLOD3	0,155570415	5,361873797	0,905661203	0,977564845
ZBTB11	-0,023906497	5,223204183	0,905710153	0,977564845
MBD2	-0,022236836	7,756344957	0,905821591	0,977564845
EDC4	-0,021512621	4,698665448	0,906110466	0,977564845
MX1	0,143882838	6,568278826	0,906131866	0,977564845
ADI1	-0,022146996	6,196489384	0,906309541	0,977564845
FAM32A	-0,023625348	6,149077061	0,906315435	0,977564845
DHX35	-0,021568297	4,780428361	0,906985845	0,97813324
FXC1	0,096304065	7,01747082	0,907598254	0,978599144
ENC1	-0,017430097	6,046773513	0,907745549	0,978599144
SCAP	-0,020491403	7,011772273	0,907848392	0,978599144
SDHAF1	0,090803256	5,257331701	0,908005189	0,978613463
RRN3	0,096248916	5,634545305	0,908736991	0,979247399
RBM27	-0,024489879	5,486466718	0,909288381	0,979299641
TAF4B	-0,025098493	2,843326819	0,909349276	0,979299641
TBCB	-0,023158422	5,447679686	0,909415072	0,979299641
MPZL1	-0,020064614	5,566685571	0,909433111	0,979299641
SOCS7	0,07669067	5,300558854	0,909533497	0,979299641
STC1	-0,008821281	6,393419265	0,909924658	0,979299641
SMARCA5	-0,007499058	7,083124748	0,909946785	0,979299641
PKIA	0,157619135	5,149560022	0,910013299	0,979299641
PML	0,095113699	5,837788956	0,910199533	0,979299641
SYVN1	0,079594144	5,758429477	0,910335845	0,979299641
KIAA1377	-0,025736279	3,964863664	0,910448055	0,979299641
PTPLB	0,098432782	5,209345933	0,910508832	0,979299641
LRP11	-0,009103986	5,80349675	0,91081652	0,979400814
DHCR7	0,144307544	5,371899812	0,910890154	0,979400814
NDUFA10	-0,020876658	6,02059182	0,911443983	0,979841797

GBF1	-0,019630164	6,315926048	0,911841912	0,979979775
SAE1	0,132659107	5,645171197	0,912035727	0,979979775
ATRN	0,087317966	5,726969732	0,912346468	0,979979775
AKAP11	0,081471834	5,460617339	0,912352941	0,979979775
PARP14	0,070403443	7,674691305	0,912360857	0,979979775
MAN2B2	-0,017617217	5,485952294	0,912434608	0,979979775
FAM165B	-0,016057885	5,818850835	0,912596544	0,979999344
SLAIN2	-0,019535618	7,495107409	0,912791661	0,980054532
TNKS2	0,070820325	6,746123129	0,913013784	0,980138695
CORO1C	0,077637855	7,654310666	0,913231317	0,980198527
BIN2	-0,017219581	5,047843876	0,913433773	0,980198527
ZNF506	-0,011302894	6,262440318	0,913541722	0,980198527
ZNF136	-0,020109828	3,742978461	0,913644499	0,980198527
PDCD6IP	-0,018221636	7,731424546	0,9139762	0,980269753
EXOC1	-0,021807512	4,389939896	0,9140681	0,980269753
COL6A1	0,184089247	7,52369015	0,914142156	0,980269753
GPR107	0,068486651	6,082871517	0,914297674	0,980282364
SURF2	-0,02111847	3,66103983	0,914734635	0,980515803
DCTN5	0,072160926	5,928882852	0,914993761	0,980515803
ATE1	-0,026024995	3,589134052	0,914994972	0,980515803
PLK1S1	-0,015513836	4,615319659	0,915217434	0,980515803
N6AMT1	0,123678138	4,435063998	0,915234358	0,980515803
SEC61A1	0,086622758	8,881824085	0,915614677	0,980769162
PTEN	-0,005816101	7,711870367	0,915815377	0,980797901
XRN1	-0,013003002	5,105269446	0,915959081	0,980797901
GNG10	0,084193554	7,931407342	0,9162863	0,980797901
HMGN4	0,076805462	5,822657434	0,916312519	0,980797901
MMGT1	-0,014788019	6,123439361	0,916360673	0,980797901
APPBP2	0,06828815	6,611736722	0,916636071	0,980925489
RSRC2	-0,018405986	6,199051713	0,916767582	0,980925489
AKAP1	0,15940248	5,978169901	0,917087355	0,981113692
B4GALT5	0,079557981	6,139948583	0,917287027	0,98117337
SMAD4	0,08231702	5,48402267	0,917586594	0,981196477
MRRF	-0,016646344	4,793274835	0,917596412	0,981196477
C19orf6	0,094132695	7,281194575	0,918909607	0,982166628
TCEA3	-0,008811658	6,717709322	0,918946579	0,982166628
POU2F2	0,115882762	5,122433347	0,918995339	0,982166628
HSBP1L1	0,111718702	4,057467462	0,919346552	0,982166628
TERF2	0,063327058	5,913251577	0,919394459	0,982166628
GNS	0,071752824	7,176338853	0,919681857	0,982166628
PEX3	-0,014084683	4,84564904	0,919749735	0,982166628
ITPR2	0,086240523	5,962447245	0,919864412	0,982166628
VPS37C	0,091683436	5,021023973	0,919931458	0,982166628
WASF2	0,089286213	6,143205059	0,92000897	0,982166628
AKR1A1	-0,016810307	6,762963788	0,920088051	0,982166628
TXNL1	0,072195772	5,566338156	0,920564149	0,982521041
DIS3L	0,091729878	5,353839701	0,921230532	0,982992416
IL4R	0,075782294	6,635990649	0,921294109	0,982992416
PPTC7	0,070366152	6,372767081	0,921513042	0,983025778
PPAPDC1B	0,095578055	4,820006359	0,921613697	0,983025778

ETFA	-0,015823478	5,816374977	0,922029081	0,983172261
ZC3HAV1	0,069298478	5,637721232	0,922039391	0,983172261
ELMO3	-0,013353581	4,545360813	0,922235964	0,983228117
DNAJC15	-0,001856571	5,616403545	0,92291171	0,98379474
C20orf111	0,075590611	5,541279436	0,9230719	0,983811704
FAM160B1	0,061197776	5,411200914	0,92397546	0,984620826
PLEKHA4	-0,007660807	4,892258487	0,924120083	0,984621069
PCMTD2	-0,010941362	6,142496592	0,924830644	0,985082654
VDR	0,106808745	5,977673413	0,925157952	0,985082654
ELK3	-0,009268652	7,782742628	0,925233463	0,985082654
XRCC1	-0,013831889	4,901158166	0,925236059	0,985082654
TBC1D14	-0,012803408	4,969606093	0,925297614	0,985082654
CTR9	0,083079927	5,152230153	0,925420074	0,985082654
IKBKAP	-0,009060203	5,018082747	0,926415529	0,985988371
PGS1	0,083405139	5,974327861	0,926819899	0,986041876
STK38	0,092084763	4,943029196	0,926845855	0,986041876
MAN2A1	-0,000936347	6,366833475	0,926899608	0,986041876
MED9	0,122821577	4,262504263	0,927333856	0,986349956
FBXO7	-0,012075018	7,058309776	0,927608189	0,986487873
ZNF613	-0,011402573	3,591252603	0,92797667	0,986725856
ZBTB33	0,112942025	5,950695221	0,928454394	0,987003895
XPC	-0,011151577	5,929561707	0,928793324	0,987003895
MORC3	0,070462964	6,292630919	0,928989123	0,987003895
RAB3GAP1	-0,010374292	6,122904351	0,929283805	0,987003895
COX17	-0,007570775	7,079159793	0,92930143	0,987003895
TRPC4AP	0,067489603	7,093933526	0,929381416	0,987003895
DRG2	-0,006482393	3,982148618	0,929514537	0,987003895
CYP4V2	-0,008004059	4,876669137	0,929622375	0,987003895
RNMT	-0,008578115	3,333760554	0,9298711	0,987003895
CD300A	-0,010849101	4,848679008	0,929923635	0,987003895
NUAK1	0,086828849	6,07568006	0,92993362	0,987003895
CWC25	-0,010554764	5,628273532	0,930487965	0,987003895
TMEM185A	0,091465724	4,651152053	0,930529439	0,987003895
UBTF	-0,009385918	5,771241866	0,930664289	0,987003895
KIAA0196	-0,009468282	5,695749559	0,930673906	0,987003895
SIN3A	-0,001031816	5,935108676	0,930820497	0,987003895
ZNF121	-0,004311203	5,636720035	0,930892372	0,987003895
ZMIZ2	-0,011726948	6,977968073	0,930999869	0,987003895
ICT1	-0,010183262	5,483425004	0,93112992	0,987003895
USP11	0,084217099	7,205993951	0,931454703	0,987003895
DDX18	0,067565536	4,303516183	0,931688632	0,987003895
TMEM55A	-0,010351613	4,960823449	0,931745283	0,987003895
SERGEF	-0,006297096	4,925817376	0,931771589	0,987003895
NDUFA6	-0,007832629	6,969619827	0,932120383	0,987003895
DST	0,104064758	7,36615015	0,932191807	0,987003895
RPL12	-0,00691136	9,814973556	0,932490479	0,987003895
CEP350	-0,009752924	5,889957763	0,932639208	0,987003895
POLE3	5,12563E-05	5,710312203	0,932733114	0,987003895
RAB40C	-0,004869609	5,807423971	0,932774792	0,987003895
PRKX	0,15433051	5,643911771	0,933028421	0,987003895

USP15	0,093504558	5,41915877	0,933175193	0,987003895
LOC493754	0,102274647	4,317903351	0,933226584	0,987003895
LAP3	0,00047134	7,556929492	0,933355681	0,987003895
CASP8AP2	0,094365519	5,317968931	0,933410882	0,987003895
PCCB	-0,003697474	4,574393511	0,933451311	0,987003895
ZBTB45	0,077083714	4,787769422	0,933636545	0,987003895
SPCS2	-0,006803788	7,602087532	0,933689588	0,987003895
MSL2	-0,002631712	6,81592676	0,933905403	0,987003895
TET2	-0,006419206	4,923734399	0,934058153	0,987003895
WWTR1	0,090225347	8,693468043	0,934342404	0,987003895
LYRM2	-0,006902188	5,865986607	0,934430894	0,987003895
CSTB	0,100411442	10,4043032	0,934707068	0,987003895
JHDM1D	-0,004605267	6,220247639	0,934801061	0,987003895
VTI1B	-0,003769057	5,786324981	0,934808743	0,987003895
GEM	0,09157968	6,520112082	0,935220235	0,987003895
PLAT	0,121159278	6,462208703	0,935231268	0,987003895
HGS	-0,003978961	4,812121363	0,935356506	0,987003895
LSM7	-0,003567217	6,669340115	0,935389578	0,987003895
DCAF7	-0,005006044	5,787457021	0,935469114	0,987003895
RPL36	0,07301626	10,62965433	0,935475315	0,987003895
FAF1	0,061466943	5,745675502	0,935795156	0,987058104
SNX17	0,064210319	6,729892844	0,935848829	0,987058104
KIAA0232	-0,006017253	4,74410601	0,9360489	0,987058104
ABHD3	0,090942422	5,217295727	0,93627414	0,987058104
UHRF2	-0,004218054	6,63793573	0,936276167	0,987058104
NFKBIL1	0,091960119	5,320708155	0,936395201	0,987058104
COX6B1	0,077106756	8,796929223	0,936830522	0,987364347
EEF2	0,080057278	10,77119647	0,93705501	0,98738445
MBTPS2	0,085075707	4,63610763	0,937236768	0,98738445
GGPS1	0,118503302	4,97941525	0,937283993	0,98738445
AURKAIP1	0,081183617	6,344254828	0,937658069	0,987625946
ANKRD28	-0,002415066	4,451721171	0,938033278	0,98786856
ZNF277	0,069156578	5,272352826	0,938661918	0,988377952
RAB27A	0,09342908	6,527074508	0,939172569	0,988552342
ZNF830	0,082031162	4,748417515	0,939420273	0,988552342
TCTN2	-0,001967888	5,032350274	0,939508718	0,988552342
XRCC6	-0,004458701	8,399463387	0,939625927	0,988552342
SCCPDH	-0,004120763	6,397233666	0,939688243	0,988552342
MFGE8	0,010871324	8,018294328	0,93970625	0,988552342
SUPT6H	0,066625866	7,225660689	0,939842328	0,988552342
NRIP1	-0,00629589	6,460830653	0,940566545	0,989161516
MDH2	0,071324463	7,343023992	0,940962231	0,989405806
USP10	0,06935565	4,964039372	0,941204169	0,989405806
COX7C	-0,00510563	8,034459329	0,94123412	0,989405806
ATP13A1	-0,000356299	6,173795478	0,941383991	0,989410825
MAX	0,075092207	5,169433861	0,942401672	0,990124641
EIF2C4	0,002053484	6,179395503	0,942527603	0,990124641
ARHGAP23	0,004748131	7,254187711	0,942636995	0,990124641
KIAA1598	0,017284873	6,011145823	0,94264396	0,990124641
MPV17	-0,002943758	5,674573219	0,943558879	0,990852737

MTMR12	0,059002703	5,834713887	0,943627756	0,990852737
ATF6	0,006921133	5,862917076	0,943893726	0,990862705
DCTD	0,069386957	7,163957839	0,943927868	0,990862705
C5orf22	0,001812268	4,405781531	0,944166044	0,990960174
WIZ	0,062916326	6,993584618	0,944711526	0,990978969
U2AF2	-0,000876082	6,594261388	0,944794259	0,990978969
EAF1	-0,001106156	5,480588519	0,945164047	0,990978969
TMEM165	0,067107398	6,903738972	0,945212295	0,990978969
HDAC2	0,067632102	6,333083515	0,945344222	0,990978969
UBE2D3	-0,00271034	8,957228471	0,945685064	0,990978969
SEC22B	-0,000239942	7,136229509	0,945806825	0,990978969
DDX56	-0,00067742	4,910946548	0,945809084	0,990978969
HMGN1	0,004898951	6,38515487	0,945991767	0,990978969
EVC	0,062360161	5,518450731	0,946021304	0,990978969
PPDPF	0,092001168	9,613640136	0,946064276	0,990978969
ZNF45	0,099222429	3,462851242	0,946163947	0,990978969
UBE3A	0,061623732	5,981582523	0,946227224	0,990978969
C2CD3	0,070220613	6,285158885	0,946241815	0,990978969
SDHA	-0,000731819	5,896350616	0,946363843	0,990978969
CDH11	0,019133536	8,130712573	0,946610818	0,991085393
PLAUR	0,076906954	7,880689549	0,947059281	0,991402708
CHMP7	0,053407334	6,06580011	0,947402394	0,99145296
PHC1	0,006580457	5,861888229	0,947645461	0,99145296
PPP4R1	0,057617436	6,655807476	0,947652232	0,99145296
HEXIM1	0,065757265	6,520825792	0,947688868	0,99145296
KIAA1432	-0,000519834	6,598538714	0,947951994	0,991576108
DGAT1	-0,006314247	3,211061975	0,948491625	0,991788285
LPIN2	0,087962838	5,952775995	0,948596126	0,991788285
TNRC18	0,087238198	6,035847274	0,948673757	0,991788285
LTBP3	0,108234709	8,52888206	0,948736616	0,991788285
VPS16	0,086268754	5,351345368	0,949148254	0,992066515
GNG5	0,00470948	8,492505046	0,949572841	0,992358192
IDH2	0,00978726	6,156331039	0,949888008	0,992465251
OVCA2	0,069937048	4,689513412	0,949966372	0,992465251
TCF12	-0,002309169	5,822229027	0,950169947	0,992525868
ETV6	0,009548695	6,928169098	0,950860885	0,9929477
SEPT10	0,001757581	7,158459497	0,950865007	0,9929477
GATAD2B	0,004339412	6,270583644	0,951220143	0,993166461
EXOC2	0,00146504	6,381889186	0,951389797	0,993191522
ADO	-0,003452737	4,991771011	0,951864099	0,993495338
RASL11A	0,108355718	6,620129502	0,951972216	0,993495338
WDR61	0,000881319	6,255651869	0,952246996	0,993628415
DHTKD1	0,084671554	4,333423944	0,952391161	0,993628415
KIAA1737	0,014434835	6,305203185	0,953545487	0,994680538
ZNF276	0,099885026	5,808393864	0,954018315	0,994895888
CCNY	0,077466943	7,180116528	0,954043733	0,994895888
HEBP1	0,008841067	5,92622454	0,954786721	0,995096916
GOT2	0,073676202	5,687245609	0,954871437	0,995096916
RPS25	0,065779455	10,67983458	0,954931815	0,995096916
PSMA5	0,073423325	6,577868239	0,955020038	0,995096916

PRRC1	0,054243437	7,87726737	0,955225243	0,995096916
CARS2	0,110152094	6,243463852	0,95525605	0,995096916
MAP2K1	0,059905324	6,276745213	0,955258016	0,995096916
GNA13	0,005956582	8,341660545	0,955527187	0,995158977
FTSJD1	0,060443839	4,547933538	0,95564878	0,995158977
CEP68	0,066036831	5,115157182	0,95575541	0,995158977
PEG10	0,04734169	6,860192879	0,956043521	0,995306988
PPFIA1	0,002396012	6,212574911	0,956318654	0,995441445
SNX11	0,064028275	4,965020275	0,956487862	0,995453239
ATL3	0,056784816	6,764466866	0,95662195	0,995453239
UTP3	0,055164311	5,447009856	0,956772247	0,995457728
CCDC50	0,062732096	7,487371923	0,957159556	0,995708774
TM7SF3	0,065097364	6,11023859	0,957485778	0,995834651
SSU72	0,003015747	7,756909746	0,957572637	0,995834651
VPS37B	0,059453039	5,376426708	0,957800146	0,995852506
RPL19	0,008568169	10,62101102	0,958020572	0,995852506
CEP290	0,074730758	4,318239059	0,958027928	0,995852506
EID1	0,008295374	6,909101828	0,958638141	0,996334932
RPL18	0,070829968	10,61101109	0,958815168	0,996367058
DAPK3	0,075024189	6,209990348	0,959328758	0,996386982
MED19	0,063140198	5,790637554	0,959347523	0,996386982
C3orf37	0,056711743	4,274759338	0,959403456	0,996386982
ZBTB41	0,055158914	4,496642444	0,95943808	0,996386982
DAD1	0,068142063	8,54279572	0,959564938	0,996386982
EIF1	0,006241897	9,514197188	0,960280033	0,996742854
USP22	0,010050938	7,00498122	0,960285541	0,996742854
TMEM214	0,053719268	7,464298653	0,960346173	0,996742854
ATHL1	0,085288637	5,994269321	0,961026629	0,997057167
RNF25	0,054639653	5,735493653	0,961220753	0,997057167
FAM54B	0,064745423	6,125123676	0,961221337	0,997057167
PBX2	0,016407077	7,174099053	0,961233879	0,997057167
SSNA1	0,003657744	6,901091289	0,961645201	0,99733211
FUT11	0,017317701	6,752490257	0,96210177	0,997653888
TMOD3	0,014989661	7,928509651	0,962831958	0,997955335
COPS7B	0,063275992	4,767225988	0,962934287	0,997955335
ARGLU1	0,090737517	6,396819118	0,962934746	0,997955335
TM2D1	0,007563232	6,25345452	0,962977872	0,997955335
ANGEL2	0,064235438	5,441811392	0,963795793	0,998555309
CD47	0,020568859	8,023260533	0,963849691	0,998555309
C11orf58	0,041725777	7,857251834	0,965008185	0,999356888
PSIP1	0,105076969	5,236040649	0,965021272	0,999356888
GAA	0,018830455	6,577326106	0,965063075	0,999356888
WDR41	0,047876536	4,0385583	0,96532961	0,999481113
NDFIP2	0,059114077	5,040344121	0,965554323	0,999562005
FLNA	0,081734507	9,707554627	0,965897286	0,999765269
MFSD11	0,043102992	5,392459502	0,966216539	0,999943934
TARBP1	0,101087565	5,796540953	0,966375732	0,999956922
MBOAT1	0,066155416	4,739901716	0,966905822	1
MRPL18	0,047409978	5,848051782	0,966915638	1
RELA	0,051792181	6,461484964	0,967066715	1

SNF8	0,013226418	6,605782699	0,967179772	1
GULP1	0,036502429	6,476491745	0,967386981	1
EIF1B	0,012001665	7,08464313	0,967570558	1
FN3KRP	0,089035241	4,453420776	0,967650555	1
HIBCH	0,020794871	6,007958362	0,967714976	1
ZNF24	0,007828866	5,939588879	0,968152189	1
STXBP5	0,055872134	4,914370268	0,968296123	1
LAMP1	0,182023152	6,300694462	0,968989729	1
BIRC6	0,055210518	7,281879602	0,969076228	1
BCAS2	0,021083293	7,41364698	0,969837583	1
FADS2	0,056647944	6,951342231	0,970093476	1
MLEC	0,011889464	7,171007667	0,970658197	1
TAF12	0,056561295	5,85915157	0,97081901	1
PSMF1	0,043823542	7,508338223	0,970862382	1
MT2A	0,113070269	10,93034125	0,970907747	1
COMMD8	0,092791939	3,888314316	0,970933717	1
TTC17	0,063446507	5,537779465	0,971380725	1
NAP1L5	0,068253233	4,666725875	0,971514163	1
TMUB1	0,054516506	6,58938836	0,97166906	1
MED6	0,044946625	5,739885628	0,971976472	1
SRP9	0,045021841	8,765537618	0,972453574	1
ARPC3	0,049543708	7,916302062	0,973328372	1
MAZ	0,019843345	8,255168809	0,974569976	1
RPS10	0,011750391	9,411272054	0,974770977	1
C8orf33	0,093766822	4,683114255	0,974870275	1
PPIA	0,069675068	9,592940194	0,974914919	1
LPIN1	0,048200267	4,29959837	0,974935211	1
DTNB	0,05970651	6,018524567	0,975234903	1
RUFY2	0,058682571	4,0732145	0,975808462	1
VKORC1	0,045170239	8,119983401	0,975958015	1
NUDT5	0,014115069	6,018784149	0,976216849	1
RPUSD3	0,049266428	4,587639997	0,97636152	1
GORASP2	0,036624499	5,866123555	0,976438905	1
KIAA0226	0,04971256	7,094627696	0,976729276	1
TRIM33	0,053841085	4,661219071	0,976932285	1
NEDD4	0,05688735	5,34873877	0,977689477	1
MLL3	0,062369841	6,114049424	0,977760294	1
EPHB4	0,062521535	6,705221459	0,977902793	1
SUOX	0,049155322	4,34577586	0,977979583	1
RAE1	0,050658236	5,249136981	0,978700796	1
FAM127B	0,018199694	7,453877832	0,978956332	1
VSIG10	0,063063572	3,478760338	0,979565118	1
GRAMD1A	0,043253877	6,302773339	0,979875033	1
CTDSPL2	0,065394888	6,193185883	0,979896679	1
FAM106A	0,111770105	5,653393782	0,980220977	1
CALM3	0,016155501	8,672115191	0,980242673	1
USP12	0,025074028	6,887656418	0,980426463	1
PAPD4	0,081101436	5,054493883	0,980434541	1
CARHSP1	0,023969745	6,3311519	0,980500435	1
RPS11	0,014779066	11,78284243	0,980923195	1

POLR2E	0,034380993	7,17436198	0,981036831	1
OS9	0,043211589	6,847019412	0,981958062	1
C6orf57	0,049100344	4,28759454	0,982042943	1
H2AFV	0,015790138	7,965133032	0,982181006	1
LANCL2	0,019548353	5,945288433	0,982426843	1
TCTN1	0,052336101	5,143829454	0,982484961	1
SNRNP40	0,042878952	5,496353697	0,982829894	1
OAT	0,043749632	5,984370585	0,982859639	1
AP3M1	0,025018222	6,515564758	0,983014788	1
MRPL28	0,037378743	5,744657876	0,98304321	1
DUSP5	0,086480605	5,55072239	0,983071535	1
OSBPL3	0,035026307	5,963412109	0,983212726	1
POLR2G	0,047679507	5,746384378	0,98336791	1
GPS2	0,034936617	5,788800366	0,983570225	1
PURB	0,047335573	6,106750064	0,983688617	1
MAPK1	0,033889973	6,990553407	0,983829201	1
NOMO2	0,049316021	5,077446079	0,984158374	1
SETD1B	0,064185179	5,645630744	0,98498282	1
KIF1B	0,037572606	5,893836163	0,985000185	1
LAMC1	0,019788765	7,984291114	0,985460313	1
DENND4B	0,058417752	5,359641833	0,985557507	1
NRBP1	0,045540246	5,744301912	0,985615407	1
SEC24C	0,038985816	6,108354527	0,98591996	1
IQCE	0,052515407	4,13441077	0,986260266	1
GLRX5	0,038558325	6,693398996	0,986264959	1
COX5B	0,037535149	8,223391518	0,986816571	1
AMPD3	0,053497491	4,749164362	0,986878704	1
ZFYVE26	0,017448251	6,984048305	0,987546709	1
RAB6A	0,02226214	8,48921028	0,987803894	1
DPP7	0,054178622	6,009169017	0,9881438	1
C5orf15	0,045197547	7,362240727	0,988294353	1
FNBP4	0,034668296	5,721204103	0,988361609	1
LONP2	0,038601133	7,141456984	0,98841859	1
PARVB	0,04415719	4,787247973	0,988612062	1
MRPL27	0,052932986	4,157198153	0,988973084	1
CALM2	0,038775153	9,058281238	0,989446941	1
LOC550112	0,0578621	4,098952642	0,989633472	1
NLGN2	0,070833857	5,904046367	0,989786785	1
TNRC6A	0,034112849	5,960131272	0,989850166	1
SH3GLB2	0,056071021	6,220011332	0,99046942	1
GLT25D1	0,05498776	7,267799927	0,990734479	1
PPARA	0,046932605	3,929506769	0,990794489	1
NOTCH3	0,119195216	7,972228473	0,990803567	1
KLHDC2	0,035790463	7,192602889	0,990978577	1
ROMO1	0,024577116	7,110658236	0,991810709	1
EIF3F	0,03332385	8,60952462	0,992356218	1
PIAS4	0,064896843	4,756165962	0,993238046	1
FOXJ3	0,036287285	6,30879027	0,993245669	1
CCDC71	0,046148468	4,582796665	0,993729404	1
UHMK1	0,032560355	5,654724176	0,993929476	1

PHF10	0,024603305	7,402989526	0,994056934	1
API5	0,026119115	6,169847166	0,994517513	1
NOP10	0,029363306	8,258558813	0,994592239	1
RPL13A	0,026170552	10,43866085	0,9947424	1
SMAD2	0,022648155	7,197421484	0,994958126	1
SDHC	0,022638572	7,645320808	0,994999374	1
MT1E	0,102087756	6,47046732	0,995134266	1
KEAP1	0,045513329	6,270182518	0,995238806	1
FMNL2	0,033930972	6,921300679	0,996447825	1
POLE4	0,047331466	5,09889603	0,996644159	1
DRAP1	0,03475824	6,716285059	0,99686372	1
TAP2	0,03392938	5,877618309	0,996904497	1
EXOC7	0,032757537	5,983972478	0,997053701	1
PNKP	0,041927024	6,531619179	0,997097869	1
ZC3H13	0,023813474	6,465010494	0,997179638	1
KLHL15	0,035625211	4,106150444	0,997683419	1
PSMC3	0,027011216	7,717530465	0,997793388	1
RPL3	0,036762069	10,51212427	0,99795368	1
COG7	0,048402208	4,242197518	0,998258773	1
ANKRD13C	0,030204836	5,573080262	0,998273351	1
AFF4	0,029220319	7,599014975	0,998777062	1
CAV2	0,114518364	8,821663615	0,999001937	1
KIAA0368	0,025687324	7,188123322	0,999193535	1
ZNF415	0,07884605	4,156633743	0,999841874	1
GXYLT2	0,06237945	5,332608159	1	1
NMI	0,060322765	4,117506507	1	1
PLEKHA7	0,058485147	4,435646298	1	1
TXNDC12	0,057154065	5,287840033	1	1
USP13	0,053846524	4,637216204	1	1
ANTXR2	0,052352868	6,588661793	1	1
TMEM161A	0,051437043	4,781510492	1	1
LYRM7	0,050448567	3,748209437	1	1
MPP5	0,049210138	5,569835842	1	1
TET1	0,047725823	4,020850116	1	1
ADCY7	0,045763725	5,715231747	1	1
RBM45	0,04181296	5,235016693	1	1
RSAD1	0,041542329	4,913595443	1	1
LETM1	0,041286238	4,906046116	1	1
IQSEC1	0,04109918	3,298323148	1	1
SEL1L	0,040432526	5,972404853	1	1
SCAMP1	0,040387485	5,107270067	1	1
C5orf44	0,039592621	4,878316494	1	1
SVIP	0,039055226	4,251437961	1	1
B9D1	0,03771462	4,508181521	1	1
ZNF211	0,037415796	4,379584374	1	1
OGFR	0,036719221	6,144860267	1	1
FAM63B	0,036130931	4,559749903	1	1
RXRB	0,035053941	5,269047191	1	1
PANK2	0,034193598	4,89990529	1	1
NXN	0,033940008	4,577160408	1	1

FZR1	0,033466852	5,513157064	1	1
PRKAR2A	0,033458766	4,69118053	1	1
ABCD4	0,033137769	5,210222695	1	1
DCTN3	0,032772249	5,28673496	1	1
MRPL22	0,032398487	4,958540825	1	1
GABPA	0,032040532	5,723739625	1	1
COG3	0,031001358	4,175522821	1	1
FAM53C	0,030468945	4,600870561	1	1
IFIT5	0,029502572	5,412109776	1	1
NAA35	0,029454757	5,205929069	1	1
CNOT6	0,029213907	5,828917163	1	1
UHRF1BP1L	0,028631834	4,593663153	1	1
C3orf17	0,028571469	4,88407757	1	1
LOC283070	0,028048303	5,200343156	1	1
COX15	0,027866776	4,770446076	1	1
IDI1	0,027554874	5,549781794	1	1
SH2D4A	0,026322988	5,574732255	1	1
PIP5K1A	0,026273191	6,066553485	1	1
SMAP1	0,025911238	5,394915786	1	1
ZDHHC5	0,025516607	5,625399069	1	1
HAUS4	0,024152372	3,931789079	1	1
NIT2	0,024034838	5,245931656	1	1
UBN1	0,023897377	5,190720124	1	1
SHPK	0,023245557	5,016715114	1	1
ZC3H3	0,023157583	5,063783566	1	1
CNST	0,022492662	3,305204023	1	1
SMYD2	0,021672104	4,807919682	1	1
RBM7	0,021646476	4,915005956	1	1
MLKL	0,020923141	5,25307844	1	1
TMEM54	0,020295275	4,518287489	1	1
TTC7B	0,019914548	4,742036087	1	1
LATS1	0,019541052	5,673701095	1	1
GOLGA2	0,019384078	4,137539731	1	1
DYNC1LI2	0,019381737	5,634975355	1	1
NDEL1	0,019326757	5,249890134	1	1
TNFAIP8	0,019284521	4,822824697	1	1
DYM	0,019139228	5,08755878	1	1
HEXDC	0,01899415	4,825520278	1	1
PPIP5K2	0,018625736	4,642758691	1	1
OTUD7B	0,017835216	4,539047	1	1
ATG12	0,016405245	5,3368861	1	1
C12orf65	0,01495513	5,078652906	1	1
NPRL2	0,014656324	3,784608453	1	1
SPATA2L	0,014536178	3,415016081	1	1
ENOPH1	0,014426637	5,735166111	1	1
DDHD1	0,014291862	5,227271347	1	1
KPNA1	0,014026366	4,895915618	1	1
KDM4C	0,013775266	5,206143321	1	1
RAB43	0,013073464	5,718341454	1	1
FAM118B	0,011787408	5,049498153	1	1

RCL1	0,011400698	5,232432386	1	1
NOL8	0,011324952	4,561202583	1	1
STK35	0,011004579	5,168272564	1	1
TFIP11	0,010800821	4,356836012	1	1
SH3GL1	0,009634479	5,445681291	1	1
KLC1	0,008465468	4,189365145	1	1
TRIM65	0,008130147	5,330631738	1	1
HIRA	0,008015796	4,005051523	1	1
TMEM170B	0,007268605	3,841097778	1	1
FZD8	0,007146301	5,604838615	1	1
MORC4	0,007047941	5,128901332	1	1
LYPLAL1	0,005975456	4,616808774	1	1
METRNL	0,004825951	5,218312897	1	1
C8orf40	0,004775431	5,659362781	1	1
TBC1D3	0,003963042	5,356896767	1	1
BRAF	0,003790601	5,739754216	1	1
FRYL	0,003624917	4,28274642	1	1
HDGFRP2	0,003495559	4,608582265	1	1
ATP5S	0,003363292	3,735783429	1	1
BAHCC1	0,003354196	4,785857477	1	1
PRKACB	0,002811702	4,947738475	1	1
CMC1	0,001845359	4,38023585	1	1
NARS2	-0,001362829	4,93115956	1	1
ZFP161	0,001077971	4,832125534	1	1
ZFAND3	0,000642689	5,483796493	1	1
TMEM135	0,000252069	5,760829382	1	1

Table S1_B: edgeR results

logFC, log fold change; logCPM, log counts per million; p value; FDR, Benjamini-Hochberg adjusted p value are reported for lung adenocarcinoma (LUAD) dataset

Gene	logFC	logCPM	PValue	FDR
EMP2	-2,889109475	9,840066224	4,82E-120	3,29E-116
STX11	-2,949125157	5,31838762	6,99E-112	2,38E-108
EPAS1	-2,883487185	10,17081942	1,61E-110	3,66E-107
GRK5	-2,788143567	5,714391585	2,78E-108	4,7412E-105
PYCR1	3,674599452	7,260126978	8,36901E-91	1,14137E-87
RASIP1	-2,472733358	4,665649265	5,83471E-83	6,63114E-80
AGER	-5,516499851	9,760823179	3,004E-82	2,92633E-79
PNPLA6	-1,637210823	7,004886314	1,2691E-81	1,08175E-78
GPR146	-2,264980936	3,671769476	5,21814E-76	3,95361E-73
ADRB2	-3,254426128	4,40227713	6,16001E-73	4,20051E-70
SMAD6	-2,719953429	4,605312915	4,82083E-66	2,98848E-63
HPS5	-1,191666515	5,790890531	1,08436E-62	6,16188E-60
SERINC1	-1,188808924	8,462782739	1,0717E-60	5,62147E-58
CAV2	-2,495264887	7,939299768	4,64293E-59	2,26144E-56
CSRNP1	-2,287581271	6,996382734	3,5023E-57	1,59214E-54
SNRK	-1,253414637	6,071643022	8,61485E-57	3,67154E-54
BMPR2	-1,396118546	7,632763753	4,72012E-56	1,89332E-53
ARHGAP31	-2,117518979	6,572130819	6,05697E-56	2,29458E-53
SPTBN1	-1,94734219	9,655093893	9,36764E-56	3,362E-53
AGTPBP1	-1,408443787	5,030129707	1,12729E-55	3,84349E-53
DOCK4	-1,810732306	5,802470341	2,55295E-55	8,28978E-53
TNS1	-2,358721343	9,198578765	8,26435E-55	2,56157E-52
CAT	-1,866044272	7,83310222	1,22826E-54	3,64153E-52
FEZ1	-1,805823381	4,220431702	1,40779E-54	3,99989E-52
SYNM	-2,162440403	4,932690383	2,23709E-54	6,1019E-52
TGFBR3	-2,650951904	5,62776107	3,87274E-54	1,0157E-51
NDST1	-1,716770863	7,59025682	8,09715E-54	2,04498E-51
THBD	-2,43529632	6,882739718	9,58316E-54	2,33384E-51
KIF1C	-1,568682362	7,862258201	8,53282E-53	2,00639E-50
TACC1	-1,958783669	7,852074673	1,12358E-52	2,55389E-50
HEG1	-2,221980354	7,536018365	3,34847E-52	7,36556E-50
MEF2A	-0,991481477	6,454219286	3,58045E-52	7,62971E-50
CD93	-2,156466038	7,785677226	4,76034E-52	9,8366E-50
RCAN1	-1,814563435	6,28820322	8,39287E-52	1,68326E-49
PPP1R15A	-1,882044104	7,50331745	1,30554E-51	2,54357E-49
GNAQ	-1,319797736	7,343737575	1,58452E-51	3,00134E-49
N4BP1	-1,12786104	6,793456077	1,95306E-51	3,59944E-49
TOP2A	4,013202065	7,279193709	2,20151E-51	3,95055E-49
ADARB1	-1,978617962	5,463985355	4,33781E-51	7,5845E-49
TMEM204	-1,8073939	4,81840444	6,10578E-51	1,04088E-48
KANK2	-1,637003626	7,417314654	1,12665E-49	1,87381E-47
C10orf54	-1,562239548	6,618679385	1,15452E-49	1,87445E-47
ALDH18A1	1,562731107	7,0408601	1,23972E-49	1,96596E-47
UBE2T	3,428222731	4,615489352	3,13531E-49	4,85902E-47
PLK1	3,570614258	4,991654691	5,75397E-49	8,71918E-47

ZFP106	-1,491718549	7,012625408	1,27596E-48	1,89147E-46
PTRF	-2,03086736	8,841421142	5,97089E-48	8,66287E-46
KIAA1462	-2,129559268	6,790992121	7,40553E-48	1,05205E-45
PPP1R14B	2,09152231	7,445777784	4,53124E-47	6,30583E-45
TBX2	-2,086076603	5,648277162	6,71105E-47	9,08657E-45
PAICS	1,771413065	7,374248082	6,79594E-47	9,08657E-45
CTHRC1	4,213672956	6,448063268	1,42402E-46	1,86739E-44
UHRF1	3,43299177	5,082710687	1,78479E-46	2,29632E-44
TOM1L2	-1,644475712	6,291992257	2,17184E-46	2,74255E-44
RCC1	1,777058335	5,320434975	2,28102E-46	2,82805E-44
SLC39A8	-2,401900512	8,5705656	3,1888E-46	3,88294E-44
ZWINT	2,640241076	5,192861351	4,07678E-46	4,87711E-44
MTMR10	-1,203643492	5,570006488	7,21409E-46	8,40513E-44
MDK	3,370294378	8,660452873	7,27237E-46	8,40513E-44
DLGAP5	3,80616867	4,357566156	7,92312E-46	9,00462E-44
GIMAP6	-1,95933531	5,841720116	1,17788E-45	1,31672E-43
GOLM1	2,471542641	8,42765101	1,21582E-45	1,33721E-43
AHNAK	-2,379898874	10,65230405	1,23838E-45	1,3404E-43
C20orf194	-1,52931068	5,219476796	1,73874E-45	1,85257E-43
GAB1	-1,552812015	5,453874501	2,02042E-45	2,11958E-43
CCNB1	3,264165109	5,815091473	2,95486E-45	3,05291E-43
SRPK1	1,522575962	6,811839799	3,11747E-45	3,17284E-43
QKI	-1,322431552	7,53689221	4,15578E-45	4,1674E-43
MYADM	-1,939501855	8,381225519	4,61766E-45	4,56345E-43
ATIC	1,214449798	6,93534445	6,32031E-45	6,15688E-43
SPARCL1	-2,032053296	8,566447123	6,6191E-45	6,35714E-43
SNX1	-0,987301061	7,466520078	1,11661E-44	1,05752E-42
NUSAP1	2,730852908	5,27362149	8,76306E-44	8,18566E-42
RRM2	3,101785762	6,187822325	5,41789E-43	4,99251E-41
KLF13	-1,582417847	7,445201354	8,20799E-43	7,4627E-41
KLF6	-1,732638942	8,552767976	6,60548E-42	5,92668E-40
LTBP4	-2,197193816	7,879459014	6,93146E-42	6,1384E-40
FLI1	-1,63285531	5,443582209	8,17609E-42	7,14779E-40
CFD	-2,394238226	5,898594749	8,829E-42	7,62088E-40
CALCOCO1	-1,429973558	6,973490181	1,77386E-41	1,51199E-39
TK1	2,899350392	6,253146507	2,09539E-41	1,76401E-39
GYPC	-1,559548299	5,341678145	6,08591E-41	5,06095E-39
LMCD1	-1,572154308	5,804510246	7,00753E-41	5,75715E-39
GINS2	3,094016376	3,930054591	1,12111E-40	8,99749E-39
SSFA2	-1,34880141	7,667587226	1,12155E-40	8,99749E-39
MMP11	5,814943216	7,176201911	1,72329E-40	1,36641E-38
C16orf88	1,234334884	4,563180284	3,09772E-40	2,42797E-38
MYBL2	4,414449899	5,976285934	3,78137E-40	2,93013E-38
ADAMTS1	-2,439599521	6,661666453	8,61859E-40	6,60339E-38
MYL9	-1,625060287	8,397525957	2,72466E-39	2,06438E-37
SLC2A1	3,732635286	7,795927749	2,76029E-39	2,0684E-37
GAS6	-1,776246308	7,740690196	6,50605E-39	4,82226E-37
TTK	3,457714705	3,805405721	6,96029E-39	5,10346E-37
FAM82A2	-0,962538824	6,379124496	1,26835E-38	9,20094E-37
ASPM	3,490965922	4,47167373	1,94928E-38	1,39918E-36

HBB	-3,676679294	8,386776209	2,03084E-38	1,44253E-36
CCNB2	3,585355242	4,801374501	2,08056E-38	1,46261E-36
CCNA2	3,116027273	4,800973451	2,37568E-38	1,65304E-36
AFAP1L1	-1,649298102	4,678349946	2,45406E-38	1,69033E-36
VAMP2	-1,269068585	6,811845654	5,0193E-38	3,42266E-36
UBE2C	4,346058078	5,657152524	5,37721E-38	3,63041E-36
CDCA5	3,071829412	4,760892919	5,55276E-38	3,69101E-36
VWF	-2,307291628	8,971279742	5,57521E-38	3,69101E-36
LATS2	-1,22037291	5,600529817	1,2654E-37	8,29686E-36
TRAP1	1,077162661	6,780314333	1,55985E-37	1,01301E-35
PDK4	-2,620900101	7,001380803	1,60938E-37	1,03532E-35
CCT3	1,346404808	8,689696095	1,73791E-37	1,10755E-35
INPP5K	-1,104414572	6,12260369	3,01951E-37	1,90649E-35
ZFYVE9	-1,463794567	5,192470627	3,86613E-37	2,41864E-35
SPAG5	3,04412519	5,231392349	7,37893E-37	4,57427E-35
LDLR	-2,142959171	8,092702356	9,22862E-37	5,66937E-35
SIGLEC11	-1,748460605	2,144648368	3,66206E-36	2,22961E-34
FXYD6	-1,686280707	5,941126487	4,26786E-36	2,57545E-34
RBMS2	-1,283581457	7,157809651	6,86924E-36	4,10889E-34
LMO7	-1,726187772	8,912881035	8,62205E-36	5,1125E-34
UTRN	-1,709013375	7,695827452	1,09516E-35	6,43785E-34
CRABP2	6,002566857	8,247507427	1,28667E-35	7,49899E-34
PAPSS1	-0,979787668	6,750406743	4,42696E-35	2,55826E-33
DLC1	-2,377576451	7,622753992	5,52079E-35	3,16355E-33
C2orf29	1,062855106	6,618577038	5,8224E-35	3,30858E-33
WDR12	1,31937237	4,878768038	5,90559E-35	3,32812E-33
HYAL2	-1,150792161	7,286701625	7,04618E-35	3,93835E-33
PTTG1	2,45522468	4,929526062	8,3556E-35	4,63226E-33
AMOTL1	-1,794437199	6,526308577	9,02875E-35	4,96509E-33
ITM2A	-1,885578726	5,544653589	1,48634E-34	8,10827E-33
OCIAD2	2,378132698	7,33913173	1,76273E-34	9,53973E-33
PDIA4	1,685177376	9,135020861	1,8301E-34	9,82633E-33
ARAP3	-1,43013308	5,06611238	3,95364E-34	2,10624E-32
PTPN1	-1,119863147	7,19573977	4,33029E-34	2,28901E-32
KPNA2	2,059888147	7,145273061	6,754E-34	3,54273E-32
CDK1	2,665305927	5,554782371	9,43784E-34	4,91272E-32
CSTF2	1,047429486	4,742248334	1,19941E-33	6,19607E-32
ENG	-1,493473282	8,182535129	1,30387E-33	6,68503E-32
BUB1	2,781832148	4,901433644	1,98344E-33	1,00934E-31
ZEB1	-1,465035081	5,39852205	2,90232E-33	1,46599E-31
FERMT2	-1,387781017	6,49955167	5,76591E-33	2,89101E-31
CALM1	-0,820595651	9,150389107	1,0234E-32	5,09384E-31
ARAP1	-0,955699027	7,662854634	1,17019E-32	5,78224E-31
LRRFIP1	-1,05461387	7,997396823	1,44509E-32	7,08926E-31
C1orf112	1,419326369	3,900695941	1,50658E-32	7,33811E-31
RECK	-1,4946187	4,463149073	1,92456E-32	9,30751E-31
LIMD1	-1,43280197	6,825633627	2,35936E-32	1,13299E-30
MCM4	2,126039834	6,430721964	2,56771E-32	1,22442E-30
INPP5A	-1,004273081	5,289993835	2,6532E-32	1,2564E-30
GGA2	-1,031188707	7,503744237	2,7461E-32	1,29142E-30

PTPLAD2	-1,477422287	5,300828446	3,60145E-32	1,68207E-30
ADD1	-0,787937494	8,301650724	4,59208E-32	2,13016E-30
MRPL3	1,115636091	6,992020059	6,83121E-32	3,14744E-30
HELLS	2,377637464	3,098722288	6,978E-32	3,19349E-30
PLSCR4	-1,41688624	5,479578486	1,12905E-31	5,13268E-30
MEIS1	-1,39312929	4,382340251	1,38981E-31	6,27625E-30
KIAA0101	2,291370377	4,551266618	1,60694E-31	7,20903E-30
MANF	1,320690928	6,710959088	1,68601E-31	7,51433E-30
BNIP2	-0,818788877	5,881278537	2,12988E-31	9,43093E-30
CRIM1	-1,532940344	7,219770349	2,75785E-31	1,21328E-29
PCYOX1	-1,17712017	7,913508348	2,78249E-31	1,21627E-29
TYMS	2,133146764	5,226405221	3,1408E-31	1,36415E-29
SPP1	5,379465066	9,933791872	3,84712E-31	1,66035E-29
STK39	1,538388681	6,328401311	6,27369E-31	2,69059E-29
HN1	1,806987273	7,679709277	6,45376E-31	2,75051E-29
ETF1	-0,744386548	7,605463544	8,04024E-31	3,40537E-29
DOK2	-1,75256918	5,185336523	9,00372E-31	3,7899E-29
MARCKSL1	2,039048371	7,930076842	1,02388E-30	4,28333E-29
SHROOM4	-1,965587338	5,704856655	1,10813E-30	4,60753E-29
MSTO1	1,222621456	5,616861837	1,43858E-30	5,94527E-29
CD52	-2,028801756	6,858946084	1,61205E-30	6,62202E-29
PTPRM	-1,653140489	6,547502898	2,43071E-30	9,88812E-29
MAP3K3	-1,091323735	6,02211836	2,43614E-30	9,88812E-29
MCM6	1,524157189	6,143385646	2,47263E-30	9,97684E-29
B4GALT2	1,219317644	6,210170578	2,7444E-30	1,09689E-28
CD83	-1,640145627	5,762956223	2,75066E-30	1,09689E-28
HIST1H2BD	2,472757545	4,575926846	3,7234E-30	1,47615E-28
ANKS1A	-1,244410065	6,536940396	5,01066E-30	1,97501E-28
MYO19	1,311777533	5,680316599	5,93128E-30	2,32445E-28
ARHGEF6	-1,368641331	5,660303726	6,98891E-30	2,72328E-28
SERPING1	-1,381545244	9,168837972	1,13762E-29	4,40765E-28
ALOX5	-1,766379072	6,772067667	1,21564E-29	4,68331E-28
HMGA1	2,399789254	8,451863366	1,25247E-29	4,7981E-28
DKC1	1,100885076	6,5924674	1,28269E-29	4,88641E-28
ZEB2	-1,464364789	6,250616586	1,31632E-29	4,98665E-28
C1orf198	-1,315413958	7,644814266	1,36917E-29	5,15821E-28
PION	-1,358248066	5,628825413	1,48949E-29	5,58067E-28
HMGB3	3,138901456	8,297550221	1,63528E-29	6,09342E-28
NPM3	1,935847794	5,025795255	2,60601E-29	9,65782E-28
EIF2AK1	0,912746718	8,271574924	2,70747E-29	9,9796E-28
GALNT7	1,64216387	6,659222824	2,81072E-29	1,03044E-27
CYBRD1	-1,794204296	8,26730543	2,93412E-29	1,06993E-27
APBB2	-1,2164039	6,005002702	3,37854E-29	1,22544E-27
FAM63B	-1,089086553	4,19671463	3,60895E-29	1,30209E-27
A2M	-1,849778583	11,25207895	4,21116E-29	1,51136E-27
NEXN	-1,642949929	4,646030646	4,5245E-29	1,61532E-27
FRMD4A	-1,331507949	5,128307784	6,30494E-29	2,23924E-27
NARF	1,150848791	6,371398742	6,52228E-29	2,30443E-27
BZW2	1,37377949	6,998193011	6,56654E-29	2,30668E-27
ACP5	-1,647199172	7,93440045	6,59631E-29	2,30668E-27

GAPDH	1,833372402	11,73532141	1,096E-28	3,81307E-27
SLC25A25	-1,535914022	5,771625653	1,12794E-28	3,90426E-27
TRAF4	1,284994415	7,044046414	1,36044E-28	4,68527E-27
FEN1	1,551036543	5,375222592	1,43979E-28	4,93362E-27
GORASP2	0,857128577	7,698112315	1,63344E-28	5,5692E-27
SORBS3	-1,130280006	6,92801601	1,73438E-28	5,88394E-27
RASL12	-1,508985345	4,611896317	2,00186E-28	6,75776E-27
SFXN4	1,412123643	5,032877002	2,75706E-28	9,26126E-27
FLAD1	1,339402602	6,106222569	2,98042E-28	9,96251E-27
ARHGEF10	-1,384524894	5,55425216	3,2939E-28	1,09566E-26
TNFRSF21	1,877426982	7,901068674	3,8273E-28	1,26691E-26
RPP40	1,655427553	3,015670006	5,43668E-28	1,79095E-26
PIK3R1	-1,228144843	6,414969224	5,73304E-28	1,8795E-26
SLC25A39	1,319715614	7,830955452	5,8291E-28	1,90185E-26
KLF4	-2,18445908	6,372909785	6,79278E-28	2,20571E-26
RDX	-1,058109102	7,457560669	6,83893E-28	2,20887E-26
P4HB	1,104568559	10,806187	6,86728E-28	2,20887E-26
OR2A9P	-1,252523565	3,917993994	1,06817E-27	3,41963E-26
ZBTB4	-1,13921034	7,449270788	1,13428E-27	3,61433E-26
DPH2	0,842837822	5,044261301	1,1776E-27	3,73491E-26
DNA2	1,913425264	3,14544201	1,27263E-27	4,01763E-26
FAP	2,724507599	5,315169342	1,37624E-27	4,3247E-26
HSPD1	1,210209521	8,913170377	1,53066E-27	4,78787E-26
FLCN	-0,819662191	5,620994365	1,75959E-27	5,47884E-26
SHMT2	1,703793	7,188582821	2,31747E-27	7,18312E-26
TFB2M	1,207790019	5,026046213	2,81304E-27	8,67968E-26
KCTD10	-0,719518482	6,862120237	2,98892E-27	9,18083E-26
SRD5A1	1,802568858	4,612669053	3,00633E-27	9,19289E-26
FBXO7	-0,638002019	7,108886759	3,14731E-27	9,58102E-26
TIMP1	1,834833973	9,358449808	3,44976E-27	1,04551E-25
JUND	-1,49072498	7,941076509	3,81289E-27	1,15045E-25
FAM136A	1,072444509	5,888107275	5,41619E-27	1,627E-25
PAK1	1,183225952	6,790162403	5,58193E-27	1,66944E-25
TJP1	-1,084068988	7,546784956	5,9756E-27	1,77937E-25
TIMP3	-1,865318445	10,12235826	6,3387E-27	1,87929E-25
ILF2	1,173137814	8,291506517	6,4667E-27	1,90894E-25
C10orf116	-2,59587748	6,518414345	6,98342E-27	2,05258E-25
DCAF13	1,308635185	6,133346066	1,02427E-26	2,99763E-25
SDCBP	-0,893115025	8,540845492	1,42892E-26	4,16401E-25
TNS3	-1,200274222	8,052084179	1,50095E-26	4,35531E-25
LDHA	1,463514604	10,24663065	1,54136E-26	4,45363E-25
FBXO32	2,652888252	5,247555667	2,10391E-26	6,05339E-25
HSD17B4	-1,059212564	7,607249685	2,28067E-26	6,53442E-25
RPGR	-1,347367129	3,983036924	2,40265E-26	6,8551E-25
ULK2	-1,069464521	5,29923448	2,44527E-26	6,94762E-25
GADD45B	-1,749455917	6,979258573	2,73569E-26	7,74053E-25
CHPF2	0,938766143	6,913112577	3,09467E-26	8,72007E-25
RUSC1	1,11528083	6,059955393	3,45708E-26	9,70116E-25
PROS1	-1,492980419	6,677926856	3,87561E-26	1,08311E-24
POC1A	1,683290734	3,648481492	4,15861E-26	1,15745E-24

MYH10	-1,666216244	7,657085894	4,76279E-26	1,32022E-24
DAB2IP	-1,235084954	6,574095138	4,9383E-26	1,36333E-24
SNRPE	1,178969132	6,424474982	5,46298E-26	1,5021E-24
FANCG	1,257469532	4,399054741	5,57951E-26	1,52798E-24
CCT5	1,262928247	8,460878236	6,23632E-26	1,70102E-24
CFL2	-1,324929028	5,858614976	6,42096E-26	1,7444E-24
ENO1	1,210290012	11,2433854	7,15965E-26	1,93737E-24
SLC44A2	-1,007251762	8,361581125	7,6628E-26	2,06532E-24
FABP5	-1,842866812	6,92428628	9,16932E-26	2,46164E-24
MRAS	-1,116460889	5,242131008	1,01903E-25	2,725E-24
EPB41L2	-1,397484483	6,401383258	1,04687E-25	2,78851E-24
TBC1D4	-1,205300189	5,407334974	1,13756E-25	3,01831E-24
KAT2B	-1,234678492	5,364721484	1,15925E-25	3,06392E-24
PDK1	1,683264957	5,247464455	1,75498E-25	4,62055E-24
PTGER4	-1,494918468	4,874423181	1,78305E-25	4,66628E-24
FHL2	2,489672849	6,450483624	1,78604E-25	4,66628E-24
INSIG1	-1,212546573	6,460634183	1,97675E-25	5,14482E-24
ERO1L	2,295588835	7,654601301	2,23885E-25	5,80483E-24
B4GALT3	1,049960407	6,438636183	2,59697E-25	6,70786E-24
PLEKHM3	-1,095800308	5,069444946	2,62758E-25	6,76131E-24
SACM1L	-0,836058804	6,289391795	2,92451E-25	7,49708E-24
BOP1	1,592243256	5,157719712	2,93602E-25	7,49839E-24
INTS8	0,971524299	5,722792753	2,97477E-25	7,56902E-24
FKBP11	1,807117105	6,097118024	3,28588E-25	8,32952E-24
IRAK1	1,162817815	7,69809149	3,58129E-25	9,04475E-24
CARD8	-0,910638912	5,529205507	3,89715E-25	9,80615E-24
GART	0,904636859	6,524497085	3,93234E-25	9,85832E-24
UBL3	-1,117177177	7,147585873	4,03068E-25	1,00678E-23
LMNB1	1,896796349	5,785989764	4,90606E-25	1,22096E-23
LAMB2	-1,156755749	8,120667045	5,29142E-25	1,31208E-23
ELMO1	-1,224769468	5,19429332	7,34174E-25	1,81389E-23
NLN	1,252832308	4,894591768	7,99373E-25	1,96784E-23
SSH2	-1,07161787	5,822023509	9,11472E-25	2,23573E-23
XPR1	1,663146311	7,55994515	9,70454E-25	2,37187E-23
ANO6	-0,921525049	7,242292544	1,12076E-24	2,72945E-23
ZNF438	-0,726135931	4,125441474	1,24877E-24	3,03038E-23
SERPINH1	1,315582901	8,535317022	1,27656E-24	3,08684E-23
CTNNAL1	-1,227914012	6,187142233	1,31569E-24	3,1702E-23
FLJ10038	-0,996961792	3,730822203	1,39581E-24	3,35141E-23
C11orf24	1,024575994	5,969156384	1,59426E-24	3,81449E-23
COL3A1	2,734312106	11,30438098	1,68479E-24	4,01698E-23
BRI3BP	1,496637279	3,085399961	1,71151E-24	4,06647E-23
PAFAH1B1	-0,719454022	7,610363582	1,96731E-24	4,65801E-23
RAB14	-0,659395707	7,738419053	2,34596E-24	5,53532E-23
NME4	1,495225099	6,790057078	2,45107E-24	5,7634E-23
VARS	1,16081905	7,283306755	2,56946E-24	6,02101E-23
TTC28	-1,40924216	5,488564092	2,84924E-24	6,65376E-23
CYP51A1	-1,055716278	7,378767207	3,00349E-24	6,99002E-23
MYO1B	-1,041358947	8,084710269	3,04897E-24	7,07174E-23
DARS2	1,208845837	5,465189002	3,074E-24	7,10564E-23

KNTC1	1,716089666	4,773907694	3,3954E-24	7,82203E-23
RNASEH2A	1,461764401	4,876225837	3,4829E-24	7,99659E-23
IQSEC1	-1,01897981	6,721414933	3,90076E-24	8,92593E-23
KCTD12	-1,335862388	7,912009434	4,03994E-24	9,21349E-23
NEDD9	-1,624524957	8,182286065	4,32542E-24	9,83169E-23
RAPGEF2	-1,052459982	6,048516411	4,69174E-24	1,06289E-22
C5orf34	1,854431383	2,115360274	4,9002E-24	1,10644E-22
MACF1	-1,376722661	8,78325002	5,1677E-24	1,16299E-22
SLC15A3	-1,372700108	6,404083109	5,76287E-24	1,29267E-22
MRC1	-2,146682782	8,029427893	6,08094E-24	1,35954E-22
PRDX4	1,641485015	7,524492782	6,11067E-24	1,36172E-22
FANCI	1,453735093	5,465946105	6,44126E-24	1,43072E-22
COL1A1	3,015883015	11,66685044	7,92118E-24	1,75372E-22
ZFP36	-1,819736262	8,844889303	8,37577E-24	1,84836E-22
CYTH1	-0,900167739	6,672862076	9,51064E-24	2,08911E-22
POLR2D	0,952602891	5,580838238	9,52798E-24	2,08911E-22
TARS2	0,95123392	5,449180579	9,61614E-24	2,10168E-22
FAM86A	0,817271245	4,285331614	1,01622E-23	2,21394E-22
RAB11FIP2	-0,9202306	5,000036375	1,03781E-23	2,25376E-22
EPB41L5	-1,259955883	6,08568888	1,05323E-23	2,27998E-22
STEAP1	3,015658373	5,085500957	1,07637E-23	2,32271E-22
VPS72	1,012958917	6,314431701	1,24662E-23	2,6816E-22
RC3H1	-0,744870961	5,609982074	1,29402E-23	2,77483E-22
PPFIBP1	-1,223479651	6,448244708	1,53669E-23	3,28486E-22
STIP1	0,820620156	7,880100694	1,61932E-23	3,45067E-22
CKS1B	2,036833694	6,551287014	1,66041E-23	3,5272E-22
ZNF25	-0,882931869	4,104038246	1,69194E-23	3,58301E-22
GGCT	1,483957639	6,281326244	1,71777E-23	3,62645E-22
UCHL5	0,856936123	5,359775051	1,74266E-23	3,66765E-22
FIGNL1	1,386719583	4,295621043	1,83289E-23	3,84568E-22
DENND5A	-0,855229467	6,707974206	1,86763E-23	3,90655E-22
SPTAN1	-1,076190912	8,728395802	2,27579E-23	4,74575E-22
HN1L	0,958738854	7,708260588	2,59227E-23	5,38922E-22
TCP11L2	-1,142537842	3,978818487	2,86935E-23	5,94714E-22
EXOSC5	1,424337236	4,833519926	2,95891E-23	6,11419E-22
ABTB1	-0,924648318	5,38060792	3,43397E-23	7,0744E-22
TPI1	1,295014802	9,76031217	3,47888E-23	7,14532E-22
CCDC69	-1,45314896	6,611286863	3,51208E-23	7,19185E-22
DTL	1,980574345	4,285765089	3,59526E-23	7,34014E-22
RACGAP1	1,701918862	5,664946364	3,90263E-23	7,94388E-22
N4BP2L1	-1,253583469	4,527943274	3,94569E-23	8,00763E-22
MYO7A	1,771821022	4,164567778	3,99451E-23	8,08266E-22
FAM13B	-1,003606004	5,342095636	4,90651E-23	9,89866E-22
PIAS1	-0,745451993	4,935206119	4,93739E-23	9,93159E-22
RALGPS2	1,199096274	5,024956147	6,09217E-23	1,22184E-21
NOL11	0,725854373	6,093882809	6,54805E-23	1,30942E-21
POLR2H	1,174466765	5,95637438	6,71664E-23	1,3392E-21
SAP30L	-0,862363527	5,621203819	8,80686E-23	1,75084E-21
PCNA	1,192751316	6,895653448	8,85696E-23	1,75569E-21
EHD2	-1,335169462	8,082075572	9,12863E-23	1,80429E-21

DDOST	0,87111687	8,851342395	9,18556E-23	1,8103E-21
SNAP47	0,667437234	5,548904253	1,03621E-22	2,03629E-21
WDR19	-0,995531818	4,730552112	1,15504E-22	2,26328E-21
LNX2	-1,093221997	6,065255267	1,25117E-22	2,44461E-21
EZH1	-0,889737647	5,515034352	1,31737E-22	2,56661E-21
KIAA0247	-0,931443956	7,230017491	1,57611E-22	3,06196E-21
LILRA6	-1,342293098	4,320694396	1,59792E-22	3,09551E-21
HMGCS1	-1,334965542	6,866019746	1,67471E-22	3,23508E-21
TMOD3	-0,775282899	7,193287439	1,87068E-22	3,60344E-21
DOCK11	-1,33055319	5,572027607	2,09574E-22	4,02559E-21
TMEM109	-0,676786032	7,558729724	2,16977E-22	4,15608E-21
TBRG4	1,031945257	6,232788964	2,20478E-22	4,21132E-21
DNAJA3	0,775213984	6,275201388	2,27398E-22	4,33135E-21
RFC4	1,720725825	4,537472774	2,33401E-22	4,43331E-21
ACTL6A	1,091266071	6,381495848	2,3997E-22	4,54544E-21
TRAK2	-0,8543475	6,678197442	2,42258E-22	4,57606E-21
AIMP2	1,119358271	5,157873736	2,47152E-22	4,6556E-21
GPI	1,199525501	8,782525089	2,52538E-22	4,74396E-21
RAB11FIP1	-1,507370862	8,934613432	2,8736E-22	5,38326E-21
HSPA5	0,796544694	10,29359791	3,09041E-22	5,77357E-21
EPCAM	1,569149819	9,245991983	3,35907E-22	6,25833E-21
APBB1	-1,36107851	5,370265872	3,50606E-22	6,5144E-21
ARRB2	-0,908781009	6,494778224	3,89715E-22	7,22138E-21
HSPE1	1,364585581	7,255586789	4,0669E-22	7,51549E-21
C15orf48	3,523812108	6,635648909	4,12402E-22	7,60046E-21
IL7R	-1,957979871	5,25303607	4,21913E-22	7,75479E-21
PPIF	1,155603634	6,33555918	4,80427E-22	8,80654E-21
HECA	-0,982833088	5,958817104	4,95316E-22	9,05512E-21
NCAPG2	1,661011936	5,147510837	4,97153E-22	9,0644E-21
DBF4	1,444745138	4,410121775	5,18498E-22	9,42836E-21
AVL9	1,165595461	5,046309938	5,213E-22	9,45411E-21
KIAA1524	2,053982538	3,616918031	5,30911E-22	9,58135E-21
PMM2	0,965589978	6,230396856	5,31126E-22	9,58135E-21
PFKP	2,092428891	7,598954721	5,43366E-22	9,77629E-21
SORD	1,435064272	6,024216317	5,66462E-22	1,0165E-20
SMAD7	-1,151555773	5,638706063	6,41623E-22	1,14835E-20
UBE2S	2,052807386	5,818527379	6,83498E-22	1,2201E-20
INO80	-0,77120473	5,735949158	8,1868E-22	1,45759E-20
C1orf131	0,843647447	4,0930042	8,32795E-22	1,47886E-20
E2F3	1,260304269	5,1903632	9,16548E-22	1,62336E-20
NUP155	1,352754888	5,460030758	9,73892E-22	1,72046E-20
SNX2	-0,72586641	6,838742809	1,00773E-21	1,77564E-20
OLA1	1,032554701	7,030139205	1,0364E-21	1,82144E-20
MRPL12	1,355411245	5,854002933	1,20821E-21	2,11795E-20
KDELR2	0,995855782	8,936274974	1,32323E-21	2,31361E-20
SRM	1,085646764	6,28064107	1,33049E-21	2,32036E-20
BPHL	1,311800874	4,956454903	1,36643E-21	2,37696E-20
SLC35B1	0,81294059	6,445300815	1,38062E-21	2,39554E-20
IL18R1	-1,668920506	3,198960849	1,4538E-21	2,51611E-20
COL5A2	2,293662917	7,872467245	1,54084E-21	2,66E-20

FAM53B	-0,986280435	5,88070227	1,55577E-21	2,67898E-20
FIBIN	-1,920422811	4,606979135	1,6566E-21	2,84543E-20
TUBG1	1,220869522	5,923839663	1,73518E-21	2,96585E-20
PLEKHA1	-0,903960797	6,508204497	1,7354E-21	2,96585E-20
KLF2	-1,612583525	5,586847443	1,79527E-21	3,06048E-20
DTYMK	1,431222563	5,385677909	1,85447E-21	3,15352E-20
IDH2	1,197241349	7,379539054	2,12884E-21	3,61108E-20
SEC22C	-0,692423058	5,319729301	2,36275E-21	3,99792E-20
SIK2	-1,040690538	5,927620546	2,62865E-21	4,43041E-20
NAT9	0,916924168	5,383485089	2,63135E-21	4,43041E-20
UBFD1	0,798650969	6,509971773	3,0792E-21	5,1717E-20
SEPT10	-0,956204801	6,887247979	3,12901E-21	5,24243E-20
ARRDC4	-1,22693711	5,323356099	3,36898E-21	5,63065E-20
FAM122A	-0,639094044	4,789663719	3,54706E-21	5,9138E-20
BRIX1	1,184529691	5,896514018	3,69636E-21	6,14767E-20
FBXW2	-0,726491751	6,694166104	3,75534E-21	6,23057E-20
SRPX2	2,719101597	5,344343991	3,88604E-21	6,43177E-20
DAP3	0,952644862	7,270567427	3,90866E-21	6,45354E-20
HLA-E	-1,059917443	10,16923658	3,9756E-21	6,54822E-20
MFAP2	2,009208126	5,726085836	4,03918E-21	6,6369E-20
HERC1	-1,053448223	6,279453687	4,09764E-21	6,71679E-20
DUSP8	-1,270352393	4,703018424	4,19931E-21	6,86692E-20
ADAM8	2,048576816	6,142698751	4,32125E-21	7,04943E-20
KLF10	-1,110039583	7,077802358	4,72153E-21	7,68403E-20
SGPL1	0,960900585	7,222845877	4,84569E-21	7,86732E-20
SGOL2	1,517788279	3,693465509	5,01816E-21	8,12798E-20
DST	-1,519779307	7,646868982	5,10848E-21	8,25468E-20
DHCR24	-1,188875103	10,08006737	5,54474E-21	8,93843E-20
TMEM69	0,78483054	5,132266568	5,7462E-21	9,24127E-20
EMP1	-1,682816849	8,257839012	5,7597E-21	9,24127E-20
TBC1D9	-0,932375891	6,269958457	6,1537E-21	9,85026E-20
MTHFD2	1,715117109	6,808683866	6,28108E-21	1,00306E-19
PIK3R5	-1,357373002	4,644324998	6,44141E-21	1,02626E-19
ARHGAP18	-1,046811449	6,574979855	6,76863E-21	1,07588E-19
ZNF331	-1,248827427	5,535945367	6,83796E-21	1,08437E-19
AHCY	1,150231513	7,951281919	7,21428E-21	1,1414E-19
SEMA4B	1,485503396	7,818689851	7,36628E-21	1,16275E-19
CYR61	-1,589920776	8,47329219	8,53278E-21	1,34376E-19
RXRA	-0,879151222	6,952595871	8,86936E-21	1,39355E-19
LSM12	0,764800931	6,601873713	9,47092E-21	1,48465E-19
METTL1	1,54232431	5,06788341	1,03074E-20	1,61207E-19
MTFR1	1,332693844	5,645757743	1,05854E-20	1,65176E-19
GNL3	0,946980105	6,700925473	1,07113E-20	1,66759E-19
NCF2	-1,495668753	6,540743049	1,10726E-20	1,71991E-19
ABCC3	2,298188993	8,000180294	1,13859E-20	1,76455E-19
LRRC59	0,924456009	8,1418153	1,25167E-20	1,93541E-19
MRPL9	1,025723376	6,320440083	1,3171E-20	2,03185E-19
PDZD11	1,083233878	5,861778318	1,32E-20	2,03185E-19
CTGF	-1,525062632	8,55174032	1,45127E-20	2,22887E-19
TMEM110	-0,707769309	3,889541993	1,64351E-20	2,51845E-19

PARD6B	-1,126598889	5,250086519	1,70148E-20	2,60143E-19
TMEM48	1,079240422	4,491609598	1,75686E-20	2,68009E-19
CBX3	1,037002854	8,102219278	1,82663E-20	2,78032E-19
GNPNAT1	1,506136104	6,44895399	1,85238E-20	2,81322E-19
BUB3	0,755966883	6,879257573	1,96359E-20	2,97549E-19
GPR89A	0,890094943	4,774603473	2,0749E-20	3,1372E-19
SYNJ2	1,337034803	4,829768227	2,23961E-20	3,37873E-19
PSMD11	0,958750735	6,731041588	2,36136E-20	3,55455E-19
EGLN3	2,85499268	6,064862479	2,7593E-20	4,14441E-19
MYO9A	-1,148798777	4,85715899	3,07197E-20	4,6039E-19
ARRB1	-1,193259087	6,392425795	3,10664E-20	4,64565E-19
TXNDC17	1,783541761	6,801619414	3,14911E-20	4,69886E-19
DNAJC9	0,931191916	5,571517662	3,274E-20	4,87454E-19
SYNE1	-1,596783421	7,416245695	3,32062E-20	4,93319E-19
CCDC50	-0,970889024	6,564472594	3,38339E-20	5,0155E-19
DAPK1	-1,403363604	7,199864335	3,441E-20	5,08985E-19
STOM	-1,157311626	9,043479007	3,44915E-20	5,09086E-19
NUMB	-0,651916759	7,026558561	3,54691E-20	5,22383E-19
SH3BGRL	-0,838993574	7,56700506	3,71138E-20	5,4543E-19
LONRF1	-1,047306709	4,725458505	3,77338E-20	5,53348E-19
CNP	0,862316963	7,220910869	3,93195E-20	5,75364E-19
SUN2	-0,82726683	7,764950275	4,30796E-20	6,29035E-19
APC	-0,944541854	5,454320467	4,72433E-20	6,88359E-19
IPO4	0,854795683	6,438210898	4,74363E-20	6,89698E-19
ATAD3A	1,057758249	5,478335416	5,0287E-20	7,2959E-19
H2AFX	1,288120763	5,855897542	5,07871E-20	7,35281E-19
NIPSNAP1	1,145134324	6,760266708	5,11076E-20	7,38354E-19
LRRC8C	-1,141815639	5,172787874	5,19131E-20	7,48405E-19
HDGF	0,972316412	8,99451107	5,2568E-20	7,56248E-19
NEK7	-0,925483338	7,136613158	5,71025E-20	8,19751E-19
NR4A3	-2,290464171	5,126875008	5,74197E-20	8,22574E-19
WDR75	0,654452381	5,611477263	5,9362E-20	8,48615E-19
SRPRB	1,064495457	6,803131196	6,01312E-20	8,57812E-19
ARHGAP11A	1,586580807	4,680560382	6,25018E-20	8,8977E-19
SSR4	1,560716964	8,819545574	6,33949E-20	9,00603E-19
GORASP1	-0,572375505	5,957165542	6,74639E-20	9,56416E-19
MEST	1,571700012	6,712062178	7,34729E-20	1,03944E-18
PRMT3	0,850456418	4,47185454	7,42869E-20	1,04878E-18
DDX56	0,803624821	6,608556341	8,05015E-20	1,13417E-18
THOC3	1,084646706	5,302508069	1,0076E-19	1,41667E-18
SLCO2A1	-1,786361384	7,133923719	1,02627E-19	1,43994E-18
PFDN6	1,066433842	5,484844217	1,07793E-19	1,50932E-18
SMARCA2	-1,088377915	7,006835323	1,35482E-19	1,89314E-18
B3GNT2	-0,851552842	6,14306087	1,36417E-19	1,90231E-18
SRD5A3	1,776102109	6,690415618	1,48595E-19	2,06789E-18
PPM1D	-0,813549166	4,620379367	1,54481E-19	2,14542E-18
HPCAL1	-1,07485929	7,154712238	1,55028E-19	2,14865E-18
RANBP10	-0,957293196	5,075515152	1,61857E-19	2,23874E-18
CALU	1,099189873	8,559851788	1,71115E-19	2,36201E-18
PLAU	2,751847334	7,941011098	1,83048E-19	2,52162E-18

SH3D19	-0,871434461	6,80548071	2,12871E-19	2,92655E-18
FADD	0,837265001	5,478733904	2,30399E-19	3,16115E-18
PDIA6	1,122172159	9,19746739	2,39817E-19	3,28376E-18
EGR2	-1,57552238	4,875046656	2,42726E-19	3,31065E-18
ETV5	-1,19827609	6,551318339	2,42752E-19	3,31065E-18
COL5A1	2,198338248	8,548703073	2,55579E-19	3,47863E-18
XPO5	0,953956251	6,190252488	2,61583E-19	3,55325E-18
ITM2C	1,437621501	7,646530877	2,81444E-19	3,81544E-18
RABIF	0,740770907	4,870036147	2,95369E-19	3,99627E-18
CBX4	0,95912277	5,636723202	3,03332E-19	4,09588E-18
NEDD4L	-1,291149983	7,574786285	3,27513E-19	4,41366E-18
YWHAZ	0,856719186	10,57730839	3,38807E-19	4,55685E-18
SULF1	2,425710796	7,619708575	3,47145E-19	4,6598E-18
ABI2	-0,719312255	6,328330437	3,60695E-19	4,83219E-18
NUP85	0,771510046	5,704255571	3,72614E-19	4,98207E-18
SLC7A7	-1,254774457	6,214125838	3,73959E-19	4,99027E-18
TMED3	1,354105554	7,625874368	3,78276E-19	5,03802E-18
HK3	-1,733646469	5,034909984	3,91932E-19	5,20972E-18
GATA6	-1,817263486	5,029276333	4,24468E-19	5,63122E-18
FAM188B	-1,076378917	4,412672889	4,29236E-19	5,68342E-18
STON1	-1,21336475	4,282476823	4,32631E-19	5,71727E-18
UACA	-1,091185024	6,086458385	4,70051E-19	6,19976E-18
MRPL37	0,750476327	6,464814697	4,97357E-19	6,54725E-18
PYGO2	0,802719742	6,62998648	5,01051E-19	6,5751E-18
CALR	0,754476314	10,653457	5,01401E-19	6,5751E-18
DOLPP1	0,835814317	4,898423796	5,75829E-19	7,53662E-18
PMP22	-1,068003282	7,101750527	5,99896E-19	7,83657E-18
AKAP11	-0,79199527	6,372788152	6,36728E-19	8,30181E-18
NQO1	2,936907765	8,629249711	6,40933E-19	8,3407E-18
SNRPC	0,926795062	6,748926089	6,77567E-19	8,80062E-18
SPATS2	0,905397748	5,64884657	7,12702E-19	9,23938E-18
USP12	-0,957627959	4,732143181	7,25014E-19	9,38116E-18
DUS1L	0,967479239	6,791102576	7,37242E-19	9,52131E-18
CYGB	-1,091497587	4,801647507	7,39018E-19	9,5262E-18
ARHGEF3	-0,826229381	6,029705603	7,77651E-19	1,00053E-17
CHPF	1,160734438	7,43554274	8,04041E-19	1,03253E-17
ASNS	1,590315251	5,603350196	8,16384E-19	1,04641E-17
SRF	-0,774259654	6,455559097	9,77729E-19	1,25087E-17
MC1R	1,770670747	3,087855296	9,94819E-19	1,27035E-17
NFIX	-1,536113792	7,227912697	1,08669E-18	1,38508E-17
C17orf62	0,749601108	6,98642984	1,18389E-18	1,50615E-17
NFYB	-0,685694954	5,52802243	1,19274E-18	1,51458E-17
TJP2	-1,013628628	7,057167609	1,21579E-18	1,54098E-17
SEC14L1	-1,077375671	7,349957354	1,31905E-18	1,66876E-17
PPP2CB	-0,773925383	7,289722893	1,33559E-18	1,68656E-17
TRIM25	-0,866647789	6,118817636	1,33981E-18	1,68876E-17
EVL	-1,010485321	6,696119986	1,54215E-18	1,9367E-17
NSUN5	0,913782862	5,380214088	1,5422E-18	1,9367E-17
PGAM5	1,070566342	5,259459662	1,61748E-18	2,02749E-17
NOL10	0,628744712	5,453768909	1,73817E-18	2,17478E-17

TSTA3	1,369148573	7,089459712	1,74942E-18	2,18485E-17
CENPH	1,274908132	3,577318193	1,78629E-18	2,22682E-17
MTX2	0,816590213	5,680627039	1,89506E-18	2,35811E-17
TIMM17A	0,841410802	6,367621417	1,90445E-18	2,36548E-17
STAT5B	-0,658651884	6,588444456	2,04882E-18	2,53985E-17
MPP6	1,46045644	2,78301899	2,05229E-18	2,53985E-17
TXNDC5	0,955938816	9,110329514	2,08856E-18	2,58006E-17
R3HDM1	0,784969009	5,326088499	2,14977E-18	2,65087E-17
TSPYL1	-0,795150027	6,979253179	2,17895E-18	2,68199E-17
ALDH2	-1,26190045	8,929940913	2,24552E-18	2,75895E-17
CNPY2	1,082194908	6,92813442	2,2695E-18	2,7834E-17
PABPC1	1,011928325	10,9126224	2,4317E-18	2,97698E-17
SLC25A13	0,970402366	5,465896308	2,57273E-18	3,14399E-17
DECR2	1,180775641	4,795788858	2,6029E-18	3,17516E-17
FAM105A	-1,569590208	6,291203387	2,63341E-18	3,20665E-17
APEX1	0,800571036	7,591564527	2,751E-18	3,34386E-17
MRPL15	1,240174002	6,262638201	2,75977E-18	3,34856E-17
BCL2L12	0,958643279	4,447975643	2,84656E-18	3,44772E-17
TLR4	-1,381399441	5,582005042	2,94034E-18	3,55499E-17
HEATR1	0,746407455	6,125202164	2,99428E-18	3,61381E-17
HMOX1	-1,836962194	7,25563733	3,27412E-18	3,94456E-17
PREB	0,795601631	6,528275084	3,31573E-18	3,98765E-17
CLDN12	1,078531642	6,551768035	3,47685E-18	4,17406E-17
FBXL3	-0,733160324	6,379989397	3,64112E-18	4,36359E-17
LOC728554	1,14648401	4,765747383	3,67421E-18	4,39463E-17
ZCCHC24	-0,998907293	5,664946909	3,67991E-18	4,39463E-17
SBDS	-0,774098213	7,125274268	3,75106E-18	4,47177E-17
ATXN1L	-0,817624505	6,180377752	3,80945E-18	4,53344E-17
H1F0	1,142444073	8,667118974	4,06085E-18	4,82421E-17
WBP4	-0,618980367	4,392364159	4,17561E-18	4,95191E-17
RNH1	-0,618216642	7,967308537	4,26128E-18	5,04473E-17
ADCY9	-1,279314121	6,306103837	4,32276E-18	5,10864E-17
HPS3	1,651624006	7,028982943	4,50548E-18	5,31538E-17
LAGE3	1,314486551	5,067020373	4,89903E-18	5,76968E-17
C10orf10	-1,506783811	7,870153448	4,95343E-18	5,8237E-17
SNRPA1	1,029499402	5,429857969	5,49658E-18	6,45115E-17
BAK1	1,038017016	6,079916271	5,53581E-18	6,48603E-17
PCF11	-0,742017071	5,972828727	5,68665E-18	6,65133E-17
MAP4	-0,793874056	8,468444903	5,92467E-18	6,91787E-17
C12orf45	1,06337587	3,786877843	6,59892E-18	7,69197E-17
MTX1	1,002020726	5,84310427	6,7302E-18	7,83161E-17
PUS7L	1,018186832	3,53398666	7,36348E-18	8,55393E-17
ARNTL2	2,693357825	4,114456445	7,70789E-18	8,9388E-17
ATXN3	-0,565365185	5,065207106	7,98321E-18	9,24236E-17
MRTO4	0,98749968	6,119476366	8,18907E-18	9,46462E-17
MRPS30	0,875039831	5,059998606	8,5689E-18	9,88686E-17
EIF6	0,864858415	7,606912584	8,73345E-18	1,00597E-16
ALG8	0,887884928	5,680099395	8,77075E-18	1,00856E-16
TARS	0,959091509	7,082118592	9,12777E-18	1,04646E-16
LZTFL1	-0,829524547	5,246183288	9,13097E-18	1,04646E-16

DOCK6	-0,909859436	6,12038457	9,243E-18	1,05752E-16
KIAA0355	-0,778856809	5,801136064	9,66993E-18	1,10451E-16
MXD4	-0,759342109	6,853316674	9,79149E-18	1,11652E-16
TWF1	1,052208942	7,512483634	1,02345E-17	1,16509E-16
ABHD5	-0,764088248	5,706850971	1,23186E-17	1,4E-16
SNRNP25	1,045847101	5,342053755	1,25437E-17	1,42322E-16
MPHOSPH8	-0,735528718	5,873897336	1,39294E-17	1,57782E-16
UTP6	0,621486956	5,898097436	1,46823E-17	1,66034E-16
HOXA5	-1,545477711	3,01334238	1,47875E-17	1,66948E-16
RPE	0,816548293	5,711953004	1,55358E-17	1,75105E-16
CCDC86	0,954590875	5,194336377	1,5772E-17	1,77474E-16
CYP27A1	-1,517913734	7,19247513	1,65634E-17	1,86072E-16
GPR89C	0,844827226	3,207003826	1,68448E-17	1,88922E-16
C1orf85	0,933643891	6,857898667	1,72786E-17	1,93469E-16
SREBF2	-0,901252652	8,01341144	1,73909E-17	1,94407E-16
TDRD3	-0,821278646	4,491657782	1,75656E-17	1,96039E-16
ANKRD40	-0,566897522	6,714532036	1,78255E-17	1,98614E-16
MYO1F	-1,163377636	5,770119958	1,79495E-17	1,9967E-16
CD68	-1,226036755	9,053525608	1,84767E-17	2,05199E-16
GARS	1,009434438	7,412589421	2,00036E-17	2,21579E-16
DDX5	-0,598375637	9,59221014	2,00165E-17	2,21579E-16
TTYH3	1,449468387	7,510084725	2,01534E-17	2,22733E-16
PSMD12	0,739021168	6,032075182	2,05802E-17	2,27082E-16
INCENP	1,192899451	4,774797352	2,08547E-17	2,29738E-16
PLOD2	2,175532302	7,374701294	2,13584E-17	2,34908E-16
FTO	-0,799436137	6,229937706	2,16604E-17	2,37846E-16
MARS2	0,907038415	3,728138915	2,19496E-17	2,40634E-16
HNRNPC	0,672510507	9,473384224	2,30605E-17	2,52407E-16
PSMB4	1,010471024	8,387841873	2,42433E-17	2,64928E-16
DENND4A	-0,856810573	5,051211632	2,51296E-17	2,74174E-16
RAB34	0,959364142	7,066822758	2,55063E-17	2,77839E-16
PLEKHA8	0,972696471	3,189951826	2,80556E-17	3,05122E-16
ERAL1	0,621782331	6,131811995	3,07716E-17	3,34126E-16
EPRS	0,748316217	7,747202706	3,23266E-17	3,50453E-16
COLEC12	-1,600046723	6,447335772	3,36748E-17	3,6449E-16
CWF19L2	-0,636018712	4,5660344	3,4405E-17	3,71803E-16
STX7	-0,718024866	6,600819295	3,51705E-17	3,79475E-16
COASY	0,757604218	6,716728947	3,76722E-17	4,05824E-16
NFKBIZ	-1,53019549	6,26020092	3,94485E-17	4,2429E-16
PDCD6	1,106588687	7,311279671	4,02106E-17	4,31805E-16
CRYAB	-1,730970451	4,517816193	4,03319E-17	4,32426E-16
HIST1H4H	2,043745294	2,886657632	4,22451E-17	4,52229E-16
KIF18A	1,368395665	3,271366811	4,44035E-17	4,74588E-16
UBASH3B	-1,356948973	4,937306845	4,47985E-17	4,78061E-16
DOT1L	-0,979409759	5,575090997	4,51205E-17	4,80744E-16
ZMYND19	0,910517852	4,447127404	4,69327E-17	4,99273E-16
ZNF207	0,479997248	7,712086219	4,77874E-17	5,07574E-16
CACYBP	0,899501359	6,97803488	4,81021E-17	5,10121E-16
PPM1G	0,658269667	7,547372054	4,86116E-17	5,14725E-16
NDRG2	-1,365575116	6,344534338	4,89727E-17	5,17744E-16

VASH1	-1,059801369	5,780317974	5,35088E-17	5,64825E-16
PDCD6IP	-0,546045244	7,755213086	5,40347E-17	5,69494E-16
IARS2	0,70760506	7,579221062	5,53399E-17	5,8235E-16
NDEL1	-0,669041106	5,95710945	5,54989E-17	5,83124E-16
PTGES2	0,783039753	5,676710195	5,64015E-17	5,91695E-16
RMI1	0,869811194	4,329467184	5,68066E-17	5,95029E-16
MFSD2A	-1,630765084	6,524430265	5,75247E-17	6,01628E-16
TSPAN6	1,230051074	6,541352983	5,82758E-17	6,0855E-16
PITPNA	-0,568459222	6,998145427	5,99841E-17	6,2543E-16
PFKFB2	-1,072361903	6,165866779	6,13709E-17	6,38913E-16
HPRT1	1,132305137	5,720183714	6,16157E-17	6,40484E-16
PALB2	0,661688935	4,360563025	6,17844E-17	6,4126E-16
TAOK2	-0,747607661	6,619363112	6,24436E-17	6,47117E-16
ITGAL	-1,293853409	6,094967604	6,27287E-17	6,49085E-16
GFPT1	0,969017739	7,911790515	6,40574E-17	6,61829E-16
EIF2B2	0,653797126	5,313771297	6,46752E-17	6,67202E-16
HDAC1	0,772555876	7,404962926	6,96022E-17	7,16945E-16
LRRK2	-2,265646001	8,012926648	7,28535E-17	7,49304E-16
TNFAIP1	-0,620752657	6,959349581	7,34369E-17	7,54166E-16
PREX1	-1,193913977	6,539588036	7,3921E-17	7,57996E-16
PLEKHO2	-0,994505217	6,627396045	7,69297E-17	7,87663E-16
DHFR	1,008812639	5,907091911	7,78075E-17	7,95457E-16
CKAP2	1,24855219	5,24390654	7,86172E-17	8,02531E-16
FANCF	0,748214199	4,777714357	7,9404E-17	8,09351E-16
LIMK1	1,142835184	6,898696081	8,20319E-17	8,34889E-16
INTS7	0,731233161	5,361799804	8,28821E-17	8,42285E-16
MRPL24	1,140907704	6,365992053	9,34646E-17	9,48416E-16
CHTF18	1,281612034	3,866946199	9,54085E-17	9,66702E-16
IL4I1	1,690630132	4,809229298	9,88422E-17	1,00001E-15
MSI2	1,068656089	4,668825633	1,05356E-16	1,06433E-15
PSMD14	0,96939097	6,846000517	1,06491E-16	1,0742E-15
DPP3	0,807539101	6,434577304	1,07083E-16	1,07858E-15
PSMD4	0,829047186	7,739966601	1,16015E-16	1,16682E-15
IGFBP3	2,32817109	8,798873421	1,19997E-16	1,20509E-15
KANK1	-0,934443397	5,835402023	1,23349E-16	1,23694E-15
EPS15	-0,475930906	6,818708906	1,24838E-16	1,25003E-15
DARS	0,630151851	7,02953206	1,31973E-16	1,31954E-15
PARP1	0,919287309	7,955441192	1,4225E-16	1,42021E-15
SMUG1	1,019573794	5,44835047	1,4296E-16	1,42521E-15
LAPTM4B	1,37019551	8,736042281	1,51483E-16	1,50798E-15
SLC6A8	1,899495873	5,775613339	1,586E-16	1,57652E-15
CSRP1	-0,803055458	8,750557354	1,749E-16	1,73537E-15
MRPL36	1,234513132	5,110203466	1,7509E-16	1,73537E-15
KRTCAP2	1,106839687	7,323735356	1,80684E-16	1,78822E-15
UNG	0,93053276	5,796433148	1,82618E-16	1,80474E-15
DAP	1,055034833	8,41483266	1,8713E-16	1,84665E-15
ITGAX	-1,338939354	6,161111726	1,87832E-16	1,8509E-15
GMPS	0,907251354	6,31911818	1,88267E-16	1,85251E-15
FKBP10	2,15339372	7,505452276	1,88821E-16	1,85529E-15
SNX25	-1,310906645	6,089654362	1,96492E-16	1,92789E-15

CIRBP	-0,864711815	8,084651326	2,21962E-16	2,17316E-15
STT3A	0,736373823	7,887112286	2,22128E-16	2,17316E-15
RBMS1	-0,663353022	7,085721124	2,27045E-16	2,21808E-15
SIRPA	-1,092489911	6,897753358	2,3218E-16	2,265E-15
RPP21	0,944543683	4,379434786	2,32572E-16	2,26559E-15
RAB8B	-0,888447466	5,965142395	2,45083E-16	2,38406E-15
ZMYND11	-0,622988633	6,910227494	2,47066E-16	2,39992E-15
CLNS1A	0,781717293	6,610768808	2,48817E-16	2,41349E-15
IARS	0,690672194	7,342674323	2,51889E-16	2,43982E-15
IMP4	0,73930477	5,720647549	2,62198E-16	2,53607E-15
SUSD2	-2,301762921	8,700268769	2,64715E-16	2,55679E-15
UQCRHL	1,122002105	3,95802517	2,69798E-16	2,6022E-15
RCC2	0,965365578	7,762301609	2,78117E-16	2,67865E-15
MICAL1	-0,865352825	5,801609353	2,79011E-16	2,68347E-15
NOB1	0,802663188	6,173909507	2,84193E-16	2,72945E-15
SESTD1	-0,925326276	6,347680894	2,87448E-16	2,75683E-15
USP54	-1,018079691	6,637777101	2,88787E-16	2,76578E-15
EIF2C4	-0,774012239	4,833180756	2,89779E-16	2,77139E-15
DCAF12	0,877362411	7,105297432	2,98354E-16	2,8494E-15
CKAP4	1,086127791	7,594415256	3,13741E-16	2,99217E-15
EFEMP1	-1,459042614	7,979662341	3,17653E-16	3,02525E-15
TRIM27	0,650418321	6,726257218	3,18548E-16	3,02954E-15
NR2C2AP	0,952921577	4,715281129	3,23584E-16	3,07315E-15
TMEM164	-0,982026956	6,127273733	3,44048E-16	3,26296E-15
EARS2	0,696442397	5,321756392	3,45501E-16	3,27218E-15
RANBP1	0,96740734	6,396935028	3,69043E-16	3,4903E-15
SMYD4	-0,582010145	4,42948223	3,75267E-16	3,54424E-15
THY1	1,786008742	7,107865723	4,05779E-16	3,82712E-15
WDR76	0,961777554	3,890828494	4,2161E-16	3,97094E-15
CARD16	-1,226618348	4,340210781	4,37143E-16	4,11155E-15
KIAA1109	-0,981519631	6,442232845	4,46486E-16	4,19365E-15
RYBP	-0,696856892	6,409360573	4,64583E-16	4,35762E-15
ETS2	-1,14345754	8,152645805	4,97224E-16	4,65738E-15
HYOU1	1,056568015	8,452343586	5,04994E-16	4,72367E-15
MPZL1	0,625708008	7,935265851	5,18557E-16	4,84389E-15
SLC35F5	0,932247418	6,432870287	5,20383E-16	4,8543E-15
GLRX3	1,091926065	6,545340202	5,41169E-16	5,0413E-15
HEATR2	0,881794595	5,867672552	5,48166E-16	5,09951E-15
ZNF485	0,838036301	2,410627176	5,53009E-16	5,13756E-15
CXorf40B	0,685996276	4,784112096	5,74782E-16	5,33112E-15
BCS1L	0,644523826	4,465898688	5,75408E-16	5,33112E-15
KDM6B	-1,163438905	6,429490622	5,80006E-16	5,36644E-15
DNTTIP1	1,923812514	6,742515791	6,23756E-16	5,76341E-15
TMED9	0,750718744	8,329049391	6,397E-16	5,90273E-15
RPS6KB2	0,751157792	6,15197633	6,61208E-16	6,09295E-15
ZNF217	1,303224891	6,852956757	6,65086E-16	6,12041E-15
POLDIP2	0,641678272	7,330098008	7,00185E-16	6,43472E-15
ZDHHC9	1,249483178	7,823496627	7,23522E-16	6,64024E-15
NISCH	-0,816239436	6,656998422	7,37098E-16	6,75574E-15
NPR3	-1,549456528	3,02597658	7,81724E-16	7,15513E-15

RCN3	1,767488534	5,570628514	8,49678E-16	7,76669E-15
GTF2H4	0,704176382	4,946516748	9,02604E-16	8,23943E-15
MARS	0,823792489	7,235434695	9,13834E-16	8,33079E-15
STAT5A	-0,756565983	5,853134451	9,21658E-16	8,39091E-15
APOA1BP	1,160292287	7,417421533	9,25502E-16	8,41467E-15
MTHFR	-0,825393476	6,009525997	9,29038E-16	8,43557E-15
TSSC1	0,817988187	5,326938539	9,62664E-16	8,72926E-15
TLN1	-0,881942857	9,244063305	9,73754E-16	8,8181E-15
MDH2	0,757886547	7,962632167	9,99839E-16	9,04231E-15
BTG2	-1,324774701	8,016324347	1,00734E-15	9,09806E-15
STARD7	-0,578044738	8,099398848	1,0492E-15	9,46365E-15
FOXO1	-1,002905567	5,602743313	1,12754E-15	1,01568E-14
MTBP	1,132831014	2,84584902	1,16502E-15	1,04806E-14
TRPT1	0,836633156	4,723065525	1,16996E-15	1,05111E-14
GNAI2	-0,702191471	8,998925638	1,31541E-15	1,18024E-14
MRPS24	1,137771837	6,532684734	1,3537E-15	1,21299E-14
KDM5B	0,907406861	7,113149862	1,36454E-15	1,2211E-14
SLC9A3R2	-1,086682513	7,458290157	1,4109E-15	1,26093E-14
ZZEF1	-0,862416608	6,249261054	1,45254E-15	1,29645E-14
EGLN2	-0,657403876	6,989605191	1,46442E-15	1,30535E-14
MTIF2	0,601487783	5,588408196	1,48254E-15	1,31977E-14
SMARCA5	-0,603154668	7,26286463	1,53921E-15	1,36843E-14
DCUN1D5	0,914202888	5,04116776	1,61624E-15	1,43504E-14
EIF4EBP1	2,087886611	6,130780027	1,62885E-15	1,44436E-14
CASP6	0,864224226	5,135490354	1,65283E-15	1,46372E-14
SNF8	0,700894042	6,300819112	1,66602E-15	1,47348E-14
JAK2	-0,896666504	4,900987888	1,73444E-15	1,53201E-14
DSG2	1,383763856	8,134635206	1,77912E-15	1,56945E-14
METAP1	0,649150586	6,342490054	1,79145E-15	1,57828E-14
POLR1C	0,789021939	5,142003738	1,82185E-15	1,60299E-14
ZC3H8	0,9316264	2,991350812	1,83794E-15	1,61507E-14
CLCN2	1,08090232	2,976549704	1,92032E-15	1,68528E-14
DSN1	0,933682869	4,877030706	1,99046E-15	1,74459E-14
TBL2	0,782234869	6,400822638	2,05554E-15	1,79932E-14
MRPS23	1,12405497	6,431534179	2,13582E-15	1,8672E-14
NOTCH1	-1,123324398	6,424116917	2,14709E-15	1,87464E-14
PPP6C	-0,467817727	6,694558705	2,17474E-15	1,8955E-14
WDR47	-0,692552265	4,502633759	2,17653E-15	1,8955E-14
RAVER2	-0,938317816	4,578025687	2,27549E-15	1,97915E-14
UCK2	1,835303263	4,362692367	2,32876E-15	2,02291E-14
KIAA1009	-0,810609225	3,497946625	2,44685E-15	2,12279E-14
SLBP	0,749338289	6,265494166	2,4549E-15	2,1249E-14
KCNAB2	-1,083810991	5,373784146	2,45552E-15	2,1249E-14
MCM7	1,131330902	7,122718102	2,59129E-15	2,23941E-14
ACOT11	1,236512207	3,804301292	2,59442E-15	2,23941E-14
TCF3	0,852608283	6,498565707	2,78039E-15	2,3969E-14
RBMS3	-1,356914168	2,78787403	2,80749E-15	2,41721E-14
NAP1L5	-1,022356863	2,66844422	2,84114E-15	2,44309E-14
CDK4	1,309720333	7,689923064	2,92059E-15	2,50825E-14
EGR1	-1,665296899	8,921933808	3,0515E-15	2,61424E-14

SKI	-0,804565482	7,264038128	3,05167E-15	2,61424E-14
FOXO3	-0,922133418	6,837081153	3,05723E-15	2,61572E-14
COPB2	0,602196108	8,009710555	3,22962E-15	2,75962E-14
SORT1	-0,958389914	7,824060423	3,23352E-15	2,75962E-14
FANCD2	0,858087239	4,429515778	3,29088E-15	2,80506E-14
SEC61A1	0,651979439	9,237619224	3,35781E-15	2,85854E-14
DBN1	1,195789583	7,033115993	3,57702E-15	3,04136E-14
MAD2L2	1,195236267	5,390676116	3,63934E-15	3,0905E-14
PUS1	0,940306981	4,648950788	3,83406E-15	3,2518E-14
AMOTL2	-0,99227608	6,834819494	3,84441E-15	3,25652E-14
POLR1B	0,602312611	5,292844963	3,85591E-15	3,26222E-14
HIST1H2BK	1,330832002	6,743449099	3,92792E-15	3,31902E-14
ALOX5AP	-1,51935162	6,704683511	3,96988E-15	3,35032E-14
RFC2	0,809445808	5,610484811	4,03318E-15	3,39954E-14
TSEN54	0,670816569	5,400142579	4,07532E-15	3,43082E-14
DUSP18	-0,847318716	3,849108786	4,13425E-15	3,47613E-14
SLAIN2	-0,500481941	6,565976166	4,15745E-15	3,49134E-14
PRPF19	0,672920621	7,300193311	4,22761E-15	3,54589E-14
TBC1D17	-0,638703708	5,756972012	4,33417E-15	3,6308E-14
ZRANB1	-0,576167215	5,783558358	4,38167E-15	3,66609E-14
TBCEL	-0,799891334	4,732438205	4,5455E-15	3,7985E-14
DCTPP1	1,21903997	6,04356526	4,56677E-15	3,8116E-14
ISG20L2	0,643640197	5,685965125	4,65946E-15	3,88421E-14
SOX12	1,243532543	5,350733591	4,80504E-15	4,00068E-14
NSF	0,866452602	6,873875779	5,14725E-15	4,28038E-14
XPOT	1,055930619	7,059757942	5,24915E-15	4,3598E-14
FASTKD3	0,985397459	4,011424773	5,32814E-15	4,42002E-14
DOCK9	-1,137523435	7,179797649	5,49403E-15	4,5521E-14
SAC3D1	1,032146542	3,797418297	5,52772E-15	4,57446E-14
PDS5B	-0,60352766	5,568161323	5,56276E-15	4,59787E-14
KDELR1	0,62014916	8,287624115	5,99905E-15	4,95248E-14
CCT6A	1,04317238	8,170630597	6,06103E-15	4,9976E-14
PTK2B	-0,896657447	6,255868972	6,26394E-15	5,15867E-14
RABEP1	-0,541399458	6,12723871	6,55629E-15	5,39292E-14
MEA1	0,73985	6,461872351	6,70253E-15	5,50658E-14
MRPL30	0,690760312	5,89768463	6,91319E-15	5,67281E-14
ZMIZ1	-0,867253909	7,267976458	7,094E-15	5,81418E-14
CEP72	1,269180135	3,036726884	7,3686E-15	6,03199E-14
SNTB2	-0,680689788	5,785465601	7,66278E-15	6,26529E-14
UGDH	1,515058945	7,449210734	8,10674E-15	6,62034E-14
CASP3	0,769152053	5,739900585	8,44575E-15	6,88894E-14
EHD1	-0,874023648	7,07031564	8,8217E-15	7,187E-14
RARS	0,582920786	6,380151997	9,03948E-15	7,35563E-14
PRMT5	0,710469646	6,496400124	9,38954E-15	7,63138E-14
TMED2	0,925452735	9,25323901	9,50085E-15	7,71265E-14
PSMC4	0,893009788	7,012277305	9,52997E-15	7,7271E-14
PJA2	-0,634696531	7,608975096	9,65282E-15	7,8174E-14
IER5L	1,676820673	4,443951165	9,82454E-15	7,94704E-14
PLOD1	0,969242567	7,889028152	9,88362E-15	7,98535E-14
CYP20A1	-0,516506074	6,224373042	9,90237E-15	7,99104E-14

HARBI1	0,691182343	2,512112278	1,02266E-14	8,24295E-14
FUBP1	0,606775055	6,694239733	1,03136E-14	8,30328E-14
FOXP1	-0,786667092	6,73046885	1,05281E-14	8,46592E-14
LOXL2	2,11886069	6,311822547	1,08183E-14	8,68908E-14
ICT1	0,984675978	5,188935639	1,20677E-14	9,68113E-14
PMAIP1	2,029642091	5,006378014	1,22832E-14	9,8424E-14
CABLES1	-1,26349368	5,784639798	1,26439E-14	1,01196E-13
VPS13D	-0,929710723	6,675533395	1,32175E-14	1,05663E-13
LRRC16A	-0,805462618	5,592347659	1,34111E-14	1,07085E-13
TBRG1	-0,566946127	5,867201665	1,383E-14	1,10301E-13
TCF4	-1,13246654	6,61277933	1,39603E-14	1,1121E-13
MOAP1	-0,747551773	5,744094484	1,41153E-14	1,12313E-13
SLC39A4	1,256443042	5,950998309	1,47528E-14	1,17248E-13
ZNF92	0,866807168	4,067690428	1,51882E-14	1,20569E-13
CYC1	1,189130709	7,334499455	1,52222E-14	1,20698E-13
LIG3	0,812072594	5,328280641	1,65069E-14	1,30732E-13
NES	-1,534365884	6,682803931	1,65957E-14	1,31283E-13
KLC1	-0,617012243	6,661471864	1,66369E-14	1,31456E-13
ZBTB41	0,695462123	5,681459849	1,69736E-14	1,33962E-13
MTHFD1L	0,775053071	5,360492975	1,71127E-14	1,34904E-13
SLC1A4	1,073833848	5,854180312	1,78264E-14	1,40367E-13
UBP1	-0,525959597	6,609001863	1,78482E-14	1,40377E-13
TMEM106C	1,257368531	6,876983545	1,83195E-14	1,43918E-13
YIF1A	0,886255628	6,762474099	1,92853E-14	1,51331E-13
BZW1	0,842825652	9,012654755	1,95267E-14	1,53049E-13
SLC16A3	1,423011421	7,827116333	1,97503E-14	1,54624E-13
C11orf83	1,198017692	4,801582833	2,16017E-14	1,68924E-13
TOMM40	0,869491521	6,486931328	2,16986E-14	1,69487E-13
RCN1	0,810698703	7,873173633	2,29324E-14	1,7892E-13
RUNX2	1,435108117	4,378713279	2,4288E-14	1,8928E-13
CLDN4	1,198267268	9,142662492	2,48691E-14	1,93587E-13
ERGIC2	0,885134222	6,079797955	2,52296E-14	1,96169E-13
UNC13B	-1,180883697	7,520296312	2,58725E-14	2,00939E-13
METTL2A	0,644648423	5,292765221	2,66173E-14	2,06488E-13
TMEM106B	0,967045055	7,325988048	2,78526E-14	2,15826E-13
PDF	0,66991624	3,163453834	2,978E-14	2,30499E-13
NUDT5	0,803625788	5,935087532	3,03345E-14	2,34525E-13
DNMT3A	1,022155815	5,502384148	3,06706E-14	2,36855E-13
TBC1D20	-0,530908326	6,197741935	3,08332E-14	2,37841E-13
EMILIN2	-1,09141504	5,801888552	3,15462E-14	2,43066E-13
JUNB	-1,026086342	8,203731003	3,17422E-14	2,443E-13
CAMTA2	-0,677403494	6,079461533	3,17987E-14	2,44459E-13
CD276	0,775779148	7,231626619	3,23991E-14	2,48795E-13
COL13A1	-1,43284905	3,297153527	3,29844E-14	2,53004E-13
SURF4	0,581346947	8,892131477	3,33867E-14	2,55802E-13
MINK1	-0,759263469	7,097946395	3,34384E-14	2,55911E-13
RNASEH1	0,672602612	4,423719853	3,43276E-14	2,62422E-13
LOXL1	1,389055894	5,167446794	3,55543E-14	2,71439E-13
FAM114A1	0,85907081	5,692766984	3,55868E-14	2,71439E-13
CD81	-0,790089503	9,732717911	3,59685E-14	2,74044E-13

SGCB	-0,84324975	5,816105503	3,60766E-14	2,74561E-13
POP7	1,03034785	5,249851059	3,65225E-14	2,77644E-13
GPX8	1,287808318	5,459877267	3,84382E-14	2,91882E-13
MPP5	-0,646890726	5,980621607	3,85568E-14	2,92457E-13
TUBA1C	0,985136108	8,964274934	3,95288E-14	2,99497E-13
GSS	0,684941947	6,17339258	4,17572E-14	3,16029E-13
COIL	0,550861514	5,13112986	4,22998E-14	3,19781E-13
CHD1L	0,825506918	6,288348837	4,34916E-14	3,28426E-13
TTF2	0,772161664	4,514258571	4,55968E-14	3,43943E-13
ARID4A	-0,783740917	5,208348279	4,67278E-14	3,52085E-13
PTGIS	-1,36572202	5,876267312	4,69081E-14	3,53053E-13
VPS25	0,699379771	6,238530338	4,71512E-14	3,54492E-13
GPX3	-1,692198986	9,258393056	4,73382E-14	3,55394E-13
TMEM87B	0,632037083	6,511348273	4,73754E-14	3,55394E-13
RRP1	0,739557878	5,267485156	4,76242E-14	3,56867E-13
MKNK2	-0,763277404	7,999738326	4,78467E-14	3,58141E-13
TSFM	1,061435494	5,608314575	5,17998E-14	3,87306E-13
NFAT5	-0,856631013	6,468945127	5,2999E-14	3,95838E-13
ANKFY1	-0,657595985	6,723725936	5,35129E-14	3,99239E-13
SASS6	0,909587454	3,114798713	5,4296E-14	4,04639E-13
PIAS3	0,803654729	6,210904576	5,54454E-14	4,12753E-13
HNRNPL	0,381853335	8,666066967	5,59635E-14	4,15751E-13
MRPS17	0,937956982	4,286057169	5,597E-14	4,15751E-13
FRYL	-0,692448087	6,137827468	5,66167E-14	4,20097E-13
NPHP3	-0,701409959	4,525987773	6,10954E-14	4,52837E-13
SNRPA	0,590842668	6,075205938	6,81121E-14	5,04296E-13
SLC39A7	0,768525257	8,10714937	6,9699E-14	5,15485E-13
GTF2E2	0,745250367	5,455418138	7,0749E-14	5,22684E-13
GEMIN6	1,033101797	3,977460311	7,16529E-14	5,28789E-13
RNPS1	0,487255897	7,539021868	7,19748E-14	5,30294E-13
CCT7	0,620860172	8,265180704	7,20124E-14	5,30294E-13
LRRC42	0,789029094	5,49764247	7,22421E-14	5,31382E-13
OSTC	1,073753254	7,318514457	7,2316E-14	5,31382E-13
FBXW4	-0,803613895	6,359721031	7,59152E-14	5,57066E-13
MEMO1	0,832571591	5,667423297	7,59747E-14	5,57066E-13
SLC25A30	-0,635634381	4,821278466	7,62718E-14	5,58644E-13
UQCRH	0,962021418	7,262231655	7,82315E-14	5,72382E-13
C10orf35	0,99296207	4,001890606	7,89923E-14	5,7733E-13
SOCS3	-1,391946651	8,305904431	8,06022E-14	5,8838E-13
SAMHD1	-1,10275397	6,881962636	8,06768E-14	5,8838E-13
ANKRD12	-0,725117705	6,015886371	8,17302E-14	5,95426E-13
FBXL6	0,925467061	4,828655054	8,23954E-14	5,99631E-13
CCDC58	1,033802408	4,222594271	8,29874E-14	6,03295E-13
MTCH2	0,623784796	6,793989248	8,40699E-14	6,10514E-13
DNAJB11	0,794016497	6,712923125	8,52708E-14	6,18576E-13
FOS	-1,616837947	9,162966295	8,5682E-14	6,20899E-13
ITPKC	-1,201818713	6,90890999	8,63638E-14	6,25175E-13
PPP2R5A	-0,82589663	7,251260591	8,99099E-14	6,50154E-13
MED8	0,601602281	5,885820453	9,07136E-14	6,54894E-13
ADAMTS12	1,796605428	3,736431143	9,07574E-14	6,54894E-13

LEPRE1	0,811528985	5,816560125	9,10202E-14	6,56096E-13
ARHGAP23	-1,106941529	6,169325935	9,33446E-14	6,7214E-13
DDX41	0,602017789	6,473111514	9,55E-14	6,86935E-13
CORO1C	-0,75302301	7,947046524	9,78348E-14	7,02274E-13
DYNC2H1	-1,403841267	4,46398009	9,78384E-14	7,02274E-13
HSP90B1	0,817361428	10,35815791	9,87089E-14	7,07777E-13
NDUFS6	1,01656463	6,423787424	1,01836E-13	7,29429E-13
TH1L	0,730132036	6,622953047	1,03548E-13	7,40917E-13
PAK1IP1	0,842851392	4,933911664	1,05013E-13	7,50611E-13
TFAM	0,730414213	5,42660643	1,06365E-13	7,59481E-13
WASF2	-0,679535169	7,882095708	1,08534E-13	7,74154E-13
ADAM15	1,079489666	8,210799103	1,09825E-13	7,82549E-13
TTC7B	-0,896335029	4,553519557	1,10575E-13	7,87068E-13
DAB2	-1,041862534	6,95813246	1,11119E-13	7,90114E-13
DENR	0,577604987	6,694336672	1,11618E-13	7,92835E-13
AMZ2	0,673203644	6,532945344	1,13265E-13	8,037E-13
HDAC7	-0,644424191	6,98878826	1,14841E-13	8,14037E-13
CD33	-1,173071163	3,703847505	1,15801E-13	8,19985E-13
PFDN2	1,009786232	6,192266184	1,2361E-13	8,74377E-13
LPGAT1	0,847796254	7,003612662	1,28139E-13	9,05474E-13
LTA4H	-0,889415222	8,441273739	1,29577E-13	9,14681E-13
TOR3A	0,722368633	6,537222912	1,33193E-13	9,39235E-13
NHP2	0,893007365	6,580904146	1,34836E-13	9,49843E-13
EIF4EBP2	-0,508622782	7,928289245	1,39994E-13	9,85156E-13
RASSF8	-1,331880539	5,610664144	1,40249E-13	9,85938E-13
EIF3I	0,721191122	8,19440289	1,42841E-13	1,00313E-12
SLCO3A1	-0,977256405	5,904622106	1,43735E-13	1,00836E-12
HOMER3	0,972442105	5,373819927	1,44804E-13	1,01482E-12
GPN1	0,872362944	6,271137242	1,46867E-13	1,02822E-12
NIF3L1	0,738529412	5,144930051	1,49235E-13	1,04358E-12
PABPC3	0,989505458	2,319169148	1,49368E-13	1,04358E-12
E2F5	1,055511311	4,212206664	1,50017E-13	1,04704E-12
PHB	0,675397326	7,74864039	1,50674E-13	1,05056E-12
PSMD3	0,794635753	7,751865524	1,53666E-13	1,07033E-12
SEPP1	-1,38877682	9,158721337	1,56949E-13	1,09208E-12
EID1	-0,59707636	7,764486071	1,57736E-13	1,09644E-12
FAM98A	0,562582442	5,789841025	1,60762E-13	1,11633E-12
ARHGEF2	-0,744317851	7,301573599	1,61038E-13	1,11711E-12
PGK1	1,082082087	9,951399819	1,65343E-13	1,14581E-12
EBNA1BP2	0,678291322	6,362717784	1,67782E-13	1,16153E-12
MRPS10	0,835190593	6,425037119	1,78421E-13	1,23393E-12
RNF125	-0,753768831	4,383825017	1,80096E-13	1,24314E-12
LSM2	1,072721799	5,969439028	1,80118E-13	1,24314E-12
GPS1	0,612054539	7,160238136	1,8034E-13	1,24342E-12
C2	-1,231298126	7,90006191	1,83044E-13	1,26078E-12
DCPS	0,692291863	4,67014695	1,88241E-13	1,29527E-12
PQLC1	-0,609470017	6,070755433	1,94445E-13	1,33661E-12
VIM	-1,031523093	10,74352152	2,04544E-13	1,40345E-12
FBXL19	0,940489611	4,741614424	2,0458E-13	1,40345E-12
FKBP4	1,192230645	7,327993616	2,06365E-13	1,41428E-12

WDYHV1	0,964734638	4,121078545	2,11154E-13	1,44564E-12
PSMB3	1,091758463	7,578528987	2,14257E-13	1,46541E-12
PNRC2	-0,524959256	7,276256414	2,1706E-13	1,4831E-12
MMP14	1,38763302	8,773947015	2,38329E-13	1,62679E-12
COBLL1	-1,006384132	5,9117775	2,40178E-13	1,63778E-12
GRN	-0,726480715	10,01346702	2,42704E-13	1,65334E-12
C1QA	-1,303918218	8,715353846	2,43403E-13	1,65645E-12
PAIP1	0,755814812	7,423499967	2,45228E-13	1,66721E-12
C12orf23	0,86609907	6,720947898	2,54364E-13	1,7276E-12
VSIG10	-1,084081283	5,545282442	2,60748E-13	1,76919E-12
UBE2G2	-0,559145607	6,657820583	2,64962E-13	1,796E-12
NELF	1,049305176	5,891891475	2,71983E-13	1,84176E-12
FAM174A	-0,700871896	4,995535169	2,74643E-13	1,85793E-12
CREBL2	-0,617309965	6,843065849	2,79702E-13	1,89028E-12
VCAN	1,747451685	8,245346025	2,8118E-13	1,89838E-12
C12orf49	-0,907509848	6,537551951	2,88609E-13	1,94661E-12
NET1	1,223183154	7,739821887	2,99558E-13	2,01846E-12
C5orf43	0,607153885	6,595437823	3,07749E-13	2,07161E-12
WWP2	-0,736090543	6,788889218	3,10218E-13	2,08617E-12
MDFI	1,569450184	4,835891105	3,10654E-13	2,08704E-12
IL17RA	-0,663089342	4,765992024	3,1218E-13	2,09523E-12
CDH24	1,38550057	3,795700995	3,15476E-13	2,11527E-12
C17orf89	0,975896442	4,295916372	3,23727E-13	2,16846E-12
TUBB	0,836263376	10,11870098	3,35149E-13	2,24277E-12
ACSL4	-0,917370624	7,533240932	3,41305E-13	2,28172E-12
C11orf84	0,815481127	5,088501386	3,54339E-13	2,36654E-12
ARHGAP29	-1,185400344	6,746067698	3,55528E-13	2,37216E-12
PNPLA2	-0,783413225	6,761711768	3,56143E-13	2,37394E-12
RHOD	1,404205613	5,221353658	3,57725E-13	2,38215E-12
NMRAL1	0,726906192	5,778625066	3,58815E-13	2,38709E-12
GTF3C2	0,482036236	6,549353973	3,67713E-13	2,44389E-12
C12orf52	0,658566711	5,044817114	3,71921E-13	2,46945E-12
C11orf10	0,79744283	6,805531003	3,7586E-13	2,49318E-12
CCNL1	-0,861771053	6,321464896	3,79538E-13	2,51513E-12
CRELD2	0,955145814	5,988399472	3,81415E-13	2,52512E-12
WASF1	1,358668872	3,405112035	3,82775E-13	2,53166E-12
BOLA3	1,114915061	4,421448181	3,97689E-13	2,62775E-12
SCNM1	0,840185519	5,400339384	3,99058E-13	2,63425E-12
NOP16	1,051601025	4,564199861	4,05632E-13	2,67505E-12
AKAP12	-1,692446588	6,604700916	4,22946E-13	2,78405E-12
RPN2	0,698434514	9,246167448	4,22976E-13	2,78405E-12
ZNRD1	0,827256153	4,826093779	4,28188E-13	2,81563E-12
SETX	-0,655760542	6,877167789	4,41469E-13	2,90017E-12
SCD	-1,187445434	9,451604902	4,49059E-13	2,94719E-12
ENOPH1	0,619406466	6,076188844	4,63869E-13	3,04147E-12
ATF3	-1,492698038	6,807578131	4,73685E-13	3,10284E-12
SAR1B	0,968391356	5,650697493	4,75029E-13	3,10866E-12
HIST1H2BC	1,79245984	3,200012168	4,81838E-13	3,15019E-12
MEN1	0,600170914	5,7051733	4,8616E-13	3,1754E-12
TIMM17B	1,01824555	6,014207639	4,97247E-13	3,24471E-12

C1orf109	0,610314694	4,389217794	5,24371E-13	3,41844E-12
ERGIC3	0,636410157	8,588960058	5,3094E-13	3,45795E-12
CISD2	0,789302281	6,065701684	5,38291E-13	3,50249E-12
CDYL	0,588134083	5,517562819	5,39239E-13	3,50531E-12
WDR77	0,598738585	5,60313328	5,44772E-13	3,53791E-12
NAA10	0,966005976	6,233290486	5,4937E-13	3,56437E-12
PER1	-1,261777706	7,409100271	5,54732E-13	3,59574E-12
PPP4C	0,826347305	7,313409038	5,62995E-13	3,64583E-12
SBDSP1	-0,695994703	5,312625868	5,7449E-13	3,71674E-12
NVL	0,535453105	5,158582054	5,8617E-13	3,78872E-12
SNRPD1	0,925452762	6,028185137	5,94907E-13	3,84154E-12
RSU1	-0,478830955	6,909688625	6,02838E-13	3,88908E-12
PRELP	-1,375579664	7,355668735	6,05698E-13	3,90187E-12
MSH2	0,927333508	5,75258725	6,05965E-13	3,90187E-12
BNIP3	1,223728445	6,893242037	6,18202E-13	3,9769E-12
HIPK1	-0,751157911	6,981826844	6,42472E-13	4,12914E-12
MRPL10	0,55717546	6,072403433	6,45818E-13	4,14604E-12
RAN	0,994315015	8,542590236	6,46317E-13	4,14604E-12
POSTN	1,777485423	8,362810961	6,50245E-13	4,16731E-12
PSMB5	0,768515404	7,287426607	6,53465E-13	4,18402E-12
POLA2	0,782092971	4,686724965	6,61401E-13	4,23086E-12
S100A2	3,564822101	6,23180611	6,66193E-13	4,25752E-12
C1orf122	0,823992766	5,546278413	6,72257E-13	4,29044E-12
CNTROB	-0,722578171	5,763742308	6,72602E-13	4,29044E-12
NT5E	2,219346334	6,796582537	6,75781E-13	4,30668E-12
RAE1	0,830829232	5,493005973	7,00976E-13	4,46307E-12
GAS7	-1,053310816	5,952226496	7,15809E-13	4,55189E-12
PDIA3P	0,749644619	5,838613497	7,1626E-13	4,55189E-12
CDK5	0,825948906	4,57316651	7,49527E-13	4,75887E-12
MBNL1	-0,644937168	8,106104115	7,64872E-13	4,85178E-12
POLR3K	0,851750575	4,728298302	7,74729E-13	4,90973E-12
ANKRD44	-0,962741868	3,837293399	8,22003E-13	5,20449E-12
CDKN1C	-1,062236932	4,719404892	8,2541E-13	5,22121E-12
DCP1A	-0,522662601	5,605141395	8,81151E-13	5,56864E-12
SEC61G	0,933570181	6,49858946	9,09734E-13	5,74396E-12
CPD	1,454676436	8,7711557	9,39727E-13	5,92784E-12
ARHGEF17	-0,853633755	6,481229316	9,53735E-13	6,01065E-12
CIRH1A	0,561528417	5,867760704	9,56673E-13	6,02359E-12
TUBB6	-1,025090806	6,880913734	9,71494E-13	6,11127E-12
FKBP3	0,91213289	6,354301864	1,00025E-12	6,28636E-12
H2AFZ	0,962683099	7,832391922	1,0209E-12	6,41025E-12
HNRNPAB	0,70581454	7,531606255	1,03497E-12	6,4926E-12
B3GALNT1	-1,108213313	4,965809325	1,05246E-12	6,59626E-12
XPC	-0,705750258	5,911100179	1,0566E-12	6,6161E-12
NCOA1	-0,743103115	6,330700999	1,06778E-12	6,67997E-12
PSMD7	0,600050273	7,019421319	1,073E-12	6,70647E-12
MXRA5	1,775035354	7,420366083	1,07601E-12	6,71912E-12
ZDHHC18	0,709979715	6,04255687	1,08586E-12	6,77443E-12
APOL4	-0,93673331	5,444382261	1,08853E-12	6,78492E-12
PSMA5	0,84084106	7,143283703	1,12758E-12	7,02188E-12

NFE2L3	1,514641499	5,975972529	1,13053E-12	7,03384E-12
SPINT2	0,995310446	9,579155747	1,15725E-12	7,19353E-12
RPS2	0,8160553	10,77925175	1,17314E-12	7,28564E-12
PSMB2	0,585820798	7,271318373	1,17951E-12	7,31857E-12
DUSP1	-1,329570356	9,301824095	1,22474E-12	7,59226E-12
MORC2	0,627874601	5,941598744	1,23491E-12	7,64838E-12
STAMBP	0,595183765	5,744732953	1,24011E-12	7,67357E-12
NSUN2	0,848366297	6,904177142	1,25888E-12	7,78268E-12
MCM3	0,777423163	6,897504213	1,27028E-12	7,84022E-12
POLD2	0,723508486	6,861935269	1,27049E-12	7,84022E-12
COL1A2	1,664389121	11,13656658	1,27751E-12	7,87646E-12
C15orf23	1,196836724	4,500908574	1,30193E-12	8,01974E-12
NOP56	0,730583405	6,870332849	1,33372E-12	8,20813E-12
TIMM9	0,836016618	5,095201907	1,34428E-12	8,26569E-12
MRPS33	1,004535162	5,612152907	1,3523E-12	8,30751E-12
ABCF2	0,563750349	6,489403278	1,35832E-12	8,33699E-12
RNF34	0,56662541	5,343646847	1,37207E-12	8,41379E-12
SNRPB	0,917079705	7,857077746	1,41329E-12	8,65879E-12
FAM60A	1,189652417	7,267459503	1,46584E-12	8,97268E-12
UBQLN4	0,713563902	6,907360076	1,4798E-12	9,05002E-12
PDXK	-0,694196247	7,864372321	1,48647E-12	9,08262E-12
FAM3C	1,428959192	7,8660886	1,54316E-12	9,4206E-12
EIF4A3	0,685173471	6,978356985	1,55043E-12	9,45649E-12
PGAP1	-0,897793329	4,438084196	1,57057E-12	9,57078E-12
NOC2L	0,693841058	6,953412235	1,61866E-12	9,85503E-12
ACBD6	0,605911933	5,41706321	1,62165E-12	9,86444E-12
FCHSD2	-0,559520923	6,106811724	1,64353E-12	9,9886E-12
JMJD1C	-0,679170199	6,518222323	1,66198E-12	1,00917E-11
ATG16L1	0,540135749	5,590715852	1,68168E-12	1,02023E-11
ZC3HAV1L	0,943813462	2,3235851	1,70918E-12	1,03599E-11
RRM1	0,651108228	6,378805143	1,74398E-12	1,05614E-11
FAM129B	-0,718516989	9,110540766	1,76367E-12	1,06712E-11
ZNF107	0,88857262	3,97922781	1,77273E-12	1,07165E-11
BCKDK	0,73283499	6,422139347	1,79961E-12	1,08694E-11
SAMD4A	-1,089575	5,455009024	1,80844E-12	1,09131E-11
STK10	-0,777792182	6,086311657	1,82532E-12	1,10052E-11
AKAP9	-0,836154083	6,633345041	1,8646E-12	1,12321E-11
TRIB3	1,450054893	5,670256277	1,88872E-12	1,13673E-11
LYPLA1	1,225389795	7,587039432	1,93095E-12	1,16112E-11
PDE3B	-1,117690884	4,743116049	1,9681E-12	1,18242E-11
JTB	0,825332904	7,860633159	2,04626E-12	1,22829E-11
EIF3B	0,795195538	8,106873914	2,06776E-12	1,23924E-11
NFATC3	-0,762299959	5,944004873	2,06813E-12	1,23924E-11
SKIV2L	0,555251949	6,195443631	2,21993E-12	1,32904E-11
NAT10	0,586815422	6,285710635	2,22996E-12	1,33387E-11
PRPF4	0,572609491	5,645900659	2,40132E-12	1,43511E-11
MRPS16	0,617529573	6,935768326	2,41495E-12	1,44199E-11
VAMP3	-0,50142442	7,52806854	2,42239E-12	1,44517E-11
DDX17	-0,568068021	9,011010853	2,51179E-12	1,49719E-11
ITPK1	-0,735850648	6,603181082	2,53592E-12	1,51026E-11

RSL1D1	0,574180247	7,951399193	2,65841E-12	1,58182E-11
NCKAP5L	-0,674724161	5,380918637	2,69961E-12	1,60494E-11
TCEB1	0,965141497	6,310187253	2,72825E-12	1,62055E-11
HSP90AB1	0,600210449	10,80816013	2,73933E-12	1,62571E-11
CORO1B	0,561086024	7,397154637	2,86671E-12	1,69984E-11
SNHG1	1,290951173	5,317339644	2,93963E-12	1,74156E-11
USP39	0,590854458	6,488418042	3,02089E-12	1,78815E-11
ABCB6	1,183716368	5,874984704	3,17504E-12	1,87776E-11
CCT4	0,716104045	8,002845269	3,25964E-12	1,92613E-11
OTUD4	-0,542629623	6,403621371	3,34842E-12	1,97687E-11
CXCL16	-0,925049408	7,980315965	3,4392E-12	2,02871E-11
KIAA0754	-1,176690648	3,565789158	3,46941E-12	2,04476E-11
KDSR	-0,502338035	6,62093271	3,51266E-12	2,06846E-11
CAPRIN1	0,427624846	8,499617103	3,55977E-12	2,0944E-11
MLST8	0,562960697	5,732323411	3,58624E-12	2,10815E-11
SMARCB1	0,485387272	6,553473334	3,64216E-12	2,13797E-11
NUP37	0,849727709	4,79651379	3,64323E-12	2,13797E-11
KRTCAP3	1,687597765	6,021176997	3,71275E-12	2,17689E-11
ZFP64	0,559101421	4,796447718	3,73171E-12	2,18613E-11
NFKBIA	-1,014773374	8,602166917	3,73717E-12	2,18745E-11
MRPL19	0,504589897	6,224714937	3,79077E-12	2,21692E-11
PPIB	0,909021083	9,358147198	3,82716E-12	2,23628E-11
CYP4V2	-0,72487871	5,832992381	3,84221E-12	2,24315E-11
SSR1	0,56338005	8,297758167	3,95524E-12	2,30717E-11
SHFM1	0,869777628	6,888574573	3,99271E-12	2,32703E-11
PARVG	-0,986740369	5,076098014	4,0238E-12	2,34315E-11
DDX52	0,747962662	5,581141529	4,04921E-12	2,35594E-11
TLE4	-0,990572845	4,894789463	4,19983E-12	2,44009E-11
UBE2O	0,700406333	5,846929891	4,20101E-12	2,44009E-11
DPY30	0,874657315	5,81470222	4,2406E-12	2,46099E-11
GALNT2	0,74874499	7,695891892	4,28896E-12	2,48694E-11
DSP	1,814274563	8,637210957	4,36269E-12	2,52754E-11
PKD2	-0,755668996	5,603851637	4,55261E-12	2,63533E-11
FEM1C	-0,66107163	5,882589592	4,59665E-12	2,65857E-11
DPY19L1	1,113387231	7,210870287	4,71027E-12	2,72197E-11
C1orf56	0,757401386	4,247697223	4,73087E-12	2,73057E-11
PSMA1	0,586116032	7,469607753	4,73314E-12	2,73057E-11
P4HA1	0,917402643	7,275159011	4,74642E-12	2,73591E-11
ISG15	1,771574656	7,021787903	4,89722E-12	2,82045E-11
WBSCR22	0,718255447	6,881182818	5,05835E-12	2,91079E-11
TOMM70A	0,551195366	6,822264818	5,10762E-12	2,93667E-11
SSSCA1	0,710382841	4,794040277	5,15779E-12	2,96302E-11
NDUFS8	0,866938599	6,643004334	5,19314E-12	2,98081E-11
RPL37	0,899338935	10,06592067	5,21508E-12	2,99089E-11
MAST2	0,681299399	6,017094141	5,47856E-12	3,13935E-11
STARD4	-1,013301301	4,00241681	5,56986E-12	3,18899E-11
SEC13	0,614022191	7,315066177	5,68324E-12	3,25117E-11
C1QB	-1,301610011	9,074947619	5,6896E-12	3,25209E-11
FAM21A	-0,584470979	5,623379212	5,69821E-12	3,25428E-11
PRELID1	0,778001169	7,269223988	5,82107E-12	3,32166E-11

DLG5	0,935843494	6,329814654	5,9878E-12	3,41395E-11
GALNT6	1,626563674	6,425220957	6,02096E-12	3,42998E-11
PDIA3	0,779109726	9,893967678	6,13199E-12	3,49032E-11
GOLGA5	0,480350865	6,574382014	6,16266E-12	3,50485E-11
MCTS1	0,602663511	6,122720664	6,51371E-12	3,70142E-11
HMGN1	0,692456755	8,128108213	6,85059E-12	3,88961E-11
DHTKD1	0,7308945	5,936419934	7,0272E-12	3,98656E-11
FAM162A	0,968080428	5,905895512	7,40596E-12	4,19794E-11
GTPBP2	0,569262509	6,391624174	7,477E-12	4,23469E-11
TMCO1	0,726683958	7,5117519	7,54748E-12	4,27106E-11
SLCO2B1	-1,183015597	7,544615474	7,78955E-12	4,40439E-11
PUF60	0,637555237	7,676456649	8,08399E-12	4,56709E-11
ITSN2	-0,56933897	6,611923096	8,09238E-12	4,56804E-11
AZI2	-0,484828587	5,979682502	8,24139E-12	4,64791E-11
AGR2	2,237067511	9,300125989	8,2475E-12	4,64791E-11
XPO1	0,533100515	7,847685841	8,25984E-12	4,65102E-11
PRCC	0,567173561	6,640668757	8,41451E-12	4,7342E-11
NOMO1	0,630316332	7,088144558	8,5371E-12	4,79921E-11
FH	0,609855556	6,448224144	8,94798E-12	5,02605E-11
SAR1A	-0,428284812	7,488221043	9,00574E-12	5,05433E-11
VPS33A	0,522211507	5,734133844	9,01819E-12	5,05689E-11
EFTUD2	0,542939633	7,274261515	9,02513E-12	5,05689E-11
KIAA1377	-1,133436203	3,991597482	9,47027E-12	5,30195E-11
NUDCD1	0,862287572	5,286140416	9,63805E-12	5,39146E-11
AIFM1	0,577307861	5,901941389	9,88166E-12	5,5232E-11
KIAA0907	0,818745573	5,843171189	1,00371E-11	5,60547E-11
PKD1	-0,874142874	6,5440513	1,02784E-11	5,73555E-11
TMEM170B	-0,89209955	4,31049077	1,04364E-11	5,81895E-11
ITM2B	-0,694401863	10,0785098	1,05266E-11	5,86444E-11
EXOSC1	0,633691514	5,018295625	1,07548E-11	5,9867E-11
CDK17	-0,549279887	5,593337501	1,08297E-11	6,02347E-11
TAF1D	0,663752255	5,560716466	1,09231E-11	6,06795E-11
MLLT4	-0,796705212	7,510184972	1,09275E-11	6,06795E-11
LRRFIP2	-0,508365064	5,72979771	1,12835E-11	6,26057E-11
TUBB2A	1,376017082	5,385644954	1,15651E-11	6,41158E-11
GTF2IRD2B	-0,680264204	4,983955713	1,1621E-11	6,43731E-11
TIMM10	0,861806233	5,018451263	1,17045E-11	6,47831E-11
HAX1	0,866443678	7,155669907	1,19594E-11	6,61405E-11
CSTF3	0,493586276	5,268352155	1,25443E-11	6,93188E-11
ZC3H15	0,55791694	6,597458758	1,28507E-11	7,09545E-11
NUDT9	-0,472897211	5,423417883	1,30403E-11	7,19433E-11
RASAL2	-0,804508374	5,809153959	1,30595E-11	7,19703E-11
TTBK2	-0,881591961	2,696311487	1,30663E-11	7,19703E-11
YARS2	0,841787322	4,679980236	1,32011E-11	7,26539E-11
CCDC43	0,585679282	5,317577208	1,32766E-11	7,30107E-11
PHF6	0,540364659	5,428517362	1,34703E-11	7,40162E-11
NOP2	0,961108077	5,740845134	1,36386E-11	7,48808E-11
PDCD5	1,000444736	6,114404126	1,38337E-11	7,58904E-11
DGAT1	0,786677334	6,098777678	1,39065E-11	7,62286E-11
MTA3	0,640412963	5,772338084	1,40214E-11	7,67968E-11

WHSC2	0,51692197	5,428283807	1,42889E-11	7,81988E-11
MRPL42	0,77016465	5,763182882	1,44055E-11	7,87738E-11
PDCL3	0,636805222	5,25120762	1,54041E-11	8,41671E-11
GJA1	-1,248067326	7,799363183	1,59673E-11	8,71744E-11
C1orf21	-1,140961126	6,259315358	1,61997E-11	8,83727E-11
RFC3	0,904461744	4,288394491	1,6226E-11	8,84455E-11
TRAF2	0,7960779	5,066289111	1,64301E-11	8,94861E-11
LY75	-0,767322675	6,983826225	1,67066E-11	9,09199E-11
ALG6	0,584316312	4,268053385	1,70326E-11	9,26197E-11
ANP32E	1,061253026	7,24224212	1,72128E-11	9,35252E-11
ABCE1	0,53259439	6,729296609	1,75151E-11	9,5092E-11
YKT6	0,687080933	7,228288981	1,80512E-11	9,79247E-11
RNF149	-0,507421348	6,766263643	1,81609E-11	9,84414E-11
SMS	0,850193012	7,144926473	1,86257E-11	1,00881E-10
SLMO2	0,858816519	7,227547915	1,86682E-11	1,01031E-10
PRIM1	0,987358442	3,747621452	1,87463E-11	1,01373E-10
NAA25	0,631857414	5,163769039	1,88008E-11	1,01587E-10
ARMC1	0,739355895	5,869122642	1,88848E-11	1,0196E-10
STK4	-0,530413092	6,264299993	1,91091E-11	1,0309E-10
PIGC	0,658188275	5,747326601	1,91771E-11	1,03374E-10
C14orf166	0,645074275	7,19686283	1,95979E-11	1,05559E-10
SCAMP3	0,608267674	6,897962752	1,96962E-11	1,06005E-10
SNRPG	1,176051627	6,59108664	1,99497E-11	1,07285E-10
RRAGB	-0,610064405	3,943498468	1,99822E-11	1,07375E-10
MPHOSPH9	0,919677788	3,758678524	2,00565E-11	1,07689E-10
AUP1	0,714518296	7,755986559	2,07311E-11	1,11224E-10
HIST1H1C	1,493441556	5,951573711	2,11729E-11	1,13505E-10
PTPN6	-0,640952844	6,751080581	2,1309E-11	1,14145E-10
RAP1A	-0,578347376	6,896020201	2,16093E-11	1,15662E-10
CARM1	0,504839319	6,220318011	2,16329E-11	1,15698E-10
MGC72080	1,035497575	3,62076693	2,18033E-11	1,16518E-10
WDR45L	0,687130717	7,547278237	2,19088E-11	1,1699E-10
ACAD11	-0,718535071	4,267254329	2,28074E-11	1,21693E-10
ZNF44	-0,583416841	3,960866216	2,34424E-11	1,24984E-10
ZNHIT3	0,664388305	5,530080703	2,35262E-11	1,25332E-10
CDK14	-0,911803297	5,232125716	2,38679E-11	1,27053E-10
CHAF1A	0,915096882	4,454613851	2,39844E-11	1,27574E-10
CKS2	1,512759106	5,641896443	2,42637E-11	1,28891E-10
LIPA	-1,08628142	8,343633117	2,42698E-11	1,28891E-10
MAZ	0,650787757	7,765566966	2,60776E-11	1,38329E-10
DIAPH2	-0,750617215	4,69513851	2,60875E-11	1,38329E-10
GCSH	1,015490072	5,074788398	2,61796E-11	1,38709E-10
CCT8	0,511233303	7,912585507	2,64541E-11	1,40055E-10
MSH6	0,769473294	6,522857641	2,72523E-11	1,44169E-10
FBXO22	0,67515936	4,987220971	2,74336E-11	1,45015E-10
HDAC4	-0,682651552	4,614226762	2,88347E-11	1,52304E-10
KRT8	1,244079697	10,63741047	2,88844E-11	1,52448E-10
LIG1	0,687313588	5,228141071	2,91195E-11	1,5357E-10
APRT	0,886448039	7,133223083	2,91584E-11	1,53656E-10
LMNB2	0,984047376	6,500181153	2,9326E-11	1,5442E-10

NPM1	0,803023451	9,867914222	2,97266E-11	1,56409E-10
ZDHHC3	-0,684490216	7,191183122	3,06494E-11	1,6114E-10
HNRPDL	-0,394463432	7,847817846	3,14165E-11	1,65045E-10
C4orf46	0,878939997	3,977636154	3,17976E-11	1,66919E-10
GORAB	0,815564632	4,455612725	3,20594E-11	1,6813E-10
COPA	0,624796092	8,822097438	3,20776E-11	1,6813E-10
NFYA	0,654760435	5,946446536	3,21884E-11	1,68581E-10
NOP58	0,556433437	6,434404616	3,24003E-11	1,69561E-10
ELOVL6	1,764063639	4,514252846	3,3273E-11	1,73994E-10
ARRDC2	-0,783645693	6,036015779	3,33147E-11	1,74079E-10
SHC1	0,8433904	8,048754531	3,4573E-11	1,80515E-10
GNA11	-0,719460392	5,236887161	3,46241E-11	1,80644E-10
TXNIP	-0,944732828	10,29198231	3,47415E-11	1,81118E-10
YARS	0,529077407	7,28354822	3,48234E-11	1,81406E-10
SDHC	0,666962675	7,548338085	3,50027E-11	1,82201E-10
MRPL38	0,625062249	5,663712626	3,51965E-11	1,8307E-10
SLC5A3	1,243054908	6,222507934	3,64085E-11	1,8923E-10
RPS6KA2	-1,018716916	7,502135488	3,67849E-11	1,9104E-10
KPNB1	0,659458785	8,355702575	3,69326E-11	1,91662E-10
USP25	-0,612713376	6,228726027	3,7016E-11	1,91948E-10
SNX30	-1,178267274	6,946902798	3,70788E-11	1,92128E-10
ABCA1	-0,931737109	6,64774243	3,75386E-11	1,94363E-10
SERPINE2	2,367502335	6,182716801	3,88992E-11	2,01255E-10
UBL4A	0,715947153	6,55916313	3,93799E-11	2,03587E-10
RNF11	-0,53177562	7,285634087	3,9523E-11	2,04073E-10
CENPN	0,867016599	4,784147921	3,95337E-11	2,04073E-10
DCN	-1,172507294	9,738806621	3,97026E-11	2,0479E-10
RPAP3	0,499392853	5,236230175	3,99494E-11	2,05907E-10
DYNC1LI2	-0,53156877	7,543746463	4,10488E-11	2,11414E-10
TMEM147	1,0041584	6,907975922	4,16325E-11	2,14258E-10
FOXF2	-1,214599765	3,731109058	4,38286E-11	2,2539E-10
ATXN7L3	0,566679799	6,638160584	4,47139E-11	2,2977E-10
YY1	0,470924686	6,6272037	4,49103E-11	2,30605E-10
PPAPDC1B	1,217128036	6,146645055	4,5129E-11	2,31553E-10
C11orf9	-1,771184368	6,3818002	4,62028E-11	2,36885E-10
TMEM2	-0,889232542	7,425456141	4,64628E-11	2,38039E-10
ENC1	1,020271033	6,805775848	4,73879E-11	2,42596E-10
EED	0,634363334	4,745936256	4,77E-11	2,4401E-10
NEK1	-0,652487467	4,323181644	4,80373E-11	2,45552E-10
C5orf22	0,652926964	5,619558523	4,80778E-11	2,45575E-10
KIDINS220	-0,664471196	7,090285457	4,96755E-11	2,53546E-10
SEPHS2	0,870187261	7,220218423	4,99487E-11	2,54749E-10
FARP1	-0,742284145	7,281026345	5,04769E-11	2,57251E-10
EDEM1	-0,707799172	7,568746455	5,08779E-11	2,59101E-10
DHX37	0,65634301	5,115555489	5,33692E-11	2,71586E-10
PRKAR1A	-0,538939317	9,015207786	5,42077E-11	2,75647E-10
BAIAP2L1	0,913956244	6,818362125	5,51168E-11	2,80061E-10
MRPS35	1,03235572	6,761804283	5,64213E-11	2,86476E-10
RBL2	-0,615207215	7,198678635	5,67199E-11	2,87777E-10
CD2AP	0,752895822	7,460560041	5,90766E-11	2,99512E-10

LAMA4	-0,997906038	6,75119118	5,92724E-11	3,00281E-10
PTPN12	-0,499412621	7,373900791	5,96477E-11	3,01958E-10
RNF181	0,967453058	7,01090192	5,9734E-11	3,02171E-10
MCL1	-0,644693466	9,669690284	6,0549E-11	3,06066E-10
SSBP1	0,701606822	6,431909384	6,06491E-11	3,06345E-10
NPLOC4	0,494642631	7,689096884	6,17788E-11	3,1182E-10
F8A1	0,707642634	4,544249274	6,18814E-11	3,12107E-10
ING3	-0,505609706	4,182852417	6,30664E-11	3,17849E-10
TRMT2A	0,594129602	5,310534002	6,39594E-11	3,22112E-10
GAPVD1	-0,557318209	6,398163827	6,44713E-11	3,2445E-10
SF3B14	0,664558133	6,46011153	6,46736E-11	3,25228E-10
XYLT2	0,659593345	5,833671768	6,6439E-11	3,3386E-10
RNF13	-0,644731781	7,12532198	6,69292E-11	3,36075E-10
ING5	-0,545207082	4,719889482	6,80767E-11	3,41586E-10
NONO	0,584458799	8,918441919	7,08921E-11	3,55451E-10
NCAPD2	1,027282377	6,789533944	7,11514E-11	3,56489E-10
PSME3	0,513663585	7,394736939	7,12462E-11	3,56702E-10
DKK3	-0,968811906	7,126365655	7,4134E-11	3,70888E-10
GPATCH2	0,56554714	4,322297069	7,83196E-11	3,91541E-10
APH1A	0,543634281	8,555932535	8,1139E-11	4,05339E-10
GGH	1,843742078	5,559902432	8,14828E-11	4,06758E-10
C16orf13	0,850737492	5,890527968	8,20714E-11	4,09396E-10
ALG1	0,506803748	5,995804665	8,31177E-11	4,14313E-10
HSDL2	-0,737336366	6,473074467	8,75016E-11	4,35846E-10
BICD2	-0,684035256	5,870167374	8,95663E-11	4,45805E-10
OST4	0,872096268	7,781601056	9,07605E-11	4,51419E-10
VDAC1	0,685414707	8,029264324	9,24887E-11	4,5968E-10
ENTPD6	0,711236823	6,972208285	9,31963E-11	4,62859E-10
SRP9	0,727737026	8,623267195	9,35499E-11	4,64277E-10
POLR2J	0,801206883	6,028212088	9,48073E-11	4,70175E-10
PGAM1	0,663435532	8,597706247	9,7167E-11	4,81528E-10
CDC34	0,745374764	5,99425866	9,72852E-11	4,81763E-10
ADSS	0,544832627	6,726954975	9,81045E-11	4,85468E-10
CSF2RB	-1,011230266	5,432087857	9,99799E-11	4,94389E-10
WDR74	0,639564011	5,498670636	1,00448E-10	4,96343E-10
DDX11	1,060506314	5,047715014	1,02687E-10	5,07038E-10
TPD52	0,826218144	7,317537868	1,05286E-10	5,19498E-10
RBM34	0,465520989	5,233780759	1,14054E-10	5,62353E-10
URB1	0,777887255	6,034701281	1,14477E-10	5,64029E-10
C17orf70	0,538945782	5,71183759	1,14594E-10	5,642E-10
UNC119	0,612880434	5,070215241	1,1478E-10	5,64706E-10
GBA	0,677127489	7,14088116	1,15101E-10	5,65878E-10
PIGM	0,6468125	5,292619692	1,15491E-10	5,67386E-10
ITGAV	0,885979131	8,07338421	1,22638E-10	6,02066E-10
TMEM14A	0,864242758	5,305968935	1,2301E-10	6,03458E-10
ZNF662	-0,920416942	3,35400285	1,23568E-10	6,0576E-10
NDUFS1	0,544478855	6,579726878	1,28694E-10	6,30433E-10
DCTN1	-0,504615524	7,89650814	1,2896E-10	6,31227E-10
HEBP1	-0,582773793	5,981767321	1,29041E-10	6,31227E-10
RFXAP	-0,586291564	2,801085138	1,29228E-10	6,3169E-10

XRCC5	0,410784735	8,739652067	1,30607E-10	6,37971E-10
CAD	0,875628201	6,199084401	1,30967E-10	6,39275E-10
LCMT1	0,511274935	5,355290018	1,33097E-10	6,49207E-10
NCAPD3	0,825463991	5,279106689	1,341E-10	6,5363E-10
RHOA	-0,456705336	9,82641487	1,3486E-10	6,56862E-10
FAM103A1	0,623510428	5,296720783	1,35695E-10	6,60458E-10
CRBN	-0,702919762	5,539478915	1,36066E-10	6,61794E-10
MAGOHB	0,833434657	4,437531841	1,36649E-10	6,63823E-10
TMEM54	0,931674688	6,490439695	1,36678E-10	6,63823E-10
AKT3	-1,034898594	5,021838583	1,38964E-10	6,74444E-10
CFB	1,680612279	8,467158812	1,42605E-10	6,91296E-10
ZFAND5	-0,61737691	8,408107321	1,42639E-10	6,91296E-10
BDH2	-0,78983011	5,608542302	1,45001E-10	7,02247E-10
RPL36A	0,889024963	9,181429489	1,4721E-10	7,12436E-10
STYXL1	0,816913306	5,692243358	1,52462E-10	7,37334E-10
B4GALT4	0,968019775	5,517139806	1,52762E-10	7,38257E-10
DUSP3	-0,557356362	7,275682596	1,5404E-10	7,43909E-10
CHCHD2	0,934791236	8,798248666	1,55808E-10	7,51915E-10
C9orf116	-1,290314402	3,074996995	1,58238E-10	7,63101E-10
GFER	0,573308896	4,829691441	1,58412E-10	7,63398E-10
MBTPS2	0,524740102	5,252831066	1,59173E-10	7,66525E-10
WFDC2	1,722584522	8,84916087	1,60269E-10	7,71258E-10
HLTF	0,848875528	5,930197368	1,62841E-10	7,83083E-10
AK1	-1,096033394	7,113755474	1,62979E-10	7,83193E-10
C1QBP	0,826168364	6,713739432	1,68031E-10	8,06905E-10
HTT	-0,629355891	7,125047562	1,83033E-10	8,78328E-10
PPM1K	-0,696768308	4,646414356	1,84045E-10	8,82564E-10
SETD2	-0,550131603	6,471641593	1,89002E-10	9,05695E-10
PTMA	0,545392529	10,8487523	1,92444E-10	9,20915E-10
PFN2	1,510218016	7,073697925	1,92448E-10	9,20915E-10
MRPS7	0,660275058	6,624211055	1,94326E-10	9,29248E-10
PTPN14	-0,740196911	3,966361151	1,99004E-10	9,50953E-10
PIGT	0,659840553	7,926985561	2,00154E-10	9,55777E-10
GOLPH3L	0,706516304	6,302244789	2,06439E-10	9,85098E-10
PARVA	-0,532721662	6,776889546	2,12775E-10	1,01463E-09
RINT1	0,595016261	5,051184323	2,13328E-10	1,01655E-09
DUSP23	1,266936654	6,219893982	2,22033E-10	1,05729E-09
TEX2	-0,518975074	6,212046919	2,26702E-10	1,07877E-09
SH3GL1	0,552097332	6,69412726	2,27475E-10	1,0817E-09
PAAF1	0,633036036	4,757195514	2,31501E-10	1,10007E-09
PA2G4	0,62341237	7,753177288	2,33652E-10	1,10952E-09
NDUFB4	0,706975561	6,881025939	2,34696E-10	1,11371E-09
KATNAL1	-0,665943025	4,464390339	2,39494E-10	1,13568E-09
CLK2	0,636943248	5,512369878	2,4224E-10	1,1479E-09
SMG6	-0,598428136	5,994955736	2,45562E-10	1,16284E-09
ISCA2	0,810211684	4,992683174	2,52709E-10	1,19585E-09
TUBA4A	1,078503062	7,0190914	2,59659E-10	1,22789E-09
CDKN2C	1,018419722	4,31732423	2,60047E-10	1,22887E-09
ZC3HC1	0,52611405	4,507888698	2,62154E-10	1,23797E-09
NOL6	0,60788674	6,486662834	2,67354E-10	1,26165E-09

NUCB1	-0,500175203	9,027556644	2,69458E-10	1,2707E-09
CCDC134	0,586508796	2,211678584	2,80089E-10	1,31992E-09
CPOX	0,833502529	4,977656518	2,80965E-10	1,32314E-09
SNRPD2	0,909548519	7,641265371	2,82034E-10	1,32726E-09
C2orf49	0,641712296	3,438542418	2,99908E-10	1,41039E-09
EPB41L3	-1,103285654	5,698829986	3,00842E-10	1,41381E-09
PPP1CA	0,615698659	8,217706004	3,02048E-10	1,4185E-09
FAM76A	-0,540491756	4,434531833	3,02865E-10	1,42136E-09
MAD2L1BP	0,545184388	5,106658478	3,15885E-10	1,48144E-09
KCNK5	1,427736105	6,317562564	3,20977E-10	1,50429E-09
C10orf76	-0,381948515	5,658004991	3,22117E-10	1,5086E-09
HAPLN3	1,133552634	4,458795383	3,23133E-10	1,51231E-09
TDP1	0,737366662	4,346343016	3,2474E-10	1,51879E-09
POLR3E	0,470904633	5,463923709	3,28837E-10	1,5369E-09
AFF1	-0,658988311	6,950143938	3,34533E-10	1,56245E-09
SLC20A1	0,842812719	6,638474199	3,37311E-10	1,57435E-09
IL1R1	-0,845589958	7,811860007	3,38523E-10	1,57892E-09
TIPRL	0,64060169	6,402494492	3,46646E-10	1,61571E-09
SRRD	0,463442657	4,075324387	3,58551E-10	1,67006E-09
MRPL11	0,775347296	5,986023912	3,6278E-10	1,6886E-09
FBXO5	0,922105823	3,635981433	3,71025E-10	1,7258E-09
DOCK2	-1,000989844	5,870626149	3,72325E-10	1,73066E-09
RGS5	-1,025586085	7,412349776	3,76902E-10	1,75075E-09
SAAL1	0,63130661	3,621621137	3,89625E-10	1,80861E-09
ZC3H7A	-0,470378246	6,088928712	3,90313E-10	1,81057E-09
FNBP1	-0,64623869	6,473902211	3,91191E-10	1,81341E-09
OAS1	1,350607481	6,790441479	3,91838E-10	1,81412E-09
CTDSP1	-0,431416243	7,575470869	3,91875E-10	1,81412E-09
KLHDC7B	1,789172067	2,988699825	4,04034E-10	1,86898E-09
CDK7	0,66515106	5,19462385	4,04274E-10	1,86898E-09
SATB1	-1,008443135	5,240523123	4,11278E-10	1,90007E-09
ROMO1	1,02617683	6,39281079	4,11667E-10	1,90058E-09
UBAC2	0,563048264	6,742118599	4,15357E-10	1,91632E-09
ZC3H14	-0,461828079	6,169311823	4,3271E-10	1,99503E-09
TMEM165	0,823712095	7,552318505	4,35315E-10	2,00568E-09
NRF1	-0,323675216	4,613869736	4,40106E-10	2,02639E-09
TAF2	0,540075878	5,529186998	4,4281E-10	2,03746E-09
HSD17B10	0,587300294	6,363576711	4,52167E-10	2,07911E-09
CUL7	0,643701523	6,33520953	4,61735E-10	2,12168E-09
ARHGAP21	-0,633268196	6,836873138	4,64838E-10	2,1345E-09
TRIM47	0,921201779	5,971261459	4,81704E-10	2,21046E-09
NUDT3	0,463573016	5,279374022	4,89236E-10	2,24264E-09
KIAA1737	-0,488778882	5,567737331	4,89375E-10	2,24264E-09
C19orf10	0,827763232	7,493885057	4,9636E-10	2,27312E-09
PLEKHA6	1,422027429	5,917001473	5,03389E-10	2,30377E-09
AP1S2	-0,894733003	5,385560841	5,0796E-10	2,32312E-09
CRY1	-0,6806945	5,219260601	5,11028E-10	2,33559E-09
TUFM	0,544123644	8,150540142	5,12435E-10	2,33949E-09
SNRPF	0,778803654	5,805132696	5,12567E-10	2,33949E-09
PTS	0,907201362	4,448687213	5,17297E-10	2,3595E-09

CHST15	0,9131013	6,506470761	5,21784E-10	2,37837E-09
KRT18	1,100720093	10,13923946	5,30701E-10	2,4174E-09
WWTR1	-0,82793891	5,618820678	5,35685E-10	2,43848E-09
THOP1	0,652597098	5,284078839	5,36941E-10	2,44128E-09
OSBPL1A	-0,691225087	5,845073781	5,37016E-10	2,44128E-09
NIT2	0,794927411	5,711226086	5,39325E-10	2,45014E-09
LRFN4	1,263415122	4,528203557	5,47431E-10	2,48531E-09
KIAA0232	-0,590507913	6,253455399	5,7821E-10	2,6233E-09
WAPAL	-0,416752001	6,482631503	5,81041E-10	2,63439E-09
NR2F6	0,714258052	6,122289559	5,82414E-10	2,63886E-09
BET1	0,961461135	5,70857408	5,85729E-10	2,65212E-09
EVC	-0,824211582	5,211478375	5,87626E-10	2,65894E-09
RGPD8	-0,633441464	3,484568288	5,92822E-10	2,68067E-09
SLFN13	0,960383174	5,848081346	6,02684E-10	2,72346E-09
CCDC82	-0,49320981	4,995236985	6,05367E-10	2,73377E-09
GTPBP4	0,675384611	6,196575481	6,08203E-10	2,74476E-09
DDX3X	-0,539109371	8,770237191	6,09679E-10	2,7496E-09
NFIA	-0,994033011	5,705203139	6,2866E-10	2,83333E-09
RPAP2	0,617001074	3,931867643	6,30922E-10	2,84165E-09
CDC123	0,535147124	6,331266584	6,47212E-10	2,91309E-09
TMEM159	0,934678307	6,48366229	6,55798E-10	2,94979E-09
FAM45A	0,491135454	5,559338325	6,59819E-10	2,96406E-09
ZBTB40	-0,664528115	5,403181007	6,59838E-10	2,96406E-09
C7orf23	-0,883618382	5,596527809	6,70506E-10	3,01E-09
OSTF1	-0,505256734	6,201275353	6,80946E-10	3,05485E-09
FAM120B	-0,500492071	5,2873514	6,81901E-10	3,05712E-09
MRPL21	0,96700502	5,522479744	6,87567E-10	3,0805E-09
HIST1H2AC	1,2781839	6,28007009	6,94844E-10	3,11106E-09
NIP7	0,63334335	5,581811079	7,12062E-10	3,18606E-09
RUVBL1	0,691623103	6,229285222	7,2387E-10	3,23677E-09
MTHFD1	0,58657697	6,541416574	7,25288E-10	3,24098E-09
ZNF692	0,840305469	4,541489678	7,26407E-10	3,24386E-09
LYSMD1	0,722587557	4,215273315	7,33517E-10	3,27346E-09
NUPL2	0,568927605	4,952890427	7,35405E-10	3,27974E-09
ATP5H	0,669886401	7,456085764	7,38788E-10	3,29268E-09
FASN	-1,035164874	8,763559136	7,39642E-10	3,29433E-09
IP6K1	-0,413670911	6,225074615	7,51093E-10	3,34315E-09
LRPPRC	0,570135876	7,700296333	7,51655E-10	3,34347E-09
FZD5	-0,841200603	6,131324076	7,55579E-10	3,35873E-09
IFNGR2	0,748080788	7,592072289	7,62574E-10	3,38692E-09
TFDP1	0,650166443	6,760791553	7,62914E-10	3,38692E-09
SEMA4C	0,675013206	6,263221334	7,73153E-10	3,43014E-09
LRP1	-0,955147298	9,257416124	7,74557E-10	3,43414E-09
NXN	-0,722279064	6,538018425	7,7918E-10	3,45239E-09
DONSON	0,640131299	5,323831873	7,80156E-10	3,45447E-09
ITFG2	-0,574143891	4,816587677	7,86367E-10	3,47971E-09
HIPK3	-0,7740904	6,205065686	8,01223E-10	3,54315E-09
GTF2IRD1	0,644770348	5,362546711	8,10554E-10	3,58209E-09
ABLIM1	-1,109932671	7,926981641	8,13075E-10	3,59091E-09
PFDN4	0,969417351	4,934911478	8,15704E-10	3,60018E-09

TCEA1	0,62310523	7,845863622	8,19287E-10	3,61366E-09
MSN	-0,691328799	10,1773578	8,45382E-10	3,72635E-09
ECM1	1,862631314	6,726066872	8,6991E-10	3,83199E-09
S100A16	1,37794331	8,112274442	8,75549E-10	3,85434E-09
RCOR1	-0,609237164	6,190228951	8,87194E-10	3,90308E-09
MCM5	0,697858641	6,183307618	8,88021E-10	3,9042E-09
ARFGAP1	0,501376876	6,592584461	9,08758E-10	3,9928E-09
ATP2B4	-0,811692494	8,135825697	9,16556E-10	4,02446E-09
FXR2	-0,517515145	5,798164458	9,25484E-10	4,06105E-09
TP53BP2	-0,602688449	6,567858166	9,38696E-10	4,11638E-09
LEPREL2	1,168199704	4,919646443	9,44533E-10	4,13931E-09
TSPYL2	-0,786215217	5,324139915	9,72075E-10	4,25728E-09
STAU2	0,584778967	6,186364927	9,85628E-10	4,31386E-09
EIF1B	-0,512034918	5,64562656	9,87794E-10	4,31983E-09
CLPTM1L	0,941204057	8,458919902	9,88258E-10	4,31983E-09
TDG	0,710878887	5,573982669	9,95206E-10	4,34741E-09
C22orf13	-0,516686357	7,232955448	1,01675E-09	4,43868E-09
SGK3	-0,509560728	5,179381402	1,03344E-09	4,50867E-09
KARS	0,46232765	7,57356248	1,05484E-09	4,59909E-09
TEAD1	-0,667995189	6,937854887	1,08009E-09	4,70614E-09
FGFR1OP	0,65036387	3,535744176	1,08662E-09	4,73157E-09
NT5C3L	0,797954306	5,939117722	1,08792E-09	4,73423E-09
FHL3	0,892208379	4,916185426	1,09874E-09	4,77828E-09
POLR3D	0,560633461	4,648653921	1,10332E-09	4,79511E-09
HMGB2	0,922438317	6,825416541	1,12383E-09	4,88113E-09
SNX9	0,695347391	6,609363877	1,14658E-09	4,97679E-09
LMAN2	0,627026071	8,164349465	1,15947E-09	5,02952E-09
SLC2A3	-1,106498265	6,996191394	1,17284E-09	5,08428E-09
CSNK2B	0,512435146	7,442917949	1,19277E-09	5,16742E-09
SENP6	-0,448818182	6,449162275	1,21517E-09	5,26112E-09
C3orf14	0,946368408	4,009813469	1,22692E-09	5,30863E-09
TSPAN4	-0,831963071	6,675305024	1,23642E-09	5,34633E-09
SLC38A7	0,651375418	4,234117076	1,27375E-09	5,50427E-09
QPCTL	0,61286859	4,673197513	1,28789E-09	5,56182E-09
COPB1	0,44146784	7,941151758	1,29875E-09	5,60519E-09
AHNAK2	1,731639343	7,086230522	1,31588E-09	5,67552E-09
CYBB	-1,169406254	7,875542001	1,32491E-09	5,71086E-09
ZNF581	0,80939395	4,711966856	1,32746E-09	5,71821E-09
BHLHE41	-1,006406033	5,299310459	1,34214E-09	5,77782E-09
TMEM183A	0,74263254	7,083136981	1,34784E-09	5,7987E-09
SSRP1	0,50277447	7,823039209	1,38574E-09	5,95798E-09
CLIC4	-0,659243777	8,417205158	1,3905E-09	5,9747E-09
UMPS	0,522473883	5,355195888	1,39331E-09	5,98297E-09
NPIP	-0,786954686	4,824300131	1,40511E-09	6,02984E-09
TSC1	-0,565851129	5,506412397	1,48684E-09	6,37656E-09
SDC2	-0,95611636	6,552391195	1,49636E-09	6,41339E-09
INPP4B	1,368313527	4,272864078	1,49829E-09	6,41761E-09
BANF1	0,676639582	7,525168964	1,53874E-09	6,58672E-09
XRCC2	0,789121953	2,888978323	1,54906E-09	6,62674E-09
ENPP2	-0,994270848	6,67115039	1,6238E-09	6,94213E-09

TBCE	0,639730878	5,010766558	1,63344E-09	6,97896E-09
CRIPT	0,84473549	5,114966502	1,64911E-09	7,04152E-09
RSRC1	0,645736548	4,42679171	1,71375E-09	7,31293E-09
PSMD2	0,623333501	8,351751982	1,72434E-09	7,35352E-09
CCT2	1,467436518	8,266945713	1,76903E-09	7,53937E-09
FBXL20	0,709558274	3,828747543	1,79084E-09	7,62758E-09
PLXNA2	-1,107270731	6,712129741	1,81963E-09	7,74534E-09
TRUB1	0,592239954	5,245948906	1,83065E-09	7,78741E-09
MORC3	-0,412940749	5,517842495	1,85612E-09	7,89084E-09
ROCK1	-0,569525356	6,713646043	1,8658E-09	7,92702E-09
SNAPIN	0,703538586	5,948964329	1,87448E-09	7,95895E-09
NUP88	0,582856269	5,718799364	1,87922E-09	7,96998E-09
KAT5	-0,36852554	6,053914781	1,87942E-09	7,96998E-09
SSR3	0,683909012	8,403436276	1,8822E-09	7,97682E-09
DDIT4	1,395771281	8,22415138	1,89284E-09	8,01693E-09
C1GALT1	0,926445448	5,351771737	1,89591E-09	8,02498E-09
NFXL1	0,58573697	4,057739384	1,92925E-09	8,15643E-09
SRP72	0,519260584	7,515576808	1,92936E-09	8,15643E-09
MEIS2	-0,868344324	4,170557611	1,9322E-09	8,16338E-09
DHX34	0,653252442	4,752892999	1,93405E-09	8,16613E-09
GOLT1B	0,940414908	6,713735272	1,96931E-09	8,30984E-09
TAF10	0,632638911	6,172755148	2,05065E-09	8,64774E-09
BMPR1A	-0,48091725	5,56262914	2,07497E-09	8,74489E-09
GGCX	0,846310199	6,639011376	2,12996E-09	8,97109E-09
C5orf28	0,752165379	4,699444976	2,13331E-09	8,97964E-09
ELMO3	0,946667485	5,667149605	2,13934E-09	8,99775E-09
MRPL27	0,718661778	5,800180781	2,14025E-09	8,99775E-09
NIPAL3	-0,686129942	6,190118185	2,16006E-09	9,07544E-09
HIBADH	0,60040216	6,232895379	2,17166E-09	9,11856E-09
FBL	0,651297486	7,201172842	2,1886E-09	9,18405E-09
ERI3	0,510075097	6,106991289	2,19695E-09	9,2134E-09
SFT2D2	0,681223732	3,061783406	2,27682E-09	9,54249E-09
RIN3	-0,70190536	5,795712082	2,32303E-09	9,72667E-09
SPATA20	0,793462102	6,341046236	2,32362E-09	9,72667E-09
FYCO1	-0,729196544	6,536342575	2,32709E-09	9,73523E-09
UBD	2,155148162	6,035027147	2,39363E-09	1,00024E-08
TMEM99	1,060358423	4,4747962	2,39389E-09	1,00024E-08
AAMP	0,411105048	7,27796896	2,41993E-09	1,0105E-08
NPEPPS	0,529517809	7,52899313	2,42636E-09	1,01257E-08
LAPTM5	-0,891001571	9,253141725	2,43898E-09	1,01721E-08
ADIPOR2	-0,667205349	6,990517697	2,53089E-09	1,0549E-08
RRS1	0,83350074	5,066945179	2,53526E-09	1,05608E-08
GSTA4	-0,994626622	5,410401967	2,5795E-09	1,07385E-08
ARPP19	-0,436907901	8,001789374	2,58928E-09	1,07726E-08
MRPS18A	0,615827015	5,782399781	2,65085E-09	1,1022E-08
VPS36	-0,515561426	6,151510922	2,72092E-09	1,13065E-08
SLC26A6	0,776554888	3,893482685	2,75256E-09	1,1431E-08
METTL5	0,653576	5,181177312	2,77929E-09	1,1535E-08
MRPS15	0,883044431	6,654335897	2,86908E-09	1,19004E-08
PPIA	0,671930121	10,60249852	2,87306E-09	1,19097E-08

RABGAP1	-0,503081654	6,322251319	2,88234E-09	1,19409E-08
GOT2	0,551898372	6,939134518	2,89628E-09	1,19913E-08
EPM2AIP1	-0,52592632	5,655310219	2,94476E-09	1,21847E-08
MLLT1	-0,605699087	6,74768339	2,9533E-09	1,22126E-08
EEF1E1	0,684313939	4,401343083	2,99546E-09	1,23794E-08
ADRM1	0,554805232	7,123319849	3,02826E-09	1,25074E-08
TMUB1	0,612894779	5,673396944	3,07841E-09	1,27028E-08
TMEM176B	1,216015359	7,470979281	3,07931E-09	1,27028E-08
STRBP	0,635131828	5,430072649	3,1479E-09	1,29779E-08
COPS6	0,593736377	7,036914694	3,17057E-09	1,30635E-08
CHPT1	-0,877035905	5,917992129	3,18522E-09	1,31159E-08
UBE2Z	0,400084207	7,566825284	3,22383E-09	1,32669E-08
MGC2752	-0,42182321	5,496549001	3,23439E-09	1,33024E-08
MED14	0,527363391	6,441419933	3,25363E-09	1,33734E-08
PHKG2	0,602093465	5,438812274	3,29797E-09	1,35475E-08
COPS8	0,498314484	6,134402766	3,33217E-09	1,36798E-08
IDI1	-0,809073671	6,801032975	3,44861E-09	1,41493E-08
RHBDD3	0,756796701	4,520050013	3,49291E-09	1,43224E-08
MORF4L2	0,602730326	8,308126695	3,50413E-09	1,43598E-08
SLC23A2	-0,70079127	5,626443829	3,51525E-09	1,43967E-08
RUFY3	-0,608138601	5,697728063	3,53274E-09	1,44596E-08
GPR56	1,152846407	7,649840762	3,58026E-09	1,46453E-08
HCK	-1,044724124	6,167271079	3,59224E-09	1,46855E-08
LOC550112	0,490544112	3,980395681	3,82025E-09	1,56083E-08
MRPS26	0,749787175	5,774803375	3,84738E-09	1,57097E-08
RPL8	0,877367809	11,29000328	3,86194E-09	1,57598E-08
IFI30	-0,882925535	8,295939242	3,92897E-09	1,60237E-08
RPS18	1,112261866	11,56374767	3,94153E-09	1,60653E-08
IDE	0,633039484	5,377511186	4,00794E-09	1,63263E-08
NDE1	0,628732603	5,131961004	4,04389E-09	1,64629E-08
MRPL44	0,507316848	5,511321579	4,07933E-09	1,65972E-08
EXOC6	-0,456121084	5,254670584	4,10086E-09	1,66749E-08
PINK1	-0,480985795	6,794311281	4,12956E-09	1,67816E-08
PDCL	-0,368669189	5,232298498	4,19397E-09	1,70332E-08
LAIR1	-0,799261751	6,319179644	4,20913E-09	1,70845E-08
CLTC	0,498028377	9,586711511	4,23585E-09	1,71828E-08
BIN2	-0,881329995	4,556251787	4,291E-09	1,73962E-08
GALNT4	1,164867678	5,852158798	4,29476E-09	1,74011E-08
SNX14	-0,479641832	6,517671643	4,32884E-09	1,75287E-08
DCTN5	0,507943483	6,637514137	4,40987E-09	1,78462E-08
B3GALNT2	0,588642228	5,470779652	4,48815E-09	1,81523E-08
STEAP2	1,160976701	5,587860541	4,49323E-09	1,8162E-08
FAM57A	0,776222784	5,104873833	4,52644E-09	1,82854E-08
ASAH1	-0,794785155	9,28386679	4,54677E-09	1,83567E-08
JUP	0,944980545	9,727607924	4,60607E-09	1,85851E-08
PRDX2	0,775823539	7,713253912	4,68474E-09	1,88913E-08
SSR2	0,684128801	8,931390205	4,74385E-09	1,91184E-08
MTF1	-0,532028879	5,211596396	4,76176E-09	1,91792E-08
CLIP4	-0,677809931	5,796620462	4,76733E-09	1,91903E-08
RGS10	1,004170012	5,577535153	4,80241E-09	1,93202E-08

GPX7	1,016546269	4,400929596	4,81694E-09	1,93672E-08
SAV1	-0,430972684	5,835889611	4,87695E-09	1,95969E-08
TSPAN13	-0,84227562	8,690927042	4,90238E-09	1,96875E-08
NR3C1	-0,591673438	7,17868447	4,94291E-09	1,98385E-08
USP21	0,576517459	5,270256516	4,98338E-09	1,99892E-08
LOC388796	0,859713713	4,572585249	4,99249E-09	2,0014E-08
U2AF2	0,39984155	7,490599349	5,05529E-09	2,02493E-08
JAK1	-0,531484555	8,001517913	5,05712E-09	2,02493E-08
ARHGAP17	-0,417530229	6,095476936	5,06331E-09	2,02622E-08
YIPF6	0,673209002	4,997070303	5,2237E-09	2,08917E-08
RANBP3	-0,352815775	6,17278278	5,24439E-09	2,09622E-08
RPL7L1	0,408662263	6,876934	5,25062E-09	2,09748E-08
RBBP7	0,453567146	7,169640679	5,27567E-09	2,10574E-08
EMB	1,176376135	7,107669833	5,27747E-09	2,10574E-08
SETDB1	0,534479583	6,063808231	5,37484E-09	2,14334E-08
KCTD5	0,679245775	6,060241107	5,43713E-09	2,16691E-08
MKNK1	-0,435947375	5,453377807	5,50099E-09	2,19108E-08
SLC25A22	0,642965719	5,238592759	5,51584E-09	2,19571E-08
PEBP1	-0,565545601	8,537142012	5,52994E-09	2,20004E-08
PRR3	0,587686662	4,053766224	5,54416E-09	2,20441E-08
MAPK13	0,728231702	6,975535301	5,56362E-09	2,21086E-08
MASTL	0,690225312	4,422135509	5,5859E-09	2,21842E-08
ZYX	-0,646876631	8,228848649	5,64093E-09	2,23784E-08
OAT	0,869375042	7,474112359	5,64136E-09	2,23784E-08
NOL7	0,407992216	5,898727422	5,64757E-09	2,239E-08
GAN	0,824724788	2,01885607	5,8045E-09	2,29988E-08
NUP210	1,018356509	6,878379481	5,82032E-09	2,30481E-08
SND1	0,459723111	8,371387991	5,83341E-09	2,30865E-08
WDR43	0,573261766	6,130287126	5,88485E-09	2,32765E-08
CHID1	0,579992938	6,931399022	5,9253E-09	2,3423E-08
MRPS6	1,057857522	6,380231605	5,98672E-09	2,36521E-08
LOC387647	-0,42383512	4,753092833	6,04538E-09	2,387E-08
TPD52L2	0,569826761	7,75858249	6,08412E-09	2,4009E-08
SOX13	-0,848391677	5,684012563	6,29599E-09	2,48266E-08
ADCY7	-0,736567884	6,398228611	6,29858E-09	2,48266E-08
POLR3C	0,490612943	5,400537685	6,59202E-09	2,59682E-08
TRMT6	0,578630423	4,986681996	6,62178E-09	2,60704E-08
NUP35	0,580752578	4,11136182	6,74601E-09	2,65374E-08
APPL1	-0,375470099	6,345739879	6,74818E-09	2,65374E-08
MUM1	-0,535318359	5,288299319	6,75727E-09	2,65578E-08
LSM4	0,678007361	6,812704412	6,79147E-09	2,66769E-08
GATC	0,705532403	2,266663636	6,79964E-09	2,66936E-08
TOB2	-0,542235562	6,883484194	6,92598E-09	2,71739E-08
TMEM231	-1,099874815	5,12045195	7,00228E-09	2,74575E-08
NRBP1	0,361034888	7,25630202	7,03455E-09	2,75682E-08
COPS7B	0,432551656	5,552845771	7,1481E-09	2,79971E-08
EIF2AK2	0,638476588	5,331841293	7,16173E-09	2,80343E-08
SUMO2	0,493931407	8,400714889	7,37911E-09	2,88586E-08
NDUFB11	0,595231612	6,826539484	7,38176E-09	2,88586E-08
EXOSC2	0,497146604	4,882699901	7,38498E-09	2,88586E-08

MARCH5	0,436508506	6,100530509	7,43332E-09	2,90308E-08
ZNF160	-0,509943754	5,128742286	7,48762E-09	2,92261E-08
ZC3HAV1	-0,456615611	6,442589234	7,57847E-09	2,95589E-08
USP14	0,449485178	6,580493412	7,58155E-09	2,95589E-08
ITGA2	1,498723705	7,109593692	7,61587E-09	2,96758E-08
TFRC	-1,019340775	8,213664389	7,64202E-09	2,97607E-08
C16orf52	-0,476898768	4,3840558	7,70091E-09	2,99729E-08
EEF1G	0,620251624	11,14774768	7,79134E-09	3,03076E-08
C7orf13	1,062054008	3,63128504	7,79612E-09	3,03089E-08
MPRIP	-0,647344343	7,341812018	7,86884E-09	3,05741E-08
SLC41A2	0,791966529	5,196294048	7,94034E-09	3,08344E-08
FTSJ2	0,485062612	5,560813788	8,09164E-09	3,14041E-08
NACC1	0,679887228	6,827246256	8,12369E-09	3,15105E-08
RASSF3	-0,761845176	4,704773286	8,30428E-09	3,21927E-08
DPAGT1	0,503656468	5,983680443	8,39121E-09	3,25112E-08
ATF5	0,977955256	6,201082618	8,46755E-09	3,27883E-08
ATP5G3	0,678275155	7,779453894	8,5492E-09	3,30857E-08
PDIK1L	0,686585132	4,717200784	8,83474E-09	3,41699E-08
ETV1	-1,099339445	6,344727996	8,83937E-09	3,41699E-08
RGPD6	-0,598836446	5,184728163	8,89209E-09	3,43542E-08
KLF11	-0,614681284	5,578701031	9,00037E-09	3,47451E-08
ZDHHC4	0,538513424	6,315459761	9,00347E-09	3,47451E-08
HDAC2	0,589319849	7,048139198	9,22482E-09	3,55792E-08
UBA7	-0,646519123	6,300092023	9,28869E-09	3,57943E-08
SRP54	0,699445999	6,782667709	9,29109E-09	3,57943E-08
POLE3	0,598478861	6,74589626	9,54997E-09	3,67709E-08
RNF187	0,459495618	7,388510358	9,67529E-09	3,72324E-08
CKAP5	0,699198113	7,05350753	9,83312E-09	3,78184E-08
TRPM2	1,216465712	4,303591495	1,00289E-08	3,85497E-08
FAF1	0,378246953	6,0102261	1,00768E-08	3,87118E-08
CSE1L	0,662144495	6,97512076	1,00834E-08	3,87154E-08
KIAA0913	-0,478962177	6,557808288	1,01E-08	3,87573E-08
RPLP0	0,857480107	11,3646215	1,01275E-08	3,8841E-08
MBOAT7	0,582524775	6,652604252	1,01346E-08	3,88464E-08
TYROBP	-1,011374186	7,382225902	1,04284E-08	3,99501E-08
TSEN34	0,529023892	6,150606136	1,05598E-08	4,04308E-08
RPL26L1	0,7647003	5,113214193	1,06666E-08	4,08168E-08
METTL9	0,551074494	7,192390077	1,07343E-08	4,1053E-08
PRKDC	0,868348026	8,020318711	1,07473E-08	4,10796E-08
C8orf59	0,798005161	5,746166337	1,07648E-08	4,11233E-08
HGS	0,474497543	7,120141686	1,08E-08	4,12349E-08
NLRP1	-0,873817611	4,997358828	1,09773E-08	4,18677E-08
GTPBP1	-0,483160716	6,526074924	1,09781E-08	4,18677E-08
LIN9	0,701127011	3,682359301	1,10995E-08	4,23071E-08
FAM104A	0,358405961	5,510347966	1,11374E-08	4,24278E-08
FARSA	0,448145578	6,144339398	1,12346E-08	4,27741E-08
THUMPD3	0,346439573	5,735770177	1,15452E-08	4,39324E-08
YIPF3	0,480375751	7,786658713	1,15571E-08	4,3953E-08
FAM89B	0,565769104	6,266172116	1,15879E-08	4,40455E-08
FCGR3A	-1,048428017	8,045413365	1,16938E-08	4,44235E-08

BATF	1,14289954	4,099658051	1,17327E-08	4,45463E-08
DYNC2LI1	-0,526948633	4,724113482	1,1766E-08	4,46481E-08
ELMOD2	0,517301685	4,120600577	1,18619E-08	4,49867E-08
ARF1	0,449066962	9,77375579	1,21931E-08	4,6217E-08
KLHL23	0,942805968	3,677783653	1,22698E-08	4,64822E-08
MECP2	-0,534727966	6,384347449	1,23106E-08	4,66107E-08
TOPBP1	0,680260182	5,757909016	1,23577E-08	4,6763E-08
SEL1L3	0,69857911	8,063302329	1,24083E-08	4,69285E-08
PLAT	2,10623509	7,879178358	1,24179E-08	4,6939E-08
HSPA14	0,698645902	5,234554881	1,27418E-08	4,81363E-08
HMGXB4	0,456745759	5,365280823	1,27891E-08	4,82883E-08
CCL2	-1,251894963	6,664848331	1,28008E-08	4,83059E-08
MCCC2	0,576427029	6,682723445	1,29806E-08	4,89574E-08
PDLIM4	1,62804915	5,091825957	1,30398E-08	4,91532E-08
MAPKBP1	-0,617527433	4,795358004	1,3054E-08	4,91798E-08
DRAP1	0,670295233	6,627387037	1,32235E-08	4,97909E-08
MAN2A2	-0,581016074	6,321681194	1,33541E-08	5,02549E-08
EIF5A	0,647948537	9,103910493	1,34815E-08	5,07063E-08
FGD6	0,836731972	6,134271077	1,35509E-08	5,09392E-08
MINPP1	0,547412189	5,215824675	1,36346E-08	5,12255E-08
ZYG11B	-0,375460746	6,00629716	1,37326E-08	5,15653E-08
KDM4C	-0,434453204	5,178015999	1,37641E-08	5,16483E-08
CBL	-0,629304134	5,754190652	1,37699E-08	5,16483E-08
CRTAP	-0,50756483	7,992027689	1,38433E-08	5,18954E-08
ZHX2	-0,543436808	5,635463267	1,38852E-08	5,20238E-08
MFSD3	0,860054082	4,367261926	1,3926E-08	5,2148E-08
ATG2B	-0,570870574	5,557040106	1,41061E-08	5,27933E-08
MESDC1	-0,601535661	5,436429512	1,41651E-08	5,29852E-08
MTERFD1	0,687748724	4,552118686	1,41802E-08	5,30125E-08
C19orf24	0,649088542	5,322790062	1,42131E-08	5,31065E-08
RTEL1	0,895931702	5,148810089	1,42556E-08	5,32359E-08
AKIRIN2	-0,422728152	5,86949479	1,49922E-08	5,5956E-08
KPNA3	-0,43771248	6,479074634	1,52169E-08	5,67638E-08
NUDT16L1	0,677574075	5,455794779	1,53616E-08	5,7272E-08
NOP14	0,410670115	6,061193917	1,55549E-08	5,79612E-08
FBLIM1	0,68486127	6,541381959	1,56829E-08	5,84063E-08
RNF115	0,558513085	5,262783959	1,61704E-08	6,01889E-08
OLFML2A	-0,998072679	5,592338025	1,63067E-08	6,06629E-08
CYCS	0,719153373	7,799024884	1,63183E-08	6,0673E-08
DDX23	0,463434105	7,552231256	1,65569E-08	6,15267E-08
CCDC93	-0,485411158	5,588066374	1,67458E-08	6,21949E-08
ATP5G1	0,794524783	6,150707162	1,69841E-08	6,30453E-08
ARGLU1	-0,767209925	6,734849492	1,70943E-08	6,34202E-08
NOC3L	0,503214521	4,803654208	1,73954E-08	6,44676E-08
MRPL51	0,678850488	6,795293139	1,73956E-08	6,44676E-08
JAK3	0,762393255	5,127059399	1,74585E-08	6,46658E-08
TAF12	0,526120064	5,036329333	1,75526E-08	6,49788E-08
VEZF1	-0,448533907	6,822894856	1,76995E-08	6,54871E-08
CTBP2	0,431690201	6,995904355	1,80325E-08	6,6683E-08
INPP5B	-0,693443993	5,572582818	1,84361E-08	6,81388E-08

MRPL28	0,514132116	6,078823297	1,85387E-08	6,84807E-08
RHEB	0,790109557	7,620053627	1,87887E-08	6,93666E-08
OLFML2B	1,106796	5,579071341	1,89244E-08	6,98297E-08
PRDX3	0,698457996	7,872826228	1,90077E-08	7,00993E-08
TCEB3	-0,457949394	6,890115648	1,91481E-08	7,0579E-08
ATP2A2	0,588358083	9,012881673	1,92289E-08	7,08383E-08
METTL2B	0,548641564	5,009641204	1,95025E-08	7,18076E-08
SIK3	-0,537174353	5,792329556	1,95837E-08	7,20677E-08
AURKAIP1	0,708914546	6,587590924	1,96036E-08	7,21021E-08
ANKMY1	-0,666795995	4,094697149	1,98348E-08	7,29131E-08
ATP5J2	0,907342733	7,256434489	1,98761E-08	7,30254E-08
FOXO3B	-0,630274182	3,488786786	1,99908E-08	7,34071E-08
TPMT	0,511015539	6,054141718	2,0352E-08	7,46933E-08
SLC33A1	0,449043189	5,69763038	2,03865E-08	7,47799E-08
SLC1A5	0,668517738	7,833216998	2,06665E-08	7,57662E-08
TSPAN5	1,101504828	3,522375417	2,0851E-08	7,64015E-08
USP53	-0,619473349	5,911432613	2,09376E-08	7,66776E-08
MRPL4	0,542233625	5,941144367	2,18124E-08	7,98383E-08
ZNF136	-0,432601073	3,922738964	2,19177E-08	8,01808E-08
HAUS2	-0,346137493	5,550025811	2,20502E-08	8,06223E-08
FADS3	-0,774581001	5,106420479	2,22656E-08	8,13661E-08
TMEM134	0,679262548	5,556943519	2,22996E-08	8,14468E-08
PLEKHM2	-0,388069414	6,804829586	2,26517E-08	8,26815E-08
DICER1	-0,551487829	6,352614382	2,26619E-08	8,26815E-08
E2F6	0,530491552	4,912285934	2,32796E-08	8,48895E-08
UBE2W	0,711448058	6,14447458	2,34468E-08	8,54535E-08
ATP6V1B2	-0,515246512	7,57160075	2,34682E-08	8,5486E-08
PMS2CL	0,515603407	3,564050057	2,35989E-08	8,5916E-08
HSF1	0,492580428	6,905575828	2,44677E-08	8,90317E-08
MGAT2	0,596776067	6,142993416	2,45039E-08	8,91156E-08
ERH	0,636331313	7,211590429	2,47175E-08	8,98446E-08
KLHL15	-0,515542146	4,107223367	2,48665E-08	9,0338E-08
ZNF654	-0,430973181	4,381505952	2,49065E-08	9,04354E-08
SYNJ1	-0,564879532	4,527263846	2,56495E-08	9,30834E-08
NBEAL1	-0,863624903	4,038402565	2,5846E-08	9,37466E-08
CAMKK2	-0,388177301	6,350162741	2,5905E-08	9,39109E-08
VPS45	0,50054194	5,88558533	2,61901E-08	9,48938E-08
COQ6	0,50615445	4,593919873	2,62956E-08	9,52256E-08
IDH3G	0,491716153	6,233359771	2,65684E-08	9,61623E-08
SRP68	0,393414167	7,290786186	2,68336E-08	9,70707E-08
UBTD1	-0,680990296	5,658692323	2,70676E-08	9,78652E-08
WDR35	-0,621517289	4,640855709	2,77483E-08	1,00273E-07
PSMC6	0,639496154	6,972544952	2,78895E-08	1,00723E-07
SAT2	-0,585142544	5,58768666	2,79023E-08	1,00723E-07
ZNF259	0,458093191	5,83603598	2,8161E-08	1,01575E-07
ECHS1	0,55353387	7,369081442	2,8168E-08	1,01575E-07
ZNF264	-0,514723544	5,730910597	2,87191E-08	1,03507E-07
ACAT1	-0,642356015	6,318497838	2,88727E-08	1,04006E-07
PCNX	-0,555015218	6,228890469	2,90166E-08	1,04469E-07
GPSM2	0,594530299	5,027381765	2,97119E-08	1,06916E-07

TMEM66	-0,507750087	8,659553765	3,01135E-08	1,08265E-07
YTHDF1	0,438517721	6,669999686	3,01187E-08	1,08265E-07
PSMA6	0,959352191	7,890690862	3,03338E-08	1,08981E-07
MRPL47	0,637930812	5,450739263	3,04702E-08	1,0938E-07
ZNF623	0,589829012	4,703086131	3,0477E-08	1,0938E-07
FBF1	0,464965428	5,110301868	3,06933E-08	1,10099E-07
ATXN2L	0,449706929	7,463430515	3,08435E-08	1,10579E-07
PLCB2	-0,877470742	5,36823042	3,1359E-08	1,12368E-07
RAB28	-0,436866251	4,532735553	3,14981E-08	1,12807E-07
RAD51C	0,497520764	4,063506756	3,15853E-08	1,1306E-07
KIAA1522	0,658622934	7,911886698	3,19086E-08	1,14158E-07
ACTR3	0,507442398	8,551840482	3,24367E-08	1,15986E-07
PHTF2	0,586781974	5,583665395	3,28377E-08	1,17335E-07
RPL39	1,000822362	10,23483405	3,28482E-08	1,17335E-07
COX6B1	0,914374844	7,875147453	3,29892E-08	1,17777E-07
SWAP70	-0,576737316	7,042394776	3,33584E-08	1,19033E-07
ZNFX1	-0,553784946	7,158875003	3,34477E-08	1,19289E-07
CBFB	0,361192085	6,357832715	3,37187E-08	1,20192E-07
LUZP1	-0,596628308	5,872699803	3,42367E-08	1,21975E-07
BRMS1	0,466496289	6,015916591	3,48519E-08	1,24102E-07
COX5B	0,825745031	7,164932625	3,51737E-08	1,25182E-07
RASSF5	-0,735226568	6,217579231	3,5956E-08	1,279E-07
ZFPL1	0,432765789	5,382463585	3,62603E-08	1,28915E-07
SYVN1	0,520060143	6,968674093	3,64246E-08	1,29432E-07
UTP15	0,42799506	4,147825919	3,69208E-08	1,31126E-07
MYO1E	0,852664638	6,542204278	3,74263E-08	1,32853E-07
TIGD2	0,812717717	3,624756438	3,83019E-08	1,3589E-07
SNX29	-0,57066007	5,488117397	3,86015E-08	1,36882E-07
NAE1	0,441587659	5,742298881	3,90666E-08	1,38459E-07
PSMC2	0,530658397	7,0272188	3,91967E-08	1,38848E-07
TMSB10	0,932528912	11,12448912	3,96333E-08	1,40322E-07
RAB2A	0,407877853	6,676147168	4,032E-08	1,42679E-07
HMG20A	-0,319942442	5,597759419	4,04344E-08	1,43009E-07
ZRSR2	-0,546065256	4,033146607	4,25115E-08	1,50278E-07
TIPIN	0,574422194	2,747629881	4,28695E-08	1,51465E-07
LRIG1	-0,727335223	7,042419567	4,4367E-08	1,56674E-07
TMEM14B	0,823493619	6,926595306	4,44952E-08	1,57046E-07
PGS1	-0,480659145	5,524407888	4,45356E-08	1,57066E-07
UBE2Q1	0,426491813	7,233948324	4,45469E-08	1,57066E-07
UQCRQ	0,865741149	7,161039504	4,52664E-08	1,5952E-07
NUP43	0,418353388	5,495736084	4,53131E-08	1,59602E-07
ACP1	0,482015962	6,895567385	4,60035E-08	1,6195E-07
MTG1	0,597460576	5,023302033	4,6142E-08	1,62354E-07
AKTIP	-0,485726296	5,57370469	4,66909E-08	1,64201E-07
SDF2	0,575182336	6,202889368	4,68094E-08	1,64533E-07
PSMD8	0,59956485	7,833506902	4,68735E-08	1,64673E-07
NAA15	0,480395101	6,18122273	4,71748E-08	1,65646E-07
ATP6V0D1	-0,485126071	7,544014118	4,74548E-08	1,66544E-07
TBL1XR1	0,439123525	7,587498968	4,86931E-08	1,70801E-07
UTP18	0,44344851	5,788903663	4,92258E-08	1,72581E-07

FBXL5	-0,556494931	7,453012968	4,96237E-08	1,73887E-07
SDAD1	0,357677448	5,929966728	5,08081E-08	1,77946E-07
EIF2B4	0,339773465	5,58327183	5,09551E-08	1,78369E-07
CHMP4B	0,427269261	8,09832188	5,13569E-08	1,79683E-07
KPNA5	-0,658507515	2,595896387	5,14806E-08	1,79933E-07
FN3KRP	0,497815566	5,939370739	5,1481E-08	1,79933E-07
NDUFS2	0,480355421	7,066011442	5,2782E-08	1,84385E-07
DDX49	0,539834809	5,693917045	5,32404E-08	1,85892E-07
CHORDC1	0,573963922	4,908671202	5,3864E-08	1,87973E-07
TMEM214	0,439130764	7,380391108	5,4907E-08	1,91515E-07
ISOC2	0,656199667	5,82460213	5,50555E-08	1,91934E-07
FEM1B	-0,394059694	6,619264081	5,67319E-08	1,97678E-07
RDBP	0,548201383	6,789757119	5,69231E-08	1,98242E-07
RNF2	0,497409379	5,236352706	5,697E-08	1,98304E-07
CECR5	0,550394787	5,389210812	5,81876E-08	2,02439E-07
ADSL	0,603478301	6,171688555	5,95464E-08	2,07061E-07
SLC39A14	0,954084258	6,298572385	5,96431E-08	2,07292E-07
RAB21	-0,408979283	6,264705027	5,98373E-08	2,07861E-07
LYRM7	-0,403182901	4,914886621	6,00154E-08	2,08373E-07
FAM168B	-0,505244428	7,247500438	6,05696E-08	2,1019E-07
CTPS2	0,536821988	5,344382207	6,07637E-08	2,10757E-07
PSD3	0,767906049	5,6606414	6,09045E-08	2,11138E-07
HDHD2	-0,506608759	5,344967004	6,15397E-08	2,13231E-07
TOMM6	0,662537202	6,967414917	6,22133E-08	2,15456E-07
AXL	-0,823447511	6,738484135	6,31623E-08	2,18631E-07
CCDC138	0,814297986	2,382121366	6,34606E-08	2,19552E-07
CDK2AP1	0,581086895	7,229475613	6,42147E-08	2,22049E-07
MARCH6	0,618746479	7,199453768	6,45695E-08	2,23162E-07
RPL38	0,677766025	8,943185896	6,54463E-08	2,26078E-07
SP2	-0,418647389	5,295901252	6,60038E-08	2,27889E-07
NR2F1	-0,890567737	5,003459201	6,79165E-08	2,34374E-07
COX5A	0,631663831	6,765929158	6,80674E-08	2,3472E-07
COL18A1	0,958876758	8,042346053	6,80856E-08	2,3472E-07
RFNG	0,471092459	5,694468319	6,83389E-08	2,35474E-07
FCER1G	-0,902648125	6,740243348	7,04211E-08	2,42526E-07
CSNK2A1	0,503767764	6,652348443	7,19092E-08	2,47526E-07
TET1	1,046842356	1,670242295	7,26998E-08	2,50121E-07
UBXN4	0,352133238	7,808274617	7,3439E-08	2,52537E-07
RBM6	-0,567127195	6,1650328	7,56706E-08	2,6008E-07
RPL23A	0,521661443	10,16169657	7,71042E-08	2,64873E-07
TRAPPC10	-0,410483094	6,160380047	7,7663E-08	2,66659E-07
SCYL1	0,369922269	7,048310707	7,90461E-08	2,71271E-07
ZNF408	0,415399948	4,098565718	7,99242E-08	2,74147E-07
AHSA1	0,449342649	6,970584009	8,02045E-08	2,74969E-07
FAM190B	-0,481983596	6,390271667	8,03344E-08	2,75276E-07
COX6A1	0,809709997	8,165445632	8,04184E-08	2,75426E-07
CCAR1	0,389460659	6,312269965	8,06981E-08	2,76245E-07
UBE2F	0,510154394	5,730843755	8,15406E-08	2,78989E-07
SYDE1	-0,725230082	4,367480065	8,29756E-08	2,83757E-07
ANXA6	-0,598186493	7,611220451	8,38351E-08	2,86552E-07

NR2C1	-0,456040697	4,745839631	8,53787E-08	2,91682E-07
DDX55	0,502369874	4,94863986	8,54987E-08	2,91946E-07
EXOG	-0,378209904	3,219940881	8,58481E-08	2,92992E-07
PRMT1	0,499641491	7,229291802	8,64739E-08	2,9498E-07
SLC25A36	-0,481596456	6,391478638	8,738E-08	2,97922E-07
UBE2K	0,382225894	7,0894903	8,76516E-08	2,98699E-07
POLR2G	0,614874595	6,231684305	8,96651E-08	3,05408E-07
PHF23	0,427888007	5,638689228	9,00059E-08	3,06416E-07
FAM100B	0,651264927	6,382419377	9,00811E-08	3,06518E-07
AP4E1	-0,347898245	4,516825868	9,03456E-08	3,07265E-07
SHISA4	0,689905005	4,689384155	9,06509E-08	3,0815E-07
PAR-SN	-0,742161366	3,698538446	9,14616E-08	3,10751E-07
CHD9	-0,550548167	5,894858422	9,3412E-08	3,17219E-07
BCL6	-0,615748361	6,693705229	9,36624E-08	3,17911E-07
GAS5	1,158002771	8,23017022	9,528E-08	3,23241E-07
ITPA	0,514111183	5,734767615	9,57593E-08	3,24705E-07
TRMT5	0,460537776	4,887998004	9,65755E-08	3,2731E-07
TYSND1	0,591395716	5,008840418	9,66621E-08	3,27441E-07
SNHG4	0,803284139	0,548527747	9,69849E-08	3,28371E-07
IRF3	0,44264245	6,235166949	9,73964E-08	3,29601E-07
MAML2	-0,838860025	4,86250421	9,77022E-08	3,30472E-07
TINF2	-0,397628005	6,442834102	9,92405E-08	3,35509E-07
ZXDC	-0,428390259	6,197352132	9,95027E-08	3,36229E-07
TKT	0,746815887	9,449246254	9,98779E-08	3,37329E-07
NOMO2	0,530935853	7,761259092	1,0716E-07	3,61746E-07
CPEB4	-0,59834951	6,204993824	1,07403E-07	3,62385E-07
EIF2S3	0,675153247	8,607729955	1,07921E-07	3,63952E-07
EPN2	-0,506248481	6,107200724	1,08105E-07	3,64393E-07
MYD88	-0,470703744	6,815779701	1,08844E-07	3,66702E-07
CTSA	0,618900711	8,549144477	1,09418E-07	3,68456E-07
WDR48	-0,352841561	5,764246954	1,12826E-07	3,79745E-07
SSH1	-0,578621068	6,472853723	1,1298E-07	3,80074E-07
SMG5	0,529901429	7,396312872	1,13576E-07	3,8189E-07
IFT57	-0,940673789	7,41010372	1,1376E-07	3,82322E-07
NPAS2	1,110249725	4,991735549	1,15351E-07	3,87476E-07
SMAD4	-0,465925241	6,725304071	1,16651E-07	3,91652E-07
HSPB1	0,861615763	9,228171507	1,17375E-07	3,93887E-07
GPR180	0,715161541	4,647894992	1,19596E-07	4,01145E-07
NUP107	0,964769987	6,032527121	1,19772E-07	4,01536E-07
HSPA9	0,476778853	8,35487324	1,2041E-07	4,03477E-07
FAM91A1	0,434270438	6,608577875	1,20629E-07	4,04011E-07
PSMG1	0,731454588	4,942965415	1,22834E-07	4,11195E-07
AES	-0,475973858	8,98357332	1,23196E-07	4,12203E-07
PIP5K1C	-0,461930169	6,165569099	1,23771E-07	4,13925E-07
MAP7D1	-0,52848332	7,319803013	1,23968E-07	4,1438E-07
UBA2	0,627053591	7,439702509	1,24423E-07	4,15698E-07
EXOSC3	0,528837439	4,38210537	1,24933E-07	4,17198E-07
CBR3	1,434477588	3,883994127	1,26E-07	4,20554E-07
GNPDA1	0,589750984	6,226143288	1,26321E-07	4,21421E-07
CHI3L1	1,945789281	8,1494477	1,26921E-07	4,23213E-07

NAA38	0,433714782	4,794581022	1,27125E-07	4,23689E-07
HSD3B7	0,837285444	5,828479726	1,28616E-07	4,28448E-07
ABI3	-0,713690066	4,585149805	1,28962E-07	4,29389E-07
IFNGR1	-0,713962067	7,849987527	1,29055E-07	4,29492E-07
UBAP2L	0,453457299	7,845383251	1,29385E-07	4,30378E-07
RBM8A	0,449237031	7,239393705	1,29805E-07	4,31566E-07
DDR1	0,736293094	8,577914979	1,30736E-07	4,34399E-07
CLIC1	0,653098493	9,369548214	1,30785E-07	4,34399E-07
GSR	0,927535381	6,819898685	1,31466E-07	4,36448E-07
AMMECR1	0,512583143	4,716915142	1,31643E-07	4,36824E-07
TMEM67	-0,665562492	4,111055298	1,32E-07	4,37687E-07
H3F3A	0,553943085	9,909979879	1,32031E-07	4,37687E-07
LRIG3	1,325380839	6,31144476	1,32344E-07	4,38512E-07
RPL30	0,608120437	10,08893043	1,32915E-07	4,40187E-07
TNFRSF10D	-0,919129795	4,375625762	1,3301E-07	4,40288E-07
RSBN1	-0,436541879	5,262969621	1,33135E-07	4,40489E-07
DCLRE1A	0,635841085	4,634319215	1,33436E-07	4,41271E-07
MBNL2	-0,523474297	6,828528382	1,33592E-07	4,41572E-07
ENY2	0,544059249	5,751919634	1,33952E-07	4,42549E-07
MGST1	0,898294244	8,603252803	1,34733E-07	4,44912E-07
SLC39A1	0,477818462	8,078151276	1,34912E-07	4,45288E-07
XRCC4	0,736822371	3,895237473	1,35649E-07	4,47503E-07
DYRK1A	-0,400157219	6,545201392	1,35854E-07	4,47962E-07
NOLC1	0,520028227	7,253090849	1,38086E-07	4,55103E-07
RPS19	0,773545954	10,08112865	1,38621E-07	4,56647E-07
CEP76	0,549819301	3,257135515	1,40121E-07	4,61365E-07
EIF3H	0,473260581	8,363387416	1,40713E-07	4,63088E-07
ATP1B1	1,027878619	9,495056784	1,4122E-07	4,64533E-07
IMPDH2	0,534983203	7,434134342	1,43743E-07	4,72606E-07
LEF1	0,800025178	3,609558225	1,45665E-07	4,78695E-07
ABCA5	-0,746886841	4,273211148	1,46841E-07	4,82327E-07
KRT19	1,173242818	9,357468546	1,47527E-07	4,84345E-07
ARF4	0,502151325	8,579961084	1,48498E-07	4,873E-07
PLXNB1	-0,781584731	6,589696518	1,49387E-07	4,89981E-07
SLC25A20	-0,524096509	4,787595673	1,49963E-07	4,91635E-07
TNC	1,600223251	8,68796463	1,51418E-07	4,96164E-07
ABHD13	-0,428918005	5,218523909	1,53361E-07	5,02291E-07
DUSP12	0,507612858	4,433032037	1,54938E-07	5,07213E-07
TRIB1	-0,747475551	7,47004081	1,55778E-07	5,09708E-07
TAF3	-0,480549366	3,825730971	1,55901E-07	5,09708E-07
ZFYVE26	-0,543482102	5,827381865	1,55925E-07	5,09708E-07
NSMCE2	0,736841346	4,969017401	1,56966E-07	5,12866E-07
YIF1B	0,559263174	5,88532481	1,57979E-07	5,1593E-07
AATF	0,503342322	6,433692818	1,58244E-07	5,16546E-07
SYAP1	0,576701553	6,429630575	1,58584E-07	5,17408E-07
CEPT1	-0,436070986	5,429660558	1,60073E-07	5,22017E-07
FBXO28	0,408050975	6,320448853	1,60584E-07	5,23433E-07
SLC35B3	0,450035598	5,192749916	1,61804E-07	5,27157E-07
TRMT112	0,621209123	7,570077303	1,63024E-07	5,30878E-07
PBRM1	-0,449114013	6,317427681	1,63877E-07	5,33403E-07

UBE2V2	0,515560495	5,847145665	1,64353E-07	5,34696E-07
ARHGEF12	-0,56083295	8,117392686	1,67354E-07	5,44199E-07
C10orf88	0,433685782	3,718697095	1,69009E-07	5,4932E-07
PLCD1	-0,659689028	4,720050904	1,69431E-07	5,50428E-07
PA2G4P4	0,719791215	0,70052659	1,70468E-07	5,53431E-07
SDHA	0,554245326	7,437639946	1,70518E-07	5,53431E-07
SDC1	0,771054261	9,70581218	1,72624E-07	5,60001E-07
YEATS4	1,264141228	5,215744476	1,75767E-07	5,69928E-07
TSC22D3	-0,868664938	8,542980584	1,7883E-07	5,79583E-07
ZNF592	-0,541090263	6,050076592	1,82934E-07	5,92383E-07
TADA2B	-0,312161168	5,749171009	1,82953E-07	5,92383E-07
HIAT1	-0,373122716	6,415482421	1,83699E-07	5,94517E-07
TSN	0,51078957	7,06741136	1,84022E-07	5,95278E-07
HCCS	0,415144318	5,085295931	1,85742E-07	6,00556E-07
UNC119B	-0,693390217	5,947186386	1,86677E-07	6,03293E-07
FMNL3	-0,536617933	6,088359485	1,8774E-07	6,06442E-07
CDC5L	0,388557197	6,170164561	1,88633E-07	6,09037E-07
CPSF6	0,426000155	6,651400415	1,89394E-07	6,11012E-07
APIP	-0,565223901	5,025043034	1,89424E-07	6,11012E-07
RBBP4	0,381166203	8,016931585	1,92206E-07	6,19694E-07
GNPTAB	-0,464633099	6,928687536	1,93354E-07	6,23101E-07
FSCN1	1,221586608	7,251348229	1,94279E-07	6,25787E-07
LOC440173	1,664689047	1,505442652	1,95906E-07	6,30728E-07
GPAA1	0,517171563	7,385392824	1,97466E-07	6,35452E-07
CEP68	-0,430747145	5,339079547	1,97994E-07	6,36849E-07
SLC43A2	-0,590419202	6,165953693	2,00149E-07	6,43478E-07
PPIC	0,540109738	6,190742597	2,02064E-07	6,49327E-07
AAGAB	0,493309069	6,147225337	2,03758E-07	6,54464E-07
TAF1A	0,447936792	3,25303389	2,07542E-07	6,66305E-07
TRIM22	-0,75430629	7,044264223	2,0801E-07	6,67492E-07
DPM2	0,590019585	6,190541901	2,08451E-07	6,68594E-07
PIM2	0,946086047	6,515306903	2,11352E-07	6,77578E-07
C1orf43	0,655735091	8,764624406	2,14337E-07	6,86827E-07
ACVR1B	0,717052502	6,514240837	2,15862E-07	6,91387E-07
GLRX2	0,617184808	3,257563501	2,17135E-07	6,95139E-07
RPS16	0,926631276	10,33909721	2,18505E-07	6,99164E-07
STX3	-0,499866946	6,392031353	2,18598E-07	6,99164E-07
LSM7	0,676771477	5,358671955	2,21479E-07	7,08049E-07
TMEM208	0,700832052	5,938788321	2,26946E-07	7,25184E-07
SEC61A2	0,725019035	2,957656332	2,2879E-07	7,30734E-07
SEPT9	0,491692285	8,78952533	2,29364E-07	7,32225E-07
TRAF3IP2	-0,513247638	5,101271993	2,29848E-07	7,33426E-07
NDUFB9	0,770000013	7,674263314	2,34252E-07	7,47129E-07
ZBTB7A	-0,517180202	5,835661313	2,34856E-07	7,48706E-07
EIF2B1	0,358323191	6,113383153	2,35572E-07	7,50639E-07
SUPV3L1	0,378277855	4,904300353	2,4032E-07	7,65409E-07
CDK8	0,630598323	3,695828782	2,42592E-07	7,72284E-07
ATF7	-0,401106785	6,419214047	2,43847E-07	7,75566E-07
IFITM1	1,025841281	7,991231442	2,4385E-07	7,75566E-07
ZNF548	-0,442188405	4,188531267	2,49625E-07	7,93563E-07

XPO6	0,394633705	7,00575305	2,51721E-07	7,99853E-07
SCRIB	0,676491361	6,838897763	2,51867E-07	7,99945E-07
VPS13C	-0,601617982	6,827207516	2,52718E-07	8,02274E-07
RAD23B	0,372251094	8,057654521	2,6165E-07	8,30243E-07
CTTN	0,49957301	8,367018607	2,62898E-07	8,33815E-07
SDF4	0,433142567	7,770119997	2,66331E-07	8,4431E-07
PTPRG	-0,855545867	5,532681217	2,66676E-07	8,45009E-07
DHX40	0,47827468	6,723769877	2,70828E-07	8,57768E-07
TMED4	0,439295757	6,773830602	2,73464E-07	8,65716E-07
VKORC1	0,631795364	6,6348155	2,746E-07	8,68907E-07
SEC22B	0,453166939	7,01278444	2,77115E-07	8,7646E-07
CUL4B	0,404731061	6,727830754	2,79086E-07	8,82283E-07
CAPN7	-0,340643265	5,403868885	2,8327E-07	8,95098E-07
VBP1	0,592339206	6,242486208	2,83991E-07	8,96959E-07
YRDC	0,527261983	5,041052095	2,85592E-07	9,01599E-07
ALAS1	-0,465434651	6,812658597	2,89629E-07	9,13918E-07
ANAPC1	0,484202958	5,569196852	2,93885E-07	9,26921E-07
NDUFAF2	0,661674215	4,557062137	2,94289E-07	9,27765E-07
SYNE2	-0,754476674	7,532687873	2,96645E-07	9,34762E-07
PDXP	0,635111605	3,059223223	2,98338E-07	9,3966E-07
CCM2	0,618885442	6,298221191	3,05991E-07	9,6332E-07
UAP1	0,600982022	6,639850796	3,06296E-07	9,63836E-07
USP8	-0,381820379	6,284481486	3,11679E-07	9,80323E-07
MLL5	-0,482482315	6,818173514	3,19609E-07	1,0048E-06
CCDC88A	-0,642632141	5,856978108	3,21134E-07	1,00913E-06
USP47	-0,483256036	6,641194901	3,21975E-07	1,01131E-06
PTGES3	0,46100005	8,934158362	3,26094E-07	1,02377E-06
DRAM1	-0,988726212	8,157552764	3,29964E-07	1,03523E-06
CRLS1	-0,609938577	6,873763353	3,30048E-07	1,03523E-06
ERI2	0,446492459	4,519813802	3,34145E-07	1,0476E-06
UBE2E2	-0,596251662	5,028070319	3,40916E-07	1,06834E-06
KTI12	0,457003874	3,961521227	3,45878E-07	1,08339E-06
VPS35	0,344802006	7,600743134	3,46731E-07	1,08556E-06
UTP3	-0,393952833	5,490787553	3,50913E-07	1,09815E-06
CPSF1	0,512478299	6,75637001	3,54542E-07	1,109E-06
SEC23A	0,630529486	6,625547595	3,56057E-07	1,11323E-06
NANOG	-0,52600228	1,210638491	3,5832E-07	1,11972E-06
SLC25A17	0,456188877	5,155191789	3,58461E-07	1,11972E-06
SRXN1	1,334262198	7,25829471	3,58709E-07	1,11998E-06
SNRNP40	0,444936306	5,836040008	3,6204E-07	1,12986E-06
CDH1	0,841437038	9,175125548	3,64992E-07	1,13855E-06
ARPC1A	0,617246556	7,778380865	3,65192E-07	1,13866E-06
PABPC1L	1,125688014	4,250246343	3,67772E-07	1,14618E-06
HIF1A	0,707385462	8,335011096	3,70156E-07	1,15308E-06
MRPS2	0,526673005	5,635855835	3,7763E-07	1,17583E-06
NCBP2	0,406798903	6,814331609	3,86333E-07	1,20184E-06
LEO1	0,437542749	4,780572397	3,86339E-07	1,20184E-06
TMEM9	0,684576001	7,647212763	3,87477E-07	1,20484E-06
FAM20B	-0,395535884	6,70946053	3,93078E-07	1,22169E-06
ZC3H6	-0,508665354	4,589484477	3,9488E-07	1,22674E-06

ZC3H7B	-0,496749703	7,297270616	3,97522E-07	1,23438E-06
GTF3C3	0,394701599	5,460963243	3,98061E-07	1,23549E-06
PRPF38B	-0,361598442	5,94204516	3,98707E-07	1,23694E-06
ZNF212	0,462090627	4,52394242	4,05569E-07	1,25765E-06
GTF3A	0,839121401	7,279891648	4,08835E-07	1,2672E-06
AMPD2	0,42432184	5,671246518	4,13438E-07	1,28089E-06
TXNDC12	0,486325687	7,063667124	4,15304E-07	1,28609E-06
AQP1	-1,396615895	10,14713981	4,17682E-07	1,29277E-06
VDAC3	0,646924933	7,026312503	4,17841E-07	1,29277E-06
HSPA4	0,405891455	7,206068565	4,19358E-07	1,29646E-06
PHF10	-0,470872417	6,286315495	4,19415E-07	1,29646E-06
EEA1	-0,474699898	5,595520865	4,19688E-07	1,29672E-06
WDR34	0,611701689	6,635853182	4,22797E-07	1,30573E-06
EHMT2	0,547026931	6,411747651	4,26946E-07	1,31795E-06
LGALS3BP	0,752660321	10,56238999	4,28813E-07	1,32311E-06
PLTP	0,996294332	7,822743645	4,30887E-07	1,32891E-06
NICN1	-0,532324361	4,62071381	4,32007E-07	1,33176E-06
ATP5G2	0,564510957	8,416988632	4,38123E-07	1,35001E-06
KIAA0391	0,75258246	6,87761046	4,47122E-07	1,37711E-06
NDUFA12	0,520173663	5,773757232	4,4961E-07	1,38415E-06
MAPK1	-0,359627187	7,641966851	4,50661E-07	1,38676E-06
ORMDL2	0,63467058	6,084654386	4,53263E-07	1,39414E-06
ASPH	1,093327506	9,122615549	4,54595E-07	1,3976E-06
ATXN1	-0,462299129	6,137980847	4,58451E-07	1,40882E-06
PRMT2	-0,388820992	6,888835911	4,58961E-07	1,40975E-06
VRK1	0,704059166	4,237379891	4,62635E-07	1,41996E-06
SPATS2L	0,62081002	7,633303058	4,62701E-07	1,41996E-06
RABGAP1L	-0,438428627	5,047421332	4,66333E-07	1,43046E-06
PRRC1	0,4119478	6,931024634	4,68898E-07	1,43769E-06
ASAP3	-0,703731833	5,408274509	4,76284E-07	1,45968E-06
PIP4K2C	0,67682759	6,823751678	4,77228E-07	1,46191E-06
DDX27	0,518780608	6,41263137	4,78886E-07	1,46633E-06
HLX	-0,973421972	4,161850389	4,794E-07	1,46725E-06
KIF3A	-0,465808731	5,051751995	4,79849E-07	1,46796E-06
TMEM63B	-0,607291958	7,271616738	4,80394E-07	1,46897E-06
HDAC5	-0,436534703	6,458562162	4,80982E-07	1,47011E-06
PGM1	-0,567835676	7,253491522	4,91158E-07	1,50054E-06
ARIH2	-0,315317261	6,361007224	4,98701E-07	1,5229E-06
ABT1	0,498116149	5,777335602	4,99948E-07	1,52603E-06
DGKA	0,782360218	5,101426921	5,04688E-07	1,53981E-06
GSN	-0,691746182	9,528613246	5,0573E-07	1,5423E-06
PPP2R5E	0,467172183	5,90316506	5,08682E-07	1,55061E-06
FYN	-0,673766123	6,003605678	5,14347E-07	1,56717E-06
MRPS14	0,489836566	4,800131232	5,24531E-07	1,59749E-06
GAL3ST4	0,765140005	4,345369035	5,24879E-07	1,59784E-06
SUB1	0,594956108	7,875219316	5,33353E-07	1,62291E-06
IER3	1,05514879	7,099607228	5,38278E-07	1,63716E-06
LITAF	-0,465069171	8,47825938	5,40604E-07	1,6435E-06
EIF3E	0,660727755	9,048817546	5,42493E-07	1,64851E-06
RBM4	0,319063072	7,12994704	5,43695E-07	1,65143E-06

SLC26A2	-0,767276025	5,811065003	5,50706E-07	1,67198E-06
PLP2	0,826682657	7,704507162	5,51501E-07	1,67365E-06
ARMC10	0,518508811	6,450584467	5,78609E-07	1,75513E-06
MAEA	0,33046632	6,199737325	5,79476E-07	1,75698E-06
C7orf43	0,516481816	4,594798418	5,8069E-07	1,75988E-06
CISD1	0,536940506	5,032766438	5,87537E-07	1,77984E-06
SLC7A6	-0,495949457	5,973060482	5,89903E-07	1,78621E-06
XRCC3	0,585561421	4,236436201	5,91835E-07	1,79127E-06
EFNB1	-0,873981819	5,865077756	5,99587E-07	1,81392E-06
C16orf58	0,527697549	7,285971389	6,06098E-07	1,83281E-06
MPPE1	-0,435731071	5,945882813	6,20528E-07	1,87561E-06
PAPD5	-0,438718696	4,820480465	6,2248E-07	1,88068E-06
NDUFAB1	0,546331953	6,163486563	6,30472E-07	1,90398E-06
NUDT16	-0,529783559	5,835422903	6,4366E-07	1,94295E-06
ATL3	0,531035022	5,414743447	6,49603E-07	1,96002E-06
LBR	0,493118079	6,545720811	6,58694E-07	1,98657E-06
RANGAP1	0,49458387	6,912992485	6,62426E-07	1,99694E-06
LDOC1	1,046440309	5,642277234	6,76614E-07	2,03881E-06
POLDIP3	-0,365089612	6,938409435	6,85957E-07	2,06569E-06
NEK9	-0,419905325	6,744038324	6,86139E-07	2,06569E-06
CEBPD	-0,769958189	7,29404594	6,95007E-07	2,09146E-06
FAM127B	0,510497588	6,020566865	6,97419E-07	2,09779E-06
SERPINB6	-0,502825864	7,611655775	7,05095E-07	2,11995E-06
DCAF4	0,516036843	4,275551359	7,10513E-07	2,1353E-06
PSMA2	0,521529318	7,291984857	7,13608E-07	2,14365E-06
PCGF5	-0,453289681	6,684534046	7,16144E-07	2,15032E-06
PRKCD	-0,510388702	6,93872225	7,19549E-07	2,1596E-06
DUSP16	-0,578272342	6,677502875	7,24845E-07	2,17454E-06
FBXO6	0,705376627	5,411795544	7,5402E-07	2,26107E-06
EFNB2	-0,875714212	6,658654559	7,56341E-07	2,26703E-06
PES1	0,419790582	6,599196083	7,57058E-07	2,26818E-06
TMEM115	-0,349558081	6,051806	7,74124E-07	2,31829E-06
ALAD	-0,43529149	6,228125052	7,75405E-07	2,32111E-06
TBCC	0,502615441	5,170229822	7,758E-07	2,32127E-06
RRAGA	-0,356903551	6,698600221	7,81403E-07	2,33701E-06
DYNC1LI1	-0,309740871	5,737585502	7,82101E-07	2,33807E-06
MAPK6	0,616482103	6,415986935	7,88395E-07	2,35586E-06
MRS2	0,566782005	5,951977887	7,9246E-07	2,36697E-06
FBXL12	-0,337231737	4,416761141	8,0816E-07	2,4128E-06
POLI	-0,468833386	4,71816416	8,10883E-07	2,41987E-06
NKTR	-0,658510294	6,217969306	8,20837E-07	2,44851E-06
RPN1	0,412826672	9,147392448	8,25534E-07	2,46144E-06
UFD1L	0,491926279	6,320039513	8,28466E-07	2,4691E-06
WBP2	-0,411516466	7,811895908	8,31676E-07	2,47759E-06
CD47	-0,604118923	8,631514143	8,32861E-07	2,48003E-06
GMCL1	0,470605582	4,817656326	8,66771E-07	2,57988E-06
AP1M1	-0,385789915	6,152076076	8,68509E-07	2,58393E-06
YWHAQ	0,51632021	8,960853655	8,69228E-07	2,58494E-06
DNMT1	0,64266283	6,695295392	8,70631E-07	2,58798E-06
LCLAT1	0,404411846	5,434103607	8,93514E-07	2,65485E-06

RAB5B	-0,368331684	8,197518996	8,94156E-07	2,6556E-06
PPP1R16A	0,566348038	5,533511032	9,02963E-07	2,68059E-06
TSC22D1	-0,661782646	8,699474777	9,07468E-07	2,69279E-06
ANKRD11	-0,487403598	7,200921249	9,08369E-07	2,69429E-06
GOLPH3	0,464378534	8,007525032	9,14017E-07	2,70986E-06
RPL12	0,70864874	10,17208406	9,22872E-07	2,73492E-06
MRFAP1	-0,315018355	8,436764046	9,4142E-07	2,78868E-06
APOC1	-1,01643715	7,846975966	9,42514E-07	2,79071E-06
CNOT6L	-0,343566405	6,170034369	9,43389E-07	2,79209E-06
PLEKHA2	-0,534176495	6,267467465	9,48891E-07	2,80715E-06
TMEM64	-0,740204995	5,593061514	9,51429E-07	2,81344E-06
CDV3	-0,457645899	7,893486594	9,52846E-07	2,81641E-06
PARK7	0,491285357	7,999981813	9,56025E-07	2,82458E-06
FKBP2	0,590884566	6,539375454	9,57322E-07	2,82719E-06
STX6	0,385113986	6,526086941	9,63201E-07	2,84332E-06
AARS	0,492010215	7,726559496	9,82267E-07	2,89835E-06
ASCC3	0,518741477	6,164619364	9,902E-07	2,91984E-06
TEX10	0,47132258	4,691659306	9,90408E-07	2,91984E-06
RP2	-0,483159155	5,450765606	9,97282E-07	2,93884E-06
IDH1	0,800243073	8,045763605	1,0031E-06	2,95472E-06
RNF123	-0,388009122	5,55701737	1,00803E-06	2,96793E-06
DPH3	-0,48721798	5,600028899	1,01091E-06	2,97514E-06
PNPT1	0,479165451	5,821337163	1,01663E-06	2,99068E-06
HNRNPF	0,354474761	8,368098898	1,02254E-06	3,00676E-06
NT5DC2	0,643337136	6,227744867	1,03176E-06	3,03256E-06
TTC27	0,402562905	4,782713848	1,03765E-06	3,04858E-06
PLXDC2	-0,684825624	5,472199966	1,03941E-06	3,05242E-06
COPE	0,504426888	7,439875474	1,04147E-06	3,05715E-06
KRT17	2,179358466	6,9272729	1,04436E-06	3,06432E-06
ACVR1	0,426085253	6,201867588	1,04632E-06	3,06874E-06
ELF1	-0,392777869	6,812930484	1,05158E-06	3,08286E-06
IGFBP2	1,570515736	7,969147108	1,05362E-06	3,08752E-06
ANKIB1	0,416948664	6,418577766	1,06766E-06	3,12731E-06
MRPL22	0,57248271	5,010070294	1,06898E-06	3,12982E-06
DCHS1	-0,870493439	5,699490102	1,07255E-06	3,13893E-06
RPS5	0,896622211	9,950369858	1,07505E-06	3,14489E-06
ZNF468	0,585208688	5,007378021	1,08542E-06	3,17386E-06
ZNF131	0,451024703	5,193915682	1,08742E-06	3,17837E-06
SLC30A1	0,687902364	4,720024881	1,08885E-06	3,18118E-06
GNL2	0,423712176	6,40570972	1,10516E-06	3,22744E-06
TSR1	0,39195854	6,017167758	1,12054E-06	3,27097E-06
PRPF40A	0,304335947	7,238754136	1,1394E-06	3,32459E-06
ENAH	0,527084807	7,691750235	1,14275E-06	3,33293E-06
MLLT10	-0,41388662	5,313813707	1,15251E-06	3,35998E-06
FAM188A	-0,464726979	4,861197917	1,15703E-06	3,37171E-06
GOLIM4	-0,590583229	5,996167198	1,16826E-06	3,40299E-06
TGFB2	-1,064949776	5,301675429	1,17691E-06	3,42672E-06
RPF2	0,493614992	4,553318972	1,20173E-06	3,49748E-06
RNF20	-0,367815964	6,09842015	1,2051E-06	3,50579E-06
H19	2,915645225	9,022136335	1,20827E-06	3,51352E-06

DHRSX	0,531068279	5,129495317	1,21422E-06	3,52931E-06
GLUL	-0,565439784	10,19095497	1,22461E-06	3,558E-06
ZNF7	0,375026191	5,024650788	1,26354E-06	3,66954E-06
NR4A2	-1,142832678	5,552352439	1,26813E-06	3,68131E-06
ERCC2	0,508964164	4,852438632	1,27218E-06	3,6915E-06
YWHAG	0,481968374	8,346044044	1,29443E-06	3,75446E-06
C11orf31	0,64416643	6,709006976	1,32612E-06	3,84474E-06
CHCHD10	0,770365011	5,215818868	1,3337E-06	3,86507E-06
LSM1	0,76008805	5,517821105	1,34699E-06	3,90191E-06
CDK2	0,528824073	5,582506615	1,35217E-06	3,91527E-06
COMMD5	0,566338902	5,532208698	1,35346E-06	3,91733E-06
GAS2L1	-0,534615607	5,124750135	1,3668E-06	3,95287E-06
ID2	-0,740550664	6,336967619	1,3669E-06	3,95287E-06
SGMS1	-0,653441081	6,198322036	1,36807E-06	3,95459E-06
CLSTN3	0,558584009	6,636642779	1,37279E-06	3,96653E-06
OSGIN2	-0,605333007	5,947920217	1,37366E-06	3,96737E-06
IL6ST	-0,83028582	6,575552555	1,38377E-06	3,99488E-06
PEF1	-0,379595894	6,871740796	1,38639E-06	3,99925E-06
PLEKHB2	-0,378952689	7,944577086	1,38645E-06	3,99925E-06
RAB10	0,424377679	8,01393268	1,39712E-06	4,02832E-06
PIH1D1	0,378512565	6,032362163	1,40009E-06	4,03518E-06
IRF1	-0,684573673	7,196477353	1,41638E-06	4,08041E-06
RPS21	0,895005462	9,270064386	1,41964E-06	4,08805E-06
MRPS11	0,525029957	5,115204248	1,43847E-06	4,14052E-06
NAV1	0,734169096	6,224761934	1,43958E-06	4,14199E-06
STAT1	0,758220188	9,015973602	1,44161E-06	4,14608E-06
EEF1B2	0,778955713	9,312202275	1,4616E-06	4,20179E-06
EIF3M	0,404227613	7,288005994	1,47025E-06	4,22488E-06
NMD3	0,476338977	6,40202873	1,47112E-06	4,2256E-06
RHBDD2	-0,443576638	7,457091963	1,48993E-06	4,27781E-06
NFE2L2	-0,499836194	7,65214751	1,49262E-06	4,28373E-06
COX18	0,475859782	5,33680428	1,49594E-06	4,29148E-06
RALGDS	-0,508587849	6,727209367	1,50946E-06	4,32844E-06
SPCS2	0,445123361	7,032580184	1,51753E-06	4,34973E-06
SHPRH	-0,465126622	4,217839472	1,55255E-06	4,44824E-06
DDX24	-0,369531275	7,636918424	1,5547E-06	4,45172E-06
POLE	0,681762557	5,451909589	1,55507E-06	4,45172E-06
NAF1	-0,343285098	3,681641564	1,56582E-06	4,48061E-06
PARP2	0,487032067	4,691554	1,56822E-06	4,48561E-06
TXN	0,863991399	8,21727669	1,57146E-06	4,49301E-06
SNHG6	0,760523518	6,189772886	1,61637E-06	4,61946E-06
ANKRD28	-0,447113993	6,324942078	1,61914E-06	4,62545E-06
COX6C	0,575010237	7,067106497	1,66337E-06	4,74981E-06
PARVB	-0,565878939	5,171267447	1,68683E-06	4,81477E-06
DNM1	0,810066116	3,964091446	1,69931E-06	4,84837E-06
RBM23	-0,279598365	6,610083095	1,7021E-06	4,85428E-06
CREB1	-0,267637278	6,158354662	1,71203E-06	4,88058E-06
RBM5	-0,463785238	6,57455971	1,7214E-06	4,90522E-06
TM9SF4	0,419589811	7,200764474	1,72262E-06	4,90667E-06
G3BP1	0,396876248	7,138746076	1,73983E-06	4,95361E-06

PRKACA	-0,323473729	6,688621267	1,74269E-06	4,95968E-06
SF3B3	0,408521854	7,756410724	1,76301E-06	5,01542E-06
KIAA0494	-0,444660986	7,986025349	1,76416E-06	5,01661E-06
E2F4	0,310883292	6,44427681	1,79883E-06	5,11305E-06
S100A11	0,680579076	9,69843817	1,80517E-06	5,12893E-06
C2orf43	0,449103568	4,730626309	1,80976E-06	5,13983E-06
CCDC8	-0,89221487	3,91507872	1,83757E-06	5,21665E-06
PIKFYVE	-0,42033196	5,591643394	1,85769E-06	5,27156E-06
LIN37	0,629837015	3,650706501	1,86926E-06	5,30221E-06
OAS3	0,818199272	7,412865893	1,88516E-06	5,34508E-06
SEC11C	1,241776194	7,296470638	1,90078E-06	5,38712E-06
AZIN1	0,504438761	8,144043663	1,90462E-06	5,39576E-06
TRIM28	0,41750448	8,343897205	1,9148E-06	5,42235E-06
CSGALNACT2	-0,415349403	5,680802502	1,96926E-06	5,57426E-06
STEAP3	0,896665934	6,845305249	1,97995E-06	5,6022E-06
PSMA7	0,583600689	7,962696765	1,9987E-06	5,65291E-06
PTBP1	0,334550054	8,331896627	2,00525E-06	5,66906E-06
CCS	0,389498507	5,181742649	2,0104E-06	5,68128E-06
EMG1	0,732634827	5,893732609	2,01392E-06	5,68886E-06
MEIS3P1	-0,4863999	5,965583511	2,03525E-06	5,74629E-06
ZBED4	0,442171111	5,423074096	2,03593E-06	5,74629E-06
DHDDS	0,329779426	5,705955171	2,0641E-06	5,82337E-06
ITFG3	0,487646694	7,121840534	2,07141E-06	5,84061E-06
DSCR3	-0,341607807	6,166555536	2,07192E-06	5,84061E-06
FAM49B	0,573728373	6,979421394	2,1066E-06	5,9359E-06
POLM	0,475868683	4,775568662	2,11753E-06	5,96423E-06
OGDH	-0,456129103	7,789180972	2,12954E-06	5,99561E-06
SMPD4	0,406189466	6,682376357	2,1372E-06	6,01468E-06
CDKN1A	-0,741169611	7,782288829	2,15997E-06	6,07626E-06
POLR2B	0,370017388	7,146492132	2,18329E-06	6,13932E-06
MFN2	-0,362552439	7,395617503	2,21666E-06	6,23058E-06
IAH1	0,640950406	5,664000002	2,23018E-06	6,26602E-06
MAVS	-0,475227443	7,202507274	2,24829E-06	6,3143E-06
USP37	0,376324764	4,91357089	2,2549E-06	6,33026E-06
TGFBI	1,391078794	9,235408945	2,26707E-06	6,36179E-06
CBLB	0,496374511	5,053780748	2,26937E-06	6,36561E-06
ADAT1	0,439582515	4,389343279	2,31252E-06	6,48399E-06
GADD45GIP1	0,623574151	5,773304987	2,35927E-06	6,61236E-06
CBR1	1,266014185	7,54338657	2,3774E-06	6,66044E-06
POLR3H	-0,553904889	6,522979176	2,38694E-06	6,68442E-06
LSG1	0,516264416	6,067139127	2,39044E-06	6,69146E-06
SLU7	-0,322589662	6,319691792	2,39789E-06	6,70956E-06
AACS	0,53128569	5,752352648	2,39978E-06	6,71209E-06
DCUN1D2	-0,381524802	4,080420015	2,40518E-06	6,72444E-06
ZNF395	-0,506672466	6,509099382	2,41621E-06	6,75252E-06
WHSC1	0,565505127	6,60741495	2,42425E-06	6,7722E-06
IK	-0,352947101	7,253182439	2,44096E-06	6,81609E-06
GLIS2	-0,576303634	5,891710815	2,44282E-06	6,8185E-06
BTF3	0,467704268	9,124384635	2,45673E-06	6,85453E-06
UBA6	0,413285161	6,057236611	2,47459E-06	6,90152E-06

GTF3C6	0,452679026	5,814204596	2,48292E-06	6,92193E-06
KIAA0513	-0,646943938	5,442199546	2,49418E-06	6,95048E-06
CFLAR	-0,540976552	7,215939213	2,4978E-06	6,95773E-06
SNX6	0,527806139	7,296900327	2,54202E-06	7,078E-06
RPL36	0,682947409	8,909804434	2,55656E-06	7,11559E-06
USP16	-0,325642724	5,990266816	2,56398E-06	7,13332E-06
MED10	0,569123371	5,339447401	2,58047E-06	7,17629E-06
IRAK1BP1	-0,564646312	1,816011842	2,58182E-06	7,1771E-06
PTCD2	0,395232968	3,031896502	2,61151E-06	7,25667E-06
BCCIP	0,403044566	6,06704888	2,62907E-06	7,30251E-06
THOC6	0,465226001	5,281822302	2,66442E-06	7,39767E-06
KIAA1147	-0,506337631	6,136275283	2,69071E-06	7,46762E-06
PPIL3	0,588158899	4,579187032	2,71609E-06	7,53498E-06
ZDBF2	-0,659300065	3,767633116	2,72071E-06	7,54475E-06
SRRT	0,352118188	6,696158611	2,72415E-06	7,55121E-06
RAB12	-0,403715219	6,13466853	2,73931E-06	7,59014E-06
PLK1S1	-0,442685722	3,983878055	2,75342E-06	7,62615E-06
RPS10	0,793986585	10,16202687	2,76932E-06	7,66706E-06
LONP2	0,374624778	6,409984318	2,79059E-06	7,72282E-06
TMX3	-0,422437748	6,05440426	2,84058E-06	7,85798E-06
MTMR14	-0,351712446	6,021511581	2,84744E-06	7,87377E-06
C19orf53	0,587167459	6,748677408	2,85015E-06	7,87805E-06
ATL2	0,563087951	6,81771457	2,8656E-06	7,91756E-06
ARCN1	0,348508366	8,23471134	2,90157E-06	8,0137E-06
ZNF511	0,574329084	5,256621449	2,93191E-06	8,09422E-06
NLGN2	0,680842029	4,870037007	2,95171E-06	8,14558E-06
TMEM170A	-0,314473366	5,388298464	2,9718E-06	8,19659E-06
REXO4	0,386538855	5,12694352	2,9726E-06	8,19659E-06
ARNT	-0,379270424	6,663751045	2,97612E-06	8,20299E-06
PSMD1	0,400287538	7,261194263	2,97999E-06	8,21033E-06
SMC2	0,60523477	5,231509213	2,98499E-06	8,22079E-06
RPS6KA5	-0,632207953	3,420633307	2,98903E-06	8,22858E-06
ZC3H13	-0,545177933	6,421345954	3,00592E-06	8,27175E-06
ILK	-0,387847452	7,400334466	3,03064E-06	8,33639E-06
TCEB2	0,604847906	7,616265355	3,04456E-06	8,3713E-06
TOMM34	0,51743322	5,760803414	3,05332E-06	8,392E-06
WDFY3	-0,441340791	6,218620952	3,09329E-06	8,49684E-06
GANAB	0,400549147	9,292889468	3,09395E-06	8,49684E-06
PSME2	0,607845337	7,699905534	3,11587E-06	8,5536E-06
NR1H2	-0,349122083	6,981673389	3,16086E-06	8,6736E-06
SFRP4	1,278896672	6,52402732	3,17912E-06	8,72019E-06
CAND1	0,7344494	7,566276732	3,20287E-06	8,7818E-06
SDCCAG8	-0,36075926	5,510231267	3,21604E-06	8,81438E-06
PPHLN1	0,335953292	6,078068906	3,21892E-06	8,81873E-06
TNIK	-0,890550414	4,855955386	3,25687E-06	8,91913E-06
ETV6	0,48674974	6,391059539	3,26954E-06	8,95023E-06
SLTM	-0,410350796	6,645588694	3,27339E-06	8,95716E-06
MRRF	0,428189352	4,714042689	3,34717E-06	9,15538E-06
NDUFB10	0,504709715	6,428580562	3,37048E-06	9,21543E-06
FAM102A	0,622674244	6,910023139	3,43854E-06	9,39775E-06

SMARCAL1	0,254351206	5,156140253	3,44922E-06	9,42318E-06
HINFP	0,368740409	4,636217585	3,49333E-06	9,53985E-06
FAM108A1	-0,392020564	5,750861099	3,4992E-06	9,55207E-06
PXK	-0,450773829	3,954231725	3,53784E-06	9,65367E-06
ASS1	0,940854529	7,818838853	3,54987E-06	9,68263E-06
HAUS5	0,544258629	4,707283259	3,55563E-06	9,69447E-06
EMP3	-0,730595645	6,25272673	3,55781E-06	9,69652E-06
SPRYD3	-0,4121502	6,470208379	3,56553E-06	9,7106E-06
VAT1	-0,520897935	8,408747929	3,56582E-06	9,7106E-06
ARPC5L	0,428831039	6,175627101	3,60209E-06	9,8035E-06
ZC3H3	0,478734578	5,318107363	3,60281E-06	9,8035E-06
SDC4	-0,617300218	9,785624996	3,60975E-06	9,81848E-06
NKG7	-1,001295368	4,400068493	3,70362E-06	1,00698E-05
CRISPLD2	-0,769544567	6,557592526	3,7197E-06	1,01094E-05
RSPRY1	0,428828689	4,985927663	3,76474E-06	1,02278E-05
C5orf24	-0,370984065	6,60050784	3,87092E-06	1,05121E-05
HSPA6	1,400569438	5,088620568	3,9188E-06	1,06378E-05
TRMT1	0,446688599	5,008067032	4,02496E-06	1,09217E-05
ARL8B	-0,334405285	7,301182037	4,02974E-06	1,09303E-05
SNX8	0,601863661	5,312161742	4,0681E-06	1,103E-05
PSMD13	0,471008143	7,504660849	4,08196E-06	1,10631E-05
CHD2	-0,497307502	7,257271602	4,09471E-06	1,10933E-05
AGAP3	-0,428441992	6,73708268	4,10619E-06	1,112E-05
SON	-0,392159058	8,354448556	4,14088E-06	1,12095E-05
NPRL2	0,488633352	4,677139885	4,15209E-06	1,12353E-05
MBOAT2	0,712438862	5,739075855	4,17898E-06	1,13036E-05
MOV10	0,457767861	6,12102269	4,21285E-06	1,13907E-05
CAPRIN2	-0,584766664	4,285720326	4,21449E-06	1,13907E-05
TTC21B	-0,417631555	4,668879284	4,22776E-06	1,1422E-05
RANBP9	0,387355232	6,471097914	4,26769E-06	1,15253E-05
CMC1	0,591944779	4,411827363	4,27783E-06	1,15481E-05
CNIH	0,653693013	6,947480956	4,28494E-06	1,15627E-05
MSC	1,083982424	3,51514571	4,30481E-06	1,16118E-05
CCDC51	0,469781899	4,202787289	4,33552E-06	1,169E-05
C7orf49	0,419413292	5,55017065	4,40491E-06	1,18724E-05
NOC4L	0,434992599	4,738950471	4,46175E-06	1,20208E-05
KLC2	0,497771624	5,454900411	4,46579E-06	1,2027E-05
PTEN	-0,328108285	6,891219348	4,4691E-06	1,20311E-05
CDKN2AIPNL	0,535887995	5,012984851	4,49845E-06	1,21053E-05
TALDO1	0,664887247	8,292687583	4,50477E-06	1,21117E-05
RAD9A	0,462366734	4,310983632	4,505E-06	1,21117E-05
REEP5	-0,427169886	7,99188177	4,50615E-06	1,21117E-05
SMC5	-0,42540729	5,902100842	4,53164E-06	1,21754E-05
SMC4	0,575896881	6,764236826	4,55582E-06	1,22356E-05
KCMF1	0,33918379	6,550065586	4,57907E-06	1,22932E-05
C14orf119	0,560558245	6,61128032	4,58221E-06	1,22968E-05
FAM106A	-0,486364868	1,303330219	4,59898E-06	1,23369E-05
MRPL53	0,563704579	4,599164914	4,64685E-06	1,24604E-05
ELP4	-0,307667769	4,011984869	4,65618E-06	1,24804E-05
TOLLIP	-0,341684628	6,461428825	4,65795E-06	1,24804E-05

MARK2	0,348401981	6,640794473	4,68268E-06	1,25417E-05
ZNF426	-0,437290138	3,15081429	4,71959E-06	1,26356E-05
RFX3	-0,733220748	2,395812856	4,73963E-06	1,26843E-05
SLC4A7	0,625600148	4,890288807	4,8099E-06	1,28673E-05
SVIL	-0,537691819	7,027911621	4,82751E-06	1,29093E-05
TTLL4	0,444859409	5,026899986	4,86324E-06	1,29998E-05
RRP1B	0,408648654	5,935383286	4,88663E-06	1,30544E-05
FEZ2	-0,348779426	6,049121429	4,88751E-06	1,30544E-05
ZCCHC7	0,423305466	4,987218514	4,93294E-06	1,31706E-05
C8orf33	0,468208062	6,405192661	5,00839E-06	1,33668E-05
DYSF	-0,702299144	5,932157669	5,07704E-06	1,35447E-05
ADAR	0,47647213	9,200571708	5,10025E-06	1,36013E-05
ERCC8	0,388843332	3,655721597	5,11483E-06	1,36349E-05
ENPP4	-0,626211806	6,301408442	5,13907E-06	1,36942E-05
MYO5C	-0,730668638	6,500699887	5,16603E-06	1,37606E-05
ACTG1	0,428937042	12,20501952	5,18088E-06	1,37948E-05
SSX2IP	0,705654879	4,685809377	5,19788E-06	1,38346E-05
UGGT1	0,470890274	7,387251465	5,21436E-06	1,38731E-05
CD320	0,663038269	5,642007657	5,21724E-06	1,38753E-05
DUSP22	-0,415520485	6,066991214	5,24669E-06	1,39443E-05
WDR70	0,39585152	5,324756786	5,24727E-06	1,39443E-05
TAF4	0,445445168	4,970004806	5,26671E-06	1,39905E-05
PEX13	0,3425947	6,342527182	5,26999E-06	1,39938E-05
HDAC3	0,358097774	6,218407825	5,27294E-06	1,39962E-05
VPS13A	-0,550403528	5,270827567	5,29948E-06	1,40611E-05
MFN1	0,429432238	5,622371231	5,33967E-06	1,41623E-05
TATDN1	0,563325599	4,723514299	5,49324E-06	1,45639E-05
ZNF304	-0,446189274	4,119339236	5,50556E-06	1,45909E-05
SIAH2	0,433571562	5,418049509	5,57618E-06	1,47723E-05
BRPF3	0,539299788	6,472082898	5,62634E-06	1,48994E-05
ARHGEF7	-0,395091868	6,116150334	5,64555E-06	1,49445E-05
SH3PXD2B	0,704500518	6,360383078	5,72488E-06	1,51486E-05
DOPEY2	0,568961614	5,884224458	5,78472E-06	1,5301E-05
ARL4C	0,73043959	6,934042154	5,84833E-06	1,54633E-05
RGS1	1,151089099	7,127338821	5,8568E-06	1,54796E-05
CTTNBP2NL	-0,415663144	6,003164098	5,92715E-06	1,56595E-05
ZNF37A	-0,544304215	4,682921633	5,93384E-06	1,56711E-05
HYMAI	-0,449523995	0,240893302	5,93835E-06	1,5677E-05
MTMR6	-0,456706657	6,165273887	6,01192E-06	1,5865E-05
RBMX	0,29137884	7,908699497	6,06568E-06	1,60003E-05
TOMM22	0,503193447	6,827410267	6,06788E-06	1,60003E-05
CPT1A	-0,595004585	6,908322341	6,2569E-06	1,64924E-05
COPG2	0,466366301	5,785989072	6,25971E-06	1,64934E-05
NOTCH3	0,764391445	7,884650054	6,35069E-06	1,67238E-05
TYW3	-0,361047157	4,762135573	6,35206E-06	1,67238E-05
CXXC5	0,542351533	5,125614205	6,37352E-06	1,67738E-05
BRCA2	0,695775303	3,085027688	6,43471E-06	1,69284E-05
HRSP12	0,575849846	4,48936722	6,49666E-06	1,70847E-05
VPS37B	-0,484862469	6,704895833	6,56181E-06	1,72428E-05
ZW10	0,391716996	4,743329853	6,56183E-06	1,72428E-05

TBC1D1	-0,543382229	6,056516563	6,64577E-06	1,74567E-05
SMO	0,949668784	4,176853367	6,65754E-06	1,74809E-05
COPZ1	0,417791324	8,092160479	6,73158E-06	1,76684E-05
PSMD10	0,441152141	6,25118531	6,88046E-06	1,80523E-05
PQBP1	0,467274812	6,260964712	6,93424E-06	1,81864E-05
LAMP1	-0,425328238	9,019196079	7,00682E-06	1,83697E-05
SOLH	0,436003068	5,845294737	7,03497E-06	1,84364E-05
SSB	0,364099971	7,0421462	7,05674E-06	1,84863E-05
TMEM39A	0,3884798	5,673380159	7,07092E-06	1,85164E-05
OSMR	0,886069444	7,625141885	7,16641E-06	1,87583E-05
MTA2	0,307996877	6,927795575	7,16882E-06	1,87583E-05
RNF113A	0,423943642	4,423834445	7,18661E-06	1,87977E-05
YTHDC1	-0,286511833	6,547171875	7,22239E-06	1,8884E-05
ZNF17	-0,384845558	2,797385469	7,30845E-06	1,91017E-05
PHPT1	0,654053087	6,597281833	7,32662E-06	1,91419E-05
IGBP1	0,458053165	6,682579003	7,37114E-06	1,92508E-05
DCBLD2	1,149819156	7,558693455	7,38894E-06	1,92899E-05
SV2A	1,140820621	3,615690515	7,63272E-06	1,99187E-05
RNF5	0,465835719	6,258507513	7,66157E-06	1,99863E-05
MYCBP2	-0,549060886	6,408079165	7,70014E-06	2,00793E-05
DNAJC2	0,482287327	5,383252682	7,78177E-06	2,02844E-05
PKN1	-0,352652816	7,561034749	7,924E-06	2,06472E-05
NDUFS5	0,544277293	7,208762868	8,0016E-06	2,08415E-05
HTRA2	0,395776892	5,337851848	8,01802E-06	2,08762E-05
DCAF5	-0,352091672	6,539729621	8,07279E-06	2,10108E-05
SLC25A5	0,594796982	9,048390387	8,09356E-06	2,10568E-05
RPS8	0,685699985	10,67099079	8,18542E-06	2,12877E-05
HACL1	-0,438764975	4,47349106	8,28977E-06	2,15509E-05
FAM58A	0,645535973	5,027737208	8,30092E-06	2,15716E-05
AKR1A1	0,444524178	7,538738662	8,33839E-06	2,16607E-05
GUF1	0,375544645	5,217342388	8,49645E-06	2,20629E-05
ZNF260	0,448644099	5,280389538	8,52187E-06	2,21205E-05
ATN1	-0,471546447	7,907217626	8,62512E-06	2,238E-05
ATF6B	0,374252182	7,274813026	8,86992E-06	2,30065E-05
CMAS	0,59208244	5,669096921	8,90837E-06	2,30974E-05
RPL19	0,476245289	10,54084927	8,92345E-06	2,31277E-05
FBXW5	0,447890153	7,193080834	8,94491E-06	2,31745E-05
DDX10	0,376334682	5,237802855	8,98743E-06	2,32758E-05
RBBP8	0,577436257	6,031952908	8,99738E-06	2,32928E-05
ADAM9	0,706024343	8,415889948	9,01842E-06	2,33384E-05
CAPN1	0,391020914	8,539462716	9,08241E-06	2,3495E-05
TAOK3	-0,415154823	6,185337623	9,10583E-06	2,35467E-05
PPP2R5D	0,314316444	6,236309367	9,18436E-06	2,37408E-05
PDGFA	-0,778157001	5,546454915	9,28023E-06	2,39795E-05
RAB11B	-0,314057726	7,155574662	9,28843E-06	2,39916E-05
CWC25	-0,356084081	4,880402787	9,50379E-06	2,45386E-05
UHRF1BP1L	-0,357289298	4,882837078	9,57486E-06	2,47127E-05
PABPC4	0,423550449	7,715160573	9,68728E-06	2,49934E-05
RPL37A	0,541066911	10,00669239	9,79491E-06	2,52615E-05
CISD3	0,462323081	5,71618896	9,80467E-06	2,52771E-05

ALDOC	0,904746858	5,464951877	9,84071E-06	2,53605E-05
RNF26	0,414824047	6,194724779	9,91612E-06	2,55452E-05
POP4	0,564485012	5,561933321	9,98192E-06	2,57049E-05
PANX1	0,486552325	5,109307814	1,02148E-05	2,62947E-05
DHRS4	-0,394042275	4,966069788	1,02404E-05	2,63507E-05
PWP2	0,36509746	5,521475652	1,03058E-05	2,65089E-05
ATP5E	0,495302748	8,30382195	1,03949E-05	2,6728E-05
ANAPC7	0,379889876	5,991646801	1,04156E-05	2,67712E-05
HIST2H2BE	0,90660894	5,297343004	1,04341E-05	2,68087E-05
GIT2	-0,311374673	6,166470909	1,06155E-05	2,72644E-05
NDUFV1	0,357589429	7,147330212	1,06368E-05	2,73089E-05
NAA50	0,419033486	7,448697567	1,07067E-05	2,74781E-05
SNTA1	0,844006427	5,190858371	1,0755E-05	2,75915E-05
SOD1	0,510679507	7,822331694	1,08426E-05	2,78058E-05
ZDHHC2	-0,635022264	6,052868448	1,0906E-05	2,79579E-05
MGAT4B	0,455645149	7,420826451	1,09578E-05	2,80802E-05
LOC650623	-0,577634245	2,694714126	1,10404E-05	2,82813E-05
USP28	0,50746363	5,196820058	1,10902E-05	2,83981E-05
S100A13	0,784651809	7,192539404	1,11098E-05	2,84376E-05
SPAG9	-0,461173041	7,45393783	1,1493E-05	2,94074E-05
DKFZP586I1420	-0,522634524	3,917237363	1,15664E-05	2,95842E-05
LACTB	-0,439475414	4,975144806	1,17774E-05	3,01125E-05
INO80D	-0,393457202	4,881057664	1,18846E-05	3,03752E-05
CRTC1	-0,501415962	4,698226959	1,19547E-05	3,05428E-05
TMEM33	0,366586604	7,001793514	1,19817E-05	3,06005E-05
PSMB7	0,420267897	7,046698646	1,20942E-05	3,08763E-05
NDUFA9	0,576402883	6,451380162	1,21771E-05	3,10761E-05
TMEM185B	0,478209137	5,292499103	1,22224E-05	3,11801E-05
ENTPD5	0,534057417	3,747426746	1,22504E-05	3,12398E-05
TUT1	0,343485156	5,202647031	1,22923E-05	3,1335E-05
SMAD1	0,615902116	5,525822336	1,2342E-05	3,1439E-05
PPL	-0,689181533	7,568408385	1,23423E-05	3,1439E-05
HIVEP1	-0,511641856	5,385054395	1,2419E-05	3,16225E-05
TRIM56	-0,598092376	4,856333698	1,24904E-05	3,17925E-05
POLR2F	0,698645034	5,808612926	1,25241E-05	3,18662E-05
GCLM	0,784559553	5,572111857	1,28115E-05	3,25854E-05
NR2C2	-0,425434835	5,872410004	1,28429E-05	3,26531E-05
GALNT1	0,467026235	7,317211363	1,29893E-05	3,30131E-05
BTN3A2	-0,587684913	6,771214456	1,30168E-05	3,30705E-05
WIBG	0,435582571	5,537448302	1,32049E-05	3,35361E-05
ZNRF2	0,460242606	5,410828737	1,32517E-05	3,36425E-05
KIAA0240	-0,424505618	5,402951721	1,32639E-05	3,36608E-05
SLC35D1	0,433685611	5,060801387	1,34343E-05	3,40806E-05
DLAT	0,374850174	6,231458246	1,34631E-05	3,41408E-05
ACLY	0,468493228	7,979532605	1,38633E-05	3,51427E-05
ARMC5	-0,415235179	4,376618424	1,40862E-05	3,56946E-05
NFKBID	-0,630548122	2,176903302	1,41058E-05	3,57309E-05
BOD1	0,528790403	5,777332628	1,43659E-05	3,63762E-05
ZNF680	0,60464321	4,334807228	1,47951E-05	3,74422E-05
INO80B	0,380582364	5,283909516	1,47979E-05	3,74422E-05

VEZT	0,365904763	6,952654798	1,50221E-05	3,7991E-05
DNAJB14	-0,35917289	4,747349552	1,50259E-05	3,7991E-05
THOC5	0,427831869	5,520176039	1,50513E-05	3,80409E-05
ABHD12	0,512953847	6,83075937	1,50669E-05	3,80663E-05
CIAPIN1	0,368673779	6,029224219	1,50867E-05	3,80994E-05
HIP1R	0,528236284	6,079109326	1,50911E-05	3,80994E-05
MTMR12	-0,338795458	6,566564374	1,52291E-05	3,84334E-05
MRPS28	0,594142371	5,02692101	1,52438E-05	3,84563E-05
TIAL1	0,300048912	6,471585149	1,52535E-05	3,84666E-05
DDX47	0,4408083	6,30967955	1,5451E-05	3,89502E-05
TMEM167B	-0,303431699	6,401263534	1,56032E-05	3,93193E-05
ZNF317	-0,307525154	5,265882434	1,56233E-05	3,93556E-05
EEF1D	0,457144988	9,166283282	1,56649E-05	3,94457E-05
TRIAP1	0,488850634	5,337664254	1,56749E-05	3,94564E-05
GALK1	0,520512398	5,058942291	1,58947E-05	3,99949E-05
UBE2A	0,461744627	6,900782766	1,59355E-05	4,00826E-05
CCDC66	-0,439988885	4,151165735	1,59533E-05	4,01125E-05
XRCC6	0,320342126	8,940425222	1,6201E-05	4,07205E-05
TCF7L1	-0,758408423	5,140118092	1,62395E-05	4,08022E-05
IL4R	-0,459849484	7,048094028	1,62589E-05	4,0836E-05
NT5C	0,460486547	4,99464197	1,62959E-05	4,09137E-05
MFSD1	-0,391697908	7,022392758	1,64046E-05	4,11716E-05
SMARCC1	0,423907913	7,344805873	1,66197E-05	4,1696E-05
IFT122	-0,469935568	5,12706352	1,66286E-05	4,17029E-05
TAF9	0,385044395	6,31015748	1,67213E-05	4,192E-05
CEP290	-0,462045517	4,451356314	1,68126E-05	4,21335E-05
RPL10A	0,630955267	9,983487593	1,69269E-05	4,24044E-05
MOSPD3	0,432632001	4,744556859	1,69392E-05	4,24195E-05
RNF166	-0,373573967	4,600093308	1,70701E-05	4,27317E-05
CGGBP1	-0,283664542	6,965433027	1,72718E-05	4,32207E-05
CFL1	0,35184012	10,17256471	1,73239E-05	4,33352E-05
LOC90834	-0,471616508	2,377673804	1,73774E-05	4,34458E-05
RPL29	0,640002099	10,13626555	1,73809E-05	4,34458E-05
TRIM37	0,439657304	5,441772094	1,74124E-05	4,35085E-05
ERBB2	0,639225362	8,519213166	1,7524E-05	4,37714E-05
LDHB	0,855153878	9,348089052	1,76446E-05	4,40567E-05
BCL7C	0,522865914	5,805848989	1,76621E-05	4,40841E-05
NCBP1	0,340344903	5,736771803	1,7778E-05	4,43572E-05
AP2S1	0,432439445	6,609784452	1,781E-05	4,44169E-05
ARMC8	-0,268836929	5,513289376	1,78149E-05	4,44169E-05
MAST4	-0,666859539	5,364306401	1,79253E-05	4,46758E-05
SPATA6	-0,604269972	2,912063461	1,79466E-05	4,47124E-05
YME1L1	0,29267585	7,818308963	1,81397E-05	4,51769E-05
FAT1	0,892281864	7,631686437	1,82214E-05	4,53638E-05
CRYZL1	-0,343496631	4,735591039	1,83411E-05	4,56454E-05
L2HGDH	0,351013856	4,883864665	1,83619E-05	4,56803E-05
MAPKAPK5	0,347080668	5,246844224	1,86967E-05	4,64962E-05
LYST	-0,507565689	5,622767827	1,87517E-05	4,66161E-05
FLNA	-0,614793823	10,72240124	1,88136E-05	4,6753E-05
SLC44A1	0,481359139	7,407823622	1,88275E-05	4,67704E-05

GNB5	0,352218105	5,228728226	1,88755E-05	4,68725E-05
SUMO1	0,380473822	7,378639894	1,89259E-05	4,69805E-05
PSENEN	0,721636872	6,76860218	1,91667E-05	4,7561E-05
NUDCD2	0,448129968	5,017056556	1,91974E-05	4,76198E-05
CIB1	0,565394503	7,801326523	1,93516E-05	4,79848E-05
KIAA0020	0,439287915	5,206289276	1,93731E-05	4,80208E-05
SPCS1	0,3895949	7,045385643	1,94345E-05	4,81554E-05
DSTN	-0,468300293	9,465720889	1,96183E-05	4,85933E-05
GNPAT	0,344186634	6,436475897	1,97277E-05	4,88466E-05
SMG7	0,369936511	7,091091596	1,97768E-05	4,89502E-05
RER1	0,397289574	7,598336191	1,98651E-05	4,91509E-05
PCOLCE	0,875997623	6,787472395	1,98912E-05	4,91977E-05
HACE1	-0,404414692	3,991745632	2,00104E-05	4,94746E-05
FAM50A	0,428993906	6,744844975	2,00844E-05	4,96396E-05
TUBA1A	-0,499153196	8,901198414	2,03937E-05	5,03858E-05
AASDHPPT	0,31276174	5,856242999	2,09711E-05	5,17934E-05
GDI2	0,398828241	8,66765395	2,10976E-05	5,20872E-05
ZNF800	-0,318311521	4,916160061	2,12854E-05	5,25317E-05
FKBP14	0,411939242	4,201376026	2,1402E-05	5,28004E-05
ERCC3	0,267246689	6,145050149	2,14728E-05	5,29558E-05
ZNF706	0,428196441	6,55136543	2,15018E-05	5,30082E-05
TPST2	-0,416601443	5,640681874	2,16795E-05	5,34255E-05
ZFP36L2	-0,486876811	8,076840943	2,16867E-05	5,34255E-05
PDPK1	-0,414976426	6,450304443	2,18641E-05	5,38429E-05
BUD31	0,574486977	6,574469859	2,19182E-05	5,39566E-05
SRPK2	0,355532395	5,928308953	2,21323E-05	5,4464E-05
TCP1	0,393683646	7,676307916	2,21642E-05	5,45231E-05
HIP1	-0,648281658	6,808804043	2,22713E-05	5,47666E-05
MED6	0,353704316	4,957827094	2,23245E-05	5,48778E-05
MLLT6	-0,460957759	7,868176852	2,23524E-05	5,49266E-05
NANP	0,458100789	3,896042599	2,26612E-05	5,56652E-05
SLK	-0,44591988	6,967191484	2,27096E-05	5,57642E-05
CHSY1	-0,516245461	6,213404774	2,28063E-05	5,59665E-05
SH2D2A	0,816065656	3,249961309	2,28085E-05	5,59665E-05
ANKRD13A	-0,377792631	6,80793538	2,31105E-05	5,66871E-05
RPL18A	0,661115927	10,24304775	2,31274E-05	5,67083E-05
PHB2	0,567207347	8,022765914	2,32403E-05	5,69648E-05
C5orf51	0,394948082	5,933564156	2,34273E-05	5,73833E-05
ATP5C1	0,4930546	7,655517109	2,34279E-05	5,73833E-05
ADAM19	0,84678115	5,307504071	2,34665E-05	5,74571E-05
MPHOSPH10	0,285927751	5,71423151	2,36843E-05	5,79696E-05
RFT1	0,300858533	5,321843433	2,37644E-05	5,81448E-05
NUTF2	0,456298999	6,990045432	2,38371E-05	5,83018E-05
SUZ12	0,417586005	6,119947889	2,40256E-05	5,87417E-05
GOSR2	0,363498511	6,349670375	2,40579E-05	5,87995E-05
EHD4	-0,382582248	6,738650283	2,4309E-05	5,93919E-05
SPEN	-0,499402144	6,750835283	2,45998E-05	6,0081E-05
DSC2	0,757239235	6,09171105	2,466E-05	6,02065E-05
SPIN1	-0,262047461	6,886681847	2,47642E-05	6,04391E-05
DCAF11	-0,293120782	6,926893863	2,50684E-05	6,11409E-05

LYSMD3	-0,375917179	5,647635155	2,50697E-05	6,11409E-05
MRPL49	0,512868782	6,935545664	2,5231E-05	6,15124E-05
AP3S1	0,371645707	6,178063756	2,52635E-05	6,15586E-05
KTN1	-0,410045919	8,151772544	2,5268E-05	6,15586E-05
TCF7L2	-0,428424645	5,645219799	2,54538E-05	6,1989E-05
DDHD1	-0,469686395	4,956038172	2,55756E-05	6,22636E-05
RNF6	-0,332658141	5,72467796	2,61147E-05	6,35323E-05
TOMM20	0,418928253	8,542334708	2,61154E-05	6,35323E-05
ACTR1A	-0,263496578	7,515255901	2,62776E-05	6,3904E-05
CASK	0,474966441	6,362151768	2,63363E-05	6,40114E-05
TSPYL4	-0,454117476	5,095256662	2,63405E-05	6,40114E-05
SMCR8	-0,42806746	5,849079675	2,67742E-05	6,5042E-05
PTK7	0,594135344	7,928296861	2,68002E-05	6,50821E-05
RGL1	-0,612493229	6,249146794	2,69259E-05	6,53641E-05
ARF5	0,478525507	7,542758869	2,69657E-05	6,54373E-05
ANAPC5	0,372873912	7,248270602	2,71011E-05	6,57426E-05
OXSM	0,471181737	3,554169292	2,72844E-05	6,61575E-05
FAM82B	-0,307773734	5,623826225	2,72915E-05	6,61575E-05
MBOAT1	0,50897672	5,530657754	2,74604E-05	6,65431E-05
PSIP1	-0,501745017	6,35432856	2,75376E-05	6,67065E-05
TTYH2	-0,615071769	3,723705587	2,75556E-05	6,67263E-05
RPA2	0,40238882	6,190658178	2,76804E-05	6,69967E-05
PDSS1	0,506482364	2,822121983	2,76869E-05	6,69967E-05
TRAF3IP1	-0,41022814	5,255735753	2,76985E-05	6,7001E-05
RHOT2	0,349380245	6,253048261	2,77104E-05	6,70061E-05
CDK2AP2	0,589918573	7,052775537	2,8161E-05	6,80716E-05
UFC1	0,440968314	7,487154914	2,81808E-05	6,80952E-05
PSME4	0,397657177	6,86955955	2,84583E-05	6,87373E-05
CINP	0,404525479	4,977335629	2,84666E-05	6,87373E-05
ERCC5	-0,397314718	6,13197701	2,84893E-05	6,87677E-05
USP30	-0,311929205	4,501752436	2,8642E-05	6,91119E-05
NT5C3	0,421970565	5,373356001	2,87096E-05	6,92504E-05
ARFGAP2	-0,251940508	6,978197132	2,93374E-05	7,07397E-05
CPNE1	0,51546716	7,324719979	2,94723E-05	7,10397E-05
SLC10A3	0,393737944	5,898898255	2,97969E-05	7,17968E-05
RBM45	0,209014792	3,599907164	2,9922E-05	7,20728E-05
TOR1AIP1	-0,315098567	6,994503792	2,99982E-05	7,22308E-05
PYGB	0,66748052	8,424161396	3,02333E-05	7,27712E-05
DSTYK	-0,411282757	5,534483855	3,05273E-05	7,3453E-05
LMNA	-0,538425073	9,35213178	3,05861E-05	7,35684E-05
RCN2	0,501420407	6,666775555	3,09002E-05	7,42979E-05
PSMC3	0,304957045	7,302898389	3,09382E-05	7,4363E-05
PAN3	-0,382428156	5,602608358	3,09769E-05	7,44298E-05
IQGAP1	-0,367774215	9,155161728	3,10178E-05	7,45018E-05
NCOR1	-0,45463045	6,983856791	3,12566E-05	7,50489E-05
PHC3	-0,337112095	6,460748121	3,1319E-05	7,51612E-05
EPC1	-0,358547696	4,837124061	3,13255E-05	7,51612E-05
ZNF117	0,870153239	6,622032386	3,14565E-05	7,54492E-05
NINJ2	-0,753325447	4,927138045	3,15267E-05	7,55908E-05
MYO9B	-0,43290751	7,020576835	3,18219E-05	7,6272E-05

ROCK2	-0,511290832	6,051378774	3,19077E-05	7,64506E-05
SNRNP200	0,417655648	8,557288811	3,19476E-05	7,65194E-05
STAT6	-0,359844404	8,210395989	3,22E-05	7,70969E-05
PHF21A	-0,355390165	5,641827036	3,24504E-05	7,76691E-05
PGP	0,463016834	4,816830044	3,25579E-05	7,7899E-05
UBE2D1	0,376735002	5,429575063	3,25838E-05	7,79338E-05
MMADHC	0,373410739	6,894824959	3,29766E-05	7,88456E-05
HDLBP	0,381124462	9,76425183	3,32416E-05	7,94332E-05
BIVM	-0,457654872	4,46814428	3,32457E-05	7,94332E-05
KRAS	0,534445623	6,717360858	3,33545E-05	7,96653E-05
MET	1,146635536	8,554128281	3,34617E-05	7,98933E-05
SUGT1	0,381018659	5,432652534	3,37619E-05	8,05818E-05
ATP1B3	-0,460102291	7,164623255	3,38584E-05	8,07838E-05
NOD1	-0,610776876	5,492738945	3,45586E-05	8,24257E-05
CHIC1	-0,449928129	4,001503952	3,46754E-05	8,26662E-05
RALY	0,327858634	8,076961825	3,46837E-05	8,26662E-05
PI4KA	-0,409120778	6,737310645	3,47532E-05	8,28029E-05
TBC1D10B	0,312779884	6,460492409	3,48237E-05	8,29419E-05
CNN3	-0,516376331	8,194407422	3,52865E-05	8,40148E-05
ISCU	-0,347977472	6,823001082	3,54568E-05	8,4391E-05
RNF4	0,249278335	6,545456128	3,55918E-05	8,46825E-05
EIF4E	0,370245439	6,720321532	3,58831E-05	8,53459E-05
OPA1	0,426766318	6,500799314	3,60373E-05	8,56827E-05
CCNG2	0,536809675	6,417087043	3,6074E-05	8,57403E-05
ETFA	0,452353086	6,732920039	3,6243E-05	8,61117E-05
NAA20	0,482353878	6,444654349	3,62752E-05	8,61584E-05
PMPCA	0,318113057	6,114247786	3,62901E-05	8,61638E-05
USP13	-0,52374218	5,591863373	3,6305E-05	8,6169E-05
TAP1	0,736120384	8,247961122	3,63252E-05	8,6187E-05
MEGF9	-0,616384481	7,03940187	3,63512E-05	8,62188E-05
CYP7B1	0,701809935	3,147480409	3,65721E-05	8,67125E-05
AMPD3	0,537497509	4,791768078	3,67452E-05	8,70926E-05
GABPA	-0,329723638	5,824591658	3,67927E-05	8,7175E-05
ACBD3	0,349364361	7,078757677	3,69932E-05	8,76196E-05
CCDC88B	-0,490015969	4,950959814	3,73511E-05	8,84365E-05
BCL2L11	0,408521914	5,692354389	3,77009E-05	8,92336E-05
DAXX	0,253063057	6,4152089	3,77374E-05	8,92892E-05
BRD9	0,426866071	5,945830552	3,83163E-05	9,06275E-05
SLC35C1	0,579938083	6,23170732	3,89528E-05	9,21011E-05
MUS81	0,313969728	5,330309679	3,90637E-05	9,2331E-05
GPR125	0,518059485	5,40583882	3,90978E-05	9,23798E-05
EI24	0,344424066	7,452547158	3,92068E-05	9,26051E-05
ARHGEF1	-0,400872861	7,175663219	3,92356E-05	9,2641E-05
GMPR	-0,627310787	3,928691382	3,97037E-05	9,37138E-05
RBM33	-0,358952376	6,003561109	4,019E-05	9,4829E-05
B2M	-0,579358659	12,53465032	4,03088E-05	9,50764E-05
CANX	0,374091467	10,24013978	4,03373E-05	9,51107E-05
SRPR	0,323221306	8,17581749	4,0732E-05	9,60066E-05
RHOB	-0,637297916	9,019754786	4,07454E-05	9,60066E-05
CREB3L2	0,487961635	7,57595129	4,07868E-05	9,60709E-05

RPL35	0,709440111	9,547917659	4,09507E-05	9,64236E-05
DDX18	0,358804814	7,106196313	4,10555E-05	9,6637E-05
RLTPR	0,956225167	2,734879024	4,1166E-05	9,68383E-05
HOMER1	-0,596826166	4,168951715	4,11694E-05	9,68383E-05
LSM14B	0,423728539	6,088294595	4,14939E-05	9,75679E-05
TMEM70	0,551394301	5,577357783	4,16017E-05	9,77875E-05
OBFC1	-0,472018462	5,975952283	4,20538E-05	9,88162E-05
FOXK2	0,366287435	6,665655948	4,21789E-05	9,90762E-05
FCGRT	-0,513972112	8,199822794	4,26921E-05	0,000100247
PDLIM7	0,612003879	6,149067272	4,2775E-05	0,000100397
RPIA	0,384602259	5,185918872	4,27855E-05	0,000100397
CLEC16A	-0,39463414	5,853016647	4,28445E-05	0,000100501
DHX32	0,471108503	6,042003904	4,31947E-05	0,000101288
MRPL1	0,475618089	4,808665638	4,32839E-05	0,000101462
PAPOLG	0,319697099	4,662360764	4,3508E-05	0,000101952
MCAM	-0,628911499	7,225523885	4,35674E-05	0,000102056
ZFYVE16	-0,298767911	6,06014339	4,35893E-05	0,000102073
MRPL32	0,517249098	5,818961699	4,37398E-05	0,00010239
DAD1	0,447544245	7,639239775	4,39812E-05	0,00010292
USP5	0,387673766	6,969217413	4,41179E-05	0,000103204
UBE2E3	0,400631762	6,26785424	4,44586E-05	0,000103965
ZCCHC17	0,418723904	5,760952299	4,45821E-05	0,000104219
GSK3B	-0,378550674	6,422551094	4,46196E-05	0,00010427
CIAO1	0,276275887	7,064997205	4,46461E-05	0,000104297
NEK11	-0,656909789	3,985455591	4,47325E-05	0,000104463
SFN	0,893245113	7,534815563	4,50039E-05	0,00010506
PCCB	0,385634052	6,283305639	4,50621E-05	0,00010516
PTPN11	-0,379450029	7,454914724	4,50914E-05	0,000105193
NR2F2	-0,520974398	6,228469985	4,53249E-05	0,000105701
RECQL5	0,439669474	5,110915807	4,53789E-05	0,000105791
RPL28	0,582699829	9,91786075	4,57225E-05	0,000106552
MRC2	-0,608831059	7,678177246	4,57364E-05	0,000106552
ECD	0,25138686	5,280237846	4,65641E-05	0,000108443
SUPT4H1	0,411339963	6,747790137	4,66706E-05	0,000108654
PDCD11	0,46761317	6,070574602	4,69056E-05	0,000109164
APBA3	0,325332339	4,083847786	4,7003E-05	0,000109353
VPS53	-0,367724952	6,236535832	4,71764E-05	0,000109719
HLA-DPB1	-0,730685989	9,604589244	4,74422E-05	0,000110299
CHD3	-0,531484371	7,630329497	4,74847E-05	0,000110361
UTP14C	-0,354889121	6,048483208	4,75607E-05	0,0001105
C1orf174	0,391611996	5,144008828	4,77529E-05	0,000110908
RPS3	0,68716711	11,03603744	4,77776E-05	0,000110928
AXIN1	0,326059677	5,786477552	4,79859E-05	0,000111374
STK16	0,448344846	5,666124422	4,84585E-05	0,000112432
DRG1	0,436882826	6,171350999	4,84907E-05	0,000112469
ATHL1	1,293577335	6,856123876	4,8747E-05	0,000113025
KIAA1671	-0,571912963	6,607458643	4,89036E-05	0,000113349
NIPA2	0,299558631	6,36292965	4,91727E-05	0,000113934
MRPS18B	0,416147964	6,736962578	4,96713E-05	0,000115051
TMEM209	0,323386721	5,598682281	4,99483E-05	0,000115653

POLD1	0,407315213	5,310108135	5,01507E-05	0,000116082
UQCRC1	0,371601057	7,578506499	5,01849E-05	0,000116101
VDAC2	0,413746075	7,73560216	5,01928E-05	0,000116101
RNF130	-0,385664311	7,171198726	5,03543E-05	0,000116435
SRC	0,482471153	6,829537432	5,03993E-05	0,000116499
STAM	-0,311797911	5,479042172	5,09927E-05	0,000117831
SLC2A4RG	0,511473395	6,45519332	5,16866E-05	0,000119394
SOX9	1,270607542	5,547385975	5,17403E-05	0,000119478
ZDHHC17	-0,32449204	5,16269008	5,20148E-05	0,000120071
ATP5B	0,459407162	9,523434467	5,233E-05	0,000120757
RCL1	-0,350405275	4,663072898	5,27946E-05	0,000121788
LTBR	0,561879312	7,540758667	5,42907E-05	0,000125197
UCKL1	0,431748408	5,388265464	5,49968E-05	0,000126783
ZNHIT1	0,600909123	6,865375392	5,52052E-05	0,00012722
STARD10	0,765440095	7,35270748	5,53973E-05	0,00012762
SIRT6	0,346338526	4,380799074	5,55044E-05	0,000127823
RAB11FIP3	-0,478685902	5,798787211	5,61354E-05	0,000129233
CXCL9	1,365357656	7,088210335	5,61614E-05	0,000129249
TGM2	-0,647779625	9,641862808	5,62926E-05	0,000129507
FTSJ3	0,354092912	6,322510443	5,63201E-05	0,000129527
LATS1	-0,444395207	4,462881517	5,63628E-05	0,000129581
MAP3K6	-0,546448507	5,990886986	5,68023E-05	0,000130548
DNM2	-0,337809339	7,915619497	5,71852E-05	0,000131383
ALKBH4	0,351733148	4,094255344	5,88335E-05	0,000135125
RPL27	0,667067274	10,07671371	5,89905E-05	0,00013544
DYRK3	-0,589232423	3,852996735	5,94862E-05	0,000136532
SNAP23	-0,323291925	6,822057978	5,97951E-05	0,000137195
CUTA	0,39714935	7,115787825	5,99832E-05	0,00013758
FAM109B	0,440434983	4,955631075	6,00517E-05	0,000137691
INTS4	0,329287489	5,148478146	6,01247E-05	0,000137812
TECPR2	-0,399071147	5,200486679	6,03224E-05	0,000138218
ARID5B	-0,469855406	6,511252964	6,10138E-05	0,000139756
ITGB3	1,166939788	4,572809722	6,13149E-05	0,000140398
TSEN15	0,442306378	5,809217115	6,14339E-05	0,000140624
FAM43A	-0,829924265	4,233016982	6,17164E-05	0,000141223
SURF2	0,462882301	4,166207201	6,23392E-05	0,0001426
MGC57346	0,505201719	3,743856387	6,31038E-05	0,000144301
RPL10	0,54582999	11,41847771	6,33722E-05	0,000144866
ZNF16	0,337767797	3,7626975	6,38029E-05	0,000145802
WRNIP1	0,33633539	6,265447383	6,48787E-05	0,00014821
LLPH	0,440259254	5,877302265	6,58648E-05	0,000150413
RIT1	0,518021488	6,302365115	6,62315E-05	0,000151199
CALD1	-0,526447716	8,163137824	6,63651E-05	0,000151454
CCDC25	-0,31971569	5,555664427	6,64012E-05	0,000151485
C6orf106	-0,345879749	7,881743074	6,74309E-05	0,000153783
COMMD2	-0,371357694	5,934695286	6,81819E-05	0,000155444
TRPM7	-0,351780447	5,734320187	6,93902E-05	0,000158146
MAPK3	-0,346965305	6,754702315	6,94937E-05	0,000158329
TMEM9B	-0,388514366	6,793612058	6,97551E-05	0,000158871
TIMP2	-0,544121832	9,10438015	7,04401E-05	0,000160378

OVCA2	0,403714885	3,106174117	7,08999E-05	0,000161371
ACOT2	-0,419375848	5,255592148	7,11195E-05	0,000161817
ICMT	0,334520819	7,355223561	7,17982E-05	0,000163306
NFIC	-0,546274524	5,867160223	7,20821E-05	0,000163897
RHOC	0,581318437	8,436199963	7,2189E-05	0,000164086
NENF	0,532466382	6,524140712	7,22144E-05	0,000164089
ZBTB38	-0,378279922	7,072479428	7,22517E-05	0,000164119
ARPC3	0,458152871	8,64952362	7,24007E-05	0,000164402
GFM2	0,368419718	5,328593926	7,28956E-05	0,000165471
C14orf169	0,38930932	4,391006182	7,29447E-05	0,000165524
ZNF3	0,330867775	5,50460822	7,29674E-05	0,000165524
PLXNA3	0,581824564	6,048328676	7,36323E-05	0,000166977
TMEM65	0,539831733	5,216187146	7,41842E-05	0,000168172
TBC1D14	-0,330334791	6,691051576	7,4228E-05	0,000168216
MESDC2	0,285762472	6,615940114	7,4678E-05	0,000169179
SNRPB2	0,374148307	6,213844629	7,58005E-05	0,000171609
INPP1	-0,501833093	4,857103665	7,5801E-05	0,000171609
PFAS	0,435850619	4,882927506	7,59669E-05	0,000171928
HCST	-0,682960725	4,430473601	7,60959E-05	0,000172163
YIPF5	0,32684914	6,623547059	7,62335E-05	0,000172417
WIPI2	0,286198384	6,878480541	7,65755E-05	0,000173133
OTUB1	0,304715933	7,027120276	7,66021E-05	0,000173135
PPID	0,308531832	5,36988106	7,76543E-05	0,000175455
LMBR1L	0,368265548	5,076892815	7,79703E-05	0,000176111
C12orf44	0,42469157	6,142361527	7,87121E-05	0,000177728
CRAMP1L	-0,38983056	5,451202146	7,96008E-05	0,000179675
MAGOH	0,382040529	5,279834444	8,07777E-05	0,000182271
OXSR1	-0,292245105	6,15987381	8,10142E-05	0,000182744
USP10	0,321365196	6,599948846	8,11037E-05	0,000182829
EIF5	-0,290626503	8,151805246	8,11053E-05	0,000182829
RARRES2	-0,719809474	7,097006979	8,13587E-05	0,000183339
IMMT	0,276004661	6,817013701	8,17158E-05	0,000184083
MT1F	0,857477258	3,715862722	8,23096E-05	0,00018536
NDUFA8	0,414064831	5,760305239	8,23857E-05	0,00018547
RPL27A	0,612339309	10,68295846	8,25952E-05	0,00018588
KHDRBS1	0,253629343	7,850904364	8,31893E-05	0,000187155
LSM10	0,466896309	5,229768162	8,52246E-05	0,000191671
GXYLT2	0,766549637	2,7232755	8,63389E-05	0,000194113
CNPY3	0,350352354	6,854146708	8,64735E-05	0,000194352
BDP1	-0,402361392	5,563509428	8,65277E-05	0,000194364
SCO1	0,299284017	4,702160434	8,65361E-05	0,000194364
SEC31A	0,285778589	8,243853083	8,69845E-05	0,000195307
KDELC1	0,729057172	3,120967702	8,76699E-05	0,000196781
NUFIP1	0,333929013	3,778545505	8,91489E-05	0,000200035
RPS6KB1	0,345193525	5,835196336	8,93167E-05	0,000200346
CCDC115	-0,301805129	6,052465075	8,97548E-05	0,000201262
MAP2K7	-0,275238599	5,547665395	9,00873E-05	0,000201941
CDK12	0,399106621	6,428317503	9,0487E-05	0,000202771
ZNF280C	0,409384953	3,372115769	9,06045E-05	0,000202967
MRPL18	0,418392295	5,942097298	9,09591E-05	0,000203695

ITGB5	0,490079794	7,746533135	9,09996E-05	0,000203719
MPEG1	-0,691558944	6,478443791	9,12161E-05	0,000204136
DUSP11	0,3851952	5,284137108	9,12649E-05	0,000204178
SLC30A5	0,295609937	6,439770139	9,13965E-05	0,000204406
GRPEL1	0,352910121	5,557227617	9,15959E-05	0,000204784
FNIP2	-0,668535005	6,357416454	9,18513E-05	0,000205288
NDUFA13	0,547611447	7,623646208	9,20505E-05	0,000205666
CS	0,360930958	8,014852819	9,29758E-05	0,000207665
TMEM41A	0,411693812	6,069269241	9,31685E-05	0,000208027
TP53	0,529440475	6,42079009	9,4016E-05	0,000209851
CENPQ	0,519512878	3,424337939	9,45628E-05	0,000211003
RIPK1	-0,266486099	6,087507071	9,46106E-05	0,00021104
NBPF14	-0,480640523	5,072484195	9,49185E-05	0,000211658
RPLP1	0,648323145	10,90512245	9,50162E-05	0,000211806
ZFAND2A	0,674292847	4,650362118	9,50734E-05	0,000211865
MAPK1IP1L	-0,30677543	7,197824133	9,52315E-05	0,000212148
TRNAU1AP	0,581595102	5,151331121	9,56458E-05	0,000213001
TBC1D13	-0,355831354	5,597697627	9,67213E-05	0,000215261
RBL1	0,436249031	3,772839639	9,6724E-05	0,000215261
C11orf75	0,464641127	5,273347215	9,78866E-05	0,000217778
SIL1	0,450231219	6,632592112	9,79252E-05	0,000217793
STYX	0,335669028	5,488702931	9,80898E-05	0,000218087
CRAT	0,419101155	5,745650722	9,82325E-05	0,000218334
SERINC3	-0,309824991	7,409001623	9,86342E-05	0,000219155
TMEM138	0,315552659	5,215004575	9,87012E-05	0,000219232
PHF14	0,358070615	5,817791391	9,88968E-05	0,000219556
UBIAD1	0,248435426	5,127861858	9,89111E-05	0,000219556
ZNF267	0,410511418	4,809488239	9,92782E-05	0,000220299
NFKBIL1	0,377379029	4,737499247	9,93593E-05	0,000220407
MYO5A	-0,454122612	5,766283122	9,98941E-05	0,000221521
CUL2	0,312995077	5,718829159	0,000100268	0,000222278
FOSL2	-0,583827422	7,188614903	0,000100706	0,000223176
TTC19	-0,370909296	6,076442868	0,000101451	0,000224754
RAP2B	0,487699091	6,460914719	0,000101802	0,000225436
LRRC8D	0,383309848	5,31537143	0,000101825	0,000225436
LASP1	0,412529649	8,841020615	0,00010251	0,00022688
ATMIN	0,24548134	6,507162107	0,000102821	0,000227495
C14orf2	0,458815227	6,308095122	0,000103256	0,000228382
CTNNBIP1	-0,466986547	6,416441797	0,000103988	0,000229925
TET2	-0,386616992	5,234117276	0,000104021	0,000229925
PPP1R10	-0,407708724	6,8862427	0,000104182	0,000230205
NBEAL2	-0,55188572	6,710906689	0,000105095	0,000232149
FLT3LG	-0,462759493	3,165772177	0,000105357	0,000232652
CDR2	0,400476373	5,539708241	0,000106502	0,000235105
MAN2A1	0,63995695	5,71503898	0,000107718	0,000237711
PAG1	-0,554393354	6,072782576	0,000110628	0,000244054
IL10RA	-0,597536053	6,022645149	0,000111958	0,00024691
GPD2	0,32236361	6,532803181	0,000112046	0,000247023
ESYT1	-0,286621045	7,689927548	0,000112603	0,000248171
UBE2R2	0,313070169	7,032068629	0,000113891	0,000250929

RRP8	0,321032542	4,443430162	0,000114435	0,000252046
PRR24	-0,417933931	3,260995108	0,000115424	0,000254141
TBC1D22A	-0,284286927	5,447478779	0,000115785	0,000254854
NAA16	-0,387075092	4,085419412	0,000116057	0,000255344
APLP2	-0,465470042	10,75667151	0,000116082	0,000255344
GBA2	-0,351497072	5,975646995	0,000116449	0,000256067
LIMD2	0,631199433	5,327820883	0,000116576	0,000256264
HARS2	0,311810285	5,401767924	0,000117669	0,000258566
BTG1	-0,447702586	8,076278076	0,000117699	0,000258566
STRAP	0,457291129	7,371904498	0,000119374	0,000262161
ZNF768	0,37263176	5,738566821	0,000120007	0,000263468
PCIF1	-0,235403456	6,143097919	0,000120462	0,00026438
PSMC5	0,287881485	7,321550462	0,000120529	0,000264442
ABCC10	0,371151212	5,273353772	0,000120767	0,000264879
SPSB1	0,574235476	6,31660788	0,000120977	0,000265254
ZNF710	0,696345433	5,332684763	0,000121102	0,000265443
ZMPSTE24	0,324087647	6,965270216	0,000121384	0,000265956
IPO7	0,3683106	8,196419007	0,000121414	0,000265956
SEC62	-0,25830704	8,221565786	0,000121497	0,000266052
GIMAP2	-0,568228455	4,467081209	0,000122377	0,000267895
HIPK2	-0,561659257	7,062349265	0,00012394	0,000271228
MAGT1	0,420788016	7,916449593	0,000124011	0,000271296
TNPO1	0,292386345	7,334562705	0,00012524	0,000273896
BAG4	0,502452388	3,982927518	0,000125755	0,000274864
MAP4K2	-0,428327574	4,998023727	0,000125763	0,000274864
GUSBP3	-0,360229333	3,360464688	0,000125963	0,000275213
KLHL5	0,511041858	6,06794013	0,000126035	0,000275282
SGK196	0,725237598	1,666092448	0,000126118	0,000275375
POT1	0,441136628	5,129693255	0,000126257	0,000275592
IFRD2	0,402643694	6,1498818	0,000127436	0,000278075
UBE2J2	0,329715848	5,967246299	0,000127995	0,000279205
GNB2L1	0,459577943	10,57114208	0,000128548	0,000280323
PXN	-0,408390858	7,654490763	0,000128634	0,000280421
PTPLAD1	0,466833754	7,530606358	0,000128681	0,000280434
THYN1	0,454674576	5,577510326	0,00012961	0,000282368
NPRL3	0,343419947	5,909048223	0,000130307	0,000283796
RNF7	0,394029456	6,500243254	0,0001322	0,000287827
GFM1	0,296046673	6,171169597	0,000132558	0,000288514
PDCD4	-0,403010986	6,544324383	0,00013311	0,000289622
MLF2	0,41872481	7,940848188	0,000134076	0,00029163
DAAM1	-0,424569001	5,083095295	0,00013466	0,000292808
CABIN1	-0,399872687	6,702759955	0,000135604	0,000294766
FMNL2	-0,448001957	6,506431903	0,000136	0,000295533
ECHDC2	-0,48424347	5,638670612	0,000136427	0,000296366
TCEAL3	-0,420212523	4,853048681	0,000139201	0,000302297
CPE	1,062935378	7,688939529	0,000139721	0,00030333
POLD4	0,454143371	6,839197334	0,000139931	0,00030369
RUFY2	-0,250543536	4,458532162	0,000141591	0,000307193
ARID1B	-0,374314254	6,59676508	0,000142287	0,000308606
TMEM87A	-0,411960973	7,589183419	0,000143278	0,00031062

WDR5	0,364842484	5,77660089	0,000143307	0,00031062
ZFR	0,217823358	7,080626037	0,000143377	0,000310674
TCEAL4	-0,362352596	6,484771888	0,000143664	0,000311196
HAUS4	0,466567323	6,480492119	0,000143821	0,000311437
ARL1	0,379794867	7,230208683	0,000146724	0,000317622
MAGEH1	-0,491598998	5,205614021	0,000146901	0,000317885
TXNL4A	0,40145845	5,638768162	0,000146939	0,000317885
PACSIN2	-0,448076956	6,728021064	0,00014773	0,000319497
C6orf89	-0,253704267	7,430107452	0,000148712	0,000321517
NCOR2	-0,431016237	8,462522095	0,000148815	0,000321639
ARFGEF1	0,359151553	6,865844996	0,000149962	0,000324014
NDUFV2	0,463181452	7,126541441	0,000150266	0,000324569
SLC25A24	-0,435735346	6,341056867	0,00015065	0,000325296
KIAA1143	-0,249922867	6,088867772	0,000150972	0,000325887
HES4	0,757572408	2,867266926	0,000153179	0,000330547
CDC42SE1	0,354692248	7,74739355	0,000153355	0,000330822
ATP13A1	0,335445515	6,615673468	0,000155799	0,000335987
PIN1	-0,31338034	5,528712343	0,000156	0,000336315
ABHD4	0,415398425	6,087787367	0,000156077	0,000336375
IFI6	0,917858043	8,47709303	0,000156134	0,000336391
POLR3A	0,397291405	4,951204095	0,000156514	0,000337104
INF2	-0,454348644	7,285457713	0,000156837	0,000337643
GPCPD1	-0,560863629	6,824388247	0,000156864	0,000337643
ZSWIM6	-0,396239366	5,076790686	0,000157525	0,000338959
PHF3	-0,354290408	6,689752051	0,000157705	0,00033924
SERINC5	-0,571646329	4,003936042	0,000158839	0,000341572
PTP4A2	0,310698196	7,677080852	0,000159077	0,000341975
RRN3	0,277113391	6,060405673	0,000160947	0,000345887
PCBD1	0,410605387	6,727163206	0,000161262	0,000346453
TMEM167A	0,334150417	7,448941278	0,000161792	0,000347483
TMEM41B	-0,373611789	6,57946596	0,000162334	0,000348538
RAF1	-0,194119029	7,04468764	0,000162397	0,000348564
FAM122B	0,331074731	5,571874692	0,000162486	0,000348644
ZNF544	0,426220603	5,026659854	0,000162996	0,000349629
IFT74	-0,373703311	3,974056336	0,000164966	0,000353737
TMEM141	0,742362458	6,492628449	0,00016506	0,000353737
CDK5RAP3	0,392012736	6,812664581	0,000165067	0,000353737
THAP4	0,27630707	6,036823667	0,000165772	0,000355137
DYNLT1	0,503514843	7,00010525	0,00016606	0,000355642
HP1BP3	-0,257988436	7,881401752	0,000166403	0,000356264
MRPL20	0,353941893	6,441041812	0,000166631	0,000356641
DHX15	0,28319346	7,422045033	0,000167393	0,000358158
MMAB	-0,40724327	5,51196287	0,00017044	0,000364563
KIAA2013	0,286384368	6,600101477	0,000170845	0,000365315
SEPHS1	0,311402614	6,056595272	0,000172978	0,000369761
TIMM13	0,365172213	5,900299277	0,000173315	0,000370364
PIK3C2B	-0,548377579	5,855902928	0,00017376	0,0003712
NANS	0,447081771	6,291792616	0,000174233	0,000372094
RPL18	0,50458957	9,763998646	0,000175071	0,000373766
EEF2K	-0,345885114	6,320527824	0,000175187	0,000373896

PPP3CA	-0,35067295	6,73457768	0,000176562	0,000376714
CHCHD3	0,338533842	6,087410424	0,000178586	0,000380913
SMNDC1	0,274679627	5,57322663	0,000178876	0,000381413
BCAS3	-0,38035612	5,326196145	0,000179318	0,000382228
NXF1	-0,306714961	6,440420565	0,000179371	0,000382228
RAD1	0,3597707	5,993813435	0,000179485	0,000382352
TK2	-0,403394399	5,8341978	0,000179621	0,000382523
EIF4A1	0,328902794	9,603146097	0,000179739	0,000382654
SDHAF2	0,311207124	5,712332716	0,00018069	0,000384558
RFWD2	0,284387277	6,196744952	0,000181841	0,000386887
NUP93	0,282758036	5,912018532	0,000184453	0,000392322
SPAG7	-0,343653648	5,822124174	0,000185401	0,000394215
ZNF621	-0,31418885	5,144483192	0,000185654	0,00039463
WTIP	-0,627922538	4,039243131	0,000187172	0,000397734
PTPN13	-0,949518395	7,043403883	0,000187312	0,000397906
TIMM44	0,306929958	4,969431818	0,00018974	0,000402939
LLGL1	0,353367572	5,526757337	0,00019036	0,00040413
RPL35A	0,508458535	9,470707561	0,000190592	0,000404415
IKZF5	-0,321403266	5,132998022	0,000190613	0,000404415
ATP1A1	-0,479298312	10,33812777	0,000192181	0,000407615
APBB1IP	-0,577785492	5,023801874	0,000192323	0,000407714
STK24	0,352907311	7,679794434	0,000192347	0,000407714
SRA1	0,403974458	5,680121965	0,000195198	0,000413628
FNIP1	-0,302850124	5,358263062	0,000199701	0,000423038
SEPN1	-0,33815063	8,01907311	0,000200088	0,000423728
TNRC6C	-0,506717693	4,790251288	0,000200334	0,000424115
PARP14	-0,370518094	7,764901612	0,000200799	0,000424969
SLC7A1	0,571982068	6,084846001	0,000204076	0,000431771
C18orf21	0,414649429	4,151211635	0,000204783	0,000433014
SLC25A40	0,438420456	3,790935679	0,00020481	0,000433014
CXCL10	1,20775366	5,978356285	0,000204854	0,000433014
SKA2	0,411805092	5,819722917	0,00020541	0,000434053
NDUFS3	0,357376167	6,518206087	0,000206715	0,000436676
KIAA1033	-0,256013012	6,269374848	0,000207106	0,000437367
GMFB	-0,351176736	6,824053673	0,000207916	0,00043894
KCTD2	-0,305368535	5,960203444	0,000208954	0,000440996
C4orf52	0,465631106	5,184507946	0,000211129	0,000445449
SDHAF1	0,525961521	4,151149429	0,000211315	0,000445703
STAU1	0,281318813	7,810128834	0,000212226	0,000447485
DPF2	0,22485286	6,183360329	0,000212405	0,000447724
SAP30	0,499746267	3,274906047	0,000213027	0,000448898
HCLS1	-0,520579717	6,615179412	0,000213326	0,000449388
MTR	-0,360731569	6,112368135	0,00021362	0,000449868
ZDHHC5	0,252349187	7,486136117	0,000214006	0,000450425
DMXL2	-0,438882386	5,521106784	0,000214016	0,000450425
RPL13	0,5039526	10,63846043	0,000214743	0,000451815
CYB5A	-0,768971084	8,368051481	0,000214985	0,000452185
CIR1	-0,276995666	6,139273333	0,000215079	0,000452243
PDE4DIP	-0,359154927	7,308910233	0,000217443	0,000457073
ABHD2	-0,626033664	8,711867908	0,000217997	0,000457959

SYT11	-0,577612547	5,120659118	0,000217999	0,000457959
RTN4	-0,336026595	8,654399719	0,000219641	0,000461266
HSF4	0,835104293	3,46565954	0,000222908	0,000467983
FRMD8	0,271389589	5,915921735	0,000223431	0,000468937
ZNF33B	-0,39993056	5,603534644	0,000223502	0,000468941
ZNF808	-0,336392716	3,955598836	0,000223579	0,000468959
PRPS1	0,310275768	5,971134279	0,000223868	0,00046942
BTBD2	0,293508635	6,802097318	0,000224059	0,000469632
C1orf50	0,392848614	3,521879792	0,000224107	0,000469632
KLHL2	-0,38577473	5,519792901	0,000224283	0,000469856
RPL24	0,565627148	9,822833275	0,000224443	0,000470049
C4orf34	0,51933978	6,493241703	0,000224793	0,000470636
MKL1	-0,303976704	6,113144411	0,000225926	0,000472864
MEPCE	-0,29780265	6,108673989	0,000226552	0,000474029
PACS2	-0,285848018	6,522427238	0,000226919	0,000474622
CCDC23	0,482607077	4,627563656	0,000226975	0,000474622
PCGF3	0,312805665	6,043044645	0,00022705	0,000474633
ATOX1	0,403249741	5,644839116	0,000228364	0,000477115
GOLGA4	-0,416932016	6,971379861	0,000228377	0,000477115
PLBD1	-0,720587722	6,627199237	0,000230622	0,000481595
DAPK3	-0,404318333	5,937456177	0,000230663	0,000481595
SCCPDH	0,547405344	6,693402966	0,000233028	0,000486385
RFC1	-0,317333775	6,353028689	0,000233169	0,00048653
VRK2	0,316369954	5,217104968	0,000234527	0,000489214
RPL7A	0,489810504	10,77035804	0,00023481	0,000489572
SPAST	0,29296554	5,5591659	0,000234842	0,000489572
PSMC1	0,290936067	7,378244525	0,000235052	0,000489858
EIF2S2	0,266366054	7,428569002	0,000237116	0,00049401
SUMO1P3	0,326447656	3,16328624	0,000238805	0,000497377
NIPSNAP3A	-0,455300133	5,307182987	0,000239707	0,000499103
TAF1C	-0,37497286	5,439039455	0,00023987	0,00049929
TRAK1	-0,446292925	6,775891289	0,0002407	0,000500864
ZNF12	0,312460435	5,524546224	0,000241042	0,000501424
DVL3	0,401360748	6,948360581	0,000241317	0,000501843
USP4	-0,236231829	6,456372882	0,000242663	0,000504487
PIP5K1A	0,377768499	6,789399011	0,000243958	0,000507026
SERPINF1	0,742838772	7,519615603	0,000248163	0,000515608
VDR	0,60097645	6,237637154	0,000249229	0,000517664
HSD17B11	-0,511152031	7,544602425	0,000249372	0,000517805
UBE2M	0,328823405	6,260755219	0,00025109	0,000521212
RAB5A	-0,239154311	6,494270582	0,000252034	0,000523012
EDF1	0,338304757	7,977690859	0,00025282	0,000524484
UTP20	0,458860377	5,567618606	0,000252978	0,000524653
FKBP1A	-0,275296446	8,505147943	0,000253212	0,000524979
PHC1	-0,296754737	6,875742442	0,000253957	0,000526362
PSMA4	0,382389029	7,394979475	0,000256428	0,000531323
SCFD1	0,368199369	6,301081609	0,000259264	0,000537036
IMPA2	0,640106659	6,668531386	0,000259697	0,00053777
TRANK1	-0,490503158	5,780032848	0,000260456	0,000539176
PPP1CC	0,305302272	7,639149256	0,000262273	0,000542774

C9orf142	0,411251863	4,876569826	0,000264383	0,000546974
ZFX	-0,305352302	5,370809881	0,000265603	0,000549332
KIAA1429	0,261514406	6,526620805	0,000265737	0,000549442
GNB4	-0,433515501	6,442666368	0,000267174	0,000552245
IFT80	-0,381909325	5,652144724	0,00026748	0,000552711
CDC42BPB	-0,401277654	7,666973934	0,000267663	0,000552921
ACAA1	-0,40013251	6,36207655	0,000268409	0,000554294
ZNF28	0,43987398	4,806542294	0,000269463	0,000556304
HSPA7	0,915600937	3,701673643	0,00026956	0,000556334
B4GALT1	0,44513083	8,762219669	0,000270553	0,000558216
BAG2	0,708631638	4,585902605	0,0002719	0,000560825
SLC30A7	0,282811422	6,081336498	0,000273638	0,000564239
PLEKHG2	0,645835816	6,149017884	0,000274221	0,00056527
RUVBL2	0,341612212	6,681760541	0,000274702	0,000566091
EZR	-0,508078982	9,863077065	0,000275729	0,000568034
C15orf38	-0,354906196	4,774773734	0,000275915	0,000568247
TPRG1L	-0,356191824	6,684107396	0,000278344	0,000573076
RPS26	0,668549636	8,753954375	0,000282448	0,000581351
LAS1L	0,297047736	5,734785298	0,000283507	0,000583353
RPL13AP20	0,519048346	5,202382674	0,000283812	0,000583804
MARCH8	-0,440604251	4,176557705	0,00028464	0,000585331
FBXO8	-0,299590415	5,011622985	0,000287148	0,000590312
LMBRD2	0,53563405	3,872248553	0,000288279	0,000592457
GLTSCR2	-0,377842359	8,314427471	0,000289791	0,000595385
DFFA	0,275041985	5,574888487	0,000291704	0,000599136
NFIB	-0,510409052	6,980491873	0,000291811	0,000599175
MARK4	0,333111531	5,542054704	0,00029213	0,00059965
SGSM2	-0,44212891	5,986326225	0,00029282	0,000600838
NCOA7	-0,623901165	7,890347932	0,000292886	0,000600838
DCK	0,466524625	5,645575965	0,000296173	0,000607399
CD86	-0,589905739	5,212402797	0,000296415	0,000607712
ZBTB44	-0,300970238	6,415142938	0,000298502	0,000611808
FAM111A	-0,330839044	5,874493394	0,000301651	0,000618076
NCSTN	0,312118448	7,928921028	0,000302195	0,000619004
RNF215	0,408305781	4,002100498	0,000304912	0,000624383
S100A6	0,79070189	11,30321992	0,000305713	0,000625836
PRKAG1	0,34124134	6,32587248	0,000307334	0,000628964
SENP7	-0,344522063	4,549683432	0,000310261	0,000634764
CAPZA1	0,317007305	8,058644066	0,000311537	0,000637184
MTDH	0,291282618	7,977400843	0,000312082	0,000638106
FRMD6	0,730615417	5,259984221	0,000312541	0,000638853
CCNJ	0,394874438	3,954804758	0,000313308	0,00064023
LONP1	0,321920692	6,774847381	0,000313952	0,000641354
HLA-DRB1	-0,693498102	10,7973619	0,000314648	0,000642582
POLR1A	0,427605342	5,351617836	0,00031483	0,000642763
GRSF1	0,245427685	7,172644822	0,000316234	0,000645435
TM9SF3	0,323450575	8,470980305	0,000316691	0,00064616
MBD2	-0,282296097	6,534118647	0,000316779	0,00064616
SLC27A1	-0,423111213	5,654209178	0,000316989	0,000646395
PEX10	0,308372054	5,189341788	0,000318459	0,000649199

BCAP29	-0,38764187	6,50186854	0,000318835	0,000649772
MOCS3	0,298787831	4,148913315	0,000319633	0,000651202
CD9	-0,549225677	9,306345322	0,000320078	0,000651915
MRPS27	0,317446365	6,448804882	0,000321639	0,000654898
RPLP2	0,426403699	9,792700931	0,000323321	0,000658128
HUS1	0,269375654	4,589297765	0,000323966	0,000659244
VCAM1	0,809354046	5,891640009	0,00032592	0,000663023
SSBP2	-0,474260377	4,452301953	0,000326805	0,000664623
LTBP2	-0,665523841	8,543722628	0,00032845	0,000667771
PSMG4	0,36490822	3,994933932	0,000330158	0,000671042
LILRB2	-0,635385167	4,550494818	0,000331469	0,000673506
ULK1	0,408709218	6,241538311	0,000333146	0,000676711
SLC25A1	0,373521783	6,596731175	0,000333556	0,000677343
PEPD	-0,3365302	6,641160824	0,000334698	0,00067946
FBXO33	-0,382701964	4,973477634	0,00033524	0,000680359
MSL2	-0,27979961	5,516458455	0,000335417	0,000680515
KIAA0196	0,32915846	6,464257814	0,000337682	0,000684905
PITPNM1	0,454183446	6,373808361	0,000338181	0,000685713
NASP	0,396992545	6,905633621	0,000340072	0,000689344
ARHGAP33	0,734122383	3,154934034	0,000340398	0,0006898
RNASET2	0,549354267	6,675422573	0,000341559	0,000691947
PNKP	0,313339314	5,122813473	0,00034224	0,00069312
GGPS1	0,283908843	6,088874018	0,000345431	0,000699375
CENPV	0,800055313	3,437170999	0,000350636	0,000709545
PCDHB19P	-0,432191474	0,314022608	0,000350663	0,000709545
L3MBTL4	-0,60042787	3,538951512	0,000351293	0,00071061
EIF4ENIF1	-0,23505856	5,116985672	0,000355185	0,000718271
KBTBD2	-0,274938209	6,346809729	0,000355382	0,000718454
NDOR1	0,351644285	5,222284104	0,000357406	0,000722333
VOPP1	0,378127895	7,445915226	0,000358696	0,000724726
BLNK	0,544063734	3,864901532	0,000360743	0,000728645
ZNF251	0,416701797	4,2604324	0,00036166	0,000730281
BTBD10	0,283297586	5,375012338	0,000363481	0,000733741
PCNXL2	0,470699636	4,14904663	0,00036371	0,000733987
SEH1L	0,338456523	5,895072339	0,000364585	0,000735533
MAN2B2	-0,318580617	6,854553292	0,000365706	0,000737578
NEAT1	-0,715829553	9,17186567	0,000368269	0,000742526
DEK	0,296718921	7,609568642	0,00036918	0,000744144
LYRM4	0,467453811	5,128064079	0,000371105	0,000747804
DDX54	0,32280932	6,597413918	0,000374376	0,000754171
IFI16	0,652224381	7,754430763	0,000376313	0,00075785
POFUT1	0,311087182	6,655353283	0,000376786	0,000758578
MVD	-0,383402082	5,558636036	0,000378336	0,000761474
SRGN	-0,676739178	8,335839983	0,000380119	0,000764837
C18orf8	-0,307815671	5,095265546	0,000383719	0,000771853
SYNGR2	0,487659681	8,994814929	0,000385651	0,00077551
XBP1	0,556331688	9,359110445	0,000387185	0,000778365
PRMT7	0,312578879	5,04954904	0,000388217	0,000780028
LRP6	-0,452284542	5,966412823	0,000388241	0,000780028
TAB2	-0,261926015	7,042885	0,000389264	0,000781854

BAZ2B	-0,362897634	5,698494728	0,000389905	0,00078291
ODC1	0,940917743	8,318235382	0,000390216	0,000783303
FAM40A	-0,254932242	5,245589721	0,000393093	0,000788846
PDE4B	-0,599069869	5,26149083	0,000394855	0,000792149
FAM126A	0,670599959	5,920896287	0,000396794	0,000795806
COPS5	0,367186822	6,217470489	0,000397927	0,000797843
ARPC2	-0,281352095	8,723122909	0,00039936	0,00080048
SEC63	-0,285441168	7,342214289	0,000401899	0,000805333
UQCC	0,283792851	5,620340222	0,000405229	0,000811767
SF3A1	-0,274542184	7,611842531	0,000405393	0,000811858
SAFB2	-0,341186609	5,891574457	0,000411041	0,000822927
CD82	-0,51608314	6,740925961	0,000411363	0,00082333
MRPL33	0,517357813	5,903176946	0,000411701	0,000823764
FDX1	-0,344084622	5,533246783	0,000414251	0,000828623
MRPS9	0,249206638	5,026672535	0,000414737	0,000829353
COX7B	0,586474869	6,948115947	0,000416691	0,000832849
FMNL1	-0,473406258	6,271529898	0,00041673	0,000832849
DEAF1	0,368856099	5,241261695	0,00042215	0,000843434
SMU1	-0,223318184	6,75976507	0,000424462	0,000847804
COX8A	0,393550393	7,474112691	0,000427499	0,000853622
ESRRA	0,363469758	6,021147826	0,000429296	0,000856959
CIITA	-0,575633035	5,510870758	0,000434208	0,00086651
RPUSD3	0,30167748	4,949415571	0,000434446	0,000866731
GPX4	0,43798262	8,449670407	0,000435945	0,000869468
RIC8A	0,240748325	7,10844306	0,000438404	0,000874116
NCKAP1	0,271084053	8,11297604	0,000439034	0,000875115
TRIM4	-0,314787607	5,755009721	0,000439555	0,000875899
HAUS1	0,528833363	4,614600537	0,000440093	0,000876714
TYMP	0,537868293	7,456648914	0,000445332	0,000886892
PDHX	0,302959384	5,355129065	0,000447107	0,000890167
SRI	0,501108549	7,557329574	0,000447692	0,000891073
KIAA1432	-0,341233158	5,218727092	0,000451107	0,000897607
DIP2A	-0,305257319	5,539830614	0,000451289	0,000897708
NAA35	0,265226485	4,730007199	0,000455095	0,000905013
GSK3A	0,280879926	6,657205539	0,00045794	0,000910406
PMEPA1	0,608766369	7,299668907	0,000466155	0,000926425
RPS4X	0,45577377	10,95591451	0,000466269	0,000926425
NSL1	0,360279005	5,781673494	0,000466522	0,000926657
SMN1	0,364482527	1,937041609	0,000467226	0,000927786
NID2	0,711750577	4,779962597	0,000468202	0,000929453
GPSM1	-0,481596774	4,591792441	0,000470967	0,00093467
HIF1AN	-0,296711814	6,216010464	0,000472172	0,000936788
RHOQ	-0,321562042	7,025040985	0,000477514	0,000947111
NUPL1	-0,3355118	6,560283971	0,000479248	0,000950274
DIS3L	-0,263172717	5,302691633	0,000481602	0,00095447
MRPS31	-0,275605813	4,33081332	0,000481644	0,00095447
DMTF1	-0,331480623	5,502970273	0,000485012	0,000960866
AARS2	0,323765372	4,981092198	0,000487687	0,000965883
ATP2B1	-0,475413393	6,209237849	0,000488355	0,000966694
MITD1	0,371501337	4,180825237	0,00048838	0,000966694

FPGS	0,315838906	6,426427485	0,000489897	0,000969415
NCKIPSD	0,295117403	5,053661134	0,000492585	0,000974452
RAP2C	0,313285692	5,952340089	0,000493603	0,000976183
C9orf114	0,290086447	4,973339058	0,000494672	0,000978014
LSP1	-0,502951849	6,827802576	0,000497189	0,000982704
ABHD3	0,390162258	5,208986974	0,000497359	0,000982736
C17orf59	-0,306935966	3,842631941	0,000497493	0,000982736
PAPOLA	0,246108478	7,93825107	0,000497714	0,000982887
MAPK12	0,566104771	4,014885122	0,000499645	0,000986416
THBS1	-0,73222659	9,263276621	0,000501392	0,000989578
AIF1	-0,581853392	5,901555026	0,00050322	0,000992898
DBI	-0,431149306	7,579514316	0,000503841	0,000993442
MAP1B	0,879576077	6,370410736	0,000503856	0,000993442
CTDSP2	-0,361029756	8,666277572	0,000503933	0,000993442
PTCD3	0,270215984	6,179556909	0,00050599	0,00099689
NEDD8	0,364520014	7,319182715	0,000506016	0,00099689
PDE8A	-0,389223682	5,327836537	0,00050612	0,00099689
MRPL48	0,498419462	5,017327549	0,000506398	0,000997149
SET	0,297719135	8,835155537	0,000507468	0,000998967
TMEM56	-0,538865124	4,874551436	0,000508037	0,000999799
TRIM21	-0,322510911	5,507631972	0,000513165	0,0010096
ASPN	0,825402424	5,44627038	0,000514256	0,001011454
AHI1	-0,361995549	4,453558512	0,000519422	0,00102132
PWP1	0,251247423	5,854694771	0,000520401	0,00102295
PIGF	0,403421423	4,781044211	0,000525326	0,001032333
MYBBP1A	0,384561076	5,977604108	0,000525897	0,001033157
CNOT2	0,361588535	6,519825973	0,000529195	0,001039337
MRPL16	0,332833191	5,510737407	0,000531902	0,001044354
DNAJC4	0,329570235	5,442913963	0,000532954	0,001046119
TUBA1B	0,41326729	10,31431866	0,000534379	0,001048613
RPS6KA4	0,354878131	6,023868298	0,000535182	0,001049886
POMP	0,441192577	6,983438919	0,000536292	0,001051761
COMMD1	0,397785945	5,119034349	0,000538527	0,001055841
TRMT12	0,302516811	4,230232987	0,000538757	0,001055989
ANAPC16	-0,350490109	7,514434731	0,000541525	0,001060823
COX11	0,356161296	6,033915258	0,000541535	0,001060823
ZFHX3	-0,482630481	6,120171931	0,000542687	0,001062536
ZDHHC24	0,343077932	4,595761161	0,000542875	0,001062536
ZFYVE21	-0,288250186	5,896557101	0,000542876	0,001062536
DDB1	0,24081037	8,433439459	0,0005484	0,001072991
BRAF	0,376626701	3,670088339	0,000548533	0,001072991
OGFOD1	0,217796014	5,843079931	0,000550445	0,001076422
CCDC9	-0,334942086	4,911516093	0,000551704	0,001078576
WWP1	-0,289651936	6,380687071	0,000553227	0,001081243
SDHB	0,355668506	6,690273753	0,000556507	0,001087341
HLA-DRA	-0,66351543	11,61348453	0,000557161	0,001088307
RBPMS	-0,53654434	6,827326979	0,000560352	0,001094226
NKIRAS1	-0,313902188	3,803180926	0,000565069	0,001103122
ZGPAT	0,350844902	5,414339095	0,000565966	0,001104558
PTPRS	-0,460154962	6,127874047	0,000566372	0,001105034

PLEKHA4	-0,516701669	5,014968936	0,000569369	0,001110562
DHX8	0,296853611	6,306088184	0,000569717	0,001110924
TMED1	0,343886675	5,220672075	0,000570361	0,001111861
C11orf73	0,336936342	5,051980982	0,000574571	0,001119748
MED13L	-0,458359349	7,051483593	0,000576165	0,001122534
GRAMD1A	0,475967749	7,341631473	0,000576866	0,001123578
FAM172A	-0,316574936	5,125199554	0,000580298	0,00112994
RAB31	-0,450163979	7,934169931	0,000580568	0,001130143
SIKE1	0,282726641	5,592846523	0,000581515	0,001131664
TAF4B	0,541885313	3,392893742	0,000583315	0,001134843
TCFL5	0,376721098	4,261861081	0,000584339	0,001136511
VPS8	-0,25598471	5,454744819	0,000586433	0,001140259
MED19	0,332739991	4,224336584	0,000588543	0,001143932
NDUFB5	0,464528256	6,754383126	0,000588658	0,001143932
NUP54	0,312556989	5,679520046	0,000592347	0,001150773
SNORA8	0,430547221	4,431380531	0,000594266	0,001154173
LOC100190986	-0,551483903	3,303495795	0,000600549	0,001166044
SMEK2	0,240158143	7,113610934	0,000600981	0,00116655
ZHX1	-0,276591258	5,702694573	0,000602025	0,001168027
TERF2IP	-0,271518038	6,544538907	0,000602085	0,001168027
TNKS2	-0,249912502	6,783660593	0,000606103	0,001175488
EP300	-0,360288907	7,170869341	0,000611892	0,001186379
ARHGEF9	-0,361122155	4,846739814	0,000615217	0,001192485
RAB4B	-0,318727551	5,340532281	0,000616096	0,00119385
CRCP	0,249583715	6,185721914	0,000616768	0,001194813
HSPA8	0,380768771	10,72142077	0,00061902	0,001198834
RPS6	0,575188761	11,30714805	0,0006219	0,00120407
COMMD4	0,323417216	6,064767247	0,000623348	0,001206531
TBCA	0,355113152	6,850381673	0,00062881	0,001216757
KDELC2	0,409251389	6,281444495	0,000632822	0,001224049
MAGEF1	0,376583212	5,965114335	0,000632937	0,001224049
ZNF394	-0,237460758	4,920780856	0,000636773	0,001231119
ZNF23	-0,28462122	3,819941294	0,000644919	0,001246515
DERA	0,483243703	6,212837405	0,000645869	0,001247817
POP5	0,400372383	4,679292879	0,000645959	0,001247817
C2orf68	0,327100266	6,172261363	0,00064783	0,001251077
NBPF9	-0,423031885	5,122983228	0,000648509	0,001252034
RARRES1	1,13539932	6,146470969	0,00064884	0,001252318
PPP1R12A	-0,251195805	6,452807685	0,00065081	0,001255765
TMCO3	0,414865474	6,593965247	0,000652562	0,00125879
HSPA13	0,42824796	6,209403849	0,000658	0,001268921
ASXL1	-0,301845552	6,611698096	0,000661901	0,001276083
CD200	-0,483390712	3,849738516	0,000666871	0,001285301
SEC23IP	0,25248645	6,306465174	0,000667351	0,001285659
IRS1	0,660750165	5,813061038	0,000667434	0,001285659
SBF2	-0,378842952	5,594636292	0,000668876	0,001288073
SUPT5H	0,34662852	7,821694407	0,000669584	0,001289072
USP11	-0,336115786	6,806556287	0,000672351	0,001294034
STAT3	-0,308864571	8,970236901	0,0006726	0,001294147
NDUFA1	0,425038836	6,746052471	0,000674272	0,001296999

AEBP1	0,701579336	8,968680462	0,000677466	0,001302775
FDX1L	0,362588575	4,134264936	0,000678266	0,001303946
PMF1	0,382248741	6,229917093	0,000679725	0,001306383
SLC16A1	0,766368744	5,222282971	0,000684248	0,00131448
SCMH1	-0,28904719	5,637650445	0,000684324	0,00131448
SQSTM1	0,481552543	9,673232954	0,000688876	0,001322851
C6orf48	0,502272981	7,144568706	0,000689631	0,001323928
TMEM14C	0,466587151	7,43617007	0,000691528	0,001327196
RFFL	0,287292889	5,846761864	0,000695178	0,001333827
DENND1A	0,382479234	5,195968049	0,000698276	0,001339394
SAP130	0,277663269	5,482944549	0,000704229	0,001350431
SKAP1	0,72814216	4,050231913	0,000706933	0,001355071
EIF2B5	0,249848863	6,126413938	0,000707046	0,001355071
ZC3H4	-0,324616789	5,902657121	0,000707977	0,001356383
RAB2B	-0,255298764	5,378092682	0,000708128	0,001356383
PPP1R15B	-0,265582247	7,230002327	0,000709635	0,001358887
MMD	0,604161452	5,411725753	0,000710049	0,001359299
MANBA	-0,345331638	6,12046957	0,000711245	0,001361206
LARP1	0,395316727	8,0876532	0,000717525	0,00137284
SLC39A3	0,260493049	4,998938982	0,000718254	0,00137385
AHCTF1	0,308167995	6,493477907	0,00072519	0,001386728
TPM3	0,294929808	9,537062132	0,000727258	0,001390292
ENO2	0,61690699	6,437479735	0,000731715	0,00139842
MTPAP	0,281126662	5,164471004	0,000734417	0,001403191
HABP4	-0,368133359	4,371382109	0,000741639	0,001416592
OSBPL9	-0,258773531	7,134108459	0,000743631	0,001419999
CLCN7	0,346022019	6,98136126	0,000746215	0,001424536
RPL13A	0,479075285	11,37282542	0,000747739	0,001427044
PPIL4	-0,215755453	5,410246291	0,000752224	0,001435204
PQLC2	0,30013178	4,6744612	0,000754529	0,001439198
NPDC1	-0,580720127	5,831169553	0,000755272	0,001440212
FAM96B	0,437087196	6,522085055	0,000758684	0,001445953
FANCE	0,426720146	3,661315587	0,000758707	0,001445953
MTA1	0,358179966	5,912117716	0,000759548	0,001447153
PITPNC1	0,596399808	5,191779191	0,000760476	0,001448515
NME7	0,314435686	5,219381728	0,000763423	0,001453723
COX10	0,264470933	4,244698044	0,000767192	0,001460131
CPSF3L	0,24084044	6,652520639	0,000767217	0,001460131
SEC61B	0,360874427	6,787782951	0,000767923	0,001461068
GTF2A2	0,449823427	5,887831226	0,000768759	0,001462251
CLDN1	0,975217898	7,07264604	0,000769338	0,001462943
UHMK1	0,503994556	5,145800859	0,000777132	0,001477352
PPWD1	-0,249077415	4,702283839	0,000779186	0,001480845
FKBP8	-0,268327256	8,305519517	0,000781785	0,001485369
FAM126B	-0,28249306	5,393014704	0,000790182	0,001500905
EPS8	0,555119796	7,459460004	0,000793315	0,001506437
AAAS	0,281575996	5,419923218	0,000798284	0,001515451
TMEM30A	-0,320455031	8,647313303	0,000804398	0,001526388
PXMP2	0,437689482	3,791982302	0,000804493	0,001526388
RANBP2	-0,33021613	7,322390484	0,000804863	0,001526636

OTUD7B	-0,372050533	5,783170262	0,000805071	0,001526636
CNIH4	0,441833443	6,758967197	0,000809026	0,001533408
AMD1	-0,323262288	7,14073822	0,000809092	0,001533408
ASCC2	0,34470065	6,637671743	0,000818641	0,001551073
SPA17	-0,535092795	3,866212346	0,000820373	0,001553924
FXC1	-0,315120661	5,921919169	0,000823258	0,001558955
RPS15A	0,570363901	9,986986366	0,000828641	0,001568712
ZNF532	0,400628105	6,489433813	0,000841004	0,001591676
KLRK1	-0,675288912	3,102043619	0,000842575	0,001594205
C10orf12	0,435825368	2,828830956	0,000843556	0,00159562
HSPA1B	0,619544901	8,359023823	0,00084816	0,001603883
SMARCD2	0,277047653	7,132651148	0,000862848	0,001631206
EML3	-0,225409199	5,848440333	0,000869056	0,001642487
QSER1	0,301791493	5,651610101	0,000873692	0,001650791
SLC35A4	-0,247281845	6,98499824	0,000875283	0,00165334
GSTP1	0,485070611	9,912778329	0,000876492	0,001655165
CRIP1	-0,663615429	8,058787825	0,000881157	0,001663513
LY6E	0,639663214	9,238006965	0,000881787	0,001664242
ULK3	0,319643209	5,471537452	0,000882695	0,001665494
RPS3A	0,642138399	11,47594262	0,000888953	0,001676838
ANKZF1	0,318909913	5,44403831	0,000906024	0,001708566
ZBTB1	-0,2243171	5,759665625	0,000907381	0,001710654
FNDC3B	0,341144191	7,547391563	0,000908972	0,001713178
PAIP2	-0,254399171	6,90599604	0,000910676	0,001715916
HSPA4L	0,627268032	3,028990304	0,000914586	0,001722808
USMG5	0,452725569	6,091764127	0,000920116	0,001732745
PTP4A3	0,5554184	6,378254411	0,000921832	0,001735498
DCP2	-0,300712279	5,500895758	0,000928548	0,001747658
PFKL	0,269379759	7,754834131	0,000934704	0,00175876
OSGEPL1	0,322585802	3,513934458	0,000935409	0,001759601
TRO	0,748393547	3,169951686	0,000936805	0,001761615
HSPB11	0,351268762	4,788683973	0,000937201	0,001761615
TMEM43	-0,334169839	7,696264408	0,000937255	0,001761615
HMG20B	0,291112092	6,213525251	0,000944719	0,001775155
BBX	-0,300888212	6,406674075	0,000949226	0,001783132
RAP2A	-0,339704623	6,223473755	0,000955062	0,001793602
CCNC	0,427997464	6,628340752	0,000959622	0,001801669
C11orf74	-0,314610486	3,980287582	0,000962752	0,001807047
SERBP1	0,253616456	8,633039977	0,00096778	0,001815986
TBC1D3	0,431136508	4,775075771	0,000970013	0,001819364
MAT2A	-0,299627638	8,023121387	0,000970114	0,001819364
STXBP3	-0,240796592	5,749748514	0,000980572	0,001838471
UXT	0,385401275	6,22977839	0,000982741	0,001842032
CLPP	0,297113015	5,715192235	0,000987462	0,001850372
FAM185A	0,296499344	2,952483641	0,00098826	0,001851359
DUSP7	-0,476891166	5,593225412	0,000990096	0,001854289
NDUFA2	0,517500209	6,373394236	0,000996231	0,001865266
DBT	-0,248763154	5,623142761	0,000999916	0,001871651
PFKFB3	-0,541099316	7,736113983	0,001002698	0,001876344
ADAM17	-0,34197872	6,095162875	0,001003327	0,001877006

CWC15	0,290479706	6,086423084	0,001004241	0,0018782
TMEM60	0,339044244	4,56173655	0,001009825	0,001888126
CCDC142	0,266864949	5,525120209	0,001017048	0,001901111
PTAR1	-0,318896842	5,843361631	0,001021858	0,001909578
SLC4A1AP	0,237252342	5,688775054	0,001023791	0,001912667
NEO1	-0,546691022	6,573958778	0,001031225	0,001925672
PAWR	0,365962223	4,506622542	0,001031318	0,001925672
DCAF17	0,231783626	4,572661427	0,001032782	0,001927879
INO80E	0,279738853	5,478730392	0,001036701	0,001934665
INPP5F	0,441511921	4,928361156	0,001037649	0,001935904
MYH9	-0,428001321	10,86329206	0,00104054	0,001940766
ZDHHC6	0,277818496	5,724808002	0,001040916	0,001940938
RFXANK	0,299379553	5,741422974	0,001052302	0,001961631
DERL1	0,231763036	7,350172879	0,00106169	0,00197859
TWISTNB	0,337432489	5,340978425	0,001064316	0,001982943
ELAVL1	0,205882542	6,693999605	0,001065624	0,001984838
ZNF330	-0,288571802	5,499479714	0,00106764	0,001987754
BCL7B	-0,283921858	6,408707931	0,001067773	0,001987754
TCERG1	0,282483612	5,701371886	0,001070838	0,001992917
GCA	-0,359675634	5,717830501	0,001074858	0,001999852
TMEFF1	0,54361254	2,942823018	0,001082134	0,00201284
XRN1	-0,277729213	5,926227172	0,001084298	0,002016315
ZNF503	-0,490303756	5,204163744	0,001085157	0,002017362
MRPL39	0,381517551	4,727300865	0,001086907	0,002020064
TXNL1	0,397786541	6,520483181	0,001091059	0,002027228
SHOC2	-0,205809475	6,260949235	0,001103023	0,002048901
SETD6	0,316925681	4,256061188	0,001116118	0,002072661
MGAT4A	-0,389688179	6,47344644	0,001119092	0,002077101
TSR2	-0,239564189	5,923459225	0,001119118	0,002077101
TBC1D12	-0,23643333	4,099749619	0,001128319	0,002093608
PTPN18	-0,263925206	6,60885273	0,001128814	0,002093957
JHDM1D	-0,336183618	5,660013799	0,001132144	0,002099562
CLSTN1	0,372555921	8,263650001	0,001146309	0,002125254
CSF1	-0,576789723	5,951903091	0,001147645	0,002127151
PRPF8	-0,359023448	8,779182695	0,001148576	0,002128299
BMS1	0,280940376	6,355947778	0,001152687	0,002135337
CLIC6	0,994805529	7,473059925	0,001157739	0,002144113
PRKRIP1	0,276098012	5,075895428	0,00116242	0,002152198
COX7A2L	0,40651465	7,295924133	0,001165529	0,002157368
IER3IP1	0,480434808	6,697209388	0,001179559	0,002182745
THG1L	0,380119685	3,714114149	0,001181568	0,002185869
PTK2	0,270562435	7,468818034	0,001193045	0,002206503
ACACA	-0,389990391	6,961997005	0,001195574	0,00221058
MYEOV2	0,476783219	5,638051279	0,001201735	0,002221369
ACAA2	-0,460874701	6,685689172	0,001212451	0,002240569
PPM1B	-0,206750984	6,148678891	0,001212941	0,002240869
ENSA	0,288087291	8,361693377	0,001215962	0,002245841
HADHA	-0,178358018	8,149407666	0,001219904	0,002252512
C1orf54	-0,508803385	3,582451379	0,001220865	0,002253676
VMA21	0,278677546	6,321916895	0,001222697	0,002256447

WDR6	-0,294119256	7,275565366	0,001224744	0,002259613
RB1	-0,318266768	6,253347467	0,0012254	0,002260211
PYGL	0,59623223	6,524889087	0,001225823	0,00226038
CD7	0,699470371	4,088486259	0,00122772	0,002263267
DEF6	0,355479045	4,930169508	0,001228819	0,00226468
UGP2	0,276672017	7,582126826	0,001230683	0,002267502
REL	-0,471607683	3,382007236	0,001236516	0,002277635
KIAA0556	-0,347588155	5,22503336	0,001237086	0,002278069
ZNF22	-0,300830334	5,431382988	0,001238171	0,002279451
PMM1	-0,480246266	6,070193305	0,00123992	0,002282055
RABGGTA	-0,249019968	5,384820581	0,001241849	0,002284989
CLIP1	-0,322447122	7,01106106	0,001244865	0,002289921
PMPCB	0,305596449	6,43473717	0,001260856	0,00231871
NFX1	-0,255455479	6,195900023	0,001261371	0,002319033
CHMP4A	0,305010739	5,948818295	0,001262686	0,002320824
DCAF8	-0,252899453	7,087307608	0,001265094	0,002324622
LOC152217	0,380801047	4,263277093	0,001267839	0,00232904
CCDC117	0,262585575	5,780293905	0,001269996	0,002332374
PLEKHG3	0,529061453	5,289804283	0,001294375	0,002376506
TEAD4	-0,487016072	4,271724516	0,001297373	0,00238137
KLHL24	-0,343908286	6,616725211	0,001301081	0,002387532
NCF4	-0,492047269	4,908102057	0,001308394	0,002400307
ACOT13	0,399621801	5,915416259	0,001310748	0,002403978
FANCM	0,431205946	3,002324933	0,00131678	0,002413991
HERC2	-0,408342822	6,33142793	0,001316915	0,002413991
NUPR1	-0,593378327	7,137377525	0,001318386	0,002416038
UQCRFS1	0,41218413	6,819179932	0,001323032	0,002423899
PSMD9	0,315916052	6,003565325	0,001332952	0,002441418
SPSB2	0,52376767	4,822401517	0,001340807	0,002455146
C7orf29	0,610982165	4,308749736	0,001346791	0,002465441
OAF	0,538760917	4,80601185	0,001358259	0,002485768
PLOD3	0,37646129	6,837329366	0,001359553	0,002487467
NRBP2	-0,427203578	5,427392541	0,001366138	0,002498845
RNPEPL1	-0,316023085	7,274809211	0,001367751	0,002501124
FAM53C	-0,264272744	5,821326104	0,001375126	0,002513936
NAP1L4	0,222977658	7,29089225	0,001378857	0,002520082
STK3	0,254392396	5,518344876	0,001382462	0,002525994
FNTA	0,372720639	6,826686414	0,001397872	0,002553466
OGFR	-0,308466667	6,1776387	0,001399316	0,002555321
GLRX5	0,265206308	5,68114638	0,001399637	0,002555321
SAE1	0,270591274	6,910757959	0,001401137	0,002556747
AP2A1	0,25071847	7,008235559	0,001401168	0,002556747
BTN3A1	-0,365649159	6,420800658	0,001418601	0,002587864
DHX57	0,258020005	5,227469467	0,001420644	0,0025909
MINA	0,249933596	5,766539342	0,001422524	0,002593634
NDUFA11	0,463626973	6,605390684	0,001432683	0,002611459
USP3	0,323175777	5,489495398	0,001434378	0,002613849
MYL12A	-0,32656749	9,206672303	0,001435373	0,002614963
MLEC	0,385482714	8,48060106	0,001437181	0,002617558
ARFIP2	0,285191328	6,267776335	0,001452317	0,00264442

STAG1	-0,309919813	5,363645783	0,001460961	0,002659449
DBR1	-0,197160008	4,781997441	0,00146795	0,002671458
SMN2	0,355244214	6,143578435	0,00148041	0,002693413
PATZ1	0,337582696	6,019334792	0,001491365	0,002712621
DNAJB12	-0,22758763	6,363078022	0,001495156	0,002718791
AMIGO2	0,700654291	6,64007493	0,001497058	0,002721525
FIP1L1	0,189055927	5,57331828	0,001519014	0,002760702
SBNO1	0,416921324	5,009470461	0,001533321	0,002785963
CCDC90B	0,394701766	5,45584564	0,001534687	0,002787701
STAM2	-0,251783694	6,080223154	0,001541423	0,002799191
PPA2	0,353574196	6,493225735	0,001545194	0,002805293
UXS1	-0,309270227	6,833996907	0,001560569	0,002832451
ITGB3BP	0,364277042	4,20485021	0,001568846	0,002846717
TRPC4AP	0,177763296	7,393791388	0,001572315	0,002852253
SF3A3	0,189103785	6,796378759	0,001586148	0,00287658
PGLS	0,37288812	6,119036552	0,001591197	0,002884486
ACTN4	-0,338620211	9,560370523	0,001591353	0,002884486
SIN3B	-0,274983043	5,85279905	0,001593954	0,002888432
MLL3	-0,378508933	6,850361863	0,001595928	0,002891241
H3F3B	-0,259260437	9,796828749	0,001608696	0,002913598
TNFRSF12A	0,678712761	7,002308148	0,001628296	0,002948314
PPA1	0,370141617	8,172300645	0,001640645	0,002969885
APEH	0,250909156	7,020172467	0,00164263	0,002972689
PDCD10	0,419036384	5,960646519	0,001643154	0,002972849
RCAN3	0,314962024	3,650037616	0,001645908	0,002977042
TTC8	0,265684983	4,559346636	0,001659847	0,003001458
HYI	-0,417536899	4,926596163	0,001666194	0,003012136
DPYD	-0,510280856	6,641690881	0,001668839	0,003016117
SLC25A37	0,528176222	5,763379792	0,001678678	0,003033096
CCDC90A	0,273781179	4,821080167	0,001679482	0,003033745
EXOC8	-0,264296178	5,164233259	0,001682144	0,003037748
ACAP3	0,352152482	5,302190539	0,001682945	0,003038391
HSD17B12	-0,289731312	7,314124795	0,001697408	0,00306369
NMI	0,369871799	5,520993435	0,001700437	0,003068346
C7orf60	0,460459674	4,699905783	0,001716072	0,003095618
TOP2B	-0,288800379	7,417382651	0,001716459	0,003095618
DPH5	0,342204892	4,710638773	0,001720158	0,00310147
SNRPD3	0,255734233	6,62598823	0,001738765	0,00313419
ZZZ3	0,2961339	5,493827338	0,001755307	0,003163172
EDEM3	0,349753358	6,499539283	0,001760021	0,003170827
DNAJA4	-0,525180601	5,628508848	0,001762781	0,003174961
ZNF337	-0,342882671	4,494441935	0,001764573	0,00317735
ABCF1	0,305235787	7,017632011	0,001776167	0,003197381
ZMIZ2	0,379431772	7,420974758	0,001780186	0,003203771
KIF3B	-0,295408636	6,651203505	0,001781358	0,003205034
IKBIP	0,350210991	5,123771658	0,001800979	0,003238855
RAVER1	0,307760388	6,447096992	0,001801105	0,003238855
HMHA1	-0,365126266	6,438167282	0,001803457	0,003242228
LOC642852	0,423714633	3,867328453	0,001804032	0,003242407
PIK3AP1	-0,498583554	5,885667441	0,001814109	0,00325966

WDR18	0,368356858	5,194327533	0,001831657	0,003290324
RNF25	0,267813764	4,812134172	0,001840306	0,00330499
RNPEP	0,290582249	7,243216516	0,001857486	0,003334965
NNT	0,424259913	6,718729285	0,001863953	0,003345695
INTS1	0,404560807	7,458862299	0,001877745	0,003369563
PARP11	-0,343799603	4,025727557	0,001887928	0,003386947
SORL1	0,671577806	7,390470617	0,001891489	0,003392441
PDE4D	-0,61813111	6,353063287	0,001899903	0,003406637
IWS1	0,227564462	6,296522822	0,001901181	0,003408032
BET1L	0,26808449	6,951023142	0,001906561	0,003416777
FBXO11	-0,224047932	6,432284358	0,00191567	0,003432201
FLOT1	0,333053896	8,367420529	0,001918107	0,003435664
UBE2H	0,287789378	6,66677829	0,001920338	0,003438756
AGAP1	0,400335263	5,042689789	0,001922551	0,003441815
DCTD	0,234930275	6,779339183	0,001926753	0,003448433
TRIM24	0,37304135	5,851805655	0,001943128	0,003476827
SAMD1	0,28796994	4,348453128	0,001945494	0,003480147
ZADH2	-0,342328667	5,502896679	0,001948183	0,003484045
NCL	0,296073772	9,511720928	0,001953558	0,00349274
NR1D2	-0,348169061	6,162129628	0,001971903	0,003524616
LGALS1	0,702947391	8,883101754	0,00198463	0,003546435
DDT	0,385987056	5,980509478	0,002030401	0,003627275
PHF8	0,408624198	5,901336871	0,002031921	0,003629038
NDUFA10	0,202423746	6,623674376	0,002039115	0,003640933
DCTN2	0,26171664	7,420351241	0,002053091	0,003664929
DCUN1D1	0,302909623	5,511051058	0,002056724	0,003670454
TRAPPC2L	0,42186086	5,590509926	0,002059448	0,003674353
FAR1	-0,25932462	6,306142271	0,002064197	0,003681862
PELO	0,317881878	5,562183472	0,002081896	0,003712461
HNRNPA1	0,230628817	10,18360482	0,002100874	0,003745322
RFK	0,443348486	5,901002878	0,002107387	0,003755952
RNASEK	-0,266376874	5,630386807	0,002128477	0,003792549
RPS15	0,386433914	8,89884735	0,002133791	0,003801024
WTAP	-0,250124907	6,99251217	0,002146766	0,003823138
EIF1	-0,26040732	9,75267456	0,002153618	0,00383434
DNMBP	0,388482713	5,762803438	0,002170864	0,003864035
TMTC3	0,299279798	5,549241155	0,002181584	0,003882103
MAF1	0,249934582	7,464104361	0,002194212	0,003903556
RPL32	0,431215142	10,13147307	0,002205244	0,00392216
RASA4	-0,364000325	6,113630617	0,002211878	0,003932933
RAB22A	0,234056834	6,501854061	0,002213576	0,003934925
FBXO46	0,31450801	4,658803337	0,002217386	0,003940672
EAF1	-0,219500533	5,947522643	0,002221572	0,003947082
FJX1	0,700975287	2,736901396	0,002226445	0,00395471
SHPK	0,329328226	4,84257507	0,00224368	0,003984285
ZNF274	-0,272002843	5,059838656	0,002256854	0,004006635
LPCAT4	0,587635334	5,573758805	0,002262846	0,004016227
TRIM44	-0,339043721	6,417496192	0,002275858	0,004037276
CD74	-0,53667791	12,69606995	0,002275889	0,004037276
LOC285359	0,40773192	1,805874164	0,002287303	0,004056469

CDC40	-0,216094713	5,350988994	0,002306292	0,00408908
NSMCE1	0,302751145	5,967353039	0,002325204	0,004121541
BCLAF1	-0,230123435	7,508054142	0,002327607	0,004124728
ATG12	0,271649287	5,334177007	0,002329452	0,004126925
CCDC159	-0,292906404	3,99868676	0,002331017	0,004128625
HELB	0,506604001	1,517432225	0,002341316	0,00414579
ATP8B1	0,478877562	6,562601469	0,002357489	0,004173343
ANXA5	-0,305242546	9,255467794	0,002367113	0,004189292
LAMA5	-0,499183771	7,633378045	0,00237652	0,004204849
HINT1	0,434179844	8,015313785	0,002398214	0,004242133
RAI14	0,431438583	7,513640779	0,00240892	0,004259965
C20orf111	0,378648806	5,786326221	0,002421191	0,004280556
DYNLT3	-0,272459452	5,730430926	0,002433217	0,004300702
FTL	-0,484779133	12,88988025	0,002436683	0,004305712
ALMS1	-0,403593481	5,103627735	0,002446772	0,004320942
MRPL40	0,345393743	5,37162882	0,002447032	0,004320942
DHX38	-0,258794907	6,330022389	0,002447203	0,004320942
BRI3	-0,372272458	7,620185504	0,002449337	0,00432359
PCM1	-0,396239021	7,417332379	0,002451018	0,004325438
C10orf11	-0,419330759	3,527468567	0,002453879	0,004329367
UBL5	0,402100398	7,28526111	0,00248155	0,004377054
ZNF445	-0,317542115	5,189277909	0,002495671	0,004400823
GOLGA8B	-0,590355819	5,863902761	0,002505516	0,004416895
NGFRAP1	-0,399547144	6,758413902	0,002506081	0,004416895
TMC6	0,358306704	7,030858294	0,002513073	0,004428074
EIF4G3	0,311241792	6,523397808	0,002535356	0,004466182
TMED10	0,238742679	9,086678469	0,002539219	0,004471832
RPS27	0,382448035	10,29099476	0,002549131	0,004488129
MUL1	-0,220122193	5,887680354	0,002551988	0,004491999
ZNF805	-0,254503598	4,503296063	0,002557998	0,004501416
CES2	-0,345114755	6,268892379	0,002559975	0,004503222
PSMB1	0,338059092	7,641044876	0,002560345	0,004503222
ATP6V1C1	0,263617772	6,757529352	0,002567631	0,004514392
H2AFY	0,22995081	8,058830336	0,00256802	0,004514392
MAMDC4	0,713119443	2,567882463	0,002570205	0,004517068
TBC1D25	-0,188918126	4,932001087	0,002580712	0,004534366
INTS2	0,25398928	4,633466004	0,002581558	0,004534684
DCTN6	-0,282440074	5,259770226	0,002594436	0,004556131
STRN3	0,315626533	5,785277338	0,002596391	0,004558391
PLIN2	-0,519057161	7,162445936	0,002602725	0,004568335
INO80C	-0,337563272	4,305427939	0,002625256	0,004606697
ATP13A3	0,369629624	7,313112144	0,002635334	0,004623191
PGD	0,572844279	8,417955923	0,002653619	0,004654071
BTBD7	-0,277741264	5,866426972	0,002657323	0,00465832
IQGAP2	-0,558546162	5,303165319	0,002657408	0,00465832
DNAJC11	0,224106539	5,804287842	0,0026599	0,00466149
HNRNPH3	-0,223305682	7,134427206	0,002661399	0,00466292
NPC1	-0,384039461	6,821029487	0,00269999	0,004729318
FLJ45340	-0,392695148	6,168132542	0,002703091	0,004733533
TNKS1BP1	-0,36784439	8,012628231	0,002711081	0,004746306

C5orf15	0,305863245	7,17683149	0,002754746	0,004821513
HSPA1A	0,554259077	8,65244657	0,002768918	0,004845073
ATP5I	0,414714724	6,612739012	0,002771353	0,00484809
ZNF143	0,197621258	4,583905506	0,002772678	0,004849164
CLIP3	0,605471019	4,57806483	0,002815913	0,004923515
FXR1	0,288724478	6,989910645	0,002819907	0,004929235
HECTD2	-0,283019323	3,731846685	0,002820672	0,004929309
SIVA1	0,382200619	6,38299889	0,002826358	0,004937979
RPL7	0,356784401	10,53746491	0,002854713	0,004986241
COMMD10	0,404210754	5,251447277	0,002859787	0,004993825
DEDD	0,225620173	5,895536301	0,002885981	0,005038276
KHNYN	-0,23163047	6,771702954	0,002896752	0,005055785
TYW1	0,248213901	4,956140308	0,002909557	0,005076834
LZTS2	0,268854949	6,499431706	0,002922445	0,005096869
IGF2BP2	0,777050624	5,173420187	0,002922534	0,005096869
MDN1	-0,419030602	5,66747918	0,002939498	0,005125144
LANCL1	-0,301657567	6,261840139	0,002944652	0,005132818
RAB6A	-0,211417167	7,868533537	0,002947758	0,005136918
TSPO	-0,399352314	7,444990749	0,002949668	0,005138933
PDGFC	0,435266105	5,741808246	0,002964504	0,005163045
FZR1	0,229155048	5,749241757	0,002965022	0,005163045
LRIG2	-0,275792138	3,579040725	0,002981458	0,005190341
LMTK2	-0,445556348	5,20167327	0,002985007	0,005195192
LRWD1	0,321677433	4,773452363	0,002986168	0,005195887
PHF5A	0,366752045	5,405527573	0,003007425	0,005231539
EIF3D	0,246747372	8,034604633	0,003030915	0,005271057
ZNF33A	-0,296710085	5,734444068	0,003036769	0,00527989
ATR	0,27243558	5,295450256	0,003046578	0,005295593
KLF3	-0,277023146	6,874598758	0,00307068	0,005336129
MRE11A	0,275128417	4,781147449	0,003080557	0,005351929
AKT1S1	0,277932467	5,866052923	0,00308164	0,005352446
CDC25B	0,467141376	7,097064431	0,003082509	0,005352592
TRERF1	0,419990169	4,38027395	0,003086863	0,005358788
IGFBP7	-0,415145157	8,870978968	0,003107083	0,005392518
XPNPEP1	0,242452235	6,104588945	0,003108351	0,005393345
KRR1	0,241689111	5,719100358	0,0031112	0,005396915
GABPB1	0,230697466	4,950934403	0,003142701	0,005450173
ALG14	0,34111985	3,872963117	0,003147645	0,005456667
TMEM45A	0,411685721	7,113728917	0,003148046	0,005456667
C12orf10	0,303748241	5,560064898	0,003157157	0,005471068
RGS2	-0,65917363	6,756439138	0,003158163	0,005471421
NDUFV3	0,251864913	5,573147205	0,003165337	0,005481316
ITGA7	-0,472294966	3,65106342	0,003165482	0,005481316
PDCD7	-0,176525144	4,854874658	0,003185589	0,005514733
LRP11	0,3310977	6,462823075	0,003190938	0,005522591
TMEM126A	0,356737816	4,536969081	0,003193975	0,005526445
TIPARP	-0,576370237	7,084365074	0,003213217	0,005558328
TMUB2	0,215618505	6,015478353	0,003235025	0,005594632
PRKD3	-0,293299724	6,28494	0,003237206	0,005596984
SLC17A5	-0,284456594	5,726792656	0,00323837	0,005597577

ZCRB1	0,263779433	6,088695877	0,003248409	0,005613508
SF1	-0,192705574	8,063801957	0,003288739	0,005681762
KCTD6	0,30996461	3,322177651	0,003291433	0,005684974
VPS4B	-0,229424842	6,511394282	0,003308133	0,005712373
SSNA1	0,334034058	6,172778707	0,003316865	0,005726
EIF2C2	0,388358668	4,173060144	0,0033225	0,005734276
CCDC109B	0,456306402	4,67440131	0,003334127	0,005752888
RABGEF1	-0,183573503	5,837744778	0,003358176	0,005792917
KIAA0430	-0,283853805	6,798959393	0,003379168	0,005827655
IGSF8	0,416333945	6,387454876	0,003384582	0,005835516
ST8SIA4	0,484261113	5,782510484	0,003395611	0,005853051
PTOV1	0,251479405	6,884803663	0,003398419	0,005856411
DUSP5	0,582286741	5,976886993	0,003412073	0,005878455
PI4KAP2	-0,299383972	4,366618979	0,003414094	0,005880451
SIRT1	-0,245307653	5,36817142	0,003421795	0,005892227
SNHG11	0,311473373	3,336563406	0,003433921	0,005911614
C12orf65	0,2635272	5,042673231	0,003467705	0,005968268
EIF2S1	0,262676828	6,799176253	0,003482375	0,005992005
TBC1D22B	0,219299898	5,061129924	0,003490294	0,006004117
MAGED2	0,322292499	8,10849326	0,003521137	0,006055645
ZDHHC7	-0,30670968	6,934949851	0,003551996	0,006107177
GTF2H2	0,29889469	4,19112407	0,00355741	0,006114943
TMCO7	-0,232242761	4,411438355	0,003604064	0,006193577
RPL4	0,294593174	10,8365226	0,003626448	0,006230474
LCP2	-0,436067555	5,957042296	0,003631876	0,006238227
NDRG1	0,53751438	9,641517137	0,003635751	0,00624331
DPM1	0,320130296	5,570767147	0,003636922	0,006243749
HEATR3	0,25711608	4,618293572	0,003640862	0,006248939
ATXN2	-0,259253736	5,933848803	0,003665582	0,006289784
ALKBH5	-0,213042444	7,023984971	0,003666961	0,006290568
MXI1	-0,30282285	5,911282398	0,00370524	0,006354063
SLC38A10	0,281598858	7,791546195	0,003705838	0,006354063
VPS13B	-0,308611977	5,627124879	0,003718396	0,006373992
HPS1	-0,220318654	6,539901349	0,003719365	0,006374052
COG1	0,265306661	5,95506661	0,003732816	0,006395496
JRKL	0,299949526	4,47580488	0,003752169	0,006427038
TGS1	0,235500062	5,320678284	0,003754163	0,006428839
CDKAL1	0,236016889	5,00659159	0,00378277	0,006476201
TCTN2	-0,32345721	4,977810328	0,003804981	0,006512592
PHGDH	0,793408513	5,410304291	0,003826141	0,006547165
DCTN4	-0,224152149	6,75953049	0,003827142	0,006547236
FASTKD2	0,188334061	6,01738391	0,003835399	0,006559715
CNOT4	-0,17904643	4,834718993	0,003836562	0,006560059
NUCB2	0,334064124	6,827079949	0,003857553	0,006594298
GRB10	0,480028269	6,137261862	0,003859309	0,006595647
COX15	0,215794555	6,091865696	0,003877997	0,006625923
PACS1	0,287855859	7,281724718	0,003880489	0,00662852
IFITM2	-0,366890368	8,008048001	0,00390584	0,006670154
DVL1	0,346812108	6,595846748	0,003912379	0,006679648
ARL2BP	-0,231949339	6,799035142	0,003924053	0,006697901

LIX1L	-0,351444897	5,519640723	0,003930578	0,00670736
RAB13	0,20919643	7,22672227	0,003949107	0,006737292
DSEL	-0,472041752	4,572129004	0,003967104	0,006766304
TUBGCP6	-0,357920484	5,650396617	0,003980677	0,006787757
ZNF526	0,211392814	4,491495172	0,003992957	0,006806993
COX17	0,336055338	5,5679494	0,003996647	0,006811581
RNF40	0,246926881	7,217060874	0,004009382	0,006829977
BTN2A1	-0,241753129	5,391103079	0,004009444	0,006829977
CRTC2	0,230847934	6,328963177	0,004036391	0,006874163
SEPT6	0,389295938	6,010065962	0,004041539	0,006881212
SLC25A6	0,308568936	9,402923392	0,004055093	0,006902566
S100A4	-0,693351525	8,624822246	0,004077836	0,006939547
TRIP6	0,430680573	6,571424598	0,004085301	0,006950515
CDC37	-0,206648211	7,533144764	0,004113228	0,006996284
ALKBH1	0,224482025	3,95699323	0,004127826	0,007019363
TBCD	0,301254547	7,101497493	0,004133666	0,007027541
SLC6A6	-0,332120479	7,048060492	0,004163084	0,00707579
METRNL	-0,381884654	5,587179015	0,004178706	0,007100553
GRB2	-0,20033454	8,055490304	0,004179736	0,007100553
AKR7A2	0,291932891	6,061459535	0,004187431	0,007111853
C6orf57	0,481188249	3,269600538	0,004190038	0,00711451
EIF4G1	0,325338856	9,210082912	0,004194868	0,007120937
SLC19A2	-0,447161577	5,604276788	0,004203458	0,007133744
FUCA2	0,304958621	6,821588836	0,004220395	0,007159522
PLEKHM1	-0,304145145	6,0671447	0,004220747	0,007159522
CCRN4L	-0,313251802	3,419913967	0,004222547	0,007160793
USP33	-0,205278738	6,50878373	0,004257242	0,007217835
C17orf85	-0,286750633	5,112043634	0,004262218	0,007224475
PPP4R2	0,253754536	5,129402653	0,004314565	0,007311386
ZBTB8OS	0,302151311	4,320158289	0,004375099	0,007412125
ITGA9	-0,561791921	4,657411315	0,004376222	0,007412185
SP1	-0,215735543	7,258337812	0,004404535	0,007458288
RPS19BP1	0,313525268	6,171569887	0,004426029	0,007492823
ZMYM4	0,190865835	6,439316135	0,004445029	0,007522146
RNF141	-0,265473596	6,558391325	0,004445556	0,007522146
ELL	-0,245746921	4,943847564	0,004514711	0,007636395
MLL	-0,355124253	6,651202363	0,004515317	0,007636395
BSG	0,302505594	9,56661581	0,004517799	0,007638698
DHX16	0,224861826	5,999447356	0,004541374	0,007675863
FZD8	-0,552131516	4,460091267	0,004542031	0,007675863
C22orf28	0,205745708	6,852073579	0,004592106	0,007758565
HNRNPK	0,131005719	9,582541212	0,004597943	0,007766504
SUPT16H	0,275330346	7,416608857	0,004606776	0,007779496
SLMAP	-0,220715951	6,062889548	0,004612624	0,007787442
POLH	-0,229806505	5,462094127	0,004620047	0,007798045
DNM1L	0,338236865	6,535440043	0,004634507	0,007820515
KIF1B	-0,293272676	6,691784415	0,004636912	0,007822637
RAC1	0,251960753	9,233569943	0,004640704	0,007827098
PSAP	-0,274524054	11,64000489	0,004670107	0,007874743
STK38	0,252404878	6,48780899	0,004678755	0,007887374

FGL2	-0,531103912	6,336423263	0,00469729	0,007916663
LMBR1	0,238459686	6,062892752	0,004734754	0,007977832
RASA3	-0,397108169	5,025892728	0,004757169	0,008013621
MICB	0,435700718	4,110021913	0,004760407	0,008017094
RBM22	-0,170157134	6,05382236	0,004762961	0,008019415
HNRNPR	0,189268791	7,405567278	0,004785405	0,008055214
GTF2H3	0,287348932	4,301010551	0,004818538	0,008108986
S100PBP	0,226477943	5,122793047	0,004833195	0,008131646
ACAT2	-0,346789954	5,361161172	0,004851099	0,008159755
MX1	0,570023331	7,510370101	0,004877191	0,008200588
SLC3A2	0,338116999	7,82382672	0,00487778	0,008200588
RAB3GAP2	0,187472213	6,128721032	0,004888493	0,008216573
LOC727896	-0,28946377	1,589835693	0,004892319	0,008220977
PBX3	-0,316927568	5,308675454	0,004903954	0,008238498
COPS3	0,269485941	6,19847772	0,004910899	0,008248132
MAP2K4	-0,197316923	5,606226142	0,004950358	0,008312359
LENG8	-0,42400485	7,187917737	0,004962329	0,008327746
YWHAB	-0,166270668	8,916048944	0,004962689	0,008327746
TCTN1	-0,310722488	5,485921715	0,004964261	0,008327746
CMTM3	0,421448973	6,317902565	0,004964407	0,008327746
DCUN1D3	-0,358030907	4,45221772	0,004970714	0,008336277
PASK	0,384042958	4,18960117	0,004986678	0,008360992
STX4	0,251300131	5,871766378	0,004988143	0,008361393
BAX	0,237052267	6,045750853	0,005057899	0,008476239
HAS2	-0,726406257	3,610493741	0,005085518	0,00852043
PRKCSH	0,26170033	8,399029297	0,005086987	0,008520479
SHKBP1	0,259917251	6,734267645	0,005088047	0,008520479
GHITM	0,26765214	8,21844803	0,005091623	0,008522804
PDE12	-0,173734656	5,253402512	0,005091935	0,008522804
PPIL2	0,236889876	6,037688283	0,005093367	0,008523109
SPNS1	-0,215054723	6,28051391	0,005100034	0,008532171
FAM104B	-0,323817405	4,031578407	0,005111397	0,008549084
PARP8	0,321648422	5,283284192	0,005120765	0,008562652
ACPL2	0,334968153	4,133033531	0,005123974	0,008565918
ERLEC1	0,338245678	7,572452366	0,00512924	0,008572619
ME1	0,520044763	5,728172394	0,005135165	0,00858042
C9orf89	0,374572782	4,500389412	0,005171695	0,008639341
NUMA1	-0,322221923	8,657123485	0,005174975	0,008642702
ARHGAP1	0,247816391	7,63762216	0,005197569	0,008678312
PARL	0,26141517	6,068957169	0,005204971	0,008688543
RHOBTB1	-0,416251741	5,00612531	0,005237541	0,008740771
WBP1	0,285143679	6,33837063	0,005257235	0,008771083
PSMB9	0,562646192	6,794401944	0,005259246	0,008771083
JAG1	-0,490650233	6,389303279	0,005259563	0,008771083
LIMS1	-0,301292206	5,099320529	0,005294634	0,00882741
YAP1	-0,306854711	7,78272638	0,005320427	0,008868245
AFF4	-0,275198294	7,322712537	0,005353633	0,008921414
RAB40C	0,334275858	5,962831161	0,005372596	0,008950826
QTRT1	0,296981358	4,745377386	0,005390875	0,008979086
ASNA1	0,292820351	6,623098785	0,005409897	0,009008569

LOC550643	0,283472381	5,312659618	0,005412977	0,009011497
USP35	0,31263043	3,386869844	0,005425917	0,009030834
ERO1LB	0,583014344	4,944225763	0,00544125	0,009054145
CCDC85B	-0,431936766	4,508522454	0,005444896	0,009058002
TMX1	0,271943961	6,583028825	0,005482637	0,009118562
BBS7	-0,197653296	4,07215609	0,005504934	0,009153413
ABCF3	0,213389722	6,153969144	0,005508298	0,009156774
IFIT5	-0,325086126	5,796244143	0,005548404	0,009221196
UBE2B	-0,258170423	6,617510866	0,005568793	0,009251799
LOC441454	0,282665928	1,876478921	0,005569531	0,009251799
TRAM1	0,368126994	9,567581746	0,005603587	0,009306103
GULP1	0,554529723	5,648913008	0,005605668	0,009307292
IKBKAP	-0,2186944	5,773461032	0,005683305	0,009433898
ZMYM6	-0,180448696	4,9539187	0,005702003	0,009462632
SLC25A16	0,364719488	4,608712404	0,005703434	0,009462705
ATF7IP	0,342443079	5,920425257	0,005708762	0,009469241
ATP5L	0,361329136	7,809139483	0,005710428	0,0094697
RAPGEF6	-0,38943253	4,424956289	0,005730065	0,009499954
DEXI	-0,210336931	5,403689268	0,005742825	0,009518795
CAST	-0,266269606	8,618059686	0,005807104	0,009622999
LYN	0,365470583	7,347879368	0,005847818	0,009688112
ITPRIPL2	-0,253755432	7,022921758	0,005878166	0,009736025
RNFT1	0,438307616	4,957346962	0,005884998	0,009744973
LYPLAL1	0,337078089	4,978116302	0,005900145	0,009767684
F3	-0,597230963	7,549339208	0,005906566	0,00977594
SNX4	-0,198435187	5,996258562	0,005911358	0,009779784
TMX2	0,223979724	7,075350209	0,005911757	0,009779784
RBM12	0,162808597	6,897999776	0,005927825	0,009803988
ADNP	0,263310337	7,028281042	0,00595032	0,009838805
UBR2	-0,213131755	5,845200695	0,005987074	0,009897177
KDM4B	-0,258292363	5,618297104	0,005988799	0,00989763
COL16A1	0,464093978	5,778519903	0,006007287	0,009925779
CSTB	0,461698852	8,74831994	0,006015849	0,009937517
TMEM126B	0,403416137	5,673567591	0,006031618	0,009961153
C7orf31	-0,325161563	2,994889049	0,006059835	0,01000533
PDCD2	0,231180883	5,520106456	0,006072247	0,010023398
CLK4	-0,355533166	4,366768845	0,006085719	0,010043204
KIF5B	-0,220717498	8,218048482	0,006097019	0,010059418
CYTIP	-0,420902595	5,023476438	0,006108669	0,010076201
GBAS	0,368656339	7,054686325	0,006113043	0,010080976
ZNF607	0,358824614	3,363934244	0,006119373	0,010088976
SNX21	0,481897749	5,672129891	0,006143987	0,010127109
CDKN2D	-0,331973704	3,498369424	0,006155165	0,010142622
TCEA3	0,529507053	6,375335218	0,006156374	0,010142622
CLK1	-0,332905076	6,289428923	0,006168082	0,010159457
NRAS	0,294947563	6,920220117	0,006209524	0,010225246
SLC9A1	-0,365190449	6,684488509	0,006212504	0,010227683
BNIP3L	-0,269325972	7,779774978	0,006252625	0,01029125
CLP1	0,192796973	4,207742997	0,006259015	0,010299281
PNP	0,403694891	6,547909453	0,006266769	0,010309553

TSPAN15	0,381000441	6,991429653	0,006291295	0,010347405
ST20	0,420555362	2,154737534	0,00635878	0,010455876
SEP15	0,375276315	8,404147658	0,006360836	0,010456736
TAF5L	0,182637251	5,654965466	0,006376335	0,010479688
DNAJC7	0,196909128	6,619931258	0,006378514	0,010480743
LYZ	-0,628480113	10,60802721	0,006397477	0,01050937
MTRF1L	-0,179274213	5,268475587	0,006420755	0,01054507
IGF2R	-0,366619413	8,278503735	0,006429258	0,010556491
NINJ1	0,349728779	6,618578217	0,006434444	0,010562464
NT5DC3	0,389774391	4,61952047	0,006445228	0,010575263
MAPK7	-0,250481791	4,775758141	0,006445343	0,010575263
MAPRE1	0,204879921	7,733986651	0,006495835	0,010655544
TPP1	-0,295133499	8,554981805	0,006515947	0,010685966
SEMA4A	-0,430764644	6,08389277	0,006549951	0,010739148
AKAP1	0,304211305	6,934468063	0,006579071	0,0107843
MPV17	0,272247571	6,024051389	0,00660118	0,01081794
EDC3	0,258707723	5,593257253	0,006611081	0,010831562
LETM1	0,229824875	6,185001354	0,006634983	0,010868111
FAM54B	-0,213479591	5,937989454	0,006645087	0,010882048
HERC2P2	-0,464886057	5,243945078	0,006658091	0,010900198
PPP1R11	0,206615444	6,943967714	0,006659367	0,010900198
PELI2	-0,464457561	4,610750403	0,006688571	0,010945373
RNF139	0,297149537	6,170595597	0,006736424	0,011021035
CDC42EP1	0,468716401	7,506365963	0,006752598	0,011044846
UBE3A	-0,184056999	6,634949392	0,006774208	0,011077536
MTRR	-0,352156114	6,229676958	0,006779311	0,011083222
TRNP1	0,813961299	5,267305147	0,006795056	0,011106301
HIRA	-0,269665319	5,536119168	0,006855394	0,011202236
TEAD3	0,310991186	5,896353686	0,00687811	0,011236663
MAN1A2	0,299223901	5,562828395	0,007001557	0,011435597
PNMA1	0,348696631	5,806081188	0,007016734	0,01145764
RAD21	0,258081916	8,408951311	0,007031489	0,011478985
PMS1	0,208455468	4,124231888	0,007116376	0,011614785
GPR107	-0,205097541	7,429411394	0,007137823	0,011647001
UBXN2A	0,188098923	4,458157795	0,007163745	0,011686502
KHSRP	0,233606535	7,31728264	0,00717811	0,011707134
RPL32P3	-0,357255903	3,082998712	0,007255292	0,011830186
VWA5A	-0,420981161	6,148915945	0,007270147	0,011851574
GCN1L1	0,274578194	7,446893486	0,007330881	0,011947724
RIF1	0,337505532	5,501579633	0,007451807	0,012141155
FAM115A	-0,337078562	5,69842901	0,007453127	0,012141155
COMMD3	0,342854595	5,531985778	0,007468777	0,012163743
PLSCR1	0,36571963	6,823166204	0,007484756	0,012186855
RCHY1	-0,266445673	5,216724781	0,00755007	0,012290268
COBRA1	0,19478498	6,659684965	0,00756902	0,012318174
RSAD2	-0,458818938	5,216091349	0,007589347	0,012348307
MARCKS	0,336946505	7,982753642	0,007624482	0,012402514
SYK	-0,299555639	6,643796324	0,007626562	0,012402753
DUSP10	0,380767319	4,800962914	0,007628266	0,012402753
MED18	0,264646615	4,949982119	0,007655727	0,012444434

DIDO1	-0,249393564	6,593788593	0,007658208	0,012445501
NUP133	0,206118247	6,21014669	0,007663285	0,012450547
GABARAP	-0,220633742	8,702855806	0,007664965	0,012450547
PLXND1	-0,391974647	8,13872649	0,007688649	0,012486044
PSMB6	0,257358589	6,922176942	0,007708828	0,012515833
SCYL2	0,218975079	6,248783708	0,007762632	0,012600188
TRIO	0,417090024	7,147060375	0,007769514	0,012608356
ZBTB10	0,410740126	5,193166648	0,007816002	0,012678842
UQCR10	0,348684014	6,766169729	0,007816667	0,012678842
ARL6IP6	0,335336301	4,976163949	0,007832609	0,012701679
AKT1	-0,205127633	7,984361053	0,007838352	0,01270797
XAB2	-0,192704619	6,118528369	0,007882576	0,012776631
EVI5L	-0,229948254	4,75321772	0,007887949	0,012782301
LEMD2	0,221168624	5,855213057	0,007964597	0,012903442
UTP23	0,227417837	5,239210347	0,00797701	0,012920482
RFX1	-0,26556104	4,857193493	0,007980667	0,012923336
FAM160A2	-0,229699162	5,435903545	0,007992582	0,012939558
CSDA	-0,311723075	7,890685491	0,007997903	0,012945099
TRPS1	0,476621506	4,451556851	0,008045284	0,013018697
SYS1	-0,211253974	6,248997242	0,008069749	0,013055189
DCAF16	0,262732265	5,662118045	0,008128931	0,013147813
C16orf5	-0,320514333	5,476003056	0,008131358	0,013148619
KIAA1586	0,258377469	3,364173274	0,008200927	0,013257971
ISCA1	-0,235945212	5,582282025	0,008214241	0,013275823
EAPP	0,330924153	6,053650671	0,008215863	0,013275823
FBXO21	-0,238287758	6,350946481	0,008226464	0,013289803
ZNF780B	-0,279779346	4,219638565	0,008303625	0,013411278
DR1	0,235860668	6,338085317	0,008330271	0,013451128
CNST	-0,239983902	5,65824144	0,008469486	0,013672686
MIER2	-0,282665833	4,952983353	0,008503849	0,01372491
OPTN	-0,283070889	6,967260126	0,008524743	0,013755376
HEXB	-0,270158997	7,855898454	0,008564042	0,013815519
SMARCE1	0,218742361	7,592425605	0,008582084	0,01384135
MLKL	-0,328526506	4,658210591	0,008596303	0,013861005
SHROOM2	-0,434770315	4,563302242	0,008639156	0,01392681
PEA15	-0,24890818	8,296988428	0,008643184	0,01393001
FAM134A	0,176591529	7,451075286	0,008646145	0,013931489
CYFIP1	-0,206798085	7,674689375	0,008655813	0,013943298
ERGIC1	0,331515314	8,576948366	0,008657563	0,013943298
KCTD1	0,514358398	4,347455637	0,008675089	0,013968224
CTNNBL1	-0,201008658	6,009444469	0,008710611	0,014022109
PHKA2	-0,23131717	5,011372528	0,008720425	0,014034594
YLPM1	-0,311149392	6,60173345	0,008787302	0,014138889
PKIA	-0,576392423	3,482170137	0,008803771	0,014162047
FAM100A	-0,218828938	4,977628508	0,008892019	0,014300631
EGLN1	0,263041411	6,472786797	0,00890062	0,014311089
LYRM2	-0,209357051	5,981973684	0,008912788	0,014327275
IVNS1ABP	0,236322132	7,539795946	0,008919848	0,014335244
SUPT7L	0,18068114	6,109702956	0,008935811	0,014357516
LPCAT2	-0,328313538	5,499645635	0,008950386	0,014377546

TBCB	0,300022707	6,382014849	0,008955327	0,014382095
COG5	0,206985703	6,185673583	0,008966868	0,014397238
ANAPC2	-0,194227483	5,342591879	0,008988426	0,014428456
ARHGAP15	-0,393516553	3,872540448	0,009107179	0,01461564
STK38L	0,346402625	5,942431339	0,009180156	0,014729291
ZNF121	0,28912303	2,342476703	0,009206307	0,014767775
FBXO42	-0,170405055	5,059763569	0,009275495	0,014875259
PLXNA1	-0,334223963	7,144787294	0,009288454	0,014892538
ZNF670	0,344631681	2,468674211	0,009294435	0,014898625
STAT2	0,239307063	7,325744723	0,009303054	0,014908937
UBE2N	0,268553561	7,133843424	0,0093764	0,015022948
UQCRC2	0,229707767	7,654448266	0,009431335	0,015107417
MSH5	0,316990145	4,491693159	0,009523873	0,015252064
TRIM33	-0,222393824	6,063580546	0,009755524	0,015619375
AGXT2L2	-0,2755983	5,206103824	0,009800347	0,015687457
ARHGAP10	-0,300212947	4,653354656	0,009857882	0,015775849
DAZAP1	0,179637619	6,761894447	0,00987972	0,015807089
CASC3	-0,223193574	7,177696758	0,009907607	0,015847988
CTSZ	-0,315879197	9,176746284	0,010011754	0,016010823
EID3	-0,471873048	1,466074258	0,010017333	0,01601599
HLA-DMA	-0,443234912	8,179860374	0,010090406	0,016129038
ZNF484	0,347747493	2,870270294	0,010207908	0,016313036
CDC42SE2	0,246008209	7,148189869	0,010260245	0,016389342
ZNF673	-0,205425851	4,061461634	0,010260463	0,016389342
INPPL1	0,271558398	7,37832426	0,010265388	0,016393368
SLC35C2	0,203370072	6,295794615	0,010271073	0,016398606
GLO1	0,305163985	7,988570497	0,010278135	0,016406039
GNAI1	-0,433593947	5,30371231	0,010292571	0,016425239
ITCH	0,185488102	6,68538924	0,01036583	0,016538277
MON2	-0,213005053	5,849546555	0,010427273	0,016632415
POLR3GL	-0,24847537	5,458670975	0,010442242	0,016652397
RALGAPA2	-0,461998667	5,702356143	0,010540401	0,016805003
USO1	0,193163715	7,275996239	0,010568792	0,016844066
OSGEP	0,282770233	5,213187346	0,010569843	0,016844066
ZBTB25	0,24364042	2,978463987	0,010591141	0,016874063
FUS	0,215009067	8,155220355	0,010618422	0,01691224
CEP78	0,251620783	3,866445125	0,010622216	0,01691224
C12orf75	0,775415417	5,049163384	0,010622543	0,01691224
FAM102B	-0,324268075	5,114460522	0,010630199	0,016920477
RIPK2	0,362890091	5,400045158	0,010641297	0,01693419
TGFB1	-0,302400401	6,918916224	0,010659749	0,016959595
DDAH1	-0,354559934	7,358089795	0,010686233	0,016997766
FXYD5	0,42663105	7,437113937	0,010690006	0,016999802
MRPL43	0,285087835	6,371763889	0,010711471	0,017029966
GNA15	0,372752944	5,487096465	0,010727369	0,017051266
RPL5	0,374895214	10,43137075	0,010743185	0,017072426
MAP2K2	0,203559393	7,043386799	0,010772921	0,017115692
AIMP1	0,189197057	6,105677572	0,010785708	0,017132015
ZCCHC14	-0,268149992	5,89767657	0,010791738	0,017137145
CTNND1	-0,244907634	8,96705696	0,010793963	0,017137145

DYNC1H1	-0,294523597	8,99816953	0,010820419	0,017174809
ZNF669	0,202680421	3,423512618	0,010822724	0,017174809
RASAL3	-0,380242892	4,150571732	0,010873389	0,017251196
POU2F2	0,491990731	3,499307836	0,010911621	0,017307826
PRKRA	0,219447279	5,991098243	0,010923982	0,017323404
USP34	-0,247404547	7,170328473	0,010999264	0,01743873
CDK6	-0,437117221	5,350987141	0,011051005	0,01751669
WDR82	-0,175965024	7,441419839	0,011062156	0,017530292
OAZ1	-0,253462316	9,622630652	0,011089828	0,017570059
PTGS1	-0,384872027	5,16623236	0,011092719	0,017570558
SOCS4	-0,204364134	5,351518684	0,011103501	0,017583551
NOSIP	0,27210225	5,75761126	0,011155399	0,017661635
CDH11	0,476890093	7,174389111	0,011167002	0,017675903
SMAD2	-0,234055916	6,961434938	0,011203404	0,017729406
OCIAD1	0,234770597	7,521072806	0,011248904	0,017793375
FAM134C	0,202121673	7,082075631	0,011249045	0,017793375
TFCP2	0,214133673	5,253402852	0,011254929	0,017798436
MLL2	-0,360228864	7,206698091	0,011257465	0,017798436
ATP9B	0,354820609	6,619079142	0,011267736	0,017810545
SNX33	-0,23144458	5,938483114	0,011283676	0,017831607
SOAT1	0,293573905	6,742715488	0,011318998	0,017883282
TMEM179B	0,302574046	6,562990959	0,0113597	0,017943431
NUMBL	0,362694779	4,773690916	0,011363821	0,017945784
PDIA5	0,271111198	5,739918363	0,011412586	0,018018621
PRPF4B	-0,171657151	6,612512715	0,011423914	0,018029247
EMD	0,219728563	6,31407966	0,011424604	0,018029247
MED17	0,16377102	5,286331277	0,011456854	0,018075958
POFUT2	0,223707404	5,406293848	0,011533347	0,018192434
ABCD4	-0,188383751	5,377585567	0,0115894	0,018276622
KIAA1731	0,260718057	4,307118349	0,01174572	0,018518859
P2RX4	0,296940419	6,053523724	0,011770647	0,01855387
ELF3	0,470475106	8,864054278	0,011804995	0,018603711
PTDSS1	0,229754875	6,865322258	0,011811647	0,018609893
FHOD3	0,716456208	3,936239943	0,011823762	0,018624678
GPN3	0,271102883	5,20418628	0,011870526	0,018694023
HNRNPD	0,215147589	7,927111529	0,01189727	0,018728079
BAHD1	-0,279536887	5,910060579	0,011897645	0,018728079
EIF3C	0,237175826	7,658996029	0,012024234	0,018922975
SNAI2	0,647642223	4,301643781	0,01203989	0,018941198
SVIP	0,285499922	4,915186766	0,012041369	0,018941198
MRP63	0,283980361	5,991806734	0,012085863	0,019006803
REXO2	0,248397704	6,071564798	0,012099139	0,019023295
TLE1	0,282341845	5,50868009	0,012128698	0,019065374
PITRM1	0,219507956	6,813898445	0,012241666	0,019238516
METTL6	0,174686212	4,193562215	0,012309431	0,019340555
FAM8A1	-0,260403302	6,519769772	0,012325345	0,019361099
RNF217	-0,371948921	2,456395651	0,012369607	0,019426151
MED16	-0,20006138	6,152304733	0,012406373	0,019479406
UCP2	0,405826892	7,796343257	0,012412129	0,019480688
PIK3CA	-0,235274825	4,944319245	0,012412904	0,019480688

MGEA5	-0,213406647	7,391093866	0,012475536	0,019574478
TDRD7	-0,225365143	5,21121711	0,012484944	0,019584732
DLG1	-0,220676513	6,330595977	0,012507542	0,019615669
UBB	-0,214922265	10,14204755	0,012552086	0,019681001
UPF3A	-0,237797041	5,562291066	0,012599575	0,01975092
WIPI1	0,262552672	5,499181988	0,01268231	0,019876044
PTTG1IP	0,275650443	9,270963771	0,012700939	0,019900667
SFI1	-0,256390835	4,463830676	0,012719756	0,019925573
RC3H2	-0,260486759	4,607813415	0,012790241	0,020031385
FER	-0,23273053	3,198091522	0,012821083	0,020075078
ZNF124	0,266498382	2,967628092	0,01284944	0,02011486
GOSR1	0,211662462	6,743617048	0,012879448	0,020157209
GSPT2	-0,431567344	4,086736996	0,012906385	0,020194731
BCAT1	0,699472411	7,123192204	0,012914143	0,020202235
SRRM2	-0,315705712	9,297626668	0,012995349	0,020324606
RBM42	0,295164828	6,664279581	0,013058854	0,020419245
WARS	-0,410494737	8,857877119	0,013115736	0,020503485
HNRNPA1L2	0,181287566	6,517172257	0,013129151	0,020519752
COL6A1	0,455919024	8,625290597	0,013151727	0,020550327
TSNAX	0,268092085	6,255165855	0,013165123	0,020566547
TATDN2	-0,17386186	6,127768071	0,013181852	0,020587963
STK25	0,166549034	6,751440574	0,013199374	0,020610609
VTA1	0,204828771	5,884544907	0,013202899	0,020611394
RASSF4	0,401939996	6,279875773	0,013220235	0,020633733
ATRX	-0,263074442	6,689066386	0,013278761	0,020720337
LEPROT	-0,304923288	6,144509785	0,013341189	0,020812988
TTC5	0,292986245	4,161583138	0,01336728	0,02084892
SPIRE1	-0,33799434	6,031231283	0,013409397	0,020909828
RPS27A	0,312724452	9,857822872	0,01342968	0,020936669
ISG20	0,4157466	5,583752456	0,013444562	0,020955078
WDR36	0,187182345	5,576911661	0,013502863	0,021041138
MMP2	0,455078786	8,821797561	0,013550568	0,021110652
DHX9	0,165280776	7,951347705	0,013583336	0,021156867
RASL11A	-0,517406984	4,227527375	0,013588484	0,021160054
KLHL7	0,277686218	5,546346495	0,013640736	0,02123657
UFM1	-0,19543597	7,033402887	0,013674774	0,021280936
CDC27	0,163685245	6,488018749	0,013675475	0,021280936
TOP1	0,297301231	7,946030844	0,013694179	0,02130518
COX7C	0,28245702	8,184150418	0,01373474	0,021363411
PLEKHF2	-0,263623238	5,673188984	0,013768609	0,021411208
FAU	0,334121179	9,136640453	0,013805099	0,021463057
FBXO3	-0,235034204	5,447290346	0,01385139	0,021530118
GRWD1	0,198014816	5,687251665	0,013937468	0,021658977
TRIM41	-0,253299987	5,354842356	0,014007263	0,021762481
NDUFB7	0,338852634	6,464683261	0,014145328	0,021971981
RHOBTB3	0,437580763	7,034131681	0,014203021	0,022056571
RFX5	0,242235787	6,445293827	0,014288892	0,022184871
ATF2	0,219249944	5,076470115	0,014296445	0,022191545
COTL1	-0,338526288	8,021239019	0,014340528	0,022254906
ABCC5	0,470063757	5,887867922	0,014373004	0,022300231

AKT2	0,216362823	7,129875793	0,014395056	0,022329365
GALT	-0,195442737	4,824915721	0,014546423	0,022559031
ANKRD50	-0,321278037	6,120283292	0,014570904	0,022591859
SAT1	-0,302112823	9,433187855	0,014698154	0,022783977
CEP350	0,226854414	6,279840294	0,014725909	0,022821812
HMOX2	0,233430176	6,220866967	0,014731439	0,022825195
GPX1	0,370948339	8,884183462	0,014829705	0,02297223
BTBD3	-0,326898867	5,705435816	0,014846608	0,022993191
AKR1B1	0,514803501	7,186209241	0,014999386	0,023224526
CNNM3	-0,223307267	6,100859652	0,015015291	0,023243876
SCAP	-0,190923293	6,647387043	0,015035092	0,023269244
SCARB2	-0,200464329	8,876330627	0,015056262	0,023296721
TAGLN	-0,406506698	8,627321565	0,015068076	0,023309712
SRPX	-0,687699864	4,850434449	0,015094455	0,023345224
ZNF506	-0,274743714	4,746634905	0,015127032	0,023390302
FAM129A	-0,405784325	6,80088012	0,015137977	0,02340192
HDGFRP3	-0,392792363	5,05441703	0,01515275	0,023418513
GOLGB1	-0,274847399	7,493969177	0,015156661	0,023418513
SNX3	-0,240672169	7,74319263	0,015159014	0,023418513
LOC282997	-0,25769821	1,915820112	0,015240638	0,023539278
TMEM123	0,243234986	8,885067739	0,01526661	0,023574052
TPM1	0,356261913	7,872532282	0,015362135	0,023716187
NRP2	-0,385932571	6,810485489	0,015367121	0,023718515
TP53I3	0,354404103	5,732130105	0,015398083	0,023760925
STX18	0,18689238	5,373354657	0,015427068	0,023800266
TREX1	-0,235026908	4,385087918	0,015445524	0,02382335
TXNDC11	-0,257358649	7,363779356	0,015496768	0,023896983
CNOT7	0,203023373	6,790254683	0,015520699	0,023927235
ATG7	-0,160370082	5,965607648	0,015523403	0,023927235
RPS11	0,331934297	10,79682452	0,01563435	0,024092798
C1QTNF3	0,685914107	4,176915704	0,015663087	0,024131629
BRWD3	0,349044798	3,826347793	0,015666773	0,024131855
MAP4K3	0,203499221	5,863246803	0,015716472	0,024202941
HTRA1	0,395555716	7,079540698	0,01573561	0,024226942
PPP2R2A	0,297159256	6,351379786	0,015757661	0,024255415
FDFT1	-0,30932183	7,669024829	0,015833811	0,024367131
POLE4	0,359405152	5,743718061	0,015850529	0,024387355
MLL4	0,274484262	6,318176484	0,015855149	0,02438896
FAM193A	-0,206352375	5,41449376	0,015904835	0,024457815
APTX	0,204001617	5,133142939	0,015907084	0,024457815
PPP4R1	-0,209184058	7,498422545	0,015922632	0,024476201
NRD1	0,183246644	7,458760834	0,015964989	0,024535781
PLVAP	0,404405804	6,785232339	0,015980264	0,024553723
THOC1	0,225803483	4,54580141	0,015996998	0,024573897
GALNT10	0,384840286	7,079041573	0,016019402	0,024602771
TAF7	0,24303439	7,508226495	0,016222074	0,024908426
THAP11	-0,156162597	5,472057492	0,016302839	0,025026803
ANKRD27	-0,242237693	5,879068503	0,016320506	0,025048285
RUNX3	-0,397636634	4,916477447	0,016425447	0,025203673
NF1	0,288737706	6,846207877	0,016470096	0,025266498

TNIP2	0,211423885	5,698121805	0,016491117	0,025293056
HK2	-0,407980431	6,979027775	0,016610994	0,025471187
CLU	-0,569861896	9,061720256	0,016691073	0,025586702
CTSD	-0,314467448	11,51093901	0,016693831	0,025586702
COX16	0,229770371	6,077624343	0,016712831	0,025610066
FAM160B1	-0,174130824	5,503057223	0,016745867	0,025654924
CSTF2T	-0,153202162	5,652246376	0,016750768	0,025656668
SCRN3	0,189836655	4,604530509	0,01676676	0,025675395
KLHL20	-0,208151095	4,993733279	0,016774516	0,025681505
HIC2	-0,326731753	3,212117105	0,016794772	0,025706745
ZFAND3	0,191003728	6,938709045	0,01690988	0,025873753
SP140L	0,244288376	5,070295958	0,01691147	0,025873753
YPEL5	-0,26093805	7,661556239	0,016985314	0,025980902
FUT11	-0,231665772	3,988595834	0,016999768	0,025997178
ATP5F1	0,253383865	7,637265371	0,017114573	0,026164003
CREBZF	0,258332865	6,250379959	0,01711653	0,026164003
ANKHD1	-0,231774108	6,806464725	0,017145281	0,026200749
PCGF6	0,181367849	3,942513455	0,017148536	0,026200749
PPARD	0,237064348	6,477980981	0,017152096	0,026200749
SEC24B	-0,157805356	6,010593997	0,017221284	0,026300545
TFE3	-0,239451784	6,715834617	0,017299807	0,02641455
PCDHB14	0,40806407	3,010390539	0,017316771	0,026431485
FKBP5	-0,46711031	7,407721244	0,01731865	0,026431485
AP1G1	-0,169964673	7,308901672	0,017329046	0,026441433
TOR1A	0,178563005	6,039029462	0,017449169	0,026618767
TIAM1	-0,431363843	4,521681017	0,017509861	0,026705378
IREB2	-0,190355333	6,279728903	0,017673217	0,026948495
SIGLEC10	0,39910953	4,616981753	0,017788615	0,027118392
UBE3C	0,185128314	7,088368066	0,017793833	0,027120284
USP22	-0,244081422	8,10848873	0,017812874	0,027143238
EIF2A	0,170715204	7,034105181	0,017874702	0,027226685
MDM2	0,432656032	7,684473953	0,017875622	0,027226685
PRPF31	0,173737573	6,162656704	0,017988388	0,027392322
GNPTG	0,247074129	6,60195227	0,01801278	0,027423342
WDR54	-0,37010068	4,573821242	0,018020231	0,027428561
ARL17A	0,346231499	4,620396966	0,018213313	0,027716265
SRBD1	-0,164080317	4,736230448	0,018246093	0,027759953
RPL9	0,421989193	10,98700314	0,018255877	0,027768643
ELAC2	0,174194444	6,258748294	0,018276334	0,02779356
RPS23	0,28281319	9,921910764	0,018285654	0,027801533
C3orf17	0,162281989	5,805210108	0,018353321	0,027898194
DHRS7	0,295019997	7,005277002	0,018398752	0,027961019
HEXIM1	-0,256786343	6,280862416	0,018404966	0,027964229
NECAP2	-0,214791954	7,069276058	0,018496417	0,028096918
LOC728190	-0,168601982	5,019326101	0,0185495	0,028171278
EXOSC8	0,254422678	4,512917225	0,018584671	0,028218408
ZNF436	0,216496308	4,712346913	0,018596264	0,028229724
PCID2	0,250351738	5,558950431	0,018668065	0,028332415
MLXIP	-0,278584082	6,260401591	0,018958801	0,028767259
ZDHHC8	-0,241981507	5,472259089	0,019051124	0,028900915

MATR3	-0,124479012	8,595333326	0,019139637	0,029028733
PRPSAP2	0,22065266	5,230861691	0,019329903	0,029310787
PPP2R1A	0,16608695	8,378836866	0,019399132	0,029409222
ZNF148	-0,147408894	6,157570035	0,019465181	0,029502793
RRBP1	0,323024543	8,834168031	0,019558998	0,029638402
DUSP14	0,308735066	4,838784402	0,019626257	0,029733714
PVR	-0,302991198	6,546689591	0,019714126	0,029860202
MTF2	0,214589467	5,039594754	0,019740406	0,029893367
ADAM10	-0,233695834	6,916031592	0,019749733	0,02990085
ELN	-0,528155188	7,234131596	0,019768088	0,029921996
NDUFA4	0,345093603	8,129196771	0,019853683	0,030042813
INVS	-0,185063274	3,783568937	0,019856718	0,030042813
IFNAR2	0,261233453	5,866546473	0,019893641	0,030092001
GPC1	0,444459885	6,414720592	0,019905897	0,030103862
LUM	0,451036919	9,722814301	0,019915294	0,030111395
NFU1	0,27656115	5,484947097	0,019976634	0,030196707
MKL2	-0,27035076	6,396070906	0,019980575	0,030196707
SEC24C	0,172416052	7,659745199	0,019996083	0,030213448
MT1X	-0,563144529	5,926609954	0,020058391	0,030300879
HNRNPH1	-0,193174158	8,193109595	0,020162332	0,03045115
LOC100170939	-0,308509866	2,162649679	0,020225484	0,030539765
SPHK1	0,507489393	4,34469138	0,020318895	0,030674019
TMEM19	-0,21058384	5,894579543	0,020470866	0,030896599
FBN1	0,468108238	7,373516424	0,020499827	0,030933463
ZNF24	-0,245795459	6,924404885	0,020525205	0,030963159
PTPRA	-0,183132173	6,850149515	0,020528588	0,030963159
BAP1	-0,156953936	6,577585079	0,020598158	0,03106122
AGPAT1	0,180157562	6,888211793	0,020632387	0,031105958
PFN1	0,200407556	9,708045625	0,020654817	0,031128858
MED25	-0,211947201	5,94644802	0,020656707	0,031128858
RAB7A	-0,166310739	8,665139937	0,020696903	0,031182541
SOD2	-0,38171268	9,069844913	0,020774944	0,031293206
NDUFA3	0,273630391	5,719617138	0,020792121	0,031312163
NTAN1	-0,225034128	5,142988645	0,020808523	0,031329945
FUNDC2	0,337829843	6,952088281	0,020900464	0,031461427
SUMO3	0,255084407	7,512122895	0,020947066	0,031522847
TAGLN2	0,295472994	10,74286948	0,020950512	0,031522847
ETFDH	-0,185923972	4,492634557	0,020956765	0,031525299
CUX1	-0,235238055	7,27348393	0,021054083	0,031664709
CLPX	0,181681607	5,475173605	0,021074778	0,031688845
POLA1	0,23761172	4,701164309	0,021084619	0,031696652
MFSD6	-0,243433775	6,861032442	0,021098935	0,031711183
KLHL8	-0,180872418	4,95751553	0,021174471	0,031817699
PRKAR2B	-0,415466947	4,532298205	0,021284508	0,031975999
TLE3	0,245788288	6,586287727	0,021319063	0,032020856
SIX5	0,236653654	4,817619261	0,021423867	0,032171184
F13A1	0,626040537	6,79568725	0,02147133	0,032235358
ARHGEF35	0,321076586	4,675319729	0,021515088	0,032293943
MTMR3	-0,236501825	6,499785818	0,021645057	0,032481876
RAB3IP	0,603502101	6,29122778	0,021795473	0,032700403

FAM120A	0,178379424	8,252963695	0,021830414	0,032745622
VAV2	0,26491413	6,164194788	0,021886266	0,032822179
WDR60	-0,235436287	4,840578049	0,021891368	0,032822612
BPGM	0,305183974	5,456002044	0,021965529	0,032923661
C10orf137	0,228356999	4,418228421	0,02196842	0,032923661
KDM4A	-0,193644532	6,243935417	0,022041031	0,033025224
RPS25	0,244552026	9,671142838	0,022063424	0,033051513
HIGD2A	0,263203712	6,290208473	0,022322434	0,033430132
ANXA2P2	-0,338023144	6,446653135	0,022325975	0,033430132
MTSS1	-0,373855106	5,923553831	0,022366028	0,033482753
EWSR1	-0,165598889	7,978887838	0,022386173	0,033505557
CTSH	-0,560059048	10,15693261	0,022406572	0,033528729
BRD1	-0,224615205	5,763189431	0,022417648	0,033537942
RPL23	0,25181559	9,858452424	0,022575308	0,033766402
KIFAP3	-0,208454246	5,83743546	0,022943781	0,034310009
NEDD4	-0,274612298	4,58211331	0,022965606	0,034335116
PLEKHA5	0,29302493	5,904601768	0,023008539	0,034391764
PPP2R1B	0,257986452	5,745610833	0,023116334	0,034545317
SH3RF1	-0,283983704	6,237048613	0,023163461	0,03460816
ATP6V0B	0,333242967	7,73613411	0,023461116	0,035045202
SMC6	0,202553017	5,345196373	0,023503133	0,035100276
FN3K	0,309871259	5,039362803	0,023556388	0,035172107
DDX20	-0,155399996	4,578298804	0,023697772	0,035375462
FAM178A	-0,167020242	5,512660897	0,023746381	0,035440265
SMARCD3	-0,335701246	5,169347147	0,023797427	0,035508678
OTUD6B	0,234198666	4,15611237	0,023811701	0,035522203
PPP2R5C	-0,145205473	7,37245588	0,023855712	0,035580074
H2AFY2	0,463142486	4,422151102	0,023936992	0,035693495
CYBASC3	-0,167954966	6,251934712	0,023967352	0,035730952
NDST2	-0,180755394	5,176016409	0,024069046	0,035874716
UBE2E1	0,213079995	6,766699382	0,024310805	0,036227137
HS2ST1	0,241682325	6,234181508	0,024353856	0,036283362
TAF9B	-0,206422888	5,280264769	0,024397856	0,036340974
HSP90AA1	0,212658316	10,60488831	0,024521913	0,036517783
COX4I1	0,307717034	8,52568887	0,024578372	0,036593839
TMEM203	0,196342637	5,731693133	0,024583719	0,036593839
NDUFA6	0,272131089	6,296544035	0,024638391	0,036667217
KLHDC5	-0,27932085	5,33505787	0,02468535	0,036729086
SNHG12	0,364676318	4,075852627	0,024705047	0,036750375
POLRMT	0,231702492	5,671188993	0,024902751	0,037036393
KIAA0664	0,269508598	6,574136826	0,024911353	0,037041107
TPM4	-0,251722605	9,72750532	0,024931636	0,037063184
ZSCAN29	-0,183370409	4,924202667	0,025025551	0,037194689
RAB32	-0,339770926	5,538875031	0,025041149	0,037209761
ZNF211	-0,260106634	4,038509848	0,025170476	0,037393786
RAB27A	-0,302772739	6,691379697	0,025220265	0,037454879
BTBD1	-0,146006635	6,494835658	0,025222585	0,037454879
TIA1	0,264114096	6,410742019	0,025287329	0,037542847
RPP14	-0,131201271	5,018490623	0,025335352	0,037605956
GPNMB	-0,44973572	8,961875923	0,025353743	0,037625065

ARAF	-0,168201108	6,837833524	0,025368271	0,037638433
CXorf56	0,203069369	4,055386042	0,025443451	0,037741764
SNHG5	0,474481465	7,020257646	0,025521609	0,037849468
ITGA1	-0,38850588	6,461405873	0,025664073	0,038052471
GNAI3	0,164042199	7,158714168	0,025707486	0,038108553
HAT1	0,166625083	5,932788041	0,02581924	0,038264327
MANBAL	0,202044221	6,314791743	0,025823792	0,038264327
CWF19L1	0,234609862	5,331846151	0,025899117	0,038367603
ITGA6	-0,441654469	7,061623547	0,025911243	0,03837723
CREBBP	-0,231938966	7,204878515	0,025921961	0,038384767
PAM	0,343930905	8,476566991	0,025933487	0,038393498
CDC23	0,143454121	5,521912515	0,026047224	0,038553509
CCDC47	0,199804749	7,822163235	0,026098172	0,038620537
GTF3C5	0,188787986	6,003362916	0,026327719	0,038951771
MBD1	-0,152596912	5,92385216	0,026533262	0,039247357
EIF5A2	0,28990665	3,773376383	0,026558777	0,039276578
SUCLG1	0,24061494	6,664461757	0,026747633	0,039547292
BIRC3	0,495815753	6,479813964	0,026788492	0,039599117
PIGN	0,203751883	5,195971708	0,026957133	0,039839768
ELF4	-0,263733172	5,940329964	0,027088541	0,040025301
F11R	-0,231473174	9,06720577	0,027281389	0,040301515
NIN	-0,260083051	5,893138633	0,027351962	0,040397017
RND3	-0,381240861	5,82682939	0,027516534	0,040631278
MIOS	0,169562905	5,228850447	0,027538324	0,040654651
SPATA2L	-0,242654308	4,209190234	0,027559233	0,040676712
CDC26	0,185309484	4,840622328	0,027649647	0,04080133
PELP1	0,223896277	6,343039107	0,02769467	0,040858925
SNAPC1	0,255826269	4,568149879	0,027805455	0,041008091
CAPNS1	-0,213386173	9,062067503	0,027807804	0,041008091
ZNF282	0,204638956	5,474559203	0,02790378	0,04114073
GYS1	0,204807295	6,134700078	0,028154385	0,041501244
ZNF180	-0,208082734	3,38368565	0,02824073	0,041619524
GLA	0,269169371	5,415660605	0,028321748	0,041729904
TRIM65	0,190596323	5,404512822	0,028343	0,041752196
CD63	0,263840213	10,49215398	0,028395812	0,041820959
CAPN2	-0,259534451	9,464031536	0,028406165	0,041826138
TNFRSF10B	0,280217081	6,991491071	0,028411596	0,041826138
RPL6	0,22216981	10,05995789	0,028526779	0,041986641
PPP2R2D	0,17324126	5,318979121	0,028624251	0,042121011
TRRAP	0,30788889	6,873929731	0,028691843	0,042211365
MTMR1	0,182629176	5,719397719	0,028792975	0,042351013
POMGNT1	0,199708105	6,875341015	0,028854071	0,042431725
AHCYL2	-0,467649185	7,90521744	0,028928759	0,042532387
MRPL50	0,219679452	4,918601259	0,02919996	0,042921864
SLC38A1	0,375897447	7,12009219	0,029231041	0,04295829
RGMB	-0,289009753	5,315230163	0,029386265	0,043177104
SLC9A6	-0,196740296	5,193064112	0,029534118	0,043384997
EXOC3	0,202141952	6,483367252	0,029540904	0,043385618
PI4KB	0,173414112	6,966956686	0,02960878	0,043475941
VPRBP	-0,177572548	5,817507839	0,029638076	0,043509589

APPBP2	-0,154918879	6,096165683	0,029822683	0,043771174
HNRNPA2B1	0,175384964	9,653824182	0,029853358	0,043806768
SYF2	-0,142874086	6,225388853	0,030168516	0,044259705
NEDD1	0,173513042	4,937517404	0,030289854	0,04442816
ANKRD52	0,28193918	6,244428751	0,030462224	0,044671378
PPP3CB	-0,150602495	6,212472195	0,030482488	0,044691483
GPKOW	0,2361166	5,178265675	0,03067823	0,044968799
FBXL4	0,175110851	4,253480797	0,030727797	0,045031774
POLR2J2	0,32992986	5,215032204	0,030922272	0,045307041
ITPR2	-0,319282384	6,060728801	0,031041746	0,045465805
LARGE	-0,401237366	4,78081871	0,031043964	0,045465805
RPRD1B	0,181076057	5,826610239	0,031065576	0,045482664
AP4B1	0,215657333	4,59385882	0,031068815	0,045482664
HGSNAT	0,309599289	7,483634133	0,031150724	0,045592786
UBE2J1	0,171941008	7,183329521	0,031319351	0,045829754
RBM17	-0,164030375	6,45292332	0,031362529	0,045883091
APOBEC3G	0,380596886	5,0320064	0,03137238	0,045887657
PPAP2A	-0,272403104	5,737624832	0,031382466	0,045892567
TAB1	-0,158489338	5,369297664	0,031399624	0,045907812
PIP4K2B	-0,179334712	6,827996762	0,031517455	0,046070209
BAZ1A	0,282283819	6,734384169	0,031524914	0,046071237
TRNT1	0,201122171	4,403011578	0,031630373	0,046215452
FTH1	-0,242475999	12,07864018	0,031680753	0,046279146
PGAP3	0,25626037	6,165803555	0,031772658	0,046403461
SARNP	0,237465291	5,852027941	0,031842237	0,046495121
UBA52	0,200225028	9,104679521	0,03203607	0,046768136
NSFL1C	0,176525491	6,772135685	0,03210241	0,046854951
KIF16B	-0,250117597	5,311462849	0,032166366	0,046938252
SUDS3	0,221895844	6,316445262	0,032180463	0,046947659
EXOSC10	0,141859416	6,416855284	0,032186583	0,046947659
STRADA	0,153125008	5,226402001	0,032334156	0,047152825
HNRNPA0	-0,167893101	7,731052721	0,032384234	0,047215757
ENDOG	0,269950619	3,695456049	0,032471215	0,047332454
ATPAF1	0,153846545	6,041956602	0,032497944	0,047361292
MICALL1	-0,250496027	5,582522646	0,032560296	0,047442021
SH2B1	-0,222250683	5,75781969	0,032617505	0,047515225
MRPL23	0,240798906	5,632179444	0,032736698	0,047678672
NHP2L1	0,186651169	7,260992623	0,032771129	0,047718627
RING1	-0,143210188	5,936828591	0,032987861	0,048023959
PI4KAP1	-0,272323572	4,359994548	0,033017648	0,048057064
KIAA1598	-0,25589957	6,417377855	0,033178235	0,048280492
PSME1	0,241891885	8,283916384	0,033216734	0,048326202
ASAP1	-0,281259295	6,190493968	0,033325624	0,048474281
ARPC1B	0,254297145	8,636485989	0,033553745	0,04879569
GLCE	0,257914566	5,915311791	0,033820276	0,049172806
CLPTM1	0,183553529	7,573386489	0,034025217	0,049460233
HNRNPU	0,176270088	9,263406212	0,034117013	0,049583101
RB1CC1	-0,173644268	6,628867241	0,034129643	0,049590888
TRIP10	-0,223582954	6,299100626	0,034151848	0,04961258
RNF41	-0,160773263	6,201478875	0,03425218	0,049747735

UBE2D3	-0,165094166	8,633743975	0,03432594	0,049844247
IGFBP5	0,477438746	9,056431656	0,034449695	0,0500133
ARFGAP3	0,202340295	6,597148481	0,034530871	0,050120478
KDM5C	0,224296508	7,282087923	0,034552787	0,050141616
OXCT1	0,388517881	5,255369494	0,034656106	0,050280848
ZNF564	-0,192066627	3,905354397	0,034664866	0,050282859
PLBD2	0,219506276	5,937171311	0,034721028	0,050353613
FLYWCH1	-0,176745278	5,116306129	0,034755127	0,050392348
TACSTD2	0,407984245	8,824843266	0,034858704	0,050531782
TMBIM1	-0,251680473	8,675619326	0,035171149	0,050972323
YWHAH	-0,163094274	7,915406757	0,035177555	0,050972323
CMTM7	-0,276594171	5,371487744	0,035193619	0,050984764
CASP4	0,285168959	6,938719761	0,035276776	0,051094378
NARG2	-0,161143174	5,643912578	0,035548344	0,05147678
GLUD1	-0,17697513	7,826574881	0,035606339	0,051549815
MARCH7	-0,143591838	6,897628929	0,035616706	0,051552261
SIN3A	-0,158355385	6,669471189	0,035623149	0,051552261
CCNT2	0,163299056	5,617792347	0,035768701	0,051751914
RNF167	-0,150770598	7,01259163	0,035992294	0,052064373
RBM25	-0,167615861	6,678742602	0,036038335	0,052119916
UBR3	-0,211847007	6,062113531	0,036122022	0,052229871
IFI27	0,494637903	8,972105858	0,036245114	0,052396742
TCF12	-0,178730996	6,391039406	0,036272143	0,052424701
MKRN1	0,144437682	7,439242393	0,036614861	0,052908824
UHRF2	0,305135577	5,449579947	0,036626737	0,052914771
ENTPD4	0,205914503	5,282198335	0,036917358	0,053323335
ROGDI	-0,231614829	5,262744147	0,036958638	0,053371654
OCEL1	-0,24157948	4,449526719	0,036994999	0,053412852
FBXW11	-0,185854579	6,41632094	0,037038466	0,053464289
DCP1B	-0,20861782	4,089534334	0,037146185	0,053608431
QRICH1	-0,12943032	6,429527998	0,037331842	0,053864967
ZNF45	-0,197282556	4,009644583	0,037382576	0,053926759
PPCDC	0,245907786	3,862486458	0,037535174	0,054135438
SAMD4B	0,209313992	6,84243729	0,037798605	0,054500098
HSPBP1	0,227061283	5,694630081	0,037803998	0,054500098
RPRD1A	0,264543752	6,409171253	0,037836571	0,054535527
MLH1	-0,133053616	5,562434309	0,037860637	0,054558683
ZNF302	-0,236889544	5,018847805	0,037913325	0,054623064
TMEM189	0,21704218	6,075618027	0,037957358	0,054674952
POLR2J3	0,316500392	5,769421292	0,037974815	0,054680915
STC1	0,616448912	5,704293941	0,037977535	0,054680915
ZNF410	0,224501072	6,369965452	0,038134597	0,054895465
ZNF585A	0,223575554	3,258048811	0,0383171	0,055146539
SIPA1	0,201291075	6,360727818	0,038335315	0,055161113
SPRED1	-0,268691644	7,169508524	0,038420477	0,05527199
CD58	-0,304841941	4,72949062	0,038942459	0,056011101
MED29	0,213080742	6,709812486	0,039045351	0,056147248
CCNDBP1	-0,240269169	6,311409074	0,039056806	0,056151878
SFPQ	0,171668485	8,116420926	0,039095206	0,056195239
NAPEPLD	-0,200116792	4,904730995	0,039186329	0,056314348

TMLHE	0,32148203	3,659874429	0,03920501	0,056329322
SLC35A5	-0,18331234	5,73349619	0,039222359	0,056331186
GPSM3	-0,27966802	6,021770481	0,039222829	0,056331186
FRS2	0,402925369	5,838750973	0,039385911	0,05655349
STK17B	-0,261876497	7,213574842	0,039404768	0,056568655
PCMT1	-0,16664141	6,3982131	0,039444398	0,056613629
ST3GAL1	-0,359791555	7,043495167	0,039482821	0,056656852
CREB3L1	0,548826758	7,108363922	0,039627255	0,056852146
PHF1	-0,171538909	6,209530305	0,039717176	0,056969168
SFXN3	-0,249169004	6,479617318	0,039978485	0,057331922
PLAUR	0,387807946	6,974354747	0,040050914	0,057423714
DGKD	-0,299168766	6,209765429	0,040091644	0,057470027
IPO8	-0,187564271	6,427132313	0,040117191	0,057494561
ZNF101	0,192413369	3,290891494	0,040166851	0,057553637
PRDM4	-0,152879885	5,591073348	0,040329448	0,057774475
MORC4	0,202574048	6,41584623	0,040616429	0,058173373
NSMCE4A	-0,200305156	5,060239748	0,040641267	0,058196723
SEPT7P2	-0,195623053	3,728571821	0,040650264	0,058197386
XPNPEP3	0,179528433	4,24100541	0,040862793	0,058489375
ANKRD9	0,293905122	2,992513805	0,0408797	0,058501296
THRAP3	-0,14568177	7,666261576	0,041128676	0,058845246
HERPUD2	-0,130939915	5,874432758	0,041234728	0,058984605
OSBPL3	0,274491722	5,735544466	0,041399656	0,059208107
VCP	0,147086861	8,819645991	0,041441448	0,059255449
ILKAP	0,178533749	4,918484114	0,041505325	0,059334342
MTX3	-0,202300567	4,959761877	0,041595714	0,059451095
ATE1	-0,214030562	3,987029355	0,041611436	0,059461103
METTL4	0,204528659	3,99325144	0,041675263	0,059539832
MKLN1	-0,150101755	6,523151306	0,041737223	0,059615861
LPAR6	-0,308413976	4,828006089	0,041813266	0,05971197
G0S2	0,611518692	5,672740488	0,041877244	0,059790814
SLC7A6OS	0,138878134	3,282407192	0,041945318	0,05987547
TARBP1	0,222506412	5,293586099	0,042007688	0,059951951
WSB1	-0,323855143	8,122123594	0,042132929	0,060118108
C6orf1	0,262559832	5,046680531	0,042170957	0,060159782
ARMCX3	-0,178716636	6,415512083	0,042241476	0,060247777
TMEM161A	0,191968808	5,085800109	0,042523624	0,060637513
SPOPL	-0,153741325	5,713461507	0,042617319	0,060758415
TULP3	0,206478312	5,277281181	0,042666967	0,060816481
UNC50	0,213901073	5,763849018	0,042682224	0,060825515
STK35	0,216900571	6,040622415	0,042725876	0,060875
ITGA4	-0,315367821	5,243515729	0,042864259	0,061059407
MED22	-0,181784327	5,468440399	0,042930383	0,061140828
AGPAT6	0,247271953	6,953315466	0,042990467	0,061213614
FAM149B1	-0,151831887	4,912155135	0,043004885	0,061221359
PEX3	-0,22665762	4,423629157	0,043046911	0,061268396
KLF16	0,258136005	5,062755417	0,043069011	0,061277357
SMARCA1	0,284261544	6,568859323	0,043071179	0,061277357
ISOC1	-0,176323965	5,213790202	0,043317828	0,061615409
PPRC1	-0,221567477	6,197881788	0,043507553	0,061872368

GIGYF2	-0,176484777	6,613570974	0,043557537	0,061924336
GPS2	-0,160479532	5,545347424	0,043562259	0,061924336
NFE2L1	-0,190185797	8,666845728	0,043703146	0,062111662
MYNN	0,224495853	5,005167579	0,043728636	0,062134938
RDH11	-0,177574113	6,883302519	0,043852631	0,062298143
SUMF2	0,257187861	8,314454188	0,044018958	0,062521407
LOC349196	-0,273189598	3,48243041	0,044062369	0,062570032
PTPRJ	-0,246840625	6,443554209	0,044213076	0,06277097
ZNF320	0,213521488	5,100166102	0,044257937	0,062821581
MEX3C	0,186148854	5,70703095	0,044629389	0,063335651
UBE2D2	0,145536372	6,919478072	0,044846594	0,063630654
GBP5	0,495622455	5,639730568	0,044867459	0,063647016
TTL	-0,219079886	5,743317219	0,045025803	0,063858351
DNAJB9	0,285730397	6,672180043	0,045069576	0,06390163
PHF20L1	0,155390116	5,901440646	0,045080154	0,06390163
CTNNB1	0,216082302	9,602039016	0,045084432	0,06390163
PDPR	-0,258408547	5,71992109	0,045284486	0,064171843
CDC42BPA	0,355595127	7,066910867	0,045325165	0,064216144
MRPS22	0,188073279	5,134687578	0,045500291	0,064450869
PSPC1	0,157326276	5,60045112	0,045600846	0,06457989
EIF2C3	0,198617861	3,192621198	0,045745253	0,064770946
IL7	-0,336272439	2,797799872	0,045760804	0,064779515
ZBTB2	-0,153007191	4,802705858	0,045773009	0,064783343
ZNF280D	-0,195210119	5,156786863	0,045861382	0,064894951
DHX33	0,233163556	4,091735505	0,04588002	0,064907854
ZUFSP	-0,146800138	3,375590213	0,046038013	0,065117861
ZNF197	-0,166700211	4,375959782	0,046140625	0,065249466
TMEM175	-0,197435568	4,903431288	0,046166909	0,065271151
APOOL	0,202908817	3,630014239	0,046175104	0,065271151
VHL	0,18345654	5,53939636	0,046438362	0,065629677
BCAR3	-0,283704914	5,130670607	0,046567971	0,065799212
EFR3A	0,178434855	7,306178387	0,046675497	0,06593748
ITGB2	-0,355433158	8,342914549	0,046732656	0,066004552
NUP214	-0,178179345	6,473604917	0,046758106	0,066026822
IPO5	0,192713202	7,715500047	0,046851303	0,066144727
TMEM57	0,176360062	5,926503716	0,046997114	0,06633685
OBSL1	0,368512677	6,282102224	0,047020585	0,066356244
CELSR2	0,413780844	4,899065062	0,047407009	0,066887729
RAB43	0,193612996	7,041917768	0,04757258	0,067103089
ANXA2	-0,294731424	10,82816483	0,047579328	0,067103089
PEX26	0,185102624	4,682241227	0,047724228	0,06729353
UPK3BL	0,385976651	4,607998343	0,047815267	0,06740796
CCNY	-0,154568515	7,199423236	0,047849902	0,067442844
SLC12A2	0,387573278	6,613647933	0,047938726	0,067554075
TRIM5	-0,183125008	5,476695582	0,048002806	0,0676304
GTF2F2	0,168248676	4,959824438	0,048098628	0,067749155
GDE1	0,214056119	7,681862837	0,048106967	0,067749155
DPH1	0,147598301	6,078319086	0,048130303	0,067768024
NBAS	-0,155030156	6,122514197	0,048161139	0,067797442
FAM46A	0,351142389	6,239211599	0,048387193	0,068101604

WARS2	0,181061923	4,497097579	0,048407212	0,068115719
CNOT3	0,179859593	5,960379244	0,048461396	0,068177895
SEC23B	0,191561275	7,025906777	0,048558073	0,068299814
LAP3	-0,201127364	7,620989687	0,048618616	0,068370869
BCL10	0,253528601	5,567920192	0,048645801	0,068394993
ACOT7	0,263600569	5,503678081	0,048865164	0,068689251
EML4	0,230462603	7,407016708	0,04903355	0,068911743
TUSC3	0,427953063	6,017209847	0,049049671	0,068920195
MANSC1	0,388369366	5,816693862	0,049108622	0,068988812
SH3GLB2	-0,191675009	6,245369314	0,049230876	0,069146312
SNX5	-0,149480887	7,185881267	0,049440411	0,06942631
DAGLB	0,199443776	5,543007537	0,049494967	0,06948861
PRPF18	-0,13349636	4,881846521	0,049522649	0,069513163
KIAA0226	-0,182173042	5,781365813	0,049533348	0,069513872
ZMYND8	0,224516941	6,258835962	0,049648327	0,069660893
WDR13	-0,172639437	6,732653704	0,049709403	0,069720478
NPL	-0,366895181	5,265091497	0,049711243	0,069720478
NT5C2	0,163844097	7,170557803	0,049886467	0,069951844
MIIP	0,202370402	5,124747699	0,050084492	0,070215081
CTNNA1	-0,178828496	9,441946057	0,050217588	0,070387201
CD2BP2	0,126347073	6,704183456	0,050241449	0,070406174
DYNLL1	0,203331443	7,773267053	0,050274869	0,070438532
MIDN	-0,26704416	7,456091937	0,050602786	0,070883401
TAF1B	0,170729793	4,315856989	0,050635685	0,070914919
NDUFB2	0,236405541	6,652166167	0,050667065	0,070944295
ACAP2	-0,229607228	6,469809671	0,050700002	0,070975839
PARD3	0,238863754	6,259994676	0,050795452	0,071094866
ARL8A	-0,140932885	6,463079405	0,050852957	0,071160745
AIP	0,210502466	6,593585612	0,05092022	0,071236609
ANGPTL2	0,357885433	6,29915881	0,050928064	0,071236609
ACOT8	0,200925642	4,872421663	0,051143681	0,071523535
SNRNP48	0,190920204	4,485065824	0,051210822	0,071602747
PCSK7	-0,189416889	5,759745259	0,05132757	0,071751271
PRKAR2A	-0,227480668	4,748399329	0,051539723	0,072033075
RPRD2	0,202506571	6,645008056	0,051576849	0,072065947
TNFRSF14	-0,224727789	6,383610403	0,051585763	0,072065947
PARG	0,140422883	4,944736926	0,051594949	0,072065947
PHF16	0,199206174	4,498207227	0,05167245	0,072159418
COG8	0,133547607	4,812330575	0,051685282	0,072162559
CDKN2AIP	-0,154180464	4,927323527	0,051741723	0,072226573
CSK	0,170589643	6,580832678	0,051840252	0,072349299
FCHO2	-0,236790682	6,663380057	0,051947927	0,072484739
RPS13	0,26220204	9,252860048	0,05215977	0,07276544
PNN	0,265797587	6,763158944	0,052283179	0,07291056
KCTD11	-0,188989168	5,036140999	0,052287851	0,07291056
SLC29A1	0,227119893	6,802985394	0,052295872	0,07291056
TSC22D4	0,169696618	6,24449607	0,05230803	0,072912604
ZMYM2	0,187926756	6,260246589	0,05242524	0,073061049
MFHAS1	-0,291452502	5,584669354	0,052520083	0,073178269
ING2	-0,189593894	3,829407907	0,052608178	0,073280682

VPS11	-0,133230989	6,253631539	0,052615079	0,073280682
KDM3A	0,171539626	6,344431482	0,052925966	0,073698624
POLR2A	-0,232325231	8,260641707	0,0529885	0,073770638
BTBD9	-0,316106297	6,349637263	0,053083294	0,073887525
ZNF830	-0,136910389	4,361553197	0,05317879	0,07400534
PRKX	-0,220553042	5,401658649	0,053304921	0,074165733
DIS3	-0,122721275	6,074579969	0,053690508	0,07468698
ADO	-0,1292703	5,232113662	0,053770435	0,074782908
POLR1E	0,200818549	4,71228946	0,053879546	0,074919377
DUS3L	0,187829698	4,525270291	0,054014224	0,075091334
RRP7B	0,189621279	4,301225462	0,054337538	0,075525412
C12orf57	0,326582598	6,825179204	0,05435194	0,075530034
AGAP6	-0,269195318	2,934169929	0,054427472	0,075619587
SUPT6H	-0,196955055	7,562669636	0,054780048	0,07609394
H1FX	0,205196824	7,146752532	0,054857898	0,07618656
FXN	-0,132850058	4,149874803	0,055006382	0,076377219
GUSB	0,174889959	7,252599848	0,055182269	0,076605842
BTAF1	-0,20277791	5,53634407	0,055279044	0,076724567
LRPAP1	-0,198053171	7,808189413	0,05534464	0,076799979
CRKL	-0,144783851	7,03165381	0,055378604	0,076824596
RP9	0,201518077	3,758334563	0,055396053	0,076824596
TTC14	-0,213674487	4,980301261	0,055396178	0,076824596
GPR108	-0,171459539	6,590401894	0,055466888	0,076907017
GOLGA3	0,2106579	7,167494567	0,055966786	0,07758437
SH3BP1	0,201315577	5,307174243	0,056272121	0,077991787
EDEM2	0,201052656	6,469511799	0,056373855	0,078116911
REXO1	-0,172524877	5,337385293	0,056717494	0,078577121
ALDH7A1	0,198822159	6,174273791	0,056871606	0,078774626
SBNO2	0,259334316	7,05981455	0,057205561	0,079221105
ZNF507	0,17257702	5,08650786	0,057253432	0,0792713
PGRMC1	0,20280555	7,733975247	0,057384619	0,079436808
C19orf66	-0,169677494	5,305829826	0,057588516	0,07970146
RWDD2B	0,217204737	4,776242481	0,057599178	0,07970146
FBXO31	-0,142791193	5,075641862	0,057659085	0,079768168
BCL9L	0,250952739	7,359287895	0,057760345	0,079892046
BRAP	0,126853507	5,182039475	0,057917592	0,080087695
PLK2	-0,336434053	6,121021309	0,057925284	0,080087695
PPP1R9B	-0,184875434	6,799972898	0,058110394	0,080327342
NUDT19	0,264410338	5,38897092	0,058170541	0,080394186
MDM4	-0,20132372	5,993479888	0,058444927	0,080757033
SLC38A2	-0,240899718	8,166658889	0,058537895	0,080869106
MYOF	0,298009414	8,389423034	0,058696265	0,081057508
BRD4	-0,193100642	6,760987019	0,058698045	0,081057508
RRAGC	-0,163228272	4,966996715	0,058743964	0,081104493
GABARAPL2	-0,204238292	6,956791262	0,058851943	0,081237126
PHLDA1	0,370633076	7,114270561	0,059060544	0,081508571
SF3B2	0,127109794	8,336848679	0,059104253	0,081552388
NDUFB8	0,209720648	5,865943106	0,059307987	0,081816946
MIER3	0,171168434	5,151940465	0,059367823	0,081882926
DGCR6L	0,213902575	5,743523239	0,059390456	0,081897578

C10orf118	-0,213621002	5,516230323	0,059418602	0,081919823
UPF2	-0,180214185	5,73674238	0,059570352	0,082112439
C14orf93	0,166518782	3,728216901	0,059597906	0,082133816
CCL4	-0,430561461	4,166163072	0,059626782	0,082157007
OSBPL8	-0,144441536	6,780936054	0,059763898	0,082318158
STON2	-0,320521842	3,562485039	0,059767884	0,082318158
RNGTT	0,141711292	4,846914934	0,059826012	0,082381578
BAG1	-0,243311508	6,972525163	0,059996506	0,082599672
DNASE2	-0,146824048	6,964287872	0,060195641	0,082857101
TMED10P1	0,162589597	1,802662981	0,060439527	0,08317601
LCORL	0,202975431	2,729055246	0,060579189	0,08335139
MAFB	-0,295326004	6,20022439	0,060615837	0,083384989
ERP29	0,201317838	8,210981925	0,060741427	0,083540902
ATP6V1E1	-0,15928082	7,476465807	0,060891439	0,083730334
TM7SF3	0,281243152	7,420114538	0,06102347	0,083894968
VPS29	0,253938679	6,651713291	0,061203697	0,084125783
FUT4	0,291500811	4,318447929	0,061438418	0,084431394
DNAJC21	0,153824723	6,634733762	0,061499113	0,084497774
ATP11C	0,227502523	4,976232631	0,061702779	0,084743888
CD151	-0,221059514	9,051025968	0,061711985	0,084743888
RPS9	0,267180158	10,28810675	0,061715523	0,084743888
PUM2	-0,116465468	7,603073827	0,062165135	0,085344081
CBWD1	-0,143355597	4,739439252	0,062439272	0,085703179
UBE2L3	0,140724152	7,313634162	0,062514254	0,085788831
GLRX	-0,317326838	6,242916357	0,062817049	0,086187013
CCDC104	-0,181010675	5,754240907	0,062886044	0,08626432
DDX1	0,121364517	7,142761032	0,063065684	0,086493342
NDUFA5	-0,151036766	6,494247648	0,063244431	0,086721049
SMARCC2	-0,167405806	7,370935388	0,063298294	0,086777456
RNF8	-0,127286651	4,814806523	0,063312802	0,086779899
COMT	-0,215674697	7,548233583	0,063513713	0,087037783
SURF1	0,213960013	6,021162032	0,063852805	0,087484886
NCDN	0,18687352	5,490869441	0,063939295	0,087585789
GPR153	-0,322590814	5,022813277	0,064059382	0,087732662
ATP6AP1	-0,155022696	8,223519474	0,06424727	0,087972316
ATP6V1A	-0,131551997	7,127379335	0,064401919	0,08816637
WDR59	0,158211048	5,417904965	0,064555774	0,088344363
SRGAP1	0,324559452	4,451860038	0,06456229	0,088344363
ZNF543	-0,151967132	3,270807131	0,064570803	0,088344363
WIPF1	-0,233392366	6,732813412	0,0646498	0,088434702
STXBP1	-0,33310776	6,871504152	0,064889554	0,088744859
CIC	-0,18338894	6,649965688	0,064930451	0,088782985
PGGT1B	-0,121203062	4,708861369	0,065026349	0,088896286
ACO2	-0,15401247	7,194533611	0,065151914	0,089050091
C3orf18	-0,224207014	3,265060181	0,065312459	0,089251634
FLII	-0,165030077	7,890709909	0,065383371	0,089330637
EIF3K	0,206250521	7,677933523	0,065726634	0,089781634
CDKN1B	-0,151843878	6,633519578	0,065994297	0,090129203
CKB	-0,407509159	7,189710539	0,066109166	0,090268003
PDS5A	0,127870866	7,636393822	0,066137656	0,090271325

PCBP2	0,147662286	9,671951146	0,066138076	0,090271325
PIGX	0,175809792	5,331536675	0,06691369	0,091311678
TRAFD1	0,153648658	6,383950874	0,067143109	0,091606414
ELP2	0,184473367	6,10051112	0,067189497	0,09164739
MAPK8IP3	0,285141435	5,680220857	0,067200022	0,09164739
ZNF431	-0,218296723	4,08116985	0,067512584	0,092049019
RGS19	-0,197685208	5,006543884	0,067521512	0,092049019
ATP11A	-0,327652151	8,642776693	0,067691119	0,092261791
VEGFC	-0,401522442	3,906693002	0,067706512	0,09226433
LOC100216545	0,28337403	2,271855762	0,067772442	0,092325829
GTF2H1	-0,129843668	5,73520154	0,067778721	0,092325829
PTPN23	-0,154688322	6,49282127	0,067845377	0,092398167
GAA	-0,231154337	7,974412128	0,068196942	0,092858415
CDC16	-0,140845102	6,238190103	0,0682559	0,09292014
ERI1	0,176654558	4,856528513	0,068335733	0,093010253
PGM2	0,216746575	5,28852135	0,0684386	0,093131674
FAM133B	-0,12318412	4,992751125	0,069022325	0,093907269
ZNF271	-0,164773429	5,145547219	0,069137402	0,094045072
RSL24D1	0,311173223	7,327385566	0,0691946	0,094104105
ALKBH8	-0,193081848	3,675755381	0,069617768	0,09466073
AGPAT3	-0,161553432	7,116111503	0,069631994	0,094661197
ESYT2	0,211092089	8,088888674	0,069706359	0,094743405
C19orf6	-0,151469632	7,044086704	0,069930925	0,095029689
ANP32B	0,152181655	7,711924947	0,070152404	0,095311664
MLX	0,174269948	6,722849652	0,070186089	0,095320496
SKIL	-0,212584578	5,50198834	0,070186862	0,095320496
EFNA1	0,312890314	8,041685879	0,070407233	0,095600741
ACTN1	0,256555191	8,225581099	0,070942703	0,096308639
ADCK2	0,181267519	5,619009346	0,071072388	0,096465489
MFGE8	0,240122479	7,147448303	0,071135175	0,096531494
SUMF1	-0,17504502	6,146985671	0,071259415	0,096680849
NUP98	-0,13974422	7,252487927	0,071592292	0,097113157
UQCR11	0,237658908	6,943782964	0,072103894	0,09778768
GCC2	-0,187959627	6,121158672	0,072644264	0,098500942
TMEM205	0,193033837	6,547772132	0,072839098	0,098745489
DCLRE1C	0,146718667	4,501766463	0,072853581	0,098745491
MIA3	0,190142709	6,943761526	0,072879004	0,098760319
TMEM106A	0,198829298	4,015855871	0,073012945	0,098922168
FRRS1	0,280012168	3,346684608	0,073184757	0,099135252
LPP	0,252959149	5,086388362	0,073212192	0,099152719
RFX7	-0,168830875	4,876806668	0,073450595	0,099455839
MYSM1	-0,215048919	2,907406065	0,073531695	0,099545887
ZNF678	0,17814094	2,479973607	0,073919655	0,100051236
MFAP3	-0,128126609	5,749149563	0,074092431	0,100265189
FMR1	-0,151606468	6,203136769	0,074118088	0,100280008
ASB3	0,185228465	4,742294306	0,074295826	0,100500543
C21orf91	-0,234609945	4,959913939	0,074397336	0,100617896
RBM3	0,204304476	8,688560054	0,074430469	0,100642747
CALM2	0,254275143	10,05202424	0,074591697	0,100840758
TNRC6A	-0,17865314	5,978421485	0,074849253	0,101168891

KIAA0528	-0,195861327	5,669148739	0,075030965	0,101394401
ZNF177	-0,24860655	1,827372441	0,075074598	0,101433264
CD248	0,320430658	5,580686432	0,07518012	0,10153591
CHIC2	-0,185854833	4,371425684	0,07518035	0,10153591
STARD3	0,159628201	6,212283019	0,075197061	0,101538369
COQ2	0,170367237	4,283218344	0,075275065	0,101623573
ADD3	-0,287713758	6,853177825	0,075300598	0,101637922
TMEM135	0,193135554	5,086096295	0,075317496	0,101640611
MAP3K5	-0,230700097	5,765868081	0,075441356	0,101787615
SAFB	-0,167399652	6,861946247	0,075546167	0,101908865
LUC7L2	-0,103614694	6,820546969	0,075593779	0,101952923
CHD4	0,189168597	8,428130332	0,075953279	0,102417522
DGCR2	-0,16332207	7,720358775	0,076073314	0,1025591
SCAMP2	-0,1672589	7,662182219	0,076366963	0,102934636
KLHDC3	0,156742194	6,998532133	0,07691698	0,103655511
NOP10	0,237903705	6,603829582	0,077576709	0,104523924
CISH	-0,292109047	6,049815612	0,077834384	0,104850388
KLHDC2	0,207996688	6,252688862	0,077931712	0,104960764
SKIV2L2	0,132621141	6,504965495	0,077968076	0,104989003
SLC25A43	0,195228499	5,371079757	0,07846428	0,105636314
DOLK	0,144058537	4,925884595	0,078577693	0,105768118
LMF2	0,15710993	6,700079349	0,078898438	0,106178892
UROD	0,194716374	6,425222903	0,079122869	0,106459913
EPHB4	0,238946157	7,300148879	0,07923968	0,10659605
WLS	-0,326892887	7,065529808	0,079260714	0,106603315
ATG9A	0,134591012	6,400959796	0,079286604	0,106617108
NGDN	0,156603923	5,276852258	0,079392805	0,106738868
CDIPT	-0,130449736	7,025037211	0,079507279	0,1068717
UBTD2	0,162247806	5,631727895	0,079785668	0,107224767
PABPN1	0,160328658	7,242605797	0,079869286	0,107315993
UCK1	-0,131186208	5,284149677	0,080111118	0,107619723
HAGH	-0,18726403	5,870183569	0,08014153	0,107639373
MIER1	-0,160022285	6,277327817	0,080174613	0,1076626
SERGEF	-0,159670423	4,348357874	0,080552525	0,108148782
MALT1	-0,19387755	5,110341196	0,080697877	0,108322603
AKIRIN1	-0,141087468	6,638494205	0,080794498	0,108430955
C1orf123	-0,155647042	5,414643246	0,080885185	0,108531302
CFDP1	-0,180704043	5,914574031	0,081346156	0,109128357
EPHA4	0,378667825	4,952247946	0,081471365	0,109274831
ZXDB	-0,167451907	4,536899559	0,081637958	0,109476743
COL4A1	0,329973508	9,182999093	0,082045322	0,110001386
SMARCD1	0,157620427	6,285079444	0,082086859	0,110035442
CASD1	-0,17928819	5,702042278	0,08214415	0,110090598
UBR5	0,192156569	7,070873416	0,082299415	0,110277011
RNF31	0,134516428	6,788590358	0,082471852	0,110486358
IGF1R	-0,283314556	6,634509796	0,082552263	0,11057236
NARS	0,149187792	7,514607691	0,082671658	0,110710534
MT1E	-0,423969094	6,313777744	0,08272789	0,110764084
BFAR	0,192402243	7,066290815	0,082784938	0,110818706
CCND1	-0,303068648	8,770052699	0,082868839	0,110909247

FAS	-0,267338753	5,591720795	0,082922955	0,110959896
SUN1	0,160139075	7,359912899	0,082984075	0,111019895
CDC42EP4	-0,225581793	6,768862697	0,0833851	0,111534523
ZNF192	-0,275854919	4,435897336	0,083645013	0,111860236
ETNK1	0,216317013	6,882088604	0,083996207	0,11230787
WIPF2	-0,159756401	6,650860042	0,084363836	0,112777298
PCK2	-0,174694108	5,912063573	0,084469721	0,112896713
ZFP36L1	0,248871611	9,333272516	0,084564633	0,113001417
CORO7	-0,13597619	6,005209046	0,084785263	0,113274042
ADRBK1	0,153098689	7,372751871	0,085480716	0,114174955
THOC7	0,177836208	5,876277377	0,085493081	0,114174955
NMT1	0,124268442	7,347704014	0,085751497	0,114497642
EIF3F	0,133210475	7,879103454	0,085790799	0,114527694
CORO1A	0,263093017	6,772318062	0,086094911	0,114911176
ARSB	-0,169796918	5,230635809	0,086223207	0,115059892
C3orf37	0,142370539	6,059821806	0,086278503	0,115111155
MED4	-0,120297302	5,863402426	0,086465459	0,115338021
ATP5D	0,19516773	5,824828808	0,086504375	0,115367364
PICK1	-0,161998488	4,881493952	0,086565179	0,115425881
PCNP	-0,143222479	7,591751353	0,086583703	0,11542801
ARRDC1	0,189718553	6,111968297	0,08668594	0,115541717
FAM195B	0,19787708	6,539339429	0,087101764	0,116073271
DTX3L	0,163040083	7,421491059	0,087598071	0,11671185
ARHGAP5	-0,176648613	7,122879046	0,087749499	0,116890766
KIAA0368	-0,151235373	7,211697806	0,08818788	0,117451788
ELOVL1	0,172425548	7,24782144	0,088318374	0,117602615
MCM3AP	-0,130175555	6,849350343	0,088832732	0,118264428
CMPK1	0,167835089	8,187211679	0,08887398	0,118296246
NDFIP1	-0,143475511	7,832776033	0,090043392	0,119829408
EIF4EBP3	-0,271156419	3,18088434	0,090463489	0,120364981
BRD2	-0,137068426	8,32787308	0,090489447	0,120376031
ZFP112	-0,212751592	2,440029605	0,090553663	0,120437962
TMEM185A	0,129005339	4,700079106	0,090586891	0,12045866
ANXA7	0,125593277	7,789144691	0,091030934	0,121025529
ARHGAP27	-0,205387594	6,017393422	0,09155048	0,121692539
B3GALT6	0,185379909	5,058582227	0,091569601	0,121694232
SACS	0,299553527	4,353230494	0,092404367	0,122779692
B3GALT4	-0,189745492	4,461065789	0,092639837	0,123068585
LSMD1	0,242658406	5,537182847	0,092896098	0,123384981
MSRB2	0,185405823	5,152212542	0,093183433	0,123719897
ANXA11	-0,163371613	8,70131501	0,093184542	0,123719897
AP3D1	0,163928287	7,723909197	0,093390059	0,123968623
SMAP2	-0,167339185	6,615763642	0,093823316	0,1245195
CCDC88C	-0,248704747	6,095808827	0,093945494	0,124657389
BMP2K	-0,179589542	4,55506234	0,09400831	0,124716472
CTBS	0,183142122	5,610786763	0,094363666	0,125163555
AP2M1	0,160213993	8,691311614	0,094654904	0,125525436
TMED7	0,144340152	7,4950897	0,09481517	0,125713522
EPB41L1	-0,208156105	6,653953863	0,095271048	0,126293405
WAC	-0,104444415	7,514780799	0,095676715	0,126806515

SQLE	-0,236978306	6,540732344	0,096214713	0,127494778
INTS3	-0,171949575	6,973718881	0,096236823	0,1274993
MTFMT	-0,111618567	4,34883206	0,096339683	0,12761078
MAP3K2	0,150064352	5,398099168	0,096386649	0,127648196
ANAPC4	-0,131828891	5,045567654	0,096548125	0,127837216
CTBP1	0,122252974	7,44752498	0,096609062	0,127893068
ITGB1BP1	0,168064166	6,032411417	0,097331656	0,128824643
NEURL4	-0,131631363	5,220449477	0,097391768	0,12887919
RNF213	0,239287056	8,594225684	0,097482519	0,128974252
CDCA7L	0,277063571	5,66919667	0,097571848	0,129067397
PRKAB1	-0,193496347	6,555572043	0,097613599	0,129097582
EBAG9	-0,161817217	5,525189198	0,097779345	0,129291711
HSPG2	-0,272036957	9,013749125	0,098318994	0,129980074
RBM19	0,152802682	5,220002783	0,098443762	0,130119793
IGJ	0,488021317	10,08906568	0,098599642	0,130300573
KIF13B	-0,292238716	6,595848303	0,098883322	0,130642164
HMGN4	0,185426566	6,891186507	0,098896444	0,130642164
GOLGA2	0,17837599	6,959882221	0,099210361	0,131031465
TIGD1	0,174361151	3,764418347	0,09966814	0,131610582
IMPDH1	0,225978871	6,549110186	0,099832547	0,131797863
WDR41	-0,145900756	5,243703165	0,099848623	0,131797863
ANKLE2	0,152150551	6,653010244	0,099940282	0,13189332
NUCKS1	0,157615324	8,922114503	0,100145999	0,132128473
BST2	0,275838768	7,69121958	0,100157219	0,132128473
SOS1	-0,139206925	6,235682883	0,100391104	0,1324114
ALG12	0,153887008	5,442211296	0,100860169	0,13300435
MAP4K4	0,233370615	7,561688791	0,100954544	0,133088927
GMIP	-0,15506791	5,413130384	0,10096334	0,133088927
RPAIN	0,182228863	5,536746116	0,101426676	0,13367385
UNC5B	-0,234935882	6,041862097	0,101448387	0,133676628
BSCL2	0,18364878	6,82400156	0,101527853	0,133753744
WDR44	-0,139210364	4,923805217	0,101546141	0,133753744
G3BP2	0,13779803	7,841354128	0,102691323	0,135236023
PLEKHA3	0,11285898	5,014223928	0,102770821	0,135314584
PARN	0,146223908	6,328780119	0,102796289	0,135316792
CBY1	0,188600145	5,493783199	0,102812186	0,135316792
SUCLG2	0,165479263	6,886668035	0,103160031	0,135748408
API5	0,112100716	7,294584572	0,103415735	0,136058634
ZBTB33	0,142633529	5,710655419	0,103561551	0,136224193
ZNF542	-0,241650056	4,428012997	0,104189362	0,13702358
COG4	0,111496459	6,429151301	0,104734099	0,137713425
PRR13	0,139036618	7,714600715	0,105272852	0,138395138
HBP1	-0,148453101	7,053148326	0,105460672	0,138615328
UBN2	-0,168444469	5,25808344	0,105983039	0,13927507
EHHADH	0,217144723	4,58580637	0,106404402	0,139801853
VASN	0,273469485	5,755559134	0,106516837	0,139922618
C1S	0,255365962	8,969652729	0,106644642	0,140063524
VPS37C	0,128522034	5,749683502	0,106703997	0,140114492
MT2A	-0,415984997	7,897439509	0,106947087	0,140406659
KPNA1	0,122300645	6,633408443	0,107293082	0,140833788

KPTN	0,193734584	3,27602229	0,107821604	0,141500292
CC2D1B	-0,124768387	6,189953945	0,107945273	0,141635331
SMG1	-0,159274776	6,967914376	0,108012532	0,141696318
CCDC12	-0,141592617	5,518672815	0,108309615	0,142058717
SMARCA4	0,238188189	7,646198629	0,108662137	0,142493676
LZTR1	-0,132373498	5,791059357	0,108787696	0,142630897
EXOC6B	-0,177058223	3,66827223	0,108828499	0,142656966
MREG	0,220770098	5,677489837	0,109248734	0,143180303
IKBKB	0,215977682	6,401691346	0,109410771	0,143365112
DAG1	-0,16442967	7,817051909	0,110210449	0,144385216
C17orf49	-0,126585198	7,325557591	0,110360036	0,144553417
DNAJC25	0,144111796	3,823991419	0,110590809	0,144827872
SLC31A1	-0,157739029	7,006864843	0,110819842	0,145099943
PML	0,201811614	7,211764898	0,111148989	0,145495301
NAPG	0,128137911	5,828842313	0,11116447	0,145495301
NRIP1	0,222302267	6,553676439	0,111446922	0,145833218
ATP6V1G1	0,178731478	7,493619642	0,111465425	0,145833218
SCP2	-0,149207498	7,759229136	0,11171276	0,146109185
CEP135	-0,129078287	4,048108355	0,11171921	0,146109185
UBR4	-0,162354248	7,709684728	0,111913175	0,146334792
RABGGTB	0,159433722	5,858333265	0,11201457	0,146429688
PIK3CD	-0,194346263	5,450439045	0,112028697	0,146429688
FIZ1	-0,157262405	4,450385946	0,112376167	0,146855708
PIP4K2A	-0,176627362	6,131316925	0,112541378	0,147043429
HNRNPA3	0,131350634	8,937759918	0,112780385	0,14732748
PRPF6	0,154051575	7,566912192	0,112867156	0,147412591
SEPT7	-0,113975156	7,925618415	0,113093477	0,147679896
KIAA1217	0,21413909	7,040542549	0,113123942	0,147691396
PATL1	0,157068584	6,303367173	0,113154199	0,147702619
IMPAD1	0,13288337	7,446612888	0,113176942	0,147704033
OSBPL2	0,133392467	6,416348808	0,11325379	0,147776042
IQCB1	0,184883711	4,890844308	0,113429139	0,147962622
FAM165B	-0,19001127	4,347509301	0,11344018	0,147962622
PANK3	0,17673646	4,862877285	0,113832625	0,148446103
PSMB10	0,233072161	6,756878674	0,113882281	0,148482462
DCUN1D4	0,132567328	5,412627567	0,113916964	0,148499289
SLC25A38	-0,13117617	5,609325527	0,114049558	0,148643719
ARHGEF10L	-0,177125273	5,667093968	0,114117348	0,148692172
MAT2B	-0,156551212	7,079066732	0,114130346	0,148692172
LPIN1	-0,207158367	5,712851398	0,114549992	0,14921039
FOXK1	-0,191461326	6,416099399	0,114860614	0,149586426
DHX36	0,12894489	5,98965535	0,11488368	0,149587897
ZNF187	-0,153883384	4,152004324	0,115079527	0,149814298
ARF3	0,14514367	8,599190459	0,115272395	0,150036736
NCLN	0,13418235	6,780091216	0,11534662	0,150104694
EIF3L	-0,143223162	8,701322236	0,115459037	0,150222319
PIM1	-0,205346407	6,400497853	0,115551947	0,150314522
SSPN	-0,247953711	4,919479788	0,116100359	0,150999113
PPM1A	-0,114744321	6,583743502	0,116334722	0,15127507
MED1	0,183505284	6,433572778	0,116617365	0,151613691

NUFIP2	0,136854063	7,313044038	0,116769256	0,151782226
PCDHB9	0,246775153	2,868686914	0,117076648	0,152152785
BMI1	0,173945151	6,649614551	0,1173553	0,15248586
FASTKD5	-0,154588562	4,890088542	0,117530163	0,152683975
MFSD7	-0,211048489	4,294022946	0,117604978	0,152752066
VAC14	0,134815696	6,340000504	0,117647708	0,152778466
NAT14	0,216521029	4,365995437	0,117920484	0,153103538
SLC35E1	-0,120888129	7,087475656	0,118218253	0,15346093
PIGG	0,120336227	5,514911614	0,11841707	0,153689761
ALKBH3	-0,133426926	4,428145758	0,118451571	0,153705283
RHOF	0,289139797	5,376782378	0,118962159	0,154338464
XRN2	0,14222869	7,210861471	0,119098663	0,154486167
CMTM6	-0,140574027	7,797806888	0,119182439	0,154565434
SLC40A1	-0,323741787	8,671005309	0,119992999	0,155587043
CAB39	-0,127859579	7,44501803	0,120207009	0,155834904
NUAK1	-0,263007995	5,373291973	0,120457768	0,156130302
ADIPOR1	0,125693153	8,023625837	0,12064425	0,156342291
SMEK1	-0,125897443	6,708323485	0,120807089	0,156523568
GTF2A1	0,204390998	3,618185797	0,120999828	0,156743508
IGFBP4	-0,200019718	9,517991287	0,121040911	0,156766946
HSBP1	0,217705574	7,676400184	0,121268473	0,157031849
DNAJC13	-0,142857151	6,271030737	0,121654822	0,157502227
ZNF81	0,17126513	2,801321492	0,12193039	0,15782903
MALAT1	-0,307393792	7,839069788	0,122057842	0,157964021
RBPMS2	0,349243957	2,855530859	0,123010797	0,159167102
POLG	-0,118828147	5,969386686	0,123076368	0,159213176
CYTH4	-0,23749157	5,123009733	0,123093102	0,159213176
RICTOR	-0,144430586	6,054261585	0,123385737	0,159561414
TRIP12	0,136860654	7,683544604	0,123534917	0,159724042
PRKAB2	0,180309423	5,041412321	0,123713119	0,159924125
MUT	0,122837828	5,867748661	0,123762616	0,159957787
CSNK1D	0,113306819	7,96812148	0,124130538	0,160402906
VAMP8	0,24636313	8,402189318	0,124305308	0,160598313
EXOC5	-0,129839304	6,524904584	0,124537805	0,160868212
BPTF	-0,179698575	6,72637413	0,124635337	0,160963705
NSUN3	0,141267882	3,256207357	0,125603506	0,162183357
SEMA3F	-0,236634294	5,674044937	0,126625058	0,163471464
ISY1	0,104805725	5,370723488	0,126683819	0,163516366
ABCA11P	0,154325431	3,054853274	0,127539434	0,164589591
HNRNPM	-0,133262772	7,961556042	0,127582744	0,16460572
KBTBD7	-0,200044667	3,185216829	0,127620132	0,16460572
CLOCK	0,181014299	4,585653057	0,127624349	0,16460572
BAHCC1	-0,218882738	5,237954468	0,12782869	0,164838093
PRSS23	0,238321847	7,692318903	0,128617829	0,165824348
PLEKHA7	0,215293465	5,279974595	0,128913465	0,166171233
GEM	0,343492617	5,988850971	0,12893562	0,166171233
TNFAIP2	0,329929855	7,368026843	0,129133805	0,166395203
ZFP161	-0,096436202	4,555556376	0,129341034	0,16663074
SF3B5	0,168698786	6,609623929	0,129758792	0,167137364
CETN3	0,169806899	4,53903973	0,130001248	0,167418038

CHMP7	0,127434761	6,119874134	0,130088801	0,167499157
EPHA2	-0,30730984	6,734100349	0,130740701	0,168306747
CCDC124	0,147027864	5,965404838	0,130908173	0,168490531
DDX31	0,114591841	4,174551142	0,130957454	0,168522151
MPDU1	0,172425127	6,608642203	0,131017125	0,168567127
RRAS2	-0,17613285	5,746619469	0,131438849	0,169077818
DNAJC14	0,101357979	6,61910613	0,131483719	0,169103636
ZNF397	-0,198743206	4,312730144	0,131923383	0,169637101
APAF1	-0,1221478	5,339805002	0,132022783	0,16971069
TSG101	0,129173158	6,737086756	0,132030387	0,16971069
PDHA1	0,134751865	6,239581068	0,132073043	0,169733525
ZNF776	-0,145748719	4,483032644	0,13237792	0,17009328
OSBPL10	0,169660758	5,177352918	0,132874894	0,17069968
IRS2	-0,285223291	6,342806718	0,133135267	0,171001956
RPL26	0,208865344	9,766975178	0,133271788	0,17114507
ITGA5	-0,326283961	7,789027255	0,133485635	0,171387413
SEPT11	-0,197684831	7,375575729	0,133607134	0,171511116
XIAP	0,109390644	7,080047249	0,133741612	0,171651431
NUDC	0,144732394	7,220570296	0,134373434	0,172429892
NDUFA4L2	0,447401397	5,605775047	0,134529337	0,17259747
ZNF652	-0,157273049	6,195318459	0,135430513	0,173720968
SRCAP	0,103720392	7,984278007	0,136082394	0,174524326
RYK	0,125350241	6,468217048	0,13619729	0,174638835
MCPH1	-0,120450942	4,839795912	0,13662395	0,175152982
VPS28	0,159553761	7,079366572	0,136955738	0,175545335
ZFP1	0,138612217	3,819151495	0,137320978	0,175980408
SEMA3C	0,288019038	6,448365561	0,137417198	0,176070626
CCDC106	-0,15483868	3,946109576	0,138106725	0,176920864
TAF13	0,180378646	4,12949395	0,138410631	0,17724505
KIAA0947	0,160431609	6,157124679	0,138411774	0,17724505
ADPGK	-0,132550294	6,619097445	0,138637089	0,177500246
RP9P	-0,138969016	3,679052525	0,138927418	0,17783857
MFSD10	0,173233187	6,82066249	0,139406042	0,178417755
CTR9	-0,124201281	6,393172587	0,139541686	0,178557845
DOCK10	-0,225906382	5,101088537	0,140704976	0,180012613
GLI2	0,283664876	2,976015849	0,140753432	0,180040827
ZNF616	0,135315682	3,574391988	0,140829206	0,180103968
GMPR2	0,132166103	6,498688533	0,141211332	0,180558798
ACADVL	-0,140698346	8,099623951	0,141489919	0,180881094
EID2	-0,147093627	4,169780186	0,141554816	0,180930138
SNAPC3	0,130315834	5,0868245	0,141923541	0,181367434
PIM3	0,191504973	7,298590736	0,142040064	0,181482331
RPA1	0,102341699	6,823493302	0,142600819	0,182164666
ACP2	0,132023262	6,369902967	0,142931574	0,182552988
TBK1	0,124395671	5,530129303	0,142981124	0,182582076
IDH3A	-0,127957071	6,055284131	0,143014512	0,182590519
CNPY4	-0,141113972	3,861314801	0,143315685	0,182940782
ATAD2B	0,147903518	4,330624046	0,143452618	0,183067823
SNRNP70	0,135405834	7,193425244	0,143468903	0,183067823
PPT2	-0,187483485	4,198950438	0,143689788	0,183315373

CUL5	-0,11265261	5,909908147	0,143983271	0,183655429
PPT1	-0,160986406	8,533139355	0,144103459	0,183774357
SLC39A9	0,106607404	7,096099422	0,144207705	0,183872913
RNF135	0,13672495	5,310508138	0,144400641	0,184084496
STX8	-0,147053559	5,04913218	0,144517035	0,184198442
PKNOX1	-0,085100554	4,891116932	0,144707044	0,184406155
ALDH9A1	-0,13471637	7,0897065	0,145025007	0,184776816
DCLRE1B	0,13262051	4,128273352	0,145565551	0,185430878
PDGFRA	-0,24502686	6,396484722	0,145617613	0,185462552
MDC1	0,180561885	5,892962904	0,146029282	0,185952133
DDRGK1	-0,144040679	6,303751283	0,146242116	0,186188385
CHFR	0,129337942	5,192366497	0,146447005	0,186414435
PHACTR4	-0,113167011	6,457566955	0,146614837	0,186593238
SKP1	0,151693623	8,539572933	0,146800742	0,186794973
TXNDC15	-0,140778892	6,529767139	0,147004769	0,186998625
SLC20A2	0,21265299	6,354004605	0,147015637	0,186998625
GGA1	-0,147692431	6,217174027	0,147099683	0,187070634
ZNF551	0,150287565	3,07926544	0,147370279	0,187379813
GLS	0,320023058	8,1725753	0,1474106	0,18739148
TMEM160	-0,168164075	2,868764048	0,147434417	0,18739148
SHMT1	0,183656042	5,638420118	0,147473596	0,187406346
CLEC2B	-0,289154196	4,973696237	0,14798493	0,188014739
EIF5B	0,159143163	7,870270479	0,148007496	0,188014739
SNX18	-0,173397495	6,153589948	0,148080367	0,188072271
DNAJC1	-0,146916588	5,797765937	0,148976033	0,189174594
ACOT9	-0,113240934	5,66028662	0,149043678	0,189225254
APP	-0,151247867	10,14727154	0,149220129	0,18941401
ILF3	0,121860402	8,16967933	0,149357221	0,189552743
RRN3P2	-0,115036421	2,365330128	0,149462992	0,189651683
IRF5	-0,197134027	4,919632858	0,14953747	0,189710886
ZNF614	0,224935067	4,248799566	0,14979508	0,190002354
SMCHD1	-0,145205458	6,247627567	0,150154608	0,190422962
AGAP5	-0,189699192	2,60534934	0,150394329	0,190691507
DPCD	0,175916539	4,517155849	0,150635609	0,19096193
RNF144A	0,185040801	5,3150052	0,151169136	0,191588346
BLVRB	0,260270742	7,11633927	0,151185935	0,191588346
ROBO1	-0,230870909	5,672548324	0,151237493	0,191618072
LAMP2	0,156593069	8,562178618	0,151422294	0,191816575
CLINT1	0,151658393	7,435978091	0,15187656	0,19235629
TP53RK	0,143855109	5,011415268	0,15215368	0,192671485
LOC283922	-0,18873176	2,280550458	0,152484652	0,193054743
C22orf32	-0,171618834	5,857606598	0,152551216	0,193103164
C20orf112	0,176747898	5,595230547	0,153290049	0,194002384
MBD6	0,153875028	6,397981218	0,153573113	0,19432456
PLCB3	0,149252184	5,818894139	0,153664707	0,194404386
C9orf78	-0,104731341	6,374774717	0,15374728	0,194472769
NPEPL1	-0,173317377	5,555766581	0,154109248	0,194894466
ZNF613	0,185890822	3,073403619	0,154146396	0,194905298
RPL3	0,147994651	11,44294193	0,15424569	0,19499469
UBA3	0,136551056	6,329613547	0,154418237	0,195176637

PRKAA1	0,138335442	7,029648685	0,15459513	0,195364008
TXN2	0,152911407	6,586666654	0,154882196	0,195674493
EXOC4	0,104522783	6,707059091	0,154898213	0,195674493
VPS16	0,100134794	5,705908467	0,155324566	0,196176739
EFTUD1	-0,115238625	4,951452564	0,155586998	0,196471804
DOCK7	0,151631035	5,451013729	0,155754463	0,196646859
SMC1A	0,163294263	7,23121867	0,155967317	0,196879143
ATP5O	0,182712203	7,281886686	0,156364895	0,197344478
POLR2C	0,159420885	7,141958337	0,156534821	0,197522381
FOXJ3	-0,117163718	6,473279534	0,156686823	0,197677603
RNF10	-0,097430616	7,830421594	0,156733311	0,197699676
C19orf43	-0,123107921	6,826507828	0,157071211	0,198089253
PLS3	0,235677032	9,274854037	0,157167501	0,198174037
DDX59	0,121506161	4,685636001	0,157458391	0,198504117
DECR1	-0,133939412	6,229261336	0,157998233	0,199147866
ZNF713	-0,168932657	2,628199699	0,158153806	0,199270199
SPTLC2	-0,15205367	6,635224977	0,158167739	0,199270199
CEP70	0,154061806	5,194053387	0,158182958	0,199270199
ATG2A	0,141596418	5,944698275	0,159220669	0,200516074
NDUFB1	0,195027018	5,80193793	0,159230758	0,200516074
NUDT15	0,145567563	5,028946791	0,15933048	0,200604605
NPC2	-0,265199041	9,981447517	0,159534667	0,200824607
MIPEP	-0,136947118	4,620534098	0,159565072	0,200825807
CCDC6	0,142790116	7,371107832	0,160005026	0,201342364
FOXN2	-0,138373459	5,355326529	0,160332249	0,201716902
CTSB	0,209974323	11,21130129	0,160555284	0,201960243
SNHG8	0,215357784	5,549637785	0,160716746	0,202126059
GTF2H5	0,149185101	5,480376191	0,160900698	0,202320092
ATF4	-0,141692568	8,602440067	0,161001058	0,202408963
MYL12B	-0,168743194	9,428524253	0,16130387	0,202752275
PTDSS2	0,135205594	5,095629344	0,161452352	0,202901509
GNA13	-0,124414028	7,048532141	0,161810436	0,203314053
EFHD2	0,192626246	7,430719387	0,162201825	0,203768284
ZNF276	-0,140912106	4,885956132	0,162562283	0,204183497
ZFAND1	0,17997206	5,712115992	0,163645393	0,205506065
CHRAC1	0,116312278	5,888386493	0,163907716	0,205797591
C12orf29	0,163168793	4,724755039	0,165623573	0,207913686
AMMECR1L	0,096798267	5,552222778	0,165839635	0,20812181
CGN	-0,259652727	7,78506569	0,165850406	0,20812181
PTPRF	0,151506226	9,18224059	0,165991793	0,208260908
ARFGEF2	0,139408062	6,532209948	0,16682255	0,20926471
DYRK1B	-0,163250517	5,006029136	0,166997656	0,209445837
PICALM	-0,097873649	8,11920457	0,167223748	0,209690831
RPS20	0,168705451	10,17765763	0,167327542	0,209776966
RNF170	0,119179038	5,444340475	0,167381383	0,209776966
DHX35	0,122782186	4,115101375	0,16738473	0,209776966
SCAMP1	0,110923705	6,500806035	0,168165931	0,210717289
BRD3	-0,138012525	5,85113555	0,169006691	0,211695221
QRSL1	0,108922747	4,735355184	0,169008473	0,211695221
NDUFS4	0,157336509	5,616695585	0,169088601	0,211756688

STT3B	0,12641861	7,15555941	0,169647472	0,212417575
P4HA2	0,190276292	6,899998769	0,169764116	0,212524601
LAMB1	-0,220988976	7,676329422	0,169855179	0,212599572
CHMP2A	0,127729642	7,150934434	0,170224362	0,213022559
GBP2	0,223469168	7,278202649	0,170300879	0,21307921
CYBA	0,208372105	7,843028785	0,170770974	0,213628191
MED9	0,116761304	4,560480379	0,170855558	0,2136948
SETD1A	0,131265686	5,735239753	0,171329116	0,214247798
FRG1	-0,101833775	5,406453123	0,171821082	0,214823608
HMGN2	0,148474827	9,279695444	0,171917486	0,214904736
ZNF414	-0,123150818	3,396578521	0,172065309	0,215012475
MFSD11	0,098316373	5,670716289	0,172066737	0,215012475
WBP5	-0,149689869	6,248766807	0,172167954	0,215099538
IFNAR1	-0,103589573	7,140173302	0,172470194	0,215437672
SERPINE1	-0,339673773	7,678801795	0,172690033	0,215672772
FTSJD1	-0,131623756	5,403625515	0,17314973	0,216202019
TAOK1	-0,169302606	4,841798933	0,173177215	0,216202019
UBE2D4	0,138954129	4,158073203	0,17358493	0,21667136
ZNF462	-0,251560722	4,384416523	0,173652152	0,216715597
GPAM	0,238878296	4,954030238	0,173803411	0,216864677
LTBP3	-0,186645402	7,793656981	0,173842692	0,216874006
PREP	-0,113606947	5,711762334	0,173901773	0,216904032
CSAD	-0,19853461	4,158679541	0,173930378	0,216904032
MANEA	0,142402739	5,024957889	0,174296574	0,217320962
ADAP2	0,20119464	4,719125307	0,174660283	0,217734638
NEK4	0,098194482	4,933351034	0,175689867	0,218978103
ARID2	-0,133237983	5,343386179	0,17604125	0,219375966
RNF169	-0,142722597	5,017310377	0,17654569	0,219964382
CSRP2BP	0,107544609	5,204218841	0,176630977	0,220030441
TCIRG1	-0,144074234	7,088812381	0,17680739	0,220209971
RASA2	-0,159544724	3,298230734	0,176892495	0,220216191
NUBP1	0,110205114	5,195454843	0,176911215	0,220216191
RIOK2	-0,091191878	4,944332404	0,176929807	0,220216191
MYO1D	0,196819258	7,760581065	0,176941562	0,220216191
SLC7A5P2	-0,131456775	1,737748854	0,177232989	0,220538641
BRD7	-0,100169066	6,423951987	0,17816099	0,221652945
CWC22	-0,073635087	5,346640163	0,178229079	0,221697207
OS9	0,152601565	9,17597881	0,178430696	0,221907517
BAZ1B	0,134997528	7,131325789	0,178547343	0,222012095
RARRES3	0,332770863	7,254032603	0,178831194	0,222324506
SSU72	0,110159898	7,548640214	0,17891472	0,222387801
HUWE1	0,169729465	8,515144419	0,179537638	0,223121405
LSM3	0,13292404	5,643349311	0,179768765	0,223357214
ECSIT	-0,12258003	5,195658696	0,179792894	0,223357214
TRIM23	-0,12424209	4,420765591	0,179898566	0,223447782
MAPK14	0,104519789	6,805992935	0,18009533	0,22365144
DPY19L3	-0,149818752	4,616800186	0,180827822	0,224520197
ECH1	0,177858264	7,727522527	0,180940025	0,224618611
TNPO2	-0,11821349	6,424588888	0,180992321	0,224642635
POGK	-0,10622344	6,889050658	0,181299233	0,224982615

HIBCH	0,134733328	4,993351037	0,181727702	0,22547329
ZNF765	-0,09519489	4,183925247	0,181799368	0,225521173
HSF2	-0,118018433	4,313513008	0,182424728	0,22625577
MPZL3	0,216574344	2,885209494	0,182619228	0,226455813
RUFY1	-0,1123792	6,207495773	0,182927483	0,226763347
CHMP5	0,149205561	7,321959837	0,18293374	0,226763347
ZNF417	-0,125992844	4,105928538	0,183962909	0,227997651
DNAJA1	-0,117552975	7,892220293	0,18404592	0,228059082
IPO13	0,0978171	5,657113047	0,18555316	0,229884992
SELK	0,169901413	5,969237895	0,185824197	0,230178964
OPN3	0,262291772	6,008048534	0,186121035	0,230504784
RAB18	0,110892402	7,018414705	0,186426323	0,230840947
CCNB1IP1	0,203709343	5,662165238	0,186688333	0,23112341
ZNF574	0,130361733	4,843396683	0,18726212	0,231791685
NUP50	0,111278892	6,382914901	0,187333678	0,231838176
ERMP1	-0,173527962	6,044548047	0,187542751	0,232054803
PRCP	-0,143429979	7,266049486	0,187733408	0,232248568
ATP8B2	-0,170979778	5,84396887	0,187808805	0,232299699
MYC	-0,251800134	6,75250635	0,188055812	0,232563036
TMEM50B	-0,17686483	6,690201769	0,188694748	0,233310877
SPARC	0,207569159	10,8514462	0,188791727	0,233388467
OGG1	0,128498254	4,301573127	0,18919891	0,233849296
PTPRK	-0,160233098	7,016233162	0,189264899	0,233849296
ALG13	0,132035897	5,143999066	0,189298988	0,233849296
WEE1	0,19741362	5,918999279	0,189306223	0,233849296
MAK16	0,118654283	4,830365105	0,189335967	0,233849296
COL6A2	0,240163355	9,257422716	0,190065087	0,234707322
HS6ST1	0,173947146	6,3813442	0,190599527	0,235324674
SNX11	0,130419694	5,451102602	0,191104514	0,235905446
PARP4	-0,135452982	7,40708151	0,191539428	0,236399523
C9orf91	-0,142530207	5,176743203	0,191981413	0,236902145
ZC3H11A	0,124285271	7,711110747	0,192273362	0,237219478
DDX60L	-0,173251304	5,371552112	0,192385893	0,237267829
GABPB2	0,143403585	2,731583506	0,1923936	0,237267829
DDX6	-0,09066242	7,759375911	0,192416937	0,237267829
ASAP2	-0,226270637	5,646200033	0,192580499	0,237426581
GLE1	0,093284747	5,858031837	0,193152841	0,238089158
M6PR	-0,128460877	7,432660711	0,193363695	0,238305989
ASXL2	-0,169298979	5,411596243	0,193968859	0,239008611
DYM	0,108083318	5,34971996	0,194529449	0,239656063
NF2	-0,12392533	6,186096131	0,195039003	0,240218822
BCR	0,148941003	7,343088511	0,195056697	0,240218822
ZC3H18	0,105061442	5,958388347	0,195216737	0,240351277
C11orf54	-0,18359474	5,823476432	0,195265455	0,240351277
USP48	0,104216856	6,190056126	0,195269992	0,240351277
CLCC1	-0,092663422	5,752940284	0,197150454	0,242622081
ZNF428	0,172014635	4,969475368	0,197371953	0,242850838
ZNF224	-0,143566466	3,221728835	0,197918483	0,243479368
ATP6V0C	-0,109503314	7,395800899	0,198415625	0,244046924
ITGB1	0,132450116	10,12208424	0,199176108	0,244938121

MTOR	0,129233318	6,479265813	0,199426934	0,245202355
TBCCD1	0,107485723	4,512801576	0,199477116	0,24521984
THAP1	0,114588935	4,020421353	0,199820525	0,245597722
DPY19L4	0,107062926	6,055987055	0,199874617	0,245619934
ARIH1	-0,092083933	6,734523031	0,200060514	0,24580408
ERF	-0,12263498	5,767464665	0,200185808	0,245913714
MSL1	0,108641516	6,701738111	0,20073044	0,246538341
UBA1	0,106648422	8,804145299	0,200865717	0,246660062
ADH5	-0,138122337	7,775282477	0,201423894	0,24730096
RELB	0,188830182	5,332023047	0,202056014	0,248016579
ZCCHC11	-0,133569299	5,367251294	0,202079501	0,248016579
TDP2	0,156345495	6,225180988	0,203156633	0,249293698
CMTM8	-0,150017828	4,518622255	0,203289449	0,249411794
ATG10	0,11667912	3,612086158	0,20360619	0,249755461
BLOC1S2	-0,128905493	5,898154144	0,204178032	0,25041187
DZIP1	-0,181615477	4,223116062	0,20459255	0,250875131
CHST11	-0,20376611	4,682701831	0,205003318	0,251333626
C3orf38	0,133876991	5,04204765	0,20520466	0,251524954
SLC41A3	0,11007485	5,931372278	0,205233149	0,251524954
C15orf61	0,157373403	2,92783102	0,206039863	0,252468253
SERP1	-0,114006634	8,490744253	0,206259926	0,252692496
ANKH	0,154818605	6,459390295	0,206572201	0,25302961
LOC253039	-0,148145762	4,812372956	0,206738994	0,253188433
STARD3NL	-0,166731665	6,311279479	0,207644294	0,254251471
MGST3	-0,145815102	6,928427799	0,207751025	0,254336488
CEP97	0,167093613	3,107479401	0,208066585	0,254677086
TP53INP1	-0,180956363	6,729335698	0,208873437	0,255618803
SUSD1	-0,129103109	5,064018106	0,21007274	0,257040376
HELZ	-0,11763671	6,29417163	0,210383489	0,25736536
SMC3	0,12958227	6,492021222	0,210413826	0,25736536
CARHSP1	0,14487813	6,793291674	0,211669615	0,258854933
DNAJB6	0,105755401	7,467527759	0,211890209	0,259078238
TTC1	0,108999831	6,169773374	0,212113792	0,259305118
RAB3GAP1	-0,090678832	6,593491227	0,214136058	0,261730378
SRRM1	-0,089410221	6,916310706	0,214489651	0,262115579
PPP1CB	0,101370544	8,912851534	0,214786938	0,262431846
PHRF1	-0,119730196	6,077668202	0,215073332	0,262734692
SLC16A4	0,328896895	5,758992332	0,215288607	0,262950566
ZNF664	-0,12471034	7,764341591	0,215406584	0,263047546
NAMPT	0,285815267	9,218808296	0,215810031	0,263493035
IDS	-0,155072979	8,424967279	0,21669045	0,264520619
CEBPZ	0,090596641	6,485046876	0,217164225	0,265051521
SS18	-0,121967733	6,804063394	0,217754213	0,265724048
COL4A2	-0,224219354	9,372674528	0,218119209	0,266121826
NDUFAF3	-0,121080588	6,152314511	0,218199844	0,266172583
TMEM127	-0,086923523	7,327647487	0,218881432	0,266956266
CCNK	0,095867487	6,231120285	0,219301156	0,267420348
DNAJA2	0,092224523	6,840059713	0,219607626	0,267746183
ZNF740	-0,105608174	5,466998279	0,219902749	0,26805807
SSBP3	-0,131437024	5,629106285	0,219978076	0,268101966

BRWD1	-0,115018609	6,052213525	0,221217069	0,26956383
EGFR	0,296491643	6,715368678	0,221580735	0,269958734
LGALS9	-0,190463489	7,270133636	0,221814112	0,270194789
C3orf52	-0,169637026	4,286989194	0,221957213	0,270320814
RPL11	-0,122142108	10,02604232	0,222792684	0,271289877
HSDL1	0,132375529	5,411538352	0,222998077	0,271491499
F2R	-0,169848426	6,398772885	0,223644261	0,272229599
PTPN3	-0,143387584	5,808540063	0,224198134	0,272855091
GTF3C4	0,132322042	4,866134899	0,22493793	0,273706592
EXOC2	-0,095385594	5,718140585	0,225011865	0,273747709
ZNF277	0,137564487	5,502562096	0,226051805	0,274963835
SIPA1L3	0,14905235	6,646088622	0,226344375	0,275270607
PLCG1	-0,153041325	6,384170144	0,22656701	0,275492233
LRCH3	-0,139297453	4,297141001	0,22697667	0,275941151
RPL22	0,100299997	9,358844871	0,227300836	0,27628599
KIAA1430	-0,095310845	6,084558371	0,227487419	0,276463502
TRIP11	-0,120679443	5,618734165	0,227984532	0,277018268
FUT10	0,144482812	3,303423481	0,228902793	0,278084473
PANK2	-0,083497069	4,932736023	0,229188548	0,278382028
PIK3C3	-0,112675007	5,19670014	0,229483288	0,27869039
HTATSF1	0,115010652	6,665379348	0,229848464	0,279084166
CCDC7	0,146818358	1,033126095	0,230132676	0,279379512
RBAK	0,133219013	4,753749507	0,230176045	0,279382422
HCFC1	0,132957425	7,120905583	0,230290626	0,279471753
ASB7	-0,077338423	4,688656744	0,230893083	0,280153013
SHISA5	0,099151963	8,030728465	0,231756584	0,281150711
AP3S2	0,104728102	6,607937073	0,232041625	0,281446432
CLDND1	0,141989949	6,643734708	0,232161271	0,281541473
ITFG1	0,103932516	6,598499059	0,232501144	0,281903503
PBX2	-0,126870893	7,057328201	0,23257553	0,281943563
FKBP9	-0,127003009	7,696072721	0,235079607	0,284928518
VPS4A	-0,075132546	6,989608939	0,235274542	0,28511411
ANXA1	-0,263736287	9,345210072	0,235324893	0,285124457
GSPT1	-0,081736927	7,912981339	0,235473125	0,285253374
ZNF789	-0,11562422	2,45378017	0,235599093	0,285355278
TXLNG	-0,13154258	3,729984109	0,235848114	0,28560616
RBM14	0,073738516	5,934582366	0,236143216	0,285912746
ZNF638	-0,100096138	6,856185701	0,236323974	0,286080806
KDM3B	-0,103241699	7,070666088	0,237149046	0,287028638
USP24	-0,103068828	6,503555973	0,237783457	0,287745412
MKKS	0,111040457	5,666763175	0,238020413	0,287981049
IFFO2	-0,164646551	5,372428634	0,238265839	0,288226851
PPTC7	-0,132706836	5,512396308	0,238491718	0,288448923
SOCS7	-0,162976566	2,437304766	0,238624507	0,288558347
CEP57	0,097431657	5,832160981	0,23914749	0,289139492
BOK	-0,179281873	6,499449076	0,239202722	0,289155001
MYO10	-0,1666466	7,134945928	0,239483283	0,28944284
CYB5B	0,150940985	7,06490627	0,23989628	0,289890614
GOLGA7	0,135496115	6,471199725	0,240151134	0,290147162
COMMD8	0,161812347	4,916791786	0,240380238	0,290372514

RPL23AP82	0,097154217	4,274478693	0,240514574	0,290483329
CAPZB	0,088317912	8,408292413	0,241559752	0,291693988
HECTD1	-0,125982635	7,62290172	0,241863394	0,292008938
SELT	0,170143206	7,828562458	0,242240432	0,292412375
PPIG	-0,08417201	6,286197933	0,242799715	0,293035621
SPATA7	-0,11829164	3,593632255	0,243571391	0,293914938
POM121C	0,1166981	6,531574785	0,243740048	0,294066416
SNX27	0,120640109	6,435896528	0,243871485	0,294172945
GPBP1L1	-0,07367996	7,521524871	0,244662973	0,295075489
SFMBT2	-0,170676112	2,966859643	0,244771568	0,295154257
CDC42EP3	0,174174236	6,421130858	0,24509542	0,295492516
CHUK	0,095144669	5,549385425	0,245381187	0,295784747
UBA5	-0,078736004	5,684075242	0,246205047	0,296725383
VASP	-0,119247074	7,600924651	0,246841669	0,297440068
KCTD3	-0,166212574	6,060836469	0,248008841	0,29879369
DCAF10	-0,0888696	5,896382993	0,250608725	0,301872619
C5orf44	0,121627046	5,194799953	0,250999576	0,302290023
TRA2A	-0,086567401	6,553541091	0,251177052	0,302450347
SERPINB1	0,206149358	8,097668379	0,251564986	0,302863991
MMAA	-0,093049087	3,938901813	0,251877194	0,303184652
CHD1	-0,115691381	5,767507499	0,251920258	0,303184652
VTI1A	0,070456419	4,840044107	0,251992914	0,303185428
AKAP10	-0,098633382	4,961463595	0,252009826	0,303185428
MPST	0,157934005	6,549901849	0,252257068	0,303429343
ZCCHC8	0,079683338	5,114515556	0,253448652	0,304808882
POLD3	0,097947588	5,050057627	0,253659506	0,304986307
OSBPL5	-0,168712436	5,372107836	0,253685633	0,304986307
UBXN7	0,158772302	4,418012016	0,256390961	0,308184376
KEAP1	0,11404456	6,57347213	0,257002702	0,308865249
TAF6L	0,087745698	4,076719839	0,259385609	0,311647869
DNAJC3	0,112253495	7,534928391	0,259409489	0,311647869
PDLIM5	0,138192515	7,041805943	0,261223978	0,313772469
ANP32A	-0,080810746	7,35757402	0,261296096	0,313803818
NSD1	-0,125564169	6,621163643	0,261462157	0,313947957
URM1	0,101113641	6,168595436	0,262263902	0,314855202
NARS2	0,126301181	4,991209155	0,262992487	0,315601211
GPBP1	-0,073734053	6,890652852	0,263029286	0,315601211
ST6GALNAC4	-0,156334748	5,857877086	0,263039744	0,315601211
RBX1	0,159803598	6,441128131	0,263070433	0,315601211
ZNF586	-0,112897798	3,470284054	0,263306584	0,315828953
METAP2	-0,080821486	7,032058753	0,26344283	0,315936802
CLN6	0,111417191	5,850611253	0,263890225	0,316417698
ARHGEF11	-0,119023905	6,538207491	0,264609614	0,3172245
TROVE2	0,077188594	6,209529948	0,265329629	0,31803177
SPPL2A	0,11237929	6,115988359	0,266942374	0,31990862
CARKD	-0,102541005	5,808129179	0,268109053	0,32125033
ZNF335	0,099524761	5,068606926	0,268301357	0,321424271
PDE7A	-0,114618834	5,228666521	0,268371841	0,321452236
GSDMD	0,131981946	6,861843072	0,268635301	0,321711295
CDK19	-0,101679634	5,315895302	0,26868281	0,321711691

LPIN3	0,13385892	4,585012965	0,268929524	0,321950565
AP4S1	0,12075984	3,073742329	0,268994839	0,321972232
AP1B1	0,093940002	7,6134112	0,26943214	0,322439059
C5orf42	0,144958976	5,053698958	0,269781155	0,322800087
FAM117B	0,112376674	4,957292127	0,270414425	0,323501046
CEP192	-0,113201717	4,973284415	0,271527512	0,324775671
CENPB	-0,096440687	7,110287209	0,272555354	0,325947905
HBS1L	0,092357515	5,860078495	0,272669879	0,326027688
ACIN1	-0,095962867	7,360128502	0,273704282	0,327177739
CDK18	-0,132734751	5,50861959	0,273727673	0,327177739
PACRGL	0,109484772	3,957541536	0,273852972	0,327253701
QARS	0,084711439	7,796115369	0,273887208	0,327253701
FHOD1	-0,145231404	6,268173489	0,276614552	0,33045456
TNRC18	0,151795182	7,844716543	0,277378592	0,331309269
NDRG3	0,072500965	6,107558583	0,277709095	0,33164594
SH2D4A	0,163270443	5,822811387	0,277791081	0,331685761
NGRN	-0,089986349	7,029920173	0,278213456	0,332131925
SBF1	0,123210067	6,698396269	0,278269194	0,332140317
ARPC4	0,103576836	8,084716201	0,278563733	0,332433689
GNL1	-0,083218007	6,373501896	0,278928694	0,332810983
AMIGO3	0,157443776	2,523978265	0,279713032	0,333688447
MBIP	-0,197613549	7,004901099	0,279890369	0,333841599
MED15	-0,102786196	7,091276009	0,280456868	0,334413793
RAC2	-0,150449954	6,846645537	0,280468174	0,334413793
VAPB	0,096131963	6,11283097	0,280610553	0,334525063
CASP8AP2	-0,105954642	4,539015963	0,280720354	0,334597464
CAMK1D	0,17077349	4,35048208	0,280889339	0,334740371
TP53BP1	0,115354954	5,951630539	0,280969155	0,334776982
KIAA0284	0,143162762	6,529948969	0,281691624	0,335579173
ZFAND6	0,114975933	6,852118947	0,281977301	0,335860823
RAB11A	-0,103948791	7,712658596	0,283126593	0,337170841
SLC43A3	0,171106382	7,43280499	0,283309405	0,337329637
GOPC	-0,079409798	5,787389888	0,283611312	0,337630157
ACTR2	0,071160234	8,911919947	0,284929867	0,339140647
NAB1	-0,094338367	5,895207358	0,28751938	0,342163116
PRPF39	0,102093831	4,774162022	0,287888621	0,342542751
CXCR4	0,169459986	6,949737091	0,288134502	0,342775501
PRR14	0,083610231	5,446710787	0,288373373	0,34296346
USP42	0,109700475	4,616775242	0,28839309	0,34296346
WDR61	0,122736001	5,926239567	0,288616698	0,343169532
ACTA2	-0,171739728	8,288739276	0,289229699	0,343838445
R3HDM2	-0,100428492	6,517931603	0,289432501	0,344019562
ZNF461	0,120293183	3,179402785	0,289511489	0,344053476
FNBP4	-0,107711795	5,999269403	0,290355561	0,34499644
STIM2	-0,129929925	5,594718208	0,290484309	0,345089286
LAMC1	0,148080496	8,455493474	0,291194501	0,345872723
OXR1	0,109355383	6,180614264	0,291556355	0,346242213
LRRCC1	-0,106745663	4,8893824	0,29271449	0,347557044
LRRC58	-0,10231811	6,204604682	0,292844872	0,34765132
ASB8	-0,064495867	5,523295308	0,292933793	0,34769635

CD300A	0,187749477	4,740440826	0,293569278	0,348389995
CYTH2	-0,081008501	6,590553817	0,293849747	0,348662159
EXOC7	0,071730414	7,535209095	0,294086072	0,348829493
GPR183	0,209839096	5,045815497	0,294093086	0,348829493
NID1	0,200135233	7,025694238	0,294413804	0,34914917
ELMOD3	0,102477015	4,287791567	0,294477991	0,349164566
DHRS7B	-0,123911139	5,290336309	0,294946085	0,349658789
STUB1	0,086228706	6,793520051	0,295038201	0,349662077
RAB5C	0,080766427	8,045716412	0,295087938	0,349662077
DGCR14	0,086071862	4,619821423	0,295102692	0,349662077
XRCC1	0,096888357	5,574089262	0,295887586	0,350531176
DENND4B	-0,103979877	6,126520027	0,296760035	0,351503678
GCC1	-0,091623075	5,423454028	0,297194325	0,351956947
NHLRC2	0,121549408	3,412436092	0,298244041	0,353138759
RBM27	0,070709355	5,834533759	0,298635962	0,353541428
CDK5RAP2	-0,124933907	6,334345607	0,30028078	0,355426946
RPS12	0,131230599	10,13937578	0,300689081	0,355848463
IL15RA	-0,136876524	4,913642014	0,302609606	0,358021183
TTC3	0,107014033	7,764954756	0,302630019	0,358021183
SGTA	0,071190382	6,545681792	0,30278151	0,358138268
EYA3	-0,103612497	3,158001421	0,303174002	0,358540326
SHB	-0,124129757	5,803939402	0,303505141	0,3588697
PURB	-0,097873022	6,779359536	0,304062301	0,359466163
RNF103	0,099903053	5,9514071	0,304231093	0,359603367
PANK4	-0,069774274	4,735949486	0,304979255	0,360425223
SMYD2	-0,096643672	5,990307061	0,305327133	0,360773821
BBS9	-0,111035988	4,513991691	0,305848177	0,361326875
HDAC10	0,100936705	5,066186166	0,306005813	0,361450483
SLC46A1	-0,111062473	5,41689489	0,306549997	0,361943194
KDM5A	-0,099629014	6,489284143	0,30655648	0,361943194
RSC1A1	0,106162051	3,659892688	0,30658218	0,361943194
IBTK	0,088599853	6,566977771	0,306804313	0,36214274
MRPL46	0,101786093	4,555111388	0,30760731	0,363008005
DCTN3	-0,098767302	5,84300987	0,307643828	0,363008005
ASL	0,136965777	6,370813617	0,307802989	0,363107386
GNB2	0,084726882	8,220032095	0,30783455	0,363107386
DDHD2	0,190259569	6,097251386	0,30933299	0,364766195
KDM2A	0,098554655	7,652191618	0,309347838	0,364766195
ABL2	-0,103838591	6,029053066	0,309431269	0,364801491
KITLG	-0,166707195	7,137024832	0,310150546	0,36558627
GGNBP2	-0,057244479	6,43314691	0,31024691	0,365636654
MAP2K1	0,086775027	6,678538169	0,3111934	0,366688749
PRDX5	-0,126082147	8,687789022	0,311304063	0,36671945
LILRB4	0,239811532	5,61344023	0,311327012	0,36671945
ARHGEF5	-0,118469337	6,439267393	0,311499305	0,366859026
DDX51	-0,099934645	5,073440519	0,312486387	0,367957982
GATSL3	0,125143104	4,436775182	0,312671262	0,368112109
PTPN4	-0,092674928	4,185368933	0,31326894	0,368702464
PHAX	-0,070837509	4,715148915	0,313280844	0,368702464
ZNF430	-0,093369707	3,374661525	0,313933736	0,3694071

SNTB1	-0,144122507	6,723898108	0,315208793	0,370806994
SCPEP1	-0,160958631	7,943129411	0,315232167	0,370806994
RNF216	0,080708491	6,382375048	0,31542969	0,370975346
DNAJC16	-0,066699352	5,309047006	0,315764356	0,371304904
RAI1	0,124295208	6,138714589	0,316186202	0,371736847
ATP6V0E1	0,124695032	8,273849813	0,316361743	0,371879111
LGALS3	-0,163175899	9,318178775	0,316829367	0,372364608
DOCK1	-0,119732808	6,649866342	0,317223396	0,372713481
CHST3	0,223844096	5,436522124	0,317235525	0,372713481
ZNF558	0,106060724	4,265566526	0,317924399	0,37345848
PHTF1	-0,082323437	4,635922681	0,319671942	0,37542427
CBX5	0,123694559	7,343751149	0,319707983	0,37542427
SLC25A32	0,07965767	5,552131879	0,319830536	0,375503517
ACADM	-0,103054757	6,156271862	0,320087987	0,375741089
ABL1	-0,104753153	6,883008677	0,32022319	0,375818312
C11orf57	0,071027984	5,768766278	0,320263999	0,375818312
BCAS2	0,090841485	5,312724209	0,320950796	0,376526506
MSL3	-0,072477119	5,338526163	0,320977941	0,376526506
EHBP1L1	-0,106698386	6,67259987	0,321501725	0,377076069
SPTY2D1	0,0712159	5,983547675	0,321671335	0,377210118
VPS18	-0,097532032	5,893382946	0,321980183	0,377507371
NSUN4	0,068389879	5,305300491	0,324474614	0,38036658
WDR5B	0,0960557	4,205243657	0,324853399	0,380745158
ZNF14	-0,109889356	3,653284232	0,325409103	0,381330928
EPS15L1	-0,075310922	5,509018796	0,32659616	0,382656222
PTP4A1	0,136558638	8,896041708	0,327488434	0,383587333
RAPH1	0,122210331	7,115956847	0,327503366	0,383587333
ZNF688	-0,111834684	3,959216084	0,327766023	0,383829042
IRF9	0,106203359	6,087555452	0,328306169	0,384395565
RNASEH2C	0,10187527	5,304452081	0,328964402	0,38510013
DDX60	-0,145398671	6,074353379	0,329351131	0,385486674
GTF3C1	0,097400895	7,064290717	0,330611836	0,386882781
CAP2	-0,13889073	3,714630082	0,330657406	0,386882781
ST6GAL1	0,145900439	7,51167874	0,331652107	0,387980051
RAB23	-0,10984693	4,795461513	0,332148766	0,388494414
KLHDC10	0,113426584	5,478899753	0,332809806	0,389200835
TATDN3	0,08159008	4,886403195	0,333896244	0,390404405
CLCN3	0,103090548	6,177818986	0,334177785	0,390666606
ATP9A	0,140853713	7,06962819	0,334597296	0,391089983
ARL5B	0,145830179	4,549917268	0,334705177	0,391149033
LRRC47	0,062645357	6,216143361	0,334806822	0,391200775
SMAD3	-0,110162625	6,676890104	0,335016099	0,391378238
TMEM59	0,116444001	9,025857212	0,335709776	0,392121439
CSNK1G2	0,07520283	6,149671245	0,336667371	0,393172598
TBC1D23	0,079178756	5,900423695	0,336725438	0,393173076
HADH	0,122825315	6,286835022	0,338464213	0,395135673
EIF2C1	-0,093482789	5,834364402	0,339611089	0,396406713
FNDC3A	-0,092412928	6,955587367	0,340501268	0,397377742
CDK10	0,11117704	5,637574334	0,340630183	0,397460167
PLD3	-0,125072339	9,345409353	0,340857521	0,397657389

SSBP4	-0,098686262	5,496892839	0,341097372	0,397869138
REPS1	-0,090192936	5,456725261	0,342699508	0,399669565
TGFBR1	-0,08894624	6,58850869	0,343134628	0,400057141
PLA2R1	-0,129538554	3,343212122	0,343174705	0,400057141
ECE1	0,131197735	8,24695086	0,343207842	0,400057141
TFPI	-0,215543759	8,345956616	0,343638323	0,400490467
KIAA0922	-0,127527287	5,037733006	0,34428189	0,401171943
PARP3	-0,098107453	5,216602897	0,345318067	0,402310593
BTRC	-0,07376146	5,073609571	0,345702718	0,402689927
TWSG1	-0,09679602	6,429879242	0,345840576	0,402781706
PIGK	-0,084146858	5,822207111	0,347307279	0,404371876
PHKB	-0,091541538	7,002974895	0,347324546	0,404371876
TOM1	-0,08459975	6,750407934	0,349170247	0,406451334
CCDC111	-0,084079514	3,683245535	0,349290754	0,406522214
LRP5	0,118999832	7,451642148	0,350817734	0,408229715
ATXN7L3B	0,086002282	7,553678026	0,351525048	0,40898299
ANKRD17	-0,088367448	7,096187173	0,352270823	0,409780747
FAM32A	-0,073061381	6,688735604	0,352706721	0,410217829
PPP3R1	0,060282664	6,885336008	0,352906973	0,410380738
LAT	0,173384764	3,777874028	0,354603721	0,412283508
FMOD	0,177873016	6,927194749	0,354735005	0,412365837
NIPBL	-0,10104289	6,79121798	0,356074551	0,413852457
TMEM55A	-0,117770493	3,643609316	0,356243025	0,413977707
POLR3F	0,083442823	4,107150712	0,357214648	0,415017102
APPL2	-0,095826922	5,733888522	0,357266041	0,415017102
THADA	-0,061210718	5,965397202	0,357353433	0,415017102
AMBRA1	-0,070105091	5,832964866	0,35738091	0,415017102
EPC2	-0,07101123	5,434320678	0,357699653	0,415316523
MEAF6	0,08813758	6,662506573	0,358743212	0,416457263
USP31	-0,095956402	4,635342753	0,359334286	0,417072424
CTDSPL2	0,073385019	5,457021469	0,359437042	0,417120692
LRBA	0,101443616	7,008447761	0,35966699	0,417316523
NAP1L1	0,07702768	8,97667156	0,360409677	0,418107109
RAD17	0,067768623	5,238166598	0,360488606	0,41812754
DBNDD2	-0,104413536	5,820104673	0,361066014	0,418726045
HERPUD1	0,103521114	7,853006389	0,362239786	0,420015831
RNF168	-0,129538306	3,603688196	0,363543912	0,421456296
CPNE3	-0,073344145	7,662609047	0,36394894	0,421746388
EFHA1	-0,086991644	5,871454004	0,363963139	0,421746388
IL8	0,302149335	6,565793693	0,363988977	0,421746388
CTSC	-0,123943334	9,112043427	0,364041537	0,421746388
IFIH1	0,115394416	5,754717761	0,364132358	0,421750602
BLOC1S1	0,123588388	6,505630556	0,364168873	0,421750602
PRKACB	0,161878792	6,26318444	0,364471433	0,422029326
PDDC1	-0,080735959	6,228017509	0,364989378	0,422557312
DDX19B	0,063842105	5,672705418	0,365857077	0,423489969
USP36	-0,083986315	6,182573981	0,36666846	0,424303928
IQCE	-0,117788013	5,595457966	0,366684711	0,424303928
GDI1	0,090516114	7,446563786	0,367474037	0,42514514
SNX17	-0,075807854	7,611004982	0,367952492	0,425626471

PSEN1	-0,061597291	7,130902042	0,368123855	0,42575247
SMCR7L	-0,064082028	5,936620853	0,368337603	0,42592744
SENP3	0,066062704	5,769788215	0,368803442	0,426393807
CITED4	-0,186523645	3,416215476	0,369802122	0,427475956
RBM39	-0,063916231	7,680762118	0,370395984	0,428089867
PRKCI	-0,095308138	6,692288682	0,37085208	0,428538995
CCDC57	0,123192232	5,246719201	0,370910273	0,428538995
PELI1	-0,125205599	6,628608582	0,371135243	0,428726278
RPL15	0,11314958	10,56737611	0,371316274	0,428862749
AFG3L2	-0,073775675	6,320296666	0,372060222	0,429649222
ANKRD16	0,065461189	3,550233933	0,372694981	0,430309359
POR	-0,100081762	7,7709583	0,373293979	0,430927991
ATP6V1H	-0,064113453	6,312443809	0,373754844	0,431386981
PRKRIR	-0,091836723	6,458009307	0,374015098	0,431614309
GGA3	-0,07436205	5,977207043	0,37417137	0,431721586
RBCK1	0,084895297	7,013926825	0,374630051	0,432142437
SLC25A46	-0,086235995	5,385290492	0,374662866	0,432142437
PIK3CB	0,097669503	5,972423604	0,375226963	0,432719881
NBR1	-0,071448541	7,90230838	0,375365723	0,432806707
TMEM181	0,109214912	6,412392872	0,377184302	0,434830051
COG7	0,072453672	5,287260503	0,377753736	0,435412902
ABCD3	-0,095477133	6,74780087	0,378641562	0,436362483
RAB1B	-0,056045684	8,503485115	0,378977376	0,436675689
ORAI2	0,087817229	6,540747552	0,379436571	0,437130931
KIAA0319L	-0,097896908	7,528152184	0,379731506	0,437396814
RALGAPB	-0,077281832	6,660631584	0,381128223	0,43893149
ARL6IP5	-0,110371286	8,412193007	0,38143831	0,439214427
USE1	0,082989469	4,727639608	0,381925409	0,439701057
ARHGDIB	-0,121837087	8,556791118	0,382441796	0,440221237
GPR137B	-0,110098963	5,05378283	0,38329237	0,441125852
TTC30B	-0,105756904	3,754193662	0,383635953	0,441446771
RPS28	0,100939826	8,929494663	0,384903642	0,442830764
HNRNPUL1	0,071610923	8,621872765	0,385357926	0,443278626
SETD1B	-0,087866944	5,76522159	0,385457827	0,443318759
LEMD3	-0,091834336	5,656451274	0,385661747	0,443478492
PVRIG	0,139898682	2,354171192	0,385853717	0,44359798
TASP1	-0,078557278	3,912175579	0,385895765	0,44359798
LAPTM4A	-0,076837334	9,054692341	0,386225715	0,443902436
SHROOM1	0,112387276	4,600004581	0,387547355	0,44534638
TPP2	-0,073166421	5,986186779	0,387657108	0,445397442
PUM1	0,058939097	7,149489489	0,388971216	0,446831995
CWC27	0,068999136	4,931479219	0,389237232	0,447062268
ZFP91	-0,062537097	6,908549133	0,390800444	0,44878212
GCFC1	-0,071990549	5,001230071	0,391039552	0,448981092
IFT52	0,090458076	5,464983381	0,392417328	0,450487165
ELL2	-0,111376041	7,076404987	0,393266298	0,451385774
SF3A2	0,08600982	6,153732944	0,393607097	0,451700907
METTL3	0,086656166	5,279871356	0,393980389	0,452053217
PTPN2	0,071009373	5,643856577	0,394169943	0,452194623
ZNF639	0,075699403	5,341932266	0,394804888	0,452846852

EXTL3	0,09712076	6,109812518	0,395268407	0,453302265
SENP2	0,084525308	5,748087675	0,398495383	0,456926184
PLEKHO1	-0,123498088	6,238257221	0,398727348	0,457115297
SNAP29	-0,058768498	6,008404207	0,399547748	0,457978835
ARL5A	-0,073443744	6,430065574	0,400185065	0,45856573
MAD1L1	0,08842392	5,33297163	0,40019426	0,45856573
ZNF460	-0,114875284	1,751812685	0,400557321	0,458904633
DIP2B	0,090267389	6,252260093	0,401053026	0,45936155
ZNF697	-0,107218745	3,341316915	0,401090873	0,45936155
UBN1	-0,075507826	6,509035313	0,401503358	0,459756742
HEXDC	0,116803267	4,641643371	0,401665053	0,459864674
CHMP1B	0,091259864	7,364003466	0,402126503	0,4603157
CETN2	-0,10604868	6,738910971	0,40369386	0,462032297
LOC440944	0,123731009	4,035919759	0,40391873	0,462204667
KIN	0,062380312	3,892347165	0,403980029	0,462204667
CST3	-0,12080635	8,976818712	0,40480969	0,463076208
SMURF2	0,110610335	5,988976513	0,404935953	0,463142949
SMURF1	0,103945325	6,765541445	0,405936944	0,464209964
ODF2	0,086647763	5,712279211	0,406648092	0,4649007
ELMO2	-0,064516494	6,046143587	0,406677325	0,4649007
C6orf226	-0,097890118	2,566011805	0,407622557	0,465903154
DYNC1I2	0,050748568	7,367593536	0,408124421	0,466398596
WDFY1	-0,062184064	6,455230366	0,40825118	0,466465281
LSM6	-0,074846073	4,410535306	0,409218862	0,467492615
QSOX1	0,116819176	9,218339624	0,409340647	0,467553412
RREB1	-0,086709632	6,322539552	0,410090061	0,468330954
DGCR8	-0,068059284	5,413368504	0,410534105	0,468759555
SAP18	-0,077288587	7,384039939	0,411136038	0,469368264
ACO1	0,11436332	6,799362177	0,411984475	0,470258142
CHST14	-0,072691508	5,126517217	0,412135148	0,470314444
TTLL5	-0,079517121	5,168988391	0,412171743	0,470314444
COPS7A	0,080777432	6,693141021	0,41413728	0,47240569
WDR11	-0,06189897	6,243667549	0,414143015	0,47240569
CLK3	-0,074737601	5,733589451	0,414709783	0,472905277
SLC25A19	0,085440939	4,522464336	0,414719689	0,472905277
TLK2	0,059461844	5,973233385	0,415549196	0,473771939
TTC31	0,056652605	4,946805529	0,416020386	0,474229859
ICK	0,081584379	5,536390508	0,417477208	0,475810978
ACTR10	0,0721091	6,437860013	0,417860834	0,476168621
ACD	0,075517765	4,404322908	0,418280512	0,476495611
PNPLA4	0,10215253	4,850421845	0,418287538	0,476495611
TERF1	-0,046654646	5,386276417	0,419853293	0,478199366
CD164	0,084589414	9,044291204	0,420205564	0,478520665
JOSD1	-0,068949341	6,983805378	0,420884453	0,479213739
TNFAIP8	0,116850411	5,292358771	0,421846485	0,480228911
TMEM120B	0,101034674	6,212531866	0,421982508	0,480250744
PIBF1	0,078405315	4,802659657	0,42200652	0,480250744
RABAC1	0,091565192	6,977011295	0,42213393	0,48031558
LANCL2	0,090512336	4,915310399	0,423777797	0,482105572
NCK1	0,093846172	5,605614634	0,424288919	0,482606529

ASH2L	0,10032661	6,15286106	0,424792151	0,483078168
PRKD2	-0,071105956	6,276538633	0,424845252	0,483078168
PROSC	-0,099852065	6,358981833	0,426030098	0,484344654
EBPL	0,100988829	5,444829026	0,426881939	0,485232196
CROT	-0,092354456	4,376530296	0,427156243	0,48546307
LGALS8	0,075230464	6,232294998	0,427232193	0,485468476
RAB3D	0,102788977	6,163217941	0,428331236	0,486636238
C16orf72	-0,049315692	6,171486705	0,429110542	0,487411812
MRFAP1L1	-0,06237507	6,457918163	0,429156844	0,487411812
MPHOSPH6	0,07744064	4,420902508	0,430088007	0,48838803
NUDT21	0,056537351	7,004614092	0,430309449	0,488558131
LOC401397	0,09503063	5,922145935	0,430965699	0,489221758
ZNF782	-0,083814701	1,679582904	0,431223632	0,48943308
ATP6AP2	0,058245935	8,018386557	0,431449672	0,48960814
KIAA0100	-0,074066148	8,093218355	0,432167013	0,490340576
TRAM2	-0,093961101	6,169802742	0,432779801	0,490954161
RPS6KA3	-0,081397317	7,459796911	0,435165314	0,493578222
CRLF3	0,068938862	5,133698962	0,435462886	0,493833597
EXOSC9	0,059548693	4,884491891	0,435928756	0,494279712
RNPC3	-0,100518065	3,492189132	0,436579281	0,494935016
SAP30BP	0,059817643	6,209985989	0,436812137	0,495116682
MRPL14	0,116323318	6,995359306	0,438915018	0,497417568
DDA1	-0,063198737	5,870098568	0,439124839	0,497572662
EDIL3	-0,190105424	5,627023401	0,4404091	0,49894495
ELK3	0,116037783	5,610345412	0,441181119	0,499736554
CPNE8	0,102618683	4,864712802	0,441521699	0,500039273
RBM15B	-0,06138784	6,724605875	0,442400576	0,500951433
NCOA2	0,120413322	5,525184778	0,44318955	0,501761505
MYO18A	-0,093039627	6,84591889	0,443463953	0,501928284
ETFB	0,091066241	6,493113388	0,443484076	0,501928284
INPP4A	-0,065896569	5,680908543	0,443866335	0,502277554
MMGT1	-0,068594744	6,172315278	0,445881011	0,504473637
FAM46C	-0,129075064	6,468785303	0,446897227	0,505539514
PEX12	0,065587569	3,773351378	0,447834694	0,506515969
GLOD4	0,060604601	6,301972805	0,449073447	0,507832809
PPDPF	0,111655149	8,384219554	0,451148334	0,510094592
GALNT11	-0,079644008	6,355899633	0,453144002	0,512266073
CUL1	-0,047054544	6,600732355	0,453918256	0,513056289
CCDC130	-0,068407863	4,374797943	0,454023289	0,513074185
CC2D1A	0,063616434	5,832442223	0,454084573	0,513074185
PGRMC2	0,060281059	6,660614216	0,454466976	0,513421191
UQCRB	0,078656993	7,720899467	0,455366777	0,514352501
ARPC5	-0,082794909	8,603512835	0,455872543	0,514838501
SH3BP4	0,100217158	6,599540524	0,456161786	0,515003916
NECAB3	-0,104077675	5,790572699	0,456170062	0,515003916
GALNS	0,087485087	5,192723298	0,456969129	0,515820641
TRA2B	-0,043261809	7,226738146	0,457203183	0,515999421
WDR1	0,057416971	8,835503896	0,45877014	0,517598526
SH3BGRL3	0,108910188	8,51075152	0,458807822	0,517598526
IER5	-0,111819495	6,362313828	0,458847792	0,517598526

ATG5	0,073617377	5,924294724	0,459265439	0,517983962
TERF2	0,054450615	5,263437813	0,459564627	0,518235686
UBE2Q2	0,086663099	6,180290505	0,459790651	0,518269045
XPO7	0,063020315	6,70951672	0,459801578	0,518269045
SART3	0,060065024	6,205247301	0,459822221	0,518269045
AFTPH	0,057410985	6,522717678	0,460547189	0,518959035
CD3G	0,13219804	2,705854681	0,460586608	0,518959035
LARS	0,05881749	7,027076961	0,46071129	0,51901376
H2AFV	0,058703429	8,015139479	0,461357895	0,51965634
ZBTB37	0,081491575	0,867080171	0,462761058	0,521150727
TMBIM6	-0,057391498	10,72982314	0,463132666	0,521483099
ELOVL5	-0,064680889	7,529234606	0,464307879	0,522720064
TXLNA	0,055233768	6,998813382	0,465298487	0,523748825
HOOK3	-0,078255399	6,760751491	0,465922131	0,524364253
TNIP1	-0,062524657	7,868325489	0,466039585	0,524409889
JARID2	0,089440296	5,09103834	0,46650801	0,524837354
MYO6	0,084470765	7,628188665	0,466573404	0,524837354
ZKSCAN1	-0,087717525	5,443673735	0,46665965	0,52484779
ARL3	-0,072935534	5,396186338	0,467157565	0,525321147
RARS2	0,070369459	5,612781063	0,467491093	0,525609524
ANKRD36BP1	0,107207273	2,943685004	0,468542675	0,526704995
TRIM26	-0,057513926	6,586226334	0,469225958	0,527320096
SEPT8	-0,065741073	6,117990254	0,469244514	0,527320096
USP15	-0,055004508	6,090882464	0,46953641	0,527561177
C9orf3	0,111445716	5,781348199	0,469895182	0,527877306
ZNF841	0,093768417	3,977759771	0,471291428	0,529358631
SKAP2	-0,090395059	6,733809029	0,474358934	0,532716333
NOL9	0,070162883	3,904227633	0,475262227	0,533642866
KLF5	-0,113732111	7,346497248	0,475484295	0,533804315
ZFAND2B	0,060906655	5,031330246	0,47638914	0,534732106
NSMAF	-0,052801601	5,502135411	0,476757205	0,535057173
TMEM133	-0,099443422	3,409940301	0,477035749	0,535281681
NBN	0,070550619	6,508729304	0,478638113	0,536991328
SPPL2B	0,055466584	6,073958396	0,481877165	0,540536337
WDR26	0,044520558	7,579295212	0,482457168	0,541097933
CD99L2	-0,092713505	6,841363883	0,483114455	0,541746007
ST13	-0,052686773	8,602788673	0,4844851	0,543122427
TBCK	-0,054816119	4,758856614	0,484515711	0,543122427
CPT2	-0,052375928	5,685325173	0,484580855	0,543122427
GBE1	0,082198673	5,775579452	0,484905626	0,543397118
UBXN2B	-0,052771538	5,651463192	0,485034166	0,543451853
ATP6V1D	0,053252655	6,446926185	0,48581686	0,54423939
MED24	-0,074040652	6,798702877	0,486958863	0,545429121
RNF214	0,055362501	4,465018725	0,488192661	0,546669208
SGSM3	0,079764115	6,309485966	0,48822635	0,546669208
WDR20	0,045532306	4,806150426	0,488672389	0,547078809
SLC10A7	0,060881849	4,215123419	0,490226871	0,548728995
FAM35A	-0,052648568	5,495739817	0,490325752	0,548749598
PTGR2	-0,068060283	4,257306654	0,490941821	0,549297742
ANKRD36	-0,093020247	3,196793661	0,490976644	0,549297742

RAD23A	0,055805551	7,389417308	0,491708166	0,550025916
EIF1AX	0,066890459	7,758180264	0,491973373	0,550232316
WASL	0,05376647	6,912118917	0,492337069	0,550548782
AGL	0,083946148	5,561629519	0,492932039	0,551123721
GZF1	0,065667961	4,845458777	0,493208069	0,551341938
CSNK1A1	0,049817729	8,374513176	0,493843259	0,551961512
BIRC2	-0,061593722	6,375504549	0,49432143	0,552405414
PMS2L2	-0,050775482	3,297140971	0,494555275	0,552576179
ADM	0,188910706	5,047806494	0,495418411	0,553449892
LOC100288778	-0,078986211	4,896198381	0,495596269	0,553557896
NUDT4	0,07436498	7,220584492	0,496363982	0,554324598
UBE2G1	0,050144992	6,222921376	0,497033132	0,554980993
UBTF	0,053649542	7,230551543	0,497215889	0,555094163
OTUD5	-0,061786953	6,612499773	0,49742078	0,555232002
PPARA	-0,058293184	5,394509344	0,502212337	0,560488695
ANXA4	-0,111185538	8,060394186	0,502521798	0,560742292
PRR12	-0,080650813	6,029882192	0,50329416	0,561512251
NRBF2	-0,045363588	5,193023879	0,504633454	0,562914367
LENG9	-0,074463114	3,178920331	0,505606785	0,563907862
ZNF595	-0,075467103	3,802032982	0,508349103	0,566873677
HK1	-0,062241561	7,6886841	0,508705328	0,567178161
KPNA4	0,055843466	6,946808648	0,509624	0,56810954
ZFC3H1	-0,068644386	5,329310945	0,511128334	0,569693382
FBXL18	0,082326931	5,207467839	0,513275174	0,571992713
RNF14	0,065325568	6,242240996	0,513553797	0,572209697
RXRB	0,049222902	6,144778168	0,515250809	0,574006742
VAMP7	0,061871032	6,242343946	0,515368714	0,574044309
PPP2R4	0,049054703	7,643000181	0,516325425	0,575016018
ATRIP	-0,041245276	3,361044109	0,516818236	0,575470861
GLYR1	-0,044359189	7,220686997	0,51700096	0,575580334
SNW1	0,042542533	6,480524553	0,518233145	0,576857952
UBE4B	0,062711001	6,274874769	0,518600245	0,577172364
TRIM8	0,079789326	7,748378363	0,518851712	0,577358
IRF2BP2	-0,051874745	8,270303944	0,519834945	0,578347483
ZBTB11	-0,039230082	5,377705405	0,519910555	0,578347483
MGST2	0,083395953	5,704346342	0,520047348	0,578405296
PLA2G12A	-0,058844331	5,822124191	0,520424748	0,578730652
MAP3K1	0,070341898	5,720223854	0,521189733	0,579486839
FLJ45445	0,11878962	4,773840313	0,521869239	0,580147757
PCYT1A	0,060591962	4,818761565	0,523297775	0,581640999
GAK	-0,05659197	6,92138383	0,523584691	0,58186506
PPP2R3C	0,087176225	5,221694886	0,523673906	0,581869376
EXOC1	0,046405657	5,88996323	0,523857004	0,581977992
C4A	-0,134115537	9,03091249	0,524354532	0,582435829
FAM48A	-0,047887543	5,701407758	0,525472522	0,583582594
LUC7L3	0,066089592	6,861361366	0,526007853	0,584011346
PILRB	0,114054814	5,322038879	0,526029871	0,584011346
ZNF154	0,12505806	1,971426679	0,526140467	0,584039044
TEP1	-0,067467426	5,905936341	0,527892333	0,585835982
ATG4A	-0,055448316	4,754845376	0,527931091	0,585835982

RCOR3	-0,057477545	5,643448394	0,528908662	0,586825279
EIF4G2	0,043738468	10,52119938	0,529212333	0,587066682
PIAS4	0,050980819	4,128200351	0,530391785	0,588279372
USP32	0,062486324	6,428997299	0,531074143	0,588940409
STAB1	0,115840014	6,499682262	0,531446235	0,589257216
FGFR1OP2	-0,076292687	5,432067874	0,532015472	0,589792473
EDC4	-0,054088965	6,263024597	0,533567103	0,591416463
PLAGL2	-0,082179124	5,962943193	0,533728957	0,591499716
NUP188	0,065783166	6,528134144	0,533822313	0,591507044
MMS19	0,045623622	6,411564753	0,533973797	0,591578768
LIMA1	-0,077670132	7,155747451	0,534143802	0,591670986
DPYSL3	0,114423851	8,033422356	0,534369855	0,591825246
TBPL1	0,064723136	4,190565164	0,534762781	0,592164242
GTF2I	-0,051879023	8,44363446	0,536097924	0,593529385
AP2B1	0,056189009	8,641154463	0,536169675	0,593529385
NKAP	0,041281428	4,958015854	0,53633141	0,593554624
IRF2BP1	0,072510901	5,195413413	0,536366563	0,593554624
HSPH1	0,075009057	7,199100913	0,536519105	0,593627094
NINL	-0,096439833	4,613264914	0,536681944	0,593710931
PARP10	0,071642699	6,538888817	0,53772446	0,594767736
CAP1	0,054054582	9,249174151	0,538632582	0,595666388
CAPZA2	-0,081539974	8,047079598	0,538711632	0,595666388
NLK	0,052145368	4,989926853	0,539598728	0,59655054
TMEM219	0,073762202	7,406900713	0,539976114	0,596870988
CAPG	-0,079360182	8,498702648	0,541398389	0,598311157
ZNF629	-0,071331872	5,636273236	0,541536319	0,598311157
CNBP	-0,047750403	8,954847689	0,541542229	0,598311157
AGGF1	0,041316343	5,668086898	0,543035534	0,599863811
WDR33	0,039203989	6,603874953	0,543283771	0,600040821
HEATR5A	-0,065576156	6,033861756	0,544136476	0,600885285
DNAL4	-0,0541502	5,186349902	0,54517564	0,601935345
DDX21	0,076868328	7,119902013	0,545509708	0,602206686
RLF	-0,047570156	5,239081127	0,545685378	0,602303106
ELK4	0,068064398	3,385214513	0,545869601	0,602408935
WRN	0,060504218	4,229112076	0,546599287	0,603116592
WIZ	-0,055704075	5,817557621	0,547315819	0,603809508
BAG3	0,085170599	7,002378716	0,547999394	0,604465848
ZNF672	0,053791339	6,075888316	0,54840921	0,604820056
DNAJC8	0,050498734	7,115706826	0,548758928	0,60510788
PPP1R2	0,047151197	6,669946325	0,549113096	0,605400518
RNF114	0,046713095	6,874001926	0,550024514	0,606307332
VGLL4	-0,039745943	6,532583884	0,550337868	0,606554699
PFDN5	0,090404124	8,696206002	0,551174423	0,607378537
ZBED5	-0,052106357	5,746559156	0,551441399	0,607574551
BCL2L1	-0,066272045	8,061897517	0,551533722	0,607578101
GIGYF1	-0,072399357	6,141114071	0,551714346	0,607627487
DDX28	0,055141002	4,351130249	0,551756768	0,607627487
AEBP2	0,06588592	5,672389236	0,552000369	0,607797597
TOR1AIP2	0,058260022	5,294849035	0,552164433	0,607861417
HNRNPUL2	-0,05762605	7,55593673	0,552236615	0,607861417

UBAP2	-0,064534691	5,854184432	0,55299629	0,608599371
CYB5R1	0,07028901	6,946101214	0,553253672	0,608784378
LRP10	0,063794566	8,658979234	0,553371197	0,608815455
PCCA	0,101120551	4,925263527	0,553507868	0,608867584
TPCN1	-0,065153502	6,659626519	0,554149071	0,609474599
B9D1	-0,08924585	3,782632767	0,555487235	0,61084784
PLEKHJ1	-0,052981626	5,714280096	0,555832683	0,611129162
CMIP	-0,063053015	6,99227432	0,556208721	0,611383475
CUL3	0,037882874	6,803905348	0,556243302	0,611383475
DDX46	-0,045355048	6,40356611	0,556526605	0,61159628
DNASE1L1	0,067073051	5,848704096	0,557143716	0,612175798
GLT8D2	-0,101665417	3,762163937	0,558537065	0,613607901
ANKRD13C	-0,046854349	5,304038934	0,55865388	0,613637372
SUOX	0,057794233	5,51386648	0,560941833	0,616007245
TRAPPC9	0,058750327	5,742181378	0,561037536	0,616007245
BCAT2	-0,060843094	5,878125102	0,561170001	0,616007245
AKAP8L	-0,049183337	5,486231284	0,561172754	0,616007245
PAK2	-0,047901277	7,486135042	0,561896743	0,616702703
KLHL21	-0,060568316	5,976688359	0,56342594	0,618281539
LPCAT1	-0,120304587	10,1029141	0,564000609	0,618812575
CTSL1	-0,106776603	8,238954486	0,564220181	0,618953896
GATAD2B	-0,060083075	6,124593606	0,56453207	0,619196426
MAP1LC3B2	0,057661495	3,272417393	0,565777135	0,62045032
LETMD1	0,064712069	5,965957513	0,565936724	0,62045032
MALL	0,127998417	9,056364068	0,565948231	0,62045032
YTHDF2	0,036086732	7,165613367	0,566934731	0,621333929
RSF1	-0,061808238	5,571581928	0,566936458	0,621333929
UBXN1	0,045244143	6,940454812	0,567233292	0,621559348
KIRREL	-0,100963879	4,232721315	0,567536998	0,621792222
SYMPK	0,055809476	6,935896082	0,570437031	0,624869095
AMN1	0,068852501	3,749740243	0,571561427	0,62600022
IMPACT	-0,054665349	5,835531348	0,571705238	0,626057173
C10orf47	-0,077633418	4,593889371	0,57228461	0,626460829
PRDX6	0,052049377	8,477123613	0,572318635	0,626460829
SLC11A2	-0,056817287	6,873681543	0,57234946	0,626460829
FYB	-0,095361303	5,619060336	0,572462755	0,626484277
FN1	-0,111972448	12,2014632	0,572809328	0,626762966
ETHE1	0,091600237	5,398110264	0,573054729	0,626930883
GLT8D1	-0,035821823	5,873895079	0,573202425	0,626991873
FADS1	-0,080118715	6,603900241	0,573348457	0,627014211
THOC2	0,052168611	6,572445111	0,573406748	0,627014211
FBXW9	-0,059838397	3,585863109	0,57357923	0,627102255
LRCH4	-0,05516087	6,29099883	0,573866438	0,627315684
PKN2	-0,048861367	6,350919081	0,575919999	0,629459604
CAMK1	-0,064039531	4,892847602	0,576022407	0,62947064
ARFRP1	0,0548601	5,547332102	0,576238	0,629605339
ZNF326	0,053393426	4,412934457	0,576477659	0,629766286
DDX42	0,050583634	7,445155408	0,577412969	0,630687015
USP7	-0,041238336	7,189438888	0,577756106	0,630960744
OFD1	-0,054924195	5,451692629	0,579581738	0,632853142

CD99	0,065874373	8,009346122	0,580391415	0,633635777
CDC42	-0,052648246	9,170907967	0,58382929	0,637287007
PSMD6	0,03861889	6,147898919	0,584957642	0,638416479
IL32	0,102205969	7,895122515	0,58698514	0,640526751
RECQL	0,063152506	5,775981886	0,587158192	0,640613074
MSH3	-0,036629668	4,819403367	0,587450054	0,640828975
DTX3	-0,073098746	5,017504181	0,588350308	0,641708373
BMP1	0,088254358	5,881582908	0,590257576	0,643685657
CDK11B	-0,047056881	5,893461017	0,591771925	0,645233891
FAM120C	-0,068772709	3,966693399	0,592390524	0,645805114
VEGFB	-0,045752543	6,880380404	0,593339639	0,646736413
WSB2	0,050568628	7,091031572	0,593980872	0,647331879
EIF3G	0,046227982	7,345787577	0,594803306	0,648124599
MGAT5	0,090887222	4,238003229	0,595793452	0,649099784
INTS12	0,041250315	4,715894865	0,595993246	0,64921373
CNOT6	0,044274951	6,127247859	0,596143529	0,649273714
C8orf40	-0,064822393	5,174951072	0,596317753	0,64935975
ZNF514	0,071057467	3,804713986	0,596483003	0,649435988
DYNLL2	-0,05553661	6,862316599	0,597330488	0,650254885
SCARB1	0,092614441	5,958056866	0,597482274	0,650316302
RNMT	0,044117279	5,876237167	0,597686264	0,650434509
LDB1	0,046518347	6,302825055	0,598226717	0,650918778
FAM160B2	-0,049772726	5,854791292	0,598677216	0,651305032
MLLT3	0,077202543	5,019570554	0,599596193	0,65220074
ANPEP	-0,139486268	6,574073709	0,600143824	0,652692303
TAB3	-0,047653031	5,656460944	0,600776449	0,653276129
SH3YL1	-0,065212549	6,353746936	0,601113227	0,653491397
TEX264	0,054451222	6,305022491	0,601166085	0,653491397
WRB	-0,064448032	5,483143351	0,601595642	0,65385411
STX5	0,039640607	6,201126121	0,602498658	0,654731211
SAMD8	-0,050974434	2,825773913	0,602969259	0,655138206
LARP4	0,044160738	6,46269156	0,603589563	0,6557077
UBQLN1	-0,034932864	7,509630873	0,605452894	0,657627156
ANAPC13	-0,043542819	6,235930238	0,607079387	0,659288794
HERC5	-0,07526489	4,35043587	0,607193519	0,65930774
RALGAPA1	-0,081139465	5,715836402	0,607660666	0,659709932
LSM14A	0,0470312	7,46914455	0,60788261	0,659845833
LPIN2	0,085267668	7,488559467	0,609824074	0,661847901
BIRC6	-0,049382769	6,874060146	0,610013594	0,661948233
PARP6	-0,048858367	5,59542851	0,61031046	0,662120719
HSBP1L1	0,073507829	4,336468933	0,610366746	0,662120719
YTHDF3	0,036186245	6,977806796	0,612672412	0,664516173
NAGA	-0,041614159	6,588553033	0,61306514	0,664836385
STX16	0,052154748	6,628873249	0,613484694	0,665131887
MGA	-0,048987982	5,732652598	0,613532713	0,665131887
FAM18B2	0,041526394	4,293180762	0,616294035	0,668019238
C16orf62	0,048262919	6,232851437	0,616928764	0,66860096
TGFBRAP1	-0,061478159	4,340226627	0,61742348	0,669030782
KIAA1279	-0,036652271	5,285200256	0,617525663	0,669035192
C2CD3	-0,052089472	5,399568744	0,617848863	0,669279014

ANKRD36B	-0,070664505	3,019798526	0,61844869	0,669770885
KIAA1191	-0,047495033	7,507689649	0,618499378	0,669770885
FNBP1L	0,055700034	6,809236904	0,618823467	0,670015437
C9orf123	0,069145206	5,544412019	0,619694949	0,67084931
BLMH	0,068365611	5,422695005	0,619790388	0,67084931
JAG2	-0,082681067	5,394870452	0,621080857	0,672139401
FDPS	0,063829707	7,677571511	0,621517492	0,672505202
SYNCRIP	0,039037238	7,830206468	0,623174707	0,67419139
HCFC1R1	0,072173585	6,016450886	0,624499902	0,675517898
ADNP2	0,048723876	5,484375152	0,625873921	0,676896791
SPCS3	0,044230757	7,902170793	0,626958513	0,677902159
ANTXR2	0,068122121	6,567485372	0,627002334	0,677902159
CASP9	-0,049596309	4,753805233	0,627586297	0,678425961
TBC1D9B	0,049117378	7,632011498	0,627812625	0,678563051
FAM175B	-0,02694112	5,070032936	0,628744834	0,67946292
DPP7	-0,054765977	7,30669018	0,629100922	0,679691379
EIF4H	-0,035252125	8,886937828	0,629155592	0,679691379
SPDYE7P	-0,051977312	4,53680326	0,629390374	0,679837314
DCXR	0,067241911	6,174096581	0,629857867	0,680234526
BAZ2A	-0,053791542	7,523595295	0,630432678	0,680747495
RAB35	0,033292467	6,215645646	0,630608773	0,680829833
LOC100271836	-0,049873891	4,156065939	0,630867333	0,681001162
AIM1	0,092789708	6,498043559	0,631702141	0,681794381
CEP170	-0,054173454	6,081397927	0,631823017	0,681816926
GIPC1	0,05073769	7,364471162	0,632566963	0,682485826
ZBTB45	-0,041380153	4,188834727	0,632643042	0,682485826
GRINA	0,046812781	8,147164142	0,63369866	0,683516476
CUL4A	0,04049697	6,833892694	0,635309332	0,685145396
TCF25	0,038735835	7,200933701	0,636002704	0,685784699
C4orf27	0,046977365	4,617733666	0,636702424	0,686430644
PIAS2	0,058805162	3,933271734	0,63721402	0,686873601
NUS1	-0,034626048	6,94185244	0,637316447	0,686875431
PPFIA1	0,043860236	6,452795119	0,638378536	0,687911384
HPS6	-0,035968679	5,125472482	0,638690589	0,688138905
MTIF3	-0,040478127	5,302850807	0,640136742	0,689588064
PTPLB	0,063156305	4,408953905	0,643610088	0,693220216
SPG7	0,039909663	6,16958217	0,643865688	0,693385996
AHR	-0,074489547	8,452693857	0,644455807	0,693911913
ZNF880	0,097002066	3,337409919	0,644602412	0,693960191
ZNF273	0,047116785	3,939020544	0,64531793	0,694620831
LOC100129034	0,058841201	7,038178437	0,646060267	0,695310126
CHMP2B	0,041272723	6,437143363	0,647271742	0,696504025
LOC283070	-0,089415546	5,112981274	0,647808515	0,696867797
C1R	0,062071208	8,935442538	0,64781419	0,696867797
MAPK9	0,039409382	6,316084143	0,648707094	0,697718245
TRIP4	0,035653569	5,25907335	0,649052768	0,697979944
KCNQ1OT1	0,084913609	2,856872581	0,650431268	0,699352068
C11orf49	0,048611856	5,570115834	0,651104181	0,699965224
DLD	0,041512339	6,617700549	0,652228783	0,701063693
ZNF772	0,048902326	3,624844054	0,652473986	0,701216723

SLC1A3	-0,084200439	4,569344731	0,653103167	0,701684056
NOL8	0,036390047	5,177997443	0,653114636	0,701684056
AMFR	-0,041738777	7,196888525	0,653438992	0,701921941
USP38	-0,033911938	5,932491146	0,654547847	0,703002327
MBTPS1	-0,034457494	7,724415926	0,655063322	0,703443882
NDUFAF4	0,050585049	3,821944054	0,655165287	0,703443882
POLR1D	0,046897054	6,741763347	0,656864333	0,705099982
SIGIRR	-0,04835033	5,722785747	0,656914531	0,705099982
ERCC6	0,044947558	4,102698657	0,657643407	0,705771229
CTCF	-0,029003791	6,340259565	0,658102463	0,706152745
POLR2E	-0,030020442	7,338895043	0,660586603	0,708706741
TRIM14	-0,046146806	6,335286517	0,661753263	0,709846705
TM2D1	0,046884644	5,154702239	0,662129635	0,71013872
TPCN2	0,056467327	4,706840181	0,662292051	0,71014502
ATF6	0,035134932	6,876683411	0,662343793	0,71014502
ZNF227	-0,043087308	3,823175075	0,662644692	0,710343914
VKORC1L1	0,047327171	7,236239839	0,662737642	0,710343914
AIDA	-0,0316548	7,475557341	0,662975303	0,710486971
APOE	0,100907375	8,807337776	0,663277691	0,710699336
SDHD	0,049792536	7,249071557	0,663620948	0,710955419
CHKB	-0,051094051	4,265929024	0,664620146	0,71191404
B4GALT5	0,058360177	7,579799355	0,667201327	0,714566648
LGMN	-0,054077371	8,392902038	0,667434339	0,71470395
PRKCA	0,073505484	5,695728199	0,667727504	0,714905613
PCBP1	-0,022072805	8,893654689	0,667848829	0,714909949
SMAP1	-0,034982902	5,775513343	0,667941235	0,714909949
EIF4A2	-0,052981982	8,892644988	0,668267859	0,71514729
BBS5	0,050117868	4,939328123	0,668417098	0,715194758
ERP44	-0,031463074	6,930928992	0,668796427	0,715488365
RELA	-0,026929505	7,188724094	0,669341551	0,715959221
HERC6	0,058107471	5,202607754	0,66951015	0,716027245
CXorf26	0,035727521	5,097458482	0,671482038	0,718023524
ASTE1	-0,030104189	3,515473819	0,67263586	0,719144548
USP9X	0,040184393	7,665884651	0,67388649	0,720276149
DHCR7	-0,052735983	6,556401936	0,673905533	0,720276149
SF3B1	0,027565291	8,531709394	0,676175793	0,722589364
C8orf4	0,102446874	8,696521549	0,678337342	0,724785699
IMPA1	0,040554864	5,068522368	0,679324074	0,725726283
TSPAN3	0,059958724	9,121852839	0,679716536	0,726031807
TM4SF1	0,090867154	9,219237112	0,681018544	0,727308606
UNC93B1	0,052511593	6,926210271	0,681601084	0,727816754
KCTD17	0,050083747	4,178750458	0,682976858	0,729171629
TFIP11	0,028444632	5,778057792	0,684239644	0,730405468
ITPR3	-0,060338349	8,393958262	0,685878462	0,732040262
MAPKAP1	-0,027987524	6,810356956	0,685992704	0,732047613
DDB2	-0,045101442	4,983334201	0,687046301	0,733057225
SFMBT1	0,055097332	4,169060559	0,687327653	0,733242689
EPB41	0,042612024	6,580339328	0,688179546	0,734036653
LHFPL2	-0,056434572	6,71948867	0,6884872	0,734249955
AP3M1	0,02491629	6,454738881	0,689238358	0,7349361

THAP6	-0,027993818	4,426203194	0,689448139	0,734956984
SLC38A6	-0,050796098	4,242046735	0,689473504	0,734956984
PAK4	-0,044800531	6,610826439	0,689924754	0,735323053
DUSP6	-0,07499212	8,431423018	0,690338716	0,735649274
FZD1	-0,065183094	6,213021897	0,690550505	0,735759983
TARDBP	-0,023316594	7,497203617	0,691060445	0,736188279
SETMAR	0,040925794	4,280281385	0,691342522	0,736189084
DUT	0,040530524	5,821365715	0,691389467	0,736189084
TJAP1	0,030221279	5,43966066	0,69147264	0,736189084
FIS1	0,053354334	7,013822656	0,691493047	0,736189084
ATRN	-0,043288886	6,751942226	0,692226843	0,736855268
TAP2	0,054132752	7,180828234	0,692841753	0,737394711
NOTCH2	-0,04889541	7,938471956	0,693431899	0,737907634
C6orf47	-0,032715777	5,580861675	0,69439449	0,738816668
TECR	0,036415629	7,177425711	0,695979838	0,740387912
NOTCH2NL	0,054203182	5,570945835	0,697482809	0,741871046
SENP5	0,033408667	5,705327042	0,697874049	0,742171419
RAD50	0,031592466	6,099991613	0,698184103	0,742281746
EIF4E2	-0,030291811	6,881055038	0,698195501	0,742281746
NPAT	-0,031729784	4,960320691	0,699638202	0,743699595
RPL21	-0,058445344	10,04660879	0,702150312	0,746253581
FLOT2	-0,027318277	7,800607359	0,702590632	0,746605192
ZNF573	-0,040035191	1,528207358	0,702763552	0,746672587
REST	-0,048803396	4,139866396	0,703129485	0,746945001
HEATR7A	-0,036564478	5,659682895	0,704484981	0,748268393
CALM3	0,031193417	8,333615397	0,704945809	0,748641251
GLG1	0,037130469	8,236832858	0,705935491	0,749575539
DDX50	0,02664486	5,846167772	0,707290382	0,750897263
SPTLC1	-0,023091996	6,911009219	0,707511316	0,751014892
DRG2	0,028029883	5,367268621	0,708880617	0,752351272
CD14	0,075061653	7,391930451	0,70925109	0,752627323
FAM118B	-0,049631668	5,146197762	0,709759151	0,753049269
AGPS	0,029934395	6,457772252	0,709909214	0,753091308
FOXC1	-0,069978194	3,966650167	0,712284234	0,755493264
CXorf38	-0,024605801	4,469237556	0,712710809	0,75582815
SART1	0,036126864	6,53047426	0,71284381	0,755851647
PSMF1	0,025286292	7,435831672	0,713239757	0,756153903
ORAOV1	0,046930375	3,987251538	0,713797014	0,756627054
N6AMT1	-0,038827874	3,842528789	0,715182304	0,75797764
DNPEP	0,026199176	6,510655291	0,715458717	0,757998273
PAPD4	-0,025681076	5,920492636	0,715463793	0,757998273
MED13	0,038346571	7,061292708	0,715535252	0,757998273
PTPMT1	0,033763316	6,038394266	0,715654847	0,758007207
ATG4B	-0,02498682	5,83021008	0,716451202	0,758732839
SEL1L	-0,035119756	7,803856033	0,717222646	0,759431867
USP40	-0,034199347	6,129451349	0,717736696	0,759858179
SLFN5	0,053897597	6,260169529	0,718167207	0,760195931
GFPT2	-0,075176515	4,829385179	0,720796893	0,762861091
ZMYM5	-0,025928005	4,259535745	0,721268972	0,76324226
ADI1	0,04404926	7,437443693	0,721851233	0,763739884

GTF2F1	-0,026396831	6,619368034	0,722080184	0,763863602
ABCC1	0,052308429	6,90246196	0,722589519	0,764283842
C16orf80	-0,027299561	5,390075664	0,724353263	0,766022952
ASF1A	-0,040459755	5,211381842	0,724458428	0,766022952
CCNI	0,034400875	8,477036407	0,725470893	0,766921391
GNG10	-0,041698953	6,525647187	0,725533054	0,766921391
PLXNB2	0,050962926	9,05730307	0,725748075	0,767029777
GSTO1	-0,052160686	7,414294414	0,729396556	0,770760054
VPS41	0,032568155	6,608677533	0,72950365	0,770760054
SLC12A9	-0,031401792	6,058730216	0,729619694	0,770763237
ACBD5	0,033304709	6,068237837	0,729850066	0,770765309
SNX13	0,034859607	6,204709706	0,729901897	0,770765309
PAF1	0,035213807	6,541604139	0,730034531	0,770765309
PPP1R7	-0,024226729	6,402101822	0,730073784	0,770765309
DTNBP1	-0,024302206	4,769494209	0,731300039	0,771940398
VPS54	0,026681302	5,782827492	0,732071299	0,772634915
ZNF142	-0,031014204	4,932981522	0,733056611	0,773555096
STXBP5	-0,039793942	4,568919397	0,734084148	0,774519542
TPR	0,037788357	7,496556317	0,734612994	0,774957612
ATXN10	0,02686272	7,11711409	0,735021744	0,775199081
ASNSD1	0,029716209	6,068634711	0,735069256	0,775199081
PYCARD	-0,056617472	5,43764388	0,73598992	0,776049987
MED21	-0,044218368	5,476254217	0,73681514	0,776800006
COPS2	-0,030709975	6,613880175	0,739476948	0,779485749
MED12	-0,03868353	6,159232517	0,740151774	0,780076499
FLJ42627	-0,041645095	3,787537507	0,740423294	0,780242071
STK19	-0,026494263	4,666193198	0,740904888	0,780543709
PLEKHM1P	-0,040528375	4,286257663	0,74093847	0,780543709
DHODH	-0,028740106	4,052169383	0,741153357	0,780649481
ZNF649	-0,050423164	3,613527891	0,741359723	0,780746247
YWHAE	-0,02509633	9,385597469	0,74221739	0,78152878
CARS2	-0,02571498	5,575398801	0,743199441	0,782442023
NLRC5	-0,046100256	5,8214493	0,744095628	0,783264601
BCL2L13	-0,023983362	6,407958978	0,745432194	0,784550414
UBE2L6	0,043517281	7,301047004	0,745599587	0,784605491
RPL36AL	0,035176792	7,839201707	0,746041269	0,784949145
SRP14	-0,026938974	8,188756725	0,746724563	0,785546867
LRRC37B	-0,033904959	3,267418538	0,747068815	0,785787791
ZCCHC10	0,034673918	4,673410127	0,747258162	0,785865732
TMEM98	0,060709769	6,886101544	0,747869067	0,786386919
PDZD8	0,044955341	5,238068652	0,748201383	0,786615053
ACSL3	0,03242689	7,121457409	0,748767273	0,787053955
TMEM97	0,049678003	6,152270817	0,748849694	0,787053955
CHD8	-0,031423005	6,800971277	0,749532565	0,787650264
MRPL54	0,051209087	5,568077259	0,750479599	0,788523943
ZBTB6	0,030471112	4,460440168	0,751262146	0,789224552
FLNB	0,051084281	8,670272372	0,751482474	0,78933441
CRYL1	-0,042794101	5,595038393	0,752207795	0,789974581
MICAL3	-0,038359275	5,264532794	0,754061342	0,791799244
SEC16A	0,035688033	7,600410831	0,754982336	0,792644273

SPRED2	-0,03842167	6,512812119	0,756584813	0,794204409
TOMM7	-0,032381113	7,576013426	0,757763571	0,795319346
LTV1	-0,02287549	5,155747657	0,758702474	0,796182236
U2AF1	-0,024211651	6,661298817	0,760719239	0,798175795
STAG2	-0,022013162	7,310688618	0,761064163	0,79841485
FBXW7	0,035014418	4,8264127	0,762914829	0,800233228
LOC493754	-0,022085253	5,392661285	0,763059655	0,800262041
GPC4	0,061988003	7,28001712	0,763650487	0,800758522
LRRC37A	-0,037660151	3,855800679	0,763979003	0,800858954
TEX261	-0,020434239	7,102677998	0,763981155	0,800858954
ZNF415	-0,044811484	3,206608439	0,765096816	0,801803059
MRPS18C	0,025855667	4,451341119	0,765116953	0,801803059
FAM98B	-0,027261478	4,306209584	0,766191984	0,802766612
LOC93622	-0,02675424	4,321603945	0,76627187	0,802766612
CLASP1	-0,026434297	6,285765508	0,767403764	0,80382892
CCL5	0,071445457	6,660434187	0,767664077	0,80397809
COL12A1	0,09380178	7,686606495	0,768348863	0,804571698
FCF1	-0,019637265	5,998031419	0,769213835	0,805353775
AP3B1	0,022023716	6,337130838	0,769503193	0,805533048
PLEC	0,054221978	9,218363565	0,77015821	0,806094986
GLT25D1	-0,032963881	7,52159574	0,770789934	0,806632375
PAPD7	-0,029366661	5,452315246	0,771602133	0,807358438
RSBN1L	-0,022230283	4,991867886	0,772206102	0,807866433
TRAPPC6B	0,028613991	5,492817374	0,772364807	0,807908516
CDK13	0,022687001	6,041220504	0,773252837	0,808713358
SEC11A	0,034003459	7,449008401	0,773409871	0,808753552
RLIM	-0,020725154	6,532038336	0,773766261	0,809002167
EIF3A	-0,027672569	8,452447721	0,774192689	0,809227061
UGCG	0,040831077	5,043949432	0,774218705	0,809227061
EEF2	0,033647767	11,4388132	0,774382124	0,809273824
DDX26B	0,040362925	3,329088638	0,774974175	0,809768449
SYPL1	-0,026147078	7,611889571	0,775545099	0,81024085
ZNF827	0,041914783	3,512816187	0,777660555	0,812326489
SLC30A9	0,024037868	6,722927369	0,778350173	0,81292232
C1orf52	-0,026835942	4,534928498	0,778612246	0,813071501
SLC25A3	0,020749694	8,743061643	0,779188201	0,813548361
CCDC71	0,023059511	4,981807704	0,779553284	0,813804936
C21orf59	0,027154399	5,756759646	0,77975771	0,813893743
ANAPC10	-0,022780735	3,742980059	0,78017942	0,814209284
RANBP6	-0,029867045	5,234872795	0,781550574	0,815515435
PEG10	0,106294507	7,280488843	0,782360785	0,816235953
PPIP5K2	-0,020384277	5,55990907	0,78261137	0,816372485
NCOA3	-0,026701333	6,667496332	0,784193189	0,817897424
FLNC	0,098585359	5,53660996	0,787218852	0,820927566
CCDC22	-0,01716484	5,056173166	0,787962682	0,821577604
FAM192A	-0,016330865	6,358204742	0,788255319	0,821757074
IFITM3	0,035926395	9,482856754	0,789999132	0,823449111
ARMC7	-0,025901161	4,932132865	0,790130773	0,823460453
ARHGAP4	-0,038141775	5,732879665	0,791345907	0,824600816
RASA1	0,031305987	5,925102214	0,79360684	0,826705663

CCNG1	0,034133764	6,842696501	0,79360834	0,826705663
TMBIM4	0,038102371	7,230320011	0,794329524	0,827330536
QTRTD1	-0,019540627	5,269092826	0,796424232	0,829385589
ABI1	0,018708177	6,763991806	0,796943382	0,8297995
GATAD2A	0,024615059	6,76111745	0,797386337	0,830133959
HIST4H4	-0,033042073	0,860753247	0,798480436	0,831110479
MFAP1	0,021245395	5,576077906	0,798568098	0,831110479
NUP153	-0,024098658	6,470287707	0,799855145	0,832322941
NFKB2	0,034706372	6,384729968	0,801187478	0,833582151
LIN7C	-0,018137097	5,758818088	0,802084011	0,834387624
FTSJD2	-0,017715278	6,277929895	0,802381068	0,834569326
CYP1B1	0,058488755	6,847427242	0,802713717	0,834787988
CELF1	-0,023842371	6,822345738	0,802926887	0,834882348
VCPIP1	0,017706058	5,547798872	0,803464988	0,835314492
CSNK1E	0,023875583	6,979162522	0,805436151	0,83717533
RBM10	0,025888193	6,171479174	0,805500417	0,83717533
TET3	0,035387069	5,443638099	0,805723447	0,837279516
CCNT1	0,038221717	3,398241797	0,805849002	0,837282393
RNF138	0,028592653	5,423005796	0,807066546	0,83841968
JMJD8	0,024788499	6,639575371	0,807491007	0,838732852
PPOX	0,030172079	4,372299261	0,807972432	0,839105089
FAM198B	0,047322756	6,209773045	0,808813681	0,839850844
LOC220729	0,030527201	3,377098879	0,809193345	0,840117147
DOCK5	0,046632628	5,22664291	0,811225258	0,841977713
DCAF7	0,020655869	7,731656784	0,811232377	0,841977713
FBRS	0,021520663	6,699465183	0,811750542	0,8423873
ASH1L	-0,027746788	6,831939928	0,812438013	0,84297243
SCNN1A	0,047400836	8,527180133	0,81259999	0,843012221
GATAD1	-0,015027716	6,05377595	0,813609231	0,843930841
TPT1	0,038693365	12,44527168	0,815023441	0,845269178
PYROXD1	-0,023356391	5,712943749	0,815812071	0,845958411
GPATCH8	-0,020670533	6,314204846	0,815956753	0,845979793
DHX29	-0,022479624	5,820857169	0,816086173	0,845985347
SCAF1	-0,02307119	6,674117133	0,817609453	0,847435608
C2orf18	0,017544515	6,649439421	0,822185216	0,852048782
YEATS2	-0,025214796	6,042763584	0,822998425	0,85276193
USF2	0,02509814	7,154223354	0,824042526	0,853605699
ZNF823	-0,023343715	2,863299943	0,824063106	0,853605699
PIGH	0,019835287	4,545050127	0,825204736	0,854658429
TYK2	-0,017720147	6,416675982	0,827032488	0,856421342
MED23	-0,017888539	5,356882584	0,829988642	0,859352042
ZMAT2	0,014623247	6,823767266	0,830129851	0,859367762
GNG5	0,024251898	7,242691239	0,831182673	0,860280533
PITPNB	0,015741569	6,438552144	0,831263885	0,860280533
RSRC2	0,013769917	6,247003577	0,831475906	0,860369378
LYRM1	-0,027196256	5,442712928	0,831650496	0,860419471
VAV3	-0,038535552	4,117867934	0,832295163	0,860955812
AQR	-0,014129263	5,837812918	0,832523485	0,861061045
CDK3	0,020613843	4,378432239	0,83264944	0,861061045
RPF1	0,019713252	5,420854401	0,834267385	0,862603381

SEPW1	0,038703754	8,631941608	0,834469408	0,862681457
EIF4B	0,020434012	9,423954288	0,835330622	0,863440884
OGFRL1	0,030363133	4,602116903	0,837590202	0,865525495
TESK1	-0,016106101	5,650409459	0,837698391	0,865525495
CNOT1	-0,016338877	7,970120386	0,837728152	0,865525495
SETD7	0,020421711	7,180471714	0,839019961	0,866728846
HOOK2	-0,020465132	5,413360065	0,841227217	0,86887737
RHOU	-0,029655809	6,465433733	0,841373344	0,868896688
LUC7L	-0,020828357	5,386274649	0,841821817	0,869146025
INSR	0,030203593	6,566577368	0,841869702	0,869146025
ATP5S	0,02310656	3,409445416	0,84226332	0,869420766
INTS10	0,022131556	6,22448897	0,843264742	0,870322729
GRIPAP1	-0,021001389	6,190434417	0,843684927	0,870624624
DGCR11	0,024794547	2,045669329	0,844978403	0,871827467
DBNL	0,017620838	7,547181173	0,845457121	0,872189426
SETD5	0,018680368	6,863407857	0,846444891	0,873076344
CUTC	-0,017986191	4,299273806	0,846915531	0,873429674
S100A10	0,037341801	9,633293172	0,84717071	0,873560725
TPM2	-0,029762604	7,174927966	0,848556496	0,874857385
NBEA	-0,035123807	4,32657129	0,849821573	0,876029222
ABCA2	-0,022565854	5,889705397	0,850352395	0,876443921
SNX7	0,023554695	4,921013926	0,851425186	0,877417008
DRAM2	-0,018692367	6,175509941	0,851800439	0,877671078
HNMT	0,036457299	6,54689434	0,852128787	0,877876749
C9orf64	0,013223633	4,962835308	0,852295559	0,877915924
DNAJC15	0,029541573	5,877568701	0,852467102	0,877960001
CLN3	-0,017436618	6,457552243	0,853990841	0,879280319
C2orf69	-0,013408534	4,802842119	0,854006973	0,879280319
ARID1A	0,02286655	7,3896324	0,854774144	0,879937332
EXT1	-0,017790577	6,528633479	0,855396028	0,880444606
SPPL3	0,01398766	6,442652359	0,858792838	0,88380748
MAX	-0,011396013	6,428251584	0,860446876	0,885301022
HINT2	0,019998475	4,962794144	0,860503765	0,885301022
RTTN	-0,020070313	3,666214624	0,860747501	0,885418194
CPSF7	0,011502789	6,808272352	0,86188699	0,886456619
CCDC91	-0,015489938	5,163536699	0,862444891	0,886896654
HIGD1A	-0,023019104	7,302260293	0,862663742	0,886987946
FAM20A	0,040730695	6,192012231	0,864573937	0,888817982
SARS	0,013671945	7,578868095	0,865447137	0,889581553
GDAP2	0,011728896	4,87285955	0,866181912	0,890202631
ESF1	0,01966179	4,653489309	0,867819305	0,891632754
JKAMP	-0,012873689	5,994760964	0,86783496	0,891632754
SPIN4	-0,021373408	3,98184926	0,86897727	0,892543798
SUV420H1	-0,012919214	6,04005423	0,868983469	0,892543798
GNB1	-0,011369001	9,214891852	0,869127241	0,892557027
NGLY1	-0,010310005	5,13655031	0,870414013	0,893573422
GALK2	-0,011862381	4,843254586	0,870463047	0,893573422
TTC17	-0,013162646	6,39129597	0,870510081	0,893573422
ACTR8	0,011692334	4,763616594	0,871329378	0,894279805
ATP5A1	0,017581367	9,031615023	0,871740953	0,894479121

MRPS36	-0,017308504	5,116053726	0,871785927	0,894479121
LRMP	-0,028465782	3,678979765	0,872837755	0,895423597
GLB1	0,013985668	7,055195016	0,873761302	0,896236209
ZNF141	-0,013112979	2,783955586	0,874001285	0,896347535
MARVELD1	0,022926609	6,268479057	0,874237374	0,896454835
MDFIC	0,025219576	6,62756473	0,87492506	0,897025106
CSNK2A2	0,013723128	5,034686392	0,875430236	0,897408115
C7orf26	0,01245468	5,250360095	0,876099667	0,897949814
ZNF571	-0,017227597	2,888240356	0,876307752	0,897949814
RAP1B	-0,019366305	8,051472161	0,87635372	0,897949814
MCFD2	0,013501972	7,782669763	0,877460668	0,898948962
TNPO3	0,015232026	6,066427686	0,878261461	0,899634205
TAF15	0,020296528	7,393039309	0,879426091	0,900604726
PRPSAP1	0,011931729	5,688979158	0,879473071	0,900604726
COG3	0,013501208	5,403297366	0,880241566	0,901256342
ANKRD10	-0,015063484	6,485980537	0,880812905	0,90170593
RNF219	-0,012670819	4,249225698	0,881556556	0,902331756
ATP11B	-0,013523715	6,507010588	0,881717776	0,902361326
RGL2	0,015475088	6,682171462	0,883433215	0,903981256
KIF21A	0,0245217	5,333722006	0,886276384	0,90667964
SLC44A3	0,029429265	5,124640085	0,88633619	0,90667964
ZNF646	0,014629289	5,101992564	0,886692082	0,90690765
TIMM22	-0,010220411	4,697088834	0,886910386	0,906994889
MTERFD2	0,011922801	4,732067557	0,887064867	0,907016843
SDHAP2	-0,01720107	3,779845973	0,887704392	0,90753467
MLH3	0,013185495	4,608178142	0,888235684	0,907941707
HADHB	0,009194968	7,327900017	0,889476201	0,909073473
POGZ	0,017605123	6,8298348	0,891236397	0,91073595
DTNB	0,014762903	4,682297211	0,894054396	0,913478712
RAB7L1	-0,013312722	5,49286977	0,895063009	0,914279378
GSTK1	0,017607586	7,120397806	0,895106192	0,914279378
CBLL1	-0,00905303	5,273345006	0,895556755	0,914602592
GNS	0,018633568	8,909669074	0,895792825	0,914670197
CSF1R	-0,019786655	7,065192841	0,895891222	0,914670197
ORMDL3	-0,013238232	7,235314092	0,896444579	0,915098141
KIAA0753	-0,010750254	4,510467132	0,89667648	0,915197862
TRAPPC1	-0,010317183	6,799416849	0,89729754	0,915694691
PLSCR3	0,01033803	5,574075626	0,897785797	0,916035317
HDDC2	-0,012638705	5,807976328	0,897899994	0,916035317
FSTL1	-0,016230685	8,46368207	0,899126701	0,917146831
FAM76B	-0,010129172	4,225820224	0,8992585	0,917146831
FAM135A	0,015586179	5,035898101	0,900019003	0,917785192
YES1	-0,010870386	6,724347	0,90020136	0,917833893
EEFSEC	-0,008802178	4,775918675	0,901218282	0,918733363
FAM168A	-0,013982386	3,722432973	0,902023812	0,91937512
HS1BP3	0,012790431	5,917935694	0,902117456	0,91937512
TAF8	-0,009154559	5,636397125	0,902659844	0,919790418
VEGFA	0,024423625	8,321713908	0,90301461	0,920014437
PBXIP1	-0,01368503	8,166201721	0,903324245	0,920192415
HPS4	-0,009186582	5,590523584	0,90453519	0,921288343

SQRDL	0,020977933	7,372592136	0,906692666	0,923162163
SEPT2	0,008794836	9,292463281	0,906711165	0,923162163
ZNF780A	-0,009692118	4,31975559	0,906781077	0,923162163
KPNA6	0,008932741	7,090813592	0,907619007	0,923714421
TOX4	0,007954964	6,949723349	0,90770659	0,923714421
CBWD2	-0,007294647	4,656384156	0,907796501	0,923714421
CNN2	-0,011813979	8,426232391	0,907865384	0,923714421
KIAA1704	0,009971133	4,51362133	0,908015235	0,92372906
CAMK2G	-0,009407333	6,111208131	0,908463864	0,924047597
EEF1A1	0,016868261	14,18251182	0,908901498	0,924354856
FYTTD1	-0,008545708	6,650842376	0,90946915	0,924794234
RBM7	-0,010407698	5,631693163	0,909856671	0,925050341
COPS4	-0,008987428	5,717765249	0,910024032	0,925082569
CYB5R4	-0,011795016	5,238929304	0,911468469	0,926412802
DYNLRB1	-0,008567539	7,191659029	0,912416004	0,927237665
TUBGCP4	0,010195174	4,243403947	0,913304191	0,928001979
IPPK	0,011124717	3,979391866	0,913819246	0,928386984
KIAA1468	-0,00915996	5,198337072	0,914734429	0,929178321
CIZ1	-0,008126092	6,763869049	0,916423555	0,930642058
RRP15	-0,007220939	5,406093719	0,916448368	0,930642058
MIB1	0,011511537	6,366818701	0,917146267	0,93121209
TSC2	-0,00850969	6,730661819	0,918512431	0,932460365
RBBP9	-0,008837346	5,734607088	0,919035193	0,932850695
ZFYVE27	0,00878477	5,301916692	0,919170526	0,932850695
GOLGA1	-0,007332478	5,089840411	0,91962692	0,933118811
TUG1	-0,008604408	7,648570528	0,919708392	0,933118811
ACTB	-0,008389184	13,03855225	0,920279209	0,933559049
ATG3	-0,00695184	6,031409679	0,921184466	0,934338372
SPG21	0,00823129	7,186155782	0,921586897	0,934607532
FAM118A	-0,010066577	4,423842968	0,921967625	0,934854607
RELT	-0,009841864	4,196923352	0,923055622	0,935818657
RAD52	0,012917878	2,995252507	0,92366748	0,936299769
ZNF641	-0,009202307	3,648570504	0,925810788	0,938332902
BHLHE40	-0,011750384	8,720719072	0,926099373	0,9384859
UBQLN2	-0,006565148	6,325487132	0,927678636	0,9399466
EPHX1	-0,013144524	8,886363995	0,928640472	0,940781366
YPEL3	-0,009128939	6,328404817	0,92892196	0,940926744
ANGEL2	-0,005387904	5,319116339	0,929823143	0,94169969
WBP11	0,00840901	6,989040509	0,930804165	0,942520924
FUBP3	0,007056332	6,216819113	0,93091046	0,942520924
NDFIP2	-0,008435552	6,233967532	0,931554393	0,943032869
ZNF318	0,0108339	5,401261719	0,932138759	0,943484369
SLC4A2	0,008925039	7,464184163	0,932510161	0,943720212
ZDHHC20	-0,009078229	4,590524452	0,933707414	0,944791639
SREBF1	-0,008557382	7,196743112	0,934579245	0,945533513
SGPP1	0,010268644	5,666634793	0,935115394	0,945935599
SNX16	-0,007263754	3,92475457	0,935664124	0,946350291
KCTD20	-0,005934547	6,794888833	0,938089331	0,948662486
CAMLG	0,008024524	5,802211181	0,938586758	0,949024778
MAPKAPK2	0,007317868	7,93352191	0,938763542	0,949062801

HDGFRP2	-0,005455792	5,843663948	0,9410702	0,951253734
RBPJ	0,009022553	5,950910152	0,942123008	0,952111382
TRAF3	0,006988523	4,435225843	0,942197919	0,952111382
DNTTIP2	0,006860569	6,534348832	0,94261039	0,952387057
MS4A6A	0,018922051	6,594429016	0,94365538	0,953301635
FADS2	0,019651941	6,881672913	0,944522564	0,954036345
C6orf62	0,005404886	7,99389605	0,944834117	0,954209693
FAM175A	0,010195372	4,388155616	0,945671558	0,954857589
TMED5	0,009260898	7,424009391	0,945755706	0,954857589
STRN	-0,006138265	4,452401045	0,94648934	0,955456819
ZNF77	-0,006254216	2,497519774	0,947275409	0,956108794
UPF1	-0,005381321	7,299788939	0,947785633	0,956482201
RSAD1	0,006158452	5,324948465	0,948440703	0,957001651
POLR2L	-0,005825354	7,130497909	0,949840782	0,958145812
METTL10	0,005632701	3,975252003	0,949855652	0,958145812
TNK2	0,009006918	5,82440394	0,951331383	0,959492486
LYAR	-0,005060899	4,624745871	0,951508033	0,95952873
ZNF625	0,010868058	1,086209244	0,951950433	0,959832915
ATF1	0,007329673	5,327300956	0,952096172	0,959837935
NRARP	0,018530155	3,977599899	0,952513666	0,959983519
YBX1	-0,004675374	9,368870669	0,952522142	0,959983519
FAM127C	-0,005300235	4,718817632	0,954536631	0,961871625
HDAC6	-0,005072706	6,184976789	0,955199188	0,962397054
RALB	0,007802543	6,977871764	0,955720309	0,962779848
HERC4	0,003849547	5,938844843	0,956579125	0,963502667
DDX58	0,008503781	5,592052536	0,957355309	0,964105794
TWF2	-0,003723115	6,20621661	0,957460688	0,964105794
CLTB	-0,004855777	6,635087026	0,957925955	0,964431874
DHPS	-0,00349411	5,736844196	0,958200826	0,964566199
ARL6IP4	-0,003706304	7,235039085	0,96054584	0,966778252
ADCY3	-0,003815998	5,863178884	0,960681836	0,966778252
PCBP4	-0,003624256	5,177663641	0,961811401	0,967772163
PKP4	-0,003379158	6,684776551	0,962643292	0,968466304
TUBG2	-0,00290899	4,791900821	0,96585622	0,971555327
PCMTD2	0,005080034	6,262219171	0,966138101	0,971695532
KIAA1919	-0,00284823	3,268966661	0,966841747	0,972259825
DLST	-0,00206602	7,208523011	0,969534568	0,974823978
GNG12	0,007317638	7,60345406	0,969703402	0,974849992
SLC25A44	-0,0023349	5,998563826	0,970693693	0,975701694
NFATC2IP	-0,001898202	5,804233281	0,971200873	0,976067613
ARID4B	-0,001763059	6,492027741	0,972371932	0,977100531
IL13RA1	0,00387359	8,002763439	0,97315511	0,977743435
VTI1B	0,003307914	6,279473067	0,9734608	0,977871325
GBF1	-0,002035394	7,183150697	0,973661054	0,977871325
PLA2G16	-0,001375775	6,493197528	0,973712611	0,977871325
NRP1	0,007003669	8,115791628	0,973909371	0,977924901
TANC2	0,007172446	6,862463628	0,975647596	0,979526054
USP6NL	0,003589466	5,465199733	0,97590951	0,979644773
ERBB2IP	-0,00154801	7,028936179	0,976412461	0,980005383
SMAD5	0,002710635	6,524440591	0,976590851	0,980040179

CASC4	0,003574652	7,668913853	0,977467817	0,980775904
CCDC112	0,003506343	3,665468119	0,978302618	0,981469112
AHCYL1	0,003004408	7,836883249	0,978688746	0,981694432
ARRDC3	-0,000995893	8,254482816	0,978815141	0,981694432
LOC146880	0,007095853	4,977472238	0,979283573	0,982019806
VPS26A	-0,001180212	6,795483008	0,979587117	0,98217976
ORMDL1	-0,001294143	5,616895524	0,980048296	0,982497696
DNAJC5	0,003905869	7,264757547	0,980469798	0,982775769
MAP1S	-0,001161316	6,032156149	0,980890386	0,983052843
SYNRG	-0,000885333	6,558930239	0,982707871	0,984729607
SIPA1L1	-0,000633635	6,278499753	0,983101308	0,98497911
MTSS1L	0,005113435	6,416118184	0,98481462	0,986550741
CXADR	0,006203954	5,187305185	0,987199691	0,988794755
PRPF38A	-0,000540458	5,786456077	0,987516413	0,988912915
MTMR2	0,002104933	5,811241029	0,987607706	0,988912915
USP49	0,005011572	1,111307837	0,988282901	0,989443709
RUNDC1	-0,000219629	5,558833982	0,990429707	0,991447471
CCDC97	-0,000129249	6,008204559	0,991153676	0,992026554
CDK9	0,001448525	6,362828847	0,99158999	0,992317602
EXOSC7	0,001800727	5,225100964	0,99385119	0,994357334
CERK	0,001964155	6,193737539	0,993919869	0,994357334
ANO10	0,00141991	5,49301839	0,9941226	0,99441426
LOC100132832	0,00199093	2,414644934	0,996725437	0,996871628
C11orf58	0,000865566	7,973997205	0,998782391	0,998782391

**Table S1_C: edgeR results**<br>logFC, log fold change; logCPM, log counts per million; p value; FDR, Benjamini-Hochberg adjusted p value are reported for lung squamous cell carcinoma (LUSC) dataset				
Gene	logFC	logCPM	PValue	FDR
CSRNP1	-3,513502506	6,016918944	1,06E-122	7,24E-119
VWF	-3,869389251	8,355253213	9,46E-122	3,22E-118
DLC1	-4,107237839	6,058525597	1,69E-118	3,85E-115
AGER	-6,328595879	7,621821398	3,47E-115	5,92E-112
A2M	-3,888118597	10,34672173	2,50E-106	3,4092E-103
SNRK	-1,85788022	5,633731632	2,5311E-103	2,8766E-100
CD93	-3,430246875	7,000678667	2,7604E-99	2,68905E-96
TNS1	-3,348591018	8,005370368	8,60654E-95	7,336E-92
SYNE1	-3,279746041	5,832261967	5,04931E-93	3,8257E-90
SUSD2	-4,780659314	6,499717361	6,17845E-93	4,21308E-90
SNX30	-2,512376837	5,732696887	1,1594E-92	7,18724E-90
EPAS1	-3,506234166	8,979707633	1,17431E-90	6,67299E-88
SLC39A8	-3,668285792	6,906641741	8,3687E-90	4,3897E-87
GPR146	-2,87861466	2,840381872	8,16586E-89	3,97736E-86
SPTBN1	-2,385173145	8,909092985	1,74199E-88	7,91908E-86
GRK5	-2,831376921	4,853286345	2,55953E-87	1,09084E-84
SNX25	-2,390582742	4,768576763	3,12323E-82	1,25278E-79
EMP2	-2,753048839	8,770107541	3,41278E-82	1,29287E-79
LMO7	-2,939923759	7,250619967	3,83301E-80	1,37565E-77
MRC1	-3,987558834	6,645642982	7,09839E-76	2,4202E-73
KIAA1462	-3,188738491	5,737861545	2,52623E-74	8,20303E-72
ARHGAP31	-2,914340221	5,641606991	6,90993E-70	2,14176E-67
SHROOM4	-3,024709031	4,513631252	1,29626E-69	3,84313E-67
RBMS2	-1,992870256	6,270112688	4,90597E-69	1,35229E-66
KANK2	-2,581223711	6,254690401	4,9578E-69	1,35229E-66
LIMD1	-1,796076641	5,861210461	5,75916E-69	1,51045E-66
KLF2	-3,081081947	4,222317951	4,02386E-68	1,01625E-65
LTA4H	-1,848528083	7,422067755	1,2948E-67	3,15329E-65
GPX3	-3,67841198	8,111621197	1,22302E-66	2,87579E-64
GADD45B	-2,855067568	6,046334491	1,74296E-65	3,96174E-63
SMAD6	-3,033543324	3,198139599	1,05055E-64	2,31087E-62
STX11	-2,785402482	4,532879323	3,13677E-64	6,68425E-62
PLK1	4,158208573	6,042979209	8,73195E-64	1,80434E-61
CCNB2	4,213338084	5,944756686	1,26712E-63	2,54133E-61
ENG	-2,612689411	7,276060561	1,41827E-62	2,7632E-60
SIGLEC11	-2,592971305	1,200401695	1,4953E-62	2,83235E-60
RPS6KA2	-2,78681683	6,102108166	4,54737E-61	8,38067E-59
CDCA5	3,753015546	5,908293553	4,32131E-60	7,75448E-58
C1orf198	-1,691806302	6,792912984	6,83653E-58	1,19534E-55
HYAL2	-1,683257108	6,498144642	5,64746E-57	9,6275E-55
RAPGEF2	-2,075550638	5,110681345	5,81038E-57	9,66365E-55
MYBL2	4,813616271	7,041937145	1,77259E-55	2,87793E-53
PDK4	-4,028611484	5,462531817	4,93407E-55	7,82451E-53
UBE2C	4,594071903	6,366931735	7,3841E-55	1,14437E-52
MAP3K3	-1,634208289	5,50838368	9,95652E-55	1,50874E-52

PTPRM	-2,671121497	5,435187316	5,31046E-54	7,87217E-52
DLGAP5	4,043941074	5,274835205	5,47139E-54	7,93817E-52
LRRK2	-3,811126166	6,351589332	8,11079E-54	1,15224E-51
MYADM	-2,913794492	7,589082194	2,97106E-53	4,13462E-51
CCNB1	3,72959281	6,604023952	5,88866E-53	8,03095E-51
ADARB1	-2,565562421	4,703926812	8,6923E-53	1,16221E-50
AHCYL2	-2,603605721	6,126176345	1,60171E-52	2,1004E-50
C11orf9	-3,518110233	4,440465274	4,09627E-52	5,27027E-50
TTK	4,330302055	5,209224819	4,74201E-52	5,98811E-50
UTRN	-2,346150505	6,834785412	7,65832E-52	9,49492E-50
SERINC1	-1,490295377	7,942235256	1,13836E-51	1,38616E-49
AK1	-2,136018181	5,47525727	1,28951E-51	1,54266E-49
GNAQ	-1,711813819	6,589571282	2,90367E-51	3,41382E-49
CCNA2	3,978544928	5,931568287	1,55716E-50	1,79971E-48
DRAM1	-2,50629352	6,559353595	2,48821E-50	2,82786E-48
DOCK4	-2,314813621	5,045606589	4,67471E-50	5,22571E-48
CYBRD1	-2,369819332	7,436291337	6,92925E-50	7,62105E-48
NR4A3	-3,647414857	4,587213633	1,59379E-49	1,72509E-47
BUB1	3,175151929	5,715340793	7,27413E-49	7,75036E-47
ZFP106	-1,608785459	6,670720343	1,54106E-48	1,61669E-46
CAT	-1,96327542	7,026607273	1,74489E-48	1,80279E-46
SLC9A3R2	-2,310811852	5,841669383	3,86607E-48	3,93474E-46
ARRB1	-2,848223245	4,684270705	2,59941E-47	2,60667E-45
RECK	-2,241082247	3,912546142	2,91099E-47	2,87682E-45
ATP11A	-2,483010401	6,730545219	6,78844E-47	6,58925E-45
ALOX5	-2,92904518	5,620974898	6,86078E-47	6,58925E-45
CALCOCO1	-1,791879686	6,321546557	9,22088E-47	8,73294E-45
ZFP36	-2,733838944	8,23828155	1,01186E-46	9,45191E-45
IL6ST	-2,403923097	5,608459314	1,16198E-46	1,07075E-44
KCTD12	-2,143210441	7,023135088	1,66511E-46	1,51392E-44
AQP1	-3,517225686	8,719980304	5,3009E-46	4,75617E-44
PPP1R15A	-2,084837933	7,059148945	6,09934E-46	5,40148E-44
FAM82A2	-1,372056537	5,902791814	7,85928E-46	6,87083E-44
LATS2	-1,832995018	4,973737422	2,15452E-45	1,85971E-43
FAM105A	-2,600387534	4,851426225	2,19582E-45	1,87166E-43
CFD	-2,841729991	4,780923517	3,45654E-45	2,90989E-43
BTBD9	-1,970711169	4,875301619	3,05243E-44	2,53836E-42
KIF1C	-1,74586627	7,268450738	5,2565E-44	4,31857E-42
HSD17B4	-1,406668052	7,026476278	7,60865E-44	6,17659E-42
FLI1	-2,415342799	4,78615596	8,12825E-44	6,52077E-42
HBB	-4,266512997	6,81720604	9,53621E-44	7,56133E-42
GIMAP6	-2,590436777	5,002363816	2,35055E-43	1,84234E-41
NUSAP1	3,24435213	6,362265393	6,14849E-43	4,76438E-41
BMPR2	-1,694530339	7,133605102	6,93216E-43	5,31128E-41
PNPLA6	-1,680045409	6,446904102	2,77082E-42	2,09936E-40
UBE2T	3,873744217	5,410800761	3,46757E-42	2,59839E-40
EPB41L5	-1,884604132	4,927485382	4,14497E-42	3,07223E-40
RASL12	-2,437623905	3,771775897	4,56666E-42	3,34839E-40
CXCL16	-1,906639162	7,016303535	6,71861E-42	4,87385E-40
CCDC69	-2,412093541	5,75943018	8,28739E-42	5,9486E-40

ZWINT	3,043355744	6,028228502	8,781E-42	6,23726E-40
COLEC12	-2,929446064	5,339178886	2,53823E-41	1,78435E-39
RRM2	3,562615156	7,199606156	3,2538E-41	2,26405E-39
ASAH1	-1,823361163	8,075020935	6,36789E-41	4,38612E-39
MYH10	-2,402161395	7,077617112	9,6111E-41	6,55381E-39
JUND	-1,876210578	7,064293768	1,36075E-40	9,18705E-39
TMEM204	-2,229617429	4,052083377	1,81433E-40	1,21294E-38
CDK1	3,43450866	6,638165292	3,09174E-40	2,04685E-38
FYCO1	-1,805333857	5,623453306	3,90682E-40	2,5616E-38
CD83	-2,332067839	5,429746537	3,9776E-40	2,58317E-38
ZEB2	-2,24688894	5,666273401	4,76265E-40	3,06382E-38
GATA6	-2,677161647	3,84330028	1,42116E-39	9,04364E-38
RACGAP1	2,325540873	6,518872561	1,43234E-39	9,04364E-38
ALOX5AP	-2,836073356	5,686726582	1,49662E-39	9,36279E-38
ZNF331	-2,021463717	5,077793556	1,91201E-39	1,18527E-37
KLF6	-2,11241637	7,786760023	2,53061E-39	1,55462E-37
UBL3	-1,65902175	6,090302597	2,68194E-39	1,63287E-37
SH3D19	-1,698500593	6,288836904	6,10942E-39	3,6402E-37
LAMB2	-2,052436456	7,236718192	6,11851E-39	3,6402E-37
SLC2A1	5,739727405	10,34111936	6,13906E-39	3,6402E-37
TOP2A	3,764332465	7,912336268	1,12801E-38	6,63092E-37
KIAA1524	3,055048289	4,909643702	1,84867E-38	1,07744E-36
KAT2B	-1,73137416	4,590690206	2,30958E-38	1,33466E-36
SNX2	-1,099179699	6,468510668	4,00673E-38	2,29596E-36
NDST1	-1,813212749	7,121459708	5,78757E-38	3,28879E-36
HPCAL1	-1,646967591	6,05382953	6,36591E-38	3,58753E-36
PER1	-2,195037446	6,374412401	1,11117E-37	6,21072E-36
HMGA1	2,806080433	9,043670046	1,17699E-37	6,52511E-36
INPP5K	-1,178364906	5,726040662	1,19556E-37	6,5746E-36
MCM4	2,679496276	7,515171569	1,30548E-37	7,12164E-36
PTTG1	3,091485824	5,763707735	1,53615E-37	8,31351E-36
NEDD9	-2,342922694	7,337060808	2,05978E-37	1,10596E-35
ARHGEF6	-2,139928088	4,953521119	2,43082E-37	1,29498E-35
ADCY9	-1,902255085	5,443904113	4,90751E-37	2,59413E-35
FAM188B	-1,811789251	3,741027675	5,56417E-37	2,91862E-35
SOCS3	-2,221541063	7,922813021	1,229E-36	6,39737E-35
TRAK2	-1,471043421	6,007577803	3,442E-36	1,77811E-34
UHRF1	3,536125251	5,929173186	5,36195E-36	2,74911E-34
HELLS	3,026786819	4,233847149	5,59761E-36	2,84851E-34
ADRB2	-2,989624854	3,306839103	6,48994E-36	3,27814E-34
KPNA2	2,39082152	8,067281057	7,71087E-36	3,86621E-34
ASPM	3,849536368	5,448755045	7,9601E-36	3,96204E-34
NEXN	-2,410152737	3,766174145	8,17507E-36	4,03955E-34
MBIP	-1,751313116	5,316852767	8,36731E-36	4,1048E-34
CD81	-1,234748303	9,258416476	2,13817E-35	1,04144E-33
SPTAN1	-1,520560792	8,199008054	2,77152E-35	1,34035E-33
MCL1	-1,368701417	9,237689181	2,85063E-35	1,3689E-33
DOK2	-2,343857117	4,222893718	3,21123E-35	1,53128E-33
ALDH2	-2,206271983	7,796514035	5,61085E-35	2,65697E-33
TK1	3,445754404	7,227139131	6,00269E-35	2,82292E-33

AKAP12	-2,765536343	5,689831259	6,20066E-35	2,89605E-33
AFAP1L1	-2,143420556	4,271023362	8,85488E-35	4,10758E-33
ZEB1	-1,98012535	4,936031654	1,72966E-34	7,96928E-33
RAB27A	-1,573673656	5,498069831	1,82482E-34	8,35129E-33
HEG1	-2,248228944	7,124484885	4,38128E-34	1,99173E-32
DOCK11	-2,111568365	4,839035068	4,48422E-34	2,02503E-32
RFC4	3,426288798	6,236877135	9,32535E-34	4,18352E-32
GORASP1	-0,984139241	5,516026931	2,20848E-33	9,84291E-32
CD52	-2,709189177	5,731272501	2,56585E-33	1,13614E-31
IFT57	-1,648736504	6,29578025	3,04227E-33	1,3384E-31
DUSP1	-2,623495594	8,254677317	3,14914E-33	1,37654E-31
TSPAN4	-2,089199299	5,3576321	3,40909E-33	1,48067E-31
FANCI	2,424236117	6,666816582	1,10798E-32	4,78185E-31
NR4A2	-2,746580449	4,801334291	1,32626E-32	5,68788E-31
ARRB2	-1,432946378	5,980564078	1,35166E-32	5,76062E-31
DOCK9	-1,846302283	6,598690976	1,63791E-32	6,93722E-31
RASSF3	-1,83607744	3,982556467	1,6725E-32	7,03999E-31
SERPING1	-2,077220242	8,649740888	1,73205E-32	7,2459E-31
FERMT2	-2,041274338	5,774834515	1,84359E-32	7,66551E-31
ANKRD44	-1,695386616	3,100511157	2,17493E-32	8,98839E-31
DNA2	2,858296431	3,973126176	2,82792E-32	1,16166E-30
SPAG5	3,191975311	5,888411194	4,00739E-32	1,63631E-30
WWP2	-1,143679847	6,216840718	4,19395E-32	1,7023E-30
FNIP2	-2,028635552	5,13706035	7,10564E-32	2,86706E-30
JMJD1C	-1,361669229	6,026747022	7,28124E-32	2,92064E-30
UNC13B	-2,076270204	6,304869208	8,27473E-32	3,29973E-30
RPGR	-1,470688415	3,517510828	9,61001E-32	3,80992E-30
ARHGAP11A	2,406562768	5,538482414	1,11227E-31	4,38415E-30
IQSEC1	-1,594674405	6,097150433	2,03866E-31	7,98944E-30
UBE2S	3,009710524	7,123675078	2,08771E-31	8,13491E-30
GINS2	3,771520892	5,212148276	2,15547E-31	8,35122E-30
ZBTB4	-1,408568188	6,873275908	2,47697E-31	9,54263E-30
GNAI2	-1,255647281	8,522819324	3,09622E-31	1,18613E-29
ENPP2	-2,279931157	6,196190576	3,19615E-31	1,21757E-29
FOS	-2,800924047	8,340118924	5,80575E-31	2,19941E-29
MEIS3P1	-1,685932406	4,918188481	7,68314E-31	2,89455E-29
TGFBR3	-2,340557233	5,257064752	8,03035E-31	3,00873E-29
TBX2	-2,230677309	4,898027627	8,98728E-31	3,34887E-29
CENPH	2,348493198	4,535490371	9,83203E-31	3,64373E-29
NR2F1	-2,231085026	4,063656642	1,34023E-30	4,94003E-29
UCK2	2,57382241	5,755225086	1,49446E-30	5,47888E-29
SNX1	-0,968554483	7,208791836	2,3134E-30	8,43588E-29
LDLR	-2,019955813	7,733701569	2,42434E-30	8,79341E-29
NCF2	-2,06895153	5,862504907	2,71905E-30	9,81016E-29
FEN1	2,051609806	6,155554424	3,09008E-30	1,10901E-28
CD33	-2,036520199	2,891644443	3,40583E-30	1,21593E-28
RAE1	1,549297937	6,210945817	3,53953E-30	1,25709E-28
KCTD10	-0,9558103	6,683405972	3,77888E-30	1,33514E-28
SAMHD1	-1,936308358	6,34781144	3,99807E-30	1,39987E-28
PRELP	-2,595269171	6,625482028	4,00315E-30	1,39987E-28

ITGA9	-2,491570717	3,498087141	4,59511E-30	1,59868E-28
PLSCR4	-1,893563757	4,952771809	5,02826E-30	1,74049E-28
ZC3H8	1,96353634	3,819289912	5,17228E-30	1,7813E-28
KNTC1	2,168142336	5,474861666	5,49697E-30	1,88361E-28
VASH1	-1,791485388	5,255552595	6,19073E-30	2,11073E-28
C10orf10	-2,313393035	7,045849828	7,79242E-30	2,64361E-28
APBB1	-1,962955291	4,417793986	1,01602E-29	3,42981E-28
CABLES1	-2,377099124	4,436668329	1,05254E-29	3,53561E-28
RBPMS	-1,881266849	5,519187043	1,4181E-29	4,74019E-28
ARHGAP29	-2,388381652	5,619923456	1,57421E-29	5,23636E-28
ITGA1	-1,880063413	5,656528124	1,61832E-29	5,35696E-28
NES	-2,529240836	6,148092937	1,76219E-29	5,80502E-28
SLCO2A1	-2,929688087	6,133638113	2,06743E-29	6,77779E-28
PTRF	-2,015353726	8,4401163	2,16105E-29	7,0508E-28
SEC22C	-1,154615879	4,744444004	2,20094E-29	7,14676E-28
RNF125	-1,427611933	3,814789858	2,2552E-29	7,28826E-28
SHMT2	1,970110226	7,857247084	2,28134E-29	7,33796E-28
PAG1	-1,879210882	5,081553311	2,81222E-29	9,00307E-28
TJP1	-1,436305094	7,092664305	4,08486E-29	1,30162E-27
DBF4	2,16224231	5,192956755	6,36816E-29	2,01974E-27
DYSF	-2,232389811	5,332123125	6,50037E-29	2,05213E-27
KIAA0101	3,266431607	5,371003602	8,32972E-29	2,61753E-27
PLEKHO2	-1,574768431	6,245417776	1,02978E-28	3,22115E-27
CFL2	-1,635433916	5,018827677	1,08545E-28	3,37976E-27
SSH2	-1,321295037	5,465665501	1,22914E-28	3,80978E-27
ADAMTS1	-2,563828348	6,788818669	1,26705E-28	3,9095E-27
ARHGAP18	-1,883921066	5,676808915	1,36203E-28	4,18363E-27
LRRFIP1	-1,374315786	7,354003629	1,43402E-28	4,38502E-27
C7orf23	-1,535153592	4,596431703	1,71343E-28	5,216E-27
PFKFB2	-1,350981452	5,469601075	2,01482E-28	6,10626E-27
SDCBP	-1,315002579	8,114947121	2,99868E-28	9,04779E-27
C1orf112	2,022635634	4,687626437	3,72675E-28	1,1195E-26
CISH	-1,903554005	4,676911275	4,03264E-28	1,20608E-26
ARID4A	-1,188477885	4,554628021	5,43331E-28	1,61789E-26
SIK2	-1,320811673	5,582750862	5,97254E-28	1,77073E-26
C15orf23	1,976000141	5,29703413	6,45718E-28	1,9056E-26
LILRA6	-1,823781295	3,754259808	6,48335E-28	1,9056E-26
ARHGEF3	-1,444883261	5,567348941	6,74721E-28	1,97464E-26
C12orf49	-1,342671086	5,823227924	7,22448E-28	2,10273E-26
TK2	-1,301708635	5,082388763	7,24654E-28	2,10273E-26
ANO6	-1,284589904	6,960731411	1,01457E-27	2,9315E-26
DAPK1	-2,279510175	5,996217244	1,18175E-27	3,40015E-26
CRIM1	-1,676879591	6,933407737	1,27632E-27	3,65681E-26
MYO9A	-1,513083453	4,322065877	1,31165E-27	3,74231E-26
QSOX1	-1,513687899	7,904367306	1,39843E-27	3,9733E-26
MACF1	-1,857008257	8,117441735	1,54173E-27	4,36226E-26
PARD6B	-1,707047451	4,199879501	1,62497E-27	4,57878E-26
HLA-E	-1,553657462	9,630487362	1,81602E-27	5,09607E-26
GYPC	-2,180966035	4,842918328	2,64167E-27	7,38259E-26
TACC1	-1,954454663	7,470292359	2,72338E-27	7,57989E-26

NOD1	-1,324769784	4,556172161	3,3275E-27	9,22365E-26
RGS5	-2,195542417	6,598468356	3,68663E-27	1,01778E-25
CYP27A1	-2,384797039	6,125733638	3,83711E-27	1,05505E-25
MCM7	2,028296474	8,076950212	3,99412E-27	1,09381E-25
RAB11FIP2	-1,266785758	4,426140331	4,27726E-27	1,16666E-25
UNG	1,678491496	6,63259221	4,29843E-27	1,16777E-25
MRPL3	1,667428029	7,679383417	4,50177E-27	1,21816E-25
HK3	-2,381583039	4,077656238	5,29726E-27	1,42775E-25
MTBP	1,913600702	3,611135589	5,93416E-27	1,59311E-25
MPRIP	-1,395913438	6,709160115	6,34677E-27	1,6972E-25
ARHGEF10	-1,974506871	4,877137042	8,29134E-27	2,20854E-25
DUSP3	-1,052187784	6,808656102	9,93672E-27	2,63652E-25
CD68	-1,850829548	8,52765474	1,15488E-26	3,05237E-25
SLC25A25	-1,753361803	5,583880575	1,21129E-26	3,1891E-25
SORT1	-1,764286419	7,138047101	1,24726E-26	3,27119E-25
CKAP2	1,996528035	6,137751089	1,25798E-26	3,28666E-25
VAMP2	-1,555680143	6,172715548	1,82267E-26	4,74381E-25
HSD17B11	-1,720389449	6,133726714	2,1526E-26	5,58122E-25
SLCO2B1	-2,3727431	6,700544887	2,26608E-26	5,85318E-25
RNASEH2A	2,436646684	5,866803438	2,39462E-26	6,16186E-25
DSP	3,677642672	10,33914664	2,69125E-26	6,89911E-25
KIF18A	2,26058024	4,156497355	2,74316E-26	7,00585E-25
LTBP2	-2,190265004	7,968019739	4,3694E-26	1,10911E-24
DDAH1	-2,130189252	5,555187047	4,37528E-26	1,10911E-24
C2	-2,21728703	6,976321857	4,49793E-26	1,13598E-24
NPC2	-1,712034013	8,686856747	4,64544E-26	1,1689E-24
EMILIN2	-1,725443517	5,33198781	5,24223E-26	1,31422E-24
ACP5	-1,871537287	7,221521722	5,7905E-26	1,44635E-24
USP54	-1,458130631	5,889703821	6,27992E-26	1,56287E-24
STAT5B	-1,091336268	6,243695182	7,6562E-26	1,89401E-24
RAB8B	-1,40342644	5,595033267	7,66605E-26	1,89401E-24
KDM6B	-1,497792283	6,009698248	1,01448E-25	2,49737E-24
ADD1	-1,104526891	7,915280926	1,15149E-25	2,82446E-24
TMEM110	-1,065177637	3,55522451	1,18044E-25	2,88509E-24
SNRPA1	1,604435719	6,106597362	1,50407E-25	3,66294E-24
MFSD2A	-2,259801995	5,407225179	1,54582E-25	3,75123E-24
TGM2	-2,396286439	8,812652887	1,73366E-25	4,19213E-24
SMAD7	-1,763892216	4,85778754	2,17712E-25	5,24585E-24
DKC1	1,604097598	7,339292061	2,19099E-25	5,2607E-24
PLXND1	-1,747317461	7,107088175	2,2884E-25	5,4753E-24
RPP40	2,007960242	3,59903722	2,34272E-25	5,58567E-24
XPC	-1,163240158	5,411104443	2,68471E-25	6,37875E-24
DCP1A	-0,855834953	5,261461299	2,99849E-25	7,09955E-24
ITM2A	-2,290922547	4,738988183	3,54851E-25	8,37277E-24
LOC100129034	-1,480865747	5,93718684	4,86069E-25	1,14293E-23
MYD88	-1,129907239	6,498823558	4,92538E-25	1,15416E-23
CKS1B	2,701055885	7,317168001	4,99833E-25	1,16725E-23
RCC1	1,793131507	5,528905607	5,15445E-25	1,1996E-23
CYR61	-2,282642693	7,663840808	5,26954E-25	1,22221E-23
ACTL6A	2,499101102	7,845912107	5,6774E-25	1,31235E-23

NCAPG2	2,043693953	5,765144085	6,1768E-25	1,42296E-23
NRP1	-1,779757093	6,977330899	6,35139E-25	1,45825E-23
GGA2	-1,128898618	7,180746848	6,47351E-25	1,4813E-23
TIMP3	-2,502815192	9,291230859	6,74215E-25	1,53762E-23
PDCD6IP	-0,882159315	7,331465771	8,37742E-25	1,90419E-23
NUMB	-0,9938256	6,596028413	8,64931E-25	1,95946E-23
MTHFR	-1,342691843	5,255962204	8,69555E-25	1,96341E-23
STOM	-1,556625966	8,646505699	1,17284E-24	2,63946E-23
CARD8	-1,3054494	4,918217939	1,33441E-24	2,99321E-23
SPATS2	1,188345044	5,988837421	1,47453E-24	3,29666E-23
C16orf88	1,480223595	4,958534282	1,68193E-24	3,74807E-23
GAPDH	2,450211071	12,92261663	1,76102E-24	3,91154E-23
CSE1L	1,398946188	7,85645868	1,78751E-24	3,95748E-23
KIF13B	-1,862694777	5,220582633	1,79549E-24	3,96227E-23
PLCB2	-1,843060852	4,417996009	1,96863E-24	4,33036E-23
C5orf34	2,752569937	2,925384396	2,14597E-24	4,70526E-23
SGOL2	2,053162874	4,398053708	2,15914E-24	4,71896E-23
LTBP4	-2,137478556	7,303645319	2,88905E-24	6,29407E-23
HOXA5	-1,972002878	2,472591967	3,05364E-24	6,63147E-23
PCNA	1,884684427	7,842127897	3,31896E-24	7,18475E-23
ARRDC2	-1,391254584	5,174623808	3,9252E-24	8,47023E-23
DENR	1,000098049	7,056328233	4,2895E-24	9,22716E-23
ARHGEF17	-1,686846304	5,666196009	4,41309E-24	9,46315E-23
FANCD2	1,649308835	5,135535532	4,77968E-24	1,02171E-22
POC1A	1,863437088	3,899770749	4,90548E-24	1,04533E-22
USP53	-1,307746324	5,277923278	4,92125E-24	1,04542E-22
RAB11FIP1	-2,087034469	7,893090503	5,55162E-24	1,17567E-22
ANKS1A	-1,267427033	6,008243108	5,73269E-24	1,21025E-22
LAIR1	-1,648826288	5,738776072	6,10079E-24	1,28399E-22
CARD16	-1,525655087	3,849110754	7,33772E-24	1,53957E-22
RANBP1	1,8850205	7,410742073	1,12908E-23	2,36172E-22
PTPRG	-1,736532765	5,047266199	1,14087E-23	2,37907E-22
DNAJC9	1,406190085	6,163331797	1,21823E-23	2,53267E-22
PREX1	-1,858800102	6,051539286	1,34132E-23	2,78008E-22
CCL2	-2,525292349	6,367237675	1,44372E-23	2,98325E-22
COL13A1	-2,220504459	2,403051382	1,50827E-23	3,10721E-22
QKI	-1,161924264	7,238896952	1,67814E-23	3,44676E-22
HIP1	-1,513799701	6,045564768	1,75404E-23	3,59182E-22
KIAA0494	-1,022632211	7,546728787	1,92438E-23	3,92885E-22
AGTPBP1	-1,201641956	4,782468472	2,03997E-23	4,1524E-22
S100A4	-2,029345611	7,131466648	2,16007E-23	4,38378E-22
STK10	-1,461792449	5,687515272	2,2231E-23	4,49832E-22
MYO5C	-1,858018204	5,09225341	2,38057E-23	4,8027E-22
PRKAR1A	-0,993837183	8,452723983	2,5299E-23	5,0889E-22
MYO19	1,73118891	6,211653188	2,71128E-23	5,43771E-22
ITGAL	-2,107334308	5,434740656	3,58942E-23	7,17778E-22
EGR1	-2,253865259	8,404047884	3,87426E-23	7,72473E-22
SLC6A8	4,150287139	8,616694771	4,49712E-23	8,94049E-22
CPEB4	-1,362314949	5,374944285	4,60352E-23	9,12541E-22
SPARCL1	-2,308627332	8,504167785	4,7428E-23	9,37425E-22

AHNAK	-2,038379646	10,33877028	4,84615E-23	9,55083E-22
VIM	-1,924594743	10,12752778	5,44993E-23	1,07098E-21
SASS6	1,552003961	3,626335388	5,95784E-23	1,16743E-21
PMP22	-1,718699102	6,68080021	6,09402E-23	1,19069E-21
FOXP1	-1,385673067	5,938625271	7,98586E-23	1,55587E-21
H2AFX	2,086833214	6,397815588	8,46281E-23	1,6441E-21
PPP1R14B	1,941465989	7,515794651	1,01898E-22	1,97398E-21
PAICS	2,020328953	7,970228179	1,09012E-22	2,10581E-21
RHOA	-0,86072069	9,38448038	1,45294E-22	2,79875E-21
TBRG4	1,485330095	6,705114838	1,55E-22	2,9773E-21
WDR75	1,184967208	6,217922015	1,68635E-22	3,23011E-21
SACM1L	-0,907780341	5,845490778	1,71907E-22	3,28356E-21
SYNM	-1,987822006	4,855663262	1,87131E-22	3,56438E-21
GGCT	1,829045091	6,542113875	2,33065E-22	4,42693E-21
POLR2H	2,217067735	6,905201778	2,39107E-22	4,52908E-21
SLC7A7	-1,9882118	5,490637239	2,48717E-22	4,69806E-21
WDR12	1,641125501	5,571302814	2,51376E-22	4,73517E-21
RYBP	-0,997197462	5,929838789	2,7529E-22	5,17137E-21
C10orf54	-1,685617652	6,266026047	2,8339E-22	5,3089E-21
XRCC2	1,96742872	3,538248403	3,54594E-22	6,62459E-21
KCNAB2	-1,57356787	4,855265148	3,86221E-22	7,19574E-21
BRI3BP	1,833887717	3,662143744	3,9903E-22	7,41414E-21
PJA2	-1,094941039	7,202805987	4,81719E-22	8,9262E-21
NICN1	-1,112185459	3,847528619	5,07014E-22	9,36945E-21
TYMS	2,462129537	6,127632095	5,09363E-22	9,38742E-21
FLAD1	1,637621618	6,458892714	5,21607E-22	9,58716E-21
MMP11	5,628721572	7,826560855	6,26706E-22	1,14879E-20
TSC22D3	-1,789700125	7,728692346	7,34453E-22	1,34269E-20
ZFP64	1,421321921	5,600423607	7,72283E-22	1,40807E-20
CYP4V2	-1,234149708	4,888752188	1,13382E-21	2,06175E-20
TRIB1	-1,795301923	6,68316538	1,13956E-21	2,06667E-20
BTG2	-1,589572724	7,762768007	1,29448E-21	2,34139E-20
TMEM2	-1,698870922	6,721749843	1,41993E-21	2,56151E-20
PTGIS	-2,072964161	5,306552488	1,82646E-21	3,28618E-20
TMEM69	1,136823973	5,492989595	1,95891E-21	3,5152E-20
FIGNL1	1,881102136	5,114655739	1,97604E-21	3,53665E-20
EPS15	-0,79608436	6,549614988	2,04068E-21	3,64278E-20
CCT3	1,319273694	8,942842808	2,1958E-21	3,90944E-20
CCDC58	2,248798278	5,017363139	2,30734E-21	4,09733E-20
RAPGEF6	-1,253338223	3,865312477	2,3965E-21	4,23362E-20
RBMS3	-1,924747249	2,296603136	2,3965E-21	4,23362E-20
DOCK2	-2,043706367	5,282882078	2,42222E-21	4,268E-20
CENPN	1,734090887	5,642612907	2,48996E-21	4,37604E-20
GMPS	1,830079022	7,605212224	2,63481E-21	4,61871E-20
SRPK1	1,381393651	6,965414775	2,91072E-21	5,08928E-20
CCDC86	1,358709878	5,805131962	2,93389E-21	5,11668E-20
ZNF438	-1,127036986	3,707852703	3,05266E-21	5,31023E-20
FAM190B	-0,965034425	6,006948683	3,07812E-21	5,34089E-20
CHTF18	2,524698436	4,617306442	4,27442E-21	7,39779E-20
PCYOX1	-1,151837813	7,452116724	5,06699E-21	8,74729E-20

ALAS1	-0,917135675	6,28051487	5,21935E-21	8,98756E-20
NPM3	2,42723002	5,827574216	5,3047E-21	9,11153E-20
ARAP3	-1,596355503	4,659298713	5,7138E-21	9,78489E-20
FCHO2	-1,27232005	5,54632683	5,72543E-21	9,78489E-20
FEM1C	-1,112771896	5,417199061	6,5994E-21	1,12503E-19
APBB2	-1,48924222	5,641549776	6,90051E-21	1,17343E-19
THOC3	2,328176522	6,671983495	7,02589E-21	1,19178E-19
RFC2	1,365784496	6,178168024	7,36838E-21	1,24677E-19
FKBP4	2,07250939	8,398377513	8,06736E-21	1,36167E-19
ANKRD28	-1,037579118	5,808645844	8,10298E-21	1,3643E-19
FCGRT	-1,441267368	7,206420081	8,72714E-21	1,46577E-19
APLP2	-1,250001855	9,95595263	8,83627E-21	1,48046E-19
C1orf131	1,528808383	4,63016242	9,21949E-21	1,54088E-19
CGN	-2,159490499	5,805833375	1,01164E-20	1,68664E-19
TXNIP	-1,823830572	9,242565084	1,20528E-20	2,00459E-19
KIAA0355	-1,117239996	5,432216435	1,25967E-20	2,08994E-19
PDE4D	-1,623804833	5,285693601	1,34642E-20	2,22846E-19
VPS13C	-1,17863819	6,182635552	1,45048E-20	2,39487E-19
MYO1F	-1,815072194	5,109338894	1,53896E-20	2,53483E-19
CTSH	-1,996046811	8,535169575	1,56304E-20	2,5645E-19
ZNFX1	-1,275408394	6,739991877	1,5645E-20	2,5645E-19
UACA	-1,334142228	5,87796367	1,60592E-20	2,62608E-19
CREBL2	-1,02695768	6,313327588	1,64238E-20	2,67928E-19
RSRC1	1,59137152	5,562409465	1,71398E-20	2,78941E-19
TNS3	-1,851811215	7,24703534	1,75062E-20	2,84226E-19
AFF1	-1,253802682	6,382297802	1,79953E-20	2,91473E-19
RGL1	-1,547056183	5,625221215	1,80408E-20	2,91516E-19
FAM13B	-1,215248245	4,962914723	1,88573E-20	3,03991E-19
LMCD1	-1,628030953	5,497687823	1,89162E-20	3,0422E-19
MCM6	1,591355419	6,817003342	1,93886E-20	3,11085E-19
CCT5	1,728797149	9,239253456	2,00954E-20	3,21668E-19
MLLT4	-1,173421983	6,795772545	2,22116E-20	3,5471E-19
PGAM5	1,500213217	5,918608272	2,61944E-20	4,17336E-19
RUVBL1	1,516971306	7,010928255	2,8303E-20	4,4988E-19
RIN3	-1,54309911	5,205595734	2,86155E-20	4,53789E-19
TPI1	1,681836792	10,50058162	2,86959E-20	4,53789E-19
PYCR1	2,844953856	7,076569857	2,87486E-20	4,53789E-19
TLN1	-1,523013796	8,847815948	3,16631E-20	4,98639E-19
LAMA4	-1,861131267	6,414449345	3,26811E-20	5,13484E-19
IP6K1	-0,759332438	5,94948425	3,32199E-20	5,20751E-19
IFITM2	-1,751384214	7,202283803	3,556E-20	5,56155E-19
POP7	1,493545144	5,578092025	3,6168E-20	5,64369E-19
PDK1	2,201559408	6,384745318	3,96679E-20	6,1757E-19
DTL	2,195110942	5,451443023	4,10963E-20	6,38351E-19
NUP155	1,687610589	6,270915478	4,3964E-20	6,81342E-19
DENND5A	-1,148992328	6,533762562	5,79331E-20	8,95795E-19
EZH1	-1,098345595	5,042575425	6,30371E-20	9,72487E-19
SEC14L1	-1,291043749	7,214199313	6,31781E-20	9,72487E-19
KRT17	6,872941924	11,78115879	6,79453E-20	1,04351E-18
DCAF13	1,426683884	6,521896342	6,97378E-20	1,06863E-18

KIAA1109	-1,327098283	5,802527267	7,69243E-20	1,17611E-18
HERC1	-1,204264798	6,019212967	8,33212E-20	1,27107E-18
SNHG1	2,210252013	6,2244366	8,61418E-20	1,31116E-18
IL1R1	-1,622005648	7,237688667	8,73176E-20	1,3261E-18
TPP1	-1,151648202	8,109526106	8,76038E-20	1,32749E-18
MXD4	-1,196332248	6,149969774	9,02547E-20	1,36463E-18
PPM1D	-1,010955158	4,379427746	1,01303E-19	1,52828E-18
PIK3R5	-1,991359512	4,133444141	1,01594E-19	1,52929E-18
ARAP1	-1,128782077	7,218519783	1,01992E-19	1,5319E-18
AKAP11	-0,97834172	5,982009632	1,02598E-19	1,53762E-18
HIPK1	-1,106172188	6,676328771	1,06472E-19	1,59218E-18
MTMR14	-0,881731381	5,588090155	1,06758E-19	1,59296E-18
CYBB	-2,115923356	7,011246435	1,17072E-19	1,74305E-18
NIPSNAP1	1,602450083	7,340442353	1,1799E-19	1,75288E-18
ENPP4	-2,026729618	4,901784858	1,24354E-19	1,84341E-18
NUP37	1,406341884	5,373650765	1,25967E-19	1,86128E-18
MRPL47	1,763813146	6,526212029	1,26105E-19	1,86128E-18
TLR4	-2,037467884	5,090450139	1,33994E-19	1,97345E-18
MTIF2	1,158962726	6,069552442	1,6105E-19	2,36681E-18
USP12	-1,067775699	4,428873999	1,84336E-19	2,7032E-18
NBEAL1	-1,449094088	3,268202231	1,86409E-19	2,72773E-18
DAB2	-1,783963899	6,482333339	1,87314E-19	2,73511E-18
GAS6	-1,686405096	7,143294759	1,88601E-19	2,74801E-18
PLEKHM3	-1,14678511	4,671988387	2,45198E-19	3,56504E-18
ZMYND19	1,517561784	4,942639434	2,50578E-19	3,63552E-18
LOC728554	2,350426632	6,012439118	2,63206E-19	3,81061E-18
NFKBIA	-1,58887248	8,078037709	2,83653E-19	4,09794E-18
CSF1	-1,790526859	5,297796342	2,95842E-19	4,265E-18
IL7R	-2,309312169	4,862083317	3,22403E-19	4,63812E-18
ZCCHC24	-1,719476981	5,042721987	3,41458E-19	4,90189E-18
BRIX1	1,733402357	6,568159565	3,55176E-19	5,08813E-18
RCC2	1,445218363	8,481229053	3,60926E-19	5,15966E-18
PFN2	3,449006055	8,849431192	3,7252E-19	5,31425E-18
NR3C1	-1,115638911	6,681637889	3,88577E-19	5,53175E-18
JAK1	-1,08422155	7,707631589	4,37982E-19	6,22208E-18
MYL9	-1,807358691	7,90326457	4,52095E-19	6,40923E-18
BCL2L12	1,582811274	4,827696226	4,6381E-19	6,56167E-18
SAP30L	-1,048219928	5,313309209	4,89206E-19	6,90661E-18
BOP1	1,924665092	5,797438281	4,95313E-19	6,97839E-18
DTYMK	1,905993087	5,726388846	4,98773E-19	7,00304E-18
SLC15A3	-1,838607788	5,860364439	4,99117E-19	7,00304E-18
TMEM48	1,402414226	5,191103624	5,2586E-19	7,36312E-18
XPOT	1,552489535	7,880474877	5,77547E-19	8,07027E-18
ERGIC1	-0,92726552	7,62118152	5,81056E-19	8,1027E-18
FHOD1	-1,422018389	5,134697842	5,89618E-19	8,20532E-18
PRPF19	0,981351349	7,832306436	5,91695E-19	8,21745E-18
TTC28	-1,646369073	5,158179397	6,49402E-19	9,00056E-18
SLC25A30	-1,008202457	4,364928909	6,62673E-19	9,16585E-18
SAMD4A	-1,841952854	4,713282645	6,87736E-19	9,49326E-18
DSG2	2,256301679	8,942952154	7,03496E-19	9,6912E-18

RASIP1	-2,25414171	4,198757223	7,11692E-19	9,78433E-18
CAV2	-1,690606825	7,395538426	8,79326E-19	1,20646E-17
C11orf84	1,344410083	5,557148499	8,86205E-19	1,21346E-17
HN1	1,748952917	7,708165256	9,92607E-19	1,35643E-17
MEMO1	1,367579687	6,228187863	1,01461E-18	1,38373E-17
B3GALNT2	1,166550688	6,185519225	1,06606E-18	1,45099E-17
LOC440173	4,180782512	3,603173405	1,08542E-18	1,4744E-17
CYB5A	-1,765251413	6,906982443	1,09927E-18	1,49025E-17
EXOSC5	1,874902192	5,285696205	1,11726E-18	1,51163E-17
CAMTA2	-1,041096039	5,601072511	1,12233E-18	1,51548E-17
TUBA1C	1,409121967	9,75386402	1,17497E-18	1,58342E-17
DCUN1D5	1,724399603	5,928319665	1,31112E-18	1,76342E-17
TARS	1,465507529	7,809401093	1,48035E-18	1,98406E-17
ARNTL2	3,371400124	5,594127182	1,48099E-18	1,98406E-17
TRAP1	1,395280063	7,19670118	1,53234E-18	2,04883E-17
ANKRD13A	-0,835012354	6,231997865	1,5631E-18	2,08587E-17
PTPN1	-0,94225342	7,129200898	1,60432E-18	2,13669E-17
LPCAT1	-2,207259604	8,190102186	1,72248E-18	2,28959E-17
MRPL36	1,777474055	5,586267996	1,73263E-18	2,2986E-17
B4GALT2	1,292728369	6,53773227	1,88182E-18	2,49168E-17
SYNJ1	-1,137849755	4,084507453	1,89169E-18	2,49989E-17
ARHGEF2	-1,17919192	6,526001389	1,986E-18	2,61945E-17
SLC16A1	2,494645645	7,2359965	2,33432E-18	3,07291E-17
CSRP1	-1,255730201	8,338569495	2,4136E-18	3,17116E-17
HSPE1	1,740443228	7,647859855	2,56833E-18	3,36797E-17
YWHAZ	1,122015206	11,050303	2,67739E-18	3,50424E-17
HPRT1	1,641281788	6,408543955	2,95917E-18	3,86563E-17
ITSN2	-0,918339255	6,127292747	3,06253E-18	3,993E-17
ALDH18A1	1,06724831	7,053889133	3,07733E-18	4,00464E-17
PROS1	-1,799055493	6,077859582	3,816E-18	4,95644E-17
LY75	-1,642802122	6,2000037	3,87459E-18	5,02297E-17
TFB2M	1,403230267	5,227004398	4,00673E-18	5,18442E-17
HLA-DPB1	-1,841991779	8,502449555	4,97195E-18	6,42116E-17
NCAPD2	1,879539495	7,827207471	5,14551E-18	6,63275E-17
OLA1	1,451538047	7,492294565	5,91927E-18	7,61575E-17
CFLAR	-1,141100032	6,627368324	6,75403E-18	8,6734E-17
MAP4	-1,093108873	8,212924658	7,25721E-18	9,30205E-17
SNRPD1	1,411327201	6,555044289	7,95542E-18	1,01779E-16
MRPL30	1,089570702	6,303176344	8,07728E-18	1,03144E-16
DCUN1D3	-1,20275671	3,910842811	8,41183E-18	1,07215E-16
MOAP1	-1,014662779	5,269832858	8,58625E-18	1,09234E-16
GAS7	-1,75353323	5,597187332	9,19447E-18	1,16754E-16
ANKFY1	-0,991579199	6,426130566	9,26867E-18	1,17478E-16
PNPLA2	-1,282138997	5,880216279	9,29714E-18	1,1762E-16
STXBP1	-1,682423607	5,712009675	9,55264E-18	1,20629E-16
SORD	2,136405604	6,920124656	9,73425E-18	1,22695E-16
GIT2	-0,771684955	5,893827289	1,12939E-17	1,4209E-16
HLA-DMA	-1,771607374	6,896457905	1,19457E-17	1,50015E-16
EZR	-1,100619857	9,135647768	1,23026E-17	1,54212E-16
PTPLAD2	-1,592907202	4,644050744	1,31394E-17	1,64399E-16

HLX	-1,543227006	3,369231434	1,47005E-17	1,83594E-16
HSPD1	1,462115898	9,354672705	1,67452E-17	2,08749E-16
CCDC138	1,975577019	3,217201297	1,85133E-17	2,30368E-16
LRP1	-1,642988189	8,994439492	1,89135E-17	2,3492E-16
PSMD14	1,304381492	7,290705052	2,02016E-17	2,50463E-16
RNF130	-0,958092066	6,62131012	2,12234E-17	2,62654E-16
SNRPE	1,401117686	6,644685606	2,23739E-17	2,76391E-16
LIN9	1,652554523	4,256223966	2,33431E-17	2,87842E-16
CTNNA1	-0,783075982	8,877618676	2,35824E-17	2,90268E-16
DCHS1	-1,842352458	5,171458639	2,44132E-17	2,99953E-16
CD74	-1,799685661	11,66125554	2,47407E-17	3,03429E-16
PALB2	1,062653212	4,797666094	3,21642E-17	3,93766E-16
ILF2	1,226981074	8,688043268	3,33115E-17	4,0708E-16
LYSMD3	-1,087577919	4,908583088	3,69427E-17	4,50648E-16
ACAA1	-0,926280884	5,785269366	3,74368E-17	4,55859E-16
HDGF	1,122157466	9,236981894	3,84206E-17	4,67005E-16
EHD1	-1,32583255	6,473577986	4,09107E-17	4,96388E-16
LPIN2	-1,530355299	5,89198462	4,10095E-17	4,96703E-16
MTX2	1,099037124	5,890826621	4,14734E-17	5,01431E-16
INCENP	1,54567322	5,428131032	4,21736E-17	5,08994E-16
TDP1	1,072901386	4,894505479	4,25758E-17	5,12941E-16
TWF1	1,305507526	7,838873527	4,39574E-17	5,28652E-16
AHCY	1,771462217	8,853384161	4,51866E-17	5,42478E-16
SH3BGRL	-1,312367196	7,076955632	4,5301E-17	5,42896E-16
RHOB	-1,64189341	8,02011022	4,7771E-17	5,71492E-16
KIAA0232	-1,060759385	5,693037765	5,07403E-17	6,05951E-16
SLC16A4	-1,875985172	3,692932265	5,13969E-17	6,12719E-16
INTS8	1,152764355	6,077410275	5,16325E-17	6,14453E-16
BZW2	1,535844024	7,451802897	5,20519E-17	6,18366E-16
SHFM1	1,507571469	7,295398305	5,32443E-17	6,31431E-16
MTERFD1	1,292898038	5,124186675	5,81993E-17	6,88995E-16
MTHFD2	2,056974107	7,671718579	6,04055E-17	7,13874E-16
SRD5A1	2,278253663	5,312771148	6,06344E-17	7,14914E-16
TOMM40	1,275905527	7,014044056	6,07104E-17	7,14914E-16
TDG	1,218679017	6,254786962	6,08081E-17	7,14914E-16
GGH	2,81448426	6,533456727	6,12249E-17	7,18576E-16
PPIF	1,704394924	7,447343881	6,20371E-17	7,249E-16
PAK1	1,728586451	7,552992511	6,20664E-17	7,249E-16
TH1L	1,265675474	7,217204724	6,20827E-17	7,249E-16
TSPAN3	-0,982033007	8,270384415	6,21899E-17	7,24912E-16
ACSL4	-1,485460442	7,096387058	6,59161E-17	7,67034E-16
CHAF1A	1,94756831	5,511694668	6,6282E-17	7,69977E-16
UMPS	1,281134413	6,142340549	6,69787E-17	7,76748E-16
EIF4EBP1	3,024157642	7,171131266	6,95677E-17	8,05402E-16
MRPL12	1,871911636	6,278521833	7,03664E-17	8,13268E-16
PRMT3	1,5156908	5,117955267	7,18506E-17	8,29017E-16
VRK1	1,489325504	5,009601488	7,25438E-17	8,35602E-16
SIAH2	1,968065271	7,013393298	7,33997E-17	8,44034E-16
NINJ2	-1,912829891	3,472754128	7,4616E-17	8,56577E-16
JUP	2,011678719	10,80244363	7,77999E-17	8,91626E-16

PRKCD	-1,032481173	6,233627196	8,14099E-17	9,31433E-16
ARHGDIB	-1,469629124	7,662212508	8,45714E-17	9,65984E-16
MCAM	-1,52524477	7,037774973	8,56153E-17	9,76272E-16
RAN	1,338001078	9,232354916	8,84546E-17	1,00697E-15
PKD2	-1,065213124	5,356392211	8,91506E-17	1,0132E-15
HN1L	1,161138725	7,82537502	9,13195E-17	1,03607E-15
PA2G4P4	1,1823227	0,885863689	9,14674E-17	1,03607E-15
MARS	1,01286256	7,677531128	9,56091E-17	1,08119E-15
THBS1	-1,862059201	9,023065429	9,89519E-17	1,11714E-15
SLC43A2	-1,340362312	5,5201605	1,02591E-16	1,15631E-15
SSX2IP	1,474148529	5,395105532	1,03102E-16	1,16016E-15
FAM136A	1,307079966	6,131937964	1,08963E-16	1,22409E-15
POLD2	1,248712057	7,449179207	1,09474E-16	1,22781E-15
LRRC8C	-1,276138709	5,129986972	1,11005E-16	1,24293E-15
FRYL	-1,047882113	5,852358351	1,12438E-16	1,25691E-15
DOCK6	-1,134868861	5,830751095	1,14398E-16	1,27673E-15
LRFN4	1,829329497	4,832306373	1,20315E-16	1,34057E-15
DUSP6	-1,881578208	7,241903708	1,24469E-16	1,38459E-15
MPEG1	-1,759177861	5,924719764	1,26131E-16	1,4008E-15
TMEM170B	-1,502233889	3,578247403	1,27227E-16	1,41067E-15
APC	-1,102819119	5,213479541	1,31279E-16	1,45199E-15
GNS	-0,998176756	8,238652818	1,31379E-16	1,45199E-15
ALAD	-0,912777785	5,740729623	1,39848E-16	1,54308E-15
DDX11	1,844975138	5,572976502	1,47959E-16	1,62994E-15
ITGA7	-1,701026201	3,203371275	1,74454E-16	1,91871E-15
SNRPB	1,590876032	8,390514482	2,02966E-16	2,2287E-15
PINK1	-1,057842403	6,09700749	2,08345E-16	2,28409E-15
RMI1	1,574983742	4,925019583	2,33402E-16	2,55159E-15
MDH2	1,002194439	8,435538301	2,33494E-16	2,55159E-15
LMNB2	1,752210421	7,501977397	2,41096E-16	2,63046E-15
TAOK3	-1,027680088	5,541030099	2,43515E-16	2,6526E-15
TMTC3	1,618636952	6,813746974	2,44117E-16	2,65492E-15
PPP1R15B	-0,806693867	7,079633429	2,52983E-16	2,74696E-15
BDH2	-1,191249137	4,837069633	2,53924E-16	2,7528E-15
RALGAPA2	-1,610141462	4,965673341	2,63784E-16	2,85514E-15
TOPBP1	1,426315992	6,689032485	2,6625E-16	2,87727E-15
FANCE	1,881223187	5,119907705	2,86937E-16	3,09592E-15
MRPS30	1,499728951	5,730372448	2,96611E-16	3,19525E-15
TCP11L2	-1,11889667	3,737328168	2,99753E-16	3,224E-15
MCM5	1,532478586	7,193248331	3,0137E-16	3,23629E-15
SLC25A39	1,304071681	7,930181596	3,12365E-16	3,34908E-15
BUB3	1,0646638	7,150261577	3,14919E-16	3,37117E-15
TFPI	-2,047457197	6,913669077	3,19499E-16	3,41484E-15
XRCC3	1,769268848	5,074989464	3,20767E-16	3,42302E-15
CLCN2	2,369795297	4,138149269	3,39991E-16	3,6225E-15
S100A2	6,550335075	9,796299156	3,48478E-16	3,70713E-15
EHD2	-1,754470255	7,525529342	3,65021E-16	3,87707E-15
CRISPLD2	-1,774764686	6,562907009	3,82477E-16	4,05616E-15
SORBS3	-1,095975637	6,718505677	3,89202E-16	4,12107E-15
ASNS	2,556448023	6,703605401	3,90364E-16	4,12696E-15

CCT7	1,014851055	8,780201006	4,04112E-16	4,2657E-15
MRPL37	1,062688954	6,936558467	4,16322E-16	4,38779E-15
BOLA3	1,673839995	4,930190861	4,2476E-16	4,46981E-15
SH3RF1	-1,094079085	5,378559301	4,41515E-16	4,63897E-15
HDAC2	1,162254946	7,634109665	4,75347E-16	4,98675E-15
RFC3	1,482784011	4,989148504	4,7852E-16	5,01233E-15
ZZEF1	-1,078493115	5,957070423	4,83641E-16	5,0582E-15
RCAN1	-1,46517221	6,247127006	4,92444E-16	5,14238E-15
ABCC5	3,627877347	8,555494434	5,15654E-16	5,37652E-15
GRN	-1,122059245	9,55818006	5,18992E-16	5,40307E-15
PDCD5	1,54846507	6,429902586	5,23801E-16	5,44482E-15
STAT5A	-1,340289874	5,415564982	5,65394E-16	5,86822E-15
MSN	-1,29997198	9,817723308	6,25026E-16	6,47728E-15
ZNF692	1,656199514	4,711738996	6,56474E-16	6,79286E-15
SMUG1	1,188674523	5,558612159	6,74478E-16	6,96859E-15
NOL11	1,049836041	6,582520901	6,86851E-16	7,08568E-15
METAP1	1,259102991	6,973813103	7,04882E-16	7,26071E-15
C1QA	-1,856637978	7,937947394	7,61795E-16	7,83511E-15
LMNB1	1,683625865	6,37328149	7,92033E-16	8,13102E-15
DNAJB11	1,482055099	7,610326382	7,9295E-16	8,13102E-15
TMEM164	-1,199299657	5,721505977	8,09394E-16	8,28717E-15
NOL10	0,994219212	5,954144852	8,44019E-16	8,62874E-15
TUBG1	1,205815756	6,241071442	8,65021E-16	8,81734E-15
SKI	-1,107387187	6,871190443	8,65053E-16	8,81734E-15
CTSD	-1,281572999	10,62349264	8,8196E-16	8,97624E-15
ANXA6	-1,535066681	7,143814257	9,25912E-16	9,40953E-15
ANAPC7	1,03255754	6,665269144	9,35333E-16	9,49113E-15
TAOK2	-1,002946961	6,121461338	9,49873E-16	9,62435E-15
CTGF	-1,837695181	7,980010826	9,77379E-16	9,88835E-15
MYO1B	-1,263003626	7,693044264	1,01785E-15	1,02826E-14
SLC44A2	-1,194546076	7,960484234	1,08282E-15	1,09227E-14
CRLS1	-1,017302109	6,15979291	1,10049E-15	1,10846E-14
GCC2	-0,869100983	5,368276049	1,10428E-15	1,11064E-14
DHCR24	-1,357931517	9,78707391	1,14527E-15	1,15016E-14
NR2C2AP	1,586718666	5,320896725	1,17631E-15	1,1796E-14
LNX2	-1,020888064	5,555845898	1,17884E-15	1,1804E-14
TOM1L2	-1,352805224	6,035342408	1,2089E-15	1,20872E-14
LYSMD1	1,293373936	4,270456022	1,30628E-15	1,30417E-14
CDK17	-0,806691913	5,427557295	1,3468E-15	1,34266E-14
VPS13D	-1,15833436	6,31955505	1,38145E-15	1,3752E-14
MPP5	-0,939569155	5,474644115	1,4135E-15	1,40505E-14
MRAS	-1,553185483	4,779535733	1,60583E-15	1,59391E-14
TRANK1	-1,422993148	5,106711155	1,61424E-15	1,59993E-14
REEP5	-0,887231407	7,413058144	1,62167E-15	1,60496E-14
POLA2	1,297605327	5,402993894	1,68399E-15	1,66423E-14
FAM60A	1,75814598	7,798587507	1,7319E-15	1,70909E-14
FMNL1	-1,465968784	5,563266435	1,84588E-15	1,81894E-14
ITGAX	-1,629397275	5,599218704	1,85576E-15	1,82604E-14
MFN1	1,551582512	6,706523504	1,93658E-15	1,90282E-14
USP39	1,0413558	7,063375947	1,96245E-15	1,92546E-14

CYC1	1,727027866	7,98504063	1,98494E-15	1,94473E-14
GART	1,034438454	6,951464853	2,029E-15	1,98504E-14
H2AFZ	1,464057712	8,49088138	2,04136E-15	1,99253E-14
PIK3C2B	-1,138401829	5,374485245	2,0425E-15	1,99253E-14
DSN1	1,335634323	5,317157174	2,21681E-15	2,15949E-14
APBB1IP	-1,663397411	4,188636354	2,25714E-15	2,19564E-14
SLC35A4	-0,734712168	6,681586666	2,29491E-15	2,2292E-14
LSM4	1,464648482	7,342476482	2,3468E-15	2,27636E-14
PRIM1	1,526212172	4,438981681	2,38353E-15	2,30871E-14
MBNL2	-1,14316627	6,194628732	2,40237E-15	2,32365E-14
FBXO5	1,555334774	4,342802926	2,43161E-15	2,34861E-14
NUP85	1,082331059	5,979179509	2,47832E-15	2,39034E-14
PTPN6	-0,901596173	6,543473203	2,55494E-15	2,46075E-14
PAK1IP1	1,160311992	5,415121157	2,5778E-15	2,47927E-14
C6orf89	-0,717126193	7,063120241	2,6E-15	2,4971E-14
RGS2	-1,756143316	6,409307898	2,69044E-15	2,58032E-14
VAT1	-1,188011833	7,892502266	2,757E-15	2,64044E-14
EMP3	-1,531608952	5,675842848	2,79023E-15	2,66852E-14
FAM162A	2,111395445	7,244231841	2,85691E-15	2,72847E-14
EPB41L2	-1,398473357	6,330747744	2,86706E-15	2,73433E-14
CYGB	-1,296022266	4,490102865	3,02171E-15	2,8778E-14
FANCG	1,930356758	5,310007438	3,19589E-15	3,03944E-14
MEF2A	-0,875741014	6,358472833	3,22784E-15	3,06555E-14
SPATA6	-1,490135388	2,092476404	3,78896E-15	3,59346E-14
THOP1	1,178476209	5,813555437	3,86671E-15	3,66209E-14
YARS2	1,140152214	5,022826783	4,22264E-15	3,99364E-14
PSMD2	1,612600631	9,614657855	4,47997E-15	4,23115E-14
PACSIN2	-0,919136268	6,265688046	5,20457E-15	4,90871E-14
RASSF8	-1,624980634	4,984095253	5,21191E-15	4,90884E-14
SMC4	1,576651265	7,608682789	5,29368E-15	4,97898E-14
IFI30	-1,4865959	7,665258834	5,46941E-15	5,13718E-14
CDK4	1,668483332	8,328441891	5,55107E-15	5,20671E-14
FCGR3A	-1,782004524	7,405085762	5,55898E-15	5,20697E-14
TTF2	1,38997574	5,317997504	5,5872E-15	5,22622E-14
SFXN3	-1,245136321	5,650018685	5,75311E-15	5,37403E-14
TXNDC11	-0,981944614	6,595524643	5,77955E-15	5,39135E-14
ZYG11B	-0,679554655	5,628443455	5,83029E-15	5,43125E-14
MTFR1	1,526679048	5,851469213	5,89158E-15	5,48086E-14
ARHGEF12	-1,011555606	7,73979366	5,91064E-15	5,49109E-14
SPRED1	-1,02479255	6,369876125	6,04672E-15	5,60987E-14
WDR43	1,016127532	6,725410409	6,4179E-15	5,94615E-14
GPI	1,789309655	9,812702508	6,76112E-15	6,25564E-14
PBXIP1	-1,058937649	7,199910467	6,97182E-15	6,44185E-14
HDAC7	-0,969859511	6,475122634	7,07243E-15	6,52597E-14
NAA50	1,228540592	8,387831491	7,27865E-15	6,70718E-14
SRPRB	1,34998912	7,447296403	7,49238E-15	6,89481E-14
TYROBP	-1,706497835	6,578583882	7,74455E-15	7,11726E-14
ZC3H7A	-0,681434856	5,704474689	7,88208E-15	7,22881E-14
STIP1	0,990119412	8,236800111	7,88713E-15	7,22881E-14
LSM12	0,914409341	6,86690901	7,93053E-15	7,25883E-14

PMAIP1	2,470673816	5,581253282	8,06821E-15	7,37495E-14
ARHGAP17	-0,78440112	5,878451765	8,56915E-15	7,82236E-14
ELMO1	-1,489726772	5,238297981	9,12345E-15	8,31722E-14
C11orf83	1,687272703	4,941305585	9,49605E-15	8,64534E-14
DUSP8	-1,635774866	3,808582874	9,53715E-15	8,67118E-14
TMEM133	-1,317749365	2,249357103	9,60766E-15	8,72366E-14
MPHOSPH9	1,227300527	4,324522993	1,00177E-14	9,08387E-14
SMARCB1	1,035095758	7,234671282	1,10676E-14	1,00226E-13
NUP43	0,966576619	5,873060012	1,11794E-14	1,01104E-13
CPOX	1,496854201	5,603945146	1,13869E-14	1,02844E-13
SLC17A5	-0,930127745	5,116489303	1,16419E-14	1,05008E-13
SH3BP1	1,47573457	6,594145002	1,16872E-14	1,05278E-13
C1QB	-1,830035342	8,365882493	1,17664E-14	1,05851E-13
CCT4	1,650794798	8,9222646	1,19334E-14	1,07212E-13
MRPL51	1,216078154	7,212256583	1,22575E-14	1,09979E-13
RSU1	-0,909783721	6,451868225	1,28817E-14	1,15427E-13
WDR19	-1,017510557	4,16460689	1,39473E-14	1,24811E-13
NDUFS6	1,498934798	6,637126398	1,43719E-14	1,2829E-13
NOP56	1,301001758	7,713622658	1,43736E-14	1,2829E-13
GLRX3	1,599399766	7,077812181	1,4774E-14	1,31614E-13
GPC1	2,840961628	8,860144291	1,47846E-14	1,31614E-13
CKS2	2,075295062	6,061395997	1,59477E-14	1,41783E-13
CEP72	1,806685742	3,470969855	1,67086E-14	1,48354E-13
TMEM14A	1,95805347	6,210860039	1,75369E-14	1,55506E-13
TMEM189	1,316505123	7,205406694	1,76487E-14	1,56294E-13
INPP5A	-0,902520638	5,185615968	2,05147E-14	1,81439E-13
IQGAP2	-1,696087569	4,508655182	2,07894E-14	1,8363E-13
TYSND1	1,468904171	5,694387798	2,12773E-14	1,87697E-13
AXL	-1,551549615	6,340709162	2,17514E-14	1,91632E-13
OBFC1	-1,099599585	5,284365852	2,47024E-14	2,17349E-13
SESTD1	-1,191343098	5,781897078	2,47454E-14	2,17447E-13
PLOD2	2,039972694	7,613032925	2,53746E-14	2,22689E-13
HSDL2	-1,014603816	5,938288648	2,67514E-14	2,3447E-13
ANPEP	-1,819494239	5,268058904	2,68301E-14	2,34557E-13
FZD5	-1,530045548	5,098339285	2,68302E-14	2,34557E-13
DDR1	1,507391665	9,178223218	2,76237E-14	2,41186E-13
AIMP2	1,170446205	5,401882263	2,93149E-14	2,55624E-13
NOP2	1,402098271	6,454513198	2,93949E-14	2,55995E-13
TCF3	1,344594007	7,000709292	2,95759E-14	2,57243E-13
NIPAL3	-0,92126663	5,808693216	3,07901E-14	2,67462E-13
POLR2D	0,996990142	5,91027601	3,08429E-14	2,6758E-13
ANXA5	-1,007228552	8,678336053	3,20454E-14	2,77659E-13
MRTO4	1,184681328	6,471014756	3,28768E-14	2,84501E-13
SCP2	-0,725860407	7,085588801	3,32506E-14	2,87371E-13
RANGAP1	1,18300686	7,769370914	3,38648E-14	2,92309E-13
TMEM63B	-0,968082396	6,799011572	3,4119E-14	2,94131E-13
HLA-DRB1	-1,814448107	9,738627125	3,56653E-14	3,07073E-13
GPSM3	-1,398077781	5,263832702	3,75633E-14	3,23006E-13
PUS1	1,287307123	4,994204195	3,83504E-14	3,29359E-13
FCER1G	-1,594804858	6,195697688	3,8492E-14	3,3016E-13

PCM1	-1,028372561	6,788536171	3,94698E-14	3,38121E-13
MAZ	1,081662391	8,087344397	3,96072E-14	3,38873E-13
FBXO32	2,51143281	5,526101235	3,98887E-14	3,40854E-13
NOB1	1,006728465	6,49752406	4,26141E-14	3,63686E-13
STAMBP	0,940613841	6,098871806	4,35327E-14	3,71062E-13
CASP3	1,022061514	6,030782571	4,41725E-14	3,76046E-13
EHD4	-0,960246779	6,497085981	4,76372E-14	4,05035E-13
NIT2	1,594055311	6,354736805	4,78404E-14	4,06256E-13
KLHL2	-0,954462957	4,973414384	4,90273E-14	4,15817E-13
CPSF6	0,677924691	6,791082866	4,9716E-14	4,21135E-13
BIN2	-1,600528943	4,029240221	5,03678E-14	4,26127E-13
MRPL9	0,989469515	6,285578165	5,16243E-14	4,36216E-13
CD151	-1,129342601	8,104139031	5,69384E-14	4,80524E-13
FSCN1	2,404470215	8,965207504	5,71562E-14	4,81765E-13
GPN1	1,120206404	6,739111486	5,7572E-14	4,84671E-13
LAPTM5	-1,561789196	8,707533299	5,8776E-14	4,94197E-13
TPCN1	-1,159936045	5,704021174	5,91249E-14	4,96518E-13
SETX	-0,895008464	6,692854538	6,01321E-14	5,04355E-13
PSMD11	1,358107013	7,391977338	6,23848E-14	5,22607E-13
C2orf29	0,87461969	6,523365026	6,2938E-14	5,26594E-13
HPS5	-0,881009191	5,684236456	6,34566E-14	5,30282E-13
CBX3	1,108889588	8,144623369	6,4809E-14	5,40921E-13
CACYBP	1,356702116	7,375770011	6,67866E-14	5,56746E-13
LIG1	1,355713464	5,819989776	6,70166E-14	5,5798E-13
MSH6	1,35197758	7,291741352	6,71393E-14	5,5832E-13
RPP21	1,422550309	4,59515952	6,80721E-14	5,65388E-13
KLF10	-1,178363479	6,946428997	6,86769E-14	5,69717E-13
LSG1	1,504950899	7,183041092	6,95018E-14	5,7586E-13
TUBB	1,08983169	10,75928466	7,23381E-14	5,98633E-13
PTS	1,66523653	5,072215633	8,1006E-14	6,69551E-13
IFNAR1	-0,72124279	6,677062273	8,42189E-14	6,95265E-13
SNRPA	0,91030618	6,415353535	8,89539E-14	7,33466E-13
RUSC1	1,071032455	6,163785564	8,93702E-14	7,36009E-13
EGR2	-1,701594328	4,916441306	9,1022E-14	7,48708E-13
SLC25A20	-1,127518081	4,123328391	9,15707E-14	7,52314E-13
MSI2	1,722612112	5,302730849	9,46519E-14	7,76692E-13
METTL1	1,297306143	5,243402356	9,48517E-14	7,77396E-13
CECR5	1,372408719	6,254624584	9,64572E-14	7,89606E-13
SLC25A17	0,954289788	5,548235585	9,68441E-14	7,91822E-13
SWAP70	-0,990425129	6,738099373	9,78936E-14	7,99445E-13
XPO1	1,08177427	8,445984935	9,89942E-14	8,07466E-13
PTGER4	-1,554660704	4,742057948	1,01762E-13	8,29051E-13
HDAC1	1,076278437	7,947245968	1,02473E-13	8,33849E-13
LOC152217	1,668851986	5,304920304	1,05312E-13	8,55925E-13
MAPK6	1,469683904	7,442792267	1,08254E-13	8,78787E-13
SF3B14	1,063095343	6,669966401	1,09379E-13	8,86046E-13
XPO5	1,154111314	6,801550912	1,09408E-13	8,86046E-13
KLF13	-1,292657215	7,248392116	1,12643E-13	9,11162E-13
MECP2	-0,751867182	6,066627886	1,15097E-13	9,29913E-13
RRM1	1,363991362	7,248170223	1,16444E-13	9,3968E-13

NCBP2	1,193662925	7,630517027	1,18846E-13	9,57933E-13
MPHOSPH8	-0,828387098	5,53756298	1,21606E-13	9,79021E-13
ARRDC3	-1,309748288	7,482988975	1,22836E-13	9,87755E-13
R3HDM1	1,031826199	5,937077434	1,23531E-13	9,92177E-13
FIBIN	-2,019232315	4,158118181	1,31874E-13	1,05794E-12
TSSC1	1,038582507	5,695787098	1,32901E-13	1,06492E-12
DDX49	1,12836802	6,340299729	1,34419E-13	1,07582E-12
DDX56	0,894319977	6,804594198	1,37521E-13	1,09937E-12
MAGOHB	1,415395908	4,782539672	1,45593E-13	1,16252E-12
HNRNPL	0,645568051	8,940493314	1,4662E-13	1,16936E-12
INTS7	1,061734062	5,807340772	1,5439E-13	1,22989E-12
NFKBIZ	-1,843974279	5,936277531	1,65105E-13	1,31371E-12
MFSD7	-1,490075228	2,880607532	1,66915E-13	1,32656E-12
MRPL42	1,099244601	6,181970152	1,73047E-13	1,3737E-12
PSMB4	1,083879087	8,54162022	1,74555E-13	1,38406E-12
ZMYND11	-0,814408467	6,591857925	1,81751E-13	1,43944E-12
UTP14C	-0,716824656	5,639210865	1,82106E-13	1,44058E-12
KCTD1	2,592421282	6,299313517	1,86216E-13	1,47139E-12
COIL	0,927078262	5,430589207	1,89198E-13	1,49322E-12
SGPL1	0,989694728	7,514473475	1,89593E-13	1,49407E-12
SAC3D1	1,362818371	4,008341445	1,89744E-13	1,49407E-12
EXOSC2	1,007865341	5,46870964	1,92291E-13	1,51238E-12
SFXN4	1,355609833	5,0716022	1,95803E-13	1,53823E-12
POLR3GL	-0,804535124	4,826926115	1,99046E-13	1,5619E-12
USP25	-0,975664559	5,807838745	2,00295E-13	1,5699E-12
PNRC2	-0,772975535	6,917701569	2,01567E-13	1,57806E-12
TBCEL	-0,896048929	4,499137457	2,04425E-13	1,5986E-12
TP53BP2	-0,774085853	6,245342983	2,07531E-13	1,62102E-12
SUCLG2	-0,778034002	5,990511698	2,08713E-13	1,62839E-12
CARM1	1,001510259	6,711408967	2,09627E-13	1,63366E-12
AZI2	-0,748561114	5,673161293	2,10874E-13	1,64149E-12
PARVG	-1,475737137	4,347879318	2,15185E-13	1,67314E-12
ETV1	-1,811506276	5,537512164	2,19751E-13	1,7067E-12
EFEMP1	-1,855306541	7,402655431	2,24875E-13	1,74451E-12
SPP1	4,295782927	10,65368841	2,41364E-13	1,8703E-12
EDEM1	-1,073547546	7,068262771	2,43743E-13	1,88659E-12
TOR1AIP1	-0,683370125	6,63736902	2,45139E-13	1,89524E-12
LSM7	1,36424685	5,794678293	2,52657E-13	1,95115E-12
MASTL	1,240143068	5,042686177	2,60286E-13	2,0078E-12
KIAA0754	-1,70428262	3,119946222	2,78994E-13	2,14967E-12
PRMT1	1,135246219	7,925897112	2,82063E-13	2,17087E-12
WDYHV1	1,214383875	4,496252062	2,91546E-13	2,24132E-12
PDE3B	-1,230837884	4,609070675	2,94255E-13	2,2596E-12
HMGB3	2,484532603	7,545114185	2,98958E-13	2,29313E-12
IL18R1	-1,644393731	2,95360338	3,09406E-13	2,3706E-12
PSMD12	0,918754353	6,405716245	3,1241E-13	2,38975E-12
ARL6IP5	-1,135865305	7,439117061	3,12605E-13	2,38975E-12
FAM21A	-0,859039178	5,337079453	3,19327E-13	2,4384E-12
SNRPD2	1,414996127	8,152485376	3,209E-13	2,44767E-12
RHOU	-1,586578055	5,052745461	3,26094E-13	2,4845E-12

CDK2	0,988234264	6,109173931	3,34132E-13	2,54291E-12
RDX	-0,918588291	7,208305051	3,38879E-13	2,57616E-12
CCT6A	1,472812071	8,786102973	3,49896E-13	2,65695E-12
RC3H1	-0,626228818	5,495776562	3,60128E-13	2,73161E-12
CSNK2A1	0,991323078	7,314107438	3,67471E-13	2,7842E-12
GSS	1,070950876	6,645840429	3,75228E-13	2,83982E-12
GTF2IRD1	1,22135569	5,75873371	3,77343E-13	2,85027E-12
DARS2	1,279995687	5,871381896	3,77445E-13	2,85027E-12
NFIX	-1,518280711	6,689893225	3,91739E-13	2,95494E-12
HMGXB4	0,939974763	5,91684664	4,13187E-13	3,11328E-12
TPD52	1,389456198	7,678804019	4,40181E-13	3,31302E-12
DCTPP1	1,702900176	6,496684467	4,62685E-13	3,47702E-12
ILK	-0,863310069	7,018489605	4,62991E-13	3,47702E-12
LRRC42	1,023849364	5,886049512	4,69564E-13	3,52251E-12
NDUFB4	1,381476275	7,30181277	4,86669E-13	3,64425E-12
AMOTL2	-1,272643337	6,253440314	4,86861E-13	3,64425E-12
FBXW11	-0,730399904	6,003874507	4,97879E-13	3,72263E-12
NISCH	-0,910178504	6,187160999	5,03407E-13	3,75984E-12
EIF3B	1,023944919	8,506942492	5,13992E-13	3,8347E-12
FAM103A1	1,030625727	5,528736366	5,38349E-13	4,01202E-12
UNC119	1,260070818	5,710917513	5,40929E-13	4,02685E-12
EED	1,021028471	5,063686479	5,4562E-13	4,05734E-12
SUSD1	-1,082344653	4,426262183	5,87727E-13	4,3657E-12
PRCC	0,784325832	6,851178943	5,95675E-13	4,41992E-12
PHF6	0,908335121	5,83267954	6,1969E-13	4,59311E-12
ATF7	-0,655375467	6,201064263	6,25124E-13	4,62836E-12
DYRK3	-1,314376747	3,525661027	6,53961E-13	4,83662E-12
SNAP47	0,724120765	5,741853583	6,91093E-13	5,1057E-12
DPYD	-1,553986507	5,853938144	7,12022E-13	5,25463E-12
SNRPC	1,086057659	6,967793458	7,20942E-13	5,31471E-12
NAE1	1,02225626	6,244493905	7,25329E-13	5,34127E-12
KIAA1143	-0,567668959	5,668800169	7,2795E-13	5,35479E-12
TARBP1	1,352442377	5,975648097	7,76168E-13	5,70333E-12
BDP1	-0,851442935	4,983178048	7,88982E-13	5,79124E-12
PTPRJ	-1,15196536	5,663051287	8,01426E-13	5,87626E-12
PSMG1	1,202365769	5,405342263	8,19442E-13	6,0019E-12
RHBDD3	1,216733004	4,863988538	8,27153E-13	6,05188E-12
ZNF131	1,059465326	5,887306817	8,38935E-13	6,13151E-12
ACOT2	-1,141189017	4,62252542	8,7285E-13	6,37255E-12
ZNF25	-0,959813032	3,875948415	8,7781E-13	6,40191E-12
DHX34	1,196440835	5,243247845	8,82914E-13	6,43226E-12
SNRPG	1,650519425	7,122325338	8,93207E-13	6,5003E-12
PSMD7	0,857278441	7,431776579	9,09794E-13	6,61395E-12
PAWR	1,071339734	5,103397894	9,19521E-13	6,67754E-12
ROCK1	-0,781044119	6,529005121	9,4693E-13	6,86927E-12
RAPH1	-1,146788283	6,120464256	9,52136E-13	6,8997E-12
UBA7	-1,33841783	5,508068444	9,7511E-13	7,05868E-12
UQCRHL	1,412851951	3,994477254	9,77309E-13	7,0671E-12
KLF11	-0,91175779	5,422794838	9,79747E-13	7,07236E-12
TADA2B	-0,711967238	5,227372111	9,80111E-13	7,07236E-12

RNASEH1	0,941472759	4,884120865	9,88172E-13	7,12299E-12
DVL3	1,75780366	8,377422274	9,95588E-13	7,16886E-12
EXOC6	-0,920413005	4,815515405	1,0386E-12	7,47071E-12
SLC25A13	1,018413597	5,67933699	1,07026E-12	7,69028E-12
CRABP2	4,348578278	7,145978763	1,08868E-12	7,81445E-12
MDK	2,681571224	8,317814707	1,10569E-12	7,9282E-12
CSTF2	1,325360719	5,231237173	1,12303E-12	8,04405E-12
TRIM22	-1,337305697	6,527165118	1,15338E-12	8,25275E-12
PDCD10	1,562293424	6,999332635	1,17216E-12	8,37836E-12
LMNA	-1,004697971	9,132366682	1,20392E-12	8,59635E-12
UBASH3B	-1,54848946	4,733410797	1,21024E-12	8,63246E-12
MPP6	1,821349455	3,480015877	1,26285E-12	8,99827E-12
DOLPP1	1,039007365	5,105110792	1,27571E-12	9,08047E-12
ITPK1	-0,883905951	6,313561516	1,28466E-12	9,1346E-12
RBL2	-0,724075772	6,885964273	1,28783E-12	9,14761E-12
PPP2CB	-0,880952788	6,943364833	1,29034E-12	9,15592E-12
PIGX	1,571218673	6,462496054	1,29779E-12	9,19919E-12
RNF123	-0,716419732	5,219182897	1,30442E-12	9,23662E-12
USP5	0,945489951	7,602552705	1,36742E-12	9,67267E-12
OPA1	1,146921818	7,406779032	1,38832E-12	9,81035E-12
UCHL5	0,918698942	5,531945134	1,39986E-12	9,88164E-12
CELSR2	2,26580907	6,662895861	1,42199E-12	1,00274E-11
MED10	1,293759204	5,866114348	1,42713E-12	1,00533E-11
NUP107	1,092363246	6,223344325	1,46725E-12	1,03252E-11
EIF5A	0,997372178	9,477607539	1,47243E-12	1,03511E-11
DPH2	1,105893025	5,527764306	1,51589E-12	1,06456E-11
FNBP1	-1,062696049	6,116577523	1,54934E-12	1,08693E-11
CSTF3	0,78020469	5,511040371	1,60226E-12	1,12278E-11
KIAA0907	1,379112863	6,005763099	1,60373E-12	1,12278E-11
DRG1	1,161438664	6,932087182	1,62008E-12	1,13306E-11
NLN	1,263333747	5,072120254	1,62658E-12	1,13644E-11
TBL1XR1	1,633458824	8,720076465	1,63295E-12	1,13972E-11
IK	-0,638947552	6,892820851	1,6744E-12	1,16745E-11
FNDC3A	-0,865216714	6,325696112	1,68771E-12	1,17554E-11
VAMP3	-0,724045484	7,281521679	1,71841E-12	1,1957E-11
AFF4	-0,825898469	6,851647625	1,74189E-12	1,2108E-11
EGLN3	3,538050809	7,386484898	1,74787E-12	1,21372E-11
NEK7	-0,999172342	6,646530437	1,7698E-12	1,2277E-11
ARNT	-0,651964992	6,447134186	1,81336E-12	1,25664E-11
SPAG9	-0,702154315	7,101188079	1,86136E-12	1,28859E-11
CKAP4	1,286979453	8,260770953	1,884E-12	1,30294E-11
UBTD1	-1,096477186	4,762162522	1,92388E-12	1,32917E-11
CAPN2	-1,030259682	8,628306837	1,98175E-12	1,36777E-11
FBXW4	-0,865225663	6,036105624	2,08653E-12	1,43863E-11
KCMF1	0,866791256	7,1855473	2,10408E-12	1,44927E-11
TBCCD1	1,283125162	5,584985913	2,12438E-12	1,46177E-11
MKL2	-0,960033224	6,055053057	2,15231E-12	1,4795E-11
STX7	-0,857683058	6,082830037	2,1638E-12	1,4859E-11
HIPK3	-1,0377211	6,156601627	2,1671E-12	1,48666E-11
MRPS24	1,199558386	6,550772734	2,18108E-12	1,49475E-11

SLC19A2	-1,141003304	5,048566612	2,19463E-12	1,50253E-11
MLLT6	-0,94308543	7,267018436	2,42052E-12	1,65552E-11
FCHSD2	-0,822549724	5,847631258	2,48492E-12	1,69786E-11
DDX5	-0,615605267	9,414017539	2,48931E-12	1,69916E-11
POLE3	1,170612103	7,350664438	2,50349E-12	1,70713E-11
DYNC1LI2	-0,751542288	7,140856692	2,63092E-12	1,79089E-11
MRPS26	1,300361978	6,13164994	2,63157E-12	1,79089E-11
GAB1	-1,235013373	5,223807438	2,65973E-12	1,80825E-11
XPNPEP3	1,043661817	4,634156652	2,67739E-12	1,81844E-11
ACAT1	-0,922426001	5,828371741	2,69971E-12	1,83177E-11
SPAST	1,200574901	6,320321835	2,70437E-12	1,83311E-11
SNRPF	1,109379689	6,168645222	2,73663E-12	1,85314E-11
SEC61A2	1,37731325	3,29532556	2,76182E-12	1,86834E-11
CDH24	1,75046951	4,395955093	2,80991E-12	1,89899E-11
NFAT5	-0,959142383	6,087843393	2,84153E-12	1,91846E-11
SUPV3L1	0,800663233	5,357031676	2,88481E-12	1,94575E-11
PA2G4	0,806228302	8,104851844	2,92401E-12	1,97024E-11
PCF11	-0,784188109	5,654961164	2,94192E-12	1,98035E-11
ETS2	-1,196775709	8,076933256	3,03689E-12	2,04227E-11
HSPB1	2,05888313	10,10678154	3,04389E-12	2,04495E-11
KIAA1377	-1,3172298	2,992155449	3,11131E-12	2,08819E-11
SERPINB6	-1,09492554	6,809035465	3,31194E-12	2,22066E-11
PARP4	-0,900803227	6,764747459	3,496E-12	2,34177E-11
WDR74	1,01862905	5,754944015	3,56123E-12	2,38312E-11
TCF7L2	-0,896971484	5,126033039	3,63244E-12	2,4277E-11
ATAD3A	1,217734125	5,661986763	3,63496E-12	2,4277E-11
SOX12	1,708478317	5,737418735	3,68069E-12	2,45583E-11
UBA2	1,155437654	8,125700553	3,70102E-12	2,46699E-11
UFD1L	1,151648901	7,005697562	3,7145E-12	2,47356E-11
UQCRH	1,302786699	7,587629093	3,7516E-12	2,49582E-11
NARF	1,212803318	6,445982438	3,90265E-12	2,59378E-11
DAP3	0,932310267	7,419239078	3,91772E-12	2,60126E-11
ZFYVE16	-0,706703709	5,548302072	4,06163E-12	2,69419E-11
NFIA	-1,142700697	5,448484771	4,28371E-12	2,83874E-11
PDE4DIP	-0,996986355	6,660366217	4,40796E-12	2,91824E-11
HLA-DRA	-1,698176575	10,65978383	4,62729E-12	3,06047E-11
ANTXR2	-1,325783473	5,714827919	4,69188E-12	3,10018E-11
ZFAND5	-0,821658163	8,156580251	4,87838E-12	3,2203E-11
TOB2	-0,749799587	6,634971084	5,0373E-12	3,32199E-11
MSTO1	1,00728517	5,657641616	5,19982E-12	3,42585E-11
AIF1	-1,45108482	5,239389799	5,29294E-12	3,48384E-11
SNHG4	1,755563379	1,349965951	5,32002E-12	3,49829E-11
PDZD11	1,044439457	5,987706309	5,35928E-12	3,52071E-11
INO80	-0,758667281	5,580452917	5,42546E-12	3,56075E-11
PHGDH	2,357677547	7,223492479	5,43727E-12	3,56507E-11
CEP76	1,25987588	3,970819177	5,65595E-12	3,70489E-11
GPR89A	0,775183811	4,844260296	5,68425E-12	3,71986E-11
HEATR1	0,93384892	6,542865166	5,76139E-12	3,76672E-11
EVL	-0,913866363	6,330299876	5,83235E-12	3,80946E-11
TOMM22	1,295977044	7,451849521	6,23578E-12	4,06907E-11

KIAA0319L	-0,891148579	6,69968101	6,33232E-12	4,12811E-11
TDRD3	-0,868689436	4,248183246	6,56515E-12	4,2735E-11
C8orf4	-1,950234705	7,170413773	6,56787E-12	4,2735E-11
FAM8A1	-0,862748782	5,833068322	6,61517E-12	4,30017E-11
FBL	1,584812225	8,223946922	6,67331E-12	4,33384E-11
TSPAN13	-1,321786936	7,567169713	6,70668E-12	4,35136E-11
B4GALT4	1,966783276	6,690098529	6,75834E-12	4,38071E-11
FYN	-1,214789061	5,703988647	6,79447E-12	4,39995E-11
ABHD5	-0,837837941	5,494646827	6,92462E-12	4,47998E-11
WBP2	-0,701772238	7,335164931	6,94192E-12	4,48691E-11
DARS	0,978672359	7,382120269	7,12258E-12	4,59933E-11
ZC3HAV1L	1,649156793	3,135157028	7,55851E-12	4,8762E-11
MLF2	0,99029622	8,44892123	7,60814E-12	4,90359E-11
SNRNP25	1,357349534	5,682499857	7,84642E-12	5,05238E-11
ARL6IP6	1,036958437	5,489398728	7,88047E-12	5,06952E-11
LYZ	-1,805993443	9,517763173	8,50473E-12	5,46595E-11
DCUN1D1	1,892469951	6,967260503	8,57788E-12	5,50778E-11
PAFAH1B1	-0,652983427	7,547061082	8,62167E-12	5,53068E-11
DNAJA3	1,048636501	6,548785926	8,90783E-12	5,70888E-11
TMEM66	-0,871999474	8,247063214	9,11754E-12	5,83779E-11
SENP5	1,096408755	6,686155842	9,20227E-12	5,88652E-11
MRPL15	1,259998257	6,338906338	9,26053E-12	5,91823E-11
HSPA4L	2,097544083	4,766502506	9,2945E-12	5,93438E-11
PFKP	1,569506127	7,60765116	9,38345E-12	5,98557E-11
NDUFB5	1,676857136	7,629781818	9,48073E-12	6,04197E-11
CMAS	1,139535497	6,17547254	9,84851E-12	6,2705E-11
TAF2	0,792553055	5,944620708	9,94682E-12	6,32718E-11
NUCB1	-0,806076238	8,744695946	9,96534E-12	6,33305E-11
IDS	-0,832333941	7,843544281	1,00849E-11	6,40304E-11
PPM1G	0,815827956	7,875653362	1,01023E-11	6,40815E-11
TOMM70A	0,910448787	7,241580076	1,03962E-11	6,58843E-11
CDKN1A	-1,23691192	7,913372614	1,05866E-11	6,70291E-11
CTHRC1	2,509259878	6,674919507	1,09178E-11	6,90616E-11
NEDD4L	-1,12622781	7,008060581	1,10539E-11	6,9858E-11
L2HGDH	1,000502775	5,200036366	1,11625E-11	7,04787E-11
DPY30	1,170583849	5,809262884	1,1241E-11	7,09086E-11
FAM91A1	0,770929581	6,937052568	1,13834E-11	7,17406E-11
DLG5	1,471036308	7,050604342	1,14529E-11	7,21118E-11
EIF6	1,063122664	7,884695883	1,17876E-11	7,41512E-11
OCIAD2	1,614091525	6,501273493	1,1902E-11	7,48018E-11
PTGES3	0,85913661	9,250576325	1,19695E-11	7,51563E-11
YEATS4	1,548456442	5,379712966	1,2096E-11	7,58807E-11
WASF1	2,504515076	5,251086859	1,21868E-11	7,63803E-11
RCN2	1,374683938	7,416077228	1,23228E-11	7,7162E-11
ADRM1	0,844692587	7,377909018	1,24679E-11	7,79989E-11
CRIPT	1,398484965	5,425582495	1,25312E-11	7,83229E-11
CHD1	-0,788075252	5,38631823	1,29555E-11	8,09005E-11
CTDSP1	-0,712510671	7,180314722	1,29981E-11	8,10927E-11
TSFM	0,971928727	5,546160734	1,30775E-11	8,15132E-11
UBE2V2	0,963011766	6,15789055	1,41852E-11	8,8337E-11

RUFY1	-0,718519147	5,61197401	1,42203E-11	8,84747E-11
RIPK1	-0,661705787	5,806120397	1,44361E-11	8,97353E-11
PDIA4	1,32670713	9,20964564	1,44612E-11	8,98094E-11
GALNT10	-1,219927369	5,991456955	1,46936E-11	9,117E-11
CDK2AP1	0,91457422	7,749058912	1,47648E-11	9,15281E-11
PLXDC2	-1,239934411	5,446795708	1,47782E-11	9,15281E-11
MTCH2	0,89564863	7,180459844	1,48426E-11	9,18435E-11
TRMT2A	1,156213858	5,6516202	1,5357E-11	9,49033E-11
TBC1D9	-1,089852066	6,028244674	1,53649E-11	9,49033E-11
SLC33A1	0,963509074	6,195440653	1,57033E-11	9,69057E-11
GTF2H4	0,831633554	5,156122152	1,57432E-11	9,70638E-11
RPAP3	0,768694459	5,526587636	1,60852E-11	9,90829E-11
ZC3H13	-0,868922377	6,165538262	1,63517E-11	1,00634E-10
PDIA6	1,02218841	9,384794682	1,68838E-11	1,03815E-10
LAGE3	1,380765994	4,983210471	1,71065E-11	1,05089E-10
C1orf109	1,012490895	4,749093207	1,71443E-11	1,05227E-10
PAR-SN	-1,255830342	2,961446295	1,74781E-11	1,07179E-10
LCLAT1	0,973655093	5,954064528	1,76047E-11	1,07858E-10
SAAL1	1,055069581	4,120625779	1,76461E-11	1,08015E-10
DUSP11	0,994307278	5,820217837	1,77719E-11	1,08688E-10
HEATR2	0,950534176	5,919444764	1,80387E-11	1,1022E-10
PXK	-0,829710942	3,788794212	1,80779E-11	1,10361E-10
BANF1	1,023537144	7,911835607	1,81274E-11	1,10478E-10
TAF1A	0,949053102	3,520592326	1,81294E-11	1,10478E-10
LRPAP1	-0,793872553	7,251796268	1,85319E-11	1,1283E-10
PGAM1	1,146033258	9,359038143	1,90127E-11	1,15653E-10
N4BP2L1	-1,327231003	3,861168663	1,93895E-11	1,17841E-10
CIRH1A	0,904359078	6,460979274	2,00393E-11	1,21681E-10
C4orf46	1,149190588	4,222208016	2,02127E-11	1,22625E-10
BAIAP2L1	1,074271912	6,762031533	2,05276E-11	1,24425E-10
C1QBP	1,211564656	7,260221528	2,07537E-11	1,25683E-10
SETD2	-0,714389807	6,249098593	2,09278E-11	1,26625E-10
DGKA	1,70261343	6,433550251	2,1011E-11	1,27016E-10
KLF4	-1,563717282	6,625649211	2,11166E-11	1,27541E-10
WDFY3	-0,890835799	5,900933271	2,1294E-11	1,28499E-10
PES1	1,049964403	7,332457299	2,1768E-11	1,31243E-10
VPS72	1,080909339	6,260485321	2,18709E-11	1,31747E-10
FRMD6	1,942972565	7,113340717	2,19085E-11	1,31857E-10
NAA25	0,840701205	5,479161829	2,20009E-11	1,32296E-10
CLNS1A	1,260649361	7,150831033	2,25426E-11	1,35434E-10
RINT1	0,883567032	5,345318861	2,25919E-11	1,35611E-10
SSBP1	0,971107468	6,683045117	2,26312E-11	1,35728E-10
SRRT	0,730560778	6,971008333	2,29569E-11	1,3756E-10
HIST1H2BD	2,211386084	4,343569518	2,35393E-11	1,40926E-10
IFIT5	-1,012699174	5,239177791	2,36206E-11	1,41288E-10
PTGES2	0,968903891	5,926798857	2,37884E-11	1,42168E-10
POLR2J	1,118639658	6,066557326	2,40143E-11	1,43392E-10
NUPL2	1,00729511	5,218528522	2,46698E-11	1,47177E-10
RPF2	0,944485407	5,044943442	2,55532E-11	1,52314E-10
MAML2	-1,251493327	4,555767281	2,60877E-11	1,55364E-10

PITPNA	-0,650593806	6,982761745	2,62928E-11	1,56449E-10
ATP2B4	-1,109500471	7,964558467	2,68563E-11	1,59597E-10
FAM63B	-0,868864136	3,969580509	2,68686E-11	1,59597E-10
FXR1	1,467556358	8,162764115	2,69143E-11	1,59603E-10
ABTB1	-1,002140014	4,854231251	2,69165E-11	1,59603E-10
CENPQ	1,466341303	4,125022903	2,71293E-11	1,60725E-10
RPAP2	0,982443574	4,413587718	2,73507E-11	1,61896E-10
FASTKD3	1,248385977	4,1553995	2,80354E-11	1,65805E-10
TCP1	1,001414033	8,413561168	2,8264E-11	1,67012E-10
PARP1	0,933136882	8,319742528	2,98106E-11	1,75999E-10
MSH2	1,188179865	6,17673538	3,08146E-11	1,81769E-10
FBXL19	1,292891543	4,969238465	3,24923E-11	1,915E-10
ATP6V1B2	-0,827056113	7,371231361	3,25268E-11	1,91537E-10
LOC220729	1,406211292	4,332084669	3,27081E-11	1,92439E-10
EPC1	-0,692408271	4,533494251	3,34699E-11	1,96751E-10
RNF187	0,847553605	7,880556336	3,36651E-11	1,97728E-10
FBXL3	-0,727146964	6,094241654	3,38772E-11	1,98803E-10
PAIP1	1,068137368	7,72854915	3,40715E-11	1,99771E-10
IGFBP2	3,342391451	9,653774326	3,41165E-11	1,99863E-10
SDC4	-1,410018213	8,849751854	3,44086E-11	2,01401E-10
NSUN2	1,077989252	7,202064686	3,46681E-11	2,02698E-10
CRIP1	-1,883489866	6,379768537	3,46897E-11	2,02698E-10
PARP2	0,962929342	5,02801012	3,53656E-11	2,06471E-10
IRAK1	0,980236304	7,865747964	3,60067E-11	2,10034E-10
TIMM10	1,108099413	5,27785352	3,79036E-11	2,2091E-10
RBM34	0,793061907	5,397631282	3,84379E-11	2,23833E-10
LILRB2	-1,415206152	4,14833051	3,9349E-11	2,28943E-10
HAUS5	1,153284681	5,169301373	3,96717E-11	2,30624E-10
HNMT	-1,333153506	5,286084259	4,04537E-11	2,34969E-10
DDX55	0,840147861	5,106586732	4,1715E-11	2,42089E-10
ZNF330	-0,661825048	5,053005301	4,2128E-11	2,44278E-10
KIAA0240	-0,819175542	4,999830513	4,2952E-11	2,48844E-10
PLEKHB2	-0,614742362	7,697545473	4,35794E-11	2,52265E-10
NCAPD3	1,159006674	5,916518503	4,48263E-11	2,59263E-10
METTL5	0,992771303	5,476732363	4,48854E-11	2,59384E-10
PSMC2	0,859902891	7,473100951	4,53245E-11	2,617E-10
MBNL1	-0,685218311	7,97068392	4,5842E-11	2,64351E-10
STEAP1	2,170850623	5,283941531	4,58611E-11	2,64351E-10
NIF3L1	1,054284551	5,488821217	4,65631E-11	2,6817E-10
GPR89C	0,799930674	3,158318219	4,87734E-11	2,80663E-10
EXOSC3	1,180625476	4,879021908	5,09824E-11	2,93127E-10
MAPK3	-0,801815325	6,308615245	5,14058E-11	2,95313E-10
MRPL4	1,223590569	6,425655442	5,14826E-11	2,95505E-10
EIF4A3	0,927367632	7,519091223	5,17015E-11	2,96512E-10
GTF3C3	0,843979017	5,997845064	5,2393E-11	3,00225E-10
SIRPA	-1,196771345	6,803053724	5,39887E-11	3,09109E-10
PION	-1,214369903	4,92787807	5,44401E-11	3,11432E-10
CRY1	-0,818660687	5,077613542	5,53373E-11	3,16299E-10
VTA1	0,82277677	6,464852239	5,58372E-11	3,18889E-10
EAF1	-0,582523907	5,700426099	5,70234E-11	3,25391E-10

AMZ2	1,085513865	6,925228071	5,74762E-11	3,27701E-10
PABPC3	1,218656236	2,492911737	5,89209E-11	3,35657E-10
METTL2A	0,865816093	5,404144415	5,90271E-11	3,35981E-10
GARS	1,052919856	7,780527017	5,91426E-11	3,36358E-10
HEXB	-0,79162508	7,384101518	5,98909E-11	3,4033E-10
RNPS1	0,744530721	7,812109494	6,0044E-11	3,40916E-10
LOC388796	1,451279565	5,306970647	6,12245E-11	3,47329E-10
HCST	-1,460125593	3,563872466	6,13226E-11	3,47597E-10
PNPT1	0,996756078	6,400700953	6,20673E-11	3,51518E-10
C5orf28	1,264155728	5,005384369	6,21175E-11	3,51518E-10
MGC72080	1,530762031	4,034829563	6,28873E-11	3,55579E-10
LITAF	-0,89426035	8,271955299	6,31529E-11	3,56785E-10
ANKRD40	-0,610448211	6,578282483	6,51709E-11	3,67881E-10
RUVBL2	0,958660156	7,20676614	6,59585E-11	3,72019E-10
PPP1CC	0,717273349	7,995978516	6,60713E-11	3,72347E-10
PUF60	0,89091249	7,985828016	6,71832E-11	3,78301E-10
ATF3	-1,620146205	6,814264768	6,75289E-11	3,79933E-10
ITGB2	-1,474232314	7,458973833	6,98847E-11	3,92864E-10
PARVA	-0,882183583	6,549452497	7,28276E-11	4,0907E-10
FBXO22	0,893430583	5,307343932	7,31494E-11	4,1054E-10
PYGO2	0,807280486	6,597346725	7,36758E-11	4,13154E-10
SKAP2	-0,834363654	6,228194665	7,41461E-11	4,15449E-10
MRPS10	0,924594056	6,469562395	7,47111E-11	4,18272E-10
MLL5	-0,761408661	6,615476511	7,50846E-11	4,20018E-10
DSC2	2,310070412	7,729810581	7,61108E-11	4,2541E-10
SLC1A4	1,996302369	7,290752688	7,84112E-11	4,37908E-10
TIPIN	1,138861121	3,414780069	7,86723E-11	4,38912E-10
MOCS3	0,835312844	4,433096756	7,87196E-11	4,38912E-10
SRPX	-1,849801579	4,332204556	7,9331E-11	4,41959E-10
SNRNP40	0,833228336	6,235666478	8,20446E-11	4,56704E-10
MRPS33	1,250670211	5,772908389	8,24457E-11	4,58562E-10
ATP6V0D1	-0,805735902	7,214718394	8,31389E-11	4,61831E-10
N4BP1	-0,770574979	6,654254596	8,31866E-11	4,61831E-10
LRPPRC	0,922957646	8,125405527	8,32366E-11	4,61831E-10
RABEP1	-0,691301332	6,041391754	8,33285E-11	4,61965E-10
KCTD2	-0,662347898	5,614278889	8,40374E-11	4,65517E-10
RAB5B	-0,572946627	7,930537044	8,57945E-11	4,74864E-10
TJP2	-0,9856212	6,714965381	8,95837E-11	4,95435E-10
FBXL5	-0,827019529	7,10590367	8,98246E-11	4,96365E-10
COASY	0,787089101	6,755448174	9,10327E-11	5,02633E-10
KATNAL1	-0,836104457	4,128952671	9,275E-11	5,11701E-10
PGK1	1,372323202	10,54415871	9,77975E-11	5,39112E-10
HTT	-1,00255931	6,76561037	9,83281E-11	5,41599E-10
PSMC4	1,216752964	7,488315786	1,00161E-10	5,5125E-10
ALG6	0,809160256	4,501796779	1,01964E-10	5,60722E-10
PABPC1	1,100367037	11,27158642	1,03248E-10	5,67324E-10
EIF2AK1	0,713091747	8,237231484	1,04797E-10	5,7537E-10
ADSL	1,148899191	6,904885794	1,05798E-10	5,80399E-10
ROCK2	-0,92279234	5,820255496	1,05913E-10	5,80562E-10
RAP1A	-0,759116213	6,472403215	1,06541E-10	5,83537E-10

TIMM44	1,096390544	5,637850792	1,07211E-10	5,86737E-10
HNRNPC	0,693016761	9,674806541	1,07894E-10	5,90001E-10
PLAU	2,557613847	8,267997235	1,08694E-10	5,93896E-10
VPS11	-0,656742394	5,901512953	1,10301E-10	6,02198E-10
EMP1	-1,447442705	8,53741523	1,12433E-10	6,13344E-10
VWA5A	-1,246136589	5,452354666	1,13256E-10	6,17338E-10
KIAA0513	-1,225325477	4,795276425	1,13415E-10	6,17714E-10
NPDC1	-1,273466111	4,992989664	1,13944E-10	6,20098E-10
RPLP0	1,166544171	11,86542381	1,14232E-10	6,21168E-10
CHORDC1	1,250909385	5,55750653	1,15157E-10	6,25701E-10
LZTFL1	-0,77758569	4,785943196	1,16508E-10	6,32539E-10
HMHA1	-1,10192839	5,503139109	1,16909E-10	6,34006E-10
ATP5G1	1,372480947	6,676575371	1,16964E-10	6,34006E-10
ERO1L	2,093854109	7,869995082	1,18176E-10	6,40066E-10
POLR1C	1,026714168	5,481565094	1,19292E-10	6,45599E-10
IDH2	1,319430002	7,779180318	1,20533E-10	6,51797E-10
TMEM183A	1,292699809	7,689841316	1,23678E-10	6,68273E-10
LDHA	1,278633512	10,56731631	1,26178E-10	6,8124E-10
TMED3	1,098984328	7,465928369	1,26588E-10	6,82912E-10
TRMT6	1,030137153	5,639351475	1,27535E-10	6,87481E-10
C4A	-1,80453539	7,921804241	1,27672E-10	6,87673E-10
APOA1BP	1,438352634	7,655008953	1,27933E-10	6,88536E-10
STRAP	0,953821114	8,023946898	1,29386E-10	6,95807E-10
C20orf194	-1,099706899	5,269204717	1,30802E-10	7,02868E-10
AAAS	0,653162541	5,709780763	1,31153E-10	7,04197E-10
PLVAP	-1,130238146	6,433382248	1,32069E-10	7,08561E-10
CLEC16A	-0,854159691	5,370982249	1,35626E-10	7,27072E-10
NMD3	1,090709794	7,089431049	1,36687E-10	7,32184E-10
PWP1	0,610550332	6,323767497	1,38893E-10	7,43415E-10
RALGPS2	1,279241905	5,120513752	1,4028E-10	7,5025E-10
NSUN5	1,045064881	5,326159933	1,41345E-10	7,55355E-10
CCDC142	0,700424733	5,418728493	1,42424E-10	7,60524E-10
GCSH	1,445002509	5,509255743	1,46825E-10	7,83409E-10
H2AFY2	1,732811564	5,578027763	1,48027E-10	7,89207E-10
GTF3C2	0,636546764	6,886999962	1,48275E-10	7,89912E-10
APEX1	0,907718171	7,799240782	1,48517E-10	7,90585E-10
OTUD4	-0,726665378	6,187528197	1,53059E-10	8,14126E-10
SH3GL1	0,875235153	7,196703995	1,63132E-10	8,66827E-10
MRPS35	1,078850679	6,804489676	1,63221E-10	8,66827E-10
TSC22D1	-1,100128663	8,198129503	1,68831E-10	8,95921E-10
NCOA7	-1,384338073	7,238127121	1,69219E-10	8,97282E-10
POLR2G	0,960359878	6,618774839	1,71465E-10	9,08486E-10
RAP2B	1,360271691	7,273174693	1,72258E-10	9,1198E-10
CD47	-1,07722945	7,793292014	1,72516E-10	9,12635E-10
PHF5A	1,296847444	6,186912635	1,87086E-10	9,88948E-10
TARS2	0,767092004	5,403752591	1,87264E-10	9,89117E-10
SUMO2	0,870409482	8,649822336	1,88092E-10	9,92521E-10
WDR77	0,983479639	6,163716926	1,88199E-10	9,92521E-10
ZNF7	0,76567059	5,179129667	1,88979E-10	9,95866E-10
CDYL	0,868767921	5,908110996	1,91015E-10	1,00582E-09

GNL3	0,767275264	6,791567582	1,91676E-10	1,00852E-09
SFN	2,496841874	9,539126931	1,93001E-10	1,01471E-09
LYST	-1,055174043	5,147798794	1,93178E-10	1,01485E-09
SLC27A1	-0,787763185	4,994424859	1,93655E-10	1,01658E-09
DPP3	0,852790952	6,670016043	1,95082E-10	1,02328E-09
GOT2	0,992327316	7,60088042	2,01697E-10	1,05717E-09
RBL1	1,020776036	4,312134808	2,02841E-10	1,06234E-09
FBXW2	-0,657744419	6,621174966	2,04969E-10	1,07266E-09
MRPS22	1,027977357	5,879947483	2,0619E-10	1,07823E-09
DNAJC2	0,936620314	5,771093461	2,06507E-10	1,07906E-09
OTUB1	0,703067435	7,340460296	2,07857E-10	1,08528E-09
SLU7	-0,560151781	5,897540303	2,12976E-10	1,11116E-09
VDAC2	1,01539285	8,418056218	2,22849E-10	1,16178E-09
TNFRSF10D	-1,642226111	3,808541555	2,25193E-10	1,1731E-09
DSCR3	-0,621912366	5,918224392	2,38729E-10	1,24267E-09
SAMD1	0,994933381	4,881897089	2,38936E-10	1,24279E-09
PPM1K	-1,156666078	3,919153952	2,45803E-10	1,27754E-09
PARL	1,548200107	7,270325691	2,50492E-10	1,30092E-09
NR2F2	-1,050327021	5,799367899	2,55456E-10	1,32569E-09
PREB	0,958267915	6,940353644	2,64111E-10	1,36956E-09
CAD	1,224050963	6,864700592	2,64349E-10	1,36976E-09
PDCD6	1,385839952	7,725772257	2,72423E-10	1,41052E-09
DNMT3A	1,1065068	5,594837984	2,75392E-10	1,42481E-09
MPHOSPH10	0,661268336	5,966434668	2,76126E-10	1,42752E-09
YWHAQ	0,962806312	9,566015584	2,78033E-10	1,43629E-09
CTSZ	-0,992249144	8,572325036	2,79535E-10	1,44296E-09
RNF7	1,205094011	7,260162195	2,85136E-10	1,47E-09
GPATCH2	0,791791498	4,679847861	2,85205E-10	1,47E-09
MRPL11	1,251695885	6,355117898	2,89048E-10	1,48869E-09
COX5A	1,083692595	7,249734535	2,90716E-10	1,49615E-09
CYTH1	-0,788772352	6,414483194	2,93503E-10	1,50935E-09
FMNL3	-1,044865226	5,912853386	2,95876E-10	1,5204E-09
EIF5A2	1,655036014	5,109883532	2,96244E-10	1,52115E-09
PMS2CL	0,828315331	3,671753553	2,99992E-10	1,53924E-09
AAGAB	0,788012681	6,508676226	3,02622E-10	1,55156E-09
TIMP2	-1,34277297	8,60374691	3,04203E-10	1,5585E-09
ENO1	1,114973521	11,57393255	3,04767E-10	1,56021E-09
TMEM106C	1,532678395	7,286880416	3,05803E-10	1,56434E-09
UBE2G2	-0,652687357	6,359417566	3,06077E-10	1,56457E-09
MEA1	0,836996027	6,600296513	3,0707E-10	1,56847E-09
DDX47	0,865879848	6,832117172	3,10158E-10	1,58306E-09
FARSA	0,907875251	6,634965408	3,20792E-10	1,63552E-09
GTF2H3	0,838215096	4,912428831	3,20916E-10	1,63552E-09
LHFPL2	-1,04966791	6,146131784	3,21783E-10	1,63871E-09
H1F0	1,324652655	8,726631267	3,2303E-10	1,64384E-09
LRRC8D	1,212012982	6,309596192	3,2537E-10	1,65451E-09
GLB1	-0,687618311	6,581944172	3,25681E-10	1,65486E-09
MAD2L2	1,32666447	5,82638218	3,30102E-10	1,67607E-09
YY1	0,726637409	6,874821193	3,34896E-10	1,69915E-09
SERPINE2	2,514002897	7,050794023	3,35204E-10	1,69945E-09

LSM2	1,331231611	6,339736588	3,43586E-10	1,74065E-09
SMC2	1,15303658	5,9423655	3,58405E-10	1,81438E-09
HLTF	1,341762725	6,443624502	3,58763E-10	1,81484E-09
DDX3X	-0,629086532	8,65950015	3,66914E-10	1,8547E-09
NFATC3	-0,730052254	5,824604017	3,75618E-10	1,89729E-09
NDUFA9	1,055557024	7,038678994	3,81088E-10	1,9235E-09
STX3	-0,87768274	5,881816099	3,8143E-10	1,92379E-09
NVL	0,605798748	5,110778637	3,87909E-10	1,95503E-09
UBE2E3	0,810336575	6,802195903	3,91234E-10	1,97033E-09
ZNF581	1,136372957	4,819281701	3,95822E-10	1,99196E-09
PHB2	1,052241279	8,52125643	4,00265E-10	2,01284E-09
PEA15	-0,738435498	8,191169754	4,05864E-10	2,03949E-09
CCT2	1,194254098	8,217131645	4,08212E-10	2,04978E-09
SLC1A5	1,344222876	8,698866782	4,18515E-10	2,09997E-09
ENOPH1	0,811753328	6,333460481	4,22525E-10	2,11853E-09
DUSP18	-0,759343027	3,583350538	4,3534E-10	2,18118E-09
PUS7L	1,081929236	3,981437022	4,4127E-10	2,20927E-09
TRIM28	0,986250995	8,907753477	4,45039E-10	2,2265E-09
PABPC1L	2,111955698	4,088414029	4,48961E-10	2,24447E-09
ATIC	0,874733536	6,962596956	4,51588E-10	2,25595E-09
NET1	1,316835245	7,691790378	4,68403E-10	2,33824E-09
WDR5	0,906326961	6,245583459	4,75288E-10	2,37088E-09
KPNA4	0,897957702	7,800265945	4,76421E-10	2,37479E-09
KDM5B	0,987424694	7,401242779	4,77321E-10	2,37754E-09
CIRBP	-0,843318232	7,558123431	4,83585E-10	2,40698E-09
FTO	-0,659340141	6,089547483	4,84024E-10	2,40741E-09
SCNM1	0,962959357	5,49524453	4,97588E-10	2,47307E-09
NIP7	0,951196808	5,980604149	5,16193E-10	2,56367E-09
FGFR1OP	1,026103573	3,759318275	5,16589E-10	2,56377E-09
MAN2B2	-0,85626876	6,404591914	5,17327E-10	2,56557E-09
PSMB5	0,95122068	7,553391181	5,31901E-10	2,63592E-09
ETF1	-0,538327446	7,635690262	5,64057E-10	2,79325E-09
KRT19	2,277995609	10,29956359	5,64568E-10	2,79375E-09
FANCF	1,014660829	4,736141876	5,68383E-10	2,81059E-09
C10orf76	-0,587791695	5,39348421	5,71303E-10	2,82298E-09
VPS25	0,819459483	6,386793586	5,72899E-10	2,82882E-09
ATXN1L	-0,798541002	6,118712963	5,88471E-10	2,90361E-09
SEMA4B	1,963239346	8,581297311	5,89718E-10	2,90765E-09
MYNN	1,310807874	6,067561381	5,98459E-10	2,94862E-09
ANAPC5	0,744294166	7,699151209	6,02204E-10	2,96493E-09
RBBP8	1,412047734	6,834178199	6,06789E-10	2,98535E-09
ELF1	-0,675518076	6,646410753	6,24269E-10	3,06913E-09
RPL35A	1,340198386	10,15549203	6,25564E-10	3,07329E-09
USP28	1,078442553	5,809128541	6,26205E-10	3,07422E-09
RPS6KA3	-0,905378512	6,913807393	6,28676E-10	3,08413E-09
SATB1	-1,043779832	4,807163035	6,33403E-10	3,10508E-09
SSRP1	0,789073845	8,357942815	6,37406E-10	3,12247E-09
LIPA	-1,132748868	7,773464597	6,39163E-10	3,12882E-09
GEMIN6	1,090579991	4,019800566	6,56935E-10	3,21352E-09
ADCY7	-1,024731777	5,906726136	6,59176E-10	3,22217E-09

RASAL2	-0,898486897	5,580309427	6,72417E-10	3,28454E-09
SEPP1	-1,531149625	8,573865392	6,83091E-10	3,334E-09
RAB10	0,803022869	8,522563303	6,83522E-10	3,334E-09
F13A1	-1,603760713	6,149166794	6,84648E-10	3,33711E-09
PSMD4	0,751153099	7,802054303	6,95447E-10	3,38732E-09
TFE3	-0,570344498	6,514805161	7,08133E-10	3,44665E-09
CASD1	-0,855581487	4,924222084	7,25224E-10	3,52732E-09
GNA11	-0,793880168	5,141692927	7,36778E-10	3,58096E-09
EMG1	1,314733296	6,355851479	7,44929E-10	3,618E-09
ANO10	-0,649708525	5,126892317	7,66746E-10	3,72131E-09
ABCE1	0,685921174	7,090230763	7,67407E-10	3,72187E-09
FBF1	0,950383784	5,302600121	7,72238E-10	3,74264E-09
EIF2B5	1,346553609	7,142356273	7,75592E-10	3,75622E-09
TIA1	1,032482925	6,696698493	7,85554E-10	3,80177E-09
BTN3A2	-1,113784938	6,283859705	8,0529E-10	3,89452E-09
ATP5B	0,759310709	10,01634042	8,08702E-10	3,90825E-09
YEATS2	1,517866005	7,437049726	8,12775E-10	3,92515E-09
C3orf17	0,839390601	6,398613556	8,38565E-10	4,04683E-09
DEK	0,828140597	7,992813966	8,39987E-10	4,05083E-09
SIRT1	-0,74981633	4,869022004	8,44631E-10	4,07035E-09
SLC43A3	-1,363572742	6,252322156	8,70165E-10	4,19043E-09
EIF2S2	0,67589019	7,779500915	8,81681E-10	4,2429E-09
GLIS2	-1,187847499	5,248736732	9,06447E-10	4,359E-09
UTP18	0,777211864	6,191288323	9,31094E-10	4,47437E-09
JAG1	1,901699527	8,589042702	9,36229E-10	4,49588E-09
FRRS1	1,630908158	4,922046459	9,51613E-10	4,56654E-09
ZNF639	1,303361786	6,535658503	9,54633E-10	4,57781E-09
ISCU	-0,671052778	6,411678735	9,70249E-10	4,64942E-09
CXorf40B	0,692125576	5,027000369	9,75794E-10	4,67271E-09
SMO	2,131647991	5,994390922	9,76943E-10	4,67493E-09
LIG3	1,057937416	5,778573318	9,85461E-10	4,71238E-09
PDSS1	1,103296371	3,442783168	9,92247E-10	4,74151E-09
CLK2	0,798890179	5,589894009	9,98383E-10	4,76749E-09
NAA10	0,954731772	6,234044551	1,03017E-09	4,91583E-09
C22orf13	-0,593172583	7,111722122	1,0364E-09	4,94212E-09
PPP4C	0,941828534	7,396240095	1,04583E-09	4,98361E-09
RGPD8	-0,758623835	3,097276495	1,05773E-09	5,03677E-09
C10orf11	-1,261611384	2,752155614	1,06002E-09	5,04415E-09
LYPLA1	1,494734482	7,765870794	1,06917E-09	5,08414E-09
FKBP5	-1,246482314	6,847547062	1,07769E-09	5,12109E-09
SNX29	-0,940111276	5,249216846	1,08012E-09	5,12909E-09
MAPK9	-0,638509442	5,713384058	1,08544E-09	5,15072E-09
NDEL1	-0,660108832	5,850501518	1,10394E-09	5,23489E-09
ZNF326	0,758530928	4,843543027	1,11582E-09	5,28754E-09
ISG20L2	0,684355608	6,141407606	1,11713E-09	5,29007E-09
AUP1	0,81476664	7,880038329	1,15424E-09	5,462E-09
PDPK1	-0,731399303	6,04788196	1,17123E-09	5,53857E-09
PSMD3	0,844744501	7,979617214	1,17534E-09	5,55415E-09
TBCE	0,95649142	5,264592478	1,17665E-09	5,55652E-09
TATDN1	0,93952828	4,954822295	1,20035E-09	5,66451E-09

PTDSS1	1,364235395	8,204730403	1,20988E-09	5,7055E-09
BAZ2B	-0,744739327	5,266176267	1,22287E-09	5,76279E-09
ARHGAP15	-1,361921112	3,426511532	1,2365E-09	5,82301E-09
IQCB1	1,210114882	5,752093986	1,24939E-09	5,87641E-09
BCS1L	0,863603297	4,662578203	1,24957E-09	5,87641E-09
ABCB6	1,71329983	6,593177247	1,27347E-09	5,98469E-09
STK4	-0,640932497	6,240307551	1,28785E-09	6,04808E-09
MRPS11	0,870427049	5,428612839	1,29182E-09	6,06259E-09
TCEB3	-0,602824365	6,667573443	1,31348E-09	6,16E-09
ADSS	0,818730252	7,00060467	1,33074E-09	6,23439E-09
RAD9A	0,962748519	4,36651788	1,33117E-09	6,23439E-09
METTL2B	0,767634842	5,115262282	1,38053E-09	6,4611E-09
NUP35	0,869906654	4,468261366	1,39542E-09	6,5263E-09
RPS2	0,907768326	11,00039347	1,40159E-09	6,54865E-09
GLRX	-1,288081174	5,128531148	1,40212E-09	6,54865E-09
PSME4	0,760874414	7,497324926	1,42733E-09	6,66183E-09
NUDCD1	0,933162799	5,592645978	1,44727E-09	6,75029E-09
PMM2	0,751511595	6,184389441	1,45664E-09	6,78934E-09
CRTAP	-0,783923518	7,883877316	1,48961E-09	6,93364E-09
C12orf52	0,760233628	5,031755588	1,48963E-09	6,93364E-09
NOP16	1,016444467	4,847017842	1,51492E-09	7,04655E-09
ARSB	-0,781666624	4,983724982	1,52744E-09	7,09993E-09
FRMD4A	-1,273233512	4,929723068	1,54412E-09	7,17261E-09
TBRG1	-0,552667163	5,557206634	1,57668E-09	7,31884E-09
PRPF4	1,01043947	6,358514574	1,59906E-09	7,4177E-09
IGF2BP2	2,49341714	6,815509496	1,60593E-09	7,4445E-09
RNPEPL1	-0,715069002	6,864904234	1,60854E-09	7,45151E-09
TMEM99	1,27006029	4,692347461	1,61023E-09	7,45428E-09
TAF3	-0,694546571	3,622979225	1,63297E-09	7,55444E-09
EPB41L3	-1,444131928	5,364104169	1,63488E-09	7,55815E-09
FOXO1	-0,909681221	5,831287314	1,65186E-09	7,63146E-09
MRPS16	0,847726365	7,191883569	1,67239E-09	7,72108E-09
GFM1	0,798910659	6,818587494	1,69444E-09	7,81756E-09
RPE	0,808252902	5,891502353	1,71011E-09	7,88454E-09
TMEM41A	1,067632109	6,711343907	1,71995E-09	7,92453E-09
CLDN1	3,402182178	9,832496449	1,73521E-09	7,98948E-09
FKBP3	1,040159377	6,339709286	1,74109E-09	8,01115E-09
SLBP	0,953707992	6,51551585	1,79256E-09	8,23809E-09
SON	-0,70560596	8,106729544	1,79283E-09	8,23809E-09
DHFR	1,047945979	6,139097119	1,79609E-09	8,24748E-09
CST3	-0,935413204	8,020442432	1,81401E-09	8,3242E-09
LCP2	-1,264880277	5,582941477	1,82895E-09	8,3871E-09
SMS	1,304031486	7,623996727	1,85215E-09	8,48779E-09
ABCF3	1,181282678	7,143827866	1,8734E-09	8,57938E-09
TAF1D	0,949911169	5,687859263	1,87533E-09	8,58247E-09
DUT	1,035114037	6,342627532	1,88234E-09	8,60875E-09
ZNF107	1,035429819	3,919997343	1,89307E-09	8,65204E-09
RHOBTB1	-1,065692402	4,368557153	1,91609E-09	8,75137E-09
TFDP1	1,106998822	7,42002935	1,92812E-09	8,80046E-09
SNRPD3	0,791694309	7,083000579	1,96685E-09	8,9688E-09

DNAJC3	-0,70866812	6,962383059	1,96764E-09	8,9688E-09
RPS19	1,223376956	10,39889119	1,98681E-09	9,05015E-09
IGFBP3	2,853801182	9,690741904	1,99764E-09	9,09339E-09
AP1S2	-1,04600382	5,062258172	2,00999E-09	9,14351E-09
MARCH8	-0,887801591	3,860702443	2,01639E-09	9,16651E-09
PGM2	1,226801262	6,491651525	2,04502E-09	9,29047E-09
PDXP	1,204262099	3,704523536	2,06178E-09	9,36039E-09
NDUFB9	1,154183067	7,884827428	2,0662E-09	9,37421E-09
PRMT5	0,86159332	6,86664845	2,07243E-09	9,39621E-09
ERAL1	0,769117117	6,401424181	2,07684E-09	9,40994E-09
TMEM115	-0,518908354	5,659289164	2,11389E-09	9,57145E-09
PSMA4	0,869541322	7,759500489	2,17377E-09	9,83102E-09
SMPD4	0,725430069	7,008283766	2,1741E-09	9,83102E-09
TMOD3	-0,641028181	7,094943278	2,20089E-09	9,94557E-09
MGAT4A	-1,207035267	5,658318039	2,26175E-09	1,02138E-08
PLP2	1,231629517	8,348552992	2,32731E-09	1,05029E-08
PIAS1	-0,570720115	4,935032844	2,33506E-09	1,05309E-08
C2orf49	0,912764194	3,752292243	2,36061E-09	1,06391E-08
MRPL21	1,2114605	5,736878142	2,43026E-09	1,09458E-08
SNRNP70	0,703845257	7,241269361	2,45268E-09	1,10395E-08
SDC1	1,585103883	10,2732614	2,5129E-09	1,13031E-08
TRAF4	1,195994068	6,973830594	2,51476E-09	1,1304E-08
VMA21	0,799654774	6,743462571	2,5607E-09	1,15029E-08
AHSA1	0,721517575	7,198857762	2,65033E-09	1,18977E-08
C10orf116	-1,864851832	5,66688134	2,69461E-09	1,20885E-08
PSMB3	1,102046521	7,608829494	2,71251E-09	1,21608E-08
CHIC1	-0,782927788	3,63742179	2,71889E-09	1,21814E-08
CEP78	0,894782464	4,359131741	2,7515E-09	1,23194E-08
SLC26A6	1,042202276	4,135093345	2,79958E-09	1,25265E-08
MFAP2	2,026036555	6,575930518	2,81393E-09	1,25824E-08
ERGIC2	0,943791709	6,240870329	2,8346E-09	1,26665E-08
NAA20	1,190451372	6,993339479	2,84076E-09	1,26857E-08
TRUB1	0,907665703	5,454739577	2,89642E-09	1,29252E-08
FAM58A	1,08797637	5,359199265	2,89818E-09	1,29252E-08
WDR18	0,924268529	5,638215749	2,94091E-09	1,31073E-08
TRIM21	-0,791608741	5,066491969	2,97706E-09	1,32597E-08
TBC1D1	-0,892912042	5,865356502	2,98121E-09	1,32695E-08
CDC42BPA	-0,883781548	6,03218107	2,99539E-09	1,33239E-08
CBX4	1,002683006	5,45593945	3,11117E-09	1,38299E-08
WAPAL	-0,520222086	6,344691716	3,11994E-09	1,38551E-08
DDX10	0,888706704	5,747625252	3,12089E-09	1,38551E-08
EIF2C4	-0,800460289	4,612161956	3,13751E-09	1,39198E-08
MEN1	0,740843058	5,91230877	3,14864E-09	1,39601E-08
C3orf18	-1,021831969	2,4319679	3,18535E-09	1,41136E-08
CCDC51	1,15389273	4,845343071	3,1915E-09	1,41317E-08
PTBP1	0,576076484	8,517142758	3,22718E-09	1,42804E-08
UBL4A	0,713791321	6,577986206	3,24197E-09	1,43366E-08
ACP1	0,798457039	7,081153931	3,32771E-09	1,47062E-08
TMEM138	0,598807237	5,433176929	3,39904E-09	1,50083E-08
MRPS9	0,681011621	5,22547297	3,40048E-09	1,50083E-08

ZYX	-0,916873075	8,095459894	3,47541E-09	1,53291E-08
RASA3	-1,098090858	4,656623385	3,49234E-09	1,53938E-08
ESYT1	-0,61016774	7,653217758	3,56882E-09	1,57208E-08
GAN	1,373769302	2,949112645	3,58559E-09	1,57845E-08
COX6A1	1,168150288	8,265073969	3,62455E-09	1,59457E-08
PFDN2	1,118922041	6,357056951	3,73718E-09	1,64306E-08
ANKZF1	0,79988321	5,418976836	3,84367E-09	1,68879E-08
MTX1	0,832594181	5,672549568	3,85026E-09	1,6906E-08
ICT1	1,188893679	5,221838487	3,86017E-09	1,69386E-08
MRPS23	1,097542899	6,369690704	3,86695E-09	1,69574E-08
AP2M1	1,187962491	9,937105522	3,88544E-09	1,70275E-08
DPH3	-0,791129289	5,301957794	3,91013E-09	1,71247E-08
ATP5G3	1,111130921	8,23991158	3,93378E-09	1,72172E-08
MRPS17	1,3877318	4,817805182	3,94073E-09	1,72366E-08
HSD17B10	0,868014707	6,774456974	3,97133E-09	1,73593E-08
HAT1	0,710129945	6,458043979	4,01355E-09	1,75326E-08
STRBP	1,331865438	5,94627272	4,14243E-09	1,8084E-08
HMGB2	1,117788218	7,183104244	4,16457E-09	1,8169E-08
ANKRD12	-0,742318555	5,615467071	4,17299E-09	1,81941E-08
FAM100B	1,008038784	6,476408781	4,27743E-09	1,86376E-08
BAK1	1,167686939	6,438569137	4,39195E-09	1,91243E-08
PSMA2	0,857104477	7,629190418	4,42201E-09	1,92429E-08
HABP4	-0,985826962	3,848516095	4,46447E-09	1,94153E-08
RGPD6	-0,705710296	4,751114292	4,48319E-09	1,94843E-08
ASAP3	-0,950289126	4,949454237	4,50886E-09	1,95834E-08
POLDIP2	0,70106631	7,534351719	4,6853E-09	2,03368E-08
DOCK10	-1,295733351	4,595195073	4,69689E-09	2,03741E-08
ZNF280C	1,169481609	3,938805835	4,74889E-09	2,05866E-08
TSEN15	1,151150324	6,355023296	4,8356E-09	2,09491E-08
MGC57346	0,980673908	4,216482079	4,90679E-09	2,12441E-08
SCARB2	-0,723843483	8,453031197	4,93059E-09	2,13336E-08
TRMT112	0,894438467	7,643778601	4,9443E-09	2,13793E-08
NONO	0,667676901	9,182355926	4,9696E-09	2,14751E-08
ACAA2	-1,233305227	5,912309096	4,98583E-09	2,15316E-08
SYNRG	-0,602417611	6,03502144	4,99953E-09	2,15771E-08
DHX36	0,884637283	6,773135505	5,03585E-09	2,17109E-08
DLG1	1,019575035	7,397700835	5,03691E-09	2,17109E-08
SNHG11	0,943867385	3,533954757	5,09148E-09	2,19323E-08
POLR2F	1,018340041	5,99525018	5,17578E-09	2,22813E-08
MLKL	-1,047966577	4,138099779	5,22754E-09	2,249E-08
MTMR6	-0,790615311	5,824054617	5,25769E-09	2,26054E-08
IL10RA	-1,31881445	5,575156835	5,30546E-09	2,27964E-08
PHTF2	0,948667842	5,995426297	5,31944E-09	2,28421E-08
GCA	-0,966301947	5,103925731	5,47122E-09	2,34791E-08
GATC	0,990565979	2,660915399	5,52546E-09	2,36969E-08
MORC2	0,902142411	6,447254291	5,6288E-09	2,4125E-08
CSF1R	-1,313299925	6,498879178	5,644E-09	2,41749E-08
PLXNA2	-1,359484157	6,237564935	5,65001E-09	2,41854E-08
ELN	-1,813938269	6,455438591	5,75474E-09	2,46183E-08
COPS5	0,783561941	6,523417148	5,76593E-09	2,46507E-08

FAM20A	-1,330534237	5,057770464	5,81347E-09	2,48384E-08
CHD2	-0,714786726	6,863573755	6,02732E-09	2,57359E-08
PCSK7	-0,66114113	5,511739147	6,06412E-09	2,58769E-08
MARS2	1,029848478	4,150404841	6,09069E-09	2,5974E-08
RAB5A	-0,554431439	6,092354332	6,16664E-09	2,62815E-08
FYTTD1	0,937093188	7,650855013	6,2545E-09	2,66393E-08
INO80B	0,714495749	5,322430141	6,30861E-09	2,68529E-08
NDUFA4L2	2,973177962	8,067510527	6,32475E-09	2,69048E-08
C10orf35	1,187988467	4,106403958	6,53693E-09	2,77901E-08
TIPARP	-1,191086226	6,693729557	6,55243E-09	2,78386E-08
ALDOC	1,643793866	6,521955308	6,65173E-09	2,8243E-08
ABI3	-1,176475711	4,138665695	6,79853E-09	2,88483E-08
SBDSP1	-0,649408445	4,968118558	6,81586E-09	2,89038E-08
RRP1	0,889868396	5,546337929	6,83206E-09	2,89516E-08
U2AF2	0,717270667	7,855807933	6,83561E-09	2,89516E-08
PCNX	-0,736911033	6,183571122	6,8617E-09	2,9044E-08
RYK	0,959593702	7,22641891	6,87787E-09	2,90944E-08
IMP4	0,879220035	6,072686377	6,9266E-09	2,92824E-08
UBE2O	0,865754862	6,19063727	6,95671E-09	2,93915E-08
TSPAN6	1,328893664	6,610692432	6,96612E-09	2,9413E-08
CD63	-0,814055327	9,50806858	7,03145E-09	2,96705E-08
LRRFIP2	-0,612918526	5,561008511	7,04283E-09	2,97001E-08
PIAS3	0,932698935	6,41366584	7,06612E-09	2,97799E-08
ANKMY1	-0,772937489	3,52399303	7,13705E-09	3,00602E-08
EIF4EBP2	-0,686696007	7,799946365	7,16113E-09	3,0143E-08
GPSM2	0,961529768	5,531447564	7,21017E-09	3,03308E-08
PRDX4	1,282518594	7,511203514	7,24151E-09	3,04438E-08
GOLGA4	-0,781299992	6,548979696	7,30281E-09	3,06826E-08
CAND1	0,868487823	7,931654067	7,42962E-09	3,11962E-08
NDE1	1,043497401	5,870748392	7,54176E-09	3,16381E-08
C5orf22	0,853513386	5,933167751	7,54416E-09	3,16381E-08
STAT3	-0,721582583	8,65818811	7,61513E-09	3,19161E-08
TMEM43	-0,616099439	7,416377933	7,79069E-09	3,26319E-08
AMMECR1	1,248287451	5,480107403	7,90753E-09	3,31009E-08
SNTB1	-1,536754549	5,45817702	7,93804E-09	3,32083E-08
SUN2	-0,795993106	7,786115859	7,99899E-09	3,34428E-08
SAE1	0,766555773	7,485462996	8,18E-09	3,41786E-08
LIX1L	-0,954627138	5,197098172	8,21409E-09	3,43E-08
SMC6	1,076371046	6,181733753	8,26932E-09	3,45095E-08
TIMM17B	1,120826165	5,972288294	8,27467E-09	3,45107E-08
FGL2	-1,396954384	5,680618075	8,35043E-09	3,48053E-08
POLD1	1,058469033	5,709109557	8,45294E-09	3,52111E-08
UBE2M	0,814196122	6,539267782	8,47278E-09	3,52722E-08
POLE	1,140745257	5,979881141	8,49909E-09	3,53601E-08
ERH	0,946438422	7,280406157	8,52258E-09	3,54363E-08
SRM	0,868428715	6,55872455	8,57639E-09	3,56383E-08
ZNF706	0,831029057	6,940937927	8,66201E-09	3,59721E-08
CDC123	0,715183497	6,557918531	8,68184E-09	3,60326E-08
COX8A	0,890723235	7,583411202	8,89713E-09	3,69036E-08
TRPT1	0,863874028	4,81962256	8,95558E-09	3,71235E-08

ZNHIT3	0,836823134	5,581136355	9,00876E-09	3,73212E-08
ZC3H15	0,700538855	6,892665605	9,06606E-09	3,75295E-08
S100A16	1,703580087	8,715999647	9,07004E-09	3,75295E-08
MYL12A	-0,778805511	8,737780832	9,0994E-09	3,76282E-08
CBR3	2,931693923	5,454248201	9,24318E-09	3,81995E-08
COX5B	1,283967277	7,439633307	9,33671E-09	3,85627E-08
MTA2	0,683532775	7,466757712	9,83661E-09	4,06028E-08
TEX10	0,906539396	5,405341875	9,9821E-09	4,11784E-08
GTF2E2	0,985948864	5,884137162	1,00309E-08	4,13548E-08
MTG1	0,986496082	5,272355107	1,02754E-08	4,23373E-08
SPINT2	1,390172337	9,736872963	1,03804E-08	4,2744E-08
MICAL1	-1,069071303	5,241183407	1,03916E-08	4,27642E-08
RNF34	0,57652766	5,436322239	1,04614E-08	4,30257E-08
ZNF217	0,935895837	6,721915089	1,05239E-08	4,32565E-08
ERCC5	-0,696630656	5,701088231	1,06346E-08	4,36852E-08
WDR76	1,436861866	4,572563199	1,06549E-08	4,37421E-08
HOMER3	1,139094717	5,839968035	1,07464E-08	4,40915E-08
RAD51C	0,861179917	4,440402692	1,07755E-08	4,41842E-08
USP21	0,816180015	5,40046323	1,08115E-08	4,43052E-08
ITPKC	-1,10621457	6,869360036	1,08193E-08	4,43103E-08
NME4	1,152885707	6,724350694	1,08263E-08	4,43126E-08
NRARP	1,931259347	5,743873577	1,08466E-08	4,4369E-08
GNPNAT1	0,965077927	6,147902616	1,09876E-08	4,49186E-08
MAGEF1	1,418179129	6,761419204	1,0999E-08	4,49385E-08
TRMT1	1,021211736	5,418847762	1,13521E-08	4,63388E-08
TTYH3	1,118027318	7,562565707	1,13554E-08	4,63388E-08
UBXN2A	0,857457658	4,999116197	1,1371E-08	4,63749E-08
BRI3	-0,732946886	6,980204965	1,15521E-08	4,70853E-08
TOR3A	1,039992434	7,005294077	1,15791E-08	4,7167E-08
EBNA1BP2	0,846249083	6,567241541	1,20918E-08	4,92262E-08
WSB1	-0,793353472	7,070897397	1,2153E-08	4,9446E-08
PBRM1	-0,636438636	6,056899654	1,22108E-08	4,96515E-08
COPB2	0,796851141	8,415671701	1,23489E-08	5,01831E-08
SEPN1	-0,729012177	7,817784323	1,23947E-08	5,03392E-08
C3orf37	0,904864665	6,874482103	1,30893E-08	5,31285E-08
MRPL24	1,08714355	6,486928992	1,34393E-08	5,45167E-08
THOC6	0,838578658	5,488043152	1,34732E-08	5,46216E-08
RNH1	-0,66465344	7,777552292	1,36984E-08	5,55018E-08
MTA1	0,958484926	6,216024167	1,3775E-08	5,57789E-08
ID2	-1,19299627	5,948522911	1,38548E-08	5,60687E-08
ZW10	0,804675589	5,251220697	1,40425E-08	5,67947E-08
IARS	0,850683707	7,876497407	1,42692E-08	5,76775E-08
TMEM54	1,135706287	6,633411842	1,43083E-08	5,78009E-08
TMEM30A	-0,663318442	8,244255187	1,45853E-08	5,88852E-08
PFDN6	0,946792375	5,317893214	1,49938E-08	6,04986E-08
TSEN54	0,920073532	5,518117657	1,50679E-08	6,07619E-08
DUS1L	0,93513861	6,79113329	1,53866E-08	6,20101E-08
ARMC1	0,852469162	6,111848785	1,53958E-08	6,20107E-08
C1orf174	0,700500315	5,364478708	1,55714E-08	6,26811E-08
NUDT16	-0,768524106	5,418420575	1,59062E-08	6,39908E-08

APPL1	-0,517188185	6,134906461	1,60253E-08	6,44321E-08
ERI2	0,842463379	4,662419465	1,6235E-08	6,52366E-08
PDE12	-0,466336881	5,090354253	1,63985E-08	6,58547E-08
BPHL	1,072076143	4,715996415	1,66179E-08	6,66964E-08
ACTR1A	-0,601264688	7,266629754	1,66309E-08	6,67093E-08
KIAA0247	-0,714280466	7,346129438	1,69712E-08	6,80343E-08
SENP2	1,151636265	6,765812563	1,69871E-08	6,80581E-08
NDUFAB1	0,9012361	6,335556922	1,70588E-08	6,83054E-08
DDT	1,099709065	6,406313778	1,74264E-08	6,97364E-08
C17orf89	1,13903688	4,629774955	1,83489E-08	7,33847E-08
SGMS1	-0,824336625	5,81126571	1,86373E-08	7,44947E-08
TRIM5	-0,750871151	4,987787975	1,87658E-08	7,49642E-08
GCLM	2,493867667	7,161726287	1,90126E-08	7,59057E-08
MFSD3	1,199535897	4,439317978	1,9036E-08	7,59545E-08
TCEAL3	-0,849202573	4,293474056	1,9111E-08	7,62095E-08
AVL9	0,84295743	5,092615998	1,94932E-08	7,76879E-08
TXNDC17	1,239151044	6,264152617	1,95328E-08	7,78004E-08
OGDH	-0,666030473	7,634966345	1,99732E-08	7,95081E-08
ATRX	-0,701693502	6,264347259	2,00457E-08	7,975E-08
WDR48	-0,535494618	5,491267943	2,00966E-08	7,99058E-08
PILRB	1,35358681	5,592781643	2,01506E-08	8,00741E-08
USP14	0,722626893	6,978269024	2,09569E-08	8,32296E-08
MARCKSL1	1,368253257	7,82075958	2,12783E-08	8,44567E-08
DDX24	-0,524655388	7,540475632	2,1761E-08	8,63225E-08
CYCS	1,049857607	8,185948728	2,21523E-08	8,78236E-08
PRKDC	1,096313104	8,650074318	2,23578E-08	8,85868E-08
PRDX2	1,282547852	8,244495995	2,27135E-08	8,99437E-08
OTUD6B	0,880379436	4,67473947	2,27595E-08	9,00739E-08
LRP10	-0,790478397	8,043053749	2,28582E-08	9,0412E-08
RPS6KB2	0,903299206	6,234161193	2,30818E-08	9,12096E-08
WBSCR22	0,759258389	6,918624642	2,30866E-08	9,12096E-08
TNIP1	-0,656187379	7,462449623	2,31627E-08	9,14571E-08
CEBPD	-1,06529769	6,546932999	2,35864E-08	9,30763E-08
MDFI	1,919567429	5,586826844	2,38173E-08	9,39329E-08
H1FX	1,112969009	7,565927924	2,40451E-08	9,47765E-08
PSMD8	1,016300947	8,209661343	2,45773E-08	9,68185E-08
SENP3	0,669283322	6,185445343	2,49974E-08	9,84166E-08
JUNB	-1,179110395	8,076865869	2,51694E-08	9,90365E-08
NOP58	0,85799876	6,768744347	2,5271E-08	9,9379E-08
UQCRC1	0,652380448	7,866846673	2,55311E-08	1,00344E-07
ARF5	0,887866103	7,906195874	2,5751E-08	1,0115E-07
MFAP3	-0,562886093	5,714949878	2,60152E-08	1,0209E-07
CTNNAL1	-1,157793797	6,148823223	2,60203E-08	1,0209E-07
UTP6	0,708009447	6,055292471	2,63255E-08	1,03228E-07
USP4	-0,548053557	6,227330279	2,64376E-08	1,03608E-07
DCAF17	0,666228967	5,005275316	2,65486E-08	1,03983E-07
BNIP2	-0,506913387	6,085870916	2,65723E-08	1,04016E-07
NUDT5	0,818217702	6,053293707	2,71414E-08	1,06183E-07
DONSON	0,839430152	5,512332227	2,72483E-08	1,0654E-07
CDK5	0,930007249	4,724744487	2,75955E-08	1,07836E-07

MRPS7	0,988555061	6,942059892	2,76295E-08	1,07907E-07
XYLT2	0,87189557	5,993425421	2,81176E-08	1,09743E-07
TXN	1,666740821	8,72308835	2,81317E-08	1,09743E-07
TMEM134	1,078773774	5,689695195	2,81769E-08	1,09856E-07
PDLIM4	1,806498447	5,225214635	2,82646E-08	1,10135E-07
NANP	0,829338632	4,272851695	2,85116E-08	1,11034E-07
CD86	-1,130855832	4,767828406	2,87763E-08	1,12001E-07
CLDND1	1,25431331	7,709070365	2,90298E-08	1,12923E-07
ZFPL1	0,708886697	5,424491468	2,91444E-08	1,13304E-07
LAPTM4B	1,162686419	8,577993472	2,93831E-08	1,14167E-07
ZNF485	0,987862242	1,980558626	2,9796E-08	1,15705E-07
COTL1	-0,976612573	7,576555449	2,99256E-08	1,16143E-07
ZFYVE9	-0,795182086	5,338497155	3,00555E-08	1,1658E-07
ALKBH4	0,717196161	4,151971877	3,02143E-08	1,1713E-07
MTMR1	0,894426521	6,289681699	3,07724E-08	1,19225E-07
TCEB1	0,887903157	6,383652219	3,08791E-08	1,19571E-07
TIGD1	1,006130434	4,093108531	3,09367E-08	1,19726E-07
FAM3C	1,251738284	7,791040735	3,10101E-08	1,19942E-07
FAM174A	-0,71431054	4,344047352	3,11096E-08	1,20259E-07
ZFYVE26	-0,792323962	5,611655953	3,14982E-08	1,21692E-07
ERI3	0,786221625	6,43205967	3,17793E-08	1,22708E-07
ARPC1A	0,921236372	8,124150494	3,21179E-08	1,23946E-07
PIP4K2A	-0,872120003	5,821165428	3,25151E-08	1,25407E-07
CHCHD2	1,256980293	8,901951588	3,28368E-08	1,26577E-07
MAPK12	1,42551549	4,959313755	3,30068E-08	1,2716E-07
MRPL19	0,595908916	6,386425655	3,32206E-08	1,27912E-07
MCM3	1,03863884	7,560050673	3,41639E-08	1,31469E-07
NFIC	-0,963291427	5,367450316	3,43515E-08	1,32117E-07
IER5L	1,656994147	4,328119479	3,44699E-08	1,32497E-07
BRCA2	1,177891322	3,658770449	3,50994E-08	1,34841E-07
ATG2B	-0,678390316	5,288048913	3,54561E-08	1,36134E-07
C11orf73	0,739287507	5,337539433	3,56999E-08	1,36993E-07
MLST8	0,778560624	5,991765357	3,60662E-08	1,38321E-07
ATP5J2	1,278384564	7,456862175	3,69396E-08	1,41592E-07
ATP13A3	0,887588735	7,890308173	3,72254E-08	1,42607E-07
ZC3HAV1	-0,562740074	6,355182055	3,73972E-08	1,43184E-07
MRPS2	0,766890323	5,890358501	3,7959E-08	1,45254E-07
COX6B1	1,06363582	8,034474038	3,8019E-08	1,45402E-07
TMEM106A	-0,876712424	3,251818192	3,82611E-08	1,46246E-07
RNF2	0,781048491	5,676859859	3,85547E-08	1,47269E-07
APAF1	-0,602988842	4,841976036	3,85721E-08	1,47269E-07
FBXL6	1,081778634	4,77745817	3,91504E-08	1,49394E-07
ANXA11	-0,725420448	8,037688194	3,95496E-08	1,50833E-07
PFKFB3	-0,962096254	7,285278921	3,97919E-08	1,51672E-07
SNAI2	1,733650127	6,17025829	3,98858E-08	1,51886E-07
YKT6	0,712949211	7,460235353	3,98927E-08	1,51886E-07
CIITA	-1,340572249	4,571314909	4,00233E-08	1,52299E-07
C10orf118	-0,725270625	4,860837355	4,04182E-08	1,53716E-07
POLR3K	0,988228428	4,948466512	4,08947E-08	1,55441E-07
GMCL1	0,834737731	5,104017247	4,0988E-08	1,55709E-07

BUD31	0,925725427	6,754645624	4,18969E-08	1,59073E-07
PDXK	-0,836076592	7,398559496	4,22026E-08	1,60144E-07
TSPYL1	-0,588385523	6,999114767	4,22683E-08	1,60304E-07
KIDINS220	-0,72648968	6,959476647	4,27799E-08	1,62155E-07
RAF1	-0,418835267	6,857672677	4,28073E-08	1,62168E-07
DOCK1	-0,781009619	6,268158679	4,28593E-08	1,62275E-07
GORAB	1,020550115	4,498525539	4,36895E-08	1,65327E-07
MTMR10	-0,976630573	5,442746773	4,38762E-08	1,65941E-07
KRTCAP3	1,843960545	5,810496647	4,40461E-08	1,66491E-07
TEAD1	-0,809378374	6,494598477	4,48088E-08	1,6928E-07
PRKCA	-1,031143993	4,908551129	4,71349E-08	1,7797E-07
RASSF5	-0,942674097	6,02598433	4,74522E-08	1,79068E-07
MRPL53	0,908326466	4,441158528	4,79288E-08	1,80767E-07
PFDN4	1,176246658	5,031311638	4,81863E-08	1,81638E-07
CLIC4	-0,793103771	8,358521269	4,87203E-08	1,83548E-07
COPS6	0,753393515	7,220728312	4,87468E-08	1,83548E-07
DYNC2H1	-1,132404844	4,155787893	4,89433E-08	1,84186E-07
NCF4	-1,19261726	4,21101709	4,91851E-08	1,84993E-07
ACPL2	1,262547577	5,191643074	4,94717E-08	1,85969E-07
ITPR2	-1,132550968	5,485228228	4,9968E-08	1,87731E-07
TMEM45A	1,35727282	7,782466868	5,06126E-08	1,90048E-07
CCAR1	0,567646407	6,334249176	5,22134E-08	1,95951E-07
PSMA5	0,812944636	7,198901214	5,25725E-08	1,9719E-07
MC1R	1,557058195	2,76840459	5,26629E-08	1,97421E-07
ACTR3	0,781624665	8,982814651	5,35225E-08	2,00533E-07
C22orf28	0,710998864	7,480406355	5,37841E-08	2,01402E-07
SULF1	1,891381243	8,118137995	5,65465E-08	2,1163E-07
TBPL1	1,070926632	5,077077438	5,6665E-08	2,11957E-07
USP47	-0,672937977	6,474266057	5,75277E-08	2,15067E-07
PSMC6	0,858485046	7,093671991	5,83982E-08	2,18201E-07
ZNF273	0,941960464	4,150338518	5,84667E-08	2,18338E-07
CNTROB	-0,652578815	5,456261951	5,88671E-08	2,19713E-07
PNKP	0,810533059	5,336980811	5,94245E-08	2,21672E-07
UHRF1BP1L	-0,544448471	4,832901059	5,94951E-08	2,21814E-07
ARIH2	-0,397494509	6,228634493	5,96076E-08	2,22112E-07
NFIB	-1,187412411	6,371525557	5,96635E-08	2,22198E-07
VBP1	0,830626803	6,459055594	6,09105E-08	2,26719E-07
DNAJB12	-0,523374918	6,007014845	6,22569E-08	2,31604E-07
ACBD6	0,727521996	5,688019102	6,28493E-08	2,3368E-07
GIMAP2	-1,187032343	3,789909593	6,39969E-08	2,37817E-07
NOC3L	0,713734797	5,068871808	6,47146E-08	2,40353E-07
PTCD3	0,706044119	6,518571751	6,48339E-08	2,40665E-07
MRPL38	0,918584575	5,726177572	6,60143E-08	2,44914E-07
MRC2	-1,202351811	7,355968049	6,81307E-08	2,52628E-07
TRA2B	0,635583196	7,909800142	6,8514E-08	2,53911E-07
NQO1	3,000727149	8,610476697	6,90908E-08	2,5591E-07
KLC2	0,832295669	5,73040932	7,15423E-08	2,64846E-07
SBDS	-0,813719114	6,870696466	7,18693E-08	2,65912E-07
SHOC2	-0,519009919	6,037791783	7,25318E-08	2,68218E-07
HIPK2	-1,027895513	6,559776372	7,3528E-08	2,71703E-07

MCTS1	0,723278041	6,047719292	7,35538E-08	2,71703E-07
FLNA	-1,092297415	10,49811329	7,38551E-08	2,72668E-07
PSMA7	0,840127314	8,28055015	7,41655E-08	2,73538E-07
AKTIP	-0,716157538	5,130622654	7,41709E-08	2,73538E-07
EVC	-1,045278686	4,918032165	7,45284E-08	2,74708E-07
DDX60L	-1,068425728	4,780299842	7,4748E-08	2,75368E-07
DAZAP1	0,565615793	6,900343804	7,49474E-08	2,75954E-07
MRPL48	0,998679331	5,372952858	7,55359E-08	2,77971E-07
CNP	0,593180375	7,190728566	7,6988E-08	2,83161E-07
HS6ST1	1,059334673	7,359382017	7,70428E-08	2,8321E-07
MED19	0,757182891	4,433907528	7,95774E-08	2,9237E-07
JARID2	1,001402246	5,947180039	8,0146E-08	2,943E-07
RBMS1	-0,656009782	7,028825489	8,02116E-08	2,94383E-07
ABCF2	0,694646883	6,903057697	8,18567E-08	3,00258E-07
IGFBP4	-0,944803478	9,274127727	8,37239E-08	3,06943E-07
XPR1	1,11128431	7,285614479	8,4396E-08	3,0924E-07
MKKS	0,82814852	6,247077981	8,56112E-08	3,13524E-07
GLRX5	0,724872102	5,974775386	8,79334E-08	3,21856E-07
NCOR1	-0,772639994	6,700114231	8,81536E-08	3,22489E-07
LGMN	-0,792824849	7,816434322	8,82959E-08	3,22836E-07
PIK3R1	-1,045093655	6,552794914	9,06274E-08	3,31183E-07
ATP8B2	-0,930031028	5,663790707	9,10579E-08	3,32578E-07
UBA6	0,942133958	6,58988895	9,11726E-08	3,32819E-07
SNAPC1	1,117305993	5,329635433	9,2457E-08	3,37327E-07
BNIP3	1,48296415	6,955536572	9,31387E-08	3,39632E-07
TBC1D12	-0,583265382	3,730012818	9,48212E-08	3,45583E-07
C14orf2	1,105495882	6,559578355	9,66757E-08	3,52154E-07
PRKAR2A	-0,709158487	4,492673986	9,7994E-08	3,56765E-07
KIAA1033	-0,538246541	5,957047775	9,80676E-08	3,56842E-07
SERINC3	-0,577242827	7,331124456	9,83645E-08	3,57732E-07
C12orf45	0,829780603	3,708141966	9,88132E-08	3,59172E-07
EFTUD2	0,657747324	7,649928501	9,93019E-08	3,60756E-07
MMADHC	0,651709129	7,28722249	9,97612E-08	3,62232E-07
ATP11B	1,396109443	7,850613081	9,98907E-08	3,62509E-07
MRPL18	0,848331507	6,199954309	1,00431E-07	3,64275E-07
MRPS18A	0,740209056	5,898523052	1,02429E-07	3,71324E-07
PCCB	0,879450245	6,839982985	1,07294E-07	3,88755E-07
HSPA14	0,808919982	5,548899395	1,08716E-07	3,93698E-07
DYNLL1	0,670989075	8,138175571	1,08972E-07	3,94415E-07
WTIP	-1,169595491	3,247573284	1,09041E-07	3,94455E-07
TFAM	0,79945233	5,537654414	1,10741E-07	4,0037E-07
PCNXL2	0,900113118	4,409391742	1,10793E-07	4,0037E-07
DUS3L	0,987398107	5,045919897	1,12156E-07	4,05082E-07
WDR70	0,782039148	5,759722215	1,12944E-07	4,07671E-07
ZNF3	0,708691368	5,728556516	1,12993E-07	4,07671E-07
PITPNB	0,632075402	7,101587089	1,14119E-07	4,11517E-07
IPO4	0,761536548	6,650094621	1,14673E-07	4,13296E-07
BTN2A1	-0,565922404	5,117662722	1,20578E-07	4,34349E-07
ZNF207	0,476531251	7,924296265	1,22231E-07	4,4001E-07
TMED2	0,790566074	9,274050101	1,22279E-07	4,4001E-07

GABARAP	-0,575312057	8,376195979	1,2265E-07	4,41115E-07
HDAC5	-0,704119944	6,143214096	1,23081E-07	4,42428E-07
PAM	-0,943354192	7,562076553	1,25043E-07	4,49244E-07
PCYT1A	1,12018293	6,008701279	1,25494E-07	4,50629E-07
STK17B	-0,859419294	6,741549764	1,26749E-07	4,54897E-07
KIF5B	-0,567940576	7,913588058	1,27832E-07	4,58539E-07
NDRG1	1,915062473	10,99120188	1,29115E-07	4,629E-07
MALL	-1,391400549	7,544493103	1,30796E-07	4,68679E-07
NUDT15	0,798045611	5,476873202	1,33817E-07	4,79149E-07
MAGOH	0,710477728	5,339076932	1,33858E-07	4,79149E-07
XRCC6	0,643857389	9,394712003	1,34448E-07	4,81007E-07
OSBPL5	-0,859346222	4,772065307	1,35891E-07	4,85917E-07
TRMT5	0,6622596	5,044724002	1,36196E-07	4,86752E-07
GLS	-0,832563608	6,915400545	1,36656E-07	4,88139E-07
PSAP	-0,734731312	11,40578597	1,37606E-07	4,91274E-07
ABI2	-0,6797295	6,072201382	1,39346E-07	4,97225E-07
CDK8	0,88958168	4,25945903	1,40184E-07	4,99954E-07
S100A11	1,16301306	10,25208033	1,40383E-07	5,00404E-07
E2F6	0,80444141	5,32731867	1,40738E-07	5,01405E-07
RBM4	0,539688159	7,328105561	1,42172E-07	5,06187E-07
TIAL1	0,587536777	6,644448028	1,42228E-07	5,06187E-07
SRXN1	2,747024682	8,893827194	1,45221E-07	5,16569E-07
ARHGAP10	-0,876557303	4,28145332	1,4742E-07	5,24116E-07
CCT8	0,675020607	8,112936822	1,48896E-07	5,29088E-07
C9orf116	-1,223524655	1,983923101	1,5004E-07	5,32655E-07
POLR1B	0,653737604	5,626829645	1,50056E-07	5,32655E-07
FAM129B	-0,840044955	8,724527427	1,50739E-07	5,34803E-07
SLC7A1	1,086443826	7,007657048	1,50855E-07	5,34936E-07
KIAA1522	0,997060963	8,007974535	1,52079E-07	5,38994E-07
ETFA	0,788951406	7,079451329	1,5236E-07	5,39711E-07
SPHK1	1,493691641	5,398399252	1,54398E-07	5,46646E-07
PSMB2	0,61272826	7,392258371	1,56804E-07	5,54876E-07
NDUFA12	0,689645967	5,866039029	1,59606E-07	5,64498E-07
CHD9	-0,6684037	5,709360012	1,60523E-07	5,67447E-07
TCFL5	0,854969916	4,677785044	1,62766E-07	5,7508E-07
AP2S1	0,792995098	6,962636407	1,63884E-07	5,78727E-07
ELOVL6	1,531210013	4,80344964	1,64431E-07	5,80359E-07
GTF2IRD2B	-0,750431828	4,465175393	1,65702E-07	5,84544E-07
SLC46A1	-0,842316858	4,720571438	1,6604E-07	5,85432E-07
INPP5B	-0,765485282	5,164038762	1,66255E-07	5,85887E-07
G3BP1	0,606830457	7,599503474	1,66915E-07	5,8791E-07
ATR	0,747997739	5,83305844	1,67528E-07	5,89763E-07
YIF1B	0,944572071	6,029580875	1,6804E-07	5,91263E-07
TRIM8	-0,819739715	6,86139762	1,68646E-07	5,93089E-07
SUDS3	0,658476321	6,562015625	1,7108E-07	6,01337E-07
FLCN	-0,566990675	5,533029199	1,7226E-07	6,05174E-07
FARP1	-0,868448167	6,837684099	1,758E-07	6,17292E-07
EIF2A	0,783103069	7,596491121	1,76926E-07	6,20926E-07
ZRANB1	-0,525569898	5,775128648	1,81125E-07	6,35336E-07
SMG6	-0,721293102	5,859031093	1,81822E-07	6,37453E-07

PICALM	-0,494902433	7,933798516	1,83296E-07	6,42291E-07
NHP2	0,83293602	6,504306852	1,841E-07	6,44774E-07
ATXN7L3	0,584172151	6,806821982	1,84721E-07	6,46619E-07
TET1	1,58200366	2,545823002	1,85512E-07	6,49053E-07
TRIB3	1,724214939	6,239040955	1,87257E-07	6,54822E-07
TSPYL2	-0,858918777	5,061300767	1,89519E-07	6,62392E-07
IRF3	0,675197358	6,232881749	1,92089E-07	6,71031E-07
SURF2	0,805513296	4,434924332	1,97218E-07	6,88596E-07
FXYD6	-1,4046485	6,121501021	1,97618E-07	6,89639E-07
NAT10	0,773246569	6,72650622	1,98319E-07	6,91734E-07
VASN	-1,217700972	4,652925453	1,98504E-07	6,92025E-07
TMED5	-0,764868625	6,677247958	2,00772E-07	6,99573E-07
CALM1	-0,711312393	9,130792049	2,02553E-07	7,0542E-07
TTC19	-0,635503006	5,723343781	2,02879E-07	7,06194E-07
FAM57A	1,123081698	5,977259859	2,03888E-07	7,09343E-07
SIK3	-0,639683899	5,684242363	2,04908E-07	7,1253E-07
DUSP16	-0,819878909	6,349990092	2,11265E-07	7,3426E-07
MYO18A	-0,77398568	6,291421699	2,13988E-07	7,43343E-07
EXOC8	-0,503830462	4,840299217	2,156E-07	7,48561E-07
VDAC3	1,17696247	7,64034816	2,164E-07	7,50748E-07
MTA3	0,698090466	5,867401856	2,1645E-07	7,50748E-07
SET	0,626657921	9,179436738	2,16717E-07	7,51156E-07
OSBPL9	-0,544443611	6,759627387	2,16788E-07	7,51156E-07
COPS7B	0,586956082	5,624227897	2,18783E-07	7,57683E-07
ZNF623	0,805725737	5,029994669	2,19156E-07	7,58591E-07
UPK3BL	1,891584946	5,592386011	2,25812E-07	7,81235E-07
NR2C2	-0,524876639	5,610399158	2,26464E-07	7,83094E-07
LTBP3	-0,832878481	7,120780377	2,28576E-07	7,89995E-07
OSGEPL1	0,799871838	3,659347812	2,32953E-07	8,04714E-07
STAB1	-1,079463195	6,074760846	2,38535E-07	8,23518E-07
KBTBD2	-0,526130417	6,350324125	2,38687E-07	8,23518E-07
ERCC3	0,475226134	6,293608373	2,38759E-07	8,23518E-07
HARBI1	0,82000195	2,775457101	2,38971E-07	8,23834E-07
PTPN18	-0,763314735	6,224986311	2,39378E-07	8,24821E-07
DPM1	0,817415481	5,906230276	2,42031E-07	8,3354E-07
SUOX	-0,613302542	4,889093268	2,54983E-07	8,77704E-07
TMEM9B	-0,602254846	6,401647172	2,56264E-07	8,81668E-07
PPID	0,689943455	5,560614926	2,56793E-07	8,83041E-07
MLL3	-0,727082433	6,484262975	2,57235E-07	8,84114E-07
MAPK1IP1L	-0,532658779	7,041791203	2,59413E-07	8,91153E-07
NBR1	-0,556803129	7,48119529	2,59656E-07	8,91538E-07
QSER1	0,838104828	6,304614327	2,61454E-07	8,97258E-07
ZFP36L2	-0,921992626	7,672247784	2,6236E-07	8,99695E-07
VEZF1	-0,531291492	6,532968656	2,62427E-07	8,99695E-07
ARRDC4	-1,020627813	5,339689667	2,63249E-07	9,02058E-07
MARCKS	0,816018826	8,596730485	2,64242E-07	9,04927E-07
SLC38A1	0,982603338	7,831754472	2,64352E-07	9,04927E-07
GSTP1	1,061925891	10,31812837	2,66404E-07	9,11496E-07
TSN	0,622211118	7,260169224	2,70832E-07	9,26181E-07
TIPRL	0,79208229	6,527380988	2,71299E-07	9,27313E-07

SOAT1	-0,728028049	6,091409336	2,74632E-07	9,38197E-07
RARS	0,473931486	6,352825636	2,74759E-07	9,38197E-07
DDX52	0,669010352	5,58800789	2,76849E-07	9,44863E-07
IQGAP1	-0,638311987	8,976733293	2,77539E-07	9,46742E-07
HAS2	-1,587358246	4,224941291	2,7847E-07	9,49444E-07
PTPRF	1,003132878	9,710640984	2,84834E-07	9,70655E-07
GOLT1B	1,049067399	6,882732177	2,88601E-07	9,83002E-07
SAR1A	-0,521186569	7,323989442	2,90508E-07	9,89002E-07
ROMO1	1,294857916	6,132945341	2,9396E-07	1,00026E-06
NRBP1	0,564789789	7,691888234	2,94598E-07	1,00193E-06
AKT1S1	0,672917752	6,074500877	2,95915E-07	1,0059E-06
CWC25	-0,506342974	4,620675298	2,98089E-07	1,01279E-06
DNM1L	0,680578543	6,971693201	3,03067E-07	1,02919E-06
C1orf123	-0,584950824	4,995628061	3,04001E-07	1,03185E-06
CYTIP	-1,25541878	4,777474374	3,08861E-07	1,04782E-06
PIP4K2B	-0,576952862	6,53909051	3,11649E-07	1,05675E-06
SRP9	0,833494743	8,574076343	3,11995E-07	1,0574E-06
PRKAB1	-0,646725483	5,850614393	3,20313E-07	1,08506E-06
ZCRB1	0,708362444	6,21885063	3,2219E-07	1,09087E-06
SPTLC2	-0,710536591	6,367538068	3,23497E-07	1,09475E-06
ARAF	-0,450114822	6,526974631	3,24446E-07	1,09742E-06
PRKCI	0,918782552	7,321768146	3,26061E-07	1,10234E-06
PPP3CA	-0,625676163	6,330951192	3,27477E-07	1,10657E-06
EEF1E1	0,809245403	4,424187329	3,31581E-07	1,11989E-06
CLU	-1,35421628	8,498000481	3,32049E-07	1,12091E-06
KCTD5	0,623468324	6,049072358	3,33765E-07	1,12615E-06
SEPT11	-0,698425486	7,2975515	3,36262E-07	1,13401E-06
DR1	0,673295318	6,747130022	3,3783E-07	1,13873E-06
P4HA1	1,098548001	7,64761411	3,38275E-07	1,13967E-06
SYDE1	-0,898537607	4,154768752	3,38937E-07	1,14134E-06
CAMK1	-0,749470236	4,161178324	3,40903E-07	1,14739E-06
SCAMP2	-0,49362394	7,332481174	3,41258E-07	1,14802E-06
NPM1	0,747750957	9,925651264	3,4244E-07	1,15143E-06
WDR45L	0,846821671	7,726546769	3,44953E-07	1,15931E-06
EIF4G1	0,976596115	10,0063284	3,48046E-07	1,16912E-06
ATP1B3	1,432456515	9,169567363	3,4964E-07	1,1739E-06
UCKL1	0,724479344	5,512176083	3,50857E-07	1,17741E-06
SLC44A1	0,947498249	8,043148809	3,51229E-07	1,17808E-06
RPL8	1,014722573	11,32838147	3,53365E-07	1,18466E-06
RPL18A	1,190061106	10,42734388	3,55651E-07	1,1914E-06
DYRK1A	-0,550811537	6,460500693	3,55724E-07	1,1914E-06
RUFY3	-0,717397225	5,217059952	3,68676E-07	1,23417E-06
SCMH1	-0,613083361	5,264383296	3,72474E-07	1,24627E-06
GPCPD1	-0,866342387	6,42103946	3,73634E-07	1,24921E-06
SRRD	0,667760176	4,599527038	3,73719E-07	1,24921E-06
UBXN7	1,117226565	5,43740621	3,7722E-07	1,2603E-06
RAB14	-0,52506689	7,602554295	3,7936E-07	1,26682E-06
SNX14	-0,689056547	6,017349993	3,85238E-07	1,28582E-06
ATL2	0,826440608	6,921017416	3,86773E-07	1,29032E-06
RBM5	-0,545018934	6,180942952	3,87957E-07	1,29363E-06

CHSY1	-0,761341253	6,593295645	3,90458E-07	1,30134E-06
PGM1	-0,795325831	6,817361975	3,93904E-07	1,31218E-06
PDCL3	0,64734027	5,435522258	3,95352E-07	1,31636E-06
PSMC1	0,612937426	7,737865364	3,99091E-07	1,32816E-06
KHSRP	0,715662027	7,736360604	4,0074E-07	1,333E-06
RHOD	1,788020756	5,408937321	4,0513E-07	1,34694E-06
TMEM159	1,111236441	6,501579801	4,06385E-07	1,35046E-06
MIDN	-0,733262888	7,019037106	4,06999E-07	1,35184E-06
RPL37	0,984944293	10,20213646	4,08769E-07	1,35706E-06
DHX37	0,7002853	5,272940051	4,0943E-07	1,35859E-06
RPL7L1	0,555391287	7,096952523	4,13662E-07	1,37138E-06
DGKD	-1,002171744	5,443704086	4,13687E-07	1,37138E-06
ZDHHC7	-0,6626491	6,438755179	4,18062E-07	1,38521E-06
MRPL44	0,639125391	5,454933879	4,21422E-07	1,39567E-06
DSTN	-0,651656371	8,99724961	4,28249E-07	1,41759E-06
TMEM161A	0,805905343	5,510403124	4,30404E-07	1,42403E-06
TRIM56	-0,807098224	4,321471841	4,31136E-07	1,42576E-06
PLEKHG3	1,222847415	6,061906836	4,32062E-07	1,42813E-06
PLIN2	-1,187412974	6,605165348	4,46166E-07	1,47403E-06
HCK	-1,151579313	6,167078503	4,50722E-07	1,48837E-06
RBBP7	0,760198784	7,511420248	4,53453E-07	1,49666E-06
RPL26L1	0,738581484	5,135417087	4,63675E-07	1,52966E-06
POT1	0,842118204	5,572027636	4,66131E-07	1,53701E-06
PARP3	-0,755437981	4,453232363	4,69152E-07	1,54623E-06
MICALL1	1,134328336	6,968629441	4,7253E-07	1,55661E-06
NUDT3	0,583890583	5,610975387	4,78186E-07	1,57448E-06
LAP3	-0,793492279	7,297586236	4,82717E-07	1,58863E-06
NDUFA8	0,779387158	5,884229016	4,84594E-07	1,59404E-06
SRGN	-1,380528993	7,91839854	5,00836E-07	1,64667E-06
ISY1	0,551702506	5,739173231	5,02712E-07	1,65204E-06
FOXO3	-0,712167843	6,836437483	5,10257E-07	1,67603E-06
THBD	-1,485693757	7,164808351	5,1717E-07	1,69792E-06
GTF2A2	0,842103517	6,101047434	5,20235E-07	1,70716E-06
CNPY2	0,631216944	6,515417562	5,2864E-07	1,73391E-06
NCOA1	-0,689905216	6,440051416	5,30327E-07	1,73861E-06
RPLP1	0,933083984	11,12221722	5,33759E-07	1,74902E-06
FLJ45445	1,46958002	5,562590395	5,41507E-07	1,77355E-06
GAPVD1	-0,559079543	6,25188977	5,47367E-07	1,79189E-06
DCN	-1,389038182	9,783197917	5,47959E-07	1,79296E-06
CBR1	2,160244832	8,227459645	5,49592E-07	1,79744E-06
TIMM9	0,780875492	5,107013496	5,55533E-07	1,816E-06
LEF1	1,556307053	4,860040114	5,62925E-07	1,83928E-06
POLR2J2	1,429574374	5,901285871	5,73941E-07	1,87438E-06
ARHGAP33	1,599598922	3,296036781	6,00652E-07	1,96022E-06
CISD2	0,765489978	5,922508436	6,008E-07	1,96022E-06
THOC5	0,667451193	5,881191396	6,04464E-07	1,97123E-06
ARMC10	0,674619453	6,562553765	6,14095E-07	2,00168E-06
WIPF1	-0,912768192	6,368658922	6,14502E-07	2,00205E-06
SLMO2	0,925577	7,330690663	6,19191E-07	2,01636E-06
C15orf48	2,519659541	5,796272157	6,20751E-07	2,02048E-06

TMEM167B	-0,507305726	6,161984967	6,28685E-07	2,04532E-06
RFXANK	0,714660075	5,926433993	6,35798E-07	2,06748E-06
BCCIP	0,647890016	6,19582039	6,457E-07	2,09868E-06
CTTNBP2NL	-0,610332589	5,966776711	6,49382E-07	2,10964E-06
CBWD2	0,55730042	4,907695151	6,49728E-07	2,10976E-06
POLR3D	0,621757252	4,921771196	6,52637E-07	2,1182E-06
SLC26A2	-1,073476119	5,698449782	6,61712E-07	2,14663E-06
ISOC2	0,866777538	5,943036406	6,65324E-07	2,15732E-06
HAPLN3	1,325832811	5,091885632	6,67628E-07	2,16376E-06
COL4A2	-1,020187574	9,177052377	6,69876E-07	2,17002E-06
FAM160B1	-0,53685519	5,22167731	6,72499E-07	2,17748E-06
TSEN34	0,642288179	6,17251758	6,81065E-07	2,20417E-06
RASAL3	-1,100941333	3,63572918	6,90162E-07	2,23255E-06
ANKHD1	-0,597168561	6,408707547	6,92235E-07	2,2382E-06
SPIRE1	-0,88213344	5,538613569	6,93195E-07	2,24024E-06
FH	0,695822417	6,649236932	6,98357E-07	2,25585E-06
ZC3HC1	0,567586337	4,68256114	7,0608E-07	2,27972E-06
FAM46A	-1,027145911	5,058642848	7,08758E-07	2,28728E-06
LPIN3	0,998476175	4,86026564	7,11412E-07	2,29476E-06
NAA38	0,643098817	4,903598903	7,21339E-07	2,32568E-06
RPL18	0,880719353	9,891360295	7,2279E-07	2,32926E-06
MAST4	-0,952240886	5,090095359	7,26425E-07	2,33986E-06
C15orf38	-0,737923758	4,472040406	7,3532E-07	2,3674E-06
EIF3M	0,735175122	7,654730548	7,40157E-07	2,38185E-06
NOC2L	0,731172113	7,126467209	7,41829E-07	2,3861E-06
TNIK	-1,305384288	4,333024375	7,45786E-07	2,3977E-06
PTCD2	0,590702832	3,142590114	7,47625E-07	2,40248E-06
ITPA	0,751398545	5,836608107	7,51369E-07	2,41337E-06
SLC25A5	1,062805814	9,583962745	7,51798E-07	2,41361E-06
TUFM	0,588938985	8,166221282	7,56592E-07	2,42722E-06
WRNIP1	0,659548313	6,461557624	7,56748E-07	2,42722E-06
DOCK5	-0,933549415	4,351432506	7,5912E-07	2,43368E-06
FLNC	-1,537566449	4,684242031	7,6759E-07	2,45968E-06
LACTB	-0,636698052	4,745369016	7,6978E-07	2,46554E-06
HMG20B	0,616815732	6,238174993	7,76211E-07	2,48497E-06
AGXT2L2	-0,56305106	4,551336164	7,86486E-07	2,51668E-06
BTBD2	0,795361233	7,158522876	7,88149E-07	2,52082E-06
RAD21	0,60657963	8,713090263	7,9673E-07	2,54707E-06
PHB	0,701289881	7,80128873	7,98765E-07	2,55238E-06
PPIA	0,827931164	10,84122046	8,03364E-07	2,56587E-06
NDUFS8	0,830725932	6,434865482	8,03848E-07	2,56622E-06
CPNE1	1,060555951	7,817468445	8,05582E-07	2,57055E-06
NEK9	-0,619664501	6,597318613	8,15241E-07	2,60015E-06
FBXO46	0,812812164	4,839278744	8,19855E-07	2,61365E-06
NT5C3L	0,91199519	5,982775887	8,23627E-07	2,62445E-06
KDM3A	0,869569153	7,01498354	8,44428E-07	2,68947E-06
AZIN1	0,687708152	8,378602585	8,49622E-07	2,70475E-06
ZFHX3	-0,824951775	5,754330142	8,5509E-07	2,72089E-06
TCF4	-0,919269194	7,005942352	8,56579E-07	2,72435E-06
PTMA	0,681858388	10,87583447	8,73577E-07	2,77712E-06

ZNF92	0,814863141	3,845972317	8,76599E-07	2,78543E-06
RP2	-0,660907952	5,253743889	9,01308E-07	2,86261E-06
PTTG1IP	-0,583738976	8,499223531	9,09966E-07	2,88876E-06
WDR82	-0,406406431	7,364540066	9,13322E-07	2,89806E-06
IRF1	-0,972384259	7,005957776	9,18317E-07	2,91256E-06
YWHAG	0,675066809	8,882177954	9,31049E-07	2,95157E-06
SP2	-0,473971533	5,241316558	9,33033E-07	2,95648E-06
DCTN5	0,572811583	6,978337694	9,36244E-07	2,96528E-06
SNORA8	0,95021139	4,651570344	9,68896E-07	3,06727E-06
BBS5	1,243425297	5,588085131	9,71477E-07	3,07402E-06
HNRNPAB	0,61447034	7,556927824	9,73506E-07	3,07901E-06
EHHADH	0,935104227	5,33022265	9,94455E-07	3,1438E-06
P4HB	0,746395338	10,7356964	9,98643E-07	3,15558E-06
STON2	1,535574721	5,336564625	1,00535E-06	3,17529E-06
ENY2	0,699612659	5,784885064	1,015E-06	3,20429E-06
NCOR2	-0,775541427	7,779217354	1,01609E-06	3,20625E-06
ARHGEF9	-0,851896847	4,439796988	1,03051E-06	3,25026E-06
LUZP1	-0,7126828	6,061928995	1,04634E-06	3,29866E-06
C8orf33	0,710800397	6,686012985	1,05442E-06	3,32258E-06
ACOT11	1,186627562	3,739452203	1,09142E-06	3,43759E-06
ANAPC1	0,6471852	6,013224427	1,12366E-06	3,5375E-06
ZNRD1	0,816476032	4,935848213	1,12578E-06	3,54253E-06
HBS1L	0,673081384	6,399994907	1,13244E-06	3,56187E-06
KIAA0430	-0,612220831	6,484989369	1,13669E-06	3,57357E-06
STAT6	-0,629694892	7,970828101	1,13805E-06	3,57622E-06
C1orf21	-0,819515066	6,17741784	1,14288E-06	3,5894E-06
SNF8	0,552643207	6,229338405	1,1433E-06	3,5894E-06
GTPBP4	0,720838867	6,511275683	1,1466E-06	3,5981E-06
RAD1	0,726328869	6,207181188	1,14836E-06	3,60196E-06
DHX57	0,545121696	5,522179331	1,15325E-06	3,61565E-06
B2M	-0,959278979	12,08617281	1,16295E-06	3,64436E-06
RPL36A	1,017906424	9,397709964	1,17139E-06	3,66912E-06
SSH1	-0,66844115	6,533453953	1,17304E-06	3,67261E-06
IMMT	0,562940956	7,298709327	1,18727E-06	3,71545E-06
ACOT7	0,960500518	6,082692889	1,21175E-06	3,79034E-06
RANBP2	-0,577263548	7,255405352	1,21569E-06	3,80091E-06
COX7A2L	0,867727379	7,562293435	1,2252E-06	3,82889E-06
BTBD10	0,687046275	5,963890339	1,23255E-06	3,85009E-06
MESDC1	-0,659286559	5,240575032	1,23629E-06	3,86002E-06
TTC27	0,584938256	5,092938005	1,2736E-06	3,97468E-06
B4GALT3	0,81292765	6,45159635	1,2749E-06	3,97691E-06
TGS1	0,623218461	5,770807126	1,28944E-06	4,02044E-06
ST6GALNAC4	-0,797905404	5,034049135	1,29024E-06	4,0211E-06
HAX1	0,832526002	7,176856851	1,29618E-06	4,03775E-06
CAST	-0,663931822	8,262566575	1,32037E-06	4,11124E-06
NUDT9	-0,568262828	5,131484346	1,32374E-06	4,11984E-06
C5orf24	-0,548505803	6,155683027	1,32572E-06	4,12411E-06
SNAPIN	0,780955718	5,958805771	1,33485E-06	4,15065E-06
WHSC2	0,785038118	5,475428146	1,35593E-06	4,21425E-06
ATXN2L	0,652048474	7,644074864	1,35783E-06	4,21825E-06

GADD45GIP1	0,948573144	5,90233733	1,36058E-06	4,22487E-06
CLSTN1	0,990135167	9,033064154	1,36213E-06	4,22774E-06
NUMBL	1,032868906	5,346504043	1,36337E-06	4,22968E-06
TBL2	0,65533355	6,389681755	1,3718E-06	4,2539E-06
INSIG1	-0,82698938	6,660215641	1,38261E-06	4,28546E-06
VDAC1	0,597815222	8,193069451	1,39383E-06	4,31827E-06
SSSCA1	0,736163415	4,782178257	1,41649E-06	4,38649E-06
UBE2Z	0,45993775	7,825050574	1,41983E-06	4,39484E-06
PSME3	0,559733551	7,74316438	1,4208E-06	4,39584E-06
MORF4L2	0,706863651	8,480928171	1,42243E-06	4,39888E-06
MRRF	0,774828234	5,159364638	1,42752E-06	4,41264E-06
DRAP1	0,761897966	6,611759698	1,43549E-06	4,43526E-06
KPTN	0,950511656	3,857847235	1,44967E-06	4,47704E-06
ST20	1,030718118	2,372871667	1,45339E-06	4,48649E-06
RPL35	1,19218285	9,657592405	1,45585E-06	4,49207E-06
EDC3	0,500853551	5,848045154	1,46957E-06	4,53235E-06
MITD1	0,652197735	4,252327953	1,47486E-06	4,54661E-06
DERA	0,974128121	6,513652732	1,47602E-06	4,54812E-06
PRKAB2	0,803494859	5,513861267	1,49705E-06	4,61084E-06
ABCC1	1,74192786	8,628978832	1,4996E-06	4,61661E-06
IFNAR2	-0,688275222	5,302777107	1,5233E-06	4,68743E-06
RPL36	0,976191587	8,992149217	1,52907E-06	4,70307E-06
ULK2	-0,737905659	5,091280187	1,53041E-06	4,70507E-06
NDUFV1	0,551706231	7,15922742	1,53786E-06	4,72586E-06
FNTA	0,767475308	7,153446985	1,54184E-06	4,73395E-06
PPHLN1	0,544745077	6,228964296	1,54188E-06	4,73395E-06
RNF168	1,320768909	5,166296585	1,54954E-06	4,75533E-06
EIF4A2	0,988320625	9,685244616	1,55313E-06	4,76419E-06
CAB39	-0,553324309	7,004006404	1,55608E-06	4,7711E-06
ATXN2	-0,483668171	5,547979583	1,56055E-06	4,78266E-06
ANKRD16	0,652179296	3,829553458	1,56509E-06	4,79426E-06
KEAP1	0,936888498	7,29892595	1,56574E-06	4,79426E-06
EXOSC1	0,640696825	5,012983926	1,57261E-06	4,8131E-06
ME1	2,071591609	7,224848082	1,58775E-06	4,85727E-06
NOC4L	0,683046202	4,894956044	1,62032E-06	4,95469E-06
CDKN1C	-1,109163014	3,850637707	1,64749E-06	5,03552E-06
C19orf24	0,771157805	5,44611116	1,65036E-06	5,04204E-06
HSPG2	-1,135253756	8,754957969	1,67828E-06	5,12503E-06
PIK3AP1	-1,014200187	5,52539646	1,72763E-06	5,27336E-06
HIST1H4H	2,047044237	3,242869385	1,73811E-06	5,30298E-06
CCDC159	-0,58775059	3,42576621	1,74272E-06	5,31467E-06
TIMM13	0,82172641	5,98121375	1,74366E-06	5,31518E-06
C8orf59	0,944777204	5,611478326	1,74914E-06	5,32948E-06
KPNB1	0,560792159	8,489728477	1,77521E-06	5,40651E-06
HMGN1	0,583519046	7,904922054	1,7848E-06	5,43329E-06
PARD3	0,876100844	6,869689626	1,78869E-06	5,4427E-06
CCDC134	0,767284409	2,615642515	1,81623E-06	5,52402E-06
SCRIB	0,97567524	6,960546192	1,83646E-06	5,58306E-06
MTF1	-0,629207696	5,12451735	1,84387E-06	5,60311E-06
HSF1	0,718713449	7,17036943	1,84702E-06	5,61016E-06

HSF4	1,455859096	2,993481857	1,86688E-06	5,66796E-06
POLR2J3	1,401934495	6,457885676	1,8702E-06	5,67551E-06
ULK1	0,795996703	6,450742939	1,88283E-06	5,7113E-06
PSMD10	1,021737063	6,792642149	1,88839E-06	5,72563E-06
SERBP1	0,505497872	8,932795625	1,89477E-06	5,74243E-06
HSPBP1	0,806185381	5,91844584	1,9106E-06	5,78781E-06
PLEKHA8	0,685989512	3,036815411	1,93338E-06	5,85423E-06
VRK2	0,824526295	5,849010925	1,93469E-06	5,85559E-06
HSP90AB1	0,643716074	11,04979611	1,94314E-06	5,87855E-06
TRMT12	0,654633393	4,609949563	1,94851E-06	5,8922E-06
TAF4B	1,425232071	4,496877776	1,95761E-06	5,91566E-06
MYO1D	-0,685431455	7,211931381	1,95801E-06	5,91566E-06
CREB3L1	-1,312440709	5,505340887	1,98048E-06	5,9809E-06
HPS3	1,029571003	6,537589956	2,00861E-06	6,06318E-06
ATP2A2	0,664500607	9,355993881	2,01127E-06	6,0685E-06
STYXL1	0,875166658	5,638046483	2,02692E-06	6,11302E-06
RSL1D1	0,559357963	7,999170772	2,04725E-06	6,17161E-06
C18orf21	0,802649324	4,360020883	2,04975E-06	6,17644E-06
ABLIM1	-0,90930853	7,804923807	2,08185E-06	6,27039E-06
TALDO1	1,241861588	8,727312629	2,09201E-06	6,29821E-06
IBTK	-0,56455252	6,051340085	2,11059E-06	6,35132E-06
C19orf53	0,904056389	6,830851445	2,11656E-06	6,36649E-06
HDHD2	-0,616310946	5,000249618	2,1238E-06	6,38546E-06
QPCTL	0,722463974	4,667210804	2,14824E-06	6,45609E-06
BOD1	0,805025894	6,153167554	2,15223E-06	6,46523E-06
CDKN2AIPNL	0,922804108	5,124325142	2,19449E-06	6,58927E-06
MRPL28	0,656841813	6,069534852	2,19878E-06	6,59924E-06
CEP97	0,908371945	3,921285482	2,20272E-06	6,60816E-06
VSIG10	-1,017723608	5,094194521	2,20678E-06	6,61742E-06
SLC25A24	-0,703665907	5,93582479	2,22128E-06	6,65799E-06
NAT9	0,702731632	5,108372396	2,23271E-06	6,6893E-06
AP1M1	-0,480703141	6,102037959	2,24492E-06	6,72292E-06
AKAP9	-0,79857838	6,210642733	2,26517E-06	6,7806E-06
LYRM4	0,872107777	5,378493148	2,28319E-06	6,83154E-06
TCEA1	0,723816994	7,971626767	2,30597E-06	6,89668E-06
TRIM37	0,577195307	5,647602687	2,3435E-06	7,00585E-06
C12orf29	0,838107861	5,081811622	2,34678E-06	7,0102E-06
HPS4	0,709681824	6,023271013	2,34701E-06	7,0102E-06
GPR56	1,132872216	8,077966212	2,3664E-06	7,06501E-06
SMARCD2	0,617369619	7,535251601	2,40814E-06	7,18647E-06
BHLHE40	-0,852313675	8,615753416	2,44265E-06	7,28366E-06
KRR1	0,544623805	6,020540724	2,44284E-06	7,28366E-06
EIF3I	0,601616937	8,307962088	2,4449E-06	7,28661E-06
OSTC	0,92393983	7,288534775	2,46349E-06	7,33882E-06
YIF1A	0,796354307	6,587871416	2,53594E-06	7,55134E-06
MAPKAPK5	0,466039206	5,45783853	2,54859E-06	7,58569E-06
GPR183	-1,156239338	4,66849519	2,55656E-06	7,60358E-06
HIST1H1C	1,890986334	6,067770129	2,55683E-06	7,60358E-06
AHNAK2	2,335355602	7,97460477	2,57381E-06	7,65075E-06
TIGD2	0,83443293	3,486853774	2,59539E-06	7,71153E-06

CMTM7	-0,869707068	5,047790622	2,60614E-06	7,7401E-06
TNFRSF21	1,27231834	7,855063436	2,61356E-06	7,75877E-06
CCL4	-1,179202899	3,886171208	2,62333E-06	7,78439E-06
NT5DC2	0,883889557	6,621438099	2,64788E-06	7,8538E-06
TBC1D20	-0,50149132	6,150230697	2,64977E-06	7,85599E-06
FLII	-0,604150795	7,609081676	2,66497E-06	7,89764E-06
CDK3	0,826660206	4,648066679	2,68814E-06	7,96283E-06
ZNF16	0,651947919	4,057000695	2,69831E-06	7,98947E-06
SRPK2	0,623741917	6,337022656	2,70118E-06	7,99451E-06
SLC3A2	0,999421598	8,617555807	2,72156E-06	8,0499E-06
CKAP5	0,845900999	7,525277776	2,72226E-06	8,0499E-06
HYI	-0,840759975	4,066659357	2,76759E-06	8,1804E-06
CALU	0,838694239	8,79779857	2,80612E-06	8,29069E-06
PRPF40A	0,440043927	7,36978978	2,80786E-06	8,29224E-06
BRMS1	0,622716609	6,164938783	2,82593E-06	8,34199E-06
FANCM	0,661418805	3,33298777	2,8502E-06	8,41E-06
GABPB1	0,552047439	5,429142953	2,86413E-06	8,44606E-06
CSGALNACT2	-0,688406896	5,522136226	2,8649E-06	8,44606E-06
DHX35	0,916928184	4,922563675	2,88316E-06	8,49624E-06
FOXK2	0,704312012	6,930181025	2,89273E-06	8,52076E-06
IPPK	0,881124989	5,006439724	2,90337E-06	8,5484E-06
PLA2G12A	-0,497971443	5,303497868	2,91474E-06	8,57816E-06
UBE2L3	0,621879591	7,701069021	2,91682E-06	8,58059E-06
ABHD3	1,119291198	5,757111485	2,93145E-06	8,61989E-06
TIMM17A	0,558015772	6,230453225	2,96288E-06	8,70856E-06
APTX	0,884345125	5,743311623	3,00733E-06	8,83541E-06
SEC61G	1,288094178	6,823701328	3,03493E-06	8,91265E-06
KARS	0,55250715	7,819441873	3,04316E-06	8,93298E-06
THOC1	0,658231591	4,744231099	3,0761E-06	9,02578E-06
SLC25A1	0,888262112	7,046086945	3,10764E-06	9,11441E-06
COPS3	0,68399798	6,648407768	3,12258E-06	9,15429E-06
NUDT19	0,767776769	5,484179259	3,13391E-06	9,18355E-06
PDCD2	0,694711921	5,96115874	3,14674E-06	9,2172E-06
DMXL2	-0,721200924	5,174518538	3,16032E-06	9,25299E-06
TOMM20	0,69239803	8,820242234	3,17621E-06	9,29552E-06
GPR180	0,73460597	4,69852495	3,19367E-06	9,34263E-06
SLC7A5P2	0,637643581	1,613068386	3,19933E-06	9,35517E-06
PMS1	0,577157944	4,26164851	3,20312E-06	9,3586E-06
AMOTL1	-0,973528737	6,843152575	3,20325E-06	9,3586E-06
C1orf54	-0,920266133	3,127232454	3,21473E-06	9,38811E-06
RPA1	0,689455445	7,562334545	3,26167E-06	9,52113E-06
SNHG6	1,02471813	6,336743942	3,2647E-06	9,52588E-06
ATP6AP1	-0,453606072	7,949044179	3,27782E-06	9,56008E-06
USP31	0,946402018	5,979063075	3,28232E-06	9,56911E-06
ALG8	0,685103787	5,642990877	3,29156E-06	9,59196E-06
RPS5	1,170720659	10,32923154	3,30014E-06	9,61284E-06
RALGAPA1	-0,62859416	5,17841017	3,35503E-06	9,76855E-06
PQLC1	-0,620690745	5,804502377	3,36313E-06	9,78796E-06
ERBB2IP	-0,458337699	6,624398274	3,37641E-06	9,82243E-06
TSR1	0,59074893	6,457750306	3,38808E-06	9,85217E-06

FJX1	1,339111026	4,024732726	3,39603E-06	9,87108E-06
OST4	0,816802009	7,737416897	3,40432E-06	9,89094E-06
TRIM23	-0,578498168	3,853776825	3,46501E-06	1,0063E-05
MARCH6	0,721045182	7,467743302	3,51217E-06	1,01956E-05
GPX7	1,448817308	4,892647336	3,57169E-06	1,0364E-05
POFUT1	0,628522853	7,152423513	3,6071E-06	1,04623E-05
ZFR	0,473457448	7,393545832	3,6295E-06	1,05228E-05
PRKAG1	0,553138549	6,641604779	3,63462E-06	1,05331E-05
PVR	-0,726648886	6,108039346	3,64568E-06	1,05607E-05
SUB1	0,937387223	8,172180287	3,67846E-06	1,06511E-05
ZNF544	0,770494589	5,408782315	3,70283E-06	1,07171E-05
CDC34	0,694532996	5,83967126	3,70908E-06	1,07307E-05
ATF5	1,585546787	6,975086617	3,72231E-06	1,07644E-05
FHL2	1,556976556	6,27504974	3,82592E-06	1,10593E-05
CBL	-0,654160857	5,829623085	3,83261E-06	1,1074E-05
SKA2	0,679767245	5,97247104	3,84739E-06	1,1112E-05
IL4I1	1,28381831	4,60386718	3,87011E-06	1,11728E-05
CLPP	0,623939848	5,850680751	3,9757E-06	1,14715E-05
CAPN1	0,712878214	8,946003235	3,97692E-06	1,14715E-05
TSTA3	0,939631201	6,882707576	3,98814E-06	1,1499E-05
MRPS15	0,919244496	6,68881708	4,05932E-06	1,16993E-05
ZDHHC17	-0,436010269	4,8850674	4,06243E-06	1,17033E-05
NAP1L4	0,535518273	7,509895947	4,10605E-06	1,1824E-05
NCBP1	0,587902525	6,126173529	4,12915E-06	1,18855E-05
AAMP	0,497244254	7,361621278	4,15636E-06	1,19587E-05
ZNF212	0,647489472	4,622286078	4,16915E-06	1,19905E-05
RUNX2	1,320689049	4,653353313	4,17474E-06	1,20015E-05
C2orf68	0,636366816	6,187880764	4,19947E-06	1,20627E-05
SEH1L	0,638373329	6,394354872	4,19958E-06	1,20627E-05
IARS2	0,577998006	7,709205739	4,24598E-06	1,21909E-05
DDOST	0,536438302	8,792581049	4,26776E-06	1,22475E-05
EIF2B4	0,476793096	5,733029228	4,2693E-06	1,22475E-05
PDHX	0,626249031	5,66692965	4,27193E-06	1,22499E-05
GORASP2	0,462783124	7,646369296	4,28014E-06	1,22683E-05
SETD7	-0,68926239	6,823691023	4,28285E-06	1,22709E-05
ABT1	0,744859385	5,939167885	4,2936E-06	1,22965E-05
MEST	1,370928668	7,498747586	4,31667E-06	1,23574E-05
CCDC117	0,66010322	6,00713926	4,33539E-06	1,24058E-05
FAM127B	0,62656415	6,08723292	4,38713E-06	1,25486E-05
SLC35B1	0,521835733	6,374189482	4,41069E-06	1,26107E-05
ZNF408	0,695830802	4,281361865	4,41854E-06	1,26278E-05
DTNB	0,818175398	5,258844583	4,45528E-06	1,27275E-05
LIMK1	0,738604597	6,734545163	4,4843E-06	1,2805E-05
EIF3D	0,612573105	8,553428904	4,52015E-06	1,2902E-05
SLC2A3	-1,355963111	7,321478771	4,55645E-06	1,30002E-05
OSGIN2	-0,732169935	5,519511693	4,61908E-06	1,31734E-05
NDFIP1	-0,571312777	7,307029328	4,62982E-06	1,31985E-05
RBM8A	0,484153278	7,379679086	4,64818E-06	1,32453E-05
C1orf122	0,807146487	5,292456742	4,66496E-06	1,32875E-05
NPLOC4	0,542129769	7,825888264	4,71785E-06	1,34326E-05

MRPL27	0,695067661	5,775591231	4,79417E-06	1,36442E-05
MED6	0,609294467	5,080412167	4,84019E-06	1,37694E-05
FHOD3	1,97333289	4,598112854	4,87069E-06	1,38466E-05
PPP2R5A	-0,718360281	6,968714348	4,87138E-06	1,38466E-05
PRPSAP2	0,864173334	5,750689487	4,87629E-06	1,38548E-05
HIST4H4	0,890616795	0,980528355	4,92377E-06	1,39838E-05
PAPOLG	0,79387462	5,317371657	4,95048E-06	1,40539E-05
ACAP2	0,924465819	7,494981408	4,96338E-06	1,40846E-05
UTP3	-0,573539979	5,291061953	4,99461E-06	1,41673E-05
SIPA1	-0,704959043	5,699802351	5,03922E-06	1,42879E-05
GYS1	0,773602461	6,837476045	5,04768E-06	1,4306E-05
NDUFB11	0,670406833	6,793101156	5,18439E-06	1,46873E-05
CTDSPL2	0,683652749	6,07589011	5,2017E-06	1,47302E-05
MRE11A	0,606029252	5,105209811	5,20662E-06	1,4738E-05
DIP2B	0,888705332	7,177620808	5,21938E-06	1,4768E-05
RBPJ	-0,536136692	5,402133463	5,23524E-06	1,48068E-05
TMEM56	-0,947750698	3,939638245	5,23935E-06	1,48122E-05
ACO1	-0,773001263	6,145022559	5,28117E-06	1,49243E-05
INF2	-0,697548309	6,744939445	5,30882E-06	1,49962E-05
MAT2A	-0,562962506	8,029447301	5,35155E-06	1,51106E-05
TMEFF1	1,053553066	3,500232976	5,50957E-06	1,55504E-05
QRICH1	-0,346085604	6,204572101	5,53307E-06	1,56097E-05
MTR	-0,587541218	5,93979586	5,53516E-06	1,56097E-05
FASN	-0,916419179	8,576905254	5,54932E-06	1,56432E-05
LEPRE1	0,76923243	6,137491951	5,55335E-06	1,5648E-05
DNM2	-0,487063105	7,612970095	5,63516E-06	1,5872E-05
ZNF197	-0,455025182	4,052265489	5,66014E-06	1,59358E-05
EIF2C2	0,931058658	4,965472494	5,69247E-06	1,60202E-05
TFCP2	0,463255411	5,602526627	5,71211E-06	1,60688E-05
CD276	0,695467883	7,595524421	5,71778E-06	1,60782E-05
TMEM87A	-0,494423085	7,263755981	5,7975E-06	1,62956E-05
ISCA2	0,800155961	4,782747501	5,82925E-06	1,63781E-05
GPS1	0,659755564	7,191058461	5,86778E-06	1,64796E-05
NFE2L3	1,416406445	6,122475111	5,87969E-06	1,65062E-05
NSMCE2	0,905196876	5,125277355	5,91006E-06	1,65847E-05
LSM14B	0,679606626	6,257755387	5,91454E-06	1,65904E-05
C14orf166	0,580388139	7,281959537	5,93144E-06	1,6631E-05
RRP7B	0,666761133	4,689532787	5,97637E-06	1,675E-05
NCK1	0,9187856	6,409399201	5,9837E-06	1,67637E-05
TOM1	-0,575058823	6,230263025	6,05121E-06	1,69459E-05
DUSP12	0,640500776	4,488166146	6,10313E-06	1,70843E-05
C15orf61	0,83524144	3,287493379	6,17409E-06	1,72758E-05
WHSC1	0,979035148	6,958567385	6,19507E-06	1,73274E-05
PIGC	0,681950239	5,8158119	6,26569E-06	1,75177E-05
FBXW9	0,871765977	3,932232656	6,27034E-06	1,75236E-05
LSM1	1,195354078	5,892616386	6,29732E-06	1,75917E-05
RPL38	0,88506966	8,898275885	6,30768E-06	1,76129E-05
TXNL1	0,726280668	6,734942659	6,31006E-06	1,76129E-05
RSBN1	-0,487750872	5,02402125	6,32837E-06	1,76568E-05
FAM122A	-0,570781466	4,805136867	6,40958E-06	1,78761E-05

TERF2IP	-0,487045661	6,298400226	6,47634E-06	1,80549E-05
NACC1	0,698212383	6,916067444	6,49117E-06	1,80888E-05
KLF5	1,385997151	8,412023456	6,50278E-06	1,81138E-05
VASP	-0,589139262	7,173722124	6,58904E-06	1,83465E-05
CSNK1G2	0,65851748	6,450580805	6,62083E-06	1,84275E-05
PAPD5	-0,483866728	4,587592049	6,69963E-06	1,86392E-05
REPS1	0,715391854	6,320399989	6,71756E-06	1,86783E-05
GTF3C5	0,881172938	6,61857791	6,71914E-06	1,86783E-05
MUL1	-0,488865719	5,574961228	6,76126E-06	1,87877E-05
PGD	1,548081485	9,36213884	6,77069E-06	1,88063E-05
ZBTB41	0,725131667	5,636478668	6,8626E-06	1,90538E-05
GMPR	-1,213087784	3,385726285	6,99926E-06	1,94253E-05
C9orf114	0,545815253	5,166951868	7,02729E-06	1,94952E-05
SSB	0,56996575	7,235376834	7,08379E-06	1,96439E-05
PDE8A	-0,545612438	5,065551851	7,11016E-06	1,9709E-05
RPN1	0,633268264	9,523331047	7,17977E-06	1,98939E-05
PCGF5	-0,637553745	6,356401455	7,18274E-06	1,9894E-05
PPP6C	-0,416111173	6,692084041	7,19987E-06	1,99334E-05
TMEM39A	0,7289559	6,183293287	7,22919E-06	2,00064E-05
CYTH4	-0,966680112	4,817561272	7,24183E-06	2,00333E-05
UGDH	1,366290771	7,582149092	7,28213E-06	2,01366E-05
ELK3	-0,772565404	5,290639135	7,34521E-06	2,03028E-05
MRPS28	0,970519605	5,159116907	7,35029E-06	2,03086E-05
NECAP2	-0,575765877	6,813284059	7,37365E-06	2,03649E-05
ZBED4	0,664976074	5,777560203	7,39671E-06	2,04203E-05
UBFD1	0,556228736	6,468650658	7,43301E-06	2,05122E-05
COPE	0,683209531	7,621592206	7,52064E-06	2,07457E-05
SLK	-0,666107373	6,750173085	7,54859E-06	2,08143E-05
ELMO3	1,023682248	5,453149106	7,64172E-06	2,10626E-05
KPNA1	0,629536373	7,300865637	7,67858E-06	2,11556E-05
ZNF436	0,785889851	5,521653327	7,7312E-06	2,1292E-05
JAG2	1,19893711	6,273767514	7,73591E-06	2,12964E-05
PMS2L2	0,662371606	3,778926171	7,75709E-06	2,13461E-05
CNOT6L	-0,512121661	6,165782429	7,77624E-06	2,13902E-05
SNAP23	-0,493214962	6,607418261	7,78417E-06	2,14033E-05
NUP93	0,825557916	6,636671554	7,86096E-06	2,16057E-05
COX6C	0,735502859	7,141472865	7,89362E-06	2,16868E-05
LOC550112	0,679834975	4,213352369	7,90598E-06	2,1712E-05
TMEM147	0,716227057	6,85449589	8,04982E-06	2,20981E-05
CDK7	0,639003257	4,97096393	8,05441E-06	2,21018E-05
TMEM170A	-0,46084968	5,089170052	8,05873E-06	2,21048E-05
EPM2AIP1	-0,551751729	5,381671331	8,07197E-06	2,21322E-05
DCAF4	0,638712491	4,375431329	8,08577E-06	2,21611E-05
CRCP	0,494869789	6,306645771	8,09481E-06	2,2177E-05
LGALS3	-0,88613562	8,437625868	8,12852E-06	2,22604E-05
FAM117B	1,104505886	5,777052812	8,1493E-06	2,23083E-05
GPD2	0,680869745	6,970856873	8,2238E-06	2,25032E-05
FEZ2	-0,478240605	5,854959551	8,27488E-06	2,26339E-05
NOTCH2	-0,769840521	7,564962078	8,31726E-06	2,27407E-05
PRKX	1,290910952	6,650901149	8,33829E-06	2,27891E-05

SNX33	-0,653337988	5,734278254	8,34992E-06	2,28117E-05
ZC3H3	0,646779962	5,409162308	8,35697E-06	2,28218E-05
METTL9	0,652492125	7,311349965	8,39152E-06	2,2907E-05
TAF6L	0,558365619	4,318368367	8,42325E-06	2,29845E-05
E2F3	1,058198388	5,514752982	8,45967E-06	2,30746E-05
ZSWIM6	-0,57441232	4,901644264	8,47492E-06	2,3107E-05
CNOT3	0,635009473	6,318980907	8,47872E-06	2,31081E-05
SMCR8	-0,634047856	5,677122682	8,55958E-06	2,33142E-05
BAG4	1,280101851	5,045692534	8,56118E-06	2,33142E-05
CORO7	-0,542158542	5,612306924	8,59801E-06	2,34051E-05
NDUFA11	0,894488597	6,725281789	8,70195E-06	2,36786E-05
ITGA4	-0,94484246	4,983921832	8,96657E-06	2,43889E-05
SMAP2	-0,797313024	6,436368455	8,98855E-06	2,4439E-05
TMBIM6	-0,434905527	10,35062081	9,05901E-06	2,46207E-05
RFWD2	0,576999012	6,570155187	9,08684E-06	2,46865E-05
PIP5K1C	-0,589698568	6,022301195	9,10667E-06	2,47305E-05
BTN3A1	-0,801754276	5,995702676	9,17031E-06	2,48934E-05
MMP14	1,122106999	9,243187341	9,24471E-06	2,50854E-05
FAM49B	0,694181418	7,213636679	9,3503E-06	2,53619E-05
DCLRE1A	0,666149627	4,750741768	9,39122E-06	2,54627E-05
APRT	0,754457815	7,024822674	9,73217E-06	2,63766E-05
PARP14	-0,728817242	7,36984943	9,76323E-06	2,64503E-05
CCDC50	-0,695327499	6,796425294	9,78205E-06	2,64908E-05
RBPMS2	-1,097770689	1,816614891	9,80053E-06	2,65303E-05
NUP50	0,596460952	6,894776486	9,80538E-06	2,65329E-05
C11orf31	0,832430447	6,755967055	9,95426E-06	2,69251E-05
IL4R	-0,930363217	6,68768861	1,00943E-05	2,72929E-05
PTK2B	-0,838879838	6,267478564	1,01144E-05	2,73365E-05
TRAPPC10	-0,523888573	6,017556618	1,02907E-05	2,78021E-05
GSR	1,448885745	7,510946403	1,03528E-05	2,79588E-05
PSMB1	0,779584357	8,075989578	1,03851E-05	2,80348E-05
PLA2G16	-1,363356741	5,101110144	1,04014E-05	2,80677E-05
REXO4	0,563179849	5,225936904	1,058E-05	2,85385E-05
EEF1G	0,630373508	11,27922409	1,06169E-05	2,86265E-05
MAPK13	0,750311058	6,977280028	1,07924E-05	2,90883E-05
FRMD8	0,707724548	6,509488937	1,0805E-05	2,91108E-05
NT5C	0,906433006	5,14375721	1,08237E-05	2,91496E-05
TET3	0,941490293	6,302618713	1,08293E-05	2,91531E-05
LOC727896	0,616471248	1,290550725	1,11053E-05	2,98844E-05
SMNDC1	0,540386375	5,86704418	1,12179E-05	3,01754E-05
NUP88	0,617415483	5,949160684	1,12952E-05	3,03714E-05
ABHD13	-0,554802869	4,996483889	1,13705E-05	3,05618E-05
HTRA2	0,498220906	5,467783519	1,14095E-05	3,06547E-05
TOMM6	0,653880746	6,851677853	1,14475E-05	3,07446E-05
RABIF	0,54998152	4,865957436	1,14611E-05	3,0769E-05
ITGB3	-1,179157579	3,202216089	1,15483E-05	3,09909E-05
PSMA1	0,768778656	7,739816854	1,1635E-05	3,12113E-05
SDHC	0,760999818	7,741790539	1,16788E-05	3,13165E-05
DNMT1	0,833213639	7,129461156	1,17611E-05	3,15247E-05
STK39	0,781570704	5,820220899	1,17712E-05	3,15395E-05

HDAC3	0,423860264	6,402658435	1,19733E-05	3,20683E-05
C12orf10	0,549559548	5,702438891	1,19956E-05	3,21155E-05
C20orf111	0,823887611	6,280081819	1,20489E-05	3,22454E-05
C7orf43	0,591834359	4,589986073	1,22386E-05	3,27404E-05
RABGAP1L	-0,515390219	4,912631511	1,23852E-05	3,31195E-05
CHCHD10	1,049608674	5,169643586	1,25013E-05	3,34168E-05
FAM98A	0,474300252	5,962062013	1,26443E-05	3,37857E-05
FAM115A	-0,737167674	5,490818314	1,28084E-05	3,42108E-05
MICAL3	0,87349992	6,213871335	1,29294E-05	3,45205E-05
EIF2S3	0,69199263	8,552168568	1,31518E-05	3,51006E-05
PHKG2	0,581735277	5,301603594	1,31656E-05	3,51238E-05
DDX18	0,472607283	7,335942853	1,33604E-05	3,56294E-05
CLN3	-0,507565973	5,830143462	1,33847E-05	3,56802E-05
HAUS1	1,052033927	5,150681089	1,3398E-05	3,57019E-05
COPS8	0,522640385	6,255879144	1,34945E-05	3,59448E-05
RNF217	1,537198275	4,413339262	1,35451E-05	3,60655E-05
ZBTB33	0,650209638	6,058762142	1,36253E-05	3,6265E-05
TANC2	-0,815103017	6,232032823	1,36377E-05	3,62838E-05
MTRR	-0,600814254	5,887045569	1,36622E-05	3,63349E-05
LRWD1	0,675736582	4,848767176	1,37343E-05	3,65123E-05
VPS37B	-0,690690458	6,271289332	1,38386E-05	3,67753E-05
SIKE1	0,544601655	5,787955729	1,38977E-05	3,6918E-05
SV2A	1,490340505	4,488854102	1,39996E-05	3,71741E-05
RSAD2	-1,305625297	5,197554025	1,40296E-05	3,72393E-05
USE1	0,63022391	4,899323087	1,40466E-05	3,727E-05
TXNDC12	0,501158723	7,13448228	1,43274E-05	3,80002E-05
PMM1	-0,591886592	5,515641962	1,43884E-05	3,81472E-05
MDFIC	-0,72328896	5,946430801	1,45364E-05	3,85246E-05
CASC4	-0,511055673	7,127579365	1,48115E-05	3,92385E-05
C11orf10	0,597530262	6,734690812	1,48221E-05	3,92511E-05
PPP1CA	0,577593073	8,248432336	1,48707E-05	3,93646E-05
NFKBID	-0,82618817	1,889789841	1,49221E-05	3,94855E-05
SMARCA5	-0,545501471	7,211947391	1,49938E-05	3,96597E-05
IL17RA	-0,602821824	4,86874519	1,5014E-05	3,96977E-05
OCEL1	-0,71649966	3,714757554	1,50768E-05	3,98483E-05
WDR60	-0,576194006	4,309192271	1,52094E-05	4,01833E-05
TUBA4A	1,151556155	7,242236566	1,53203E-05	4,04605E-05
VPS33A	0,471467705	5,779459468	1,5369E-05	4,05736E-05
RPS21	1,092149807	9,135096473	1,54039E-05	4,06411E-05
HNRPDL	-0,386532714	7,721900091	1,54066E-05	4,06411E-05
KLHL7	0,630668356	5,750497129	1,54627E-05	4,07735E-05
SMEK2	0,511977153	7,364262955	1,55629E-05	4,10219E-05
DPH5	0,702976922	4,903018895	1,55996E-05	4,11026E-05
RDBP	0,617363945	6,857498629	1,56217E-05	4,1145E-05
ZNF33B	-0,588905844	4,971190112	1,57087E-05	4,13582E-05
SPATA2L	-0,694201047	3,805213852	1,59442E-05	4,1962E-05
TBCC	0,614599562	5,102354153	1,60508E-05	4,2211E-05
RRS1	0,748736561	5,104987757	1,60512E-05	4,2211E-05
NDUFS3	0,629751166	6,620704112	1,61364E-05	4,24188E-05
SF3B3	0,535984157	8,22437739	1,61645E-05	4,24762E-05

NDUFA6	0,690789021	6,476897916	1,61948E-05	4,25393E-05
FAM76B	0,60400785	4,426014565	1,62045E-05	4,25485E-05
USP10	0,528673163	6,908333991	1,63106E-05	4,28105E-05
SIVA1	0,962264241	6,67840767	1,63527E-05	4,29047E-05
HIST1H2BK	1,267658457	6,834404244	1,64779E-05	4,32165E-05
SPRYD3	-0,46056513	6,35403948	1,67772E-05	4,39845E-05
SNHG12	1,10940255	3,985661583	1,69787E-05	4,44897E-05
ARL4C	0,952580595	7,357191058	1,69829E-05	4,44897E-05
NAP1L5	-1,076982692	2,662414318	1,71228E-05	4,48388E-05
TRIM44	-0,625198542	6,288037431	1,7579E-05	4,60159E-05
FAM104A	0,508228805	5,639936627	1,79779E-05	4,70419E-05
UBD	2,271677734	6,008750767	1,81319E-05	4,74268E-05
MPHOSPH6	0,778266574	5,164609203	1,84143E-05	4,81469E-05
DNM1	1,19102024	4,745990685	1,85048E-05	4,8365E-05
LTBR	0,791540649	7,899387123	1,85926E-05	4,85758E-05
KDM3B	-0,507002219	6,783102757	1,86556E-05	4,87219E-05
AEBP2	0,697972852	6,105932808	1,87162E-05	4,88613E-05
CISD1	0,792242639	5,156992099	1,8733E-05	4,88864E-05
EIF2B1	0,407374033	6,234344257	1,87533E-05	4,89208E-05
NAA15	0,521356511	6,417526561	1,88121E-05	4,90371E-05
MRPL40	0,751096413	5,75604069	1,88123E-05	4,90371E-05
RPS18	1,222750469	11,71501329	1,8878E-05	4,91897E-05
FN3KRP	0,614897433	6,072139627	1,89108E-05	4,92561E-05
EPRS	0,577869786	7,911228383	1,8922E-05	4,92665E-05
KLHDC7B	2,865713641	4,658808676	1,90564E-05	4,95976E-05
MRPS14	0,621468334	4,890560524	1,91398E-05	4,97957E-05
SMARCD1	0,481800832	6,792985913	1,92255E-05	4,99994E-05
ACTA2	-1,038602775	8,262864657	1,92506E-05	5,00456E-05
GFM2	0,588433707	5,498198409	1,93771E-05	5,03554E-05
CRYZL1	-0,448711069	4,408563483	1,95023E-05	5,06613E-05
FTSJ2	0,485541431	5,657968555	1,95545E-05	5,07776E-05
ULK3	0,686042069	5,601597712	1,96097E-05	5,09016E-05
RBMX	0,429675507	8,074839484	1,9661E-05	5,10154E-05
POP5	0,676664712	4,91911193	1,98651E-05	5,15254E-05
ATP5G2	0,586663328	8,478317164	2,0031E-05	5,19359E-05
EARS2	0,531989902	5,361836293	2,00695E-05	5,2016E-05
NSFL1C	0,48354825	7,054453643	2,01814E-05	5,22861E-05
UBQLN4	0,525183593	6,862553571	2,02352E-05	5,24008E-05
PTPN13	-0,962806914	6,710278981	2,02482E-05	5,24008E-05
DCPS	0,560500045	4,773677184	2,02487E-05	5,24008E-05
MSH5	0,815889345	4,352076666	2,02942E-05	5,24985E-05
GALNT7	0,903925572	6,226375877	2,03146E-05	5,25315E-05
MYO9B	-0,59644954	6,890572752	2,04115E-05	5,2762E-05
CTSL1	-0,88173103	7,663086453	2,06842E-05	5,34467E-05
ABHD2	-0,9255138	8,36311412	2,09271E-05	5,40538E-05
PSMC3	0,558078875	7,552161798	2,09817E-05	5,41742E-05
ILF3	0,547580699	8,496940576	2,12825E-05	5,493E-05
RPL39	0,988517372	10,30171109	2,12907E-05	5,49306E-05
KPNA5	-0,678542304	2,309298276	2,13484E-05	5,50586E-05
STK24	0,588323132	7,975753493	2,13914E-05	5,51486E-05

GMIP	-0,623061783	5,024675736	2,1505E-05	5,54204E-05
ZDHHC18	0,583403547	6,223469358	2,16914E-05	5,58796E-05
DNAJC1	-0,610042995	5,371909637	2,17564E-05	5,60259E-05
BBS9	-0,523476156	4,135423523	2,19479E-05	5,64977E-05
MTPAP	0,4582645	5,270436095	2,19925E-05	5,65914E-05
COX7B	0,961639587	7,231743342	2,20531E-05	5,67258E-05
YWHAH	-0,444317286	7,824455607	2,21563E-05	5,69697E-05
GNB5	0,616530012	5,391179401	2,22769E-05	5,72583E-05
PRPF39	0,597294188	4,85169026	2,23924E-05	5,75333E-05
TRIAP1	0,604034405	5,472168781	2,2489E-05	5,77598E-05
FBXO33	-0,591506486	4,539987994	2,25738E-05	5,79559E-05
UBAC2	0,512940286	6,770383605	2,2636E-05	5,80937E-05
DUSP14	0,985521483	5,708672391	2,26739E-05	5,81691E-05
KIF21A	1,128889906	5,873894316	2,30215E-05	5,90387E-05
GPR108	-0,448773464	6,276300714	2,32379E-05	5,95711E-05
BCL7B	-0,453193677	6,240331028	2,32922E-05	5,9688E-05
PSMB7	0,521157129	7,32278067	2,34791E-05	6,01442E-05
STX6	0,552512043	6,852920205	2,42074E-05	6,19714E-05
MYH9	-0,787765152	10,9062691	2,42106E-05	6,19714E-05
DDIT4	1,393988597	8,788118688	2,43513E-05	6,23083E-05
VPS36	-0,497052253	5,984778504	2,4503E-05	6,26728E-05
CIAPIN1	0,613601948	6,413003152	2,45807E-05	6,2848E-05
ATP5H	0,691192232	7,50993163	2,46232E-05	6,29332E-05
SNRNP48	0,608053764	4,542929182	2,47981E-05	6,33563E-05
NUMA1	-0,652627771	8,255893147	2,48287E-05	6,34109E-05
GABPA	-0,495214933	5,529906741	2,48985E-05	6,35653E-05
SGPP1	-0,829396716	4,750938251	2,51107E-05	6,4083E-05
CRBN	-0,690987883	5,192480143	2,51868E-05	6,42532E-05
MREG	0,820964015	5,836198566	2,52794E-05	6,44653E-05
NFXL1	0,635104647	4,203506261	2,53238E-05	6,45545E-05
PPP1R2	0,65502436	7,166244938	2,55716E-05	6,51616E-05
NCKAP5L	-0,616504824	5,067470258	2,57906E-05	6,56951E-05
ADAP2	-0,788922232	4,222422482	2,58892E-05	6,59218E-05
PTPN23	-0,482081544	6,187510263	2,59805E-05	6,61296E-05
VARS	0,640675562	7,195391525	2,60928E-05	6,63907E-05
UNC93B1	-0,695229712	6,016078858	2,62103E-05	6,66647E-05
GLRX2	0,712352785	3,215772901	2,64505E-05	6,72505E-05
MANBAL	0,589583516	6,640390328	2,65639E-05	6,75138E-05
SLC38A7	0,701120788	4,532665009	2,67866E-05	6,80544E-05
CMTM8	-0,782526735	3,656353646	2,69266E-05	6,83846E-05
CSNK2B	0,515669752	7,576188934	2,71294E-05	6,8874E-05
CHPF	0,961421039	7,521495011	2,758E-05	6,99918E-05
TECR	0,768513816	7,772328114	2,77607E-05	7,04241E-05
COL4A1	-0,833799308	9,1066381	2,78232E-05	7,05564E-05
EI24	0,525127409	7,750564281	2,81667E-05	7,14009E-05
TUSC3	1,11507012	6,527820309	2,82395E-05	7,1559E-05
PPP3R1	0,484366131	7,353463674	2,83305E-05	7,17628E-05
RPS15	0,747886354	8,999599366	2,88567E-05	7,30687E-05
ZDHHC3	-0,542357266	7,141634602	2,90794E-05	7,36052E-05
MORC4	0,869298708	6,998628384	2,9311E-05	7,41638E-05

RNF26	0,561126935	6,487677941	2,99977E-05	7,58733E-05
AURKAIP1	0,722415224	6,294341713	3,01109E-05	7,61312E-05
VPS53	-0,444927512	6,072461959	3,02076E-05	7,63476E-05
ARMCX3	-0,598990487	6,063551797	3,04836E-05	7,70166E-05
DAB2IP	-0,847139576	6,398852232	3,05027E-05	7,70362E-05
SLC35F5	0,829494131	6,446403609	3,05315E-05	7,70805E-05
NUTF2	0,65372373	7,183505996	3,07205E-05	7,7529E-05
CDK5RAP3	0,562567854	6,718357284	3,0888E-05	7,79229E-05
VEGFC	-1,018259654	3,379429132	3,11096E-05	7,84529E-05
CCNC	0,722188372	6,851810403	3,11599E-05	7,85505E-05
HOOK2	0,777504268	5,467781042	3,11877E-05	7,85915E-05
TCEAL4	-0,554788177	6,278786453	3,12034E-05	7,86021E-05
CNIH	0,860230096	7,201923589	3,13209E-05	7,88691E-05
FADD	1,233896824	5,925031298	3,13461E-05	7,89032E-05
KCNK5	-1,19921266	3,884658083	3,15573E-05	7,94057E-05
TNK2	0,929003744	6,340029838	3,16058E-05	7,94983E-05
RHOT2	0,653563276	6,22330538	3,18848E-05	8,01704E-05
DBN1	0,755431461	6,877862019	3,19029E-05	8,01866E-05
HSPB11	0,695388845	4,761264854	3,22556E-05	8,10432E-05
MANF	0,630456024	6,371635358	3,24224E-05	8,14322E-05
RBM45	0,347752452	3,644660638	3,263E-05	8,19235E-05
PEBP1	-0,580554586	8,160046416	3,2698E-05	8,20423E-05
SMG7	0,586920479	7,49109845	3,27014E-05	8,20423E-05
C7orf49	0,564511535	5,810290536	3,28992E-05	8,24955E-05
ZC3H6	-0,55815163	4,089999402	3,29062E-05	8,24955E-05
RASA4	-0,695737224	5,570476416	3,36635E-05	8,43629E-05
KIAA1598	-0,597741701	5,907790163	3,38021E-05	8,46791E-05
SRC	0,624401223	6,86427458	3,3976E-05	8,50835E-05
SUMO1P3	0,565028361	3,064980527	3,4073E-05	8,5295E-05
FNIP1	-0,415283471	5,140673317	3,43632E-05	8,599E-05
SLC31A1	-0,521007277	6,843546969	3,45703E-05	8,64765E-05
RFX1	-0,497405757	4,404540515	3,46085E-05	8,65402E-05
GLYR1	-0,452864696	6,821804656	3,46664E-05	8,66534E-05
ZNF44	-0,529585481	3,543032936	3,49325E-05	8,72864E-05
PDE4B	-1,110496423	5,345415935	3,50571E-05	8,75658E-05
APH1A	0,462602755	8,661125453	3,54341E-05	8,84749E-05
BCAP29	-0,538269412	6,222744081	3,59256E-05	8,96694E-05
KTI12	0,503265078	3,945647359	3,60378E-05	8,99164E-05
CASK	0,746041336	6,872061471	3,6407E-05	9,08045E-05
CCDC88A	-0,635537466	5,916811798	3,6537E-05	9,10953E-05
COMMD2	0,823216255	6,680365453	3,65788E-05	9,11661E-05
ADM	1,577564274	6,904223069	3,6606E-05	9,12008E-05
TMEM127	-0,393029512	7,284915413	3,66644E-05	9,13128E-05
UQCRFS1	0,776013154	7,306085014	3,7294E-05	9,2847E-05
HINFP	0,462904475	4,616654287	3,78716E-05	9,42506E-05
C7orf60	0,728458974	4,885232256	3,81768E-05	9,49547E-05
PAPOLA	0,383208318	8,115016166	3,81824E-05	9,49547E-05
EXOSC8	0,704646422	4,802582732	3,82329E-05	9,50457E-05
TMEM231	-0,8424693	4,609775645	3,8252E-05	9,50585E-05
RPL24	0,8703153	10,04463117	3,85349E-05	9,57265E-05

ELAVL1	0,431569549	6,989724197	3,8985E-05	9,6793E-05
SYT11	-0,978900559	4,960127537	3,89926E-05	9,6793E-05
DKK3	-0,903000672	7,402847354	3,90902E-05	9,70001E-05
RECQL5	0,615841173	5,07071322	3,9489E-05	9,79539E-05
ZNRF2	0,664783125	5,402287356	3,96827E-05	9,83986E-05
DSTYK	-0,502287466	5,299396602	4,00865E-05	9,93637E-05
ZNF33A	-0,524453537	5,258860166	4,04623E-05	0,000100259
TMEM65	0,727629248	5,366387391	4,05312E-05	0,000100393
FXC1	-0,487994043	5,673249397	4,05787E-05	0,000100474
TMEM41B	-0,464071712	5,984018037	4,09564E-05	0,000101373
BAHD1	-0,535287226	5,639812149	4,10939E-05	0,000101676
GRB2	-0,372909465	7,983250938	4,12113E-05	0,00010193
TCERG1	0,487291457	5,832792223	4,13778E-05	0,000102304
RNF181	0,666989889	6,722493408	4,15261E-05	0,000102634
PLA2R1	1,360256242	4,950216274	4,1832E-05	0,000103352
PDF	0,515283696	3,241406752	4,21586E-05	0,000104121
RAD52	0,678516666	3,284796627	4,23369E-05	0,000104524
SPPL3	0,459103611	6,750656332	4,2655E-05	0,000105271
FLJ10038	-0,683614809	3,589819254	4,26961E-05	0,000105335
RAB31	-0,836009517	7,579846316	4,27286E-05	0,000105377
FZR1	0,569569987	6,069291184	4,30371E-05	0,000106099
NOTCH3	1,073887564	8,736029841	4,32768E-05	0,000106651
USP38	-0,510086629	5,547282532	4,34918E-05	0,000107143
CD99L2	-0,891822462	6,295278484	4,35384E-05	0,000107219
SLFN5	-0,7776435	5,849394288	4,37941E-05	0,000107809
SFPQ	0,432286293	8,400984886	4,39084E-05	0,000108052
NDUFA10	0,416392619	6,792431554	4,40328E-05	0,000108309
CDH1	0,812786646	9,084365567	4,40447E-05	0,000108309
FKBP14	0,686633599	4,584704946	4,41713E-05	0,000108581
CXADR	1,140421161	5,94881915	4,43755E-05	0,000109044
FBXO7	-0,375049739	7,169313081	4,43954E-05	0,000109053
UBE2A	0,720823355	7,148805151	4,46532E-05	0,000109608
POP4	0,690977194	5,716018331	4,46533E-05	0,000109608
MLL	-0,653727508	6,359856251	4,47422E-05	0,000109787
NELF	0,793333014	5,853341453	4,48892E-05	0,000110108
CTDSP2	-0,550597855	8,55170474	4,51646E-05	0,000110743
AACS	0,736978023	6,059570309	4,52761E-05	0,000110977
RAD23A	0,619595859	7,743338836	4,5865E-05	0,00011238
ZBTB40	-0,549736323	5,200853019	4,60969E-05	0,000112908
SOD1	0,673904135	7,907967868	4,68508E-05	0,000114713
TECPR2	-0,557065472	5,128495699	4,75467E-05	0,000116375
QTRT1	0,678806465	4,947893021	4,76454E-05	0,000116575
SERPINH1	0,880764967	8,787810789	4,77801E-05	0,000116862
SLC25A40	0,630233255	4,015831889	4,78036E-05	0,000116878
WDR35	-0,586209133	4,449922274	4,78417E-05	0,000116929
KIAA0913	-0,534677212	6,426642331	4,78601E-05	0,000116932
ILKAP	0,537634691	5,110880301	4,79015E-05	0,000116991
MYO6	-0,613412871	6,843932291	4,82425E-05	0,000117782
ZMIZ1	-0,68013397	7,286587946	4,87406E-05	0,000118956
COPS7A	0,581826622	7,199786221	4,91768E-05	0,000119973

CC2D1B	-0,438078752	5,785966457	4,91924E-05	0,000119973
PAK2	0,576350361	8,24530634	4,96943E-05	0,000121153
MAP7D1	-0,563708844	7,098804038	4,97154E-05	0,000121161
HSPA1B	0,870102531	8,636363773	5,13792E-05	0,000125171
RNF213	-0,721444706	7,882146367	5,17124E-05	0,000125938
ZZZ3	0,4510883	5,705075173	5,18461E-05	0,000126219
ACTR8	-0,363271983	4,5588665	5,18781E-05	0,000126251
WDR6	-0,508083321	6,870026257	5,2055E-05	0,000126637
MTHFD1L	0,67793701	5,909074853	5,21239E-05	0,000126759
CDC42SE1	0,463293428	8,157214029	5,23684E-05	0,000127308
TBCB	0,658145385	6,620644002	5,25171E-05	0,000127624
TMEM106B	0,856361626	7,097025477	5,26207E-05	0,000127831
TMCO1	0,692868746	7,430180031	5,34996E-05	0,00012992
NUP133	0,419660638	6,462922355	5,39734E-05	0,000131023
DNAJB6	0,535115725	7,794537432	5,51691E-05	0,000133878
UTP15	0,429450305	4,286626239	5,52481E-05	0,000134022
SUMF1	-0,531275265	5,645345608	5,56497E-05	0,000134949
FNBP1L	-0,676428476	6,003647913	5,60141E-05	0,000135784
GEM	-1,045485869	5,278170006	5,68289E-05	0,00013771
FAM129A	-0,836786692	6,757695109	5,74315E-05	0,000139121
RPS16	0,936225741	10,26528921	5,75032E-05	0,000139245
PKN1	-0,717805125	7,160557151	5,79353E-05	0,000140242
SLC4A2	-0,53804245	6,869904299	5,79658E-05	0,000140266
NASP	0,635868628	7,158959151	5,81663E-05	0,000140701
RPN2	0,526492763	9,414884433	5,82946E-05	0,000140961
SEC24B	-0,380985136	5,928489933	5,85905E-05	0,000141627
SDHAP2	0,962030943	4,623958391	5,87375E-05	0,000141932
HES4	1,255558901	2,89291156	5,92377E-05	0,00014309
PLEKHM1	-0,582767059	5,785079183	5,93632E-05	0,000143342
KDELC1	0,791523555	3,62740571	5,94604E-05	0,000143526
MLL2	-0,680770168	6,848172752	5,9967E-05	0,000144697
ZNF121	0,814211293	2,851208431	6,03001E-05	0,00014545
PPAPDC1B	1,010679622	6,053978038	6,04892E-05	0,000145854
HSP90AA1	0,562107849	10,90700633	6,05464E-05	0,000145941
PRPF8	-0,579612479	8,737060027	6,08746E-05	0,00014668
ATP5C1	0,684130083	7,795306255	6,21309E-05	0,000149654
FADS3	-0,842332205	4,689984495	6,2451E-05	0,000150372
PPIL3	0,747244195	4,515584009	6,26758E-05	0,00015086
CASP6	0,591879956	4,636306709	6,27773E-05	0,000151051
PAPSS1	-0,535909531	6,793246491	6,29909E-05	0,000151511
SLC12A2	-0,783517038	5,783341336	6,34615E-05	0,00015259
IFI16	0,870681033	8,529170735	6,3494E-05	0,000152614
RNF113A	0,558968714	4,408597969	6,35906E-05	0,000152792
RPS10	0,917884872	10,35672293	6,36958E-05	0,000152991
NBEAL2	-0,695125089	6,166522172	6,39979E-05	0,000153662
UBE2G1	0,452765419	6,652767009	6,47764E-05	0,000155477
SYS1	-0,471677913	5,988524202	6,4839E-05	0,000155572
PPP1R9B	-0,514084927	6,650020896	6,49113E-05	0,000155691
ZNF282	0,585541203	5,691806854	6,51002E-05	0,000156089
RPL30	0,598967671	9,951301374	6,5332E-05	0,00015659

CD9	1,076970978	10,5533288	6,55437E-05	0,000157042
FAM86A	0,526734778	4,273847114	6,59041E-05	0,00015785
TNFAIP1	-0,491808346	7,121563641	6,60602E-05	0,000158169
PTEN	-0,584525642	6,720669977	6,62027E-05	0,000158454
PHAX	-0,351376229	4,526553576	6,62835E-05	0,000158592
LLGL1	0,614830461	5,852355413	6,63601E-05	0,000158701
C11orf24	0,530419275	5,855309015	6,63757E-05	0,000158701
CHRAC1	0,489509085	6,114660872	6,65369E-05	0,000159031
GNPDA1	0,499048088	6,406554692	6,671E-05	0,000159389
CNNM3	-0,475991775	5,728448225	6,69163E-05	0,000159826
GPBP1	-0,353640009	6,672047358	6,7035E-05	0,000160053
HEBP1	-0,498603275	5,786006098	6,72143E-05	0,000160425
HYOU1	0,642034946	8,627013377	6,86316E-05	0,00016375
APIP	-0,501619925	4,719915081	6,88666E-05	0,000164254
SHROOM1	-0,584373485	3,728349133	6,89184E-05	0,00016432
GTF2F2	0,549378716	5,132414556	6,90474E-05	0,00016457
BOK	-0,879841087	5,690233685	7,00853E-05	0,000166985
SMN2	0,629724609	6,116559436	7,07287E-05	0,000168459
APBA3	0,587804253	4,172269943	7,08616E-05	0,000168717
SYNJ2	0,689432992	4,900149242	7,12452E-05	0,000169571
SRF	-0,449077804	6,67751775	7,14859E-05	0,000170085
PDS5B	-0,489931335	5,539917125	7,17502E-05	0,000170654
ITGA6	1,252278431	8,952280664	7,20147E-05	0,000171223
PKD1	-0,719901643	6,345053943	7,28658E-05	0,000173187
INSR	-0,676638113	6,222634501	7,30266E-05	0,000173508
SDHAF1	0,734239523	4,125917562	7,32351E-05	0,000173943
FAM102A	0,798090279	7,273695386	7,34958E-05	0,000174501
UBE2E2	-0,594678741	4,850724347	7,38145E-05	0,000175171
CUL2	0,504016412	5,961187636	7,3845E-05	0,000175171
SOD2	-0,754932756	9,103585143	7,38547E-05	0,000175171
C10orf88	0,473183329	3,733456017	7,44389E-05	0,000176495
PTAR1	-0,502286316	5,503697842	7,47485E-05	0,000177167
WIPF2	-0,440117015	6,341688665	7,55071E-05	0,000178903
C16orf13	0,642262202	5,493030183	7,6509E-05	0,000181214
AASDHPPT	0,525453799	6,0271959	7,67398E-05	0,000181697
AES	-0,472521131	8,766845364	7,68153E-05	0,000181813
SGCB	-0,663653239	5,756968563	7,75462E-05	0,000183479
FMOD	-0,907256377	6,665459586	7,79462E-05	0,000184332
TUBA1B	0,484430722	10,79091465	7,79607E-05	0,000184332
SH2D4A	-0,744363271	4,762364218	7,86146E-05	0,000185814
RARRES2	-1,032485137	6,653622243	7,89753E-05	0,000186602
FLYWCH1	0,724095351	5,641294787	7,92365E-05	0,000187154
SSR3	0,652749764	8,547244912	7,93762E-05	0,000187397
DNTTIP1	0,734185841	5,749736368	7,93942E-05	0,000187397
MTHFD1	0,606294352	6,851691149	7,94779E-05	0,000187529
MRPL16	0,444603596	5,544951887	8,1852E-05	0,000193064
SEMA3F	0,868119725	6,808560783	8,21187E-05	0,000193626
ERGIC3	0,555366394	8,603037512	8,2431E-05	0,000194296
RHEB	0,646079298	7,552836033	8,25239E-05	0,000194447
HSPA4	0,450056562	7,509679421	8,27114E-05	0,000194822

NUPL1	-0,430175694	6,389861694	8,28745E-05	0,000195138
PTGS1	-0,934160382	4,978637668	8,29527E-05	0,000195255
LEO1	0,487195776	5,097721056	8,38905E-05	0,000197394
TBC1D9B	-0,451980432	7,332528438	8,44064E-05	0,00019854
C2orf43	0,759486442	5,045145829	8,48789E-05	0,000199583
CYP20A1	-0,360073422	5,90478319	8,49939E-05	0,000199784
HGS	0,488238189	7,110597868	8,54218E-05	0,000200721
ABL2	-0,534835482	5,864754855	8,64011E-05	0,000202952
TXNL4A	0,578803492	5,772714089	8,71589E-05	0,000204661
RPS19BP1	0,643840202	6,375432125	8,765E-05	0,000205653
TAGLN	-0,960497577	8,461639202	8,76564E-05	0,000205653
ENTPD6	0,613030558	6,8015557	8,76719E-05	0,000205653
DCK	0,776433444	5,887435407	8,79427E-05	0,000206218
MLXIP	-0,615747614	6,02583037	8,9322E-05	0,00020938
SF3B5	0,68050535	6,905810475	8,95144E-05	0,000209751
IGF2R	-0,59913826	8,249974857	8,95417E-05	0,000209751
ACAP3	0,829464455	5,415417371	8,97246E-05	0,000210107
RASA1	-0,510516078	5,424830761	9,01441E-05	0,000211017
MARCH5	0,484801682	6,168883997	9,05781E-05	0,00021196
LRBA	-0,64473969	6,374554542	9,20651E-05	0,000215366
PPP1R16A	0,783006268	5,150879965	9,21861E-05	0,000215575
C12orf75	1,374574089	5,284718542	9,23515E-05	0,000215888
PTPN12	-0,519512516	7,237235669	9,26472E-05	0,000216505
PTOV1	0,521756693	6,929969018	9,29368E-05	0,000217078
SLC20A2	0,994451056	7,301832729	9,29561E-05	0,000217078
TRPM2	1,017651322	4,519366255	9,31648E-05	0,000217491
SEC63	-0,428027717	7,16722037	9,3901E-05	0,000219135
POLR1D	0,63638152	7,186695301	9,47237E-05	0,000220979
ABCD4	-0,441856432	5,059694748	9,52775E-05	0,000222195
EHMT2	0,611409264	6,575541999	9,62966E-05	0,000224495
HOOK3	-0,492587202	6,259955524	9,72232E-05	0,000226577
SF3A2	0,647713985	6,398569956	9,77682E-05	0,000227769
BCL2L11	0,600597288	6,020482066	9,86471E-05	0,000229739
HRSP12	0,677959182	4,325962494	9,91088E-05	0,000230735
KDELR1	0,487260486	8,194967032	0,000100019	0,000232775
ADNP	0,491671998	7,393643271	0,000100126	0,000232944
DDX1	0,453501773	7,529232853	0,000100606	0,00023398
EPB41	-0,516155012	6,180033175	0,000100724	0,000234176
ANP32E	0,60358866	6,930479859	0,000100786	0,00023424
IDH1	0,820499989	8,062271124	0,000101281	0,000235311
RAP2A	-0,49274474	6,149985223	0,000101494	0,000235725
PLEKHA4	-0,816487407	4,739033126	0,000101737	0,00023621
DENND4A	-0,561120328	5,346500918	0,000101798	0,000236269
F2R	-0,719177334	6,272634087	0,000102406	0,0002376
TCIRG1	-0,724352948	6,29544548	0,000103156	0,000239259
BLMH	0,914270736	6,466128615	0,000103921	0,000240951
CDCA7L	0,972240025	6,133019176	0,000104028	0,000241117
SIGIRR	-0,698998827	4,756683442	0,000104184	0,000241396
NUBP1	-0,406431124	4,726813952	0,00010469	0,000242487
C3orf14	1,043337571	3,887933905	0,000105418	0,00024409

SAT2	-0,552539613	5,281098888	0,000105456	0,000244095
PPFIBP1	-0,625534277	6,681107753	0,000105655	0,000244473
SEPHS1	0,438842731	6,203569909	0,000105772	0,000244661
ARHGEF7	-0,453917742	6,003234664	0,000105946	0,00024498
GAS5	1,065916079	8,062407028	0,000106142	0,000245309
NDRG2	-0,956961869	6,539312074	0,00010616	0,000245309
CREBZF	0,623005169	6,021416656	0,000107504	0,000248331
UBXN4	0,435317674	7,867028457	0,000108178	0,000249803
SNX21	-0,550060566	4,50595196	0,000109198	0,000252072
TWF2	-0,518360555	5,742715298	0,000109699	0,000253059
PPP2R5D	0,42818054	6,515769407	0,0001097	0,000253059
GSN	-0,880473797	9,393726846	0,000110156	0,000254025
NDUFAF4	0,642999494	4,370372313	0,00011051	0,000254756
STK35	0,601360806	6,527769507	0,00011061	0,000254899
TMUB1	0,665774401	5,441977095	0,000110951	0,0002556
DDX60	-0,901325461	5,648738604	0,000111643	0,000257107
SEC61A1	0,449806408	9,460621136	0,000111866	0,000257533
PSMA6	0,774979645	7,764433253	0,000112043	0,000257854
PSMD13	0,516774452	7,601773317	0,000112721	0,000259326
PCGF6	0,472686794	4,19526209	0,000112808	0,00025944
RBM19	0,884099634	5,982416572	0,000112985	0,000259759
SLC2A4RG	0,720135294	6,358793842	0,000113028	0,00025977
RGS19	-0,577192955	4,652667848	0,000113626	0,000261057
TBC1D13	-0,511080134	5,476532407	0,000114352	0,000262636
C1orf56	0,508806009	4,030636642	0,000114472	0,000262823
DPF2	0,357690215	6,392543506	0,000115308	0,000264653
NUP54	0,638426434	6,044241049	0,000116522	0,000267349
POLR3F	0,517593051	4,387155026	0,000116643	0,000267536
PRMT7	0,502826828	5,056200417	0,000116793	0,000267791
UBE2J2	0,496747431	5,936090433	0,000117168	0,00026856
SUMO1	0,523298201	7,337257524	0,000118051	0,000270493
CXCL10	1,954795464	6,556071294	0,00011965	0,000274066
HSP90B1	0,4985643	10,43160348	0,000120038	0,000274861
CCDC124	0,542193644	6,094220484	0,000120701	0,000276288
WBP1	0,520380279	6,310163182	0,000120854	0,000276515
OXCT1	0,919949061	5,940922958	0,000120881	0,000276515
NMRAL1	0,700851096	5,670179616	0,000121649	0,000278176
SLC23A2	-0,57692428	5,672708188	0,000122472	0,000279965
SLC9A1	-0,643654919	6,562103506	0,000122949	0,000280963
H3F3A	0,569346659	9,895186876	0,000123834	0,00028289
TCEB2	0,639986192	7,280751382	0,000125588	0,0002868
CALD1	-0,796481336	8,272384865	0,000126028	0,000287709
ABL1	-0,525113349	6,760023129	0,000127167	0,000290211
LRIG3	0,920679288	6,219218391	0,000127294	0,000290404
FAP	1,341307212	5,380042278	0,000129862	0,000296118
C9orf142	0,686269372	4,801118482	0,000129885	0,000296118
PARP8	-0,650420814	4,624586065	0,000130232	0,000296809
PAAF1	0,619843924	4,916830913	0,000130438	0,00029718
LPCAT4	0,859102838	5,724477287	0,000131024	0,000298413
PABPN1	0,459758636	7,176404541	0,000131167	0,00029864

SSR1	0,41565819	8,397400392	0,000132329	0,000301186
PNPLA4	0,84346902	5,033946491	0,000132466	0,000301397
UBE2N	0,521880096	7,40642956	0,000133907	0,000304573
SDHA	0,588353275	7,598825455	0,000134352	0,000305483
VPS18	-0,440289061	5,672947698	0,000135978	0,000309003
RNF10	-0,346635928	7,725271396	0,00013599	0,000309003
SBNO1	0,590976416	5,468149819	0,000136586	0,000310254
TTC5	0,77972752	4,694630999	0,000136928	0,000310927
ARPC5L	0,481780681	6,199682134	0,000137935	0,000313012
C5orf51	0,537187002	6,154106599	0,000137938	0,000313012
STON1	-0,817362748	4,65640979	0,000138336	0,000313811
TMX1	0,496942437	6,708786344	0,000138739	0,000314619
ANXA4	-0,568383789	7,483758949	0,000138813	0,000314683
TNKS2	-0,375030781	6,673768402	0,00013955	0,000316249
LEPROT	-0,65224424	5,927562801	0,000139619	0,000316299
COMMD4	0,513560442	6,042564758	0,000140475	0,000318132
SLC40A1	-0,997886933	7,973531337	0,000141513	0,000320377
SOX9	1,512476702	5,773815207	0,000141751	0,000320809
LRRC59	0,473629866	8,149721779	0,000142944	0,000323403
DSEL	-0,928203087	4,209309375	0,000143487	0,000324523
IGFBP7	-0,816619024	8,64602037	0,000143784	0,000325087
TXNDC15	-0,498091203	6,040181751	0,000144137	0,000325777
TBCA	0,593637947	6,736964256	0,000145029	0,000327685
ZNF507	0,567714158	5,370297264	0,000145223	0,000328014
LCMT1	0,44955838	5,319493452	0,000146301	0,000330341
GIGYF2	-0,45157832	6,404319398	0,000146489	0,000330654
LOXL1	1,029579917	5,567167631	0,000146704	0,00033103
DUSP22	-0,63859905	5,784965891	0,000147408	0,000332509
KRTCAP2	0,57708811	6,886596815	0,0001486	0,000335087
RPL28	0,705139776	9,941300498	0,000148684	0,000335167
E2F4	0,472513233	6,771699121	0,000149032	0,000335838
SLC4A1AP	0,365775814	5,861924156	0,000149529	0,000336847
DDX28	0,580343857	4,680154474	0,000150001	0,000337801
MINK1	-0,497749202	7,15765202	0,000150772	0,000339423
C4orf34	-0,627962641	5,377998911	0,000151427	0,000340786
TRAK1	-0,54740428	6,47799839	0,000152335	0,000342716
TRAM1	-0,572304214	8,747554746	0,000155273	0,000349211
ICK	0,826067825	6,209629485	0,000156358	0,000351535
DCTN1	-0,433423771	7,977197407	0,000156514	0,000351769
RNASEK	-0,449974219	5,365756748	0,00015817	0,000355374
NUDCD2	0,580805565	4,895328231	0,000159589	0,000358445
ATG5	0,557974956	6,291729198	0,000160018	0,000359289
FAM76A	-0,438561321	4,283194231	0,000160797	0,000360921
TAF10	0,475967879	6,015646419	0,00016149	0,000362356
CHPT1	-0,707198568	5,748955932	0,000162473	0,000364442
NKG7	-1,077204076	4,138156429	0,000162997	0,000365498
BIRC3	-1,128853991	5,648985263	0,000163186	0,0003658
LOC100216545	1,038878646	2,661353211	0,000165712	0,000371342
ARHGEF35	1,050225935	5,064969137	0,000166832	0,000373728
PCGF3	0,614254629	6,092112864	0,00016762	0,00037537

PLOD1	0,754403761	8,222633548	0,000168821	0,000377936
SLC7A6OS	0,491715129	3,564719172	0,000170555	0,000381691
RPS3	0,773782892	11,0675673	0,000171424	0,00038351
SUN1	0,498175771	7,505726609	0,000171709	0,000384022
ATP5D	0,617141834	5,924171425	0,000171976	0,000384413
ABCD3	-0,535064871	6,207514285	0,000171997	0,000384413
XPNPEP1	0,451856498	6,514644188	0,000175698	0,000392558
ATG7	-0,397266336	5,778551835	0,00017643	0,000394064
RIOK2	-0,353899748	4,650979199	0,000176924	0,000395038
ECSIT	0,569815918	5,708502555	0,000178791	0,000399077
PRR3	0,589877332	4,189102179	0,000179071	0,00039957
IFITM3	-0,749940471	9,167188006	0,00018088	0,000403475
JTB	0,536068751	7,650678938	0,000181523	0,000404776
NDUFB10	0,512352309	6,349453951	0,000184753	0,000411843
CAPZA1	0,483730002	8,289712796	0,000184824	0,000411868
MKLN1	-0,328363329	6,310982083	0,000185188	0,000412543
IAH1	0,69004566	5,770455263	0,000188174	0,000419058
CDC42BPB	-0,56140769	7,541737059	0,000189463	0,000421791
UBR2	-0,396745085	5,716764911	0,000192548	0,000428521
DCAF8	-0,37275282	6,76770048	0,000192903	0,00042917
SLC25A36	0,655813155	6,918527941	0,000194176	0,000431861
TXN2	0,503440724	6,867762971	0,000195938	0,000435638
LIN37	0,563608582	3,526025592	0,000196129	0,00043592
MT2A	-1,02416301	7,655890616	0,000198425	0,00044088
LIN7C	0,488940159	6,041477509	0,000198635	0,000441202
NEDD1	0,908780165	5,652736139	0,000199654	0,000443322
POLRMT	0,58703471	5,846649537	0,00020009	0,000444145
JAK2	-0,800410251	4,834035171	0,000202401	0,000449128
SLC39A14	0,846846915	6,900402042	0,00020291	0,000450111
EIF3E	0,584835172	9,026073866	0,000203573	0,000451435
GSK3A	0,480448557	6,961617298	0,000204406	0,000453135
POLR3H	-0,440402627	6,4286745	0,000205802	0,000456081
PHF20L1	0,398758506	6,145359574	0,000209247	0,000463565
HCCS	0,549127081	5,371441888	0,00021033	0,000465813
RALGDS	-0,52994834	6,803288863	0,000210436	0,000465898
NKIRAS1	-0,449364081	3,491331469	0,000211167	0,000467365
NUDT16L1	0,663636965	5,188959166	0,000211255	0,000467406
GNPTAB	-0,443722557	6,883396849	0,000213951	0,000473218
COPS2	0,502444808	7,005942565	0,000214889	0,000475138
PPP1R10	-0,437548	6,756053438	0,000215102	0,000475456
GALNT1	0,745619298	7,822082712	0,000215223	0,00047557
ZRSR2	-0,532785826	3,567115008	0,000216073	0,000477293
PIH1D1	0,446722549	6,040783874	0,000216356	0,000477763
DDX27	0,520232736	6,455050488	0,000218081	0,000481415
CCDC90B	0,574126716	5,701015529	0,000218266	0,000481668
OXSM	0,553602619	3,598955831	0,000218434	0,000481883
NPAS2	1,051716685	5,245162428	0,000218635	0,000482078
TMEM175	-0,607412489	4,31981096	0,000218664	0,000482078
ABI1	0,474028631	7,18009214	0,00021926	0,000483237
SLC39A4	0,89800125	5,350687227	0,000219504	0,000483618

FAM188A	-0,492733535	4,53060546	0,000222231	0,000489468
ZCCHC7	0,594545455	5,078237252	0,000224007	0,000493221
MAD2L1BP	0,473798792	5,090911965	0,00022448	0,000494103
GOLM1	0,794586002	6,962796912	0,00022533	0,000495814
NBPF14	-0,632657342	4,719709304	0,000226502	0,000498231
PRKACA	-0,384243167	6,7554419	0,000227103	0,000499392
C17orf70	0,536154252	5,64939339	0,000227186	0,000499414
TOMM34	0,563860538	6,071227589	0,000227503	0,00049995
SGK3	-0,434203041	5,064025258	0,000227874	0,000500604
NHP2L1	0,485579467	7,5747456	0,00022978	0,000504628
BRD9	0,602627519	6,153225041	0,000230685	0,000506453
RPS8	0,735432773	10,61323735	0,000231536	0,000508157
CAPRIN1	0,429192816	8,712167711	0,00023163	0,000508201
CCDC93	-0,448808547	5,456819401	0,000232107	0,000509082
MT1E	-1,186934126	5,588143906	0,000232582	0,000509959
CSF2RB	-1,139067024	5,338149683	0,000232746	0,000510157
DGCR6L	0,776622958	6,138426724	0,000233085	0,000510734
KDELR2	0,548791169	8,807052958	0,000233172	0,000510761
NEK1	-0,579166161	4,291629877	0,000233465	0,00051124
LGALS9	-0,819228458	6,878418222	0,000234036	0,000512325
MUS81	0,424890547	5,255568181	0,000235196	0,000514699
RAVER1	0,567561305	6,564833907	0,000235832	0,000515925
ARHGAP21	-0,547498981	6,810650771	0,000237235	0,000518827
ATAD2B	0,500827882	4,614250073	0,000239258	0,000523085
EIF2AK2	0,634797572	5,674503736	0,000239364	0,000523148
KIAA1731	0,570260518	4,445080299	0,000240588	0,000525655
KLHDC3	0,561009625	7,252389272	0,000240684	0,000525697
CRKL	0,603502311	7,833947395	0,000242801	0,00053015
PLAUR	-0,77838753	6,498786278	0,000245478	0,000535824
KIAA1468	0,563170627	5,602293651	0,000246098	0,000537006
RBM42	0,536554576	6,568925156	0,000246486	0,00053768
PRKRIP1	0,425645393	5,018495664	0,000246777	0,000538144
FAM193A	-0,479606754	5,139587208	0,000249015	0,000542849
SIL1	-0,408467407	5,954258176	0,000250461	0,000545618
FAS	-0,798282432	5,207076739	0,000250473	0,000545618
LOC387647	-0,533962138	4,683716805	0,000250525	0,000545618
TRIP6	0,743724566	6,810886747	0,000250777	0,000545992
ZNF558	0,703727394	4,711745951	0,000251111	0,000546544
TRIM4	-0,433276927	5,645883296	0,000251547	0,000547319
ZNHIT1	0,765371639	6,560508901	0,000251718	0,000547516
MANBA	-0,520255978	5,90856592	0,00025231	0,000548629
VPRBP	-0,36648691	5,735011737	0,000252478	0,000548821
CNBP	0,45502249	9,475404151	0,000253136	0,000550076
COMMD5	0,485921284	5,409009472	0,00026024	0,000565331
SAMD4B	0,629976885	7,290207481	0,000263123	0,000571413
YRDC	0,603393398	5,298274698	0,000263803	0,000572707
PSMD1	0,37366519	7,472437214	0,00026448	0,000573994
ST3GAL1	-0,787713791	6,942829167	0,000265888	0,000576866
NSUN3	0,771197322	3,78557061	0,000269555	0,000584635
CCDC130	0,585491372	4,610153466	0,000269977	0,000585366

CCDC66	-0,395895442	3,643580913	0,000270323	0,000585928
DPY19L4	0,519867259	6,187595565	0,000270553	0,000586241
SMURF2	-0,497833971	5,497552479	0,000271557	0,00058823
FAM102B	-0,519767312	5,148094623	0,000273173	0,000591363
ELMO2	-0,420691691	5,974757599	0,000273177	0,000591363
ATXN10	0,500868324	7,683224857	0,000273594	0,000592077
INO80D	-0,45859869	4,551233602	0,000274456	0,000593756
YARS	0,409703565	7,392576344	0,000275358	0,000595518
TTLL4	0,738655211	5,409889152	0,000279059	0,00060333
RANBP10	-0,42892275	5,224471853	0,00027917	0,000603379
CD14	-0,800760388	6,916073324	0,000280352	0,000605606
TMEM219	-0,509715684	6,755440438	0,000280378	0,000605606
LRRC58	0,597709395	6,745988598	0,000281633	0,000608124
ZNF789	0,642502832	2,612019266	0,00028297	0,000610817
RPIA	0,604501293	5,378596426	0,000283984	0,000612813
C12orf44	0,528271046	6,285927083	0,000285892	0,000616734
PQBP1	0,522220943	6,264862331	0,000286409	0,000617654
CRELD2	0,59848435	5,899634633	0,000289935	0,000625062
NDUFAF3	-0,41559182	5,76315292	0,000291176	0,000627538
TFRC	1,243975625	10,16466421	0,000296382	0,000638556
RAB11FIP3	-0,616751148	5,547186508	0,000298897	0,000643772
POLR2B	0,484798073	7,369448719	0,000299326	0,000644492
SIRT6	0,438983349	4,294231218	0,000299572	0,000644818
MRPL32	0,620655725	5,803586731	0,000299873	0,000645262
KHDRBS1	0,29385977	7,943968268	0,000300172	0,000645701
SEPHS2	0,607282274	7,01546793	0,000300624	0,00064647
ZNF768	0,521832354	5,720977879	0,000301992	0,000649207
C9orf89	0,678372273	4,591850452	0,000303462	0,00065216
EPC2	-0,383661729	5,216819512	0,000304507	0,000654201
XRCC5	0,386223898	8,944337297	0,000305006	0,000655067
SGK196	0,956209273	2,218772978	0,000307301	0,000659787
ACD	0,468542803	4,759371144	0,000308345	0,00066182
WDR5B	0,585259928	4,337995591	0,000310327	0,000665865
BET1	0,701779157	5,13434171	0,000313713	0,000672919
TMEM57	-0,446015223	5,35122192	0,000314532	0,000674463
ZNF260	0,565255148	5,277429178	0,000316697	0,000678892
CSNK1E	0,453612001	7,285664023	0,000316928	0,000679175
MMAA	-0,444078833	3,589155554	0,000320424	0,000686449
ITGAV	0,767782645	8,305273468	0,000323769	0,000693399
CCNG1	-0,517638735	6,444703716	0,000324123	0,000693939
STK38L	0,656743733	6,524559127	0,000328418	0,000702913
SPRED2	-0,517481337	6,143770294	0,000329124	0,000704204
CCNB1IP1	0,73192483	6,085082111	0,00032972	0,000705257
MED13L	-0,567661306	6,925926746	0,000330772	0,000707285
GRWD1	0,402066204	5,974959009	0,00033202	0,000709732
NRP2	-0,868788946	6,624060892	0,000332668	0,000710895
RP9	0,499883448	3,814259736	0,000333002	0,000711384
TMEM203	0,429332196	5,90460395	0,000333665	0,000712578
DCAF5	-0,430371802	6,401556443	0,000336009	0,000717359
NDUFB7	0,859563305	6,526458358	0,000337221	0,000719721

MARK4	0,523830711	5,799430022	0,000341245	0,000728082
METTL4	0,58612817	4,317607668	0,000344158	0,000734068
G0S2	-1,137278082	4,420545773	0,000346527	0,000738889
ESYT2	-0,416909504	7,543532575	0,000346923	0,000739501
TMEM185B	0,517445674	5,66646227	0,000347775	0,000741087
MRPL10	0,418023658	6,062854879	0,000348241	0,000741847
ENO2	1,114911195	6,948690472	0,000348463	0,000742089
PRKRA	0,46686799	6,221455411	0,000350425	0,000746034
ECHS1	0,544829863	7,358662647	0,000352589	0,000750407
KLHL24	0,831711067	7,337224684	0,000352887	0,000750808
DHRS7	-0,569970837	6,293086897	0,00035408	0,00075311
MTFMT	0,387490317	4,516448187	0,000356747	0,000758492
SNX6	0,533639277	7,384391837	0,000356833	0,000758492
THAP4	0,43617603	6,193100189	0,000357095	0,000758813
TMEM109	-0,430636207	7,723074973	0,000358189	0,0007609
ZNF37A	-0,542356311	4,422055969	0,000360806	0,00076622
RPL13AP20	0,608515023	5,158371724	0,000361263	0,000766954
NDUFA13	0,691134457	7,603910926	0,000362415	0,000769159
CCDC88B	-0,647467231	4,474549091	0,000364013	0,00077231
CPSF1	0,603118707	6,754663225	0,000364285	0,000772647
EIF2B2	0,463862366	5,490499273	0,000364438	0,00077273
TMBIM4	-0,554241892	6,377622133	0,000365801	0,00077538
HPS1	-0,380197728	6,381175236	0,00036599	0,00077554
TRAF3IP1	-0,415512203	4,901395268	0,000366308	0,000775972
HIVEP1	-0,613196571	5,545562655	0,000366981	0,000777157
MRPL22	0,605777022	4,803596437	0,000367642	0,000778315
ATP9B	0,789339608	7,022896086	0,000371696	0,000786501
PARG	0,370261125	5,120328263	0,00037174	0,000786501
FMNL2	-0,547990132	6,362230324	0,000372005	0,000786817
UBL5	0,774411332	7,352764814	0,00037337	0,00078946
TSPAN15	-0,716152812	5,988559557	0,000375257	0,000793204
TMEM123	0,697780512	9,280577412	0,000376921	0,000796475
NDOR1	0,528207841	5,090949136	0,000377622	0,00079771
RBM14	0,324070912	6,14613788	0,000378818	0,000799988
RIT1	0,940183985	6,884122288	0,000379297	0,000800751
TMEM50B	-0,588998355	6,016474744	0,000380008	0,000802003
PXMP2	0,695174464	3,857923416	0,000384143	0,000810481
SLMAP	-0,33936022	5,885679672	0,000386282	0,000814741
IMPA2	0,955108543	6,635965877	0,000392128	0,000826814
PPAP2A	-0,676095191	5,494001933	0,000394405	0,000831359
ASTE1	0,52189871	4,120001699	0,000395444	0,000833101
MLH1	-0,342970637	5,448461001	0,000395503	0,000833101
BCL2L1	-0,485426936	7,606699918	0,000395598	0,000833101
MEIS1	-0,734585657	4,803685146	0,000395736	0,000833135
DGCR14	0,486211177	4,822898677	0,000397294	0,000836156
MCM3AP	-0,446351734	6,559482956	0,000402512	0,000846877
COPG2	0,542827951	6,116909142	0,000409491	0,000861296
COMMD3	0,677550504	5,718294369	0,000411086	0,000864384
STK38	0,535520253	6,706605191	0,000413563	0,000869324
PRELID1	0,532044643	7,198495357	0,000413968	0,000869906

NCKAP1	0,458821403	8,454914086	0,000415306	0,000872449
PEX13	0,556351172	6,44046114	0,000416419	0,000874519
NAA35	0,434413662	4,922853546	0,000416931	0,000875324
GNPTG	-0,465818602	5,913659041	0,000420803	0,000883182
AKT3	-0,890428286	5,237051917	0,000423377	0,00088831
KANK1	-0,697123914	5,879905945	0,000429025	0,000899884
IMPDH2	0,470864647	7,410013164	0,000434714	0,000911537
MAPK8IP3	0,85981761	5,541331277	0,000439857	0,000922037
RPRD1A	0,672349104	6,738001686	0,000441555	0,000925311
ZNF143	0,319665494	4,677335195	0,00044503	0,000932308
DKFZP586I1420	-0,570448583	3,511310029	0,000448593	0,000939483
CSTB	0,947342031	9,274855702	0,000449212	0,00094049
MYEOV2	0,770767005	5,545404608	0,000454239	0,000950723
TMEM14B	0,77545734	6,745807132	0,000463995	0,000970845
ANKRD13C	-0,31142747	4,952236586	0,000464698	0,000972018
C7orf13	1,057515483	3,579860837	0,000468013	0,00097865
SLC25A22	0,622363197	5,225172008	0,000468397	0,000979153
NBPF9	-0,545754841	4,710279588	0,000470343	0,000982921
C21orf91	0,973558583	5,979270632	0,000471204	0,000984417
ARHGEF10L	-0,614028042	5,132129502	0,000471696	0,000985145
TNRC6C	-0,642616107	4,483595899	0,000474292	0,000990263
SLC10A7	-0,434200494	3,827287972	0,000476774	0,00099514
DECR2	0,721645016	4,190463445	0,000478118	0,000997639
C1orf43	0,560122966	8,667461124	0,000479578	0,00100038
CALR	0,406501951	10,70901354	0,00048142	0,001003915
SSNA1	0,507254473	5,9985456	0,00048366	0,001008278
ZNF12	0,406559452	5,596939216	0,00048436	0,00100943
RPL12	0,569119877	10,03155344	0,000487736	0,001016092
RARRES3	-0,96235054	5,891517496	0,000487855	0,001016092
RNF215	0,565619033	4,206796556	0,000488654	0,001017412
LAS1L	0,410218855	5,871281216	0,000488788	0,001017412
ASCC2	0,469593137	6,853552224	0,000494045	0,001028042
BCL7C	0,670521501	5,611383203	0,000495534	0,001030825
SNRPB2	0,457637428	6,18435042	0,000496807	0,001033159
FOXF2	-0,853195105	4,039129747	0,000498223	0,001035787
MINA	0,475846327	5,965931648	0,000499175	0,001037449
SUZ12	0,530301915	6,278164159	0,00049978	0,001038392
GBAS	0,79297669	7,230530421	0,000500063	0,001038662
ITPRIPL2	-0,565580408	6,824708296	0,000500362	0,001038758
NAGA	-0,398685383	6,469975868	0,000500414	0,001038758
CLDN12	0,59580253	6,07968328	0,000503741	0,001045347
HIST2H2BE	1,184897045	5,513151305	0,000505292	0,001048246
PBX3	-0,676176556	4,860138559	0,000506828	0,001051113
ZNF274	-0,499968416	4,826471529	0,000507133	0,001051426
HERC6	-0,834767956	5,02762253	0,000508033	0,001052971
C12orf65	0,399951513	4,919927915	0,000510065	0,001056699
BHLHE41	-0,951357775	5,073734484	0,000510141	0,001056699
CITED4	1,352952678	4,425429532	0,000510452	0,001057022
IPO13	0,467483657	6,086214297	0,000514625	0,001065235
YBX1	0,560851537	9,907658073	0,000514731	0,001065235

PTPLAD1	0,510605215	7,619766736	0,000516813	0,00106922
UQCC	0,483862266	5,67518678	0,000517947	0,00107124
SKIV2L	0,430250656	6,192648917	0,000518457	0,001071971
TWISTNB	0,485404117	5,479591966	0,00051944	0,001073678
SMN1	0,5033857	2,049184743	0,000522045	0,001078734
METRNL	-0,699181767	5,271949164	0,000523196	0,001080786
LEMD2	0,435859526	6,019044581	0,000523497	0,00108108
TTC14	0,717149902	5,246723344	0,000524546	0,001082919
H19	2,953820636	9,284624338	0,000529263	0,001092326
NOSIP	0,61474864	5,89577667	0,000533958	0,001101682
PANK3	0,572230318	5,398565591	0,000536503	0,001106598
FN1	-1,032598269	11,82620959	0,000544626	0,001123014
SYPL1	0,613518886	8,117706991	0,000546509	0,001126556
ZNF259	0,457035253	6,14367278	0,000547903	0,001129088
RPS4X	0,589685032	10,85907993	0,000548379	0,001129728
HIST1H2BC	1,253019799	2,611955718	0,000556037	0,001145158
ARHGEF5	0,840862425	7,019089803	0,000558598	0,001150085
SNX18	-0,469182128	5,728821004	0,000559307	0,001151197
KLHL5	0,656866624	6,508751367	0,000559756	0,001151774
POLR3E	0,415601628	5,515372814	0,000560831	0,001153637
ITM2C	0,714002058	7,611291809	0,000561184	0,001154014
API5	0,388220567	7,593247575	0,000562272	0,001155904
MSC	1,535097322	4,941579428	0,000566134	0,001163493
LIMA1	0,682011497	7,919186148	0,000571837	0,001174858
CDC42SE2	-0,440173086	6,615720104	0,000578797	0,001188799
PTP4A2	-0,393662759	7,153157877	0,000580859	0,001192676
NT5DC3	0,684238462	4,913521048	0,000583906	0,001198571
PRPF31	0,42305176	6,3078716	0,000584639	0,001199715
ZNF251	0,686064944	4,283145899	0,000585668	0,001201104
ITGB1BP1	0,471079155	6,29833093	0,000585697	0,001201104
RRAGA	-0,394630725	6,643447566	0,000585942	0,001201104
SRP68	0,355255577	7,418576701	0,00058602	0,001201104
FKBP10	1,017656309	7,42691282	0,000587288	0,001203341
C6orf57	0,943871179	3,452137443	0,000587654	0,001203729
PSMC5	0,36794665	7,486388355	0,000589	0,001206123
ORAOV1	1,09684974	4,594970996	0,000590077	0,001207966
MEGF9	-0,785951768	6,720062206	0,000594385	0,001216419
PSEN1	-0,335875323	6,928796108	0,000595501	0,001218338
EIF3H	0,417494438	8,3188637	0,00060247	0,001232227
PSMG4	0,564396567	3,912050009	0,000602783	0,001232496
CUL4B	0,401338601	6,943394805	0,000605911	0,001238521
SRPX2	1,099942518	5,03391992	0,000612342	0,001251292
KLRK1	-0,823529747	2,754899212	0,000615502	0,001257371
CHD1L	0,473762576	6,028698464	0,000617602	0,001261283
COX17	0,663958599	5,487499728	0,000619929	0,001265657
ITGA2	1,047909016	7,416141088	0,000626832	0,001279367
ATXN1	-0,492951755	6,003504186	0,000633886	0,001293379
S100PBP	0,3587956	5,13957164	0,000636098	0,001297503
DAG1	-0,403896413	7,612106775	0,000638209	0,00130142
SRD5A3	0,989913277	6,049062116	0,000639081	0,001302807

DHRS7B	-0,532060461	4,871576116	0,000640665	0,001305415
IL13RA1	-0,478862271	7,572329143	0,000640743	0,001305415
ZNF621	-0,381216638	4,811459039	0,00064136	0,001306283
MAP2K2	0,400173935	7,148270455	0,000643283	0,001309808
MED14	0,404964563	6,529813478	0,000643656	0,001309959
GALNT2	0,631758666	7,904179668	0,00064379	0,001309959
EMB	-0,7779553	5,481107916	0,000643933	0,001309959
RPL27	0,758595214	9,980831509	0,000644799	0,001311329
TAF4	0,48969795	4,872760109	0,000649232	0,00131995
PSMD9	0,416526264	6,096420097	0,000651526	0,001324219
PPP1CB	0,483002946	9,334374218	0,00065469	0,001330253
RHOF	-0,829790495	4,062089677	0,000655187	0,001330868
EIF2S1	0,41164672	7,068426567	0,00065737	0,001334904
DDX54	0,491170228	6,701148659	0,000658618	0,00133704
FAF1	0,404659798	6,250379969	0,000659441	0,001338311
STT3A	0,428952592	7,983984462	0,00066625	0,001351727
CHMP7	-0,366577912	5,765081623	0,000667922	0,001354717
C11orf54	-0,566051641	4,872315173	0,000675478	0,001369636
MARK2	0,447069169	6,722261147	0,000679456	0,001377292
PCBP2	0,330188051	9,921226024	0,000680076	0,001378139
ASB3	0,419327367	4,793900837	0,000684786	0,001387271
NRAS	0,50781503	7,256514192	0,00068601	0,001389338
PAPD7	0,530990848	5,83718423	0,000686845	0,001390616
CRYL1	-0,657928636	5,036103327	0,000688358	0,001393267
PHF1	-0,387105785	5,881671954	0,000691396	0,001399
C9orf3	0,764866385	6,297657477	0,000695102	0,001406082
DBR1	0,434785913	5,22772848	0,00069659	0,001408673
SMCR7L	0,399764506	6,460616077	0,000698825	0,001412775
LUC7L3	0,521167934	6,57965103	0,000703951	0,001422715
POLR1A	0,612134586	5,886976731	0,000705978	0,00142639
PIAS4	0,455684389	4,402076416	0,000711793	0,001437712
UQCRQ	0,698066342	6,682202725	0,000718196	0,001450179
RARS2	0,506762425	5,950963897	0,000718391	0,001450179
AKT2	0,636646646	7,455924512	0,000727395	0,001467921
URB1	0,588188184	6,142667528	0,000734249	0,001481315
RBAK	0,486749085	5,02834358	0,000736532	0,00148548
LOC441454	0,507054494	1,714960863	0,000737643	0,001487283
PIAS2	0,685094638	4,7139505	0,000739935	0,001491462
SUCLG1	0,547518328	6,85999474	0,000747179	0,001505619
BZW1	0,502925819	8,930823662	0,000754078	0,001519071
TERF1	0,398173031	5,649585773	0,000755981	0,001522456
CLP1	0,367335345	4,436418092	0,00076218	0,001534486
CSRP2BP	0,43287185	5,448857297	0,000763046	0,001535776
FAM160A2	-0,385602591	5,122096283	0,000765596	0,001540454
TBC1D4	-0,529298485	5,584874506	0,000767298	0,001543103
HDGFRP3	0,656654377	6,119523201	0,00076743	0,001543103
NUFIP1	0,443288307	4,038666993	0,000767592	0,001543103
WDR61	0,563204297	6,365743956	0,000773283	0,001554087
UBA5	0,362210347	5,983959631	0,000775021	0,001557122
DHX40	0,490625251	6,762964626	0,000778763	0,001564177

SFMBT2	-0,586308875	2,587603782	0,000783563	0,001573356
CAMKK2	-0,323209215	6,453011821	0,000798945	0,00160377
EML3	-0,379210916	5,714666262	0,000802161	0,001609752
FXR2	-0,386425228	5,718428938	0,000802813	0,001610587
ABCC10	0,491726804	5,315650487	0,000804289	0,001613072
ZC3H14	-0,312819112	6,076256504	0,000804532	0,001613085
FAM198B	-0,8141348	5,743160671	0,000804964	0,001613478
UBAP2L	0,389398566	7,962942265	0,000808429	0,001619947
ZNF551	0,566823038	3,520662611	0,000808711	0,001620034
TUBA1A	-0,555683623	8,965270554	0,000809015	0,001620169
CDKN2C	0,778011584	4,501766596	0,000817206	0,001636091
METTL6	0,37131721	4,186379173	0,000826189	0,00165359
NPEPPS	0,349496927	7,336641871	0,000829632	0,001659994
INO80E	0,413181398	5,175947328	0,000843237	0,001686722
FAM122B	0,497931354	5,755564394	0,000848303	0,001696357
DCTN2	0,36045507	7,646392876	0,00084926	0,001697773
TMEM59	-0,468108713	8,397890771	0,000851857	0,001702303
C16orf62	-0,536168713	5,906006394	0,000852025	0,001702303
KIF3A	-0,387948222	4,646986591	0,000853692	0,001705134
MYCBP2	-0,56253467	6,580625918	0,000854099	0,001705447
SSR4	0,701727171	8,384759464	0,000854714	0,001706175
RAD23B	0,384525477	8,182168958	0,000855422	0,001707088
COL5A2	1,232157394	8,582367928	0,000858926	0,001713581
GNPAT	0,360494585	6,398767532	0,000871981	0,001739117
CEP57	0,386552239	5,910528904	0,000877177	0,001748968
ZDHHC4	0,477763557	6,244979036	0,000882827	0,001759719
COMMD8	0,784935502	5,134498214	0,000888522	0,001770552
DHX9	0,313949025	8,289174866	0,000892356	0,001777673
KLHL21	0,597493145	6,551079238	0,000905916	0,001804159
DHCR7	0,893917522	7,55140611	0,000906362	0,00180452
FUBP1	0,393094631	6,711125851	0,000908066	0,001807385
CSDA	0,631231568	8,767340648	0,000913586	0,001817841
PMPCA	0,420116558	6,140924357	0,000916055	0,001822015
CINP	0,521707063	5,186475671	0,000916218	0,001822015
KIAA0020	0,570416649	5,615218358	0,000919169	0,00182735
HNRNPR	0,302960421	7,602190624	0,00091956	0,001827595
TRO	1,08949491	3,948924572	0,000923823	0,001835533
UGCG	-0,548537046	5,042790233	0,000940365	0,001867855
MIA3	-0,343884117	6,597371226	0,000941444	0,001869455
RPRD1B	0,352778208	6,062348461	0,000945235	0,001876436
LASP1	-0,368928564	8,364608861	0,000947838	0,001881056
SUPT6H	-0,416660074	7,394731892	0,000950789	0,001886363
SPOPL	-0,332447655	5,477639408	0,00095436	0,001892896
PIGN	0,570571002	5,746414195	0,000955406	0,001894422
TMX2	0,349541665	7,313434687	0,000955781	0,001894615
CDC25B	0,666834066	7,282395061	0,000956756	0,001895995
ALKBH8	-0,390422717	3,485414249	0,000964546	0,001910877
EIF4A1	0,381292763	9,9954945	0,000967505	0,001916182
LEPREL2	0,95300629	5,491704218	0,000968148	0,001916899
GRIPAP1	-0,339433571	5,829594635	0,00096994	0,001919889

GUSB	-0,405059892	6,767106438	0,000979363	0,001937682
C14orf169	0,405792789	4,348482387	0,000979497	0,001937682
HNRNPA2B1	0,34270238	9,788199417	0,000982451	0,001942962
ZNF511	0,6358278	5,175179686	0,000983527	0,001944525
C6orf106	-0,349284672	7,847356757	0,000984742	0,001946363
TBC1D10B	0,360783693	6,557627297	0,000986586	0,001949443
BICD2	0,788500136	7,090191215	0,000988401	0,001952465
ZNF77	0,593379757	2,916972273	0,000989368	0,001953808
MLLT1	-0,429335143	6,595878808	0,000993912	0,001962214
DCAF12	0,536176416	7,023185044	0,000995431	0,001964643
TRIM25	-0,41902659	6,338874934	0,000998039	0,001969222
NCL	0,438077858	9,827445293	0,00099849	0,001969541
FLT3LG	-0,550193587	2,950003137	0,000999165	0,001970302
RPL13A	0,573731223	11,41134166	0,001000085	0,001971547
PHF23	0,351399927	5,605721232	0,001002014	0,001974778
KAT5	-0,301737654	5,980838546	0,001003384	0,001976906
EPN2	-0,449680153	5,985713792	0,001006918	0,001983297
TTC7B	-0,521846729	4,971948584	0,00100943	0,001987671
AARS2	0,483283381	5,098525272	0,001022435	0,00201244
POLA1	0,545435957	5,193153316	0,001022599	0,00201244
NAF1	-0,313597143	3,567830861	0,001028081	0,002022644
TMEM208	0,542399455	5,642833709	0,001029499	0,00202485
WIPI2	0,328527442	6,858938045	0,00104231	0,002049455
PGP	0,52845487	4,700781323	0,001058184	0,002080069
ANP32B	0,47253781	8,037451193	0,001060572	0,002084162
PTPN2	0,4323106	5,970541246	0,001066301	0,002094816
FUT11	-0,474706122	4,088572227	0,001071556	0,002104534
PIGF	0,555624098	4,945398788	0,001086882	0,002134019
POLR3C	0,363703201	5,404631746	0,001092147	0,002143739
CCDC43	0,405682795	5,194791208	0,001098011	0,002154585
RPS27A	0,538302609	10,27175478	0,001098304	0,002154585
CWF19L1	0,460126221	5,529291776	0,001106955	0,00217093
GOLGA7	0,643278778	6,713648436	0,001107912	0,002172184
PRKAR2B	-0,823662362	4,037277909	0,001112178	0,002179614
NOMO1	0,502674993	7,296562202	0,001112342	0,002179614
LBR	0,54815655	6,791778752	0,00111997	0,002193931
NAB1	0,513985567	6,534624238	0,001120886	0,002195095
EEFSEC	0,428114215	5,204759531	0,001126163	0,002204797
ETFB	0,815654208	6,840237736	0,001127868	0,002207501
CCNI	-0,394805327	8,38633457	0,001129044	0,002209168
GPN3	0,452462916	5,36483806	0,001137609	0,002225289
PIK3CA	0,70438392	5,980431568	0,001142502	0,002233873
TEAD3	0,520015487	6,118597394	0,001142653	0,002233873
FIP1L1	0,456230366	5,976192212	0,001153307	0,002254056
EXOC2	-0,368697211	5,58622414	0,00116342	0,00227317
CPT2	-0,331600801	5,323881898	0,001164708	0,002275034
IMPACT	0,560665756	6,290364421	0,001166893	0,002278649
DPY19L3	-0,452189406	4,245967273	0,001167535	0,00227925
CDK6	1,075660344	6,85130099	0,001175298	0,002293398
UFM1	-0,363172455	6,666523106	0,001175455	0,002293398

RPL6	0,513570728	10,31480898	0,001181547	0,002304624
OAT	0,690198914	7,39615037	0,001184517	0,002309757
EPCAM	0,953080883	8,374154883	0,001199038	0,002337404
ITGB3BP	0,590526965	4,332366898	0,001205143	0,002348633
ZNF800	-0,334934959	4,844053325	0,001206495	0,002350597
CLIP1	-0,410754574	6,899034544	0,001211501	0,002359675
RPL10A	0,553906136	10,01164097	0,001213551	0,002362995
ITPR3	-0,620351371	7,849261176	0,001235704	0,002405442
SLC41A2	-0,638110074	3,828832805	0,001236879	0,002407043
GPSM1	-0,627763469	4,275938713	0,001240583	0,002413562
ZNF445	-0,415605686	4,922837972	0,001243434	0,002418419
SEL1L	-0,413288574	7,630585395	0,001243941	0,002418714
EFNB2	-0,726705748	6,531534272	0,00125435	0,002438258
TINF2	-0,384445727	6,496409083	0,001255701	0,002440189
PLBD1	-0,799100005	6,543827769	0,001258046	0,00244405
SCAMP3	0,379028892	6,775514561	0,001258566	0,002444364
KIAA0100	-0,411014944	8,013419449	0,001259374	0,002445236
CBFB	0,402507051	6,553532817	0,001280736	0,002486006
STAM2	-0,323025156	5,758475325	0,001284497	0,002492596
USP8	-0,292493312	6,285369071	0,001291973	0,002506392
NOL7	0,415769255	5,84670223	0,001296849	0,002515135
CSK	0,399656754	6,951834682	0,001303855	0,002528004
HUS1	0,413242655	4,765270389	0,001305654	0,002530772
DCAF11	-0,330399152	6,774347055	0,001308058	0,00253471
CEBPZ	0,366994776	6,74741762	0,001311309	0,002540289
DHX29	-0,388179751	5,521024858	0,001314615	0,00254597
FAM40A	-0,361460093	5,219474102	0,001332005	0,002578915
ZNF669	0,377640325	3,391543574	0,001332895	0,002579906
EMD	0,362428464	6,410480908	0,001333667	0,002580667
ZNF680	0,691742255	4,160951258	0,001338354	0,002589003
GIPC1	0,613706013	7,637722923	0,001341774	0,002594882
ATHL1	1,025710854	5,38251016	0,001344078	0,002598601
KLC1	-0,352344299	6,669520264	0,001348821	0,002607032
RPL37A	0,547599642	9,732299562	0,001349203	0,002607032
RNF8	0,367775855	5,094642161	0,001349591	0,002607044
RFX3	-0,581338253	2,349502935	0,001351227	0,002609463
NDUFA1	0,536884142	6,740075378	0,001357187	0,002620232
GPR125	0,594637768	5,641655454	0,001359095	0,002623172
FABP5	1,601463029	9,301833789	0,001360384	0,002624918
ZNF543	0,413965941	3,675780137	0,00136507	0,002632502
TRIM14	-0,505517861	6,121777522	0,001365087	0,002632502
CCDC91	0,702871563	5,676589039	0,001370439	0,002642076
PTPRS	0,73998014	7,166187577	0,001376047	0,002651887
ACAT2	0,708818462	6,309106553	0,001376306	0,002651887
AHCYL1	-0,329499714	7,6009629	0,001381561	0,002661261
IL7	-0,721131096	2,259366995	0,00139014	0,002677031
TSC1	-0,371689504	5,387293817	0,001396421	0,002688366
SPPL2B	0,444805077	5,872508906	0,001403032	0,002700331
RRBP1	-0,534926525	8,272649501	0,001408351	0,002709805
LOXL2	1,190791827	6,376090583	0,001412299	0,002716633

DHX33	0,571572286	4,637033802	0,001413612	0,002718393
THRAP3	-0,297191876	7,565663003	0,001422845	0,002735377
ERF	0,528196687	6,209020889	0,001426796	0,002742199
DAPK3	-0,426148799	5,722505953	0,00143225	0,002751906
HSF2	0,558167155	4,77943584	0,001434788	0,002756005
IQCE	-0,481437872	5,06171519	0,001435621	0,002756276
PMPCB	0,390471075	6,505187567	0,001435737	0,002756276
UBE2W	0,523726394	5,96361037	0,001437726	0,002759318
TEX2	-0,413876526	6,382952987	0,001439391	0,002761735
SEC16A	-0,441519706	7,208992844	0,001454055	0,002789087
MBOAT1	0,758445144	5,561605894	0,001458915	0,00279762
BAHCC1	-0,625436531	4,631032076	0,001460234	0,002799363
SYF2	-0,266665088	5,776986277	0,00147068	0,002818596
MKL1	-0,401978174	6,030367094	0,001472384	0,00282107
GPX8	0,708511808	5,493557574	0,001483681	0,002841916
SLAIN2	-0,383209075	6,519225646	0,00149904	0,002870529
RBM10	0,309680506	6,331978817	0,001501557	0,002874541
DHTKD1	0,498986138	5,777901787	0,001512205	0,002894114
AIDA	-0,313763097	7,188793828	0,001513938	0,002896616
TUBGCP4	0,418395582	4,549523063	0,001521465	0,002910202
SELT	0,688999908	8,199901879	0,001522019	0,002910444
NOLC1	0,450136785	7,475300624	0,001528662	0,002922329
FAM54B	-0,357684993	5,702137791	0,001530811	0,002925616
SQLE	0,669337086	7,208480142	0,001531337	0,002925801
MBOAT2	0,805964899	5,748499418	0,001533731	0,002929556
DNASE1L1	-0,449883136	5,468661642	0,001534946	0,002931054
RBM6	-0,416262631	5,844894286	0,001536507	0,002933214
CENPV	1,067058068	3,761381594	0,001540996	0,00294096
KIAA1737	-0,383449108	5,430574122	0,001546186	0,00295004
SPAG7	-0,32970352	5,572383915	0,001560837	0,00297716
OVCA2	0,439570704	3,257494836	0,001561967	0,002978483
TACSTD2	1,233718867	9,382946621	0,001567993	0,002989138
ACBD5	-0,340256751	5,554430812	0,001569506	0,002991185
WDR1	-0,347482381	8,663468041	0,001579782	0,003009929
NDUFS5	0,542521481	7,312643305	0,001580768	0,003010965
GUF1	0,518897079	5,277711947	0,001582185	0,003012823
FAM46C	-0,914107019	6,050331807	0,001598221	0,0030425
SSFA2	-0,535650919	7,888746094	0,001598662	0,0030425
ASXL1	-0,353804885	6,486321643	0,00160853	0,003060425
WDR54	0,6394088	4,9909107	0,001611634	0,003065476
PGLS	0,463615239	6,008930679	0,00162832	0,00309635
PNN	0,471002016	6,789921902	0,00163093	0,00310045
MRPL50	0,524220248	5,129574305	0,001639577	0,003116018
TRPS1	0,851667626	5,307973995	0,001643194	0,003122023
KIAA0391	0,512891355	6,777837776	0,001647703	0,003129527
CS	0,330701154	8,216949317	0,001648061	0,003129527
EEA1	-0,40506278	5,599174407	0,001656947	0,003145523
PPIB	0,450884325	9,215444917	0,001662119	0,003154465
TRIM65	0,418536656	5,437020019	0,001666289	0,003160946
LLPH	0,61212069	5,989913315	0,001666461	0,003160946

NENF	0,611943513	6,542453293	0,001679492	0,003184777
AP2B1	-0,469866787	8,415773698	0,00168202	0,003188684
HSPA1A	0,705341422	8,87235322	0,001687184	0,003197585
PLEKHM2	-0,369641533	6,869255479	0,001692431	0,003206638
HSPH1	0,524360553	7,653778965	0,001693405	0,003207592
AMBRA1	-0,39472945	5,650614034	0,001697088	0,003213675
RPL7A	0,55383456	10,9016764	0,00172	0,003256157
RAB43	-0,444385108	6,643782022	0,001722855	0,003260657
PDIA3	0,41466388	9,817432409	0,001727146	0,003267872
GTF3A	0,601861506	7,311919331	0,00172767	0,003267956
GANAB	0,342306094	9,617332746	0,001737239	0,003285146
ITGA5	-0,702968863	7,807018212	0,00174233	0,003293859
GTPBP1	-0,391502424	6,588238985	0,001745739	0,003299388
HINT1	0,578836701	8,105573353	0,001749249	0,003305106
AFG3L2	0,429781177	6,733186559	0,00175206	0,003308738
WBP4	-0,37452701	4,217403053	0,001752142	0,003308738
TP53INP1	-0,607777603	6,378188155	0,001755113	0,003313431
TUBB2A	0,845911511	5,612499328	0,00175857	0,003319039
UBE2F	0,423922309	5,795081933	0,001763093	0,003326655
SNTB2	-0,425028524	5,955309294	0,001765194	0,003329699
ZNF532	0,571405948	6,815173083	0,00176802	0,003334107
HSPA5	0,396780034	10,16497181	0,001771055	0,003338907
ZNF526	0,371332074	4,555462438	0,001782096	0,003358793
TBC1D23	0,477955672	6,22308592	0,001783313	0,003360159
MANEA	0,474153382	5,289758355	0,001796613	0,003384283
DEXI	-0,317794515	5,189281952	0,001823408	0,003433809
ERMP1	0,778561303	7,045791961	0,001835061	0,003454798
CDC5L	0,351383889	6,270504978	0,001837304	0,003458066
GOSR1	0,424426689	6,887078798	0,001841479	0,003464968
SEMA4C	0,658913189	6,359534097	0,001850619	0,003481206
TBCK	-0,393471009	4,570204949	0,001854233	0,003487042
GLI2	1,418135706	4,521755536	0,001856345	0,003489414
NEK11	-0,615283384	3,295190771	0,001856518	0,003489414
SDHB	0,426764527	6,790959698	0,00186346	0,003501147
ANKRD9	0,682655942	2,763033381	0,001863787	0,003501147
YWHAE	0,390963315	9,727849812	0,001878281	0,003527403
OPN3	-0,71581603	5,095371418	0,00187918	0,003528119
OGFR	-0,425011543	5,755815042	0,00189067	0,003548715
BMP2K	-0,513529257	4,510396194	0,001893952	0,003553895
THUMPD3	0,309317865	5,772049226	0,001895889	0,003556552
C20orf112	-0,579275937	5,028005166	0,001906595	0,003575653
MTMR2	0,40479783	6,367650753	0,001915477	0,003591322
STAM	-0,371770928	5,350786043	0,001917593	0,003594301
MAP3K6	-0,604914879	5,849218811	0,00193025	0,00361703
CDH11	-0,804580018	6,889325086	0,001930971	0,003617387
EIF3K	0,563872878	8,039750818	0,001943152	0,003639207
OSBPL10	-0,466643836	4,733356748	0,001961356	0,003672292
AP1G1	-0,311830918	7,167148822	0,001972244	0,003691444
STARD3NL	-0,575770682	5,771168412	0,001972668	0,003691444
C16orf52	-0,38744752	4,236722892	0,001976438	0,003697484

COX4I1	0,589648752	8,480671183	0,001983687	0,003710028
LSP1	-0,768532338	6,556078594	0,001990973	0,003722634
DBNDD2	-0,542342413	5,347316876	0,001993173	0,003725726
ATG4B	0,372065601	5,969617215	0,002004774	0,003746384
ZNF670	0,567321318	2,63985638	0,00200959	0,003754355
CORO1A	-0,699151241	6,298237034	0,002014835	0,003763122
RBX1	0,599931542	6,561473957	0,002022682	0,003776744
TCF25	-0,33000672	6,815900565	0,002023763	0,003777728
GOLGA8B	0,992836388	5,79401068	0,002027643	0,003783936
ST8SIA4	-0,691561948	5,144646549	0,002029926	0,003787159
TMEM141	0,692297	6,285688767	0,002030912	0,003787962
QRSL1	0,39922762	5,015445342	0,002037125	0,003798511
SRP54	0,382348878	6,531422002	0,002040128	0,003803071
MRPL39	0,49105829	4,848476632	0,002078838	0,00387339
MRPL49	0,491370017	6,95824787	0,002078986	0,00387339
CSAD	0,661544062	4,226355968	0,00208027	0,003874722
HEATR5A	-0,477090056	5,587629986	0,002082281	0,003877411
NDUFS1	0,358431521	6,715238968	0,00208953	0,003889846
IRS2	-0,733062981	5,653319376	0,002100678	0,003909531
MAPK7	-0,399838001	4,576953893	0,002114581	0,003934331
PKP4	0,431798071	7,037954472	0,002119613	0,003942619
TPMT	0,424517633	5,977674516	0,00214562	0,003989905
USP24	-0,38237784	6,272928722	0,002159046	0,004013778
ARL8A	-0,286795489	6,172822301	0,002178125	0,004048143
SLC5A3	0,531003637	5,803786924	0,002179007	0,004048678
ZBTB11	0,359290344	5,708571865	0,00219404	0,004075303
PSMB6	0,385456756	7,088270415	0,002194532	0,004075303
PRR13	-0,320090438	7,32399229	0,002205002	0,004093631
AARS	0,470831235	8,01222252	0,002211936	0,004105388
RNF149	-0,375256294	6,640467729	0,002218257	0,004115999
ODC1	1,152232738	9,113050936	0,002221688	0,004121244
LOC650623	-0,571095654	2,636791407	0,002223346	0,004123198
EDIL3	-0,871736827	5,230287706	0,002226078	0,004127141
BTF3	0,413713986	9,062921735	0,00223206	0,004137108
ARHGEF1	-0,393594518	6,787577783	0,002241817	0,004154063
MXRA5	1,136702246	8,074586806	0,002243475	0,004156006
XIAP	-0,258985043	6,607382934	0,002246362	0,004160223
TPD52L2	0,419455057	7,691473986	0,002254792	0,004174703
WIZ	0,500236445	6,281115036	0,00226142	0,004185837
RBM22	-0,267548458	5,896357209	0,002276856	0,004213265
FBXL4	0,36757825	4,539092661	0,002295914	0,004247379
ATP5L	0,527290151	7,807106113	0,002304448	0,00426201
FKBP11	0,795117223	5,957949461	0,002313649	0,004277867
BRAF	0,516781667	4,099267638	0,002318468	0,004285614
NFYA	0,389311683	5,880332178	0,002325889	0,004298167
RTN4	-0,319977615	8,665630264	0,002330051	0,004304691
UQCR10	0,484300259	6,722354394	0,002350538	0,004341364
F8A1	0,471847494	4,441151657	0,002355498	0,004349347
FBXO8	-0,396190577	4,702646196	0,002363893	0,004363668
UTP23	0,334189664	5,254179611	0,002370829	0,004375287

GABPB2	0,584467351	3,164725211	0,002375984	0,004383614
EIF1AX	0,535511715	7,861537132	0,002384327	0,004397817
NAV1	1,014955612	7,116757835	0,002388078	0,004403543
UBA52	0,434368917	9,452558861	0,002408247	0,004439535
ATP1A1	-0,442996282	10,33653136	0,002425639	0,004470386
RRP1B	0,443450977	6,149292582	0,002427957	0,00447345
NFYB	-0,310405465	5,502479714	0,002440206	0,004494805
KCTD20	-0,33645232	6,890309242	0,002452887	0,004516942
USP37	0,386883221	5,087748245	0,002458688	0,004526402
ZFAND2A	0,668621485	4,760963567	0,002460898	0,004529247
NARG2	0,365061665	6,023582911	0,002463989	0,004533714
BCLAF1	-0,274380285	7,504054258	0,002475158	0,004553035
ATP5I	0,595714905	6,371206146	0,002479668	0,004560101
ITM2B	-0,473613827	9,928360616	0,002492773	0,004582965
MEX3C	0,381453723	6,092434297	0,002507539	0,004608871
C10orf137	0,392685896	4,409679775	0,002516747	0,004624548
OXSR1	-0,330500373	6,155287564	0,002530131	0,004647736
EXOC3	0,42544013	6,685947719	0,002530729	0,004647736
SERP1	0,492973505	8,776431815	0,002557927	0,004696419
MFSD6	-0,487093027	6,612147311	0,002575078	0,004726637
ZFAND1	0,483727077	5,719723035	0,002577072	0,004728285
FBXO21	-0,338896005	6,314178483	0,002577362	0,004728285
TRIP11	-0,377174059	5,443592099	0,002579914	0,004731693
TGFB2	-0,967966271	4,845273534	0,002580968	0,004732352
OS9	-0,32010387	8,894554146	0,002589404	0,004746544
BMPR1A	-0,366196067	5,480846316	0,002592607	0,004751139
TDP2	0,604508916	6,470626248	0,002608806	0,004779541
DAXX	0,303763204	6,632927397	0,002617223	0,004793673
TMLHE	0,515723401	4,246021815	0,00262498	0,004806589
NOP14	0,372869666	6,245747531	0,002631039	0,00481639
KLHL15	-0,475466653	4,290679917	0,002642086	0,004834242
NR2F6	0,567786576	5,629209127	0,002642209	0,004834242
EGFR	1,364034713	7,710236756	0,002662172	0,00486946
FAM118A	0,457101695	4,775036975	0,002664109	0,004871698
PASK	0,522700383	4,133779444	0,002683661	0,004905711
TMEM165	0,758160066	7,526146757	0,002684148	0,004905711
PCID2	0,609372806	5,910831713	0,002703742	0,004940197
C5orf43	0,338093183	6,239711299	0,002708325	0,004947245
CDK14	-0,644249441	5,210806225	0,002714196	0,004956643
SLC44A3	-0,69708473	3,98342418	0,002717615	0,004961557
P2RX4	-0,432754124	5,401424186	0,002726146	0,00497571
ESRRA	0,498760758	6,099791565	0,002726826	0,00497571
CRTC1	-0,53501076	4,200558848	0,002739713	0,004997888
ANKIB1	0,459016814	6,576230253	0,002762364	0,00503786
NDUFS2	0,442102709	7,17527552	0,002772065	0,005054201
CXorf56	0,422158751	4,270488055	0,002785741	0,005076599
HNRNPK	0,199764891	9,729179227	0,002785839	0,005076599
CXCL9	1,353370122	6,994836446	0,00278813	0,005079417
LONP1	0,467329746	6,942742092	0,002792773	0,005086517
GALNS	0,53671109	5,622938555	0,002803303	0,005104332

IFNGR1	-0,569361644	7,819737725	0,002830641	0,005152734
HAGH	-0,457942384	5,211558081	0,002839029	0,005166624
UBE2K	0,378254491	7,049901804	0,002865065	0,005212614
PHF3	-0,407561451	6,590993596	0,002885132	0,005247363
TNC	1,435098424	9,464509954	0,002885703	0,005247363
RCN3	1,051956649	5,651671073	0,002889258	0,005252426
MRPL46	0,433642026	4,678869301	0,002896858	0,005264839
APPL2	0,446501509	6,082289788	0,002898156	0,005265794
COPZ1	0,32021004	8,078592552	0,00289936	0,005266285
TBC1D3	0,446119369	4,371093103	0,002899971	0,005266285
PGGT1B	-0,307246807	4,474815405	0,002906185	0,005276165
SUPT7L	0,247828271	6,098997644	0,002919927	0,005299702
RSL24D1	0,602001682	7,427507656	0,002922681	0,00530329
ACTB	-0,358598996	12,85453661	0,002937216	0,005328245
PLEKHA5	0,521915281	6,084399517	0,002938199	0,005328611
SPEN	-0,463870829	6,785089755	0,002950364	0,00534925
ZBTB2	-0,257349811	4,863443471	0,002951313	0,00534955
STX16	0,368130237	6,654646757	0,002952825	0,005350866
COMMD1	0,556917197	5,191232269	0,002961929	0,005365939
TTL	0,343359135	6,369590558	0,002970705	0,005380409
UBB	-0,439370179	10,00499886	0,002985042	0,005404938
YTHDF1	0,315444485	6,627509726	0,002988061	0,005408969
MTF2	0,364585674	5,038410801	0,002998338	0,005424864
FOSL2	-0,512238498	7,685341965	0,002998433	0,005424864
OPTN	-0,443020453	6,81596404	0,003004745	0,005434843
MGAT2	0,423113421	6,27806785	0,003006268	0,005436156
PIK3CD	-0,579105535	5,169791021	0,003046971	0,00550751
SETD6	0,422087653	4,239436658	0,003047343	0,00550751
NRD1	0,310227753	7,728760936	0,003051394	0,005513369
MED8	0,376620826	5,811907666	0,003053785	0,005516228
TMEM60	0,419612607	4,449927975	0,003078472	0,005559348
RPL23AP82	0,360040536	4,560029781	0,003098866	0,005594696
PDCD11	0,47395146	6,311546702	0,003108519	0,005610637
USP16	-0,32075194	5,854771556	0,003129242	0,005646548
SMARCC1	0,418979917	7,52267515	0,003142321	0,005668405
LYPLAL1	0,67016831	4,938870946	0,003143018	0,005668405
KIAA0664	0,495249827	6,812234584	0,003156282	0,005690821
NBEA	-0,817912051	3,54542718	0,003161634	0,005698964
KIAA1009	-0,453825925	3,344925447	0,003164255	0,005702182
GPNMB	1,143621015	10,67801252	0,003170117	0,005711235
GNA15	0,813246903	6,166153745	0,003184491	0,005735616
ORMDL3	-0,459865394	6,63812122	0,003193169	0,005749728
TCEA3	-0,592745686	5,162526566	0,003207048	0,005773195
WARS	-0,645495392	8,73523007	0,003226789	0,005807198
FAM126B	-0,357058106	4,925831258	0,003249671	0,005846835
OGFOD1	0,278393539	6,019155744	0,003272629	0,005886589
SOLH	0,485321032	5,73557168	0,003278118	0,005894907
GRAMD1A	-0,418648251	6,501017596	0,003280245	0,005897177
MAGEH1	-0,534835709	4,991003666	0,003282235	0,0058992
ADCK2	0,458449598	5,870376383	0,003290404	0,005912323

ENDOG	0,488249651	3,646433007	0,00329681	0,005922273
ENAH	0,474593703	7,929872062	0,003307048	0,0059391
GTF3C6	0,405099263	5,742077806	0,003344531	0,006004834
DDX31	0,369586697	4,35388496	0,003347694	0,00600893
UQCR11	0,540530926	6,763022406	0,003353921	0,006018522
EIF1B	-0,375191281	5,622037183	0,003366116	0,006038818
OSBPL1A	-0,463221344	5,878936173	0,003378323	0,006059123
MS4A6A	-0,735679785	6,199739339	0,003391635	0,006081399
TRERF1	0,560538093	4,736209432	0,003400843	0,006096307
LDHB	0,682910804	9,37057117	0,003407824	0,006107214
GFPT2	-0,76028491	4,646116326	0,003414595	0,006117742
MRFAP1	-0,308893656	8,361264261	0,003422857	0,006130933
CNN3	-0,650875847	8,095363874	0,003426462	0,006135779
MYBBP1A	0,498339004	6,255610226	0,003432511	0,006144998
USP32	-0,327725847	6,201906378	0,003447724	0,006170612
NR1H2	-0,337296322	6,779504997	0,003450449	0,006173868
BCAS2	0,411300362	5,502022553	0,003465257	0,006198738
YIPF6	0,45738395	5,020880093	0,003482326	0,006227637
CLK4	-0,460906864	3,825981735	0,003485001	0,006230787
KLHL20	-0,325115461	4,875868116	0,00349429	0,006245757
SLC35C2	0,320830614	6,367561643	0,003511957	0,006275691
WIBG	0,380738927	5,38506943	0,003539003	0,006322364
LSM10	0,462853173	5,283906507	0,003551244	0,00634257
C16orf5	-0,550094741	5,272819005	0,003571294	0,006376709
STAU2	0,399508715	6,072000692	0,003572257	0,006376758
ZNF136	-0,366934939	3,882308803	0,003574403	0,00637892
RPL7	0,457940195	10,69921072	0,003601029	0,006424757
ELP2	0,438832177	6,431794454	0,003632428	0,006479081
HDDC2	0,703166669	6,400176813	0,003639715	0,006489353
STXBP3	-0,286292096	5,575346219	0,00364009	0,006489353
ZC3H7B	-0,405316628	7,317151473	0,003653712	0,006511936
RPL11	-0,392879736	9,668602245	0,003664539	0,006529526
FOXK1	0,574162079	6,914688738	0,003665886	0,006530219
EXOSC7	0,371107355	5,456554967	0,003681567	0,00655644
TMEM209	0,370310803	5,626834986	0,003706791	0,006599637
USP13	-0,545459979	5,479624209	0,003712684	0,006608402
LRCH4	-0,388809941	5,644816803	0,003729219	0,006636102
CTR9	-0,357261476	6,217433739	0,00373192	0,006639176
MIB1	0,468324679	6,818866765	0,003738781	0,006649647
KITLG	-0,600188152	6,667202596	0,003740013	0,006650105
IRF2BP1	0,493284073	5,184305048	0,003752504	0,006670574
WDR59	0,373259803	5,530551924	0,00377387	0,006706807
CBWD1	0,390891319	5,014997587	0,003783846	0,006722784
SYNCRIP	0,319052095	8,189879049	0,00379185	0,00673525
UXT	0,494034379	6,307753762	0,003829647	0,006800615
RPL4	0,426629849	11,08957454	0,003831747	0,006802572
VPS35	0,317988709	7,610138907	0,003838265	0,006811408
DEDD	0,376869938	6,101837524	0,003838721	0,006811408
VAPB	0,306646612	6,266837643	0,003843124	0,006817447
GLTSCR2	-0,42079006	7,864833562	0,003847912	0,006824164

NPL	0,773217472	6,146704769	0,003851162	0,006828153
CEP170	-0,414368934	5,866229833	0,003859357	0,006840903
MRPS6	0,486385717	5,809483489	0,003861406	0,006842757
PIGK	-0,360666845	5,498707408	0,003879396	0,006872851
UBE3C	0,3576603	7,404675723	0,003893247	0,006895598
SLC38A10	-0,378090329	7,226746729	0,003899131	0,006904225
SDHAF2	0,326428319	5,739868213	0,003903825	0,006910744
SHC1	0,533590424	8,112896562	0,003908665	0,006917515
XPO6	0,461938737	7,356694189	0,003910445	0,006918869
PRKD3	-0,348388134	6,272716024	0,003913297	0,00692212
CDKAL1	0,951070844	5,709395131	0,003917443	0,006925903
HMOX1	-0,793871023	6,769593821	0,003917467	0,006925903
LOC285359	0,441385651	1,374064991	0,003940514	0,006964843
COX10	0,333787808	4,487883881	0,003942282	0,006966163
NUP210	0,795600427	7,039000264	0,003956539	0,006989544
RAC2	-0,649235745	6,66217751	0,004009748	0,007081706
C1orf85	0,410097823	6,489854062	0,004020351	0,007098595
NDFIP2	0,570264602	6,571923815	0,00406942	0,007183374
UBP1	-0,29976563	6,669472873	0,004070848	0,007184036
ASNA1	0,502044626	6,847113212	0,004087619	0,007211765
IVNS1ABP	0,490509408	8,062541317	0,004108115	0,007246052
PDIA3P	0,36097543	5,7770956	0,004116682	0,007259286
HIF1A	0,530347746	8,755680584	0,00417994	0,007368926
NLRP1	-0,716609107	4,927279304	0,004181705	0,007370134
COX16	0,423302997	5,933472645	0,004188058	0,007379423
FAM118B	-0,392681539	4,858992875	0,004225288	0,0074431
ASL	-0,491582835	5,625185734	0,004256999	0,007497023
PRKCSH	0,362791891	8,552254253	0,004288552	0,007550642
HECTD1	-0,42589648	7,415723967	0,004299779	0,007568454
ARID5B	-0,467445794	6,756694396	0,004303056	0,007572267
CPSF3L	0,338153553	6,552813439	0,004326446	0,007611464
FAM89B	0,436437629	6,150807731	0,004341753	0,007636424
FAM133B	0,410474243	5,151162359	0,004354538	0,007656935
WDR13	-0,322883717	6,358726263	0,004372382	0,00768633
HELB	0,544840438	1,671416273	0,004384909	0,007706364
YLPM1	-0,403478556	6,619140707	0,00439074	0,007714001
ZCCHC14	-0,402958246	5,700947564	0,004391517	0,007714001
NUDT21	0,332113127	7,274899148	0,004408531	0,007741893
SIX5	0,474321423	4,791069789	0,004412685	0,007747193
RNF138	0,53173316	5,74783041	0,004425665	0,007767982
CCNDBP1	-0,379691629	6,037327007	0,004452747	0,007813505
ZNF662	-0,584440755	2,820352058	0,004477142	0,007854291
SLC35B3	0,316048878	5,112775651	0,004481465	0,007859854
SQRDL	-0,542334517	6,913578376	0,00448297	0,007860471
ERCC2	0,431370486	4,978822577	0,004498542	0,007885748
HNRNPF	0,32939565	8,407092662	0,004545541	0,007966087
GSPT1	-0,267244179	7,851969466	0,004554887	0,007980415
MLL4	0,475132505	6,406409718	0,004576612	0,008016418
YPEL3	-0,46373852	5,400603737	0,004649396	0,008141815
GPC4	-0,747504125	6,630440983	0,004654768	0,00814913

GBA2	-0,448675985	5,752997743	0,004663016	0,008161475
CDC23	0,294021593	5,651048161	0,004686324	0,008200166
NOL8	0,375838755	5,468303775	0,00471245	0,008243765
NOP10	0,512037718	6,788811017	0,004718955	0,008253028
DNAJC15	-0,629263173	5,205169089	0,004723932	0,008259614
MTERFD2	-0,313675853	4,286153984	0,004741468	0,00828815
VEGFB	-0,330334116	6,595109431	0,004753325	0,008306747
SGTA	0,328693781	6,684506986	0,00475684	0,008310758
FDX1L	0,428531349	3,963604558	0,004759282	0,008312896
FBLIM1	0,547122213	6,861072279	0,004761013	0,00831379
RABGAP1	-0,351701646	6,362182094	0,004811199	0,008399275
WSB2	0,371633983	7,487852016	0,004838627	0,008444995
LTV1	0,352721834	5,498836684	0,004858333	0,008477219
USMG5	0,564769113	5,864426753	0,004860125	0,008478177
ARFGAP1	0,389786778	6,41324316	0,004864472	0,008483092
POLM	0,43905064	4,555629509	0,004865431	0,008483092
INTS4	0,363448373	5,226324338	0,004878609	0,00850284
APOC1	-0,895805772	7,116437495	0,004879753	0,00850284
PCDHB14	0,843891518	3,624885161	0,004880498	0,00850284
MAP2K4	-0,326915051	5,6330575	0,004882463	0,00850409
LOC440944	0,531208009	3,796220525	0,004888703	0,008512785
SCRN3	0,372464237	4,737123798	0,004895281	0,008522064
MIER1	-0,370332179	6,01574036	0,004899009	0,008526377
FAM135A	0,501809039	5,499349718	0,004919644	0,008560105
DMTF1	0,362253547	5,652975985	0,004927436	0,008571476
MAP1LC3B2	-0,355029851	3,141786053	0,004931534	0,008576417
CEP70	0,53734505	5,309697536	0,004938033	0,00858553
KIF1B	-0,353460894	6,626281266	0,004939647	0,008586147
ESF1	0,461286496	4,953726313	0,004942174	0,00858835
FAM45A	0,361106618	5,508810755	0,004971457	0,008637036
MED17	0,307185252	5,370652206	0,004994284	0,008672423
SCPEP1	0,626737154	8,50356566	0,004994369	0,008672423
DPCD	0,501542137	4,363336894	0,005000431	0,008680738
CBLB	0,5452317	5,505317762	0,005033526	0,008735967
SGSM2	-0,484829455	5,44110742	0,005038588	0,008741865
CCDC82	-0,345878966	4,789916112	0,005039489	0,008741865
HSPA9	0,337874281	8,431993599	0,005043119	0,008745939
CWC15	0,3363167	6,019161808	0,00507014	0,008790563
RPUSD3	0,382285024	4,919972163	0,005090761	0,008824073
EXOG	-0,301407483	3,106275445	0,005111641	0,008858013
AGPAT3	-0,404263639	6,824466411	0,005119627	0,008869598
COL3A1	1,130251126	12,12517351	0,005153753	0,008926452
TULP3	0,459358145	5,627461665	0,005167536	0,008948052
INPPL1	-0,426551536	6,707061972	0,00516908	0,008948453
IER5	-0,579983957	5,953726764	0,005206011	0,009010099
DLD	0,363885027	6,970450289	0,005222269	0,009035944
HMG20A	-0,26485808	5,668862402	0,005226225	0,009039353
ATP8B1	0,596257456	6,796820196	0,005226891	0,009039353
PCBP4	-0,429702809	4,66891763	0,005252663	0,009081621
CCDC90A	0,40111385	4,96439378	0,005262716	0,009096695

SRBD1	-0,306703442	4,692032704	0,005267298	0,009102308
E2F5	0,784751589	3,829947914	0,005279559	0,009121184
POMP	0,436106163	7,058659073	0,005292253	0,009140799
QTRTD1	0,355954706	5,614684817	0,005314433	0,009176784
HERC5	-0,685782355	4,220828019	0,005332102	0,009204963
ARHGAP27	-0,437668412	5,666376128	0,005334405	0,009206607
FBXL18	0,475883406	5,27010335	0,005355348	0,009240415
SETDB1	0,333816018	6,00910195	0,005363041	0,009251348
LRP6	-0,495372311	5,797065425	0,005383467	0,009282225
PSD3	0,667921468	5,814244127	0,005383663	0,009282225
RPL27A	0,538979318	10,46854779	0,005459013	0,009409759
RPL13	0,524142763	10,51448355	0,005491811	0,009463901
PEX10	0,346097474	5,026904505	0,005570385	0,009596881
EIF4E	0,352967634	6,782246441	0,005589726	0,009627771
TM9SF4	0,399371673	7,469856159	0,005596403	0,009636836
MBOAT7	0,434775139	6,540311083	0,005608062	0,009654475
NDST2	-0,311118112	5,142770764	0,005625372	0,009681831
UBE2D4	-0,365747458	3,877796857	0,005639366	0,009703466
EIF2C3	0,375897316	3,550980922	0,005644376	0,009709636
GALNT6	0,819416207	5,85687163	0,00570371	0,009809231
RAB34	0,448481775	6,768800071	0,005717545	0,009830544
TDRD7	-0,466486148	5,068805076	0,005743089	0,009871975
HK2	0,781188984	7,984907682	0,005753283	0,009887006
LANCL2	0,646276675	5,367568737	0,00576651	0,00990724
PSENEN	0,546915306	6,266362017	0,005797648	0,009958228
SMARCA2	-0,676512608	7,214275112	0,005823341	0,009999839
PGRMC2	-0,279615913	6,227300665	0,005839026	0,01002425
SAP30	0,417426443	3,384650866	0,005922448	0,010164405
LRIG1	-0,548811607	6,94321235	0,005923646	0,010164405
CGGBP1	-0,387929668	6,755704776	0,005941175	0,010191918
VPS41	-0,288277024	6,374466673	0,006010464	0,010308187
ZNF672	-0,319349571	5,677345632	0,006030485	0,010339923
TMCO7	-0,289314406	4,553331334	0,006059756	0,0103875
ZXDB	-0,346864218	4,378804101	0,006065922	0,010395457
TSPAN5	0,743362725	3,619683745	0,006073955	0,010406607
PIP4K2C	0,317495399	6,709397308	0,00611942	0,01048187
CHPF2	0,406583415	6,704210278	0,006169953	0,010565773
TATDN2	-0,290596655	6,160666314	0,006206068	0,010624951
TAOK1	-0,404649429	4,796861332	0,006221248	0,010647392
TPP2	-0,319472999	5,726608939	0,006222299	0,010647392
LUC7L2	0,26299679	7,013845576	0,006241729	0,010677961
ZBTB1	-0,269407997	5,576346148	0,006244557	0,010680118
TLE4	-0,568727752	5,182481858	0,006255635	0,010696383
FAM178A	-0,305420766	5,267859217	0,006265225	0,010710095
HNRNPA3	0,341019479	9,168559635	0,006283312	0,010738322
GNAI3	0,310240026	7,409675736	0,006307212	0,010776467
AGPAT6	-0,452697946	6,416741508	0,006346485	0,010840851
CABIN1	-0,402746763	6,621164885	0,006372803	0,010883081
DYNC1LI1	-0,273349295	5,725318423	0,006398275	0,010923845
NFE2L2	0,68988724	8,758849936	0,006403521	0,010930065

ASPH	0,806212855	8,929548136	0,006418267	0,010952493
NGDN	0,348225194	5,394914237	0,0064246	0,010960557
PHTF1	-0,38157656	4,371977099	0,00643683	0,010978674
AIFM1	0,305378225	5,791794824	0,006452738	0,011003056
AMN1	0,550296934	3,952549688	0,006549568	0,011165376
ATXN3	-0,254069683	4,951609528	0,006567812	0,011193678
ATP6V0E1	-0,338393039	7,957378733	0,006576451	0,011205602
CORO1C	-0,373816481	8,398180563	0,006587189	0,011221095
NOL6	0,55120893	6,773402127	0,006603197	0,011245554
RPS26	0,599992241	8,663979373	0,006632518	0,011292669
WLS	-0,602510139	6,796763606	0,006643757	0,011308982
HIBADH	0,386134863	6,048433828	0,006663775	0,011340225
C9orf64	-0,343069986	4,466030073	0,006699931	0,01139891
PEF1	-0,28592743	6,865699841	0,006717722	0,011426327
PDHA1	0,355958623	6,437458965	0,006726228	0,011437942
DYNLT1	0,547534585	6,971500421	0,006754182	0,011482614
PDZD8	-0,416411707	5,117753507	0,006760625	0,011490703
TMEM126A	0,416377193	4,575736132	0,006765791	0,011496619
PPCDC	0,455970765	3,903004637	0,00677301	0,011506018
EHBP1L1	-0,44139226	6,498733528	0,006794542	0,011539721
CNOT2	0,294254339	6,513901912	0,006808233	0,011560094
RPS3A	0,742316684	11,55157886	0,006812127	0,011563827
DCLRE1C	0,333143546	4,309163076	0,006821009	0,011576022
CLPTM1L	0,440775875	8,067204114	0,006824034	0,011578275
PPP2R5E	0,312839287	5,908079445	0,006849368	0,011618369
ITGB1	-0,404803505	9,881046654	0,00685226	0,011620384
ZNF154	-0,695335027	1,374016043	0,006888868	0,01167956
MAEA	0,405339405	6,15424195	0,006931091	0,011748225
TOP1	-0,336343347	7,454553449	0,006937904	0,011756851
TSPYL4	-0,45420449	5,110192336	0,006969436	0,011807351
RELA	-0,234903104	7,149953481	0,006972021	0,011808795
BET1L	-0,283164508	6,659806488	0,006987531	0,011832126
DHX38	-0,319216137	6,288399857	0,006998662	0,011848033
SCYL2	0,278867317	6,42629105	0,007001667	0,011848407
ZDHHC5	0,312163534	7,778132236	0,007002358	0,011848407
ACACA	-0,401512199	6,964482557	0,007031734	0,011895161
MAN2A2	-0,419000998	6,316515063	0,00704801	0,011919737
PSPC1	0,355063654	5,797875152	0,007087296	0,011983206
DDX17	-0,266804546	8,883724227	0,007102318	0,012005629
TNPO2	0,427426081	6,936344248	0,007214621	0,012192442
ABHD4	0,661385237	6,565145756	0,007223238	0,01220398
FAM120B	-0,29361853	5,466876307	0,007233453	0,012217121
SART3	0,276343035	6,446293672	0,0072346	0,012217121
PIGT	0,407582978	7,918802464	0,007250776	0,012241232
VPS45	0,291169083	5,684939257	0,007252468	0,012241232
RFNG	0,400985172	5,443320214	0,007279725	0,012284198
KIAA0922	-0,420447656	4,951301091	0,007284914	0,012289912
MYO7A	0,724583033	3,862701597	0,007316916	0,012340848
NFKBIL1	0,39623804	4,590094048	0,007324194	0,012350068
PRDX6	0,404401991	8,696258075	0,00733756	0,012369549

FAM192A	0,287948898	6,532838085	0,007378508	0,012435504
RNF11	-0,370757772	7,29027299	0,007384063	0,01244179
ST6GAL1	-0,698942181	7,15578621	0,007390625	0,012449771
RPL32P3	0,587176835	3,245097684	0,007434369	0,012520366
PDE7A	0,562651347	5,687715145	0,007469411	0,012576275
CIR1	-0,263128619	5,667864305	0,007482319	0,012594898
CD320	0,584103723	5,551110192	0,007488973	0,012602988
LYN	-0,544394278	6,975781881	0,007495713	0,012611218
PREP	0,562637499	6,564659508	0,007505066	0,012623839
TOP2B	-0,347851922	7,368410587	0,007539012	0,01267781
TLE3	0,349997828	6,783916096	0,007599138	0,012774712
EIF4EBP3	-0,5428911	2,665932793	0,007600382	0,012774712
HIST1H2AC	0,799299783	5,778977399	0,007613116	0,012790344
CYP51A1	-0,511129934	7,535156205	0,007613434	0,012790344
RPF1	0,356307057	5,540190453	0,007623383	0,012802016
EBPL	0,523481879	5,459477958	0,007624137	0,012802016
TEP1	-0,394784468	5,516406126	0,007629554	0,01280796
TRAF2	0,44798053	4,857764102	0,007656692	0,012848966
MBD6	0,388344043	6,523733621	0,00765775	0,012848966
ZNF654	-0,407775565	4,244681061	0,007674897	0,012870252
IER3IP1	0,464397923	6,506057637	0,007674995	0,012870252
SBF2	-0,348149971	5,723143059	0,007676098	0,012870252
WDR34	0,519120905	6,158903103	0,007678074	0,0128704
TM7SF3	0,504964914	7,638871077	0,007687044	0,01288227
ARHGEF11	-0,328408905	6,429848273	0,007702835	0,012905561
RTEL1	0,544712839	4,793908941	0,007714355	0,012921687
ACOT9	-0,372184757	5,493707995	0,007728707	0,012942548
C17orf59	-0,316890913	3,51762867	0,007794493	0,013049509
C10orf12	0,469198329	3,127690167	0,007823911	0,013095545
GLUL	-0,487632689	10,25263965	0,007837842	0,013115643
CCNT2	0,30912492	5,546903841	0,00789002	0,013199718
TTC17	-0,256781971	6,211134278	0,007912817	0,013234146
INTS2	0,340038787	4,822955361	0,007914581	0,013234146
DUSP23	0,559309187	5,538301347	0,007916422	0,013234146
SUPT4H1	0,357301887	6,684117536	0,007920645	0,01323796
KRAS	0,503709387	6,791252389	0,007945808	0,013276761
ADAMTS12	1,077228575	4,40202995	0,007986962	0,013342257
ASF1A	0,489467398	5,571710395	0,008022199	0,013397839
TSNAX	0,402539705	6,169286763	0,008031482	0,013410057
MRS2	0,43696514	5,745424506	0,008096707	0,013515653
LDB1	0,36867392	6,560789546	0,008127373	0,013563523
TNFRSF14	-0,519745453	5,804559584	0,008142117	0,01358203
RNGTT	0,313768604	5,168836677	0,008142446	0,01358203
DNAJB14	-0,302399787	4,604024864	0,00816528	0,013616787
PLAGL2	0,482630887	6,530689781	0,008175819	0,013631029
NSL1	0,453050784	5,706214328	0,008181217	0,013636694
B9D1	0,62986634	3,883590375	0,008218894	0,013696148
SCAP	-0,270079266	6,630932222	0,008252558	0,013748886
ASH1L	-0,375977365	6,572889876	0,008263852	0,01376434
PDIK1L	0,375691881	4,30324687	0,008289415	0,013803546

PBX2	-0,329249439	6,655948032	0,008306243	0,013825213
ETFDH	-0,33313622	4,255287948	0,008306482	0,013825213
CAPG	-0,489686114	8,153490518	0,008315642	0,013837083
PYGL	0,841702856	6,987020224	0,008332282	0,013861388
ZNF740	0,305163213	5,671890436	0,008340262	0,01387128
PLXNA1	0,590543398	7,679261559	0,008397604	0,013963243
SRRM2	-0,432495566	8,984971014	0,008404582	0,013971439
ZCCHC17	0,387682708	5,81485612	0,008423463	0,013999413
IGF1R	0,67515598	7,434458099	0,008426686	0,014001358
RPA2	0,410195631	6,217859471	0,008476363	0,014080468
USP42	0,329582426	4,78187948	0,008490574	0,014100639
MCCC2	0,332906538	6,562634406	0,008533102	0,014167817
SDHD	0,386330725	7,469688636	0,008556872	0,014203824
LONRF1	-0,451048726	5,004646681	0,00857534	0,014231016
MINPP1	0,461875955	5,105404792	0,008601643	0,014269257
MPPE1	-0,317574624	5,773475243	0,008602569	0,014269257
KRT8	0,649438034	10,11921917	0,008668081	0,014374427
MRPL1	0,470439029	4,670917115	0,008700973	0,014425465
ADCY3	0,429897998	6,732158574	0,008705771	0,01442991
NARS	0,341416327	7,727800569	0,008715406	0,014442371
UBR3	-0,293530007	6,001417838	0,008726217	0,014456772
ORAI2	-0,371422437	6,076435907	0,008792865	0,01456365
NLGN2	0,51869338	5,257856485	0,008817084	0,014600217
TMEM120B	-0,395207494	5,454357055	0,008843579	0,014640535
STYX	0,312876738	5,468577017	0,008873573	0,014686624
SLC38A2	0,462819875	8,867363024	0,008893062	0,014715309
RHBDD2	-0,350236056	7,414710584	0,008897167	0,014718531
FAT1	0,748869773	8,009532054	0,008922378	0,014756657
ANKRD50	-0,480656001	5,919419737	0,008932693	0,014770134
IMPA1	0,408973055	5,279836968	0,008982465	0,014848831
WIPI1	0,532053256	6,072775097	0,008987356	0,014853316
DYNC1H1	-0,407435428	9,068691062	0,008997255	0,014866073
UHRF2	0,452756792	5,477257213	0,009015325	0,014892321
SREBF1	0,478254716	7,428192389	0,009056535	0,014956772
PPP2R1B	0,357241397	5,938574563	0,009095589	0,015017633
UBE2J1	-0,333476931	6,940961619	0,0091209	0,015055778
ATP5F1	0,388579556	7,795686889	0,009164303	0,015123762
BTBD3	-0,425546669	5,719164806	0,009182081	0,015149433
PANK2	0,296521972	5,243756224	0,009188058	0,015155628
GLT25D1	0,403008259	8,097617355	0,009240245	0,015238025
HDAC6	-0,330285643	5,810791017	0,009264577	0,015271514
POLE4	0,515101593	5,829660761	0,009265431	0,015271514
ELK4	0,370638191	3,852060315	0,009267272	0,015271514
DCAF16	0,403062653	5,635758928	0,009343458	0,015393341
PXN	-0,458258631	7,426397387	0,009368652	0,01543112
SYNE2	-0,468049119	7,352116826	0,009378111	0,01544297
PPP1R7	-0,251441723	6,031094288	0,009434922	0,01553277
TBC1D17	-0,314300249	5,719970405	0,009469824	0,015586467
PLS3	-0,402054662	8,847021297	0,009530436	0,015682442
GLA	0,552233768	5,878864386	0,009533019	0,015682909

RAB2A	0,325357808	6,49853482	0,009556708	0,015718087
DYM	0,337900859	5,758810678	0,009567063	0,015731325
SPDYE7P	0,406974395	4,533471577	0,009608841	0,015795194
POSTN	1,015605411	9,111277228	0,009610538	0,015795194
TMEM98	-0,554700005	6,301520145	0,009614593	0,015795376
IDI1	-0,446869852	6,789275241	0,009615282	0,015795376
TEX264	-0,295637163	5,802872602	0,009621292	0,015801443
AXIN1	0,372788688	5,663602781	0,009698811	0,01592492
C6orf1	0,427804028	4,983965402	0,009718788	0,01595388
FAM149B1	-0,3372016	4,677100685	0,009726488	0,015962676
CHCHD3	0,302427113	6,130276171	0,0098196	0,016111611
ASH2L	0,598024694	6,609669889	0,009836708	0,016135798
DLAT	0,309239999	6,14965294	0,009888783	0,016217319
OTUD5	-0,242345408	6,316926592	0,00991011	0,016248387
YPEL5	-0,339045626	7,488541988	0,009913764	0,01625047
SMARCD3	-0,59966756	4,89398399	0,00994018	0,016289855
RWDD2B	0,39372508	4,817008523	0,009944418	0,016292884
C1orf52	0,391856219	4,646117521	0,01000151	0,016382487
AP3B1	-0,278556637	6,251984546	0,010022661	0,01641319
VPS8	0,508472257	6,173963057	0,010054368	0,016461161
ATG3	0,337336816	6,38545951	0,010170854	0,016647876
GLO1	0,43923018	7,999909688	0,010173825	0,016648744
FAM18B2	0,310349525	4,15083904	0,010195499	0,016680208
KLHDC5	0,488469351	6,07771715	0,010201674	0,016686307
RFC1	-0,308459135	6,27955048	0,010204436	0,016686823
SEC31A	-0,284522693	7,979282881	0,010211006	0,016693563
STK25	0,292957845	6,818171675	0,010238996	0,01673531
EXT1	0,490500245	7,147821147	0,010293686	0,016820667
VOPP1	0,518200058	7,793906857	0,010311496	0,016845733
TRAF3	0,365207874	4,774699652	0,01040189	0,016989339
WRB	-0,361751629	4,83328662	0,010427417	0,017026953
SF3A3	0,291644798	6,987305221	0,010442401	0,017047338
SCAMP1	0,388862004	6,646162115	0,01046214	0,017075475
SCO1	0,28051647	4,719352322	0,010469867	0,017083997
STEAP2	0,557823769	5,409923234	0,010558366	0,017224282
SLC16A3	0,605163372	7,110395294	0,01056673	0,017233803
PRMT2	-0,296703635	6,76167571	0,010641576	0,017351723
GRSF1	0,279443865	7,198062229	0,010650095	0,017361463
SPA17	-0,503180845	3,219401589	0,010674542	0,017397156
NRF1	-0,166692208	4,62657369	0,010747519	0,017511907
WBP11	0,306585146	7,459030514	0,010750538	0,017512642
LOC100132832	0,427972788	2,605326166	0,010764651	0,017531444
C6orf47	-0,25870398	5,355952326	0,010789346	0,017567466
DFFA	0,307739171	5,637230213	0,01087664	0,017705373
TAF1B	0,366849978	4,720626704	0,010880067	0,017706725
GBP5	0,918258333	6,023241844	0,010914809	0,017759027
IKZF5	-0,336558831	4,86560413	0,010942722	0,017800196
ATE1	-0,295307286	4,075236945	0,010955607	0,017816906
HDAC10	0,40291782	5,105712201	0,011032979	0,01793647
ANKRD36	0,487865783	3,079267361	0,011034388	0,01793647

POMGNT1	0,352219315	6,873777448	0,011057245	0,017969341
DDX19B	-0,284138307	5,438848932	0,011075468	0,017994667
VPS16	0,298850536	5,862898465	0,011095537	0,01802298
AGAP3	-0,360300625	6,43335942	0,011119821	0,018058124
CUL7	0,441987745	6,188217287	0,011160729	0,018120241
ZNF652	-0,326082943	5,905589943	0,01124474	0,018252293
CDR2	-0,313426399	5,178831486	0,01130401	0,018344133
GOLPH3	0,330009961	7,853263144	0,011316779	0,018360485
KIAA1191	-0,245301874	7,219785758	0,011323686	0,01836732
APPBP2	-0,261105911	5,965274174	0,011330145	0,018373427
EEF1B2	0,499270529	9,158896096	0,011420626	0,01851575
HSPA8	0,325449493	10,83760123	0,011464142	0,018581884
HNRNPA1L2	0,274916855	6,493035566	0,011491851	0,01862237
HECA	-0,362247835	6,462110598	0,011524379	0,018670643
C8orf40	0,480713534	5,406355164	0,011578313	0,018753568
KIAA1586	0,47554298	3,581435685	0,011583023	0,018756741
PRDX3	0,451041914	7,69630827	0,011607105	0,018791275
BCKDK	0,29289328	6,24547935	0,011621471	0,018810066
PLEKHJ1	0,37480864	5,605811579	0,011640992	0,018837191
SERPINB1	-0,61863067	7,477337019	0,011647133	0,018842658
ST13	0,31594838	8,906228202	0,01174753	0,019000571
TRIP10	-0,37328049	5,938639715	0,011804445	0,019085192
LMTK2	-0,425999985	4,887083155	0,011805447	0,019085192
MYOF	-0,51140698	7,938333805	0,01183895	0,019134819
HNRNPA1	0,280832042	10,30960301	0,011891845	0,019215756
LAPTM4A	-0,296498471	8,825257199	0,011924108	0,019263324
PHKB	-0,326813491	6,748485586	0,01194146	0,019286787
C2orf69	0,314741526	5,027962224	0,011948846	0,019290409
DDHD1	-0,379405896	4,890946615	0,01194936	0,019290409
TRIM41	-0,304208721	5,064364164	0,011966823	0,019314027
SAR1B	0,48004658	5,453592905	0,011974287	0,0193215
EGLN1	0,44457911	6,762543055	0,012049945	0,019438981
S100A10	-0,515981541	9,080918182	0,012210642	0,01969356
GPAA1	0,380055838	7,266211133	0,01223106	0,019721825
CCNK	0,339342675	6,476600366	0,012270947	0,019781463
CREB1	-0,204667025	6,079342256	0,012302151	0,019827078
ZNF592	-0,32269378	6,211846821	0,01230558	0,019827918
AHCTF1	0,359146996	6,786612145	0,01235487	0,019902637
SYK	-0,452346555	6,625528445	0,012383123	0,019943438
XRN2	0,271849128	7,484210987	0,012393135	0,01995485
PWP2	0,343798899	5,659447246	0,012399356	0,019960154
GDI2	0,336167839	8,716072905	0,012481466	0,020087589
TTC8	0,389570377	4,529450875	0,012485226	0,020088899
RALY	0,291390165	7,884541831	0,012512162	0,020127491
COL1A1	1,070265852	12,21327808	0,012529072	0,020149939
EID1	-0,309890647	7,732701518	0,012589828	0,02024251
MX1	-0,675891942	7,226906683	0,012593752	0,02024251
WWTR1	-0,51900333	5,904022471	0,012595538	0,02024251
DYNC2LI1	-0,295394096	4,49764602	0,012710166	0,020421919
ABCA1	-0,465525321	6,573893565	0,012832795	0,020614094

DNAJC21	0,341579136	6,864150126	0,012897097	0,020710881
GALK1	0,51416178	4,747564894	0,012899122	0,020710881
ZNF461	0,491179398	3,192032298	0,012906059	0,020717142
NTAN1	-0,287335835	5,109542149	0,012911353	0,020720762
KIRREL	-0,633010388	4,524775379	0,012988759	0,020840082
C6orf62	-0,297912725	7,647364888	0,013050936	0,020934917
TMEM9	0,452850209	7,360910903	0,013057556	0,02094061
LOC283070	-0,560233541	4,344896437	0,013063496	0,02094521
VAV3	0,982716431	5,268868661	0,013074715	0,02095827
MAP1B	1,068863301	7,291157281	0,013152643	0,02107823
PGAP1	0,646896355	5,813169783	0,013189843	0,02113288
THOC7	0,325468658	5,941893714	0,013208377	0,021157605
PHF8	0,50118468	6,249341831	0,01324571	0,021212423
RPS15A	0,496180952	9,975935085	0,013270489	0,021247115
H2AFY	0,282273651	8,142840609	0,013278562	0,02125505
SAV1	-0,313019925	5,740880843	0,01330654	0,021294836
HDGFRP2	0,332377985	6,026784503	0,013312216	0,02129892
CUTA	0,379403796	6,951191299	0,013392023	0,021421582
SCNN1A	-0,771163702	7,67925336	0,013435541	0,021486152
TCTN1	-0,327268876	5,168439557	0,013554796	0,021669668
DDX26B	0,492313243	3,469943914	0,013556651	0,021669668
COG5	0,266481717	6,320925787	0,013594927	0,021725758
GBP2	-0,458581069	6,915331987	0,013742008	0,021955659
ZNF468	0,569234483	4,979055473	0,013822512	0,022079107
PRPSAP1	0,340993369	6,120772169	0,013835102	0,022094042
LPGAT1	0,331073469	6,703981925	0,013856573	0,022119242
KIAA0284	0,515213304	6,587753905	0,01385737	0,022119242
RTTN	0,494436379	4,282981937	0,013888984	0,022164517
PTP4A1	-0,443845167	8,386991704	0,013901767	0,022179726
HECTD2	-0,351940756	3,554590226	0,013979044	0,022297802
C16orf80	0,322286368	5,674561328	0,014102643	0,022489691
DPH1	0,256049332	6,131277195	0,014119518	0,022509331
KIAA1147	-0,409474074	6,103632219	0,01412156	0,022509331
ZBED5	0,330085373	5,830666299	0,01414035	0,022534014
PATZ1	0,4762971	6,085065565	0,014172869	0,022580559
CD248	-0,615649487	5,744125655	0,014213527	0,022640047
TRAPPC1	-0,267951186	6,509597773	0,014255136	0,022701021
CASC3	-0,297453817	7,130263443	0,014295956	0,02276071
RAB18	0,406618153	7,205279275	0,014369917	0,022873125
ANKLE2	0,286553164	6,893963964	0,014420224	0,022945798
GBF1	-0,30089938	7,03403497	0,014422304	0,022945798
PLAT	1,061674952	7,507862971	0,014475945	0,023025769
BRD4	0,385118416	7,254352135	0,014581876	0,023188855
NUP98	-0,273557489	7,323148884	0,014703897	0,023377448
C19orf66	-0,371695984	4,912639731	0,014711905	0,023384727
ACOT8	0,353045715	4,658749597	0,014715471	0,023384945
STK16	0,348622329	5,456215729	0,014742037	0,023421703
ATG16L1	0,30233328	5,443766115	0,014748997	0,023423447
SEPT6	-0,491949566	5,526850804	0,014750005	0,023423447
NECAB3	-0,41996375	4,93948818	0,01476196	0,023436974

ERCC8	0,302002192	3,607055084	0,014832146	0,023542925
PPP2R3C	-0,362067361	4,930217475	0,014873235	0,02360265
RLIM	-0,226771148	6,493459703	0,014906205	0,023649467
GSPT2	-0,526382894	3,855101236	0,014912337	0,023653692
DAAM1	-0,450098201	5,06371242	0,01499379	0,023777361
DCTN4	-0,257154803	6,620862568	0,015027547	0,023825354
RBM15B	-0,270629674	6,607347733	0,015080363	0,023903533
IGFBP5	0,889984428	9,871301468	0,015096214	0,023923096
STK3	0,289074331	5,556402183	0,015102228	0,023927066
ELMOD2	0,321687458	3,915028594	0,015222864	0,024112592
CMTM3	-0,432569199	5,75464272	0,015319471	0,02425998
LRRC16A	-0,382895712	5,628765497	0,01534512	0,024294955
DAGLB	-0,276373917	5,05737604	0,015409142	0,024390654
SERINC5	-0,493236673	4,190075874	0,015446907	0,024444758
GGA3	-0,291231449	5,722107057	0,015454147	0,02445054
GAA	-0,42166001	7,81061505	0,015512174	0,024536654
SLC29A1	-0,410035367	6,329775287	0,015609778	0,024683658
REL	-0,472264106	3,609301095	0,01561235	0,024683658
VPS13A	-0,361205189	4,971230952	0,015753384	0,024900863
EEF2K	-0,310206448	6,28656639	0,015776108	0,024931004
PABPC4	0,56972098	7,994395856	0,015791239	0,024949133
PPRC1	-0,332708371	6,257332192	0,015841763	0,02502316
ZFYVE27	-0,272249403	4,969246672	0,015863055	0,025050989
CCDC23	0,392838847	4,469367141	0,015909369	0,025115815
PRR24	-0,385317087	3,09768174	0,015911716	0,025115815
ATP6V1G1	0,345942302	7,420664575	0,015915154	0,025115815
EFNB1	0,670658912	6,989645664	0,016013014	0,025264401
TMEM185A	0,301326519	5,037268351	0,016097422	0,0253917
PI4KAP1	0,573831944	4,409039767	0,016245733	0,025619717
SUMO3	0,341341566	7,701067253	0,016317864	0,025727518
SH2D2A	0,573761285	3,331942516	0,016408834	0,025864964
MFSD1	-0,313094835	7,155256845	0,016550168	0,026081719
CMTM6	-0,31588864	7,575380305	0,016582121	0,026126037
NCOA2	-0,452752072	5,263634041	0,016648839	0,026225094
MPZL1	0,363580585	7,998017623	0,016737266	0,026358296
CXCR4	-0,587473428	7,064699667	0,016787202	0,026430832
NKTR	-0,374048667	5,655188357	0,016803245	0,026449983
TNFRSF10B	-0,433562493	6,570583953	0,016840413	0,026502371
PCCA	-0,440763144	4,437329148	0,016879925	0,026558423
BAG2	0,678829843	4,811523004	0,016884351	0,026559259
ZHX2	-0,409515139	5,832957015	0,016977448	0,026699543
SNRNP200	0,349061241	8,759178141	0,016989238	0,026711924
PPP1R12A	-0,20134828	6,587692253	0,017001441	0,026724949
PFKL	0,336573691	7,91926234	0,017063455	0,026816247
PIKFYVE	-0,353270029	5,564271517	0,01708327	0,026841202
WEE1	0,439072267	6,092406344	0,01709039	0,026846203
SPCS3	-0,324666595	7,550282859	0,01712799	0,026897196
MED22	-0,298179191	5,264669392	0,017130741	0,026897196
SMYD2	0,337479318	6,208857088	0,017184645	0,02697562
FAR1	0,361422715	6,61889738	0,017291869	0,027137688

ZNF335	0,29626694	5,082947869	0,017357197	0,027231488
MTMR3	-0,298941079	6,546267806	0,017359625	0,027231488
UBE2D3	-0,253129616	8,557287571	0,017430174	0,027331508
MAST2	0,418238129	5,98289653	0,017431402	0,027331508
PELP1	0,340454199	6,474947303	0,0175706	0,02754343
NGRN	0,319707457	7,299022438	0,01763744	0,027641853
POM121C	0,332619479	6,851770532	0,017655535	0,027663855
FAU	0,379280283	8,917287031	0,017742141	0,027793169
C1R	-0,503267639	9,140539274	0,017768281	0,027827723
COX18	0,361041791	5,115976236	0,017801233	0,02787293
PDCL	-0,268450358	5,190730939	0,017832755	0,027915876
ZNF24	-0,29894302	6,76929306	0,017838685	0,02791875
PTPN11	-0,323427691	7,520741266	0,017843873	0,02792046
ORMDL1	0,278611105	5,516424765	0,017875762	0,027963941
TMED1	0,344459343	5,073734216	0,017887019	0,027973501
PAK4	0,477705465	6,744266423	0,017890078	0,027973501
SLC9A6	-0,250791571	5,208021922	0,017906933	0,027993439
EIF5B	0,336645341	7,778712864	0,017983707	0,028107013
PLK1S1	0,513392839	4,549291749	0,017989459	0,028109561
CCDC9	-0,326146734	4,794163265	0,018077313	0,028234933
YTHDC1	-0,213871126	6,451831384	0,018077976	0,028234933
CROT	0,58626163	5,011129129	0,018140684	0,028326385
FTSJ3	0,302003339	6,446173986	0,018176913	0,028376459
DERL1	0,244750662	7,436231796	0,018261748	0,02850237
DECR1	-0,317133555	6,076814657	0,018289701	0,028539467
CDK11B	-0,280483056	5,64519084	0,018303413	0,028554329
MED21	0,38685241	5,759442187	0,01833729	0,028597037
DDRGK1	-0,29945263	6,008915466	0,018339375	0,028597037
ZMYM2	0,331848206	6,42373019	0,01834337	0,028597037
CTPS2	0,420883597	5,187027418	0,018350299	0,0286013
SLC39A3	0,308568987	5,040273683	0,01842156	0,028705808
SAFB2	-0,313632887	5,708102043	0,018428384	0,028709881
CPSF7	0,235473895	7,054425784	0,018439601	0,028720795
GOLGA5	0,260895758	6,465181538	0,018466097	0,028755496
EPS15L1	0,327994395	5,919038872	0,018481442	0,02877282
PIP5K1A	0,275202269	6,805527899	0,018499855	0,028794913
GTF2H5	0,376542208	5,544119782	0,018579542	0,028912345
MBTPS2	0,362424844	5,32684662	0,018656807	0,029025957
ODF2	0,321646983	5,963349188	0,018713966	0,029108242
SAP130	0,286783382	5,73944997	0,018792526	0,029223771
ZNF264	-0,318831259	5,660182124	0,018873457	0,029341936
UNC50	0,310908393	5,677203828	0,018877119	0,029341936
RCHY1	-0,39002652	4,869934759	0,018885749	0,029348661
LSM3	0,34132909	5,673084588	0,018960788	0,029458559
CUL1	0,254555876	6,837547273	0,019188941	0,029806239
CREBBP	-0,330801462	7,094069866	0,019211166	0,029833965
RNF166	-0,306971427	4,324920874	0,019270848	0,029919835
INO80C	0,476546048	4,755093233	0,019313015	0,029978478
UBE2Q1	0,22507631	7,140887402	0,01931829	0,029979841
NOTCH2NL	-0,390254907	5,221844674	0,019382376	0,030072451

SPATA20	0,432698048	6,048233581	0,019423954	0,030130105
RUNDC1	-0,243593596	5,235691221	0,01950451	0,030248181
TMX3	-0,308335192	6,082775766	0,019537246	0,03029206
NDUFV2	0,337874492	6,922873066	0,019542058	0,030292633
FAM96B	0,425720458	6,364102359	0,019577879	0,030341262
HELZ	-0,27208999	6,160272814	0,019604921	0,030376269
DDX59	0,328253539	4,887026039	0,019638236	0,030420975
PACS2	-0,298321147	6,321406342	0,019717249	0,030536435
NCLN	0,289510444	6,870618068	0,019797723	0,030654104
PPP2R1A	0,251208047	8,633494223	0,019928179	0,030849092
SNX27	-0,258723171	6,200508737	0,019995971	0,03094701
SNX16	0,374595838	4,123959655	0,020077926	0,031066798
MPZL3	-0,421337063	2,571778225	0,020090302	0,031078895
MLEC	-0,291748426	8,096895153	0,020197019	0,031236895
HSD17B12	-0,312593674	7,099084397	0,020295311	0,031381798
CYP7B1	-0,566653716	2,817104652	0,020310474	0,031398124
TPM2	-0,558199353	7,081339492	0,020395201	0,031521957
LSMD1	0,444835358	5,061617264	0,020460948	0,031616407
TMEM19	0,424609348	6,371571894	0,020512875	0,031689464
RBBP4	0,356041036	8,052098294	0,020518819	0,031691467
CHST14	0,355573191	5,645966526	0,020572394	0,031767018
MDN1	-0,409406516	5,654229829	0,02058263	0,031775629
TP53	0,681947475	6,755413852	0,020692403	0,031937867
F11R	0,45537607	9,571840053	0,020708141	0,031954924
LRRC47	0,246119923	6,320531327	0,020732517	0,031985301
LETM1	0,372965146	6,454215386	0,020744302	0,031996244
YAP1	-0,408364077	7,646351738	0,020879654	0,03219773
C1orf50	0,335011773	3,328494412	0,021051988	0,03245614
CHD3	-0,416379418	7,670739873	0,021069474	0,032475756
DDX23	0,200506942	7,462132818	0,021079227	0,032483445
TRIM27	0,27317607	6,501606877	0,021122378	0,032542589
FCF1	0,215579678	6,065532442	0,021209827	0,032669937
AKAP8L	0,320097679	5,637233819	0,021273094	0,032759989
HNRNPD	0,29427529	8,009524775	0,021311014	0,032808584
PHF14	0,364259959	5,867379004	0,021314273	0,032808584
RAC1	0,294125464	9,254441709	0,021344036	0,032846984
MTSS1	0,628852337	6,84406326	0,021358226	0,032856712
PMF1	0,356606316	5,954851616	0,021359995	0,032856712
ZBTB6	0,325603795	4,501775709	0,021516596	0,033090137
TMBIM1	-0,368582175	8,761780009	0,021548518	0,03312578
COBRA1	0,271241342	6,71811076	0,021549488	0,03312578
PPIP5K2	-0,25452723	5,370001469	0,021568563	0,033147629
GGPS1	0,253324007	5,878058848	0,021643112	0,033254704
LRCH3	0,36591575	4,876134655	0,021722647	0,033369392
PTK7	0,435557441	7,930333877	0,02175201	0,033406972
PTPN4	-0,254557975	4,064034396	0,021787868	0,033454509
STARD4	-0,441637283	4,289614446	0,021822756	0,033500534
RAB28	-0,262294226	4,391788447	0,021829138	0,033502789
HSBP1	0,46444457	7,718691178	0,021867955	0,033550254
SND1	0,28181959	8,411727359	0,021869905	0,033550254

SCD	-0,487772091	9,827670003	0,022025387	0,033776758
DDX58	-0,540154891	5,545671924	0,022027459	0,033776758
LSM14A	0,294569093	7,641852497	0,022038159	0,033785569
KDM5C	0,325166857	7,266924001	0,022044332	0,033787435
SMAD3	0,484299288	7,222764282	0,022129137	0,033907646
ARPC1B	-0,391702738	7,969344851	0,022132707	0,033907646
RPS6KB1	0,24444008	5,791606819	0,022342819	0,034221852
BRWD3	0,482043092	4,161786821	0,022353139	0,03422997
AP2A1	0,304055767	7,185649378	0,022386543	0,034273426
PEPD	-0,304274025	6,71938348	0,022727726	0,034787961
ZNF574	0,352813358	5,015203627	0,022762402	0,034833218
FLJ42627	-0,457050258	3,250229757	0,022831965	0,034928319
SRP72	0,305549376	7,579129071	0,022834792	0,034928319
RAB7A	0,272424071	9,050715437	0,023227464	0,035520986
C17orf62	0,280772803	6,711346927	0,023347399	0,035696393
MAMDC4	0,708845882	1,818737772	0,023371123	0,035724656
TTC31	0,244955636	4,976694961	0,023385178	0,035738128
SDF2	0,327208838	6,011892036	0,023419534	0,035782613
GDE1	-0,271861252	7,190672329	0,02344105	0,035803493
SLC25A6	0,435772398	9,47927244	0,023443701	0,035803493
TMEM87B	0,27245246	6,333818794	0,02348902	0,035864673
DDB1	0,232304293	8,630496283	0,023533515	0,035924567
ELL	-0,277357042	4,983466743	0,023552589	0,035945637
POLR3A	0,329921476	5,026266163	0,023560765	0,035950069
MRP63	0,356821986	5,760643229	0,023617605	0,036028735
CC2D1A	0,331608912	5,888189928	0,023638778	0,036052969
AKR1A1	0,343375158	7,48964907	0,024073283	0,036707451
TP53RK	0,310303519	5,215274713	0,024080352	0,03671002
PLEKHA6	-0,755648299	4,140232691	0,024146223	0,036789412
DDB2	0,400836498	5,53270957	0,024147983	0,036789412
KIAA1919	0,259338648	3,265175527	0,024148615	0,036789412
KLHDC10	-0,326730599	5,272283278	0,024210681	0,036875727
GNL2	0,320991419	6,512413074	0,024226965	0,03689229
IL15RA	-0,450847598	4,782091292	0,024241015	0,036905443
ERO1LB	0,681573745	4,869363292	0,024387545	0,037120238
RNF115	0,255339859	5,144271138	0,024423395	0,037166511
EIF4E2	-0,237004299	6,655448577	0,024429245	0,037167118
RPS6	0,537875224	11,31057863	0,024472182	0,037224138
SPCS2	0,288812404	6,72181213	0,024511831	0,037276131
IGSF8	0,336886503	6,195513832	0,024645196	0,037470589
RNF219	0,282063588	4,424985669	0,02465357	0,037474965
CPT1A	-0,395237053	6,998975773	0,024674406	0,037498279
ATF6	-0,247395027	6,771386872	0,024727629	0,037570789
RPAIN	0,338459007	5,364224583	0,024740343	0,037581733
C14orf119	0,355376267	6,507739493	0,024806286	0,037673511
CREB3L2	-0,4099446	7,08263231	0,024842468	0,03772006
SSPN	-0,539856079	4,810576802	0,024856671	0,037733223
C1GALT1	0,43725059	4,959108578	0,024874444	0,0377518
ORMDL2	0,29961591	5,884506906	0,024913734	0,037803016
FOXO3B	-0,317036084	3,568015158	0,024994903	0,03791774

TMSB10	0,502689059	10,57323953	0,025163011	0,038164273
SYAP1	0,324117556	6,178271669	0,025201934	0,038214808
AMPD3	0,443085541	4,64963533	0,02522911	0,03824751
LOC100271836	0,320751833	4,289346159	0,0253301	0,038392076
HERPUD2	-0,220272203	5,729457187	0,025353507	0,038419014
SMARCC2	-0,26857837	7,1945212	0,025403362	0,038486009
RPLP2	0,367035311	9,628652183	0,025496154	0,038618008
ZMAT2	-0,196868747	6,692073222	0,025518402	0,038643124
IMPDH1	0,406216829	6,742390734	0,02554392	0,038673177
ZNF148	0,305945142	6,51310862	0,025554641	0,038680821
AATF	0,303364581	6,478939747	0,025656086	0,038825754
EXOC6B	0,456319224	4,53374952	0,025724723	0,038920986
ATMIN	0,237881958	6,664952146	0,025792596	0,039009613
KIF3B	-0,284418414	6,624317742	0,025794742	0,039009613
TSC2	-0,339166179	6,389252736	0,025805637	0,039017437
SLC11A2	-0,257903809	6,668613009	0,025845741	0,03906941
SF3A1	-0,234169228	7,788786351	0,025871639	0,03909989
MED24	-0,274439422	6,564060941	0,025997744	0,039281768
CEP192	0,342220332	5,284568903	0,026023079	0,039311338
SDAD1	0,282633643	5,960212083	0,026083555	0,039393967
ZADH2	-0,314679453	5,420806774	0,026111286	0,039427117
DGAT1	0,370488823	5,805601457	0,026205984	0,039561348
UBE2H	0,377445341	6,836653386	0,026214134	0,039564892
MOSPD3	0,389059102	4,537008215	0,026356467	0,039770912
SEPT9	0,304928157	8,789066796	0,026444274	0,039894581
C9orf123	0,475518745	5,68458023	0,026505704	0,039978411
CHIC2	0,651752794	4,995665502	0,026703015	0,040267108
TTBK2	-0,412471592	3,020179759	0,027029095	0,040749811
MAPRE1	0,244912607	7,962466286	0,027081654	0,040820027
RCN1	0,387772051	7,815833158	0,02710451	0,040837001
PPWD1	-0,21927299	4,544098629	0,027104893	0,040837001
PLCB3	0,369061045	6,00292985	0,027144952	0,04088832
DNAJC11	0,249629472	5,892047384	0,027164583	0,040908854
THAP1	0,291651098	3,977865594	0,027203372	0,040958223
BPGM	0,426683504	5,592306539	0,027220967	0,040975667
IDE	0,287524123	5,453092254	0,027296949	0,041080975
UBR4	-0,294796648	7,64059569	0,027307942	0,041088451
COQ6	0,317348576	4,412218669	0,027338862	0,041125899
POLR1E	0,363007353	4,975649383	0,027420924	0,041240247
AP3S1	0,318512287	6,094702651	0,027442689	0,04126388
HEATR3	0,259118994	4,681371726	0,027553772	0,041418911
AGPAT1	-0,267345001	6,747581856	0,027557941	0,041418911
ZNF808	-0,392641424	3,772242065	0,027605452	0,041481176
GLOD4	0,239983977	6,330221465	0,027764462	0,04171092
HNRNPH1	0,240388588	8,245597481	0,02779961	0,041754524
VPS29	0,369456489	6,623327749	0,027838316	0,041803453
JRKL	0,32688333	4,391993633	0,027857529	0,041823094
FAM53C	-0,229258555	6,039519599	0,027983462	0,042002911
MAGT1	0,342373461	7,998205751	0,028003169	0,042023241
TAB2	-0,226510533	7,079074359	0,028067913	0,042111133

NARS2	0,400956676	5,021231254	0,02810704	0,04216056
DIDO1	-0,272729029	6,335139812	0,02817299	0,042250191
ZNF414	0,391160186	3,498404184	0,028197046	0,042276969
DUSP10	0,518522773	5,040999644	0,028221383	0,042304157
BCL2L13	0,318255465	6,719345854	0,028480044	0,04268251
SMAD1	0,362954944	5,862915777	0,028534769	0,042755129
RFFL	0,36594689	6,12145408	0,028547013	0,042764078
OR2A9P	-0,471616282	3,864676576	0,028580985	0,042805565
RIF1	0,321172545	5,772980814	0,028618084	0,042851716
DDX41	0,235419797	6,27641746	0,028663262	0,042909942
NGFRAP1	0,409285259	7,217359582	0,028684307	0,042932022
ARID1B	-0,287713635	6,638555006	0,028850096	0,043170683
TTC3	-0,319421168	7,470211769	0,028914063	0,043256911
DEAF1	0,343069129	5,055103743	0,029008688	0,043388955
SLC6A6	-0,418721865	6,931203043	0,02908596	0,043494991
AFTPH	0,270776136	6,674633422	0,029144937	0,04357363
MRPS36	-0,349990572	4,432276389	0,029182103	0,043619631
KIAA0947	0,348290869	6,44062992	0,029275273	0,043749306
PRPS1	0,433399732	6,328938537	0,029371847	0,04388401
RAI14	0,643892618	7,821707671	0,029415677	0,043939868
GAK	-0,353227801	6,445840786	0,029433153	0,043956345
KIAA0556	-0,389825561	4,933509386	0,029443639	0,043962377
BPTF	-0,310442282	6,565657529	0,029461398	0,043979263
FBXL20	0,347271919	3,669447954	0,029502579	0,044031097
METTL10	0,321718046	4,094154599	0,029803175	0,044469989
MGEA5	-0,247218303	7,117840605	0,029856727	0,044540149
ACTG1	0,270227286	12,26803944	0,029874184	0,044556444
UQCRC2	0,300552794	7,82564855	0,029958373	0,044672238
MOV10	0,324077635	6,137367762	0,030135623	0,044926719
ADI1	0,491466443	7,545115558	0,030160467	0,044953928
RPS6KA4	0,354703717	6,108670525	0,030186605	0,044983056
HGSNAT	-0,332235618	6,996595896	0,030197071	0,04498882
OSBPL3	0,389718448	5,56519729	0,030271766	0,045090252
SUGT1	0,289814879	5,285159029	0,030569366	0,045523587
RPL19	0,3111031	10,41751966	0,030606766	0,045569331
MAPKBP1	0,4471204	5,634597986	0,030658799	0,045636837
HSPA7	0,670488619	3,212945557	0,030711328	0,045705051
KIAA2013	0,246089471	6,626529253	0,030724367	0,045714479
ANKRD36B	0,437989272	2,856234147	0,03095696	0,046050504
MAT2B	-0,268487398	6,841192824	0,03119019	0,046387329
SNX11	0,295254698	5,648540048	0,031300949	0,046541904
ATP6V0B	0,487927384	7,739496375	0,031387283	0,046660101
NPRL2	0,318220266	4,375388163	0,03148136	0,046789754
ISOC1	-0,304961091	4,873389008	0,031522402	0,046840544
CUX1	-0,307183085	7,109346422	0,031619599	0,046974738
WAC	-0,213158616	7,464383898	0,031873368	0,047341428
GHITM	0,301529114	8,229070651	0,031951635	0,04744195
SUPT16H	0,308978346	7,637560054	0,031954961	0,04744195
ARHGAP5	-0,285024928	6,825587807	0,032068147	0,047599629
COL5A1	0,815527501	8,913800307	0,032165894	0,047734326

ASS1	0,825863626	7,761694961	0,032350914	0,047998451
CNST	-0,251046368	5,622265695	0,032421932	0,048093355
ALKBH1	0,269905537	4,077618067	0,032443161	0,048114379
DAD1	0,330321146	7,48861714	0,032479321	0,048157532
SIPA1L3	0,487103741	6,719013593	0,032863017	0,048715851
PAN3	-0,255794268	5,473782765	0,033061038	0,048998743
UBE4B	0,308164505	6,54532807	0,033127208	0,049086143
KDSR	-0,248345263	6,744640667	0,033181653	0,049156136
PPT2	0,342808757	4,718567716	0,033256338	0,049256075
RPL23A	0,322449256	10,04386415	0,033286523	0,049290077
TRNP1	-0,619548099	3,593901605	0,033375194	0,049410648
CFL1	0,229478568	10,2087824	0,03345725	0,049521377
ZNF124	0,338111734	2,858253058	0,033548404	0,049642943
NFATC2IP	0,259832755	5,81716876	0,033553941	0,049642943
MCFD2	-0,257056921	7,601999964	0,033586512	0,049680353
CNOT7	0,283571179	6,870639441	0,03361883	0,049717372
AGGF1	-0,196472812	5,404789604	0,033699523	0,0498259
MKNK1	-0,256368842	5,508030539	0,033709318	0,049829577
METAP2	0,188847437	7,267570974	0,033927777	0,050141637
USP35	0,406726011	3,411724264	0,033987109	0,050218439
MEIS2	-0,509807818	4,444970735	0,034153172	0,050452877
CMC1	0,386948471	4,09632086	0,034180733	0,050480868
FAM35A	-0,254389013	5,325393967	0,034186926	0,050480868
PTP4A3	-0,417726202	5,776481567	0,034243939	0,050554107
ANKRD52	0,331610807	6,400638958	0,034268891	0,050579994
EWSR1	0,218976495	8,213535125	0,034379595	0,050732408
ZDHHC8	0,373858424	5,736490789	0,034442226	0,050813834
CAP1	-0,316994212	9,104369685	0,034506641	0,050897855
NPR3	-0,759936374	3,798744365	0,034542604	0,050939882
MSL2	0,309407949	6,04328297	0,0346819	0,051134243
ZNF28	0,453045054	4,84817151	0,034748399	0,051221213
CMPK1	-0,261279522	7,684174322	0,034801157	0,051287895
KIAA1429	0,22503599	6,58458964	0,034934453	0,051473214
PATL1	0,268810779	6,635646831	0,035113178	0,051724493
EGLN2	-0,360145674	6,795625273	0,035120165	0,051724493
ZBTB25	0,293629372	2,984707729	0,035360689	0,052067488
NOMO2	0,333674516	7,902973862	0,035424557	0,052150271
MUM1	-0,254928268	5,130236418	0,035479052	0,052219222
GTF3C4	0,352446401	5,096907842	0,035588693	0,052369292
ARL17A	0,441317449	4,31443934	0,035602092	0,052377705
CIB1	0,41044615	7,568585466	0,035932083	0,052851785
PPP2R2A	0,294507548	6,427097335	0,036029341	0,052983411
C4orf27	0,308583026	4,639839714	0,036041679	0,052990126
ETV5	-0,542708011	6,558750036	0,036076399	0,053029741
TAP1	0,575592659	8,382776834	0,036195748	0,053193709
DYNLL2	-0,280530149	6,524924545	0,036216826	0,053213216
CAPN7	-0,210064268	5,310065698	0,036427117	0,053510665
MED12	-0,308893085	6,136578264	0,036536743	0,053660144
MRPS18B	0,299378517	6,578212614	0,03660704	0,05375181
ACADVL	-0,236732663	7,841837887	0,036624182	0,053765403

MGC2752	-0,21778304	5,597872947	0,036792095	0,054000279
GTPBP2	0,292674245	6,225739614	0,036820439	0,054030251
KIAA1430	-0,257787163	5,960434311	0,036846103	0,054056276
TUT1	0,242341656	5,054232486	0,036859466	0,054064249
GRPEL1	0,269601102	5,587023607	0,036983623	0,054234694
RAB3GAP2	0,211157017	6,143859931	0,037011217	0,05426349
ATG2A	-0,310396891	5,54479984	0,037049144	0,054307419
CNOT6	-0,228859264	5,880828534	0,037077297	0,054337006
PANX1	0,326821225	5,356888971	0,037135757	0,054410985
PGRMC1	0,323570842	7,724945776	0,037197309	0,054489463
SLC25A44	-0,217202685	5,925639471	0,03724102	0,054541777
KLHL23	0,518140164	3,664969327	0,037294797	0,054602672
CDC27	0,224677495	6,742652106	0,037298614	0,054602672
GCFC1	0,300562863	4,930964446	0,037458201	0,054824527
LAMB1	-0,470923181	8,045132273	0,037568369	0,054973972
SLC25A46	-0,258060951	5,295263864	0,037592305	0,054997196
OTUD7B	-0,313488895	5,588582987	0,037605659	0,05500493
SHKBP1	0,362529209	6,848987569	0,03766906	0,055085851
CSNK2A2	0,26672312	5,478732336	0,037678787	0,055088261
MAP1S	-0,302452932	5,593881772	0,037736898	0,055161394
C12orf57	0,444477833	6,844913674	0,037747224	0,055164663
PLTP	0,555084835	8,324872109	0,037795559	0,055223466
HCLS1	-0,533569412	6,797704099	0,037823147	0,055251937
ARFIP2	0,286271021	6,15195352	0,038069021	0,055598584
ANKRD11	-0,28700914	7,107879134	0,038076754	0,055598584
NEDD8	0,298270615	7,161448726	0,038104548	0,055627256
SMC5	-0,262021165	5,726726697	0,038222989	0,055788219
NDUFAF2	0,36082085	4,143524861	0,038267165	0,055840744
CTNNB1	-0,26687544	9,359995856	0,038538337	0,056224417
SNX8	0,356931509	5,32018578	0,038576881	0,056268609
GNAI1	0,461524342	5,974574449	0,038814029	0,056602409
AEBP1	-0,57070904	8,766179991	0,038861673	0,056659772
NDUFB1	0,436701907	5,821471397	0,038987477	0,056831039
GLT8D1	-0,198483714	5,75923372	0,039036892	0,05689091
TTC30B	0,385609999	3,998371441	0,039105579	0,056978834
RNF103	-0,224951129	5,668702924	0,039129053	0,057000857
CALM3	-0,234091345	8,257557431	0,039255376	0,057172663
TPT1	-0,388434777	11,98368405	0,039444316	0,057435573
GTF2H1	0,257808091	6,031347092	0,039471405	0,057462748
AHR	-0,396523334	8,203522222	0,039513828	0,057512229
ETV6	0,359481128	6,488670816	0,039546983	0,057548203
ING2	-0,291591337	3,712434146	0,039799829	0,057903783
UBTD2	-0,234548965	5,400768766	0,04007997	0,058298916
MESDC2	0,20714748	6,677898969	0,040095272	0,058308735
CORO1B	0,262775429	7,075524798	0,040170625	0,058405862
UXS1	-0,263460597	6,538023915	0,040309925	0,058595902
USP9X	-0,266879779	7,635736952	0,040385997	0,058693971
TPRG1L	-0,279498203	6,657532833	0,040427596	0,058741909
BIRC6	-0,254037484	6,886098784	0,040594884	0,058972414
PIGH	0,263986775	4,690160157	0,040617172	0,058992225

ATG12	0,247190118	5,193233183	0,040688081	0,059082628
MED18	0,294053699	4,763970819	0,040706326	0,059083503
GLE1	0,278306282	6,069285554	0,040713976	0,059083503
MAP3K5	-0,328320529	5,877128732	0,040714677	0,059083503
VKORC1L1	-0,256386279	6,709358882	0,040865237	0,059289373
CLN6	0,308132446	6,181678297	0,041067068	0,059563878
ELMOD3	0,257964916	4,167917665	0,04107191	0,059563878
FXN	0,259360353	4,32896981	0,041098519	0,059577067
SARNP	0,267074019	5,824353903	0,041103199	0,059577067
RFT1	0,192308154	5,281267575	0,041107215	0,059577067
SDCCAG8	-0,289698315	5,350356973	0,041139385	0,059611021
SLC41A3	0,273487336	6,195547028	0,041470387	0,060077878
ITCH	0,222761343	6,785212275	0,041494399	0,060087586
TYMP	0,605626438	7,720641668	0,041494712	0,060087586
RAB5C	-0,198084007	7,752230746	0,041572439	0,060176307
ASCC3	0,349868936	6,403077142	0,04157363	0,060176307
FASTKD2	0,206374195	6,051603717	0,041671944	0,060305811
FN3K	-0,397984643	4,14314422	0,041767794	0,060431697
RP9P	0,318389554	3,648654368	0,042021836	0,06078636
GOLGB1	-0,302941066	7,369287587	0,042041982	0,060802604
DEF6	0,310846714	5,054973622	0,042058635	0,060813789
TNPO3	0,275730013	6,359923746	0,042172928	0,060966122
SEC61B	0,273224595	6,56614377	0,042264216	0,06108514
TNPO1	0,311773124	7,522675653	0,04227831	0,061092561
GMFB	-0,270448732	6,700419809	0,042423922	0,061289983
NAT14	0,450274653	4,14544064	0,042689566	0,061660696
ATRN	0,386961862	7,282120731	0,042785601	0,061786322
ECHDC2	-0,384962038	4,941915198	0,042958187	0,062022418
PPIL2	0,28342599	6,073204199	0,043017482	0,06209488
CEP135	0,372951978	4,246986809	0,043114872	0,062222289
SNHG5	0,602776275	6,964337855	0,043210252	0,062346743
MON2	-0,189314482	5,739250518	0,043297848	0,062459917
DCTN6	-0,27121911	5,04928799	0,043326104	0,062487458
LRP11	0,400828917	6,407460028	0,043336519	0,062489263
PCDHB19P	-0,274058125	-0,063854865	0,043379376	0,062537836
DDX50	0,218825946	5,99525992	0,043491516	0,06268625
DIP2A	-0,262127931	5,340036389	0,043764763	0,06306363
PRSS23	-0,502649131	7,433787398	0,043771838	0,06306363
NBAS	-0,23701589	6,143514852	0,043793669	0,063081755
CTTN	0,520809288	8,409063037	0,043843631	0,063140384
SLC25A32	0,221971143	5,794319906	0,043883953	0,063185109
UBAP2	0,378119109	6,226666713	0,044041339	0,063398331
ANKH	0,538398524	6,958567471	0,044212945	0,063631928
USP22	-0,343773187	8,075552521	0,044243332	0,063662224
SPATA7	-0,349025163	3,021713195	0,044318128	0,063756396
DPM2	0,255681218	5,93033772	0,044514688	0,064019283
ARPC3	0,285617184	8,513761383	0,044519642	0,064019283
NFU1	0,339667826	5,384418526	0,044729559	0,064307583
CCDC12	-0,213886086	5,042230709	0,044753101	0,064327866
PGAP3	-0,369731807	5,558223315	0,044892706	0,064514934

SENP6	-0,223688716	6,47777517	0,044925737	0,064548799
FBN1	-0,583978108	7,389352276	0,044953468	0,064575037
METTL3	0,276388307	5,257593359	0,04540428	0,065208886
TFIP11	0,237505984	6,123559086	0,045639531	0,065532946
ADAM17	0,371811615	6,864756771	0,045673982	0,065568607
STRADA	0,201878721	5,350696303	0,045721689	0,065623279
CLIC6	-0,693105221	5,59146029	0,045802906	0,065726013
PHF10	-0,250567811	6,273962704	0,045952958	0,065927461
NDUFA3	0,344057484	5,265776951	0,046024028	0,066015534
EP300	-0,289181447	7,281853273	0,046047189	0,066034865
HSPA6	0,763329214	4,774191937	0,046060744	0,066040414
LYAR	0,305512621	4,882795842	0,046123462	0,066116436
RPS11	0,334412112	10,79764488	0,046229412	0,066254384
VCPIP1	-0,211855083	5,356906149	0,046269475	0,066297867
LRP5	-0,318192388	6,900662268	0,046589431	0,066742297
NIPA2	0,211020249	6,373416433	0,046619307	0,066771068
DDHD2	0,515888135	6,377293789	0,046665939	0,066823822
NUP153	0,261471627	6,855430404	0,047125444	0,067467647
C3orf52	-0,382063621	4,052796381	0,047165963	0,067511482
RXRA	-0,375439304	7,04095137	0,047193086	0,067536129
USP30	-0,215218618	4,404417076	0,047206113	0,067540596
MSRB2	-0,308097928	4,725142862	0,047484425	0,067924543
TJAP1	0,235287517	5,449243262	0,047526667	0,067970709
TAF9	0,268685513	6,234412064	0,047564952	0,0680112
RAP2C	0,23673862	5,955512892	0,047575762	0,068012395
C19orf10	0,289559404	7,018373055	0,047840821	0,068376977
ZNF880	-0,56862816	2,774148996	0,047927031	0,068485839
DNAJA2	0,245536251	6,942765439	0,048086586	0,06869944
CDK10	0,328050613	5,315802816	0,048111792	0,068721053
KCTD3	0,278906288	6,415955321	0,048156279	0,068770192
LMF2	0,31542242	6,72950538	0,048324163	0,068995491
TM2D1	0,332161274	5,20054959	0,048344201	0,069009652
C6orf226	0,405704291	2,524443619	0,048366311	0,069026763
MTIF3	-0,222813852	4,982880272	0,048515867	0,069225716
THY1	0,580530999	7,436918671	0,048660106	0,069417001
DGCR8	0,249761956	5,544947065	0,04871777	0,069484726
PARK7	0,248746606	7,823093527	0,048766873	0,069540215
RPS28	0,379477208	8,997697711	0,048815223	0,069594607
CCDC25	-0,228603189	5,585993915	0,04906486	0,069921853
FAM98B	0,28121908	4,694001264	0,049065269	0,069921853
PDGFRA	-0,573702194	6,630523043	0,049140055	0,070010465
SSR2	0,309450764	8,646627108	0,049147983	0,070010465
N6AMT1	-0,311934589	3,442700632	0,049160446	0,070013593
MRPL33	0,337833655	5,599123258	0,049250638	0,070127396
DCP1B	-0,36599131	3,931856821	0,049529694	0,070510017
RNMT	0,26178439	6,132787192	0,049639259	0,070651243
BNIP3L	-0,287026705	7,583925469	0,049691811	0,070711281
TMEM160	0,435581271	2,652895942	0,049827243	0,070876864
NUCKS1	0,248667086	9,055266254	0,049828961	0,070876864
FYB	-0,549394064	5,49078856	0,05000534	0,071102202

TRIM24	0,36788174	5,876611089	0,050008236	0,071102202
SLC30A9	-0,24804747	6,396681015	0,050060692	0,071161946
RECQL	0,272575358	6,113181877	0,050108621	0,071215233
ASB8	-0,172221027	5,290254357	0,050221168	0,071360313
CHKB	0,367168609	4,290440393	0,050448753	0,071668759
ZBTB7A	-0,260906105	5,586186286	0,050523297	0,071759709
C12orf23	0,364220025	6,410713768	0,050676816	0,071962767
ING3	-0,268081245	4,098167975	0,050853554	0,072184242
DICER1	-0,245023564	6,535288882	0,050853952	0,072184242
CIAO1	0,20277711	6,945746584	0,051054741	0,072454169
PDCD7	0,203136783	5,037411269	0,051119562	0,072531064
ABCA5	0,470622498	4,845767738	0,051171611	0,072589809
BCAT1	-0,544154589	6,404315303	0,051305209	0,072764189
CLK1	-0,310790862	6,011419306	0,051447835	0,072936717
SLC25A38	-0,22692653	5,326387301	0,051448249	0,072936717
TKT	0,448098995	9,262942653	0,051498523	0,072992814
NDUFB8	0,322925692	5,768341023	0,051787627	0,073387329
FTL	-0,425135893	12,75689445	0,051844644	0,073452862
PPOX	0,359664881	4,06263528	0,051973088	0,073619545
C19orf6	0,253925208	7,018754909	0,052184394	0,073903507
TNFAIP2	0,572458542	7,602033995	0,052343101	0,074112874
CYFIP1	-0,219630089	7,837100374	0,052447128	0,074244751
RBM23	-0,19364505	6,662119174	0,05249326	0,074294633
ACOT13	0,318950385	5,651403476	0,052615899	0,074452752
IL32	-0,559515525	7,417615649	0,05287034	0,074797271
ATP5A1	0,312039628	9,338840093	0,053069645	0,075063661
PIM2	0,58854656	6,713620032	0,053193914	0,075223829
HNRNPUL1	0,280442436	8,827860933	0,053359743	0,075442688
PRRC1	-0,185898658	6,525771463	0,053427615	0,07552299
KIAA0368	-0,261748152	7,153994962	0,053456438	0,075548073
STAT1	0,451196698	9,069781578	0,053616971	0,075759248
PNP	-0,375257567	6,604703024	0,053693681	0,075851919
EEF1D	0,288176478	8,986011134	0,054192721	0,076541045
AKR1B1	0,475898796	7,443396859	0,054259316	0,076619233
APOBEC3G	0,521063263	5,289307572	0,054448593	0,076870591
DHX16	0,211632471	5,983991437	0,054576068	0,077034612
SNTA1	0,445506184	4,774143057	0,055045566	0,077681232
CCS	0,269710565	4,90149981	0,055239609	0,077938939
SMARCA1	-0,388704324	5,885375376	0,05527876	0,077978044
DENND1A	0,27265992	5,407864504	0,055433091	0,078179575
GSK3B	0,279281639	6,939999963	0,055500365	0,078252237
FEM1B	0,253782367	7,170425957	0,055507563	0,078252237
COPB1	0,31876751	8,043560197	0,055578198	0,07833562
REXO2	0,25928607	6,039462349	0,055844	0,078693994
HIC2	0,484786856	3,741382721	0,056041925	0,078956589
NUP214	-0,252507046	6,485357759	0,056140227	0,079078746
RB1CC1	0,348503158	6,927081936	0,05618283	0,079122411
PIGM	0,264353012	4,855213489	0,056351609	0,079343718
CLPX	0,193093851	5,703487644	0,05719906	0,080520311
SEPT8	-0,243628484	6,191072983	0,057296571	0,080640933

HK1	0,351943029	8,323486884	0,057693131	0,081182307
PPIC	0,35362396	6,144104274	0,057907818	0,08146759
ZHX1	-0,249806317	5,701493158	0,058181211	0,08183533
DPYSL3	-0,43249918	8,129920041	0,058210913	0,081857909
ZBTB45	0,277274574	4,126830652	0,058221273	0,081857909
VPS26A	0,242507561	6,949009463	0,058260479	0,081896146
POLR2L	-0,274423454	6,501804987	0,058333681	0,081982145
STAU1	0,2025075	7,811514958	0,058410211	0,082072786
FXYD5	-0,43125652	7,036460839	0,058515	0,082194813
ZNF460	0,369870644	2,409929954	0,058521164	0,082194813
TOR1AIP2	-0,210774022	5,204963116	0,058740743	0,082486229
USP40	-0,279752751	5,702539338	0,059080499	0,082946248
PUM2	0,195260902	7,82979451	0,059138096	0,08301002
NT5C3	0,304875601	5,330075728	0,059195521	0,083073525
SORL1	0,542081358	7,665197434	0,059258841	0,083145275
DYNLT3	0,458615108	6,291182576	0,059293015	0,083176109
CDC16	-0,198115743	6,050841217	0,059567413	0,083543849
SEC23IP	-0,185345301	6,068865213	0,059669606	0,083669966
DAP	0,310713367	8,046914008	0,060003927	0,084121459
RIPK2	-0,298725167	5,076336168	0,06024834	0,084446748
NDUFB2	0,312115089	6,470076585	0,060277193	0,084469828
SMG5	0,247173079	7,216465721	0,060308504	0,08449634
GOLPH3L	0,298132634	5,941838921	0,060334522	0,084515429
OLFML2A	-0,469986109	5,886044647	0,060594862	0,084848735
SBF1	0,341073164	6,913200104	0,060597352	0,084848735
DHPS	0,290304906	5,85759437	0,060877842	0,085223979
TGFB1	-0,334320466	7,022701599	0,060942858	0,085297485
FDX1	-0,255334824	5,31889802	0,060957536	0,085300521
MAPKAPK2	-0,207048622	7,880828078	0,061105463	0,085489978
MPST	0,331106797	6,246365526	0,061184921	0,085583585
TATDN3	0,221664502	4,774273918	0,061205571	0,085594912
RCAN3	0,286861912	3,533244814	0,061248606	0,085637532
IRS1	0,565118501	5,83289887	0,061309308	0,085704832
IDH3G	0,199645438	6,064080214	0,061720693	0,086262228
ZNF506	-0,337577099	4,35554522	0,061774371	0,086319557
CDC42EP1	-0,385361199	6,898789784	0,061821441	0,086367631
SLC25A3	0,186031943	8,960636138	0,061969409	0,086539576
FAM185A	0,239032587	2,786251463	0,0619699	0,086539576
LATS1	-0,24550285	4,706645941	0,062031729	0,086608182
ASAP2	0,346370469	6,22405134	0,062128881	0,086713941
RSBN1L	0,228328386	5,000518055	0,062132911	0,086713941
ATN1	-0,275820039	7,969615645	0,062337898	0,086982223
MBD2	0,261795797	6,991245163	0,062418325	0,087076627
RND3	0,418134548	6,871572435	0,06250493	0,087168913
ADAM8	0,58980558	5,298784162	0,062510044	0,087168913
PDIA5	0,285132178	5,766226062	0,062598797	0,08727483
FLNB	-0,330598913	8,354775216	0,062886238	0,087657656
DCP2	-0,217226529	5,408961178	0,062943491	0,087719531
ARPP19	-0,207690082	8,062850736	0,063140706	0,087976394
ITFG3	0,26508412	6,948932402	0,063194468	0,088033315

SEC13	0,242199448	7,148576981	0,063305595	0,088170108
CLDN4	0,521338635	8,257840309	0,063472769	0,08837625
IDH3A	0,272525667	6,381019569	0,063479523	0,08837625
PCNP	0,272826967	7,74276217	0,063608771	0,088538112
SERGEF	-0,260121454	4,245692727	0,063927058	0,088962982
SMYD4	-0,231736631	4,478183892	0,063981627	0,089020754
CAMLG	-0,208839557	5,429624352	0,064132001	0,089211774
CASP4	-0,325443244	6,529906515	0,064558195	0,089786321
UBE2R2	0,274357152	7,193214781	0,065093485	0,090512332
POLD3	0,244369473	5,264737604	0,06529376	0,090772304
NRBF2	0,224407547	5,484931439	0,065664408	0,091268976
ITFG2	-0,271312297	4,915063009	0,065688972	0,091284512
TTYH2	-0,411452065	4,02644877	0,065740193	0,09133621
MED16	-0,23275403	5,939639781	0,065752963	0,09133621
PAIP2	-0,213347677	6,891373559	0,066151745	0,091871436
MGST3	-0,314463098	6,567061649	0,06658511	0,092454462
GRINA	-0,229806594	8,216233574	0,066947625	0,092938895
BRAP	0,175801106	5,274333248	0,067523399	0,093719124
BSG	0,253977608	9,314936924	0,067717602	0,093969541
NUPR1	-0,448729492	6,868679585	0,067760789	0,09401034
SAT1	-0,373047391	9,359303102	0,068068994	0,094418729
UBE2B	-0,255426176	6,347684831	0,068099911	0,094442403
TUG1	0,287706897	7,975047809	0,068125091	0,094458113
MED1	-0,263633091	6,234495088	0,06823932	0,09459726
ARMC8	0,217380947	6,053884444	0,068376562	0,094768247
NSMCE4A	0,262205592	5,173739188	0,068663301	0,095146322
PI4KA	-0,314263606	6,907417692	0,06889909	0,095442401
CXorf38	-0,175326939	4,234831796	0,068904963	0,095442401
KLHL8	-0,22691549	4,891426106	0,068944206	0,095477364
RHOBTB3	-0,402728128	6,101091037	0,068989945	0,095521306
ADNP2	0,243852038	5,784781599	0,069194825	0,095785528
TNKS1BP1	-0,307362406	8,020283469	0,069818649	0,096629463
XRCC4	0,284059005	3,586454252	0,069867733	0,096677775
DCXR	0,362399541	6,146542186	0,070001946	0,096843836
CYP1B1	-0,535465784	6,431299964	0,07004752	0,096887229
SAMD8	0,323269456	3,555666295	0,070093684	0,096931419
ZMYND8	0,346831286	6,206684267	0,07035317	0,097270532
AKR7A2	0,276813316	5,993293544	0,070738848	0,097766195
FBXW5	0,272922514	6,930812787	0,070740344	0,097766195
ATF4	0,258316235	8,911756066	0,070946312	0,098030983
SLC35C1	-0,301272043	5,693681669	0,071045733	0,098148472
HBP1	-0,214334416	6,948124368	0,071114613	0,098223728
SLC20A1	0,27935401	6,503222834	0,071409365	0,098610867
RHOQ	-0,265957637	7,09737851	0,071555371	0,098792483
NPC1	-0,307035389	6,991920532	0,071585157	0,098813601
GDI1	0,216580987	7,560575636	0,072078475	0,099474422
SMARCA4	0,350740748	7,703231558	0,072244663	0,099683601
NSMAF	0,23535493	5,950386041	0,072586583	0,100135123
IFT80	0,258742582	5,753602805	0,07284189	0,100459678
ITGB5	0,348903918	8,11802789	0,072851314	0,100459678

TAB3	-0,217296846	5,482394717	0,072986664	0,100625973
TMEM14C	0,342010381	7,174416492	0,073339833	0,101092444
HADHA	-0,164003911	8,245734273	0,073386335	0,1011361
UTP20	0,221431716	5,592903445	0,073825539	0,101720822
MRPS18C	0,220680575	4,407465434	0,073851562	0,101736121
SMAD4	-0,224816269	6,785028919	0,073897889	0,101779379
ELP4	0,244748833	4,347177615	0,074095618	0,102031103
MORC3	-0,211338371	5,602603603	0,074443115	0,102488916
RAVER2	-0,286734356	4,949429983	0,074542219	0,102604641
NEK4	-0,178209866	4,641039754	0,074817469	0,102962728
PACRGL	0,273368988	3,915122017	0,074879297	0,103027023
KCNQ1OT1	0,45378842	2,445908609	0,074978259	0,103142373
IPO5	0,240336685	7,913191205	0,07501178	0,103167675
GNB1	-0,174275003	9,144662928	0,075041633	0,10318792
ZNF772	0,356974368	3,863842412	0,07528219	0,103497833
SLC4A7	0,295364445	4,983151894	0,07542575	0,103674298
MRPL20	0,239201603	6,325320845	0,075457396	0,103696893
LRRC37A	-0,287669838	3,459417323	0,075477435	0,103703532
VPS13B	-0,245012054	5,661013718	0,075569608	0,103809257
TRIP12	-0,216341729	7,670002298	0,075667264	0,103922472
RRN3P2	-0,192415667	2,37789382	0,075730174	0,10398793
FKBP1A	-0,195362134	8,785120627	0,075894912	0,104193156
SMC3	0,234455555	6,674113567	0,076011288	0,104331918
UBR5	0,240394999	7,287469392	0,076393715	0,10483573
U2AF1	0,209254217	6,721452801	0,076453775	0,10489704
CD2AP	-0,230681812	6,4320244	0,076720345	0,105241608
LRMP	0,925437646	4,918258699	0,077184886	0,105854148
WDR44	-0,238982183	4,820465972	0,077197929	0,105854148
FGD6	0,321609058	5,870454983	0,077249595	0,105903697
F3	-0,535679656	7,193923737	0,077354462	0,106026146
MRPL43	0,30568464	6,236745265	0,077506866	0,106198022
PARVB	-0,33938197	5,488306882	0,077511007	0,106198022
KPNA3	-0,199669495	6,559146044	0,07774247	0,106493753
IFFO2	0,526350731	5,987470719	0,077851425	0,106621585
STT3B	-0,204263969	7,05229371	0,077989208	0,106788837
MFAP1	0,194107294	5,810296423	0,078111551	0,106934886
RPS12	0,326540243	10,38943088	0,0782707	0,107131253
LPIN1	-0,344901226	5,432972031	0,078378307	0,107257009
NR2C1	-0,199607009	4,533964923	0,078399647	0,107264686
KDM4C	-0,247274531	5,261398959	0,078636933	0,107566387
DNAL4	0,259342919	5,14189303	0,078651709	0,107566387
ANGPTL2	-0,442508752	6,378343927	0,078907922	0,107895152
PRR14	0,209814321	5,340469133	0,079191103	0,108260652
ZNF318	0,329858435	5,801739574	0,07929701	0,108383707
C19orf43	0,262596482	6,8266179	0,079725733	0,10894785
BMS1	0,238426498	6,471867915	0,079751074	0,108960644
RPL36AL	-0,255055234	7,481610505	0,079879507	0,109114254
ASB7	0,198886614	4,86913094	0,080179809	0,109502526
ZNF141	-0,267733847	2,384656906	0,080237018	0,109558715
GNG5	-0,230223439	6,813158414	0,080284763	0,109601961

CDIPT	-0,181989878	6,918900289	0,080394962	0,109730433
MPV17	0,224476014	6,177983895	0,080480068	0,109824611
DTX3	0,295085412	5,070198637	0,080526734	0,109866307
STX4	-0,188117088	5,621071202	0,080696291	0,110075617
KLF16	0,274070746	5,139589406	0,080744308	0,110119087
RBM3	-0,266163053	8,316628251	0,080768833	0,110130508
ZNF638	-0,205978137	6,668966225	0,08096595	0,110377212
SLC30A1	-0,257758198	4,375457019	0,081040994	0,110457433
YES1	0,252465165	7,044539284	0,081152561	0,110587393
DTNBP1	-0,222824396	4,570502881	0,081184476	0,11060878
DHX15	0,174682917	7,430862795	0,081336199	0,110793356
GFER	0,22600831	4,431961317	0,081375735	0,110825073
POFUT2	0,239985382	5,270447903	0,081607669	0,111118749
CCNY	-0,249932571	7,121912078	0,081660086	0,111167923
FBXO31	-0,194473442	5,092930126	0,081743085	0,111258702
ADD3	0,341168509	7,411294537	0,081834958	0,11136152
PRKACB	-0,294909736	5,895785841	0,081960332	0,111509878
BCL6	-0,293771179	6,879763879	0,082169955	0,111772775
RNF41	-0,188424929	6,224635127	0,082507877	0,112210054
AP4E1	-0,183325547	4,662847342	0,082600253	0,112313285
DNAJC25	0,221639406	3,779733708	0,082641227	0,112346597
NDUFS4	0,290097417	5,456617621	0,082674926	0,112370006
ABCF1	0,221946066	7,169518331	0,083105789	0,112933115
VKORC1	0,266186308	6,349769211	0,083129295	0,11294255
ARF3	-0,169802789	8,53101601	0,083211382	0,113031556
MAF1	0,200975842	7,441599303	0,083282216	0,113105244
ENTPD5	0,314314649	3,797037342	0,08342013	0,113269985
DIAPH2	-0,440685255	4,921641697	0,083492421	0,113333061
CDC37	-0,21549616	7,467744358	0,083499824	0,113333061
BAZ2A	-0,207891118	7,375614383	0,083685949	0,113563082
CHST11	-0,402474666	4,53661881	0,084059296	0,114047023
EPHA4	0,52126425	5,349807109	0,084225323	0,114249548
TASP1	0,216900956	4,132145469	0,084244104	0,114252296
POLDIP3	-0,172640076	7,083806624	0,084608551	0,114723745
LAMP1	-0,354149392	9,018651293	0,084736581	0,114874502
RGL2	-0,241433315	6,271177245	0,084823509	0,114969491
GSTA4	0,473605132	6,123013096	0,085352059	0,115662895
MTDH	0,195238079	8,022249771	0,085390854	0,115692476
TBC1D22B	0,17712207	5,027237856	0,085491475	0,115805795
THYN1	0,270464846	5,420887829	0,085747368	0,116129355
CD82	-0,370408046	6,912349569	0,085880576	0,116286665
TGFBI	0,565731949	8,88165892	0,086077376	0,116530003
FAM172A	-0,222164593	5,147116832	0,086112699	0,116554683
VAMP7	-0,185851008	6,165686991	0,086624757	0,117220472
TOLLIP	-0,23338057	6,369553192	0,086638976	0,117220472
SYNGR2	0,312136283	8,76761531	0,08667823	0,117245418
DNAJA1	-0,271332421	7,834305442	0,086691802	0,117245418
GLT8D2	-0,455536611	3,841404316	0,086987335	0,11762178
IER3	0,529842194	7,154224614	0,087288824	0,118006045
JMJD8	-0,205002112	6,365828886	0,087424141	0,118165553

ASAP1	-0,239984489	6,494524196	0,087704558	0,118521082
RAD17	-0,162118183	4,908565038	0,087823377	0,118658135
MSL1	0,179404577	6,752962897	0,08817313	0,119107087
INPP5F	0,307617181	5,04538688	0,08822492	0,119153442
JHDM1D	-0,250459632	5,678140592	0,088295312	0,119224897
MRFAP1L1	-0,197102034	6,212889302	0,088516068	0,11949932
RPL9	0,452300811	10,89546986	0,088564971	0,119541674
SEPT7	-0,182254199	7,88126521	0,088595529	0,119559254
CHUK	-0,171162772	5,38272394	0,088670808	0,119637166
OFD1	0,261375418	5,316432026	0,088920219	0,119949945
RPL5	0,299555774	10,39632731	0,089161546	0,120251698
NT5C2	-0,202587266	6,74444086	0,089222315	0,12030986
ATF1	0,19611696	5,329795147	0,08926598	0,120344942
ZBTB8OS	0,229025141	4,095918343	0,089655634	0,120846366
ZNF426	-0,284255562	3,368516341	0,090418805	0,121850955
ATOX1	0,271808931	5,46010412	0,090532865	0,121980559
NOTCH1	-0,367242338	6,708899744	0,09062	0,12207384
IFIH1	-0,400637939	5,584374249	0,090647354	0,12208657
ZNF607	0,488201306	3,320288092	0,090871269	0,122330866
BCAS3	0,301988653	5,926331605	0,090873577	0,122330866
ZNF484	0,254453214	2,849566325	0,090882559	0,122330866
ZNF410	0,241737285	6,637660578	0,091363763	0,122954312
CAP2	-0,404775485	3,476668018	0,091478261	0,123084109
RPS9	0,297896183	10,05710818	0,091619057	0,12324923
TGFBRAP1	-0,247691543	4,33651928	0,091681772	0,12330927
ARL1	0,213699432	7,059393362	0,091732942	0,123353762
ATF7IP	0,260125182	6,179045343	0,092412993	0,12424373
MBTPS1	-0,18986392	7,558285744	0,092453873	0,124274189
NDUFV3	0,195086724	5,349974948	0,092597743	0,124439705
CNIH4	0,298688236	6,601072192	0,092613507	0,124439705
PSIP1	-0,317742324	6,413773631	0,092646511	0,124459527
SPTY2D1	-0,20362245	5,826638315	0,092879937	0,124748531
FSTL1	-0,361660558	8,627654303	0,093124623	0,125052542
B4GALT5	-0,274691539	7,537673724	0,093256395	0,125204835
CCDC71	-0,184056189	4,690433524	0,093467729	0,125463866
CHD8	-0,236475201	6,684479014	0,093492904	0,12547296
ARID2	0,234856085	5,704276949	0,093658629	0,125670639
BMP1	0,375369748	6,001570996	0,093909358	0,125982276
LAMP2	0,236564482	8,689346319	0,09411298	0,126230607
PGS1	-0,197037971	5,471872491	0,094195001	0,126315774
VCP	0,305049158	9,249744062	0,094629511	0,126873502
RNF135	-0,233324643	5,022771168	0,094714091	0,12696194
FBXO28	0,164662531	6,236321152	0,094900622	0,127186977
SEC11C	0,533366127	6,529811066	0,094929803	0,127201087
DUSP5	-0,431180511	5,904289726	0,095007705	0,12728046
FAM165B	0,321340591	4,445341332	0,09522791	0,127550406
INTS10	0,247704161	6,360771095	0,095388947	0,127741011
KIAA1279	-0,184603257	5,174881258	0,095534825	0,127911245
ITFG1	-0,196654132	6,408451386	0,095947971	0,128439186
MRPL23	0,250255038	5,3480626	0,096053925	0,128555783

KIAA1432	-0,274660406	5,418719461	0,096238106	0,128771843
ERP44	-0,169068988	6,879525992	0,096253128	0,128771843
PSMF1	0,175243995	7,688611245	0,09637065	0,128903778
CCDC115	-0,199166169	6,051163124	0,096434719	0,128964178
SERPINE1	-0,479106121	8,284202378	0,096522645	0,129056455
PLEKHO1	-0,250828666	6,02677139	0,096767721	0,129344834
OAS3	-0,405521988	7,372769782	0,096776264	0,129344834
USP49	0,321855033	1,034732295	0,096863557	0,129436135
C6orf48	0,304114803	6,809630766	0,097049259	0,129658875
TMED9	0,212002877	8,041243792	0,098134575	0,131083186
HMGN2	0,27097815	9,358073844	0,098819239	0,131971875
SLC10A3	0,204551201	5,897757558	0,098886208	0,132035452
MAK16	0,239777254	5,103393499	0,099262879	0,132512445
ANAPC13	0,233006348	6,445546242	0,099501522	0,132805026
NNT	0,318219377	6,789415359	0,099618002	0,132934472
TIMM22	0,188431742	4,892450636	0,099703006	0,133021874
SFI1	0,272660462	4,485597843	0,099828417	0,133145564
MIER3	-0,200494418	4,749957931	0,099834766	0,133145564
SLC7A6	-0,180971654	5,99224399	0,0999376	0,133256647
BLNK	0,3986156	3,951520629	0,100261099	0,133651208
KCTD11	0,408074466	5,548480687	0,100272705	0,133651208
GSTK1	-0,250766076	6,860564739	0,100335911	0,133709317
TCF12	-0,206129576	6,629226533	0,100506546	0,133910539
ADH5	0,29851027	8,092493116	0,100576786	0,133961645
RPL29	0,271825469	9,859868737	0,100584195	0,133961645
GSTO1	0,368420952	7,743150608	0,100612521	0,133973205
TRPM7	0,253077314	6,122357084	0,100709874	0,134076656
TAF12	0,225138532	4,870142328	0,101065662	0,134524058
NRBP2	-0,322679857	5,089411637	0,102054954	0,13581435
FAM168B	-0,216071132	7,403954576	0,102179426	0,135953464
MGAT5	-0,338851521	4,337819257	0,102263846	0,136039244
PLEKHG2	-0,303529771	5,624664982	0,10240648	0,136202416
ATP13A1	0,227959195	6,704339491	0,102513523	0,136318197
FAM20B	0,276121483	7,231233354	0,102734237	0,136585057
PAPD4	-0,166785317	5,676137999	0,102901634	0,136780944
SCAF1	0,234446311	6,678590031	0,103007319	0,13689474
SLTM	-0,188766685	6,602059824	0,103031013	0,136899548
COPS4	0,218461388	5,918600236	0,103349258	0,137295654
BLOC1S2	0,235452392	5,986224476	0,103375363	0,137303585
WRN	0,265151578	4,496856663	0,103412493	0,137326152
CASP9	-0,237655237	4,569685506	0,103476387	0,137384244
MED15	0,287298064	7,25087047	0,103563013	0,137472491
RAB23	0,262631764	5,120911433	0,103641828	0,137550336
FDPS	0,277523478	7,853278357	0,104055592	0,138072597
PSME2	0,310400081	7,588965897	0,104471529	0,138597541
ZNF415	-0,453678108	2,81334518	0,105067366	0,139360897
VHL	0,190841284	5,45429969	0,105222674	0,139539754
PLEKHA7	-0,347256963	4,589168576	0,105355291	0,139688456
SMC1A	0,245686745	7,447286521	0,105885638	0,14036434
ANAPC16	-0,206643045	7,296668444	0,106638701	0,141335141

NPRL3	0,208950549	5,870253628	0,106678039	0,141359804
MAVS	-0,223168454	7,264268568	0,106734751	0,141407473
ZNF267	0,22928646	4,846887778	0,107229036	0,142034731
C3orf38	0,33137138	5,124017607	0,107352817	0,142171074
FUS	0,192101876	8,126152688	0,107887015	0,142850787
PPFIA1	0,413394407	6,750162007	0,108281366	0,143345104
TM9SF3	-0,152691907	8,166477883	0,108397623	0,143471155
HERPUD1	-0,27996537	7,645333764	0,108498221	0,143576435
BRWD1	-0,190204937	5,881525299	0,108772086	0,143910915
CLCN7	-0,21157632	6,508618157	0,10881678	0,143942119
CDC26	0,198051251	4,897131651	0,108858023	0,143968746
CD3G	-0,374426633	2,510661399	0,108932048	0,144038711
PHKA2	0,261859242	5,30614629	0,108997669	0,144097539
CANX	-0,173257696	9,92578082	0,109101073	0,144206284
GOLIM4	0,372005818	6,903529484	0,109249403	0,144374356
AIMP1	0,171010713	6,054624172	0,109452015	0,144614085
LAMC1	-0,272932523	8,580186926	0,109784351	0,145025085
KIF16B	-0,283628201	5,31239174	0,110018803	0,145306647
RC3H2	-0,206896762	4,909491818	0,110224675	0,145550359
SLC35D1	0,238256948	5,072744154	0,110286696	0,145604061
SF1	-0,143028192	8,067947689	0,110528361	0,145894869
CLCC1	-0,146782751	5,6268222	0,110668208	0,146051192
GATAD2A	0,217211333	6,954445356	0,111238141	0,146774938
ZNF337	0,250316621	4,63499423	0,111302585	0,146831559
RBM39	0,15688111	7,678125109	0,112185324	0,147967451
MIIP	0,25081987	4,986011562	0,112379451	0,148194833
HP1BP3	-0,16597998	7,849657708	0,113455111	0,149584378
EIF5	0,189723345	8,501734573	0,115023986	0,151608381
COG4	0,182816197	6,481959923	0,115034721	0,151608381
MKRN1	0,198486635	7,521817609	0,115091413	0,151653787
XRCC1	0,220210489	5,644794341	0,115237665	0,151817163
PPIL4	-0,167919467	5,430525875	0,115283274	0,151847913
INPP4A	-0,241915796	5,513761965	0,115367491	0,151929494
IFT52	0,269754517	5,653032933	0,115652723	0,152259085
CHID1	0,181602906	6,663946248	0,115662423	0,152259085
CERK	0,243120529	6,439408391	0,1157362	0,1523268
PIK3CB	0,260613748	6,18820237	0,115869399	0,152472681
EPB41L1	-0,313325683	6,574706938	0,116007921	0,152617002
ECD	0,173228073	5,328413576	0,116023836	0,152617002
TRAPPC2L	0,306895799	5,374414641	0,116085064	0,152668091
MAGED2	-0,246587537	7,644211103	0,116269684	0,152881406
C9orf78	0,171542112	6,485214802	0,116718588	0,153442077
C4orf52	0,257815614	4,736385172	0,117069298	0,153873466
ZC3H18	0,185196773	5,929808772	0,117647071	0,15460308
FAM53B	-0,269132699	6,199457186	0,117880686	0,154880231
RPS20	0,260055979	10,05653522	0,118081182	0,15511377
IL8	0,870181688	8,195522945	0,118375872	0,155470931
PARP10	-0,306690436	6,161233741	0,118610129	0,155748599
RAB22A	0,178680903	6,473780484	0,118840688	0,156021303
ATRIP	0,166411822	3,56397722	0,118882798	0,156046544

RFX7	0,245341423	5,410849793	0,119194565	0,156425662
MMP2	-0,435263697	9,109558743	0,119349229	0,156598498
FIZ1	0,344039079	4,663708384	0,119467099	0,156722999
ZNF765	0,215815882	4,346720394	0,119498606	0,156734178
FTSJD2	-0,163638684	6,207359302	0,120057283	0,157436657
NEDD4	-0,269935994	4,779147528	0,120327506	0,157760673
TRIM26	-0,182548852	6,520203872	0,120463491	0,157908601
CBLL1	0,157689762	5,49204495	0,120978985	0,158553854
SARS	-0,188837997	7,508488801	0,121180225	0,158787078
GPR107	-0,19802736	7,487046531	0,121219542	0,158808079
FBXO6	0,279872465	5,029842586	0,121258903	0,158829132
TYW1	0,242362239	5,090638804	0,12165092	0,159312007
SUPT5H	0,269558184	7,873025895	0,121944148	0,15966535
ING5	-0,210649581	4,557274258	0,122117983	0,159862262
LIMD2	0,313884996	5,221961084	0,122183509	0,159894654
VGLL4	0,235297974	6,718100402	0,122189624	0,159894654
RUFY2	-0,168141509	4,304259445	0,122334932	0,160050001
HIF1AN	-0,15581668	6,274170764	0,12235528	0,160050001
RGMB	-0,246646689	5,179925587	0,122446943	0,160139184
ZGPAT	0,231246042	5,157245227	0,122637357	0,160357456
TUBG2	0,202769687	4,824453497	0,122997806	0,160797937
SLC1A3	0,516405547	5,475493141	0,123046434	0,160830675
PIM1	-0,296922572	6,623801535	0,123150391	0,160935706
CHMP4A	0,212499575	5,931588796	0,123442974	0,161287151
PHPT1	0,29029973	5,873876395	0,12363928	0,161512692
ABCA2	-0,310842915	5,632443521	0,123702065	0,161563758
GCN1L1	0,224951833	7,626806969	0,123896599	0,161773585
AKT1	-0,184304493	7,910833477	0,123910168	0,161773585
DNAJA4	0,348346596	5,989004388	0,124172683	0,162085284
TLK2	0,147806539	6,051859129	0,124592368	0,162601982
RABGGTA	-0,196955669	5,33812286	0,124656719	0,162654835
ARFGEF1	0,206617638	6,832837226	0,124847629	0,162872773
HNRNPM	0,170522444	8,215024646	0,124923964	0,162941185
ZNF613	-0,279186021	2,709301314	0,12506083	0,163088506
FEZ1	-0,416221881	5,356593542	0,125299566	0,163368593
GCC1	-0,183660819	5,393057794	0,125374529	0,163435082
SEPT10	-0,254988667	7,103754095	0,125887441	0,164072336
CSNK1D	-0,157701359	7,627514395	0,125977974	0,164158953
DCTN3	0,331806118	6,092329288	0,126134646	0,164331706
SCYL1	0,156999592	6,917357539	0,126270841	0,16447772
C17orf49	-0,166058355	7,158337356	0,12650421	0,16475023
ZNF160	-0,242397845	5,089756352	0,126654314	0,164914219
LIMS1	-0,213303819	5,329742694	0,127023572	0,165363447
PELO	-0,183754197	5,29272413	0,127349745	0,165751067
RNF25	0,183718384	4,802546609	0,127369935	0,165751067
ATG4A	-0,245368712	4,717743325	0,127557478	0,16596345
DDA1	0,202132155	5,863905506	0,127613506	0,165986186
CCDC6	-0,191216261	7,056724341	0,127623636	0,165986186
ACADM	-0,221326361	5,752844666	0,1278611	0,166263318
HIP1R	0,285468696	5,781222475	0,127901273	0,166283847

AKAP10	-0,202589644	4,882366374	0,128249677	0,166705022
ACO2	0,20064247	7,574583003	0,128600299	0,167128919
ADO	0,170201047	5,49198962	0,128753682	0,167296371
DDX42	-0,172616496	7,285481079	0,129392358	0,168094207
IGJ	-0,573608285	9,733183066	0,129557322	0,168276453
SIPA1L1	-0,237724688	6,177479621	0,129817968	0,168582884
UBE2D2	0,168216847	6,955092689	0,130019618	0,168812599
ALG12	0,203195533	5,387606189	0,130179443	0,168987935
AGL	0,21496331	5,715243715	0,130372477	0,169206304
ZFX	0,250428156	5,543651691	0,130856883	0,16980268
PLCD1	-0,385800623	4,864263287	0,131104542	0,170091681
PLSCR1	0,328364834	6,947604766	0,131238043	0,170232493
PIN1	0,257218508	5,594024136	0,131387945	0,170394523
PPM1A	-0,150112688	6,4975795	0,13147393	0,170473613
ROBO1	0,481808392	6,614094103	0,131509985	0,170480372
MANSC1	0,367022156	6,054790448	0,131529145	0,170480372
FNBP4	0,226766108	6,059882634	0,131686173	0,170651465
USF2	0,196395056	7,257426924	0,13231651	0,171435736
HIBCH	0,223471515	4,96404015	0,132502198	0,17164371
SCFD1	0,198792883	6,109708068	0,132574323	0,171685659
MXI1	0,231860333	6,135207136	0,132584936	0,171685659
CAPNS1	-0,199979996	9,073007734	0,132672588	0,171766542
NSD1	-0,225143682	6,640911747	0,13271758	0,171792175
RNASEH2C	0,214245514	5,187843216	0,132860092	0,171944005
MAPKAP1	0,150530693	7,015112311	0,13294895	0,171978892
ZNF514	0,289965799	3,641936445	0,132959595	0,171978892
RCOR1	-0,227230057	6,297294371	0,132988438	0,171978892
NID2	-0,381055292	4,899908088	0,133004969	0,171978892
GIGYF1	0,265436473	5,924625413	0,133013151	0,171978892
NPHP3	-0,228254968	4,529890297	0,133052257	0,171996841
CCDC47	-0,158290484	7,602805041	0,133077722	0,171997154
PPA2	0,213512294	6,301334664	0,133138055	0,172042524
CDK5RAP2	0,45403014	6,814363936	0,133325666	0,172252314
HERC2	-0,25821043	6,483184223	0,133393203	0,172306924
CDV3	-0,177872276	8,13804163	0,133477593	0,172383277
GATAD1	0,240661778	5,977477327	0,133535669	0,172415896
MARCH7	0,171002646	7,117841858	0,133553419	0,172415896
CCNT1	0,272810377	3,817047167	0,133671726	0,172535965
ZDHHC24	0,192940996	4,383515111	0,133834626	0,172713534
BTBD7	-0,179936639	5,886536238	0,134327421	0,173316686
RANBP6	-0,227843164	5,11989183	0,134680853	0,173739829
SDC2	-0,418578137	6,827331677	0,134860178	0,173922522
PCDHB9	0,313114274	2,803493017	0,134873485	0,173922522
DCUN1D4	0,270077141	5,482317667	0,134923385	0,173953973
FNDC3B	0,250770839	7,729857544	0,135245912	0,174310162
PDGFA	-0,391504173	5,560764694	0,135250779	0,174310162
CWF19L2	-0,165678452	4,630154763	0,135532543	0,174640289
SNX17	0,186427321	7,813248861	0,135662511	0,174774733
DHX32	0,212391766	5,874613623	0,135903499	0,175027678
YWHAB	-0,139810155	9,015148985	0,135910185	0,175027678

CDC42EP3	-0,324574998	6,023340679	0,136000081	0,17510163
LARGE	-0,374740099	4,922102367	0,136018967	0,17510163
TXNDC5	0,230561675	9,13760953	0,136201257	0,175303204
C11orf57	0,162400032	5,69439459	0,136374903	0,175493577
VAMP8	-0,258740943	7,667289215	0,136420453	0,175519069
HSBP1L1	-0,271418336	3,481588597	0,136546513	0,175648118
CLASP1	0,259178056	6,653042744	0,136860138	0,176018347
SHROOM2	0,363485739	5,28835751	0,13740296	0,176683157
EID2	0,282462334	4,426870159	0,137481046	0,176750236
GNG10	-0,233243504	6,204673311	0,1376381	0,176918795
RBM12	0,11804293	7,014275969	0,137680339	0,176939735
VPS4B	0,193955589	6,774498876	0,138070198	0,177407327
CD300A	-0,3397023	4,625879894	0,138158763	0,177487679
RHOC	0,281202588	8,487353376	0,138192564	0,177497663
RAB3GAP1	-0,167125146	6,628450331	0,138264591	0,177556732
RNF114	0,191024453	6,944803848	0,139148542	0,178658239
BLVRB	0,398917735	6,904255543	0,139522564	0,179104737
GTF2H2	0,235885509	3,977079166	0,139844896	0,17945267
RARRES1	0,691446336	6,265397875	0,139846236	0,17945267
RAB2B	-0,169535663	5,260709834	0,14027144	0,179944874
SHISA5	-0,198650727	7,972599455	0,140282586	0,179944874
LMBR1	0,199047639	6,26098406	0,140356043	0,180005239
MDM4	0,228217786	5,904166443	0,140535825	0,180201916
RANBP9	0,176423169	6,410875426	0,140649496	0,180313765
TUBB6	-0,28741134	7,378304661	0,141300152	0,18111386
ZCCHC8	0,148035581	5,027711608	0,141362093	0,181159202
TXLNG	0,247286892	3,833059107	0,14170158	0,181560141
CCDC109B	0,307532269	4,771456061	0,141850952	0,181717385
ASXL2	-0,229090864	5,579711653	0,1420443	0,181930894
NSF	0,211725427	6,647059796	0,142157067	0,182041134
KIFAP3	-0,206610259	5,741727207	0,142347634	0,182250942
DCAF7	-0,155164505	7,716571025	0,142473649	0,182378039
ARMC7	-0,190218347	4,712944715	0,142754353	0,182703066
ZKSCAN1	0,249382862	5,689973283	0,142834597	0,182771461
TMEM64	-0,26772174	5,637356362	0,143074656	0,183044293
ATPAF1	0,170798781	6,08689817	0,143106117	0,183050199
ATXN7L3B	0,188516333	7,564120059	0,143153814	0,183076867
ATP1B1	0,41374637	8,756273588	0,144166877	0,184337884
PTPRA	-0,15835209	6,954107706	0,144600729	0,184857962
USP11	-0,213060767	6,997591849	0,144908448	0,185216628
PHC3	0,217339969	6,76739591	0,145614954	0,186084778
RPL23	0,236818802	9,732592864	0,146274765	0,186877287
C2orf18	-0,14967378	6,624961723	0,146289919	0,186877287
PRPF18	-0,135809306	4,800989675	0,146451804	0,187049045
LAT	-0,327245538	3,454632793	0,146540259	0,187126971
CPNE8	0,408254733	5,131570747	0,147779047	0,18867353
ATP5O	0,233663567	7,343062093	0,147862523	0,18872883
ZNF548	-0,190422959	4,250554135	0,147877715	0,18872883
MATR3	-0,115930297	8,546418165	0,14796746	0,188808029
LANCL1	0,222344278	6,684005772	0,148077039	0,188912503

ELAC2	0,155829241	6,376538152	0,148668248	0,189631272
KIAA1217	0,284380792	7,098376549	0,148991916	0,19000858
SVIL	-0,265883983	7,285248145	0,149045274	0,190041085
CPNE3	0,197498578	7,850888676	0,149444561	0,190514574
MTRF1L	-0,149059372	5,290735159	0,149554415	0,190618982
RPL10	0,221885166	11,12817746	0,149724238	0,190799771
MRPS27	0,169080848	6,423981662	0,150113817	0,191260486
SEPT7P2	0,21962922	3,64797938	0,150371327	0,191552789
ZNF542	-0,354028299	4,193930144	0,1514366	0,192873772
TMEM205	-0,202133992	6,096796759	0,151482645	0,192896388
CDK12	0,215703477	6,480122958	0,151643421	0,193065065
ECM1	0,46674345	6,04810867	0,151682695	0,193079017
RLTPR	0,481805503	2,479079911	0,151814714	0,193210714
ENSA	0,186147802	8,222302141	0,151885089	0,193210714
TRIM33	-0,156116046	5,89236866	0,151890314	0,193210714
RRP15	0,154096503	5,310855519	0,151899492	0,193210714
MAP4K3	0,17685121	5,866239638	0,152075309	0,193398271
RLF	0,249697016	5,653071059	0,15266814	0,194115989
BAX	0,168473556	5,948756804	0,152765343	0,19420337
PLCG1	-0,22527435	6,477569789	0,152926859	0,19437246
CRYAB	0,736918745	6,486363977	0,153234949	0,194727752
NIN	-0,222045229	6,098065697	0,153521857	0,195055998
DBT	0,148089545	5,71394546	0,153915372	0,195519545
NDUFA2	0,287323317	5,840897305	0,154356906	0,196043909
FAM168A	-0,234211399	3,751792529	0,154556093	0,196260335
MAP3K2	0,183262533	5,637359578	0,154805947	0,196541008
BTRC	-0,158251768	5,077172425	0,155788774	0,197707072
PTK2	0,1908517	7,493067958	0,155822899	0,197707072
PCBP1	0,135433801	9,055573554	0,155835452	0,197707072
PLXNA3	0,28666187	5,632139635	0,155840374	0,197707072
ZNF211	-0,225496992	3,977205782	0,156202802	0,198130005
BATF	-0,367449557	3,049965104	0,156241927	0,198142775
DVL1	0,241032477	6,085711425	0,156487166	0,198409945
ZFP36L1	-0,227766427	8,960640824	0,156510793	0,198409945
DDX21	0,214871502	7,588347131	0,156649096	0,198548362
RABGGTB	0,192880943	5,743773112	0,157113376	0,199070077
UPF1	0,219962718	7,45888576	0,157119102	0,199070077
CXorf26	-0,158241403	4,895334734	0,157268173	0,199221934
VCAN	0,469576718	8,345108291	0,157318567	0,199248758
AMPD2	0,178991672	5,805238786	0,157599695	0,199567748
PARP6	0,215240004	5,773338388	0,158887254	0,201160821
RGS10	0,299142225	5,005960394	0,158925013	0,201171276
HS2ST1	0,214566043	6,193754441	0,15914269	0,201396424
HMGN4	0,197744527	6,927159815	0,159161949	0,201396424
MAN2A1	-0,247782277	5,451166973	0,159639876	0,201963694
SLC25A43	0,24214503	5,4353949	0,160496556	0,203009835
ZUFSP	0,166740704	3,678599624	0,160551768	0,203042008
CCDC112	-0,235102843	3,155914393	0,160680692	0,203167372
POR	-0,205939685	7,526993085	0,160818381	0,203303771
TCF7L1	-0,337136649	5,508355944	0,161028609	0,203531805

LOC401397	0,23591994	5,77674242	0,161479328	0,204063666
RNF20	-0,173147433	6,278792246	0,162052053	0,204749481
ZBTB10	-0,298421258	4,599250693	0,162405854	0,205158488
UCP2	-0,348980014	7,363081378	0,16246522	0,205195468
SDF4	0,155222074	7,679187461	0,162871469	0,205670472
THAP6	0,176721573	4,347082853	0,163475515	0,206395026
FHL3	0,260200701	4,827986297	0,163970359	0,206981466
DRG2	0,15800205	5,47902709	0,164661605	0,207815563
SEC24C	-0,158541003	7,499198752	0,164751746	0,207890851
CMIP	0,23151657	7,348586097	0,164875829	0,208008931
UBXN2B	-0,185316219	5,461938402	0,164925094	0,208032597
BCAR3	-0,319535876	4,95231934	0,165006424	0,20809669
G3BP2	-0,174517042	7,768205704	0,165816276	0,209079361
LARS	0,168059332	7,173411569	0,165858795	0,20909431
UGGT1	0,171692314	7,370326234	0,16602921	0,209270458
PVRIG	-0,285038514	2,052895793	0,166502739	0,209816485
KHNYN	-0,175546157	6,714885561	0,166523951	0,209816485
PPM1B	-0,135105596	6,078029759	0,167422734	0,210909961
PQLC2	0,18463129	4,467226129	0,167969621	0,211559817
SETD1A	0,201805772	5,770930575	0,168036983	0,211605575
GALNT4	0,253672762	4,995350977	0,168785757	0,212509246
GAS2L1	0,29668945	5,585261435	0,168869017	0,212574826
MMGT1	-0,149374012	6,145617888	0,16921768	0,212974412
PRDM4	-0,155309877	5,657191851	0,170143419	0,214100014
ACBD3	0,14819712	6,927726395	0,170691684	0,21473532
ACTN4	-0,228383422	9,663090479	0,170711273	0,21473532
ZNF192	-0,237295474	4,429193383	0,170805119	0,214813742
PUM1	-0,13605099	7,008219858	0,170957884	0,21496622
ATP5E	0,211549984	8,126830453	0,171047222	0,215038903
CASP8AP2	0,18172574	4,760832141	0,172130185	0,216312902
SVIP	-0,331819956	4,081035006	0,172130275	0,216312902
ARF1	0,137091286	9,510294771	0,172155759	0,216312902
CDK9	-0,140093287	6,178882483	0,172219186	0,216352732
FKBP9	0,331320734	8,241294747	0,17255009	0,216728507
GLCE	0,272349832	6,125579988	0,172708844	0,216882124
NPEPL1	0,249175721	5,405442558	0,172736004	0,216882124
RASL11A	-0,466910328	4,091466537	0,173109047	0,217310492
FZD8	-0,414184299	4,113880718	0,173554902	0,217830089
GPX4	0,207557199	8,13839369	0,173899753	0,218222749
RNF5	0,226828265	5,99217364	0,174122933	0,218462609
GFPT1	0,217639192	7,33570312	0,174930236	0,219435114
ARFGAP2	-0,149532558	6,950112777	0,17513357	0,219611975
HIGD2A	0,198944236	5,951188393	0,175135638	0,219611975
RBM17	0,141550762	6,63180656	0,17531721	0,219799238
CTSB	0,311328167	11,4375597	0,175579919	0,220088137
KIAA0226	-0,178897819	5,962560688	0,175731256	0,220237352
FAM32A	0,158348553	6,835860891	0,176169555	0,220746085
KDM4A	0,224581016	6,581922009	0,176326686	0,220902384
STARD10	0,357776373	6,586917272	0,176724652	0,221360287
CLEC2B	-0,404544279	4,975032483	0,177682103	0,222518689

ZNF841	0,242023937	3,805603501	0,17781341	0,222642241
BCAT2	0,216337817	5,748329398	0,178093167	0,222951589
PPP3CB	-0,164760165	6,181951	0,178131534	0,222958688
LUC7L	0,226567845	4,984526043	0,178666446	0,223565202
SLC39A9	0,151541071	7,22610748	0,178681676	0,223565202
TRAF3IP2	0,250754713	5,787352712	0,178782131	0,223649853
DLST	-0,139340419	7,249166199	0,179242232	0,224184296
USP33	-0,143383463	6,4341469	0,179734521	0,224758794
DNPEP	0,139968315	6,714639994	0,180538531	0,225722817
CHMP5	0,283580998	7,351754995	0,180597438	0,225755074
MAP3K1	-0,205983599	5,605592985	0,180637898	0,225764264
RFXAP	-0,220482568	2,788359507	0,18119773	0,226422452
CKB	-0,389476212	6,729195765	0,18128474	0,226489674
DPP7	-0,195133398	6,927376163	0,181989385	0,227328378
NDUFA4	0,281857236	7,826632628	0,182025023	0,227331251
BAZ1A	0,199690744	6,651699195	0,182378874	0,227731467
HERC2P2	0,346843973	5,360519675	0,182658183	0,228038475
SYMPK	0,1854975	7,009565254	0,182694507	0,228042073
VAV2	-0,302618119	5,734686731	0,182835876	0,228176764
GTF2A1	0,223858434	3,975522797	0,182964472	0,228295468
RRN3	0,146674624	6,053237647	0,183050452	0,228360964
HACL1	-0,181390789	4,608113939	0,183175005	0,228474549
TTC1	-0,156693834	5,856103392	0,183341971	0,228640984
SOCS4	-0,140242746	5,301444861	0,183461012	0,228747603
RCOR3	0,170607467	5,555501034	0,1840368	0,229423572
EIF4B	-0,149886514	9,461125588	0,18410335	0,229464585
ZNF649	-0,282842369	3,525160796	0,184199545	0,229542525
UBE2Q2	0,2234139	6,377253154	0,184829566	0,230285549
DHRSX	0,219206213	4,916209719	0,185396117	0,230928708
UBE2D1	0,199478952	5,182979877	0,185437078	0,230928708
POLH	-0,158652315	5,239559198	0,185447368	0,230928708
EDEM2	-0,180002288	6,16829734	0,185621301	0,231103095
MDC1	0,240538776	6,109807416	0,185758488	0,231231677
HINT2	0,29978354	4,974848331	0,185851041	0,231304663
CLIC1	0,198486185	9,172129981	0,186489518	0,232056938
SRA1	0,186930008	5,50058652	0,186666455	0,232234731
WDR36	0,128131349	5,618858862	0,187143681	0,232785983
GULP1	0,503505848	5,46197885	0,187443006	0,233115786
SEP15	0,260997639	8,279099736	0,188618462	0,234534882
ACP2	-0,186625024	6,159330952	0,188820156	0,23474287
EVI5L	-0,196732934	4,666981494	0,188936044	0,234844128
MAP2K7	0,184794332	5,524358425	0,189292462	0,235244268
TEX261	-0,149595534	7,230467112	0,189397224	0,235331573
JAK3	0,342631049	5,146063215	0,189732602	0,23570534
MFSD10	-0,193413045	6,099688895	0,190414527	0,23650941
C17orf85	-0,153174216	5,093333265	0,190580067	0,236641444
USP36	-0,176013789	6,175712923	0,190590235	0,236641444
IKBIP	0,228192749	5,121649801	0,190670558	0,236698077
QARS	-0,123150619	7,608286492	0,190717201	0,236712886
MT1X	0,462222841	7,129900606	0,190886896	0,236878

S100A6	-0,292869166	10,33562523	0,190919708	0,236878
LAMA5	0,329017913	7,849780578	0,191223151	0,237211327
PDPR	-0,23321361	5,989168293	0,191373559	0,237354728
COPA	0,166664173	8,705872838	0,192449404	0,23864566
CTSA	-0,211646646	7,928605419	0,192704734	0,238918834
PHC1	-0,145688884	6,976905581	0,193233762	0,23953118
ADAM10	-0,168743252	7,157884567	0,193347897	0,239629101
ZNF276	0,221109007	4,863931371	0,193822443	0,240137848
NID1	-0,325816987	7,364929199	0,193828819	0,240137848
MMAB	0,203786506	5,753254336	0,19389267	0,240173319
TMED4	0,136545868	6,498152974	0,194535322	0,240925601
BCL9L	-0,230348311	7,001956403	0,194813314	0,241226074
TMED10P1	0,167421519	1,827391805	0,194930573	0,241327447
AGAP6	0,274491214	2,877783881	0,195099289	0,241492476
C5orf15	0,179198754	6,830175908	0,195593548	0,242060327
DDX46	-0,129749307	6,289658161	0,196133253	0,242683884
ATP9A	-0,230472741	6,668714509	0,196168583	0,242683884
SYVN1	-0,16891574	6,528337029	0,196243852	0,242721556
ABHD12	0,18149429	6,570036014	0,196270224	0,242721556
PPP4R1	-0,187180327	7,499903854	0,19653204	0,243001266
OAF	0,303123423	5,371453059	0,196973978	0,243503545
SEPT2	-0,138124127	9,180124775	0,197025858	0,243523532
DBNL	-0,158722971	7,471774744	0,197118191	0,243593502
EXOSC9	0,154246489	4,916073225	0,197279003	0,243748056
IFNGR2	0,195306174	7,299942386	0,197592714	0,244091434
FKBP8	-0,154675056	8,221535693	0,19859328	0,245283024
RPS25	0,189796454	9,366899516	0,198771681	0,245458909
SOX13	-0,25780107	5,987574654	0,199058019	0,245767995
FAM126A	0,247709159	5,79492813	0,19968574	0,246498382
PELI1	-0,234999616	6,727501648	0,200076481	0,246936022
FBXW7	-0,178734185	4,775371314	0,200415634	0,247309846
ATP2B1	0,35513328	7,188597765	0,200723719	0,247645204
PPP1R11	-0,14581298	6,696797159	0,201074466	0,248033065
INTS1	0,211806679	7,282836248	0,201118904	0,248043011
HEXIM1	-0,175392339	6,002307262	0,201350789	0,248284093
PLOD3	0,231520323	6,842491645	0,201801654	0,24879506
C14orf93	0,163133833	3,586709167	0,20183942	0,248796638
AKIRIN2	-0,142434638	6,086120728	0,203627458	0,250955293
RAB1B	-0,134344856	8,410639329	0,204163535	0,2515705
EIF3A	-0,155449878	8,472807659	0,204380622	0,251792495
UNC5B	-0,291257307	6,352618094	0,204995142	0,252503951
ZNF428	0,21478483	4,825408429	0,206348417	0,254124952
FAM114A1	0,230693861	5,542599329	0,206773974	0,254590088
ZNF646	-0,184766226	4,900190033	0,206800777	0,254590088
PEX3	0,20059143	4,628110412	0,20760256	0,255531022
MPDU1	-0,163526659	6,26600695	0,20794275	0,255903557
FUBP3	0,133619962	6,252191011	0,20799797	0,255925327
GPS2	-0,130614554	5,443896963	0,208121647	0,256009095
GUSBP3	-0,168445769	2,998957054	0,208141139	0,256009095
CDC40	0,149002233	5,597627396	0,209460513	0,257585435

POLG	0,157427075	6,219517451	0,210772461	0,259152075
NLRC5	-0,273846013	5,785876857	0,210950601	0,259300247
FAM160B2	-0,177539126	5,71764186	0,210969024	0,259300247
RRP8	0,137339814	4,223863643	0,211209069	0,259509174
ZC3H4	-0,161479262	5,879211448	0,211215122	0,259509174
SNAPC3	0,193994139	5,042135673	0,211702225	0,260060795
NXN	-0,242562003	6,653593201	0,212087508	0,260487161
PYROXD1	0,172736168	5,665801231	0,212141131	0,2605061
TAF5L	0,121606409	5,65044914	0,212216359	0,260551557
LEMD3	0,21919611	5,862829114	0,212338072	0,260654062
RPS13	0,212108771	9,145265649	0,21258181	0,26089761
EIF1	-0,148070142	9,906361833	0,212612996	0,26089761
PARP11	0,206668894	4,390563984	0,212820034	0,26110468
SPATS2L	0,19611629	7,30777216	0,213031534	0,261317148
CDKN2D	0,225677524	4,086091567	0,213262262	0,261553123
TMEM55A	-0,243630038	3,590980109	0,213580057	0,261895775
ZNF22	-0,186034213	5,322761109	0,214159392	0,262558953
SH3YL1	-0,255863432	5,744499754	0,214395898	0,262801659
ANKRD17	-0,172782642	7,179728697	0,214599566	0,262991058
SRGAP1	0,263443136	4,714813254	0,214627546	0,262991058
RNF14	-0,154781664	6,020795148	0,215524523	0,26404271
PRCP	-0,173857189	7,531919843	0,215620076	0,264112322
TARDBP	0,098410385	7,665294492	0,215862288	0,264361519
CYBASC3	-0,130054764	6,382744322	0,216168867	0,264689442
C11orf74	-0,175673193	3,644140874	0,216548008	0,26510608
CEPT1	-0,138930245	5,41128466	0,217339611	0,266027429
GABARAPL2	-0,204939349	6,786276741	0,217433931	0,266095114
COL1A2	0,477600675	11,68914833	0,217765382	0,266442612
IKBKAP	0,163825267	6,115921372	0,217796029	0,266442612
SPG7	0,142175152	6,048300965	0,21972951	0,268759736
RAB11A	-0,147317389	7,791373149	0,220103418	0,269168796
UBE3A	0,13341769	6,872113977	0,22106018	0,270290365
DCUN1D2	0,187152093	4,136612966	0,221443639	0,27071068
BST2	-0,379007014	7,179830016	0,221978299	0,271315652
NINL	0,261665319	4,353733194	0,222342338	0,271711901
SLC38A6	0,200033008	4,113977629	0,222820203	0,272247082
COL12A1	0,49234351	8,109178968	0,222994582	0,272411332
ANXA7	0,168405056	7,968221455	0,223040578	0,272418718
ALG13	0,20121548	5,053862397	0,223208254	0,272574693
OBSL1	0,267035054	6,040230811	0,223686868	0,273110252
PPP4R2	0,130056712	5,178759456	0,223768492	0,273161
ATP6V1E1	0,161920852	7,793309179	0,223990737	0,27338336
CWC22	-0,118463499	5,342775724	0,224560156	0,273980556
ANKRD36BP1	-0,204494701	2,991323401	0,224560394	0,273980556
SPCS1	0,14963628	6,817543921	0,22486032	0,27429741
PLEKHA2	-0,22584166	6,619273108	0,225087437	0,27452535
KPNA6	-0,117684455	7,044488527	0,225183206	0,27459304
THAP11	0,130290858	5,690906956	0,225399339	0,274779739
ZNF573	-0,201522035	1,05995277	0,225416903	0,274779739
PI4KAP2	0,243676096	4,471215218	0,225778445	0,275171263

SPIN4	-0,310468477	3,614701566	0,225861568	0,275223379
COBLL1	-0,243033491	6,35145682	0,225961713	0,275296216
UBQLN1	0,136691168	7,775545533	0,226087761	0,275400579
LOC146880	0,193851835	4,393284112	0,226221631	0,275513201
CAMK2G	-0,145681132	5,923933821	0,226261024	0,275513201
SURF4	0,15327454	8,754910272	0,226331139	0,275549373
FOXJ3	-0,142391359	6,442493281	0,226527775	0,275727308
MAFB	-0,240480648	6,474698144	0,226558162	0,275727308
HSPA13	0,230781328	6,209810243	0,226649271	0,275788969
THG1L	0,157948094	3,565965615	0,228493929	0,277983961
STARD7	0,169041608	8,484832942	0,229482439	0,279110971
CBY1	0,172130974	5,253799328	0,229502158	0,279110971
DGCR11	-0,193045673	1,845461445	0,22968068	0,279278274
CYB5R1	-0,21569264	6,608648435	0,229758749	0,279323393
CAPZA2	-0,21478114	7,863332832	0,230801657	0,280541266
ZNF673	-0,146140131	4,063888946	0,231186359	0,280958792
TMEM33	0,158407938	6,811100926	0,231736791	0,281577544
CCM2	0,198008792	6,050164192	0,232528138	0,282488754
ZNF227	0,179768687	4,09403441	0,232667137	0,282607269
NUCB2	-0,198286173	6,388450876	0,232741137	0,282607443
FADS2	0,361171865	7,571349178	0,232750168	0,282607443
SPTLC1	0,136828594	6,972427121	0,232814975	0,282635804
PEX26	0,160694714	4,976545033	0,233095945	0,282926531
CUL4A	0,224782824	6,93902991	0,23433992	0,284385819
WTAP	0,169114229	7,419072908	0,234406006	0,284415402
TAP2	-0,209143793	7,203670325	0,234578124	0,284573603
ANKRD27	0,18037121	6,112903931	0,235481602	0,285618827
HS1BP3	-0,150438301	5,693075436	0,236095467	0,286312465
ZNF280D	-0,134199467	5,088703195	0,236829401	0,287151438
MLLT3	-0,259190813	4,5728407	0,237146179	0,287484408
TRAM2	-0,191182252	6,448939854	0,237324746	0,287649741
ZNF45	-0,159505315	4,079381925	0,237835576	0,288192315
IFRD2	0,150112774	5,845510611	0,237856921	0,288192315
ACLY	0,166365507	7,998998917	0,238749992	0,289222988
AHI1	-0,188263241	4,151662887	0,239152349	0,289658946
SHISA4	-0,248414854	3,900087841	0,239358081	0,289856642
ARMC5	-0,147769071	4,403015746	0,239427803	0,289889593
ZNF302	-0,189640803	4,664102392	0,239470816	0,2898902
NMI	0,22512654	5,360761743	0,239532774	0,289913735
HCFC1	0,179817061	7,236567735	0,239653397	0,290008254
SEMA3C	0,381466577	6,721423791	0,239964802	0,290312125
SMCHD1	-0,170080424	6,348699593	0,239989654	0,290312125
SP1	-0,115295498	7,356636875	0,240511855	0,29089222
ARHGAP1	-0,186496961	7,640310848	0,240634156	0,290988528
MSL3	0,150220846	5,432010431	0,24155188	0,292046502
FAM134C	0,122678978	7,004260204	0,241750184	0,292234445
LOC642852	0,245570613	3,642962087	0,241804124	0,292247841
CNPY3	0,148280813	6,862734093	0,241966663	0,292392465
CTBS	-0,166807286	5,285952618	0,242023789	0,292409676
RAB3D	0,230664165	6,334722848	0,24206784	0,29241109

DPAGT1	0,145763799	5,862583897	0,242257117	0,2925879
SMAP1	0,153591062	5,975646383	0,242435482	0,29275147
ELL2	-0,219150155	7,123768838	0,242550041	0,292837948
ZNF710	-0,186313915	4,582442479	0,243827732	0,29432843
DST	0,369760312	9,064848872	0,24403873	0,294530991
BRPF3	-0,179123956	5,868138925	0,244410027	0,294926911
SH3BP4	0,206373933	6,996827638	0,244953479	0,295530392
LOC550643	0,203665687	5,285255175	0,245097066	0,295651317
AMMECR1L	0,113536628	5,63869338	0,246564661	0,297369017
ZNF101	0,158811216	3,356143021	0,246678215	0,297453359
ALKBH3	0,165365034	4,633505598	0,247095771	0,297904184
DUSP7	0,291096407	6,262437663	0,247612362	0,298474226
IGBP1	0,143020827	6,480852536	0,247693063	0,298518734
PLEKHF2	0,17637314	5,837883785	0,248492782	0,29942963
TBC1D25	-0,11105529	4,966123524	0,248556669	0,299453697
TMED7	-0,147596292	7,181242281	0,249299231	0,300295259
YTHDF2	-0,101801069	7,064060823	0,249830802	0,300882416
CCDC22	-0,114931055	5,008730214	0,25041522	0,301533001
CCDC111	0,164899996	3,837629695	0,2504834	0,301561848
C16orf58	0,118840399	6,894051747	0,250938679	0,302056638
RNF216	0,124462977	6,462110182	0,251319838	0,30246205
CCND1	-0,37696004	8,504593861	0,251454145	0,302570286
ERCC6	0,163980025	4,145024401	0,251945482	0,303108018
MBD1	0,151951233	6,183604293	0,252198077	0,303358385
NME7	0,16981503	4,755103817	0,252414736	0,303565447
C7orf29	0,294611696	3,982769454	0,252531699	0,303652558
ANKRD10	-0,197406402	6,103481068	0,252885294	0,30401267
TGFBR1	-0,201050752	6,616129449	0,252920352	0,30401267
SKIV2L2	-0,132686125	6,324215031	0,255130009	0,306614652
RELB	0,306890881	5,725319311	0,25519955	0,306644182
WDR41	0,142245439	5,421489931	0,255617072	0,307091757
AIP	-0,156583175	6,208187871	0,256141426	0,307650341
TPR	-0,154997946	7,466607989	0,25617226	0,307650341
PRKRIR	0,177010119	6,733271759	0,256606487	0,308117562
ZNF713	0,210284005	2,523254829	0,25675234	0,308238417
CD58	-0,222133395	4,662928012	0,257609022	0,309212449
ZBTB38	-0,192585766	7,320055631	0,258119246	0,309770352
MMD	0,241299322	5,184993157	0,259130601	0,310929362
ELOVL1	-0,13474876	7,043330295	0,259402871	0,311201298
PHLDA1	0,322236148	7,665141218	0,259669034	0,311465812
TRIO	0,2393594	7,157178335	0,260054021	0,311872735
POLR2E	0,131203886	7,274787453	0,260160049	0,311895032
ABCA11P	0,24935889	2,888692988	0,260164092	0,311895032
LDOC1	0,365020524	5,218739752	0,260475564	0,312213547
CDK19	-0,159563441	5,279215155	0,260693944	0,312420387
EPHX1	-0,2941545	8,392489625	0,261284662	0,313073293
GOLGA2	-0,13900364	6,629295314	0,261972281	0,313837486
BTAF1	-0,130479133	5,511300864	0,26201449	0,313837486
SNX7	0,241473851	5,011678433	0,262951713	0,314854433
STRN3	0,1580304	5,714651377	0,262955858	0,314854433

DNMBP	-0,192412024	5,434812522	0,263229261	0,315126463
CLPTM1	0,155370015	7,649091009	0,263393922	0,315268238
MFN2	-0,144772862	7,639811066	0,265021517	0,317108435
BBS7	-0,122765216	4,011540147	0,265024339	0,317108435
GPR153	-0,28625451	5,190727717	0,265429789	0,317537847
ZNF23	-0,141224733	3,592647418	0,266183047	0,318383125
ATP6V1C1	0,120704503	6,693366688	0,26632414	0,318496021
CCNG2	-0,184104981	5,693242461	0,266692313	0,318880393
C2CD3	-0,162216063	5,351719283	0,266986871	0,319176626
COG1	-0,123111207	5,625777863	0,267482372	0,319712936
HNRNPU	0,114733553	9,273345689	0,26763838	0,319843343
HNRNPA0	-0,11088936	7,593930226	0,268242986	0,320509711
MED13	-0,141529676	6,962484421	0,268313084	0,320537302
MGST1	0,383852053	7,986891833	0,268376155	0,32055649
TRNAU1AP	0,19680305	4,692330571	0,268430193	0,320564883
DNTTIP2	0,140560252	6,772276211	0,268491412	0,320581849
EFHA1	-0,156889386	5,525169788	0,268989545	0,321068342
CETN3	0,175646657	4,17014783	0,268993025	0,321068342
SNW1	0,09672817	6,59741349	0,269236747	0,321303006
GPAM	-0,201859601	4,588120467	0,269839856	0,321966401
FER	0,173193349	3,55049689	0,270574832	0,32273248
ERI1	-0,140986852	4,535984308	0,270576564	0,32273248
CUL3	-0,110316867	6,785732044	0,270626544	0,322735642
RNF169	-0,150532746	5,088996726	0,270718811	0,322789224
ZNF697	0,218555715	3,808852159	0,271011423	0,323081625
PPP2R5C	-0,124495387	7,36172512	0,271099151	0,323129717
SEL1L3	-0,204115074	7,541700262	0,271493611	0,32354333
ISCA1	0,190568456	5,744035569	0,271935204	0,324012958
NFX1	-0,15548333	6,177092197	0,272860064	0,325058137
EFHD2	0,234317841	7,572782553	0,272993316	0,325160074
STX8	-0,156965807	4,904567968	0,273134866	0,325271857
GGNBP2	-0,106571235	6,326216111	0,273266299	0,325371554
SPARC	-0,275019199	11,10918278	0,273805467	0,325956612
TMEM135	-0,142683843	4,702914998	0,274009957	0,326143114
RSRC2	0,096522858	6,265731588	0,274469898	0,326633549
PKIA	-0,311524351	3,724961791	0,274686919	0,326834775
RSF1	-0,160185291	5,493947053	0,274757625	0,326861871
SAP30BP	0,121283678	6,199169062	0,275062432	0,327167403
UBE2E1	0,140673949	6,644308067	0,27535087	0,327453363
FAM82B	-0,118813641	5,622745066	0,276240291	0,3284538
ARPC2	0,13552049	9,088768295	0,276746648	0,328998499
ADAM19	0,326696364	5,573998986	0,276796284	0,32900015
ZNF417	0,162883673	4,024680671	0,276869547	0,329029878
NFKB2	-0,215847312	6,268329943	0,277317979	0,329505366
LZTS2	0,139781773	6,321870734	0,277634365	0,329823821
COX11	0,170029517	5,697785678	0,278270331	0,330521754
PFAS	0,169573299	4,869690405	0,278421366	0,330643556
EIF3F	0,130308926	7,945085301	0,279661638	0,33205863
B3GALT6	0,165848182	4,943492443	0,279893448	0,332276014
NR1D2	-0,170758799	6,131809633	0,280290171	0,332689064

ALDH9A1	-0,141783891	7,089499724	0,280866015	0,333293703
RPL32	0,18285449	9,979234782	0,280908838	0,333293703
COX15	0,110750371	6,017862899	0,28094621	0,333293703
CDKN2AIP	-0,13496052	4,899413484	0,281125208	0,333448041
UBTF	0,119481439	7,338569937	0,281312926	0,333612668
SELK	-0,17198099	5,7545949	0,281520382	0,333800641
SPG21	0,111802637	7,288319496	0,282347874	0,334723602
ANGEL2	0,117746821	5,236259675	0,282955414	0,335385532
SETD5	0,139088632	6,956306703	0,283277382	0,335708805
ALG1	0,122357245	5,691225793	0,283790713	0,336258709
NAPEPLD	-0,136473865	4,624954069	0,2844031	0,336925771
REST	-0,172242467	4,312299051	0,284631289	0,33713753
NINJ1	-0,171543477	6,378893005	0,284915731	0,337415833
ADAM15	0,200603508	7,626378757	0,285011247	0,33747034
ZC3H11A	0,130673477	7,706491034	0,285089764	0,337504705
SLC35E1	-0,121396633	7,042303937	0,285583587	0,338030634
LSM6	0,149078648	4,36781392	0,285811709	0,338241937
YME1L1	0,106154961	7,751104256	0,286053222	0,338469013
EPHB4	0,213276628	7,313920767	0,28631605	0,338721225
MIOS	0,117569506	5,25335051	0,287587776	0,340166704
LGALS3BP	-0,171289855	9,831752895	0,287857104	0,340426222
ENTPD4	0,126408453	5,246203209	0,288257354	0,340840453
FTSJD1	-0,145668243	5,218901125	0,288676958	0,341277423
STRN	0,174392191	4,858506047	0,28923606	0,341879129
TIAM1	-0,245036829	4,964820373	0,289913904	0,342620955
DTX3L	-0,170938958	7,341980468	0,290046156	0,342717855
PTPN3	-0,165241907	5,636348416	0,290564429	0,343270763
RPS27	0,175773555	10,08825414	0,29062875	0,343287276
CCDC57	0,193496147	4,633551192	0,291321608	0,344046076
MAPK1	-0,120896257	7,915768849	0,291398021	0,344076728
BSCL2	-0,156542233	6,448030258	0,29145052	0,344079137
OAS1	0,305185713	6,193475377	0,292286054	0,345005817
MYO1E	0,195116323	6,301271806	0,292827742	0,345568147
AKIRIN1	-0,141895422	6,656640056	0,29286381	0,345568147
PPARA	0,149578526	5,559927458	0,295119663	0,348169721
STIM2	-0,169085235	5,645466524	0,295925642	0,34906019
C7orf31	-0,185491624	2,916134623	0,296747725	0,349964948
RNF13	-0,161988466	7,391182348	0,296795321	0,349964948
RALGAPB	-0,120909765	6,698995575	0,297039084	0,350191825
FAM43A	0,361657363	5,543114211	0,29861581	0,351975411
DENND4B	-0,121633322	6,050844184	0,298655188	0,351975411
VPS28	0,154174571	6,979398854	0,299395772	0,352787242
RAD50	-0,126536314	5,928671688	0,299942928	0,35337091
DYRK1B	0,220152741	4,945974403	0,300166313	0,353572998
RGS1	0,351224207	7,013497848	0,300411981	0,353753866
ACVR1B	0,14138583	6,302256654	0,300423616	0,353753866
ATF6B	0,13268322	7,102658218	0,301089445	0,354476679
ETHE1	0,223246768	5,479508036	0,301459462	0,354851039
KLF3	-0,13691932	7,008354745	0,301769761	0,355154988
SF3B1	0,095116208	8,58727091	0,302384256	0,355816781

MTMR12	-0,147323355	6,601840738	0,303648568	0,357242855
DNAJC14	0,085764042	6,658002478	0,303787236	0,357344344
FBXO42	-0,096822958	5,084122774	0,304593937	0,358231469
APP	-0,176483832	10,27492611	0,30505822	0,358715641
RAB21	-0,115880382	6,501802905	0,305457913	0,359123708
RASA2	-0,144914655	3,4835154	0,305878529	0,35955623
CSNK1A1	0,119063873	8,503279977	0,306312683	0,360004513
UBIAD1	-0,093455361	4,987186474	0,306529076	0,360196755
PEX12	0,147521269	3,769946675	0,306597825	0,360215467
SEC23A	0,18684139	6,671452018	0,306891277	0,360498125
HDLBP	0,148228872	9,76435904	0,308341257	0,362139
SEC23B	-0,108591225	6,969230029	0,308481805	0,362241678
NUS1	0,126385411	7,093203326	0,30871509	0,362453202
LOC349196	-0,217981491	3,346853975	0,308981622	0,36270368
MALAT1	-0,233745146	7,103153079	0,309165112	0,3627886
FBXO3	-0,131828424	5,369689417	0,309178607	0,3627886
NUDC	0,130452404	7,047213895	0,309213571	0,3627886
PHRF1	-0,152722372	5,971501792	0,309703043	0,36330037
SFT2D2	0,137768834	3,021323101	0,310073444	0,36367231
CD200	-0,299281978	4,509233856	0,312297664	0,366158405
MFGE8	-0,191078246	7,061366152	0,312300526	0,366158405
GOLGA3	-0,131184255	6,941575403	0,312878319	0,366772779
NLK	-0,131498558	4,759068371	0,313742517	0,367722623
RNFT1	0,188381624	4,5915016	0,313853873	0,367789922
MMS19	0,114513437	6,339477984	0,313919384	0,367803484
PRPF38B	-0,104663308	5,901084468	0,314098201	0,367949774
LMAN2	0,131052966	7,655706691	0,31442031	0,368263843
TSR2	-0,119862207	5,92220719	0,315372056	0,369315138
MED25	-0,132335291	5,684162259	0,316095868	0,370099197
BBX	-0,128260943	6,630354768	0,316239057	0,370203284
SBNO2	-0,163543381	6,920943058	0,318045691	0,372254303
EML4	-0,126519065	7,267179067	0,318268175	0,372383158
NPIP	-0,182415103	4,852627924	0,318308639	0,372383158
ZDHHC9	-0,172473488	6,819598144	0,318319611	0,372383158
LPAR6	0,286281467	5,427388325	0,31921135	0,373362297
CCDC7	0,167643774	0,865701523	0,319586601	0,373737101
MED4	0,118689177	5,91601312	0,319658342	0,373756899
FAM134A	-0,104585192	7,226712161	0,319739496	0,373787695
SLC25A19	0,167517453	4,512267504	0,320127577	0,374176478
TWSG1	0,161917195	6,600653394	0,320181808	0,374176478
PHACTR4	-0,109120077	6,339696156	0,32041861	0,374389051
EIF3G	0,137464221	7,277927264	0,320650361	0,37459565
MKNK2	-0,183848847	7,98128902	0,321530042	0,375558986
NCOA3	0,176104414	6,92818779	0,321708533	0,375703114
WWP1	-0,132221633	6,470879322	0,321898995	0,375861172
DNAJC7	0,112941811	6,58064273	0,322239501	0,376143076
ATP6V1A	0,124870627	7,458943734	0,322250748	0,376143076
STEAP3	0,219870995	6,48350206	0,322315717	0,376154522
TSC22D4	0,130698373	6,056044386	0,322855459	0,376719948
APOL4	-0,227379132	5,619951519	0,32306328	0,376897948

TMED10	0,122609915	9,111591479	0,323666053	0,377536575
SLC39A7	0,137998598	7,857170453	0,324368335	0,378291034
ANAPC2	-0,115898663	5,186365541	0,325116676	0,379087916
MIER2	-0,117244703	4,831365419	0,325162813	0,379087916
XRN1	0,128498974	6,253235843	0,326108574	0,380125533
STUB1	0,132745966	6,654353037	0,327173984	0,381302239
RPL22	0,128304248	9,268249177	0,327631765	0,381770507
BAG1	-0,178472837	6,794927213	0,32822804	0,382399967
CEP350	-0,120110362	6,137272658	0,328651029	0,38282736
GNB4	-0,193068116	6,727287217	0,328828962	0,382969205
SRRM1	-0,096127416	6,951975885	0,328986156	0,383086851
C7orf26	0,11331458	5,268744694	0,330736007	0,385058704
VDR	-0,215030832	5,675351728	0,331367395	0,385727938
WBP5	0,16747987	6,36536127	0,33220867	0,386641222
DOCK7	0,128916119	5,688199829	0,332702959	0,387150423
CYTH2	-0,108760174	6,382519352	0,332949479	0,387371182
NCDN	-0,129140031	5,287172373	0,333254736	0,387660192
INTS3	-0,114011554	6,898453533	0,333553794	0,387941893
FTH1	-0,160429213	12,18384005	0,334141868	0,388559584
OSMR	-0,226282403	7,128518288	0,334765591	0,38921851
CISD3	0,150046731	5,291148378	0,335046614	0,389429475
ARHGAP4	-0,247570953	5,374845217	0,335067115	0,389429475
SNHG8	0,172914057	5,099688746	0,335118369	0,389429475
SHMT1	0,166299876	5,542808775	0,336132541	0,390541455
IPO8	-0,10750631	6,512953705	0,33628659	0,390653877
CCL5	-0,270951319	6,534887516	0,336686617	0,391051957
HOMER1	0,154903048	4,382765012	0,337002542	0,391352237
WARS2	0,138469407	4,567712942	0,337892898	0,392319372
IPO7	0,118306491	8,064593266	0,337983864	0,392358183
HUWE1	-0,148655949	8,497051932	0,338271369	0,3926251
TSPO	0,202276354	7,386482358	0,338453029	0,392769095
ACVR1	0,148726869	6,273723327	0,339582087	0,394012294
SFRP4	0,406781327	6,204226943	0,339807013	0,394132681
GTF2I	0,117468619	8,454322026	0,339839869	0,394132681
LARP1	0,142813442	8,117472872	0,339859241	0,394132681
ATG10	0,139358672	3,447818079	0,340147111	0,394390872
RNF6	-0,114711644	5,890665431	0,340197552	0,394390872
PITRM1	0,150056234	6,786624759	0,341981854	0,396392021
PRPF6	-0,120906506	7,367726635	0,342237778	0,396621245
GALT	-0,168381073	4,618090434	0,342751469	0,397149069
PTPMT1	-0,113638495	5,779824173	0,342962932	0,397326577
PFN1	0,110856182	9,683001824	0,343561089	0,397951939
STK19	-0,122051112	4,462480478	0,34477067	0,39928519
ZMYM6	-0,091640431	4,825652227	0,345360197	0,39988639
KIAA1671	0,235008633	7,210026156	0,345407074	0,39988639
TM4SF1	-0,258081061	9,337215342	0,345835049	0,400313903
CDK18	-0,211353888	5,299970314	0,346772337	0,401330714
RAB11B	-0,130485022	6,992039301	0,34756883	0,402184262
HADH	0,162463945	6,313319546	0,347634415	0,402191902
RAB7L1	-0,133508686	5,466789882	0,347730748	0,40223511

FAM48A	-0,105036409	5,553397646	0,348560414	0,403126436
C1QTNF3	0,369711089	4,019802611	0,349306048	0,403920289
PSMB9	0,303404454	6,505154767	0,350669368	0,405392546
ALDH7A1	-0,274952728	5,715274018	0,350698142	0,405392546
TCTN2	-0,12625169	4,858637706	0,3508965	0,405553091
LOC100190986	-0,185874996	2,811821796	0,352088321	0,406861593
UPF2	-0,119836356	5,671643954	0,353303725	0,408196899
SETMAR	0,140260699	4,321594699	0,356119386	0,411380331
DCBLD2	0,235313389	6,772728732	0,356183352	0,411384533
ZNF780B	0,171688215	4,2927996	0,356822269	0,412052676
C11orf49	-0,132113144	5,206847817	0,357117942	0,412324288
TOX4	-0,092744641	6,923011134	0,357257837	0,412415979
ZNF564	-0,117981121	3,686705596	0,357853914	0,413034164
DOT1L	-0,160931039	5,724506949	0,358005398	0,413139077
CTNNBIP1	-0,140970206	6,342987728	0,358500216	0,413640097
EYA3	0,13064301	3,601128961	0,358883515	0,414012297
ZNF304	-0,135637875	4,41021619	0,359057187	0,414142584
COL6A2	-0,250140404	9,39661184	0,360066505	0,415236512
NMT1	0,094763744	7,444061893	0,360470884	0,41563256
C1S	-0,215856295	9,298716943	0,360795934	0,415937021
B3GALT4	-0,153698205	4,21603294	0,36092191	0,416011917
GPBP1L1	-0,10097147	7,407061347	0,361129553	0,416180906
LZTR1	0,146585876	5,922442842	0,362645464	0,417857286
DDX51	0,140607464	5,10124365	0,363428728	0,418689052
RREB1	-0,133489712	6,2247133	0,36369268	0,418922363
CDK13	0,102353457	6,098273226	0,363757249	0,418925972
LONP2	0,10246932	6,345629169	0,365758053	0,421151352
IFT74	-0,193654883	3,876512248	0,36581309	0,421151352
ERBB2	-0,189507083	7,56096535	0,366043835	0,421345866
DNAJC16	-0,091111191	5,237696469	0,366146455	0,421388321
BFAR	-0,086896934	6,66166639	0,36620431	0,421388321
GMPR2	-0,113766959	6,314312972	0,366789129	0,421990058
HEXDC	0,178002582	4,089445057	0,366886107	0,422030425
OXR1	0,128347519	6,166665104	0,369475399	0,424870735
GJA1	0,304704864	9,112814737	0,369497615	0,424870735
MTX3	0,110584757	4,869786784	0,36954221	0,424870735
CAPZB	-0,092376748	8,32499194	0,370039685	0,425370973
AMIGO2	-0,280040944	5,693499348	0,371437973	0,426906378
ATG9A	0,114131158	6,507476993	0,372072261	0,427563321
GOPC	0,102163493	5,902056441	0,372256076	0,427702474
TNRC6A	-0,121922597	5,815078143	0,372526843	0,427941466
NFE2L1	0,137913538	9,060165336	0,372757843	0,428134703
UBA1	0,109146107	8,835253865	0,373176671	0,428543571
ATL3	0,188076915	5,575961759	0,374409217	0,429886588
OSBPL8	-0,094551485	6,892677845	0,374493303	0,429910746
RAB40C	0,145201472	5,460346779	0,374712705	0,43009021
CWC27	-0,090802392	4,796667458	0,374836235	0,430159591
SMURF1	-0,126315514	6,602991128	0,37491959	0,430182852
ZNF430	0,173162738	3,486442744	0,375770472	0,431086616
ZNF81	0,121103248	2,954624289	0,375977073	0,431251078

ZFP112	0,195672239	2,586239437	0,376516879	0,431797612
GATSL3	0,167407943	4,438196998	0,376920603	0,432187926
APEH	-0,093523999	6,749886343	0,377080367	0,432298424
SEPW1	-0,18098451	8,061420195	0,378058176	0,433346563
UBN2	0,125076231	5,142749918	0,378227679	0,433467991
SPIN1	-0,09220434	7,053521265	0,379141949	0,434442774
MLLT10	-0,108239315	5,345727511	0,379780211	0,435101018
USP3	0,097248768	5,432378848	0,381162324	0,436611101
USP7	0,113692681	7,451478395	0,382081557	0,437517775
GSDMD	-0,150526674	6,422441852	0,382082175	0,437517775
ZXDC	0,122455793	6,421041003	0,382345348	0,437745622
ZFC3H1	-0,119127512	5,239717631	0,382513518	0,437864643
TMEM167A	-0,103110092	7,153012367	0,383626922	0,439065455
REXO1	0,133460069	5,42065253	0,384552963	0,440051461
GAL3ST4	0,173685128	4,618735309	0,385287775	0,440818345
HMGCS1	0,230535624	7,912132395	0,385673127	0,441185213
SLC25A37	-0,159035788	4,98235047	0,385972196	0,441453271
CXXC5	-0,223442315	4,196155226	0,386106443	0,441532758
EIF4H	0,087851939	9,069798807	0,386315281	0,441697501
ATP6V0C	-0,108170331	7,326664535	0,387931222	0,443470746
SKIL	0,15222675	6,01201214	0,389063566	0,444690657
CUTC	-0,124684734	4,322869406	0,390078777	0,445734945
LGALS8	0,11133185	6,294882043	0,390107956	0,445734945
COQ2	0,128109434	4,148126926	0,391260587	0,446939385
MALT1	0,142977966	5,620596266	0,39129317	0,446939385
MGA	-0,109541446	5,796831767	0,391879067	0,447447793
EXOC1	0,124776533	5,903205419	0,391933583	0,447447793
CLINT1	-0,096660206	7,123245368	0,391935133	0,447447793
EIF3L	-0,105308135	8,89967153	0,392317261	0,447809073
ZNF177	0,22863184	1,921691676	0,393212231	0,448755515
GDAP2	-0,091940313	4,724604328	0,39360422	0,449127707
CHMP2B	-0,169751711	6,157261437	0,393878378	0,449365344
RBCK1	0,111759518	6,96613235	0,395369549	0,450991126
DGCR2	-0,116648896	7,678864804	0,395803423	0,451410527
CES2	-0,153866762	6,322855184	0,397115488	0,452831189
CHST3	0,245469873	5,833932502	0,398834609	0,454715465
ZNF614	0,183677421	4,260759105	0,399166604	0,455017899
RXRB	0,108934188	6,065650971	0,399299878	0,455093744
PIM3	-0,13364746	7,011647171	0,400602656	0,456502258
COL16A1	0,275752673	6,409930272	0,400708547	0,456546631
ARID4B	-0,07507226	6,341542767	0,400889011	0,456675938
MFHAS1	-0,169820853	5,817961698	0,401637278	0,457451912
BIVM	-0,120503023	4,538962495	0,402800048	0,458699654
ZMPSTE24	0,133656976	6,821048748	0,403228509	0,459047026
DPY19L1	-0,170718794	6,245243638	0,403239725	0,459047026
INTS12	-0,084181578	4,642031353	0,403530898	0,459301818
NUP188	0,132257303	6,873550062	0,404358975	0,460167532
ZNF616	0,128872687	3,633027965	0,404550441	0,460308603
TREX1	-0,115990999	4,376398892	0,40500423	0,460748055
PTPLB	0,156685505	4,663964202	0,405503704	0,461239326

ANP32A	0,107445461	7,433275407	0,406252385	0,462013845
ZNF586	0,152431121	3,663422724	0,406331516	0,462026781
FAM111A	-0,129119924	5,676874497	0,406528609	0,462173822
ZFP1	0,117919172	3,946415991	0,406694968	0,462285879
PDLIM7	0,172451992	5,999688394	0,408043898	0,46374189
ZNF629	-0,131724038	5,541439878	0,408504878	0,464188429
LMBR1L	0,110666707	4,761919109	0,408660442	0,46428783
ADAM9	0,208850218	8,292189085	0,409403343	0,465054372
CEP290	-0,122818032	4,339387931	0,410012678	0,465668962
RER1	0,102359354	7,260328821	0,410628471	0,466290682
PHF21A	-0,109395601	5,74787883	0,411053217	0,466695286
CARHSP1	-0,121588597	6,545044987	0,411952946	0,467638944
BRD1	0,119305787	5,852311684	0,414002001	0,469886758
ZNF394	-0,079008987	4,851930734	0,414603964	0,470491668
HNRNPUL2	-0,111707964	7,615536111	0,417182821	0,473339378
ZDHHC6	0,100661301	5,527100074	0,417504317	0,473625343
ZFYVE21	-0,114255213	5,788266825	0,417904425	0,474000379
FBXL12	-0,092352973	4,564065454	0,418777374	0,474845609
ZSCAN29	0,099802431	4,993631594	0,418788898	0,474845609
PCIF1	-0,081032054	6,209458113	0,420498886	0,476705221
EPHA2	-0,204749612	6,815417166	0,421752782	0,478047245
ZBTB37	-0,113643925	0,876720052	0,421896212	0,478123898
AGAP5	0,149925624	2,29031489	0,421960642	0,478123898
GNB2L1	0,116661001	10,37106199	0,422235551	0,47835591
CLCN3	-0,116009607	6,189793317	0,422491651	0,47856654
PANK4	0,094175237	4,734781137	0,422695438	0,478717853
PLK2	-0,192278731	6,184296108	0,423147634	0,479150402
FRG1	-0,089023089	5,308910093	0,424050511	0,48009305
POLR2A	-0,123304405	8,532572288	0,425811298	0,482006514
NEURL4	-0,088165733	5,086688659	0,42669554	0,482927284
WASL	-0,092296939	6,652187406	0,427285905	0,483515198
CUL5	0,095207886	5,869997804	0,427476226	0,483532351
BIRC2	0,145165792	6,593251132	0,427543637	0,483532351
KDELC2	0,127543124	6,43532332	0,427557697	0,483532351
MYC	0,186053998	7,690950083	0,427584701	0,483532351
SLC12A9	-0,098579349	5,776838223	0,428305143	0,48426675
FAM175B	-0,070535835	4,990309768	0,428684401	0,484591683
LOC100288778	0,132238626	4,572412794	0,428734656	0,484591683
ZNF395	-0,133532187	6,702741897	0,429234262	0,485075975
BAZ1B	0,103219419	7,239056429	0,430624338	0,486566257
FAM104B	0,12043189	4,052144396	0,430785347	0,486667542
RCL1	0,138168403	5,012137757	0,431281516	0,487147367
OAZ1	0,115057494	9,604160271	0,431487368	0,487299165
ZFP91	0,077379454	7,189568663	0,432102978	0,487913596
NUFIP2	-0,088160935	7,178653617	0,432214676	0,48795892
COG8	-0,066696393	4,661461871	0,433245801	0,489042065
PTDSS2	-0,112636133	4,896131224	0,43361344	0,489376042
ICMT	0,089514757	7,251683752	0,433736422	0,489433834
DHRS4	-0,11835724	5,109742239	0,433897381	0,489534454
ZNF678	-0,103490584	2,102087628	0,434522711	0,490121422

SEC62	-0,103191502	8,126488713	0,43456139	0,490121422
RAB13	-0,082879931	7,016616483	0,435028018	0,490566571
HAUS2	0,084534835	5,754682503	0,435783537	0,491337292
FAM127C	0,134289824	4,946851329	0,436985214	0,492610709
PDS5A	-0,087834472	7,420548009	0,437304463	0,492889113
TRIM47	0,205743092	5,555462969	0,438251214	0,493874571
RBM25	0,080960261	6,66340391	0,438646118	0,494237917
UGP2	0,131356414	7,496704249	0,439967162	0,49557255
ARID1A	-0,117081426	7,313208148	0,439975981	0,49557255
XPO7	0,089107411	6,858850279	0,44018551	0,495726671
RBM33	-0,086250562	6,004755008	0,440793595	0,496329511
SNX13	-0,088143606	6,003235911	0,441854847	0,497442331
JKAMP	-0,091015097	5,843614978	0,442857029	0,498448726
SNAP29	0,103225998	6,07996614	0,442957251	0,498448726
CNOT1	0,099315051	8,235254123	0,442968072	0,498448726
PCMT1	0,104266699	6,548968173	0,44383372	0,499296655
LILRB4	0,250945775	5,428883352	0,443868063	0,499296655
CELF1	0,097059823	6,855648177	0,444553005	0,499984651
CHFR	0,129167408	5,191874089	0,444776994	0,500154076
MFSD11	0,078192039	5,589766346	0,445219439	0,500569061
ZNF571	-0,15136434	2,63947044	0,445367832	0,500653354
MLH3	-0,089359266	4,417184533	0,445745558	0,500995379
ARL2BP	-0,096192823	6,860523974	0,446339455	0,501580215
BRD7	0,093276224	6,546733454	0,446702631	0,501905625
SH3BGRL3	-0,117783908	8,472664869	0,447870864	0,503135325
SMARCAL1	0,079584706	5,172819282	0,448809556	0,504106796
CDC42	-0,100932015	9,004727689	0,449672113	0,504992447
LOC90834	0,13120013	2,137407784	0,450024453	0,505304915
PI4KB	0,075804613	6,964075292	0,450144929	0,505356976
GLG1	-0,100077664	8,381361991	0,450225809	0,505364575
ZNF664	-0,098393214	7,599695335	0,450479122	0,50556569
HARS2	0,081167237	5,149209645	0,450731108	0,505765251
RBM7	-0,094833048	5,604164748	0,451231802	0,506243774
NF1	0,133502128	6,881574952	0,453173728	0,508338814
ROGDI	0,136343974	5,194820489	0,453848752	0,509012276
GPX1	-0,135230966	8,317020947	0,454547047	0,509711613
AKAP1	0,113347709	6,623123654	0,455095383	0,510242588
PPP2R2D	0,085760166	5,207547017	0,455758378	0,51090192
BRD2	-0,077495489	8,420591754	0,45669564	0,511868436
COL18A1	0,2047995	7,95857837	0,45725488	0,512411015
WDFY1	-0,092203911	6,492810183	0,457646123	0,512765185
STAT2	0,116250193	7,51158052	0,458026775	0,513107372
ISG15	0,285339225	6,445484381	0,459755145	0,51495899
CIZ1	0,094614814	6,915875743	0,459880138	0,515014397
XAB2	0,104715293	6,142186877	0,460729216	0,515880546
CD2BP2	0,075719163	6,638090037	0,461481175	0,516637684
CYB5R4	0,127864067	5,315167445	0,463044904	0,518303217
ANAPC4	-0,073488416	4,810434072	0,464072868	0,519368602
ZNF776	-0,104874809	4,443645562	0,464753046	0,519987929
FOXC1	0,255464652	4,571459935	0,46480185	0,519987929

USP15	0,088319787	6,265582499	0,464855025	0,519987929
WDR26	-0,062518395	7,537786957	0,465469096	0,520589432
LOC100170939	0,142128341	2,032588049	0,466204494	0,52132641
CCDC106	0,156247064	3,887935512	0,467836793	0,523065928
C10orf47	0,180572452	4,482043739	0,468080932	0,523176413
TLE1	0,145129854	5,489308874	0,468195107	0,523176413
DHX8	0,090331188	6,434594996	0,468207678	0,523176413
LOC282997	-0,111876236	1,987831434	0,468242506	0,523176413
RSAD1	-0,089609832	5,149678294	0,468756735	0,523665166
COMMD10	0,167448117	5,006694445	0,469117526	0,523982377
WDR20	0,085851414	4,753498483	0,469411137	0,524224459
CRLF3	0,09679622	5,281590097	0,469823661	0,524599237
UNC119B	-0,097788238	6,217393473	0,47194376	0,526880239
NUDT4	0,114700461	7,249572259	0,473669344	0,528720127
RPL21	-0,126429238	9,945879799	0,474356708	0,529400718
GOSR2	0,070047663	6,24832544	0,476188457	0,531358057
C22orf32	-0,113423265	5,620428032	0,476885512	0,532048807
RSC1A1	0,105399404	3,838611514	0,477032914	0,532126197
EIF2C1	-0,105412973	6,013912099	0,478512547	0,53368941
H3F3B	-0,084419736	9,877249486	0,479112606	0,534271277
NSMCE1	0,08505683	5,800335617	0,479252094	0,534339443
THADA	0,063817354	6,092805653	0,47936147	0,534374017
RB1	-0,115992363	6,507006241	0,480109765	0,535120707
PCBD1	0,137210798	6,219284146	0,480326322	0,535274586
CAMK1D	-0,122912992	4,017882783	0,480472546	0,535350048
ZDHHC20	-0,102286034	4,676192456	0,482183198	0,537168311
EID3	-0,182408106	1,701539596	0,482434531	0,537360514
L3MBTL4	-0,199281617	3,629276127	0,482966944	0,537865685
DCLRE1B	0,092051411	4,346249014	0,483841138	0,538721745
FUT4	-0,125926527	3,9627556	0,483893633	0,538721745
TTLL5	0,09199584	5,303555655	0,484705199	0,539537177
PIK3C3	0,096272936	5,444052628	0,484819741	0,539576598
NXF1	-0,065371981	6,449232098	0,486359451	0,541201877
CCNL1	-0,118223447	6,633554426	0,48654415	0,541319067
DYNC1I2	-0,061399373	7,322678802	0,486692039	0,541395271
CCRN4L	-0,125551903	3,994491007	0,487448779	0,542148626
TRAPPC9	-0,102815227	5,68398735	0,488047593	0,542726115
PRR12	0,125613175	5,987431464	0,489872725	0,544666902
TPM1	-0,132573895	7,762430362	0,490375377	0,545136893
PPA1	0,113060994	7,902893203	0,490871324	0,545599276
TMCO3	-0,090948002	6,114719589	0,490994808	0,545647587
TXLNA	-0,077617351	6,976351016	0,491923778	0,546590882
ZNF503	0,146632986	4,965369242	0,493481067	0,548231899
ACTN1	0,154065994	8,548744391	0,493831404	0,548531739
UBE2L6	-0,136889741	7,389452665	0,49431088	0,548974901
PPDPF	0,135773742	7,847405851	0,494411835	0,548997607
PLBD2	-0,102665514	6,023439918	0,495505597	0,550122545
TET2	-0,099301286	5,487180437	0,496753584	0,551418312
NT5E	0,320780838	5,355852485	0,497015079	0,551618786
CD7	0,229944434	3,757763983	0,497944174	0,55256002

NEO1	-0,143104588	6,882262571	0,498444673	0,553025419
KDM2A	-0,089384716	7,597480108	0,499849119	0,554430253
KIAA0528	0,124351452	5,769220921	0,499873471	0,554430253
PLSCR3	0,098100218	5,653996687	0,501126197	0,555729311
KIAA0196	0,074168669	6,453411559	0,50190261	0,556499821
ARPC4	-0,0784195	7,842814328	0,503374564	0,558041156
SMEK1	-0,070105194	6,667510808	0,504528516	0,559229511
DNAJC4	0,087378144	5,032670003	0,505042635	0,55970839
SAP18	-0,086167799	7,167323838	0,506965224	0,561747784
TAGLN2	-0,105950738	10,43695004	0,508349165	0,563189757
ZNF320	0,172760536	4,789677932	0,508712156	0,563500356
ZNF585A	0,150907469	3,179529286	0,508807883	0,563514854
LYRM2	0,08346551	6,100802021	0,509902723	0,564635704
TMEM181	0,084108883	6,382328854	0,510339997	0,565004084
SRCAP	-0,079402306	7,848050768	0,510401108	0,565004084
B3GALNT1	-0,159203965	5,231385167	0,510609641	0,565143181
TMEM179B	0,086930133	6,404282599	0,51139773	0,565923584
C21orf59	0,083842301	5,435987245	0,511486586	0,565930072
UHMK1	0,130971647	5,203902973	0,511879912	0,566273381
ZNF431	0,117557129	3,781796294	0,512975335	0,567393156
ECH1	0,125173499	7,598139262	0,513214318	0,567565429
ASPN	0,271067873	5,472937958	0,513671708	0,567979143
USP6NL	-0,077935631	5,372438889	0,513923048	0,568164926
ZFP161	0,065121519	4,559241847	0,514991831	0,569254222
TERF2	-0,062465175	5,280511865	0,515812203	0,570068624
MCPH1	-0,07452072	4,828568587	0,517003289	0,571292405
TYW3	-0,078599868	4,952480084	0,517266975	0,571401356
VPS37C	-0,068087434	5,628353078	0,517269478	0,571401356
S100A13	-0,127242233	6,394981615	0,518108752	0,572235759
MRPL54	0,143644302	5,275177404	0,519213415	0,57336296
CENPB	0,094151725	7,210910683	0,520365261	0,574519297
RAB35	-0,056468541	6,302855893	0,52042905	0,574519297
DDX6	-0,062366924	7,767227907	0,521813877	0,575954812
PPARD	-0,092689576	6,379931027	0,523158208	0,577345173
ZNF224	-0,101951263	2,976403476	0,523388732	0,57750611
CLTC	-0,062416267	9,415566325	0,52386416	0,57793718
UBXN1	-0,057884473	6,723264354	0,524668388	0,578730789
MYO10	-0,130032936	7,389649032	0,524826625	0,578811702
TUBGCP6	-0,094561846	5,472517448	0,525172692	0,579099707
SLC35A5	-0,085438732	5,735263999	0,525270694	0,579114125
NEAT1	-0,154837617	8,548002365	0,525707118	0,579437097
BTG1	0,110644413	8,476904652	0,525733586	0,579437097
FUT10	0,120589741	3,439895139	0,52591959	0,579471189
BCR	0,108437539	7,310004957	0,525934476	0,579471189
C9orf91	-0,079546378	5,15888972	0,526352747	0,57983835
RNF144A	-0,115466789	5,208861671	0,528351471	0,58194616
ZNF397	-0,097491187	4,151570955	0,530174933	0,583860282
ZNF17	-0,086056043	3,002053735	0,530453469	0,584072696
DZIP1	0,122849277	4,590688046	0,531008644	0,584589593
CTSC	-0,12008981	9,108388127	0,532207063	0,58581436

RAI1	0,122436022	5,973661183	0,532578568	0,586121704
EEF1A1	-0,091994425	14,21294869	0,532658191	0,586121704
RAB3IP	0,112768718	5,531919861	0,532837292	0,586224184
PRDX5	0,115207879	8,286857816	0,533000938	0,58630963
ADIPOR1	0,064594244	8,048391095	0,53416031	0,587490186
TBC1D22A	0,077093404	5,585420248	0,536701387	0,59018977
TMEM126B	0,115320548	5,353555425	0,537051685	0,590479755
TPST2	-0,088541602	5,904533332	0,537166063	0,590510299
GTF3C1	0,13979159	7,281748703	0,537461778	0,590740145
AP1B1	0,079526167	7,883920511	0,537609726	0,59080753
SLC30A7	-0,063560059	5,904108961	0,538364148	0,591541271
SERPINF1	0,185713935	7,833831431	0,539553785	0,592752902
PSMB10	-0,118608754	6,303781373	0,5411142	0,594371412
INPP1	-0,149883691	5,197312531	0,542468474	0,595763009
MYSM1	0,099667096	2,750856707	0,543680134	0,596997558
ARFRP1	-0,081503162	5,312672565	0,545932713	0,599374524
GPATCH8	-0,06859066	6,260162716	0,546785735	0,600193635
TMUB2	0,05917288	5,781757207	0,546854825	0,600193635
SH2B1	-0,081060903	5,483001733	0,548836137	0,602271262
ATP11C	0,127198155	5,117233203	0,548992435	0,602345843
AGR2	0,303777088	7,312975731	0,549737269	0,60306603
ECE1	-0,111614849	8,401635224	0,550568784	0,603826462
SEMA4A	0,109669111	6,453697332	0,550607559	0,603826462
SART1	0,078955138	6,652344995	0,550787362	0,603926519
EIF4ENIF1	0,063380517	5,386677427	0,551138742	0,604214643
RASSF4	-0,135909571	5,604368839	0,551742668	0,604779497
CETN2	0,093499227	6,483885032	0,551858823	0,604809598
IKBKB	0,163679397	6,264560862	0,552284782	0,605179163
ARL3	0,10634835	5,341226068	0,552881442	0,605735629
PIGG	0,098656108	5,390739177	0,553694308	0,606488138
IRAK1BP1	0,135852489	2,051097951	0,553746172	0,606488138
STXBP5	-0,091479488	4,746985658	0,554867654	0,607618842
JOSD1	0,080480915	7,382625767	0,556335716	0,609128653
SSBP2	0,142758694	4,909758278	0,55675351	0,60948823
CPD	0,128868307	8,018383782	0,557011761	0,609673065
ZMYM4	0,065414097	6,406048282	0,56052244	0,613417191
PRKD2	-0,073429565	6,434399187	0,560857134	0,613613202
TMEM176B	-0,133874477	6,906957443	0,560881521	0,613613202
IRF9	-0,099295201	5,814642855	0,561336849	0,614012828
ATF2	0,071384324	5,138159543	0,561861598	0,614488249
B3GNT2	-0,09967594	6,304025261	0,562814881	0,615432116
SPSB1	0,11401547	6,366560104	0,563192954	0,615746794
GGA1	-0,066142017	6,115020453	0,563399642	0,615811598
FAM120C	-0,092968667	4,005912128	0,563432844	0,615811598
DNAJB9	-0,106860859	6,364704995	0,564690161	0,617086893
MED23	-0,060490038	5,34145095	0,565058572	0,617390547
TMEM97	0,128960013	6,158367482	0,565919297	0,618231926
ALKBH5	-0,067634303	7,166562519	0,566592025	0,618867695
RPL15	0,099180065	10,44516047	0,566928606	0,619136157
ANAPC10	0,071216113	3,707364692	0,567648541	0,619823122

TAB1	0,064362544	5,510252022	0,568178102	0,620208639
STARD3	0,113131747	6,082723992	0,568183512	0,620208639
MGST2	0,109560003	5,616657584	0,568567992	0,620528991
MAN1A2	0,090368009	5,641213132	0,56872696	0,620603159
POLI	-0,0811649	4,509490588	0,569268078	0,621094244
WDR11	-0,066747069	6,226645885	0,569589077	0,621345051
BTBD1	0,055466991	6,652859837	0,570234245	0,621949346
CDKN1B	0,095058269	6,478309142	0,571867527	0,623631004
LRIG2	0,077210761	3,743003276	0,572780561	0,624526806
CLIP4	-0,112697079	6,076154815	0,573119794	0,624796783
ENC1	0,153597032	6,200608867	0,574103881	0,62576956
TNRC18	-0,085053843	7,416610482	0,574356589	0,625944955
CCDC8	0,247270923	4,529477874	0,574848319	0,626380742
CHMP4B	0,069661478	7,841501991	0,575631356	0,627133763
EBAG9	-0,06840157	5,315562316	0,576081499	0,62752392
ALG14	0,106751882	3,721006814	0,576208797	0,627562337
AGPS	-0,071630443	6,461759052	0,576761579	0,62806407
GRB10	0,150196931	6,22264905	0,577327832	0,628580311
ATP5S	0,093976133	3,297804147	0,577871302	0,629071585
EXTL3	0,103387632	6,431027312	0,578384552	0,62952981
R3HDM2	-0,070808894	6,483351234	0,579659798	0,630817134
FOXN2	0,090622607	5,540322273	0,579810929	0,63088092
AMD1	-0,072524055	7,375099584	0,58191275	0,633066854
LRRCC1	0,127248043	4,787422953	0,583223931	0,634311074
RNF4	0,063704735	6,585496727	0,583242474	0,634311074
FAM120A	0,069119817	8,213162914	0,583573488	0,634569863
HYMAI	0,0943781	0,317002206	0,583973979	0,634904108
EDF1	0,06351	7,658877039	0,584430204	0,63529883
GALK2	-0,058361766	4,752260575	0,584935635	0,635746908
PDDC1	-0,073682491	5,83708891	0,585808218	0,636593823
ZDHHC2	-0,125793837	6,137980948	0,586643141	0,63739955
RBM27	-0,052594427	5,795145644	0,587166382	0,637866427
TESK1	0,105222743	5,792224018	0,587644202	0,638283819
TEAD4	0,115171842	4,815175858	0,588139411	0,638719962
PKN2	0,064128257	6,372752859	0,588266193	0,638755919
RABGEF1	0,070988542	6,012578652	0,588522135	0,638932087
TRAFD1	0,081519371	6,422825026	0,589993546	0,640427569
CLTB	0,107743043	6,570913944	0,590745289	0,641141513
ARFGAP3	0,073664492	6,583652989	0,592601545	0,643053777
GNB2	0,070511384	8,277720738	0,593098688	0,643490843
SSBP3	0,123593131	5,625037771	0,593898229	0,644255811
GNL1	0,06225225	6,526388076	0,594620935	0,644937197
RNF167	-0,056216558	7,027703528	0,594928912	0,645168615
KRT18	-0,111685368	9,08537134	0,597265597	0,647526144
CRTC2	0,05707954	6,168070578	0,597292777	0,647526144
PYGB	0,11930959	7,962369761	0,598946384	0,649215609
LARP4	0,054940325	6,437630722	0,600879623	0,651207589
RANBP3	-0,057812989	6,275070644	0,601521421	0,651799551
SAFB	0,073935752	6,993401835	0,601931527	0,652140305
ZNF277	0,076674581	5,243560341	0,603658069	0,653906969

CCDC104	-0,07373694	5,550951964	0,604125619	0,654309497
RPL3	0,073078474	11,46768419	0,605805798	0,65602505
ZNF688	0,086308973	3,562455436	0,60655908	0,656736483
MYL12B	-0,076236694	9,44824087	0,607741458	0,657912209
DCTD	-0,057614449	6,607493973	0,608856924	0,659015138
SH3PXD2B	0,114515708	6,645178458	0,609114834	0,659189661
RELT	-0,077213828	4,317781487	0,609745589	0,659767561
ZNF180	0,077312686	3,504758463	0,611201853	0,661238368
ACIN1	-0,055929544	7,238090605	0,611809551	0,66179082
SMAD2	0,073360005	7,198058253	0,612305523	0,662222262
LENG9	-0,075507464	2,867984628	0,6124831	0,66230927
SREBF2	-0,076502531	8,4666519	0,61261793	0,662350034
TSG101	0,068030633	6,612625594	0,612744301	0,662381641
VPS54	0,075116031	5,840497176	0,613605746	0,663207732
MUT	0,066515082	5,789485716	0,614182402	0,6637258
FAM109B	0,108991274	4,752103701	0,615355889	0,664888576
ABCC3	0,202000514	6,100673664	0,615492591	0,664930922
SQSTM1	0,102150537	9,354123823	0,616120974	0,665504344
PTGR2	0,082904663	3,823026586	0,616773872	0,666104059
PYCARD	0,116004883	5,365566935	0,617305545	0,666572686
RAP1B	0,094026691	8,173740855	0,617698605	0,666891511
C16orf72	-0,051988894	6,307192578	0,618081778	0,667199564
LMBRD2	0,099819767	3,711327428	0,61928874	0,668396632
MSH3	-0,04732474	4,696716263	0,62013846	0,669207812
GPR137B	0,089355675	5,49077857	0,620572795	0,669570552
CSTF2T	-0,05597276	5,848208924	0,622008984	0,671013964
SLFN13	0,149929118	4,888137994	0,622766854	0,671725273
AP3M1	0,05413923	6,513634827	0,622932882	0,67179809
KTN1	-0,070369578	8,27709471	0,623257805	0,672042216
NRIP1	0,127816116	6,652879594	0,623711524	0,672425119
SPNS1	-0,055016325	6,226391379	0,626276522	0,675083719
HTRA1	-0,107341889	7,144061627	0,62662972	0,675357683
PELI2	-0,107878459	4,887017453	0,626783491	0,67541666
TPM4	-0,069098672	9,958953438	0,627093954	0,675644442
RRAS2	0,110543725	5,90837297	0,630210929	0,67889547
RDH11	-0,061234736	6,957732384	0,631087535	0,679732412
MAP2K1	0,05673838	6,8207761	0,631781016	0,680371881
RNF170	0,068497185	5,120796613	0,631959726	0,680456872
RNF141	-0,066342536	6,52588293	0,632606027	0,681045232
DNAJC8	0,060172175	7,118575478	0,633702839	0,682118336
DCAF10	0,064335454	5,929556175	0,634249283	0,682598779
AQR	-0,051395919	5,978362693	0,63505907	0,683362443
HIGD1A	-0,078793472	7,128590295	0,635558375	0,68379182
PROSC	0,103484955	6,540635354	0,635852294	0,684000125
KIAA1704	0,068362326	4,447717761	0,636408877	0,684490873
PSME1	-0,071228625	8,06283292	0,637948701	0,686006017
FKBP2	0,072464578	5,985354098	0,638018794	0,686006017
GATAD2B	-0,056957091	6,13897643	0,63857205	0,686492639
FLOT2	-0,063013074	7,90685138	0,638840727	0,686673222
HPS6	-0,052035483	5,003761113	0,639582704	0,687362405

CCDC85B	-0,090285467	4,28279193	0,640055398	0,687762017
ISG20	-0,101490318	5,125729973	0,640256374	0,687869578
PLD3	-0,086305843	9,198120161	0,641047739	0,6886113
IFI6	-0,141567303	7,833543442	0,641191723	0,688657483
GBE1	-0,069317389	5,749247988	0,641672521	0,689009278
CDK2AP2	-0,076628865	6,24376925	0,64182861	0,689009278
SEC22B	-0,055642874	6,584551692	0,641896369	0,689009278
ETNK1	0,086602257	6,504313797	0,641923441	0,689009278
MGAT4B	0,066374877	7,078480616	0,642341252	0,689349228
TMEM67	-0,06615832	4,023387274	0,642518983	0,689431462
CTNNBL1	-0,049832227	6,124574421	0,642737679	0,68955762
ADAR	-0,055907616	9,090131613	0,643642695	0,690419936
LOC93622	0,07883483	4,070363543	0,644908954	0,691669417
GPKOW	-0,041481875	5,023346459	0,645269877	0,691947679
PTPN14	0,097239246	4,469579275	0,647030793	0,693726883
ZFAND2B	-0,068086154	4,762917841	0,647736733	0,694293585
RPL26	-0,068768319	9,537501234	0,647790641	0,694293585
CEP68	-0,062399794	5,660221065	0,647864802	0,694293585
PRPF4B	-0,04353953	6,570164019	0,648035789	0,6943677
ADAT1	0,066482022	4,349214818	0,648620609	0,694885143
RNPEP	-0,048911262	6,954473364	0,648763498	0,694929044
ATP6V1D	-0,055581976	6,243378178	0,648877451	0,694941941
PHF16	0,071586484	4,475899872	0,650088774	0,696129923
FDFT1	0,093609376	8,021106168	0,651270358	0,697216813
PAF1	0,085286456	6,68808591	0,65130827	0,697216813
KCTD6	-0,063920778	2,914611391	0,652332228	0,698203338
FAM195B	0,10743983	6,344622851	0,652754451	0,698545606
RBBP9	0,067752043	5,723629562	0,653337659	0,699060018
ZNF805	-0,051830969	4,593671703	0,653774696	0,699417894
FUNDC2	0,088530618	6,836979558	0,654273679	0,699841917
ACTR2	0,05302344	9,026566489	0,654515768	0,699991063
DIS3	0,055667401	6,268479348	0,654951342	0,70034706
PLEKHM1P	0,075451425	3,876421677	0,655156	0,700456062
TRIP4	-0,043467614	5,187871253	0,656873168	0,702181868
DHODH	0,053597071	3,859649559	0,657175319	0,702394749
HTATSF1	0,062509162	6,635515283	0,658261429	0,703445335
NCKIPSD	-0,050698687	4,905771049	0,658508857	0,703490427
RAB32	-0,08072823	5,555493541	0,658509957	0,703490427
KLHDC2	-0,058762177	5,843545988	0,659171664	0,704083381
MARVELD1	-0,071990377	6,39371659	0,659271504	0,704083381
C11orf75	-0,072681322	4,594351738	0,659947565	0,704695027
SLC25A16	-0,062069964	4,119709989	0,66060108	0,705267168
VCAM1	-0,130216703	6,082005644	0,660690229	0,705267168
VTI1B	-0,051078328	6,21778986	0,661206203	0,705681691
MED9	0,054935135	4,462965936	0,661287839	0,705681691
FGFR1OP2	-0,050945508	5,459968406	0,661389014	0,705681691
CHMP2A	0,059119041	6,980155484	0,661527207	0,705712798
ARF4	-0,060322601	8,205599796	0,661625153	0,705712798
NIPSNAP3A	-0,076188647	5,283785049	0,66223813	0,706256148
BMI1	0,063425992	6,489098055	0,662643017	0,706577441

EFNA1	0,108575967	7,482440267	0,663128095	0,706984128
EIF3C	0,068345293	7,575057177	0,663495342	0,707265083
DOLK	0,06225411	4,993414137	0,664401562	0,708120389
FAM50A	0,061664814	6,39525796	0,664591111	0,708211718
UCK1	-0,044663833	5,313082561	0,666366155	0,709992314
PARN	0,04518372	6,301738875	0,666477892	0,709995886
NHLRC2	0,064426912	3,601229319	0,666577748	0,709995886
PPIG	0,047862231	6,243554014	0,667159983	0,710505064
TOMM7	-0,062918177	7,448364211	0,669355664	0,712732085
IRF2BP2	-0,042356965	8,095908189	0,670285069	0,713610287
NSUN4	0,045614707	5,305848533	0,670786525	0,714032675
ACAD11	0,08514548	4,506218939	0,673122964	0,716387947
OGG1	-0,05308046	4,123001256	0,673209263	0,716387947
GNG12	-0,070034784	7,602409154	0,673405812	0,716485291
SLC39A1	-0,045911073	7,734465059	0,674400241	0,71741225
NF2	0,055795573	6,335865388	0,674487452	0,71741225
IFT122	-0,063119228	5,444060595	0,675017567	0,717864128
PLEKHA3	-0,046479718	4,811988554	0,676727767	0,71957066
BAP1	0,054119857	6,843970881	0,678462347	0,721302579
SF3B2	0,042160127	8,3290835	0,679936919	0,722757576
PACS1	-0,053672267	7,221656963	0,680063784	0,72277976
INPP4B	-0,10275899	3,672543321	0,681024688	0,723688227
DYNLRB1	0,05247717	7,019354029	0,681257106	0,723702822
RNPC3	-0,050390104	2,836203433	0,681322359	0,723702822
LENG8	0,091568437	6,793408432	0,681356814	0,723702822
SNX4	0,055119007	6,014385091	0,681743816	0,724001103
PLEKHA1	0,076924188	6,887750017	0,682221287	0,724395353
CHST15	-0,074848466	6,243219229	0,682611613	0,724696963
DRAM2	-0,051687504	6,092265012	0,683198085	0,725206685
ZMYM5	-0,043130413	4,190001279	0,683451357	0,725362615
SIN3B	0,057779448	5,894320705	0,683867108	0,725690914
SHB	-0,072022296	5,685222625	0,684185712	0,725916037
PURB	0,05239915	6,987839842	0,684418122	0,726049653
MEAF6	-0,050513571	6,384419501	0,685002329	0,726556366
UAP1	0,06564377	6,524397715	0,68525747	0,726713949
ANXA2	0,08162484	11,09047533	0,686488416	0,727906159
ZNF780A	-0,059400744	4,086717416	0,686686839	0,728003351
H2AFV	0,04693424	8,029533893	0,689001739	0,730343985
TRPC4AP	-0,037644896	7,268393044	0,689137274	0,730374117
PITPNM1	0,079974085	5,839058693	0,689263481	0,730394355
SP140L	0,078247686	4,834558158	0,691466525	0,732615015
TAF1C	0,063988292	5,338949263	0,693416426	0,734518863
SKAP1	0,146139653	3,745593391	0,693478874	0,734518863
HMOX2	0,05758803	6,13239334	0,696853842	0,737978933
URM1	0,050699877	6,135338695	0,698425879	0,739528893
LUM	-0,106766467	10,09458359	0,699357907	0,740400802
SRPR	-0,041278446	8,173197587	0,699618444	0,740561654
ADRBK1	0,048856695	7,357639705	0,700200711	0,74106296
HACE1	0,091662633	4,309270607	0,700487387	0,741251318
PEG10	-0,139401555	7,100801115	0,700934063	0,741608903

EDC4	-0,043348661	6,286665335	0,70148654	0,742078299
PIBF1	-0,045631413	4,633551392	0,702435223	0,742966618
ANXA2P2	0,077527522	6,780226043	0,703062472	0,743514733
IRF5	0,094513502	5,029322408	0,704508603	0,744928542
SCARB1	0,077690616	6,118557092	0,706764029	0,747197506
YIPF3	-0,035759816	7,358052684	0,709460291	0,749931751
TAF13	0,062527588	4,131490795	0,711784992	0,752272452
PCOLCE	-0,088140059	6,608526585	0,712398226	0,752678004
CHMP1B	0,056188637	7,323254941	0,712498747	0,752678004
PRPF38A	0,045211864	5,900737475	0,712595983	0,752678004
AGAP1	0,071560129	4,999315539	0,712618677	0,752678004
TAF15	-0,055844252	7,274361428	0,712720615	0,752678004
RFX5	-0,051188865	6,220576604	0,713593716	0,753452373
ACSL3	0,053650445	7,136254127	0,713780827	0,753452373
EIF4G2	0,044544101	10,70522719	0,713785354	0,753452373
TP53BP1	0,051116621	6,038685176	0,714783546	0,754389258
CLOCK	-0,066835781	4,605634553	0,71714153	0,75676077
SFMBT1	-0,047824118	4,045077725	0,720494173	0,760180994
UPF3A	-0,043992713	5,271285053	0,721003679	0,76060088
RPP14	-0,02602411	4,915670613	0,721557838	0,761067734
SPSB2	0,071329952	3,850825851	0,721755684	0,761158677
RNF40	-0,042085688	7,116798681	0,722543726	0,761871914
C18orf8	-0,044507189	5,371749318	0,724783565	0,764115512
ADIPOR2	-0,044134934	7,495373583	0,724911278	0,764132015
LGALS1	0,112393065	8,56979316	0,726461551	0,765647808
SUMF2	0,073607896	8,031231428	0,726961501	0,766056324
ZNF187	0,053277751	4,149813054	0,727469576	0,766473275
C11orf58	0,047304378	7,904588759	0,727625083	0,766518684
GGCX	-0,037504166	5,997479403	0,728110852	0,76691194
IMPAD1	0,051985509	7,505560286	0,72849527	0,76719834
VPS4A	-0,031137948	7,013241171	0,729112359	0,767528511
HSD3B7	-0,059474172	5,168700302	0,729171757	0,767528511
CCNJ	0,060952583	3,784109963	0,729207844	0,767528511
TTC21B	-0,041398699	4,795309976	0,729332895	0,767528511
ZNF595	0,102592723	3,729017927	0,729371572	0,767528511
FASTKD5	0,052873271	5,034373996	0,73001423	0,768086257
PDCD4	-0,045777502	6,761053155	0,730167573	0,768129077
DOPEY2	-0,054118043	5,418902397	0,731751219	0,769676317
NGLY1	-0,031757083	5,055614459	0,733380193	0,771270749
TNFAIP8	0,059545503	5,322588934	0,733705218	0,771493583
RIC8A	0,035200435	7,013490633	0,734329206	0,772030659
RRAGC	0,049274978	5,176197377	0,734729581	0,772309344
CCDC97	0,049284258	6,116492405	0,734820798	0,772309344
POGK	-0,040409017	6,851499493	0,735901196	0,773325668
ATP6AP2	0,045344883	8,058006927	0,736129158	0,773446029
TAF8	-0,033385426	5,432095303	0,736579723	0,773800205
MET	0,094388333	7,477903194	0,737903803	0,775071786
WASF2	-0,049898923	8,46043091	0,739381453	0,776504255
ZNF823	0,058276044	2,679179631	0,739570661	0,77658336
P4HA2	-0,058156508	6,718721899	0,740149571	0,777071582

SMG1	0,049949112	7,007313265	0,742968082	0,779910614
NPAT	0,040322746	5,074230803	0,743477501	0,78032524
LETMD1	-0,034514958	5,726306319	0,744179195	0,78094151
FAM108A1	-0,041644556	5,752659269	0,746371687	0,783121793
ELOVL5	-0,037927524	7,551576707	0,746688765	0,783333953
PCMTD2	0,058110044	6,095527137	0,747317668	0,783873124
VEGFA	0,071958782	7,903656772	0,74811145	0,784585048
IFI27	0,155642114	8,494518909	0,749458074	0,785876458
TRNT1	0,042918348	4,18368294	0,750114506	0,786443853
RNF31	-0,037742602	6,671512462	0,750313826	0,786531895
ERP29	-0,037795266	7,904349207	0,750689106	0,786804337
SGSM3	0,04183379	6,116022015	0,752450125	0,788528877
RAB12	0,042014967	6,265128894	0,752767873	0,788740646
GLUD1	0,054933332	8,098414405	0,753633856	0,789526696
CLIP3	-0,071960432	4,673817215	0,754203267	0,790001855
USP48	0,036048901	6,140918735	0,754521051	0,790213339
GZF1	-0,031523617	4,844454647	0,756344702	0,792001616
SS18	0,055094863	6,884225432	0,756854305	0,792413559
IREB2	0,037711112	6,469846905	0,7578696	0,793354744
STAG1	0,047379687	5,804600075	0,758891298	0,794302342
PPL	-0,07388235	7,741076113	0,759623215	0,794946394
CRAMP1L	-0,039709754	5,506347038	0,759875085	0,79508407
HIAT1	-0,031189406	6,719495628	0,759987971	0,79508407
ZNF641	-0,040304348	3,727410679	0,760656203	0,79566109
TAF7	0,047387956	7,374704806	0,761000038	0,795833538
C5orf44	0,055310114	5,019239417	0,76105448	0,795833538
RPS6KA5	0,067787666	3,93731606	0,761217891	0,795882367
RSPRY1	0,040557287	4,857213686	0,761420079	0,795910522
HIRA	0,047384121	5,905633185	0,761496324	0,795910522
STAG2	0,037537912	7,405522996	0,761594978	0,795910522
PDLIM5	-0,037642715	7,099970645	0,763023037	0,797280737
EXOC5	-0,031668379	6,626746248	0,763518075	0,79767577
RRAGB	-0,045259541	4,293034246	0,766414046	0,800578642
PPT1	-0,049970677	8,666427643	0,767282494	0,801363046
RALB	0,070134845	7,230806658	0,769742213	0,803808906
SETD1B	0,048875486	5,752853495	0,770019983	0,803975848
MED29	-0,040957048	6,548304736	0,77262733	0,806574672
UBA3	-0,031673498	6,051986256	0,77301975	0,80686081
USP34	-0,041191534	7,416119752	0,774389999	0,808167341
ARHGAP23	-0,057042504	6,862179149	0,776355416	0,810094504
NKAP	0,031249832	4,790707686	0,777972644	0,811657812
DNASE2	-0,035096671	6,978127343	0,778952935	0,812556228
ADPGK	0,038408485	6,708438077	0,779744005	0,813257016
NOL9	0,043914883	4,020935586	0,77986893	0,81326292
VTI1A	0,025435748	4,84978241	0,7800799	0,813358538
NBN	0,041370704	6,547908185	0,780214212	0,813374211
NAPG	0,038062185	5,691552373	0,781271452	0,814351885
CBX5	0,058013071	7,361730704	0,781413399	0,814375358
ARFGEF2	0,039895658	6,557138524	0,781578083	0,814422516
PSMD6	0,025750015	6,142124213	0,783127352	0,81591221

C5orf42	0,055523143	4,78682303	0,783686502	0,816370036
ATP6V1H	-0,028737507	6,244323004	0,78481658	0,817422371
PPP2R4	0,033693056	7,674453165	0,785519592	0,818029642
RICTOR	-0,031955919	5,886719746	0,786134827	0,818545333
NUAK1	-0,059339518	6,029813843	0,788702338	0,821093319
FBRS	0,040662948	6,616346435	0,789667988	0,821973135
ARRDC1	0,04212563	5,566044128	0,790660565	0,822880707
ARPC5	0,043602123	8,514206642	0,792535745	0,824706432
MIPEP	0,04205339	4,640194493	0,793224296	0,82529699
ZBTB44	-0,030992071	6,452098485	0,793706542	0,825672755
HSDL1	-0,033383241	5,181709064	0,795707072	0,827627596
CD99	0,051384457	8,299230209	0,796262806	0,828051244
OSGEP	0,042749287	4,880773014	0,796357246	0,828051244
TYK2	0,051108974	6,307509963	0,798535547	0,83008925
OLFML2B	0,087329615	5,633874746	0,798583414	0,83008925
ANXA1	0,090169637	9,805111381	0,798682442	0,83008925
COL6A1	-0,05991329	8,912119268	0,799512352	0,830825165
GOLGA1	-0,025596746	5,009958669	0,800587953	0,831751893
CAPRIN2	-0,036823489	4,253259439	0,800769372	0,831751893
LRRC37B	0,04453751	3,151868396	0,80077008	0,831751893
UQCRB	0,038791571	7,515489753	0,802100139	0,833006526
WDR33	-0,019458234	6,465391165	0,803980594	0,834832293
RPRD2	-0,02909322	6,527572703	0,804751805	0,83550587
ARGLU1	-0,038227165	6,43571513	0,806374147	0,837062765
SOCS7	-0,039532658	2,469938482	0,806563665	0,83713206
HNRNPH3	-0,023849786	7,095383853	0,807816077	0,838304342
KCTD17	0,047565185	4,302784132	0,808077835	0,838448381
CNN2	-0,033876995	8,452675086	0,808714129	0,838980929
CARKD	-0,029624744	5,689745104	0,809485976	0,83965392
RNASET2	-0,037220258	6,090580549	0,809669429	0,839716478
SSBP4	0,043171969	5,184398676	0,81048549	0,840435
ZFAND3	-0,022195905	6,774733476	0,810758568	0,840590341
LOC728190	0,030061999	5,031449921	0,813486492	0,843290421
TOR1A	0,026657345	6,050191112	0,814698056	0,844418003
EFTUD1	-0,027990987	5,048893897	0,816088656	0,845730782
PLXNB1	-0,03354201	6,729358224	0,819487346	0,849123873
KDM4B	0,038868758	5,721435956	0,82021064	0,849744205
VEZT	0,024018525	6,629459786	0,823053289	0,852559681
GTF2F1	0,027817606	6,735627467	0,823889621	0,853296374
KDM5A	0,034761717	6,788323955	0,82422865	0,853517868
ZNF625	0,064392488	0,835049902	0,8261781	0,85540669
AMFR	-0,023869913	7,399902207	0,826997001	0,856124571
DHDDS	-0,023752054	5,567801605	0,82718711	0,856191393
NIPBL	-0,028570026	6,900165533	0,828490439	0,857410275
PKNOX1	-0,015451092	4,948535149	0,828994632	0,857645878
PDGFC	0,068153158	5,530157863	0,829040194	0,857645878
SENP7	0,036216476	4,50701929	0,829095414	0,857645878
POU2F2	-0,044911453	3,257787336	0,829749219	0,858192011
TRRAP	-0,030315677	6,757603339	0,830117223	0,858442424
FMR1	-0,020670535	6,191944134	0,831724252	0,85997387

CLSTN3	0,047362559	6,550035266	0,831886594	0,860011322
XBP1	-0,035763906	8,923823937	0,832516045	0,860409812
MICB	0,066389527	3,903798766	0,832540894	0,860409812
AIM1	0,059116169	6,821553109	0,832650586	0,860409812
MEPCE	-0,023011243	6,329479784	0,834815382	0,862516074
HAUS4	0,035952062	6,116689266	0,837410419	0,865066148
CFB	-0,046368583	7,280370504	0,838261991	0,86581468
BRD3	-0,026738448	5,775617684	0,838563126	0,865994541
ASNSD1	0,027597338	5,987944675	0,838790711	0,866098403
MAD1L1	-0,028145481	5,278882292	0,840322958	0,867549167
ZFAND6	0,034660502	6,691124485	0,840822734	0,867933731
CLK3	-0,019149061	5,689353594	0,841209692	0,868085982
ZDBF2	-0,038777491	4,035262135	0,841224837	0,868085982
COG7	-0,01646356	5,127540586	0,841683279	0,868427641
SMAD5	0,027693578	6,547998629	0,842166715	0,868794982
ALMS1	0,04305569	5,438695736	0,842713348	0,869227397
MTOR	-0,023771675	6,570844407	0,843855707	0,870274057
NCSTN	0,029685912	7,919110618	0,84455506	0,870863595
OSBPL2	0,024066308	6,164633039	0,844771223	0,870954788
SUV420H1	-0,021386931	5,935161198	0,845291067	0,871359
SEC11A	-0,01959886	7,351989098	0,846201534	0,872165698
HERC4	0,020460344	5,80788152	0,847501212	0,873373245
RPS23	0,035667099	9,787102417	0,849082645	0,87487074
ELF4	0,033019972	6,360016936	0,849229514	0,874889871
DNAJC5	0,031684597	7,298623542	0,850143874	0,875699558
GALNT11	-0,02790899	6,261210491	0,850288447	0,875716194
MVD	0,04603122	5,621168952	0,850634723	0,875940528
STC1	-0,039305209	5,893155045	0,850853312	0,876033329
ZNF142	-0,022260764	5,038213363	0,851386535	0,876449997
BCL10	0,031270325	5,391227178	0,852415611	0,877376913
PMEPA1	0,08333424	6,919290516	0,853366648	0,878223238
CTBP1	-0,022478895	7,174887727	0,855250238	0,880028878
ARL5A	0,025765916	6,340579767	0,85554339	0,880124046
MRPS31	-0,01901747	4,21562481	0,855600866	0,880124046
PICK1	-0,021089496	4,799791771	0,855861937	0,880259812
EPS8	-0,035200529	7,101732341	0,857470283	0,881781008
CTCF	0,018329973	6,486628936	0,857643299	0,881825943
BAG3	-0,024772518	7,140092016	0,858895181	0,882979985
B4GALT1	0,041523949	8,599380093	0,859103738	0,883061258
EAPP	0,026944116	5,726593503	0,860116391	0,883968903
INVS	0,02879986	4,126164309	0,860387625	0,884114409
NANOG	-0,022633264	0,811501723	0,861573875	0,885199978
ZMIZ2	0,033345289	6,970392156	0,862310375	0,885823207
PLXNB2	0,038904393	9,003314584	0,863728002	0,887145843
MAPK14	-0,015976588	6,773514017	0,864460668	0,887764653
ARL5B	0,033475167	4,760575127	0,86641171	0,889615981
TRA2A	0,023374263	6,266589862	0,866524321	0,889615981
ZCCHC11	0,022555482	5,442024238	0,867623323	0,890610182
APOOL	-0,019108562	3,619186702	0,868059568	0,89092387
MDM2	0,026027198	7,316345803	0,868647036	0,891392647

SSU72	0,020185146	7,323394421	0,869992374	0,89263888
AP4S1	-0,020982158	2,76937555	0,871222681	0,893766732
OGFRL1	0,038038611	4,798115792	0,871929106	0,894356885
TNIP2	0,024467735	5,555765172	0,87212737	0,894425708
CDC42EP4	-0,026650626	6,892581088	0,872591641	0,894767278
CHI3L1	-0,031136472	7,108645535	0,872846896	0,89489445
OSTF1	-0,020882861	6,640510322	0,87388944	0,895828637
LYRM1	-0,02306801	5,36164462	0,874178229	0,89595127
WDR47	0,034476931	5,127108439	0,87431602	0,89595127
NANS	-0,017341806	6,002383923	0,874479428	0,89595127
TNFRSF12A	-0,029331861	6,214465349	0,874640081	0,89595127
DDX20	0,019202218	4,715066233	0,874666022	0,89595127
ZNF782	-0,020711849	1,607909204	0,875065873	0,896226222
ZNF830	-0,014604972	4,317794813	0,875556202	0,896471051
OCIAD1	0,025784456	7,169365023	0,875567854	0,896471051
RNF139	0,022325785	6,130724915	0,877267405	0,89807633
CCDC88C	0,038248443	6,028010654	0,881700035	0,902478616
FZD1	-0,024592904	6,253008488	0,881902862	0,902550745
LCORL	0,023609101	2,767448483	0,883230502	0,903773829
PTPRK	-0,018245575	7,153211806	0,883686721	0,9040109
PPTC7	-0,016948073	5,684570856	0,883727329	0,9040109
HDAC4	-0,020367993	4,933721898	0,8838892	0,904040866
KBTBD7	-0,017224921	3,26288091	0,884520966	0,90455136
STX18	-0,01420396	5,093003156	0,884815882	0,904717274
RING1	-0,013119024	5,892977075	0,88614894	0,905944471
EEF2	0,027248951	11,46177076	0,889023726	0,908747233
FBXO11	-0,013226517	6,632728854	0,889628033	0,909228651
HCFC1R1	-0,016037902	5,765399089	0,891275294	0,910775697
CARS2	-0,013149261	5,497846827	0,891497017	0,910865772
CIC	-0,018121948	6,769005483	0,892370173	0,911621305
IFITM1	-0,021420145	7,837937007	0,892596422	0,911715848
SLC30A5	-0,011971541	6,181167672	0,89328096	0,912278399
ARIH1	-0,011531376	6,84834755	0,893582485	0,912387943
AP3S2	-0,011785665	6,558196886	0,893655825	0,912387943
WFDC2	-0,020897494	7,059709475	0,895124146	0,913750232
YTHDF3	-0,012338871	6,939450328	0,895706956	0,914097132
ZCCHC10	0,024493741	4,573727395	0,895732077	0,914097132
COG3	-0,01281187	5,279713122	0,895893037	0,914124588
UBQLN2	-0,013430888	6,424558196	0,896397025	0,914501992
M6PR	0,019036714	7,672884254	0,898659788	0,916666986
SIN3A	-0,012006798	6,919587074	0,898788014	0,916666986
SACS	-0,016780118	4,513530868	0,899393279	0,917147117
FLJ45340	0,030105946	5,908357123	0,899728626	0,917351899
MYO5A	-0,016579201	6,306225593	0,902046247	0,91957742
CYBA	0,042912995	7,343496126	0,902608322	0,920012877
MLX	0,017220407	6,537325016	0,903828107	0,921118496
PCK2	-0,015409334	5,97853246	0,904259993	0,921420934
LPP	0,028459642	5,083226335	0,904850937	0,921885334
TMC6	0,026104882	6,613181544	0,905646107	0,922557635
RFK	-0,016254018	5,333155046	0,90620859	0,922992737

EXOC4	-0,010266323	6,795600417	0,907338834	0,9240059
EXOSC10	0,014301089	6,304300744	0,909752064	0,92632512
SMU1	0,019044649	7,029021446	0,910189781	0,926632445
VAC14	-0,010197664	6,289874775	0,911873572	0,928208074
TBK1	-0,009326052	5,435433059	0,912719868	0,928930862
EXOC7	-0,008860853	7,533832248	0,915450408	0,93157086
CALM2	-0,012178024	9,746731665	0,916136242	0,932129667
SHPK	-0,010164444	4,631933669	0,916919978	0,932787906
TBCD	0,02232877	6,810024283	0,917919908	0,933461764
SCCPDH	-0,012793205	6,123227408	0,917939436	0,933461764
USO1	-0,010278593	7,139959652	0,917993047	0,933461764
DBI	-0,01172081	7,734353582	0,918585178	0,933868185
POLD4	0,01835349	6,419671519	0,918666635	0,933868185
CYB5B	-0,01054478	6,94020991	0,919469983	0,934545508
CRAT	0,030277655	5,609987589	0,921541276	0,936511171
NAA16	0,016066621	4,139825173	0,921793092	0,936627491
HADHB	-0,007745806	7,335862076	0,92195872	0,936656215
FIS1	-0,010612608	6,764036612	0,923656275	0,938241045
PNMA1	-0,010519757	5,637592467	0,923854491	0,938302618
COX7C	0,017632396	7,707762231	0,92409586	0,938407992
ZNF14	0,024228876	3,574260302	0,924388593	0,938565488
FUCA2	0,020718678	6,623094555	0,926046583	0,940108925
CTNND1	-0,009256544	9,238263419	0,926241676	0,940167012
AP4B1	-0,007534548	4,421073324	0,928320268	0,942136613
CNPY4	-0,009407793	4,180997634	0,928797065	0,942480236
TROVE2	0,009931665	6,076747774	0,929203878	0,942752751
TAF9B	0,018857463	5,449251828	0,93028258	0,943706771
MAP4K4	-0,008872129	7,694290813	0,930966293	0,944259876
SRI	0,020416854	7,018943609	0,931777286	0,944941897
POGZ	-0,007986055	6,681235211	0,932084036	0,945023538
TMEM70	0,018062933	5,374317663	0,932134963	0,945023538
TPM3	0,010820735	9,34737866	0,93266183	0,945417128
ZNF827	-0,008792609	3,494801873	0,933477621	0,946103433
MRPL14	0,019978247	6,483516227	0,934059743	0,94655274
NDUFA5	0,014350116	6,184155028	0,935914369	0,948152168
SMARCE1	0,010523353	7,437511549	0,935916152	0,948152168
APOE	0,046288188	8,455951013	0,937522217	0,949638146
THOC2	-0,006143829	6,331523381	0,939422284	0,95142144
FRS2	0,015372261	5,594711084	0,940898222	0,952656095
GXYLT2	-0,005292693	2,77868649	0,940927315	0,952656095
HEATR7A	0,016708437	5,463564126	0,941060486	0,952656095
SLCO3A1	-0,006309862	6,637253205	0,941284315	0,952741241
MTSS1L	0,022362395	6,419713517	0,943996861	0,955344998
SPPL2A	0,012637723	6,193932977	0,944399253	0,955610403
CFDP1	-0,005819303	5,919604849	0,944925454	0,956000989
PLEC	-0,004943428	9,265070615	0,945526823	0,956467498
CTBP2	-0,005155824	6,59971531	0,947213655	0,958031729
RUNX3	-0,004801158	5,458078566	0,947640886	0,958321696
LY6E	-0,003048289	8,705456689	0,949187295	0,959743204
SH3GLB2	-0,004751325	5,987240599	0,951100101	0,961534706

EFR3A	0,009988456	7,184540216	0,951493179	0,961789503
TP53I3	-0,003066799	5,158945029	0,952667889	0,962834198
LOC253039	-0,004399675	4,591961959	0,952863405	0,962889087
ZNF117	0,032260366	5,636297577	0,953411332	0,963300026
RAB4B	0,016154044	5,271275982	0,953722047	0,963384493
CD164	-0,004228591	8,859249859	0,953777491	0,963384493
MAX	0,006797032	6,431735431	0,954616895	0,963927724
LYRM7	0,010231446	4,928330665	0,954703365	0,963927724
TBC1D14	0,01252748	6,873353504	0,954739382	0,963927724
FPGS	-0,003983412	6,159603325	0,955258972	0,964309538
SURF1	0,012076289	5,786790172	0,956229112	0,965145991
KIAA0753	0,011324633	4,309811634	0,956809586	0,965588955
LPCAT2	0,016535259	5,804356701	0,957373288	0,966014864
SNX9	0,008864963	6,364517953	0,959250222	0,967765537
BLOC1S1	-0,003016436	6,157739598	0,959806101	0,968183107
UBN1	0,011601359	6,619208547	0,960857172	0,969099993
ZNF271	0,010100789	5,169858082	0,96194883	0,970057538
CHD4	0,009703418	8,474535854	0,963763351	0,971743648
RABAC1	-0,001802882	6,631124325	0,964247792	0,972088364
KIN	-0,002418738	3,763416548	0,964485735	0,972184512
PITPNC1	-1,68935E-05	4,945263157	0,964836135	0,972393971
RNF214	-0,002388237	4,509360763	0,965512833	0,972932172
SIGLEC10	0,020158956	4,566584099	0,966390594	0,973672792
LOC493754	-0,002242276	5,233599637	0,966555586	0,97369516
DIS3L	0,008324413	5,481182227	0,966769915	0,973767216
IWS1	-0,002092426	6,176808576	0,967443659	0,974301922
ARL6IP4	-0,00180713	7,150074373	0,968220608	0,974923691
ZNF462	0,023521943	4,817951622	0,968346995	0,974923691
FAM106A	-0,002149038	1,104445659	0,969512429	0,975952945
STX5	-0,001820111	6,087158955	0,969758772	0,976056836
NAP1L1	0,00728781	9,10711167	0,973166337	0,97934198
TIMP1	0,003089652	8,86599545	0,973788381	0,979823369
ARCN1	0,005874657	8,126203043	0,97459855	0,980493879
RAB6A	-0,000886679	8,053270029	0,975493724	0,981249699
GBA	-0,000524715	6,821396607	0,97592768	0,981541424
YIPF5	-0,000773014	6,333366697	0,976388964	0,981860544
SRP14	-0,000714575	7,977064727	0,977269104	0,982600711
POLR2C	-0,000266491	7,00694103	0,978254036	0,983446008
ERLEC1	0,007993743	7,167567239	0,978621424	0,983670326
TRAPPC6B	0,006613443	5,376846575	0,978816253	0,983721154
PRKAA1	0,000180031	6,850235114	0,979821374	0,984548156
DNAJC13	0,006869454	6,530085201	0,979927897	0,984548156
TPCN2	0,01208094	4,565792427	0,980382753	0,984860046
MAP4K2	0,000699255	5,33650222	0,980743091	0,985076909
SHPRH	0,00627614	4,479351623	0,98159544	0,985721429
TMEM214	0,005219625	7,310618284	0,981673886	0,985721429
LOC283922	0,002640234	2,526105618	0,982174641	0,986079045
SKP1	0,000919137	8,404892526	0,984470906	0,988238938
FLOT1	0,001588139	8,093091657	0,985232169	0,988776327
FAM100A	0,006379664	4,627738623	0,985296252	0,988776327

PML	0,003000188	7,245480683	0,986326606	0,989664674
NDRG3	0,005018385	6,11249561	0,986940908	0,990135362
CNOT4	0,000659378	5,00663668	0,988191429	0,991244094
MT1F	0,008737509	3,281918426	0,99035925	0,993167427
ZNF317	0,005148364	5,502675417	0,990506181	0,993167427
ACTR10	0,005081718	6,288241364	0,99054578	0,993167427
NAMPT	0,022468595	9,616661922	0,990867301	0,993343741
EIF4G3	0,002445697	6,481401408	0,99311134	0,995447042
FADS1	0,012810189	6,9014123	0,993624875	0,995815406
COMT	0,00385856	7,538763367	0,99451869	0,996557566
FAM175A	0,003780689	4,187999376	0,994666579	0,996557566
SOS1	0,002418535	6,421209939	0,994803835	0,996557566
SNX5	0,003187599	7,23393712	0,995375568	0,996983843
GNA13	0,003322918	7,531332968	0,995654836	0,997117099
EDEM3	0,002215733	6,280792318	0,996010804	0,997327117
UROD	0,003704202	6,1720869	0,996555305	0,99772583
AMIGO3	0,008175796	2,187473586	0,997310366	0,998217056
ELF3	0,016283273	8,39502298	0,997338731	0,998217056
ARL8B	0,002985914	7,508478382	0,997726007	0,998319659
AP3D1	0,003543626	7,725356857	0,997793038	0,998319659
PFDN5	0,006610587	8,527623863	0,997880451	0,998319659
UFC1	0,004760159	7,038000657	0,999052535	0,999286837
CPE	0,021428819	7,184802244	0,999140292	0,999286837
SNX3	0,004124787	7,867605061	0,999362278	0,999362278

Table S2_A: Lung adenocarcinoma (LUAD) ID from TCGA.

LUAD patients ID available on TCGA on 07 June 2016 and used for the analysis.

Tumor Samples	Normal Samples
TCGA.05.4244.01A.01R.1107.07	TCGA.38.4632.11A.01R.1755.07
TCGA.05.4249.01A.01R.1107.07	TCGA.44.5645.11A.01R.1628.07
TCGA.05.4250.01A.01R.1107.07	TCGA.44.6144.11A.01R.1755.07
TCGA.05.4397.01A.01R.1206.07	TCGA.44.6145.11A.01R.1858.07
TCGA.05.4398.01A.01R.1206.07	TCGA.44.6146.11A.01R.1858.07
TCGA.05.4403.01A.01R.1206.07	TCGA.44.6147.11A.01R.1858.07
TCGA.05.4417.01A.22R.1858.07	TCGA.44.6148.11A.01R.1858.07
TCGA.05.4418.01A.01R.1206.07	TCGA.44.6776.11A.01R.1858.07
TCGA.05.4420.01A.01R.1206.07	TCGA.44.6777.11A.01R.1858.07
TCGA.05.4422.01A.01R.1206.07	TCGA.44.6778.11A.01R.1858.07
TCGA.05.4424.01A.22R.1858.07	TCGA.49.4490.11A.01R.1858.07
TCGA.05.4426.01A.01R.1206.07	TCGA.49.4512.11A.01R.1858.07
TCGA.05.4427.01A.21R.1858.07	TCGA.49.6742.11A.01R.1858.07
TCGA.05.4430.01A.02R.1206.07	TCGA.49.6743.11A.01R.1858.07
TCGA.05.4432.01A.01R.1206.07	TCGA.49.6744.11A.01R.1858.07
TCGA.05.4434.01A.01R.1206.07	TCGA.49.6745.11A.01R.1858.07
TCGA.05.5420.01A.01R.1628.07	TCGA.50.5930.11A.01R.1755.07
TCGA.05.5423.01A.01R.1628.07	TCGA.50.5931.11A.01R.1858.07
TCGA.05.5425.01A.02R.1628.07	TCGA.50.5932.11A.01R.1755.07
TCGA.05.5428.01A.01R.1628.07	TCGA.50.5933.11A.01R.1755.07
TCGA.05.5429.01A.01R.1628.07	TCGA.50.5935.11A.01R.1858.07
TCGA.05.5715.01A.01R.1628.07	TCGA.55.6968.11A.01R.1949.07
TCGA.35.3615.01A.01R.0946.07	TCGA.55.6969.11A.01R.1949.07
TCGA.35.4122.01A.01R.1107.07	TCGA.55.6970.11A.01R.1949.07
TCGA.35.4123.01A.01R.1107.07	TCGA.55.6971.11A.01R.1949.07
TCGA.38.4625.01A.01R.1206.07	TCGA.55.6972.11A.01R.1949.07
TCGA.38.4626.01A.01R.1206.07	TCGA.55.6978.11A.01R.1949.07
TCGA.44.2655.01A.01R.0946.07	TCGA.55.6982.11A.01R.1949.07
TCGA.44.2656.01A.02R.0946.07	TCGA.55.6983.11A.01R.1949.07
TCGA.44.2659.01A.01R.0946.07	TCGA.55.6984.11A.01R.1949.07
TCGA.44.2661.01A.01R.1107.07	TCGA.55.6985.11A.01R.1949.07
TCGA.44.2662.01A.01R.0946.07	TCGA.91.6828.11A.01R.1858.07
TCGA.44.2665.01A.01R.0946.07	TCGA.91.6829.11A.01R.1858.07
TCGA.44.2668.01A.01R.0946.07	TCGA.91.6835.11A.01R.1858.07
TCGA.44.3398.01A.01R.1107.07	TCGA.91.6836.11A.01R.1858.07
TCGA.44.3918.01A.01R.1107.07	TCGA.91.6847.11A.01R.1949.07
TCGA.44.3919.01A.02R.1107.07	TCGA.91.6849.11A.01R.1949.07
TCGA.44.4112.01A.01R.1107.07	
TCGA.44.5643.01A.01R.1628.07	
TCGA.44.5645.01A.01R.1628.07	

TCGA.44.6146.01A.11R.1755.07	
TCGA.44.6147.01A.11R.1755.07	
TCGA.44.6148.01A.11R.1755.07	
TCGA.44.6775.01A.11R.1858.07	
TCGA.44.6776.01A.11R.1858.07	
TCGA.44.6777.01A.11R.1858.07	
TCGA.44.6778.01A.11R.1858.07	
TCGA.49.4488.01A.01R.1755.07	
TCGA.49.4505.01A.01R.1206.07	
TCGA.49.4507.01A.01R.1206.07	
TCGA.49.4512.01A.21R.1858.07	
TCGA.49.4514.01A.21R.1858.07	
TCGA.49.6742.01A.11R.1858.07	
TCGA.49.6743.01A.11R.1858.07	
TCGA.49.6744.01A.11R.1858.07	
TCGA.49.6745.01A.11R.1858.07	
TCGA.49.6761.01A.31R.1949.07	
TCGA.49.6767.01A.11R.1858.07	
TCGA.50.5044.01A.21R.1858.07	
TCGA.50.5049.01A.01R.1628.07	
TCGA.50.5051.01A.21R.1858.07	
TCGA.50.5072.01A.21R.1858.07	
TCGA.50.5931.01A.11R.1755.07	
TCGA.50.5932.01A.11R.1755.07	
TCGA.50.5935.01A.11R.1755.07	
TCGA.50.5941.01A.11R.1755.07	
TCGA.50.5942.01A.21R.1755.07	
TCGA.50.5944.01A.11R.1755.07	
TCGA.50.6590.01A.12R.1858.07	
TCGA.50.6591.01A.11R.1755.07	
TCGA.50.6592.01A.11R.1755.07	
TCGA.50.6593.01A.11R.1755.07	
TCGA.50.6594.01A.11R.1755.07	
TCGA.50.6595.01A.12R.1858.07	
TCGA.50.6597.01A.11R.1858.07	
TCGA.50.6673.01A.11R.1949.07	
TCGA.55.1592.01A.01R.0946.07	
TCGA.55.1594.01A.01R.0946.07	
TCGA.55.1595.01A.01R.0946.07	
TCGA.55.1596.01A.01R.0946.07	
TCGA.55.6543.01A.11R.1755.07	
TCGA.55.6968.01A.11R.1949.07	
TCGA.55.6969.01A.11R.1949.07	
TCGA.55.6970.01A.11R.1949.07	
TCGA.55.6971.01A.11R.1949.07	
TCGA.55.6979.01A.11R.1949.07	
TCGA.55.6982.01A.11R.1949.07	
TCGA.55.6984.01A.11R.1949.07	
TCGA.55.6985.01A.11R.1949.07	
TCGA.55.6986.01A.11R.1949.07	

TCGA.64.1676.01A.01R.0946.07	
TCGA.64.1678.01A.01R.0946.07	
TCGA.64.1680.01A.02R.0946.07	
TCGA.64.5774.01A.01R.1628.07	
TCGA.64.5775.01A.01R.1628.07	
TCGA.64.5778.01A.01R.1628.07	
TCGA.64.5779.01A.01R.1628.07	
TCGA.64.5781.01A.01R.1628.07	
TCGA.64.5815.01A.01R.1628.07	
TCGA.67.3770.01A.01R.0946.07	
TCGA.67.3771.01A.01R.0946.07	
TCGA.67.3772.01A.01R.0946.07	
TCGA.67.3773.01A.01R.0946.07	
TCGA.67.3774.01A.01R.0946.07	
TCGA.67.4679.01B.01R.1755.07	
TCGA.67.6216.01A.11R.1755.07	
TCGA.67.6217.01A.11R.1755.07	
TCGA.73.4658.01A.01R.1755.07	
TCGA.73.4662.01A.01R.1206.07	
TCGA.73.4670.01A.01R.1206.07	
TCGA.73.4675.01A.01R.1206.07	
TCGA.73.4676.01A.01R.1755.07	
TCGA.75.5122.01A.01R.1755.07	
TCGA.75.5146.01A.01R.1628.07	
TCGA.75.7025.01A.12R.1949.07	
TCGA.80.5607.01A.31R.1949.07	
TCGA.80.5611.01A.01R.1628.07	
TCGA.91.6829.01A.21R.1858.07	
TCGA.91.6830.01A.11R.1949.07	
TCGA.91.6831.01A.11R.1858.07	
TCGA.91.6835.01A.11R.1858.07	
TCGA.91.6836.01A.21R.1858.07	
TCGA.91.6840.01A.11R.1949.07	
TCGA.95.7039.01A.11R.1949.07	
TCGA.95.7043.01A.11R.1949.07	

**Table S2_B: Lung squamous cell carcinoma (LUSC) ID from TCGA.**<br>LUSC patients ID available on TCGA on 07 June 2016 and used for the analysis.	
**Tumor Samples**	**Normal Samples**
TCGA.18.3406.01A.01R.0980.07	TCGA.22.4593.11A.01R.1820.07
TCGA.18.3407.01A.01R.0980.07	TCGA.22.5471.11A.01R.1635.07
TCGA.18.3408.01A.01R.0980.07	TCGA.22.5472.11A.11R.1635.07
TCGA.18.3409.01A.01R.0980.07	TCGA.22.5478.11A.11R.1635.07
TCGA.18.3410.01A.01R.0980.07	TCGA.22.5481.11A.01R.1949.07
TCGA.18.3411.01A.01R.0980.07	TCGA.22.5482.11A.01R.1635.07
TCGA.18.3412.01A.01R.0980.07	TCGA.22.5483.11A.11R.1820.07
TCGA.18.3414.01A.01R.0980.07	TCGA.22.5489.11A.01R.1635.07
TCGA.18.3415.01A.01R.0980.07	TCGA.22.5491.11A.01R.1858.07
TCGA.18.3416.01A.01R.0980.07	TCGA.33.6737.11A.01R.1820.07
TCGA.18.3417.01A.01R.1443.07	TCGA.34.7107.11A.01R.1949.07
TCGA.18.3419.01A.01R.0980.07	TCGA.43.6143.11A.01R.1820.07
TCGA.18.3421.01A.01R.0980.07	TCGA.43.6647.11A.01R.1820.07
TCGA.18.4083.01A.01R.1100.07	TCGA.43.6771.11A.01R.1820.07
TCGA.18.4086.01A.01R.1100.07	TCGA.43.6773.11A.01R.1949.07
TCGA.18.4721.01A.01R.1443.07	TCGA.60.2709.11A.01R.1820.07
TCGA.18.5592.01A.01R.1635.07	TCGA.90.6837.11A.01R.1949.07
TCGA.18.5595.01A.01R.1635.07	
TCGA.21.1070.01A.01R.0692.07	
TCGA.21.1071.01A.01R.0692.07	
TCGA.21.1072.01A.01R.0692.07	
TCGA.21.1075.01A.01R.0692.07	
TCGA.21.1076.01A.02R.0692.07	
TCGA.21.1077.01A.01R.0692.07	
TCGA.21.1078.01A.01R.0692.07	
TCGA.21.1079.01A.01R.0692.07	
TCGA.21.1080.01A.01R.0692.07	
TCGA.21.1081.01A.01R.0692.07	
TCGA.21.1082.01A.01R.0692.07	
TCGA.21.1083.01A.01R.0692.07	
TCGA.21.5782.01A.01R.1635.07	
TCGA.21.5784.01A.01R.1635.07	
TCGA.21.5786.01A.01R.1635.07	
TCGA.21.5787.01A.01R.1635.07	
TCGA.22.0940.01A.01R.0692.07	
TCGA.22.0944.01A.01R.0692.07	
TCGA.22.1002.01A.01R.0692.07	
TCGA.22.1005.01A.01R.0692.07	
TCGA.22.1011.01A.01R.0692.07	
TCGA.22.1012.01A.01R.0692.07	
TCGA.22.1016.01A.01R.0692.07	
TCGA.22.1017.01A.01R.0692.07	

TCGA.22.4591.01A.01R.1201.07	
TCGA.22.4593.01A.21R.1820.07	
TCGA.22.4594.01A.01R.1201.07	
TCGA.22.4595.01A.01R.1201.07	
TCGA.22.4596.01A.01R.1201.07	
TCGA.22.4599.01A.01R.1443.07	
TCGA.22.4601.01A.01R.1443.07	
TCGA.22.4604.01A.01R.1201.07	
TCGA.22.4607.01A.01R.1201.07	
TCGA.22.4613.01A.01R.1443.07	
TCGA.22.5471.01A.01R.1635.07	
TCGA.22.5472.01A.01R.1635.07	
TCGA.22.5473.01A.01R.1635.07	
TCGA.22.5474.01A.01R.1635.07	
TCGA.22.5477.01A.01R.1635.07	
TCGA.22.5478.01A.01R.1635.07	
TCGA.22.5479.01A.31R.1949.07	
TCGA.22.5480.01A.01R.1635.07	
TCGA.22.5481.01A.31R.1949.07	
TCGA.22.5482.01A.01R.1635.07	
TCGA.22.5483.01A.01R.1820.07	
TCGA.22.5485.01A.01R.1635.07	
TCGA.22.5489.01A.01R.1635.07	
TCGA.22.5491.01A.01R.1635.07	
TCGA.22.5492.01A.01R.1635.07	
TCGA.33.4532.01A.01R.1201.07	
TCGA.33.4533.01A.01R.1201.07	
TCGA.33.4538.01A.01R.1201.07	
TCGA.33.4547.01A.01R.1201.07	
TCGA.33.4566.01A.01R.1443.07	
TCGA.33.4582.01A.01R.1443.07	
TCGA.33.4583.01A.01R.1443.07	
TCGA.33.4586.01A.01R.1443.07	
TCGA.33.6737.01A.11R.1820.07	
TCGA.33.6738.01A.11R.1949.07	
TCGA.34.2596.01A.01R.0851.07	
TCGA.34.2600.01A.01R.0851.07	
TCGA.34.2608.01A.02R.0851.07	
TCGA.34.5231.01A.21R.1820.07	
TCGA.34.5232.01A.21R.1820.07	
TCGA.34.5234.01A.01R.1635.07	
TCGA.34.5236.01A.21R.1820.07	
TCGA.34.5239.01A.21R.1820.07	
TCGA.34.5240.01A.01R.1443.07	
TCGA.34.5241.01A.01R.1443.07	
TCGA.34.5927.01A.11R.1820.07	
TCGA.34.5928.01A.11R.1820.07	
TCGA.34.5929.01A.11R.1820.07	
TCGA.34.7107.01A.11R.1949.07	
TCGA.37.3783.01A.01R.1201.07	

TCGA.37.3789.01A.01R.0980.07	
TCGA.37.3792.01A.01R.0980.07	
TCGA.37.4129.01A.01R.1100.07	
TCGA.37.4130.01A.01R.1100.07	
TCGA.37.4132.01A.01R.1100.07	
TCGA.37.4133.01A.01R.1100.07	
TCGA.37.4135.01A.01R.1100.07	
TCGA.37.4141.01A.02R.1100.07	
TCGA.37.5819.01A.01R.1635.07	
TCGA.39.5011.01A.01R.1443.07	
TCGA.39.5016.01A.01R.1443.07	
TCGA.39.5019.01A.01R.1820.07	
TCGA.39.5021.01A.01R.1443.07	
TCGA.39.5022.01A.21R.1820.07	
TCGA.39.5024.01A.21R.1820.07	
TCGA.39.5027.01A.21R.1820.07	
TCGA.39.5028.01A.01R.1443.07	
TCGA.39.5029.01A.01R.1443.07	
TCGA.39.5030.01A.01R.1443.07	
TCGA.39.5031.01A.01R.1443.07	
TCGA.39.5034.01A.01R.1443.07	
TCGA.39.5035.01A.01R.1443.07	
TCGA.39.5036.01A.01R.1443.07	
TCGA.39.5037.01A.01R.1443.07	
TCGA.39.5039.01A.01R.1443.07	
TCGA.43.2578.01A.01R.0851.07	
TCGA.43.2581.01A.01R.0851.07	
TCGA.43.3394.01A.01R.0980.07	
TCGA.43.3920.01A.01R.0980.07	
TCGA.43.5668.01A.01R.1635.07	
TCGA.43.6143.01A.11R.1820.07	
TCGA.43.6647.01A.11R.1820.07	
TCGA.43.6770.01A.11R.1820.07	
TCGA.43.6771.01A.11R.1820.07	
TCGA.43.6773.01A.41R.1949.07	
TCGA.46.3765.01A.01R.0980.07	
TCGA.46.3766.01A.01R.0980.07	
TCGA.46.3767.01A.01R.0980.07	
TCGA.46.3768.01A.01R.0980.07	
TCGA.46.3769.01A.01R.0980.07	
TCGA.46.6025.01A.11R.1820.07	
TCGA.46.6026.01A.11R.1820.07	
TCGA.51.4079.01A.01R.1100.07	
TCGA.51.4080.01A.01R.1100.07	
TCGA.51.4081.01A.01R.1100.07	
TCGA.56.1622.01A.01R.0692.07	
TCGA.56.5897.01A.11R.1635.07	
TCGA.56.5898.01A.11R.1635.07	
TCGA.56.6545.01A.11R.1820.07	
TCGA.56.6546.01A.11R.1820.07	

TCGA.60.2695.01A.01R.0851.07	
TCGA.60.2696.01A.01R.0851.07	
TCGA.60.2698.01A.01R.0851.07	
TCGA.60.2706.01A.01R.0851.07	
TCGA.60.2707.01A.01R.0851.07	
TCGA.60.2708.01A.01R.0851.07	
TCGA.60.2709.01A.21R.1820.07	
TCGA.60.2710.01A.01R.0851.07	
TCGA.60.2711.01A.01R.0851.07	
TCGA.60.2712.01A.01R.0851.07	
TCGA.60.2713.01A.01R.0851.07	
TCGA.60.2714.01A.01R.0851.07	
TCGA.60.2715.01A.01R.0851.07	
TCGA.60.2716.01A.01R.0851.07	
TCGA.60.2719.01A.01R.0851.07	
TCGA.60.2720.01A.01R.0851.07	
TCGA.60.2721.01A.01R.0851.07	
TCGA.60.2722.01A.01R.0851.07	
TCGA.60.2723.01A.01R.0851.07	
TCGA.60.2724.01A.01R.0851.07	
TCGA.60.2725.01A.01R.1201.07	
TCGA.60.2726.01A.01R.0851.07	
TCGA.63.5128.01A.01R.1443.07	
TCGA.63.5131.01A.01R.1443.07	
TCGA.63.6202.01A.11R.1820.07	
TCGA.63.7020.01A.11R.1949.07	
TCGA.63.7021.01A.11R.1949.07	
TCGA.63.7022.01A.11R.1949.07	
TCGA.63.7023.01A.11R.1949.07	
TCGA.66.2727.01A.01R.0980.07	
TCGA.66.2734.01A.01R.0980.07	
TCGA.66.2737.01A.01R.0980.07	
TCGA.66.2742.01A.01R.0980.07	
TCGA.66.2744.01A.01R.0980.07	
TCGA.66.2753.01A.01R.0980.07	
TCGA.66.2754.01A.01R.0980.07	
TCGA.66.2755.01A.01R.0851.07	
TCGA.66.2756.01A.01R.0851.07	
TCGA.66.2757.01A.01R.0851.07	
TCGA.66.2758.01A.02R.0851.07	
TCGA.66.2759.01A.01R.0851.07	
TCGA.66.2763.01A.01R.0851.07	
TCGA.66.2765.01A.01R.0851.07	
TCGA.66.2766.01A.01R.0851.07	
TCGA.66.2767.01A.01R.0851.07	
TCGA.66.2768.01A.01R.0851.07	
TCGA.66.2769.01A.02R.0851.07	
TCGA.66.2770.01A.01R.0851.07	
TCGA.66.2771.01A.01R.0980.07	
TCGA.66.2773.01A.01R.1201.07	

TCGA.66.2777.01A.01R.1201.07	
TCGA.66.2778.01A.02R.0851.07	
TCGA.66.2780.01A.01R.0851.07	
TCGA.66.2781.01A.01R.0851.07	
TCGA.66.2782.01A.01R.0851.07	
TCGA.66.2783.01A.01R.1201.07	
TCGA.66.2785.01A.01R.0851.07	
TCGA.66.2786.01A.01R.0851.07	
TCGA.66.2787.01A.01R.0980.07	
TCGA.66.2788.01A.01R.0980.07	
TCGA.66.2789.01A.01R.0980.07	
TCGA.66.2790.01A.01R.0980.07	
TCGA.66.2791.01A.01R.0980.07	
TCGA.66.2792.01A.01R.0980.07	
TCGA.66.2793.01A.01R.1201.07	
TCGA.66.2794.01A.01R.1201.07	
TCGA.66.2795.01A.02R.0980.07	
TCGA.66.2800.01A.01R.1201.07	
TCGA.70.6722.01A.11R.1820.07	
TCGA.70.6723.01A.11R.1820.07	
TCGA.77.6842.01A.11R.1949.07	
TCGA.77.6843.01A.11R.1949.07	
TCGA.77.6844.01A.11R.1949.07	
TCGA.77.6845.01A.11R.1949.07	
TCGA.79.5596.01A.31R.1949.07	
TCGA.85.6175.01A.11R.1820.07	
TCGA.85.6560.01A.11R.1820.07	
TCGA.85.6561.01A.11R.1820.07	
TCGA.85.6798.01A.11R.1949.07	
TCGA.90.6837.01A.11R.1949.07	
TCGA.94.7033.01A.11R.1949.07	

SUPPLEMENTARY FIGURE 1

Principal component analysis and hierarchical clustering.

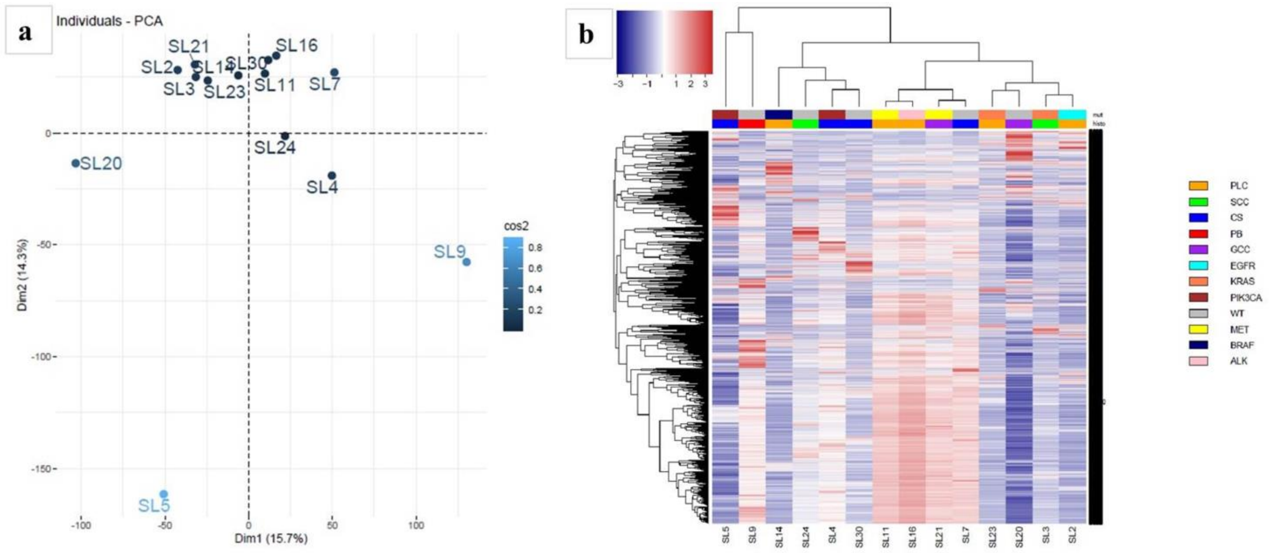

(a)Principal component analysis using transcriptome data; (b)hierarchical clustering using the expression levels of the 10.000 genes with the highest variance.

Samples histotypes: SL20, GCC; SL14, PLC; SL7, CS; SL5, CS; SL4, CS; SL30, CS; SL11, PLC; SL24, SCC; SL23, PLC; SL2, PLC; SL16, PLC; SL9, PB; SL21, GCC; SL3, SCC.

GCC, giant cell carcinoma; PLC, pleomorphic carcinoma; CS, carcinosarcoma; SCC, spindle cell carcinoma; PB, pulmonary blastoma.
